# Further Developments and Applications of Oxazoline-Containing Ligands in Asymmetric Catalysis

**DOI:** 10.1021/acs.chemrev.0c00844

**Published:** 2021-05-21

**Authors:** Robert Connon, Brendan Roche, Balaji V. Rokade, Patrick J. Guiry

**Affiliations:** † Synthesis and Solid State Pharmaceutical Centre, Centre for Synthesis and Chemical Biology, School of Chemistry, University College Dublin, Dublin 4, Ireland; ‡ BiOrbic Research Centre, Centre for Synthesis and Chemical Biology, School of Chemistry, University College Dublin, Dublin 4, Ireland

## Abstract

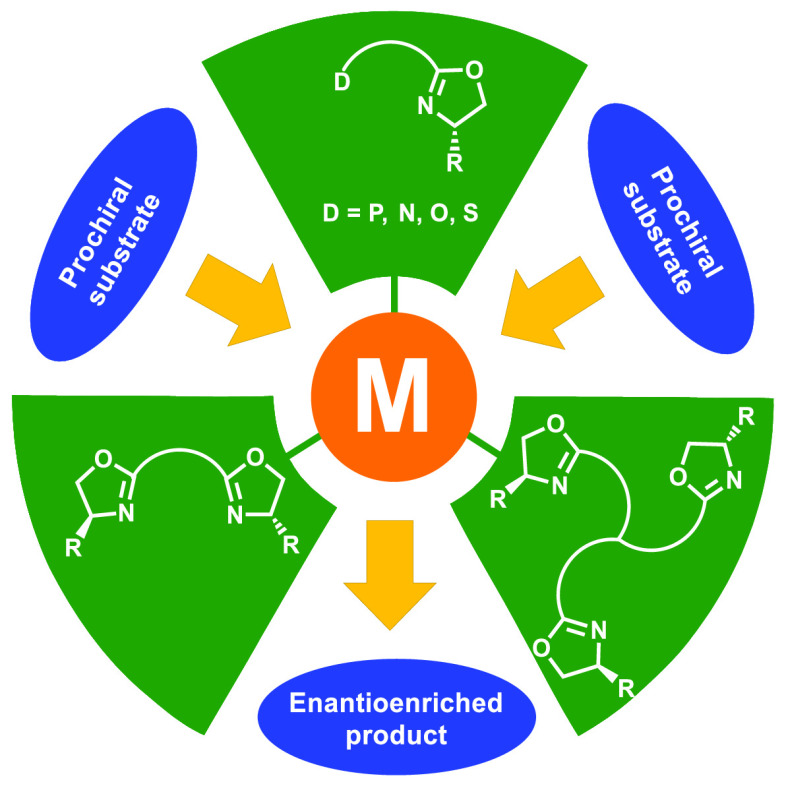

The chiral oxazoline motif is present in many ligands that have been extensively applied in a series of important metal-catalyzed enantioselective reactions. This Review aims to provide a comprehensive overview of the most significant applications of oxazoline-containing ligands reported in the literature starting from 2009 until the end of 2018. The ligands are classified not by the reaction to which their metal complexes have been applied but by the nature of the denticity, chirality, and donor atoms involved. As a result, the continued development of ligand architectural design from mono(oxazolines), to bis(oxazolines), to tris(oxazolines) and tetra(oxazolines) and variations thereof can be more easily monitored by the reader. In addition, the key transition states of selected asymmetric transformations will be given to illustrate the features that give rise to high levels of asymmetric induction. As a further aid to the reader, we summarize the majority of schemes with representative examples that highlight the variation in % yields and % *ee*s for carefully selected substrates. This Review should be of particular interest to the experts in the field but also serve as a useful starting point to new researchers in this area. It is hoped that this Review will stimulate both the development/design of new ligands and their applications in novel metal-catalyzed asymmetric transformations.

## 1 Introduction

Ligands containing a chiral oxazoline are some of the most successful, versatile, and commonly used ligand classes in asymmetric catalysis due to their ready accessibility, modular nature, and applicability in a wide range of metal-catalyzed transformations.

The vast majority of these ligands are formed in short, high yielding synthesis from readily available chiral β-amino alcohols. Therefore, the stereocenter controlling the enantioselectivity of the metal-catalyzed process resides α- to the oxazolinyl nitrogen donor and, as a result, is in close proximity to the metal active site to directly influence the asymmetry induced in the reaction.

**Figure 1:**
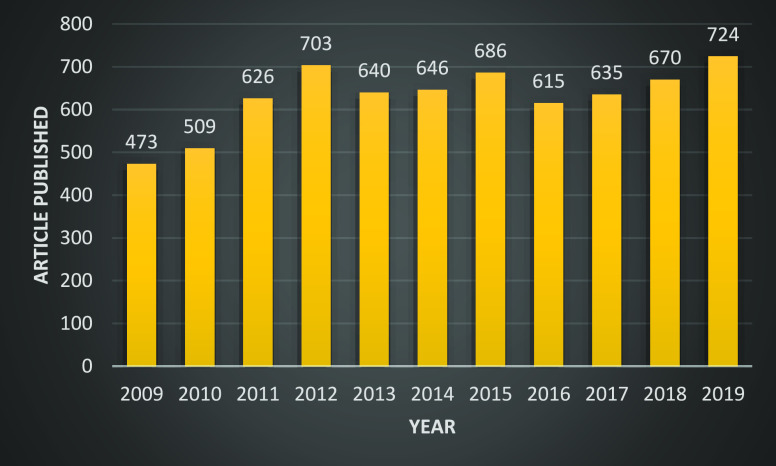
SciFinder data on oxazoline-related research for the years 2009 to 2019.

Since the first report of chiral oxazoline-based ligands in asymmetric catalysis in 1986, an enormous range of ligands containing one, two, three or four oxazolines incorporating various heteroatoms, additional chiral elements and other specific structural features have been subsequently developed with great success in a significant range of asymmetric reactions. SciFinder data for the years 2009 to 2019 confirm the significance of research related to the term “oxazoline” (Figure 1).

This review reports on the use of such ligands in homogeneous metal-catalyzed asymmetric synthesis since 2009, when the area was last reviewed by us.^1^ This review does not cover the application of such ligands in heterogeneous systems or in the synthesis of polymers. We cover, in our view, the most significant applications of oxazoline-containing ligands reported in the literature until the end of 2018. This review will be structured in the same manner as our 2004^2^ and 2009 reviews as we classify ligands, not by the reaction to which their metal complexes have been applied, but by the nature of the denticity, chirality and donor atoms involved. As a result, the continued development of ligand architectural design can be more easily monitored. In addition, the key transition states of selected asymmetric transformations employing metal complexes of oxazoline ligands will be given to illustrate the features that give rise to high levels of asymmetric induction. As a further aid to the reader, we summarize the majority of schemes with representative examples which highlight the variation in % yields and % *ee*s for carefully selected substrates.

## 2 Mono(oxazoline) Ligands

### 2.1 Mono(oxazoline) *P*,*N*-Ligands (Phosphinooxazoline)

#### 2.1.1 Phosphinooxazoline Ligands with One Stereocenter

**Figure 2:**
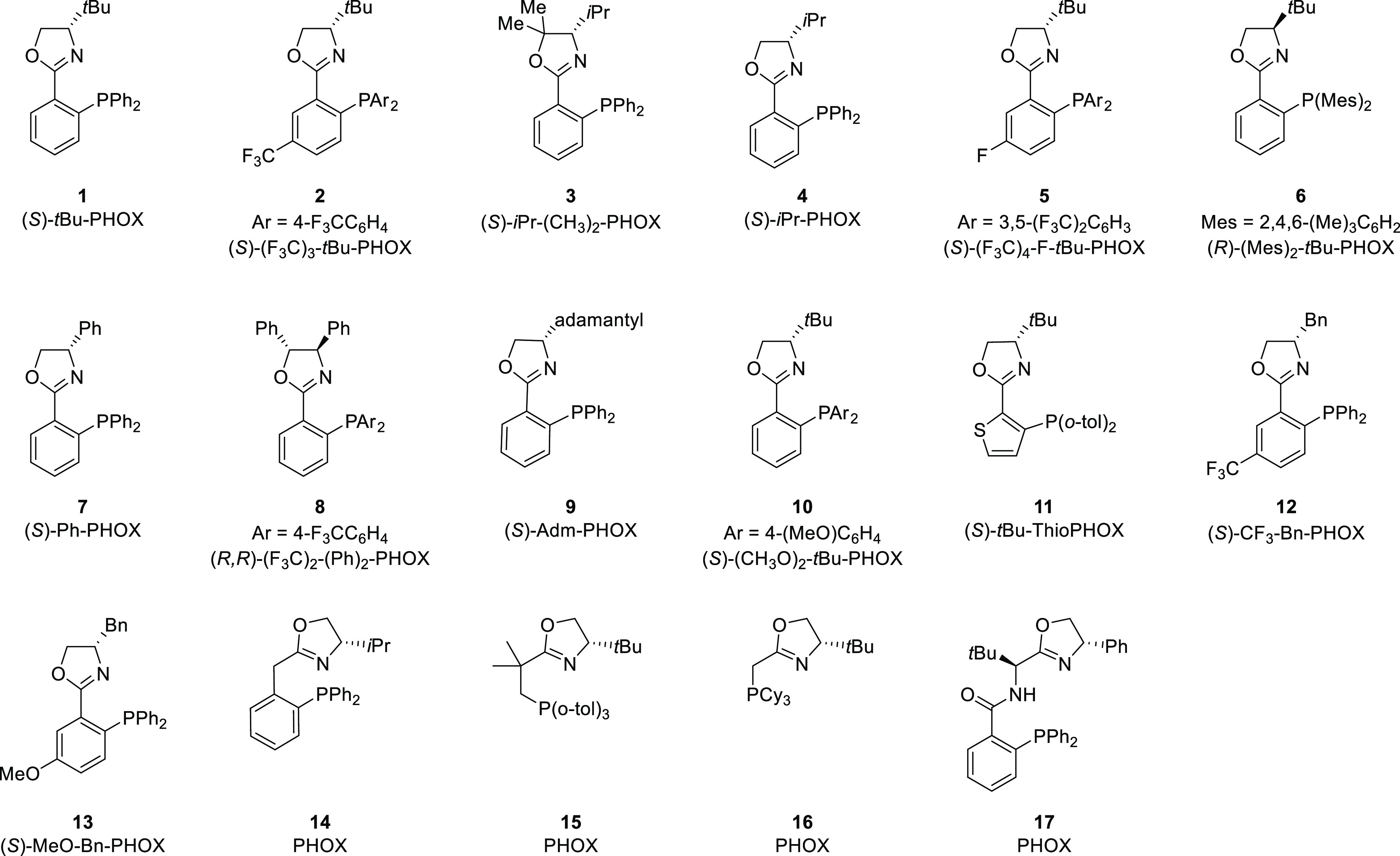
Various phosphinooxazoline (PHOX) ligands

Phosphinooxazoline (PHOX) is a popular class of bidentate ligand in which the chiral oxazoline moiety is solely responsible for asymmetric induction in product formation (Figure 2).^3−6^ In this section, various PHOX ligands (**1**–**17**) and their applications in a broad range of asymmetric transformations will be discussed. This section is further divided into subsections based on the type of reactions the metal complexes of PHOX ligands have been used for.

##### 2.1.1.1 Applications of PHOX in Asymmetric Allylation

**Scheme 1:**
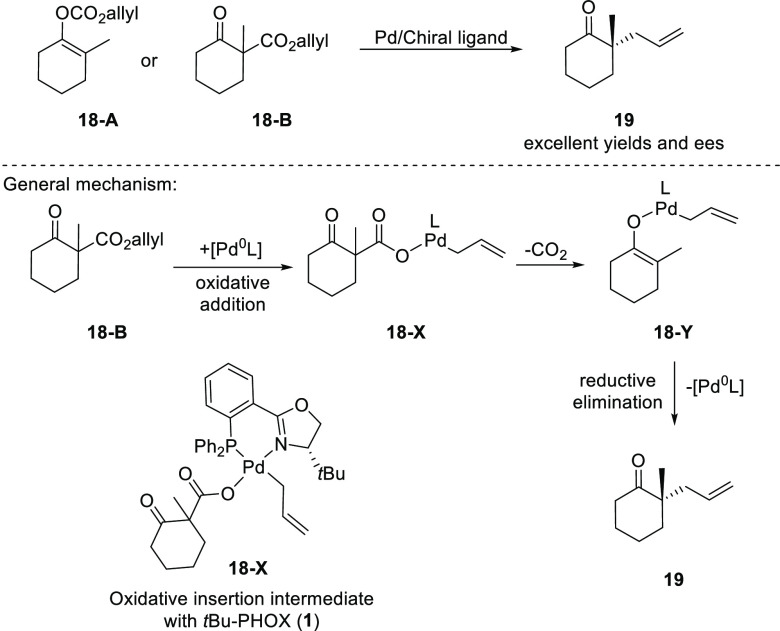
Decarboxylative Asymmetric Allylic Alkylation (DAAA)

In late 2004, Stoltz and Trost independently developed a Pd-catalyzed decarboxylative asymmetric allylic alkylation (DAAA) reaction using *t*Bu-PHOX (**1**) and Trost-type ligands, respectively (Scheme 1).^7,8^ The generally accepted mechanism of the DAAA involves the coordination of the Pd(0) complex to the allyl moiety leading to oxidative addition, followed by the loss of CO_2_ to generate the Pd enolate **18-Y**, which then attacks the intermediate allyl group to form the enantioenriched product **19** and regenerate the Pd(0) catalyst.^9^

Since the seminal report from Stoltz, the scope and application of PHOX ligand in DAAA has been expanded to different substrate classes and also in natural products synthesis.

**Scheme 2:**
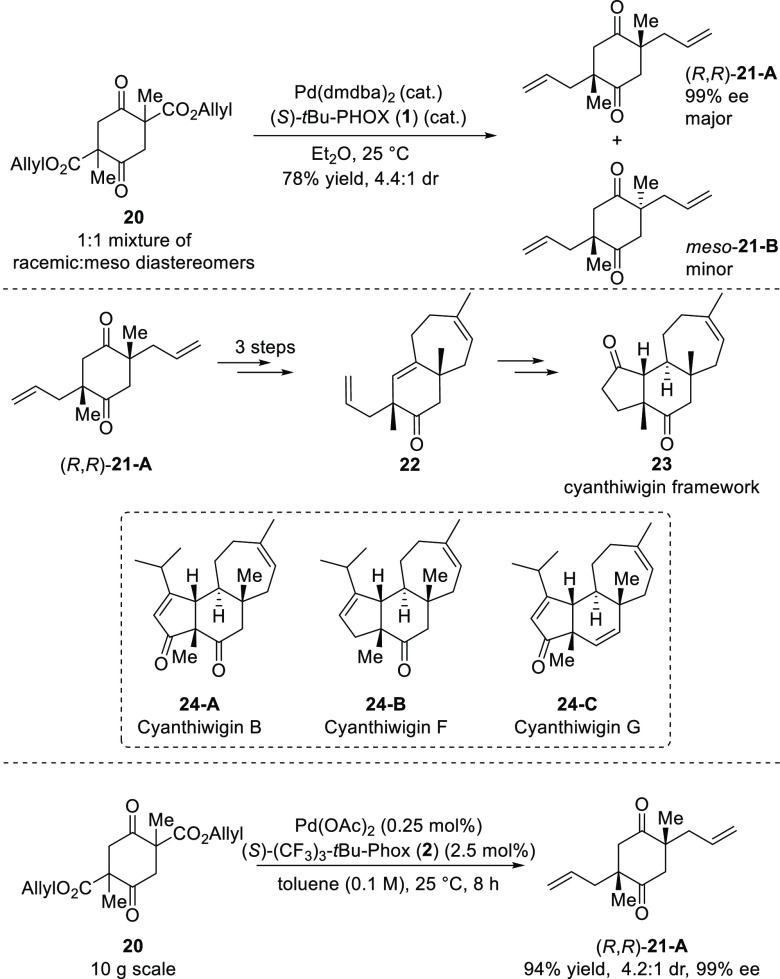
Catalytic Asymmetric Synthesis of Tricyclic Cyanthiwigin Framework

In 2008, Stoltz described a concise and versatile strategy for the preparation of the natural products, cyanthiwigin B, F and G by using a key double stereoablative decarboxylative asymmetric allylic alkylation of bis(β-keto ester) **20** (Scheme 2).^10,11^ Their strategy involved the synthesis of the central six-membered ring, cyclohexadione **21**, with a special emphasis on the early installation of two of the most critical stereocenters of the cyanthiwigin framework in good yield with a high level of stereoselectivity (4.4:1 dr and 99% *ee*). The major diastereomer of cyclohexadione (*R*,*R*)-**21-A** was subsequently converted for the efficient synthesis of cyanthiwigin B (**24-A**), F (**24-B**), and G (**24-C**). It is worthwhile noting that the observed selectivity in the double decarboxylative allylation with the (*S*)-*t*Bu-PHOX (**1**) ligand was excellent given the fact that the reaction begins with a complicated mixture of racemic and *meso*-diastereomers which leads to several possible stereochemical outcomes, and pathways.

The key double catalytic enantioselective alkylation was troublesome on a large scale due to the poor solubility of the catalyst which required low reaction concentrations (0.01 M). This problem was improved significantly in their subsequent report wherein the enantioselective alkylation was carried out on a 10 g scale furnishing the desired diketone (*R*,*R*)-**21-A** in 94% yield, 4.2:1 dr and 99% *ee*.^12^

**Scheme 3:**
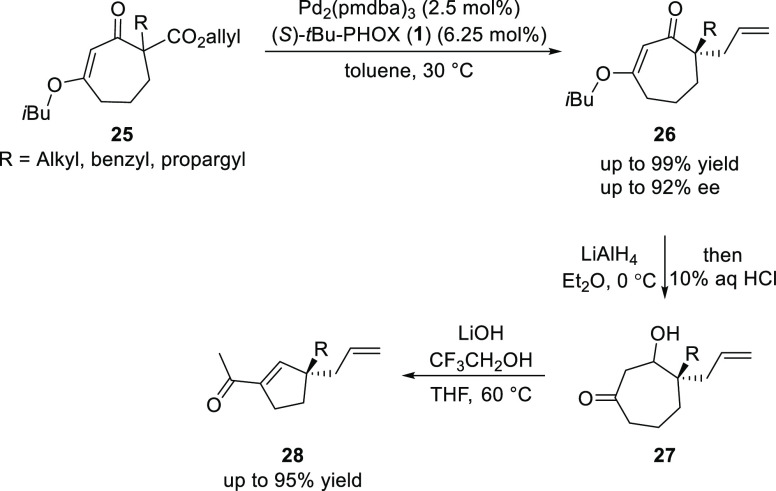
Asymmetric Synthesis of Densely Functionalized Acylcyclopentenes

Stoltz extended DAAA to seven membered cyclic β-keto-allyl ester (**25**) to generate allylated products (**26**), which were further employed in the asymmetric synthesis of densely functionalized acylcyclopentenes (**28**), valuable intermediates for the synthesis of natural products, in excellent yields (up to 99%) and high enantioselectivities (up to 92% *ee*) (Scheme 3).^13^ The synthesis of **28** from **25** involves a Pd-catalyzed decarboxylative asymmetric allylic alkylation followed by a two-carbon ring contraction.

**Scheme 4:**
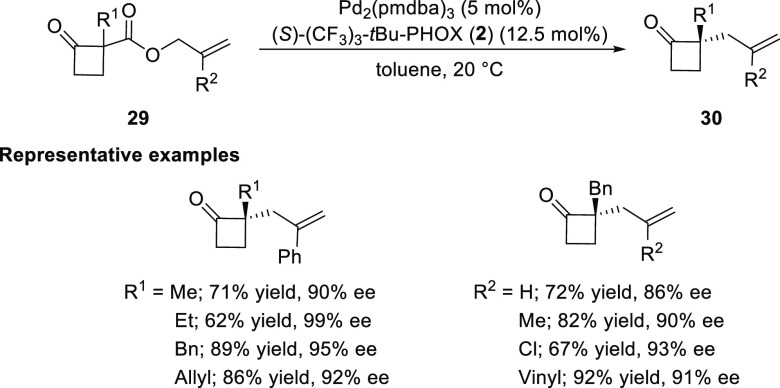
Enantioselective α-Alkylation of Cyclobutanones

Later in 2013, Stoltz reported a Pd-catalyzed enantioselective α-alkylation to cyclobutanones (**30**). The palladium complex of an electron-deficient (*S*)-(CF_3_)_3_-*t*Bu-PHOX (**2**) ligand demonstrated excellent catalytic activity to afford α-quaternary cyclobutanones in good to excellent yields and enantioselectivities (Scheme 4).^14^ A wide variety of substituents were compatible at both the α-keto and 2-allyl positions especially considering the presence of highly electrophilic cyclobutanones. Furthermore, chiral cyclobutanones were converted into dialkyl γ-lactams, dialkyl γ-lactones, α-quaternary cyclopentanones, and quaternary [4.5]-spirocycles.

**Scheme 5:**
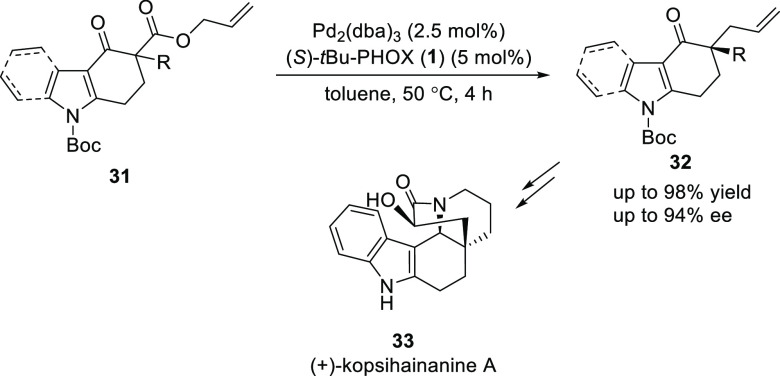
Catalytic Decarboxylative Asymmetric Allylation of Carbazolones and Indolones

In 2013, Lupton reported a Pd-catalyzed decarboxylative asymmetric allylation for the enantioselective synthesis of carbazolone and indolone heterocycles (**32**) (Scheme 5).^15^ A variety of carbazolones and indolones containing a quaternary carbon center were synthesized in excellent yields (up to 98%) and enantioselectivities (up to 94% *ee*). Moreover, the application of this methodology was shown in the synthesis of (+)-kopsihainanine (**33**).

**Scheme 6:**
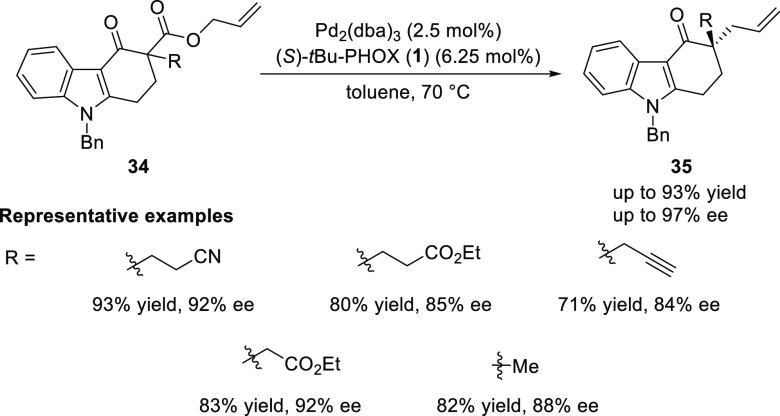
Catalytic Decarboxylative Asymmetric Allylation of Carbazolones

In 2013, Shao reported the Pd-catalyzed enantioselective decarboxylative allylic alkylation of carbazolone heterocycles (**34**) (Scheme 6).^16^ A variety of highly functionalized chiral carbazolones (**35**) featuring an α-quaternary carbon center were synthesized in good yields (up to 93%) with high levels of enantioselectivity (up to 97% *ee*).

**Scheme 7:**
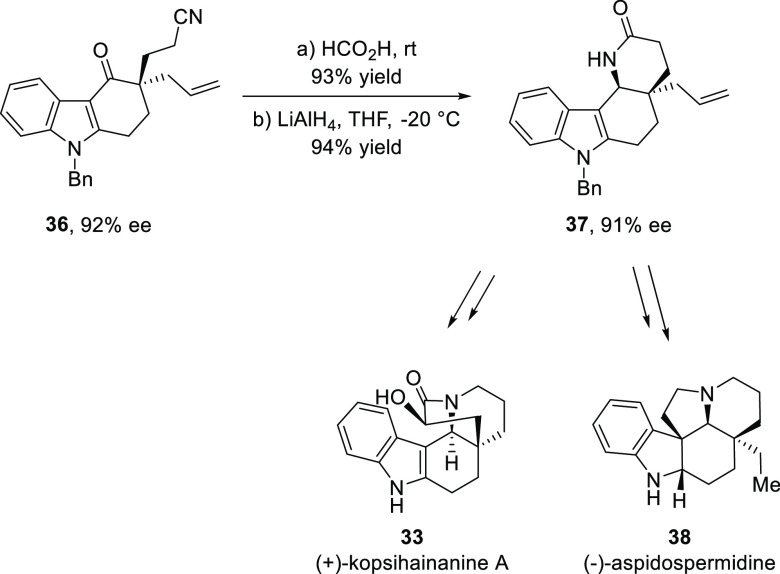
Catalytic Asymmetric Synthesis of (−)-Aspidospermidine and (+)-Kopsihainanine A

Using this methodology, a catalytic asymmetric strategy for the synthesis of (+)-kopsihainanine A (**33**) and (−)-aspidospermidine (**38**) was accomplished from a common intermediate (Scheme 7).

**Scheme 8:**
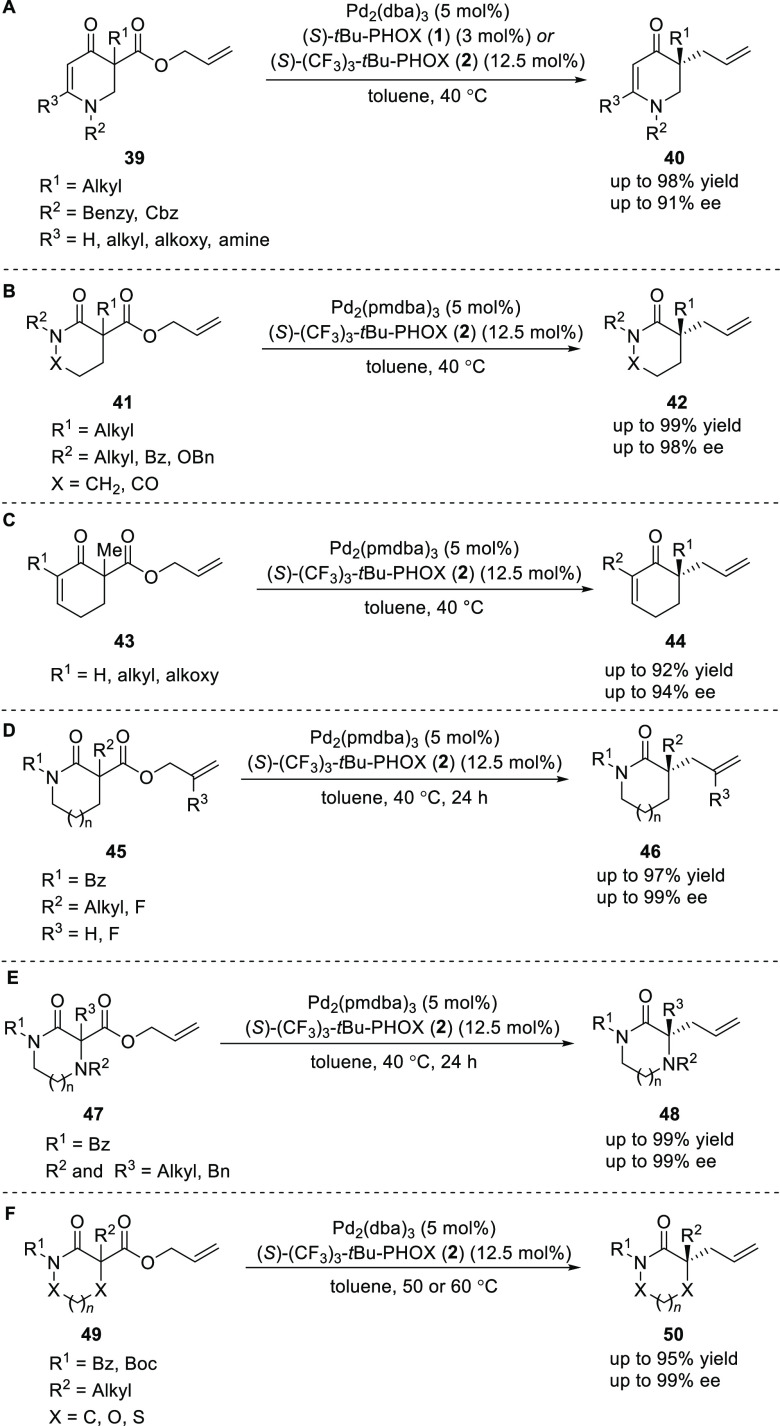
Decarboxylative Asymmetric Allylic Alkylation

Stoltz in 2013 reported a Pd-catalyzed decarboxylative allylic alkylation of novel allyl ester substrates (**39**, **41**, and **43**) to probe the influence of enolate electronics and the role of α-functionality on the selectivity (Scheme 8A–C).^17^ It was observed that the high enantioselectivities obtained with imides and lactams are due to both electronic and steric effects associated with the α-substituent, and the enolate electronics alone contribute comparatively less to the stereochemical outcome of the reaction.

In 2012, Stoltz reported the highly enantioselective Pd-catalyzed decarboxylative allylic alkylation of readily available lactams (**45**) to form 3,3-disubstituted pyrrolidinones, piperidinones, caprolactams and structurally related lactams (**46**) (Scheme 8D).^18^ This method was employed for the catalytic asymmetric synthesis of key intermediates previously used for the construction of *Aspidosperma* alkaloids quebrachamine and rhazinilam.

In 2015, Stoltz reported the synthesis of α-tertiary piperazin-2-ones (**48**) by a Pd-catalyzed decarboxylative allylic alkylation (Scheme 8E).^19^ This method employed a more electron-rich Pd catalyst, [Pd_2_(pmdba)_3_] and an electron-deficient PHOX ligand to afford products in good to excellent yields and enantioselectivities. Additionally, this method also allows for the synthesis of α-secondary piperazin-2-ones in modest to excellent yields and good to excellent enantioselectivities. A variety of substituents at nitrogen and also at the stereocenter were tolerated under the reaction conditions employed.

In 2015, Stoltz developed a method to synthesize α,α-disubstituted *N*-heterocyclic carbonyl compounds (**50**), by using the well-established DAAA reaction (Scheme 8F).^20^ Various heterocycles including morpholinone, thiomorpholinone, oxazolidin-4-one, 1,2-oxazepan-3-one, and 1,3-oxazinan-4-one performed well to furnish carbonyl products with fully a substituted stereocenter at the α-position. The presence of an electron-deficient *N*-substituent was required for high reactivity and enantioselectivity.

**Scheme 9:**
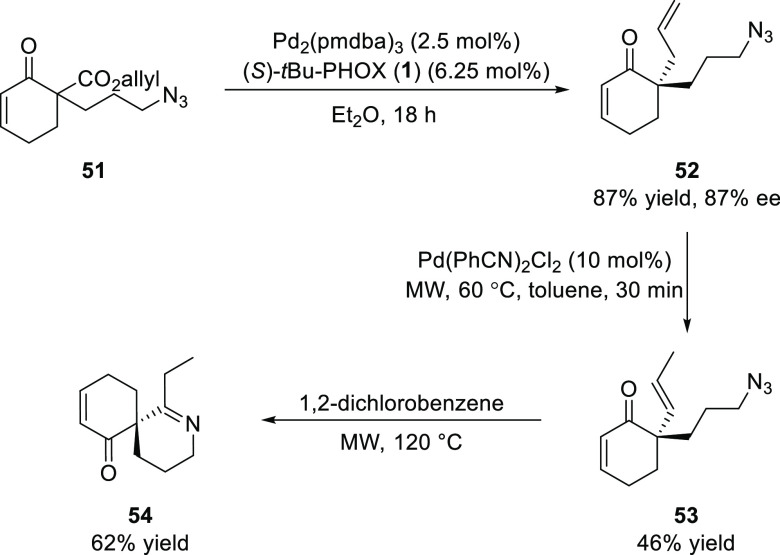
Enantioselective Synthesis of Spiroimines

Guillou reported a method to access enantioenriched spiroimines (**54**) using Pd-catalyzed decarboxylative allylic alkylation (Scheme 9).^21^ A variety of cyclic ketones (**52**) having α-allyl, propyl or butyl azido groups were synthesized in moderate to good enantioselectivity which, upon isomerization of the allyl group followed by a [3 + 2]-cycloaddition of the azidoalkene, afforded a variety of chiral spiroimines.

**Scheme 10:**
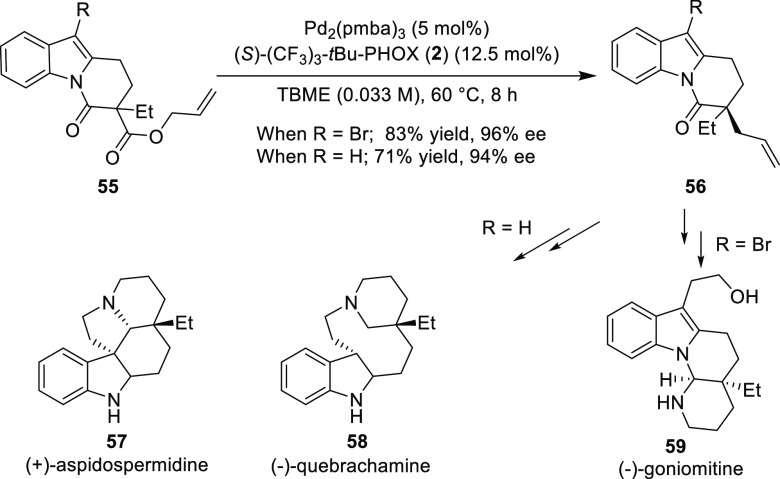
Catalytic Asymmetric Total Synthesis of (−)-Goniomitine, (+)-Aspidospermidine, and (−)-Quebrachamine

In 2016, Stoltz reported the first catalytic asymmetric total synthesis of (−)-goniomitine (**59**) starting from indole in 11 steps with an 8% overall yield (Scheme 10).^22^ The important step during this synthesis involves the Pd-catalyzed decarboxylative allylic alkylation of rationally designed heteroaryl bromide **55** furnishing product in 83% yield and 96% *ee*. Additionally, the formal synthesis of (+)-aspidospermidine (**57**) and (−)-quebrachamine (**58**) was also demonstrated.

**Scheme 11:**
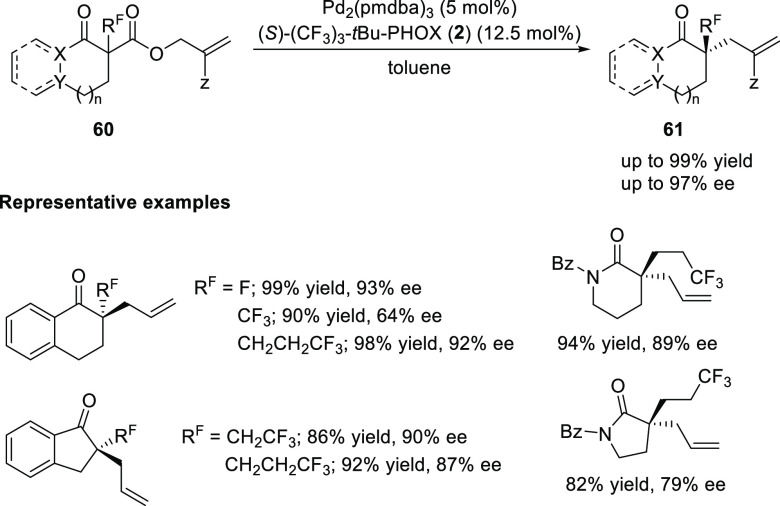
Enantioselective Synthesis of Fluorine-Containing Tetra-Substituted Stereocenters

In 2018, Stoltz reported a strategy to construct fluorine-containing tetra-substituted stereocenters by the enantioselective Pd-catalyzed decarboxylative allylic alkylation of various carbonyl compounds (**60**) (Scheme 11).^23^ An electron-deficient (*S*)-(CF_3_)_3_-*t*Bu-PHOX (**2**) ligand was optimal and this reaction showed significant substituent tolerance as a variety of five- and six-membered ketones and lactams (**60**) afforded the corresponding products with good yields and enantioselectivities.

**Scheme 12:**
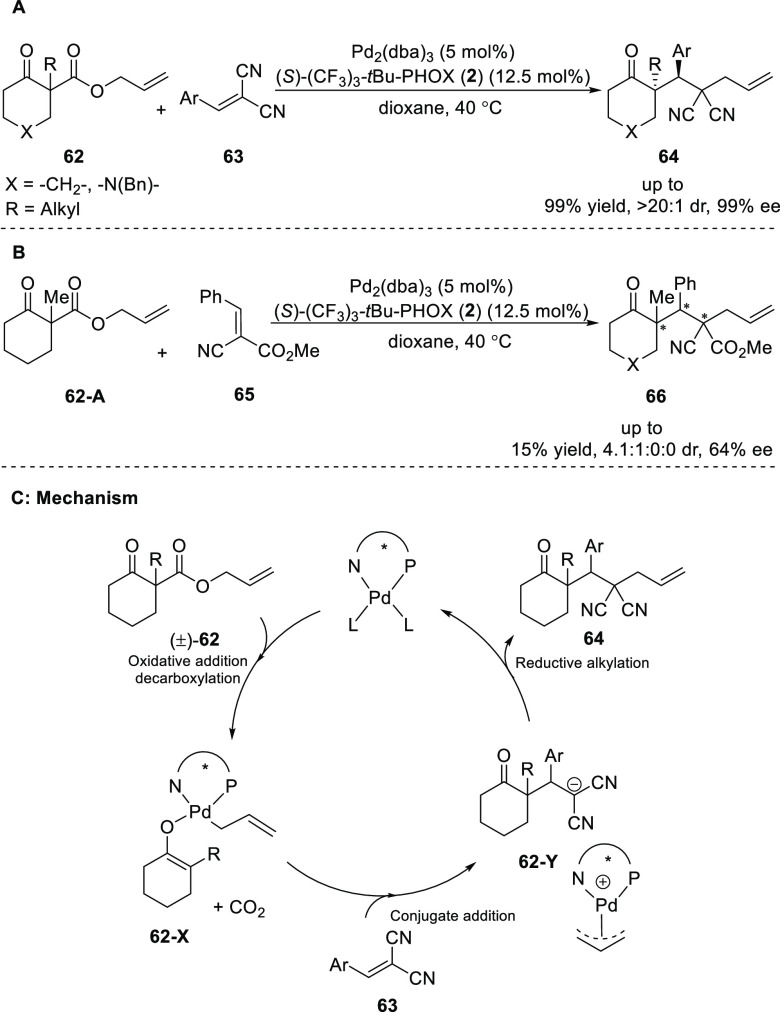
Enantioselective Decarboxylative Enolate Alkylation Cascade

In 2010, Stoltz devised a Pd-catalyzed highly enantio- and diastereoselective α-alkylation cascade protocol (Scheme 12A).^24^ This method allowed for the installation of vicinal quaternary and tertiary stereocenters in a single step. Cyclohexanone based β-keto-ally esters **62** and arylidene malononitrile **63** were suitable substrates and the desired products **64** were formed in up to 99% *ee* and with *a* > 20:1 dr. Small alkyl groups (R) were tolerated best in the reaction, although the use of electron-donating substituents on the arylidene malononitrile gave rise to lower yields. The installation of three stereocenters was also attempted by employing α-nitrile esters **65** (Scheme 12B). However, using a palladium complex of the chiral ligand (CF_3_)_3_-*t*-Bu-PHOX (**2**) in the reaction gave a poor yield of 15% and 64% *ee*. The reaction proceeds via the decarboxylation of β-keto-allyl ester (**62**) to generate a Pd-enolate of cyclohexanone (**62-X**), followed by a conjugate addition to a prochiral activated Michael acceptor which generates densely functionalized molecules (**64**) that possess a quaternary stereocenter next to a tertiary center (Scheme 12C).

**Scheme 13:**
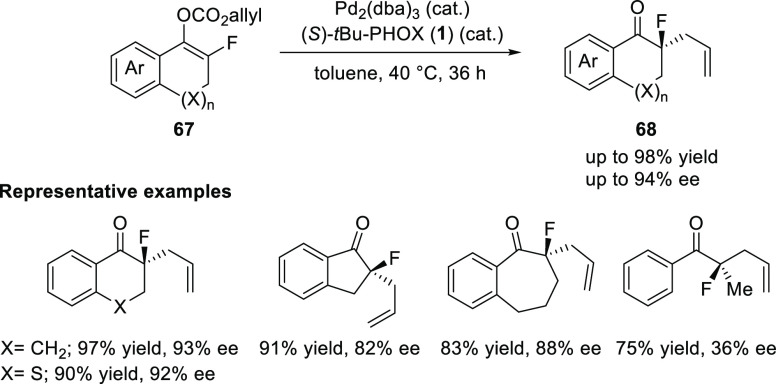
Enantioselective Decarboxylative α-Allylation of Fluorinated Allyl Enol Carbonates

In 2008, Paquin detailed a Pd-catalyzed enantioselective decarboxylative allylic alkylation of fluorinated allyl enol carbonates (**67**) (Scheme 13).^25^ They discovered an important and unusual effect of the L/Pd ratio on the enantioselectivity, whereby a L/Pd ratio of 1:1.67 (and as low as 1:4) is required to afford the α-fluoroketones (**68**) in high enantioselectivity (up to 94% *ee*).

**Scheme 14:**
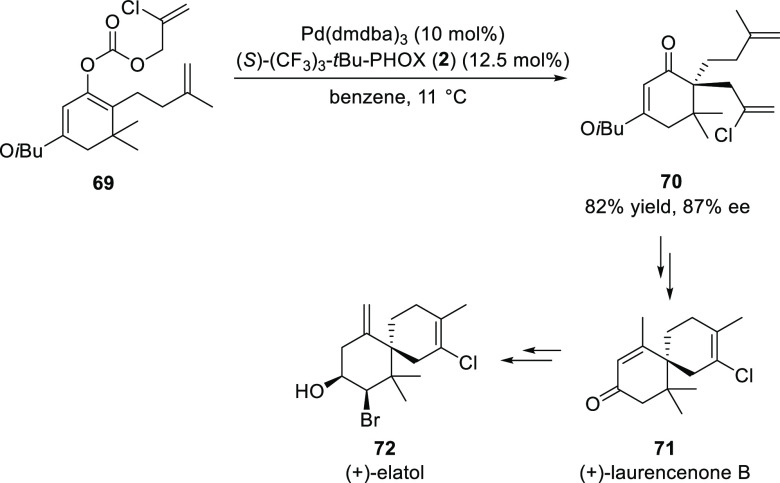
Enantioselective Synthesis of Elatol and (+)-Laurencenone B

In 2008, Stoltz devised a concise enantioselective strategy for the syntheses of elatol (**72**) and (+)-laurencenone B (**71**) (Scheme 14).^26^ The key Pd-catalyzed decarboxylative enantioselective alkylation was employed to access sterically encumbered enantioenriched vinylogous esters.

**Scheme 15:**
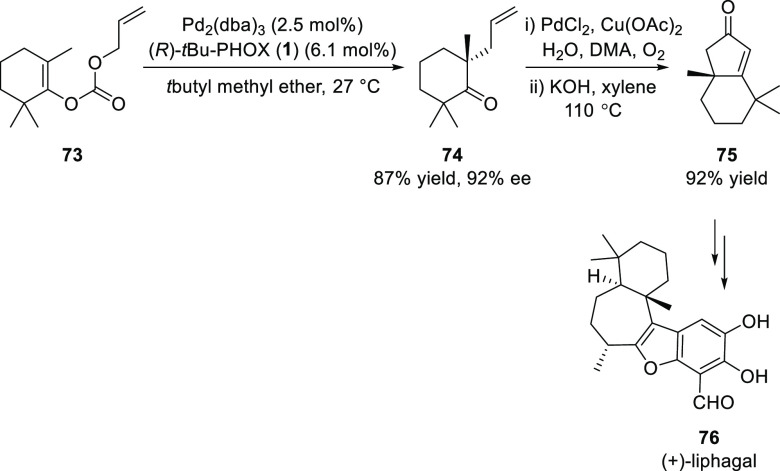
Catalytic Enantioselective Total Synthesis of (+)-Liphagal

In 2011, Stoltz completed the catalytic enantioselective total synthesis of (+)-liphagal (**76**) in 19 overall steps from commercially available starting materials (Scheme 15).^27^ The key step involves a Pd-catalyzed decarboxylative allylic alkylation of enol carbonate **73** to furnish tetra-substituted ketone **74** in 87% yield and 92% *ee*. This intermediate was elaborated to bicycle **75** in a two-step sequence which was further used for the completion of the first total synthesis of (+)-liphagal.

**Scheme 16:**
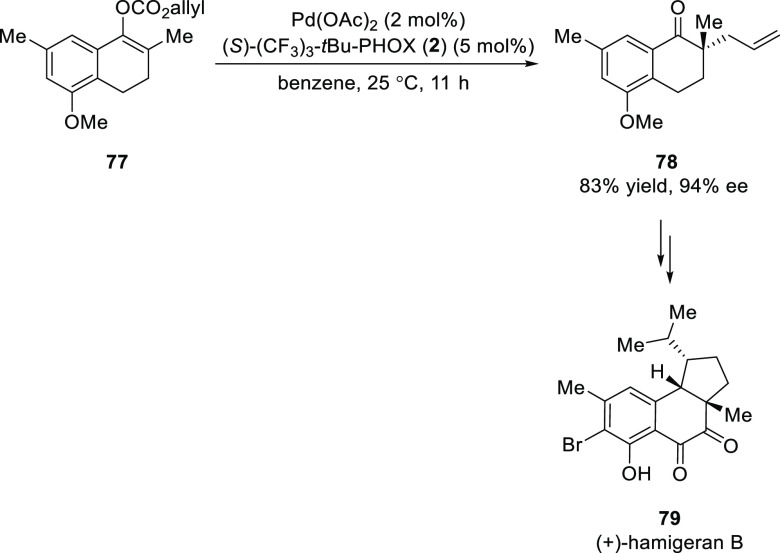
Formal Synthesis of (+)-Hamigeran B

In 2011, Stoltz developed a formal synthesis of (+)-hamigeran B **79** starting from tetralone **77** using a key Pd-catalyzed enantioselective decarboxylative allylic alkylation (Scheme 16).^28^ The other key reaction involves a Ru-mediated cross-metathesis with methyl vinyl ketones and a CuH-mediated domino conjugate reduction-cyclization.

**Scheme 17:**
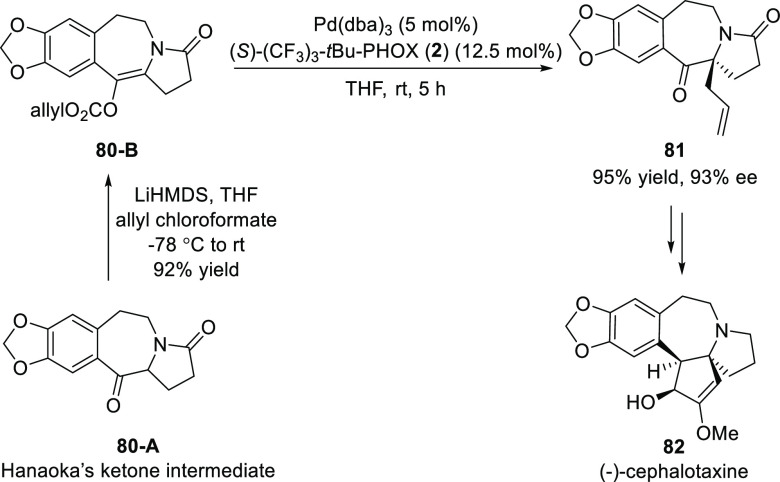
Catalytic Asymmetric Synthesis of (−)-Cephalotaxine

In 2018, Zhang reported a catalytic asymmetric synthesis of (−)-cephalotaxine **82** using a Pd-catalyzed Tsuji allylation (Scheme 17).^29^ The synthesis involved 15 steps starting from homopiperonylamine with a 6.2% overall yield. The key allyl enol carbonate precursor **80-B** was prepared from the classic Hanaoka’s pyrrolobenzazepine intermediate (**80-A**).

**Scheme 18:**
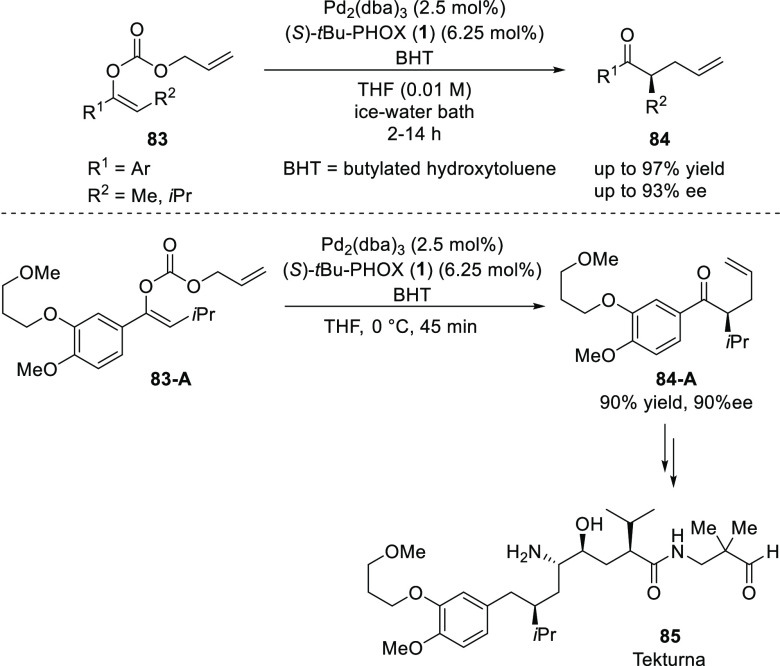
Application of DAAA for Synthesis of Tekturma

In 2012, Hanessian reported a new approach for the synthesis of tekturma (**85**), a drug used in the treatment of hypertension. This approach involves a modified Stoltz Pd-catalyzed decarboxylative asymmetric allylation (Scheme 18).^30^ A remarkable effect of BHT (2,6-di-*t*Bu-*p*-cresol) as a protic additive on the reaction time and enantioselectivity was discovered. The alkyl aryl enol carbonate (**83-A**) was converted into intermediate, (*R*)-α-isopropyl allyl aryl ketone (**84-A**) in 90% yield and 90% *ee* which was used for the synthesis of tekturna **85**.

**Scheme 19:**
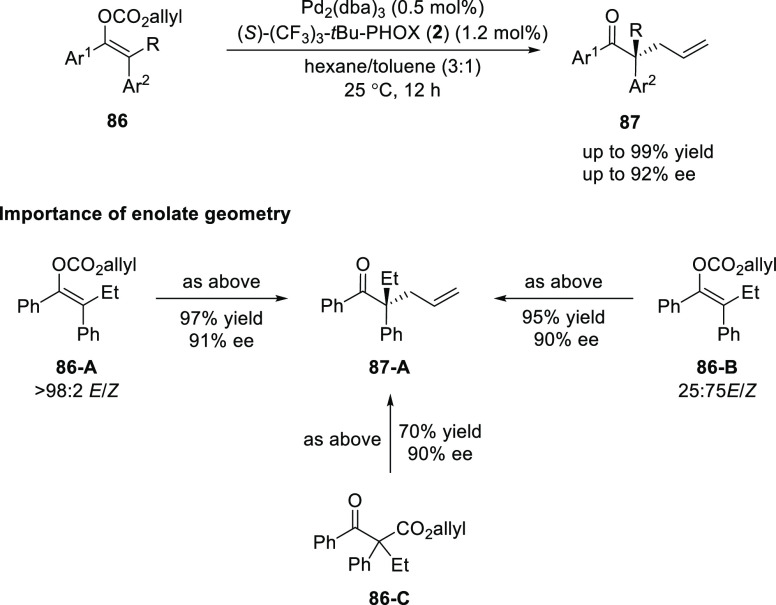
DAAA of Fully Substituted Acyclic Enol Carbonates

In 2018, Stoltz reported the first enantioselective Pd-catalyzed decarboxylative allylic alkylation of fully substituted acyclic enol carbonates (**86**) providing linear α-quaternary ketones (**87**) (Scheme 19).^31^ High yields and enantiomeric excesses of the product were achieved using the electron-deficient ligand, (*S*)-(CF_3_)_3_-*t*Bu-PHOX (**2**). Previous reports in Pd-catalyzed allylic alkylation suggested differing selectivities with different enolate geometries but here it was found that the enolate geometry of the starting material has no adverse effect as the same enantiomer of the product was obtained with the same level of selectivity regardless of the starting ratio of enolates. It was postulated that a dynamic kinetic resolution of the two enolate geometries occurs in the reaction possibly due to facile equilibration between *O*-bound and *C*-bound Pd enolates in the presence of the electron-deficient PHOX ligand.

**Scheme 20:**
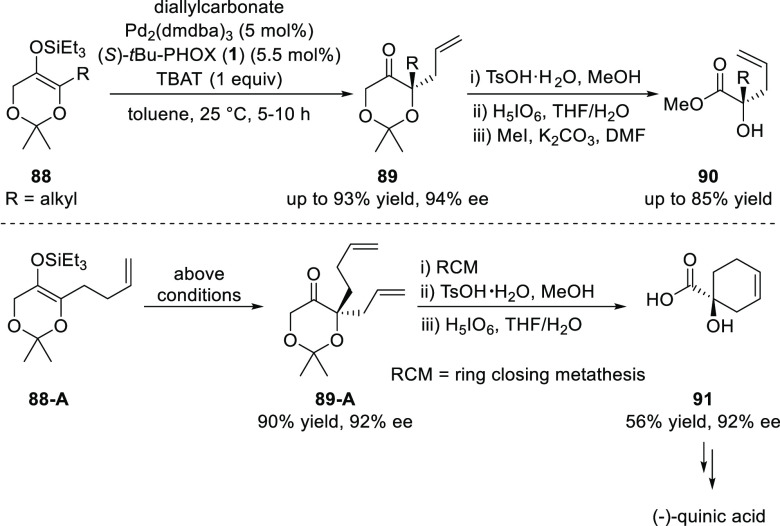
DAAA of Dioxanone Derivatives

In 2008, Stoltz developed a Pd/(*S*)-*t*Bu-PHOX-catalyzed asymmetric alkylation of simple dioxanone derivatives (**88**) to obtain enantioenriched tetra-substituted dioxanone **89** possessing allyl group at α-position (Scheme 20).^32^ The enantioenriched tetra-substituted dioxanone subsequently was transformed into the enantioenriched α-hydroxyketones, acids, and esters via a three-step sequence which involved acetonide cleavage catalyzed by *p*-toluenesulfonic acid (TsOH·H_2_O), oxidative cleavage with periodic acid (HIO_4_) and finally methylation of the acid moiety. Additionally, this procedure allowed a catalytic enantioselective formal synthesis of (−)-quinic acid, a useful chiral building block.

**Scheme 21:**
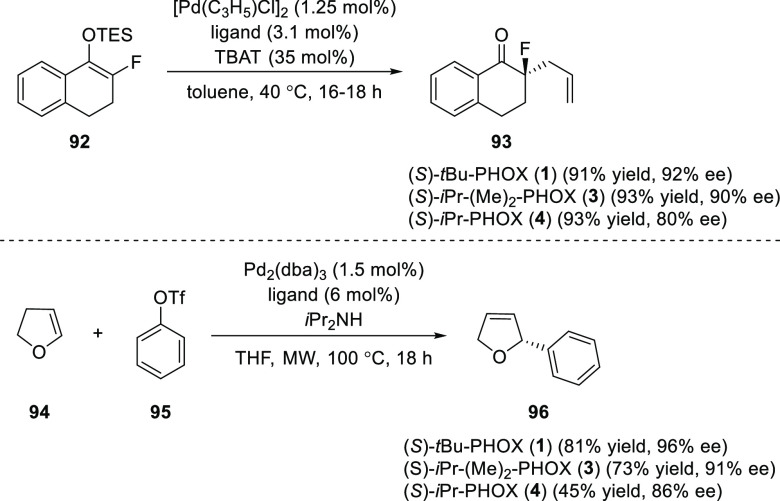
Enantioselective Allylation of Fluorinated Silyl Enol Ether and Heck Reaction

Paquin reported a new member of the PHOX family, 5,5-(dimethyl)-*i*Pr-PHOX (**3**), having parallel reactivity to (*S*)-(CF_3_)_3_-*t*Bu-PHOX (**2**) with the key advantage of being easily accessible as both enantiomers (Scheme 21).^33^ The application of ligand was carried out in the enantioselective allylation of fluorinated silyl enol ether (**92**) and the enantioselective Heck reaction between 2,3-dihydrofuran (**94**) and phenyl triflate (**95**). It was observed that the new ligand’s reactivity was as good as (*S*)-*t*Bu-PHOX (**1**) and better than the (*S*)-*i*Pr-PHOX (**4**), which lacks the *gem*-dimethyl at C-5, thus demonstrating the beneficial effect of the substituents at C-5.

**Scheme 22:**
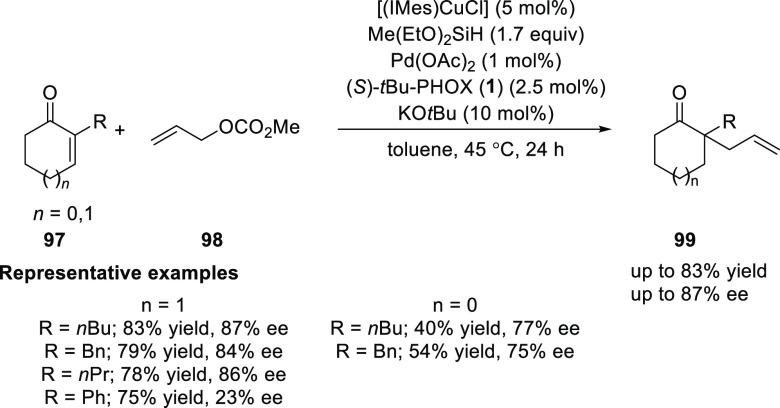
Asymmetric Cooperative Dual Catalysis of α-Substituted Cyclic Enones

Riant reported a cooperative dual catalysis based on a Pd(0)/Cu(I) system to generate quaternary carbon stereocenters (Scheme 22).^34^ In this domino process, the first reaction involves a 1,4 reduction of cyclic enones using Cu(I) hydride to generate the Cu enolate species *in situ* which further reacts with the π-allyl-Pd complex to form the desired allylation product in an asymmetric fashion. The role of KO*t*Bu as an additive was crucial to increase the enantioselectivity. This method showed tolerance toward alkyl and benzyl groups at the α-position of enones furnishing products in comparable yields and enantioselectivities. The presence of the phenyl group at the α-position of enones was also tolerated to give the expected products in good yield, but unfortunately with low levels of enantioselectivity.

**Scheme 23:**
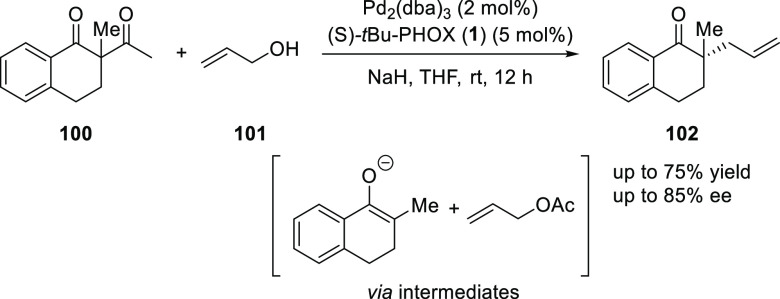
Asymmetric Deacylative Allylation

Tunge reported a method for the asymmetric allylic alkylation of α-tetralones via deacylative allylation using Pd(0) and (*S*)-*t*Bu-PHOX (**1**) ligand (Scheme 23).^35^ This method uses a readily available cyclic ketone pro-nucleophile (**100**) and allylic alcohol (**101**) as the allyl source. The reaction involves a retro-Claisen cleavage which results in the formation of both reactive intermediates, the active nucleophile (alkoxide) and electrophile (π-allyl). It was observed that the starting acetyl tetralone (**100**) must be used in slight excess (1.05 equiv) compared to the alkoxide as it had a detrimental effect on *ee*. The possible explanation for this is that excess alkoxide competes with the enolate for coordination to Pd which interferes with the inner sphere mechanism for allylation as reported by Stoltz.^36^

**Scheme 24:**
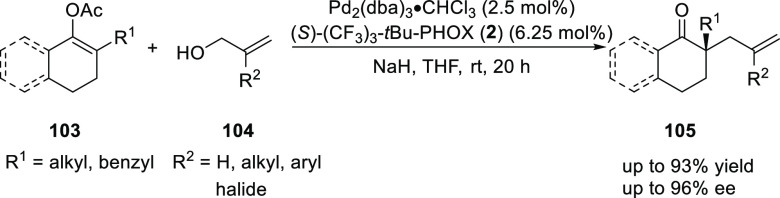
Intermolecular Tsuji Allylation of Enol Acetates

After Tunge’s initial report, Aponick extended this intermolecular Tsuji allylation to enol acetates (Scheme 24).^37^ The enol acetate substrate (**103**) is advantageous over the Tunge type 1,3-diketones as they can be easily prepared. This intramolecular allylation version facilitates rapid diversification of the α-quaternary stereocenter-containing products. The method tolerates a wide range of functional groups on the allylic part and the enol acetate of both aromatic and aliphatic ketones. The reaction proceeds with enantioselectivity up to 96% *ee* and the selectivity has a strong dependence on concentration in intermolecular acyl-transfer reactions as the lower concentrations gives products with higher enantioselectivity. The present protocol is better for 2-substituted allyl alcohols compared to simple allyl alcohol substrates.

**Scheme 25:**
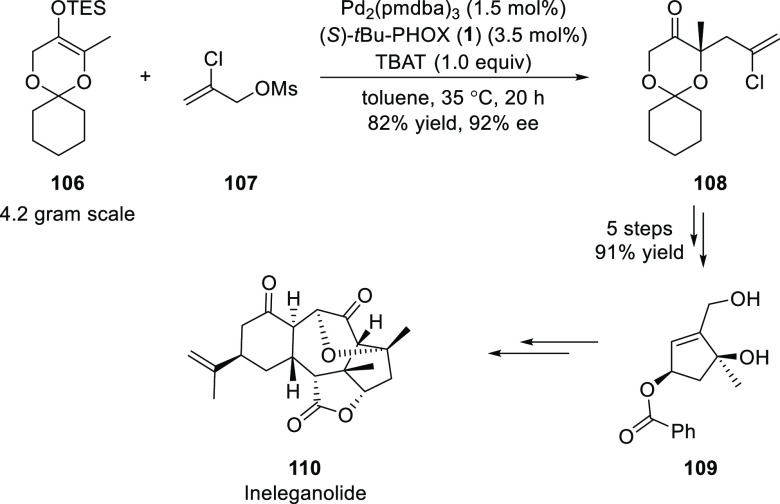
Synthesis of Polycyclic Norditerpenoid Ineleganolide

Stoltz disclosed an elegant approach toward the synthesis of the polycyclic norditerpenoid ineleganolide (Scheme 25).^38^ A key Pd/(*S*)-*t*Bu-PHOX (**1**)-catalyzed enantioselective allylic alkylation was employed to synthesize ketone **108**, bearing a chloro-substituted allyl moiety and a methyl group at the α-position, in 82% yield and 92% *ee*. Ketone **108** underwent a series of diastereoselective transformations to provide a 1,3-*cis*-cyclopentenediol **109** in 91% yield over five steps. This diol served as a building block for the synthesis of ineleganolide (**110**).

**Scheme 26:**
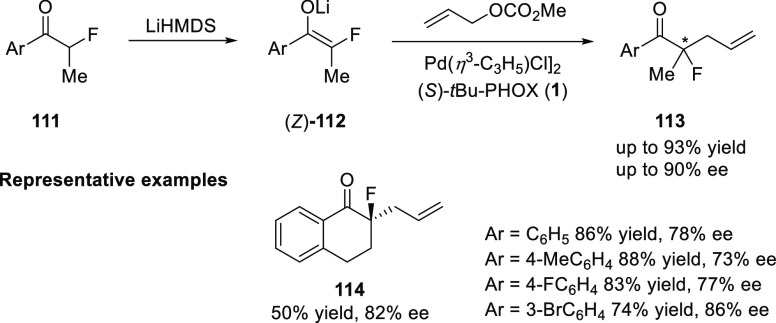
Asymmetric Synthesis of Acyclic α-Carbonyl Tertiary Alkyl Fluorides

In 2014, Guo and Chen reported a method for the asymmetric synthesis of acyclic α-carbonyl tertiary alkyl fluorides (**113**) using an enantioselective Tsuji-Trost reaction of racemic acyclic α-fluorinated ketones (**111**) (Scheme 26).^39^ The enantioselectivity for the acyclic α-fluoro ketones (**113**) products formed ranged from 60 to 90% *ee*. The good (*Z*)-configuration of *in situ* generated enolate intermediate and the presence of a fluorine atom in the starting material had a positive effect on the enantioselectivity observed. It is well documented that cyclic ketones give a better level of enantioselectivity compared to acyclic ketones due to the low selectivity of acyclic ketones toward the formation of an *E*/*Z* mixture of the Pd enolate *in situ*.

**Scheme 27:**
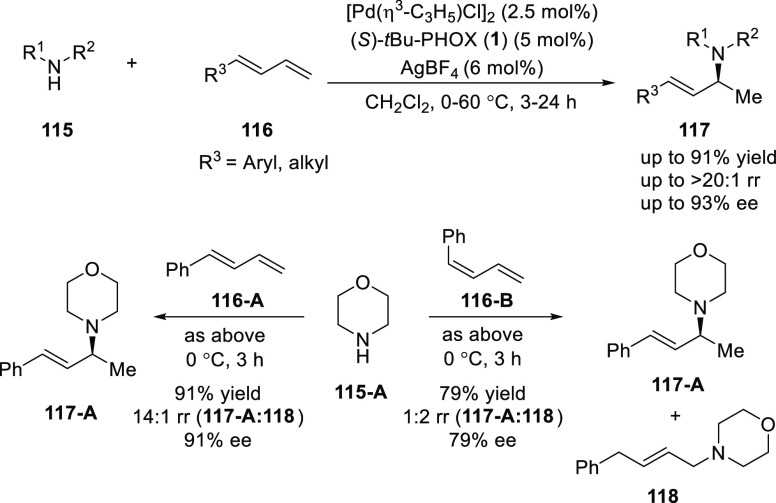
Enantioselective Hydroamination of Acyclic 1,3-Dienes

Malcolmson developed a Pd/*t*Bu-PHOX (**1**)-catalyzed enantioselective intermolecular addition of aliphatic amines (**115**) with acyclic 1,3-dienes (**116**) (Scheme 27).^40^ The chiral allylic amines (**117**) were synthesized in up to 93% *ee*. The electron-deficient phosphine ligand not only leads to a more active catalyst but also was important to achieve high levels of enantioselectivity. Mechanistic details revealed that the diene stereochemistry has a strong effect on site selectivity as (*Z*)-phenylbutadiene (**116-B**) under optimal conditions furnished a dramatically higher proportion of the 1,4-hydroamination product (**118**) instead of an expected 1,2-hydroamination product (**117-A**) (1:2 ratio) compared to the reaction of the (*E*)-isomer (**116-A**) which also proceeded with reduced enantioselectivity. It was also noted that both hydroamination products are formed exclusively as the (*E*)-isomer, indicating that olefin isomerization is faster than amine attack. Additionally, 1,2-hydroamination (**117-A**) formed the same enantiomer from either the (*E*)- or (*Z*)-isomer of the starting 1,3-butadiene. This catalytic system is highly efficient for promoting regio-and enantioselective additions of amines to terminal 1,3-dienes, although internal dienes fail to react.

**Scheme 28:**
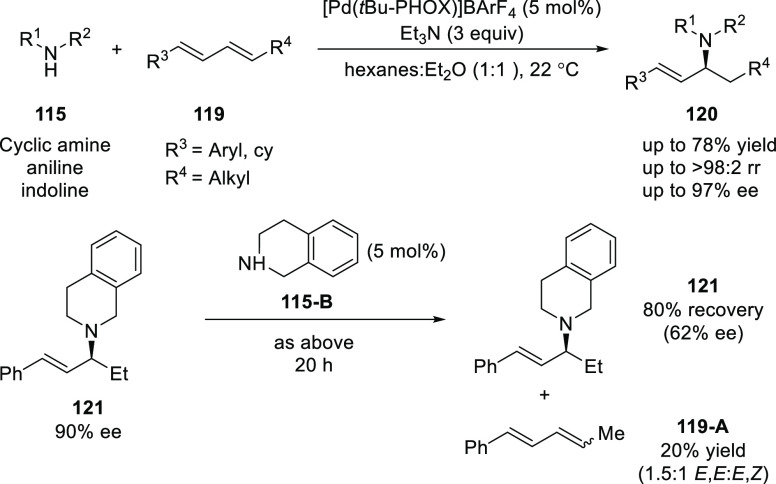
Enantioselective Hydroamination of 1,4-Disubstituted Acyclic 1,3-Dienes

After a year, Malcolmson extended their enantioselective hydroamination strategy to the challenging 1,4-disubstituted acyclic 1,3-dienes (**119**) (Scheme 28).^41^ The success of this method was largely due to a Pd-PHOX catalyst with a noncoordinating BArF_4_ counteranion, triethylamine as an additive and a nonpolar reaction medium. The variety of aryl/alkyl-disubstituted dienes (**119**) reacted with several aliphatic amines, primary anilines and indoline to generate allylic amines (**120**) possessing different α-alkyl groups in good yields and selectivities (up to 78% yield, >98:2 rr, 97% *ee*). Interestingly, secondary amines, such as piperidine and pyrrolidine, were unreactive under the optimal reaction conditions despite having greater nucleophilicity, perhaps due to the higher *p*Ka values of their conjugate acids compared to the more electron-deficient morpholine, piperazines, and tetrahydroisoquinoline (THIQ) which reacts remarkably well in this reaction. Mechanistic details indicated that the hydroamination is reversible as the *ee* of the product was found to diminish over time.

**Scheme 29:**
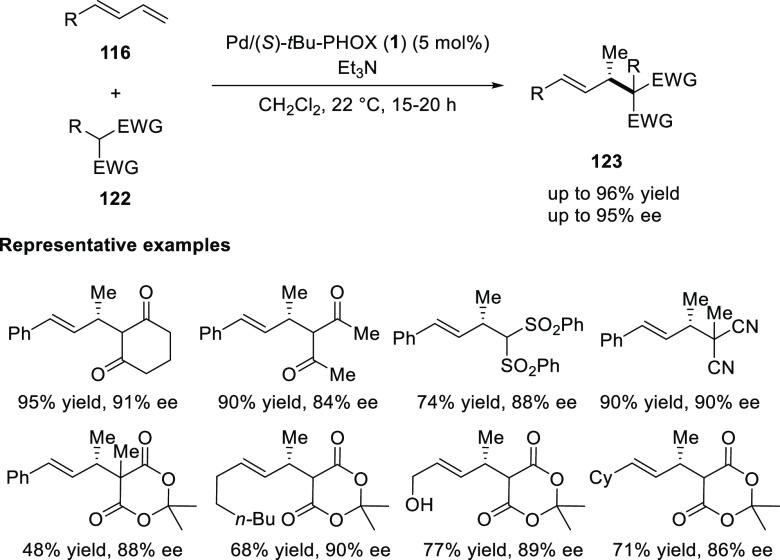
Enantioselective Hydroalkylation of Acyclic 1,3-Diene

Malcolmson employed a catalytic system of Pd-PHOX for the enantioselective intermolecular hydroalkylation of acyclic 1,3-dienes with Meldrum’s acid derivatives and other activated carbon pro-nucleophiles (Scheme 29).^42^ A variety of aryl- and alkyl-substituted dienes (**116**) react with different β-dicarbonyl-like nucleophiles (**122**) to generate allylic stereogenic centers at the carbonyl’s β-position in up to 96% yield and 95% *ee*. Similar to hydroamination (Scheme 28), here the use of triethylamine is essential as it acts as a base which upon deprotonation of carbonyl nucleophile would generate the corresponding ammonium enolate and also its protonated source, might potentially act as an acid source for Pd–H formation within the catalytic cycle. Additionally, it is important to note that the reaction is highly site-selective as addition occurs across the diene’s terminal olefin.

**Scheme 30:**
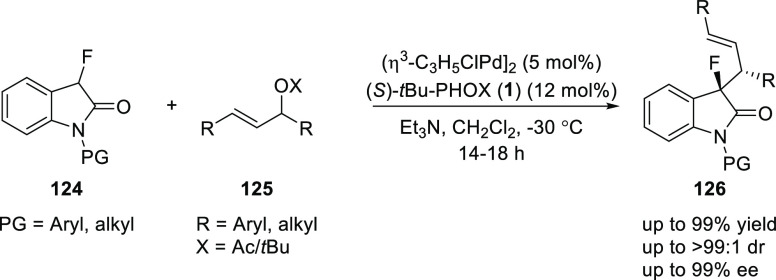
Enantioselective Allylation of 3-Fluorinated Oxindoles

Wolf presented a regio-, diastereo-, and enantioselective allylation of 3-fluorinated oxindoles (**124**) using catalytic amounts of Pd and (*S*)-*t*Bu-PHOX (**1**) (Scheme 30).^43^ This method offers a synthesis of 3,3-disubstituted fluorooxindole alkaloids (**126**) possessing vicinal chiral centers. This protocol was effective with allylic acetates which carried only one aryl terminus and C–C bond formation occurs at less hindered sites irrespective of the origin of the acetyl group.

**Scheme 31:**
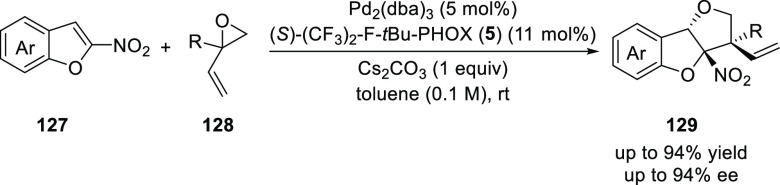
Enantioselective [3 + 2] Cycloaddition with 2-Nitrobenzofurans

You reported a stereoselective synthesis of tetrahydrofuro-benzofurans (**129**) and tetrahydrofurobenzothiophenes by the dearomatization of nitrobenzofurans (**127**) and nitrobenzo-thiophenes, respectively using a Pd/*t*Bu-PHOX (**1**)-catalyzed formal [3 + 2] cycloaddition (Scheme 31).^44^ The electronic and steric factors on the phosphine of the PHOX ligand were crucial for the success of this reaction. The products with vicinal stereogenic carbon centers were obtained in good to excellent diastereoselectivity (13/1 to >20/1 dr), and enantioselectivity (75–94% *ee*).

**Scheme 32:**
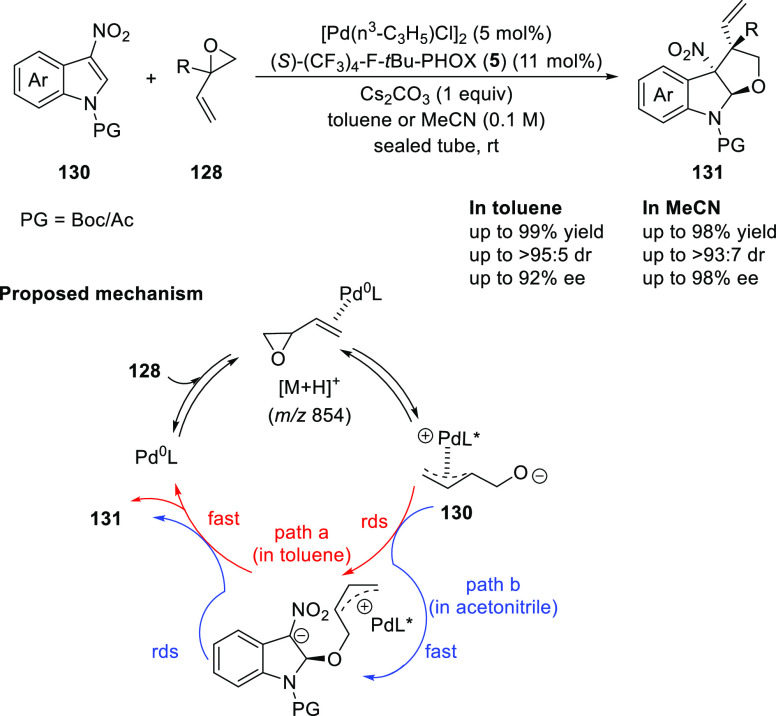
Enantioselective [3 + 2] Cycloaddition with 3-Nitroindoles

You also extended this methodology to the 3-nitroindole substrates (**130**) (Scheme 32). A stereodivergent synthesis of tetrahydrofuroindoles (**131**) is reported through a diastereoselective and enantioselective dearomative formal [3 + 2] cycloaddition by using catalytic Pd and PHOX ligand.^45^ The polarity of the solvent was key to obtain high levels of diastereoselectivity. The use of toluene furnished tetrahydrofuroindole products in good to excellent yields (70–99%), diastereoselectivity (87:13 → 95:5 dr), and enantioselectivity (70–88% *ee*) whereas acetonitrile yielded another diastereomer in good to excellent yields (75–98%) and stereoselectivity (78:22–93:7 dr and 86–98% *ee*). Mechanistic studies suggested that the origin of the diastereodivergency was due to the different rate-limiting steps in different solvents, thereby leading to a reversal of stereocontrol.

**Scheme 33:**
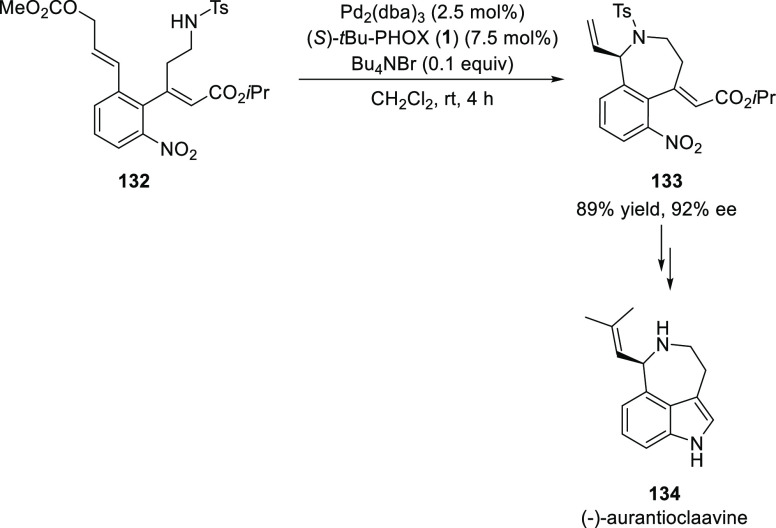
Catalytic Enantioselective Total Synthesis of (−)-Aurantioclavine

In 2014, Takemoto achieved a catalytic enantioselective total synthesis of (−)-aurantioclavine (**134**) in 16 steps starting from 3-nitro-2-iodophenol (Scheme 33).^46^ The key step involves intramolecular asymmetric amination of allylic carbonate (**132**), which has a TsNHR group, using Pd and (*S*)-*t*Bu-PHOX (**1**) which proceeded smoothly to furnish a seven-membered heterocycle (**133**) in 89% yield and 92% *ee*. This asymmetric allylic amination reaction is a powerful tool to construct a chiral azepinoindole skeleton.

**Scheme 34:**
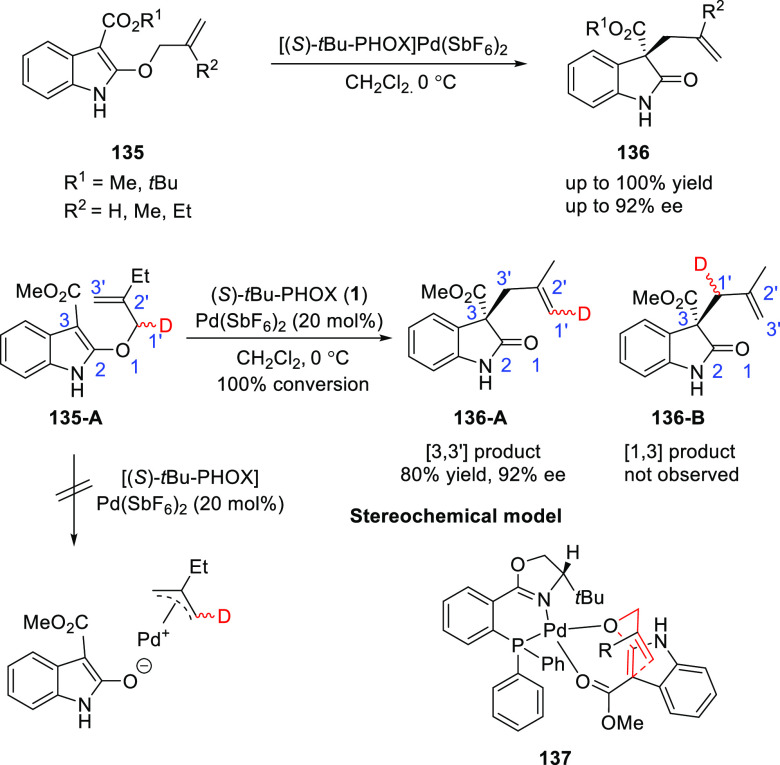
Enantioselective Meerwein–Eschenmoser Claisen Rearrangement

Kozlowski established a first catalytic, enantioselective Meerwein–Eschenmoser Claisen rearrangement using Pd/*t*-Bu-PHOX (**1**) complex (Scheme 34).^47^ This method allowed the synthesis of a range of oxindoles (**136**) bearing a quaternary stereocenter. The counterion on Pd(II) was crucial in line with catalyst coordination to the β-amidoester substrate (**135**), the SbF_6_ and BF_4_ complexes provided faster rates and greater turnover than the corresponding perchlorates, triflates, and halides. The Pd/*t*Bu-PHOX (**1**) combination provided the best enantioselectivity with the smaller C3 methyl ester (R^1^) and the larger C2′ groups (R^2^). Deuterium labeling experiments suggested a Lewis acid-catalyzed mechanism and excluded the possibility of π-allyl cation chemistry. In the proposed stereochemical model (**137**), substrate β-amidoester (**135**) shows two-point coordination from both oxygens to the chiral Pd complex.

**Scheme 35:**
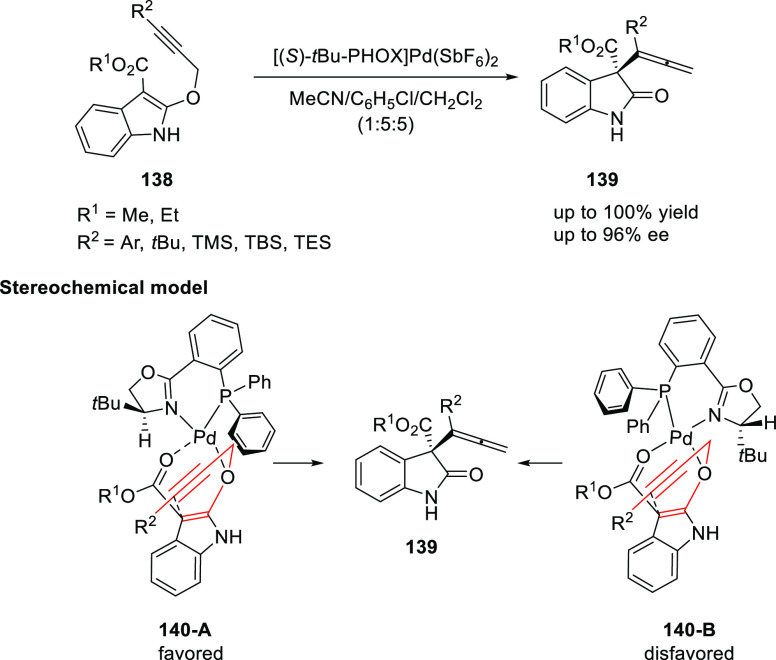
Enantioselective Saucy–Marbet Claisen Rearrangement

Kozlowski presented the first Pd/*t*Bu-PHOX (**1**)-catalyzed, enantioselective Saucy–Marbet Claisen rearrangement to generate enantiopure allenyl oxindoles (**139**) bearing a quaternary stereocenter at C3 from propargyl-substituted indole derivatives (**138**) (Scheme 35).^48^ Alkyne substrates possessing *ortho*-substituted aryl groups at R^2^ gave high levels of enantioselectivity and rapid conversion (up to 96% *ee*). However, alkynes with larger groups like trimethylsilyl (TMS), *tert*-butyl, or *N*-methyliminodiacetic acid (MIDA) boronate were less successful. The alkyne geometry significantly alters the topology of the rearrangement transition state. Additionally, the ester functionality interacts with larger alkyne terminal substituents and destabilizes the less-favorable reaction pathway. Furthermore, improved results were achieved with nonaryl alkynes using Binap and its congeners.

##### 2.1.1.2 Application of PHOX in Decarboxylative Asymmetric Protonation (DAP)

PHOX ligands have also been extensively employed in the Pd-catalyzed decarboxylative asymmetric protonation (DAP) to generate products with a tertiary stereocenter adjacent to a carbonyl group.^49^

**Scheme 36:**
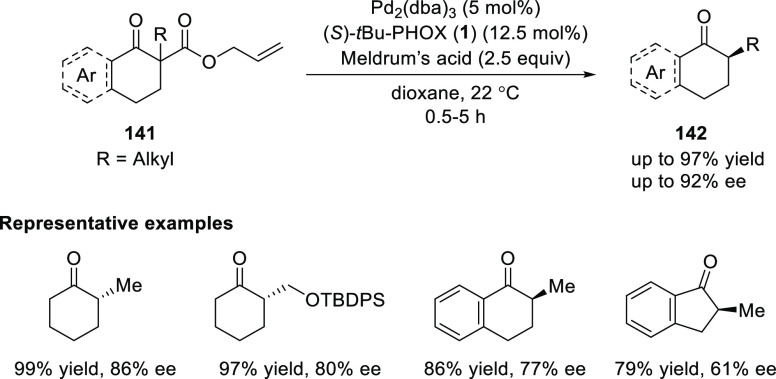
Enantioselective Decarboxylative Protonation of α-Aryl-β-ketoallyl Esters

Stoltz reported a Pd/*t*Bu-PHOX (**1**)-catalyzed DAP of racemic α-aryl-β-ketoallyl esters (**141**) (Scheme 36).^50^ The reaction generated α-alkylated cyclic ketones (**142**) in excellent yields and enantioselectivities (up to 97% yield and 92% *ee*) with Meldrum’s acid as the proton source.

**Scheme 37:**
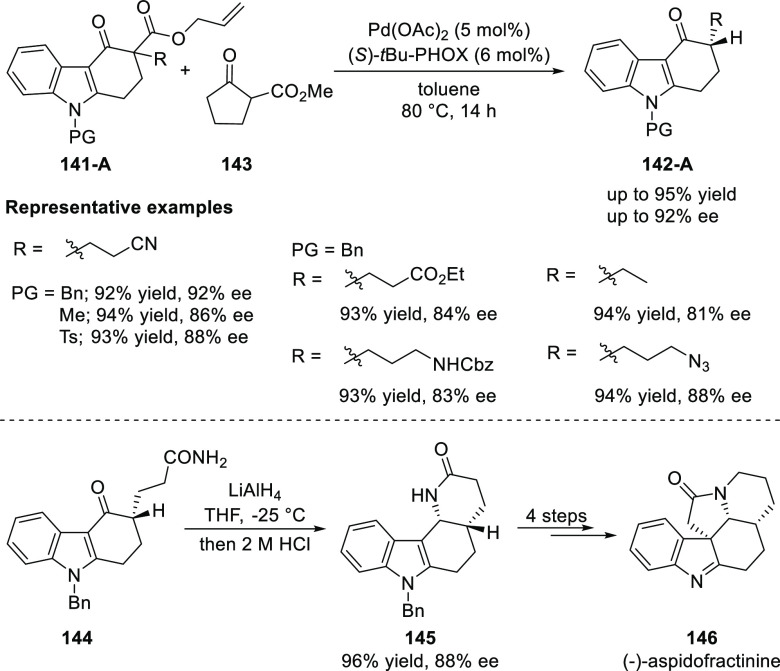
Enantioselective Synthesis of C3-Substituted Chiral Carbazolones

In 2014, Shao reported an enantioselective synthesis of C3-substituted chiral carbazolones (**142-A**) by using Pd/PHOX-catalyzed decarboxylative protonation of α-alkyl-β-keto allyl esters (**141-A**) (Scheme 37).^51^ The use of methyl 2-cyclopentanonecarboxylate (**143**) as the proton donor was found to be more effective compared to the normally used Meldrum’s acid. A range of C3-monosubstituted chiral carbazolones (**142-A**) carrying various valuable functionalities such as, nitrile, ester, amine and azide were accessed in excellent yields (up to 95%) with good enantioselectivities (up to 92% *ee*). The application of this methodology was demonstrated in the synthesis of a key pentacyclic intermediate of the naturally occurring (−)-aspidofractinine (**146**).

**Scheme 38:**
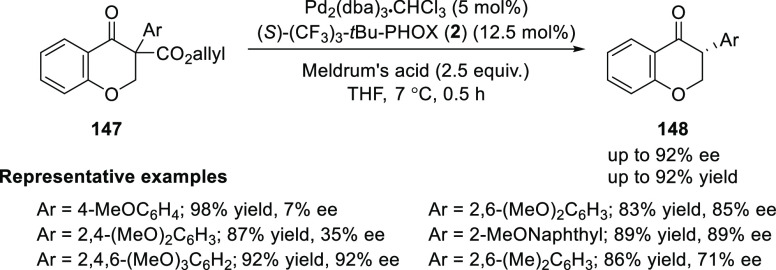
Asymmetric Synthesis of Isoflavanones

Guiry reported the first catalytic asymmetric synthesis of isoflavanones (**148**) containing an α-aryl substituent by using enantioselective decarboxylative protonation of α-aryl-β-ketoallyl ester (**147**) using Meldrum’s acid as the proton source (Scheme 38).^52^ It was observed that this method is influenced by both the sterics and the electronics on the α-aryl ring of the isoflavanone. The substrate containing coordinating methoxy substituents at both *ortho*-positions seems to be essential to achieve optimal enantioselectivity. This method allowed them to synthesize highly sterically hindered α-aryl ketones with enantioselectivities of up to 92%.

**Scheme 39:**
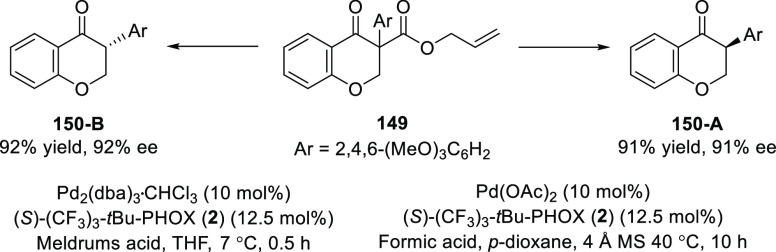
Synthesis of Tertiary α-Aryl Isoflavanones

In their following report, Guiry developed a Pd-catalyzed decarboxylative asymmetric protonation of α-aryl-β-ketoallyl esters (**149**) to generate a tertiary α-aryl isoflavanones (**150-A**) using formic acid as a proton source (Scheme 39).^53^ It is interesting to note that, a switch in the preferred enantiomer formed was observed with formic acid as the proton source compared to their earlier work using Meldrum’s acid as the proton source. It is one of the rare examples of an asymmetric protonation wherein dual stereocontrol was observed with the same chiral ligand but with a different achiral proton donor. The Pd-enolate formed after decarboxylation of α-aryl-β-ketoallyl esters (**149**) was protonated by Meldrum’s acid. Remarkably, the rates of the reactions are significantly different as the Meldrum acid-mediated reaction completes in 30 min, whereas the formic acid reaction takes up to 10 h which suggests the necessity for a carbopalladation to occur and subsequent quenching by formic acid. Alternatively, some precoordination of formic acid to the chiral Pd–enolate complex may result in an inner-sphere-type protonation to a different face of the enolate than with Meldrum’s acid.

**Scheme 40:**
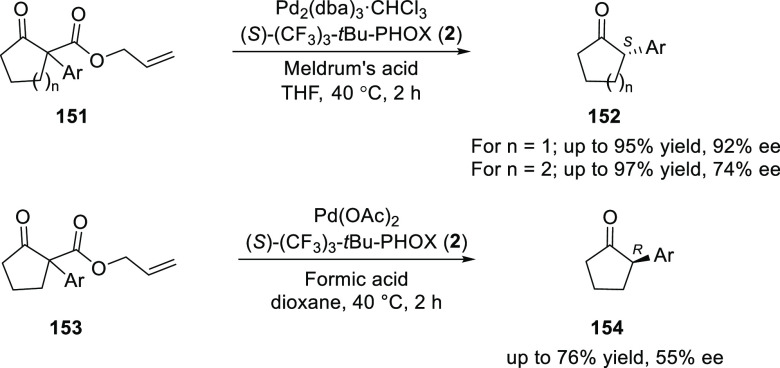
Asymmetric Synthesis of Tertiary α-Aryl Cyclopentanones and Cyclohexanones

In the same year, Guiry extended the methodology for the synthesis of a series of tertiary α-aryl cyclopentanones (**152**) and cyclohexanones (**154**) from α-aryl-β-keto allyl esters (**151** and **153**) in moderate to good levels of enantioselectivity (Scheme 40).^54^ Similar to earlier methods, good levels of enantioselectivity were achieved for substrates containing aryl groups with mono- and di-*ortho*-substitution.

**Scheme 41:**
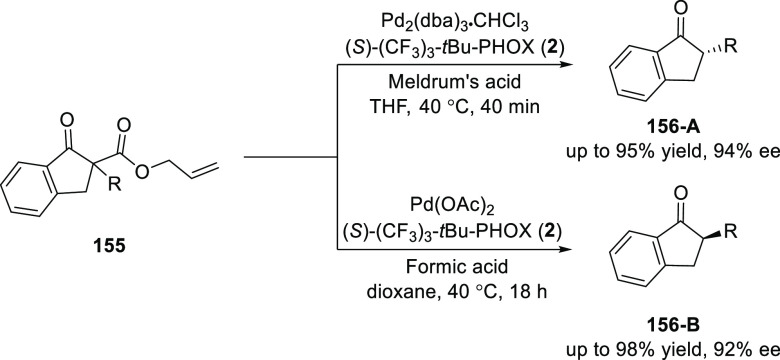
Synthesis of Tertiary α-Aryl-1-indanones

Guiry further studied a scope of the decarboxylative asymmetric protonation with indanone based α-aryl-β-keto allyl ester substrates (**155**) (Scheme 41).^55^ As seen with other cyclic ketones, both enantiomers of a series of tertiary α-aryl-1-indanones (**156**) were accessible by simply switching the achiral proton source. In this example of dual stereocontrol, enantioselectivities up to 92% *ee* (*R*) and 94% *ee* (*S*) were achieved using formic acid and Meldrum’s acid, respectively.

##### 2.1.1.3 Application of PHOX in Asymmetric Hydrogenation

**Scheme 42:**
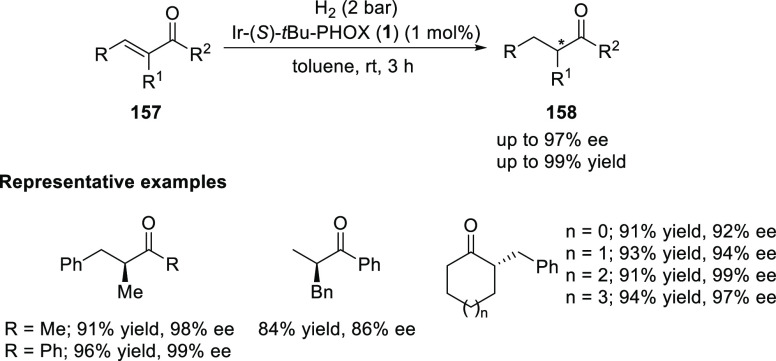
Asymmetric Hydrogenation of α-Substituted Enones

**Scheme 43:**
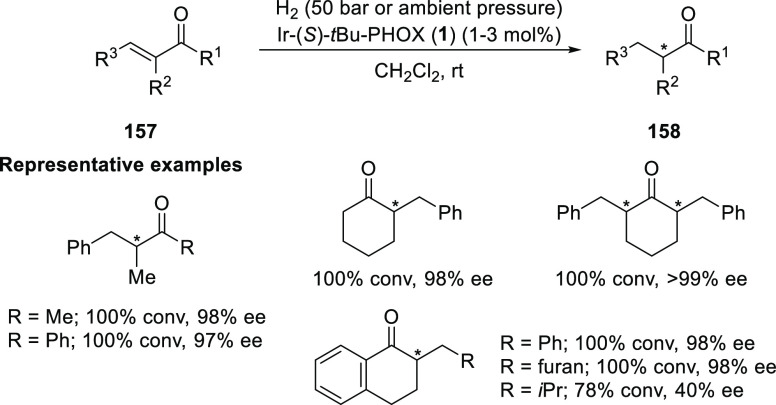
Asymmetric Hydrogenation of α-Substituted Enones

Bolm and Hou independently reported the asymmetric hydrogenation of α-substituted enones (Schemes 42 and 43).

Bolm discovered a simple and highly efficient asymmetric synthesis of α-substituted ketones (**158**) from α-substituted enones (**157**) (Scheme 42).^56^ Both acyclic and cyclic enones underwent smooth catalytic enantioselective hydrogenations using the Ir complex of *t*Bu-PHOX (**1**) furnishing α-substituted ketones in high yields and enantioselectivities while tolerating a wide variety of substituents.

Hou reported an Ir-catalyzed asymmetric hydrogenation of α-substituted-α,β-unsaturated ketones (**157**) to generate a chiral center at the α-position of ketones (**158**) (Scheme 43).^57^ Different ketone substrates possessing exocyclic alkenes furnished products in high enantioselectivity, even under ambient pressure of hydrogen.

**Scheme 44:**
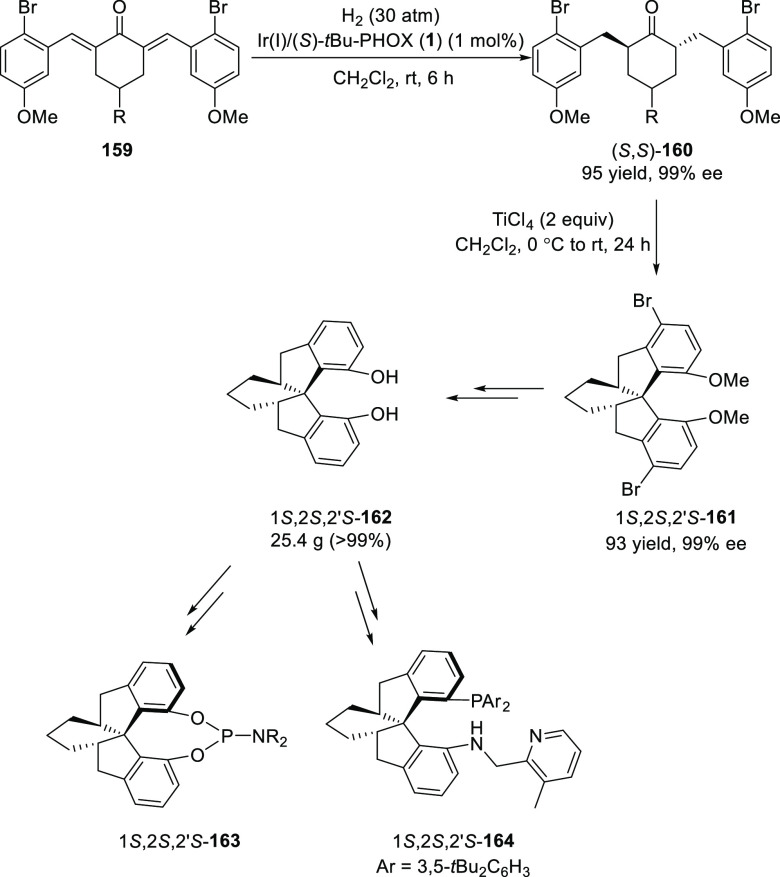
Asymmetric Hydrogenation of α,α′-Bis(arylidene)ketones

Ding reported an enantioselective synthesis of cyclohexyl-fused chiral spirobiindane derivative (**161**) via a sequence of an Ir-catalyzed asymmetric hydrogenation of α,α′-bis(arylidene)ketones (**159**), followed by an asymmetric spiroannulation of hydrogenated chiral ketones (**160**) promoted by TiCl_4_ (Scheme 44).^58^ A variety of cyclohexyl-fused chiral spirobiindanes (**161**) were synthesized in high yields and excellent stereoselectivities (up to >99% *ee*). The asymmetric synthesis of cyclohexyl-fused spiroindanediol (1*S*,2*S*,2′*S*)-**162**, was carried out in >99% *ee* and 67% overall yield over four steps without chromatographic purification. The (1*S*,2*S*,2′*S*)-**162** was then used to access chiral monodentate phosphoramidites **164** and tridentate phosphorus-aminopyridine **164** and the applications of these ligands were shown in various metal-catalyzed enantioselective reactions including hydrogenation, hydroacylation, and [2 + 2] reactions. The catalytic performances of these ligands were very similar to the corresponding well-established spirobiindane backbone based privileged ligands.

**Scheme 45:**
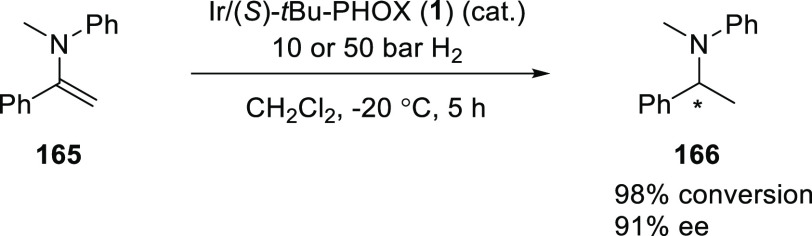
Asymmetric Hydrogenation of Enamines

Pfaltz discovered an asymmetric hydrogenation of enamines (**165**) catalyzed by cationic Ir complexes derived from *t*Bu-PHOX (**1**) (Scheme 45).^59^ The level of enantioselectivites observed in these hydrogenations greatly depend on the substitution pattern at the double bond and the nitrogen atom. An *N*-aryl or *N*-benzyl group seems to have a beneficial effect on the enantioselectivity (>90% *ee*), whereas enantioselectivities for the hydrogenation of cyclic and acyclic 1,2-disubstituted enamines were lower.

**Scheme 46:**
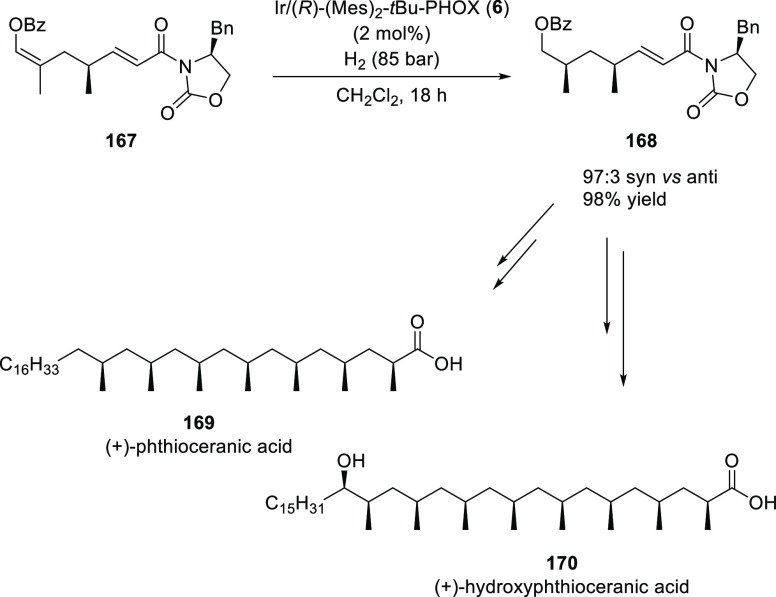
Stereoselective Hydrogenation of **167**

Pfaltz applied an Ir-catalyzed hydrogenation for the stereoselective and flexible synthesis of long-chain polydeoxypropionates (Scheme 46).^60,61^ The Ir complex of chiral PHOX-ligand **6** catalyzed the hydrogenation of **167** with excellent levels of diastereoselectivity. The benzoate anti-**167** were obtained with 96:4 diastereoselectivity and >95% isolated yield. Interestingly the opposite diastereofacial selectivity was obtained when the mesityl group on the phosphine ligand was changed to an *o*-tolyl or a cyclohexyl. Hydrogenated benzoate **168** was further utilized to complete the synthesis of the glycolipid components (+)-phthioceranic acid (**169**) and (+)-hydroxyphthioceranic acid (**170**).

**Scheme 47:**
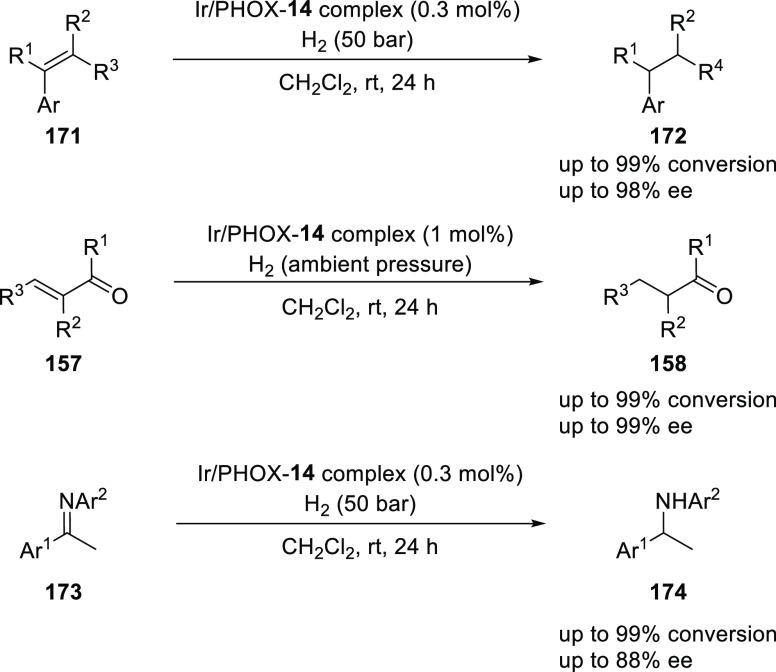
Asymmetric Hydrogenation of Unfunctionalized Alkenes

Hou reported an Ir-catalyzed asymmetric hydrogenation of unfunctionalized alkenes (**171**), α,β-unsaturated esters (**157**), allyl alcohols, α,β-unsaturated ketones (**157**), and ketimines (**173**) using benzylic substituted PHOX ligand **14** (Scheme 47).^62^ A wide range of substrates were successfully reduced to the corresponding chiral products (**172**, **158**, and **174**) with high conversions and high *ee* values (>99% conversion and up to 99% *ee*). The reaction of (*Z*)- and (*E*)-alkenes gave rise to enantiomeric products in almost the similar enantiomeric excess catalyzed by the same Ir-PHOX **14** complex. The reported *P*,*N*-ligands have a six-membered ring with Ir, whereas ligand **14** which is derived from a benzylic substituted *P*,*N*-ligand has a seven-membered ring. The increased tether length is more flexible and is believed to have a better chance to fit the steric demands of various substrates. The slightly larger bite angle of ligand was confirmed by X-ray analysis of the corresponding Ir complex.

**Scheme 48:**
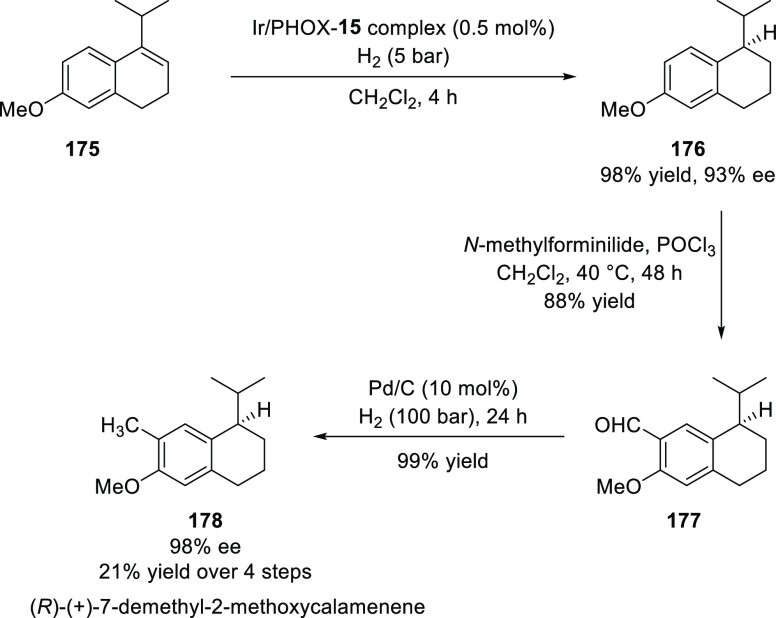
Asymmetric Hydrogenation of Unfunctionalized Alkenes

Pfaltz reported a further Ir-catalyzed asymmetric hydrogenation of unfunctionalized and functionalized alkenes (**175**) using a new *P*,*N*-ligand **15** introduced from their group (Scheme 48).^63^ The application of their methodology was demonstrated in the synthesis of the antitumor natural product, (*R*)-(+)-7-demethyl-2-methoxycalamenene (**178**), a superior approach to an earlier published route with respect to the number of steps and the overall yield (4 steps, 21% overall yield and 98% *ee*). The catalytic system was also extended to the asymmetric hydrogenation of allylic alcohols, α,β-unsaturated esters and imines which resulted in the corresponding products in good to excellent *ee*s (up to 96%).

**Scheme 49:**
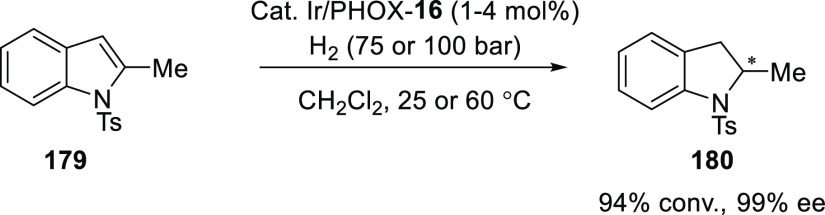
Asymmetric Hydrogenation of 2-Substituted *N*-Protected Indoles

Pfaltz described a cationic Ir/chiral *P*,*N*-ligand **16** system for the efficient asymmetric hydrogenation of 2-substituted *N*-protected indoles (**179**) (Scheme 49).^64^ The Ir catalyst employed is highly air and moisture sensitive. Various indoles with *N*-Boc-, *N*-acetyl-, and *N*-tosyl protecting groups were hydrogenated in excellent yields and enantioselectivities. The influence of *N*-protection on the reactivity and enantiomeric excess was lessened by employing the right combination of the catalytic system.

**Scheme 50:**
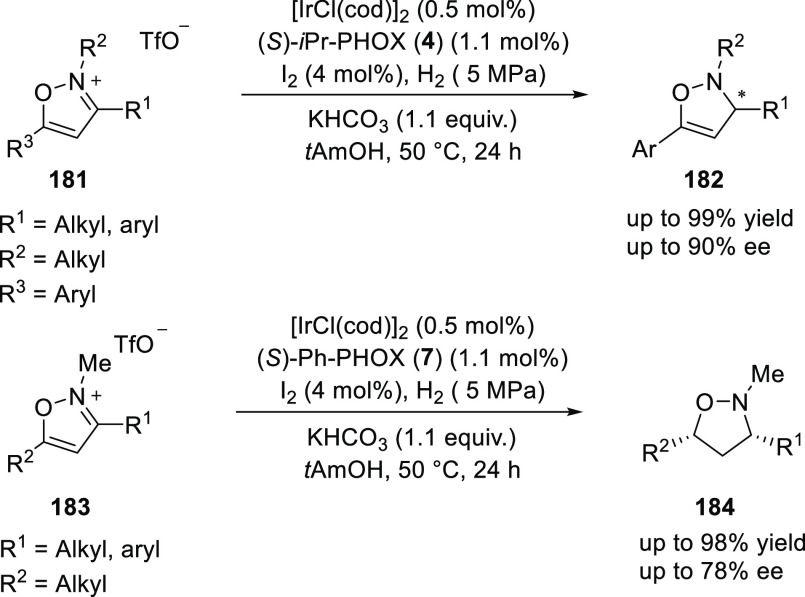
Enantioselective Hydrogenation of Isoxazolium Triflates

Kuwano developed an Ir-catalyzed enantioselective hydrogenation of isoxazolium triflates (**181** and **183**) to furnish enantioenriched 4-isoxazolines (**182**) or isoxazolidines (**184**) (Scheme 50).^65^ The hydrogenation of 5-arylisoxazolium triflates (**181**) exclusively provided the corresponding 4-isoxazolines (**182**) with good enantioselectivities (up to 90% *ee*) whereas 5-alkylated isoxazolium triflates (**183**) selectively furnished *cis*-3,5-disubstituted isoxazolidines (**184**) with *ee*s up to 78%. The enantioselectivity was strongly affected by the hindrance from the 5-substituent on the isoxazole ring. Interestingly, the commonly occurred and undesired N–O bond cleavage was not observed under the reaction conditions employed.

##### 2.1.1.4 Application of PHOX in Addition of Arylboronic Acids to Alkyne, Allene, or Imine

**Scheme 51:**
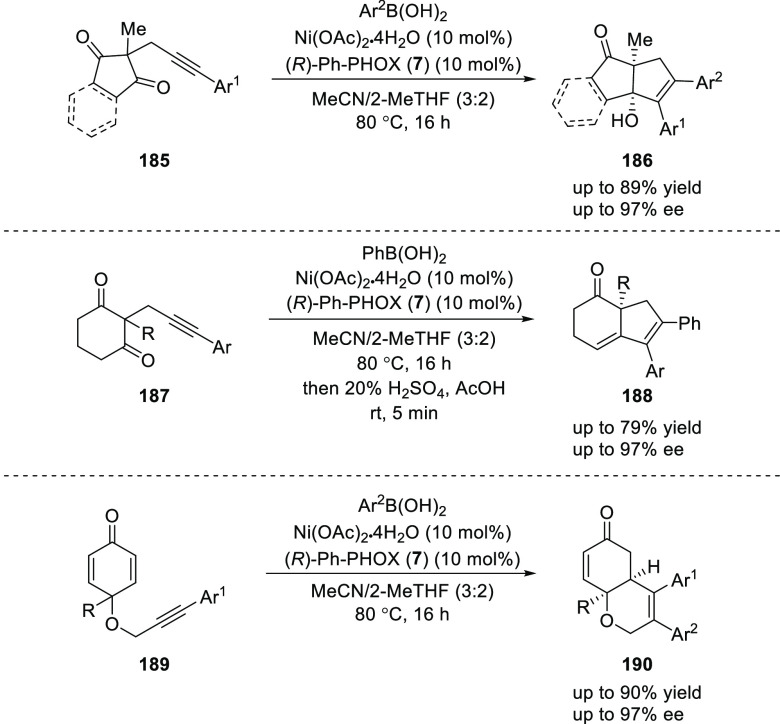
Enantioselective Domino Cyclization of Tethered Alkynes

Lam reported a Ni/(*R*)-Ph-PHOX (**7**)-catalyzed reaction of arylboronic acids with alkynes (**185**, **187**, and **189**) followed by the enantioselective cyclization of an alkenyl Ni species onto a tethered electrophilic trap such as ketones or enones (Scheme 51).^66^ The success of this domino cyclization is reliant upon the formal *anti*-carbonickelation of the alkyne, which is suggested to occur by the reversible *E*/*Z*-isomerization of an alkenylnickel species.

**Scheme 52:**
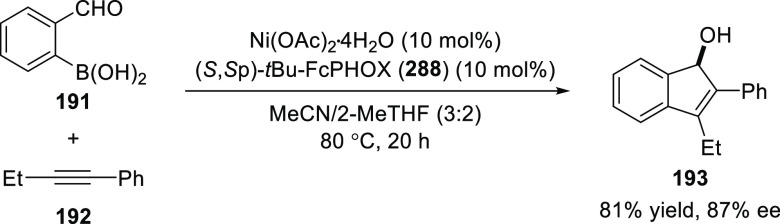
Intramolecular Reaction between 1-Phenyl-1-butyne and 2-Formylphenylboronic Acid

Furthermore, an intramolecular reaction between 1-phenyl-1-butyne (**192**) and 2-formylphenylboronic acid (**191**) using (*S*,*S*_p_)-*t*Bu-FcPHOX (**288**) (discussed in detail in section 2.3) furnished the expected indene **193** in 81% yield and 87% *ee* via a *syn*-arylnickelation of the alkyne followed by cyclization of the resulting alkenylnickel species onto the aldehyde (Scheme 52). Interestingly, the same chiral ligand, (*S*,*S*_p_)-*t*Bu-FcPHOX (**288**), earlier furnished a product *via anti*-arylnickelation. The ability to synthesize enantioenriched products from either a *syn*- or *anti*-carbometallative cyclization further suggests that reversible *E*/*Z*-isomerization of the alkenylnickel species is operative and also demonstrates the adaptive power of this Ni-based catalytic system.

**Scheme 53:**
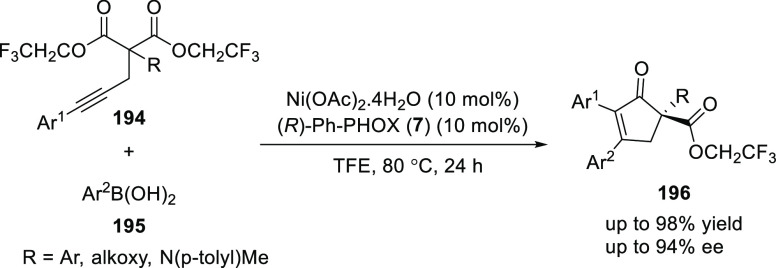
Desymmetrizing Arylative Cyclization of Alkynyl Bis(2,2,2-trifluoroethyl) Malonates

Lam reported a Ni/(*R*)-Ph-PHOX (**7**)-catalyzed desymmetrizing arylative cyclization for the enantioselective synthesis of chiral cyclopent-2-enones (**196**) by using alkynyl bis(2,2,2-trifluoroethyl) malonates (**194**) and arylboronic acids (**195**) (Scheme 53).^67^ A wide variety of cyclopent-2-enones (**196**) containing a quaternary stereocenter were synthesized in good yields and excellent enantioselectivities (up to 98% and 94% *ee*). This cyclization was promoted by the reversible *E*/*Z*-isomerization of the alkenylnickel species formed during the reaction.

**Scheme 54:**
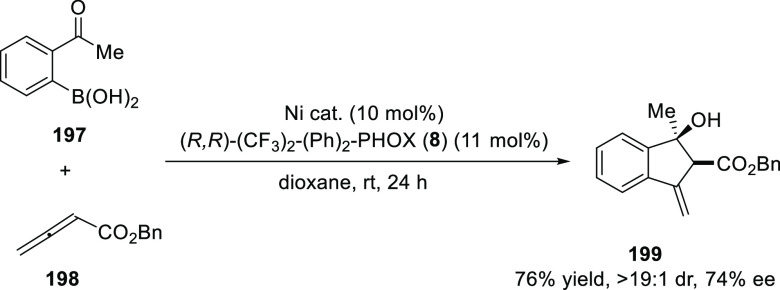
Diastereoselective Annulations between 2-Acetylphenylboronic Acid and Allene

Lam developed a Ni-catalyzed highly diastereoselective annulation between 2-acetylphenylboronic acid (**197**) or 2-formylarylboronic acid and activated allenes (**198**) to furnish 3-methyleneindanols (**199**) (Scheme 54).^68^ Preliminary results using the chiral phosphinooxazoline ligand, (*R*,*R*)-(CF_3_)_2_-(Ph)_2_-PHOX (**8**) furnished the product in 76% yield and good stereoselectivities (19:1 dr and 74% *ee*).

**Scheme 55:**
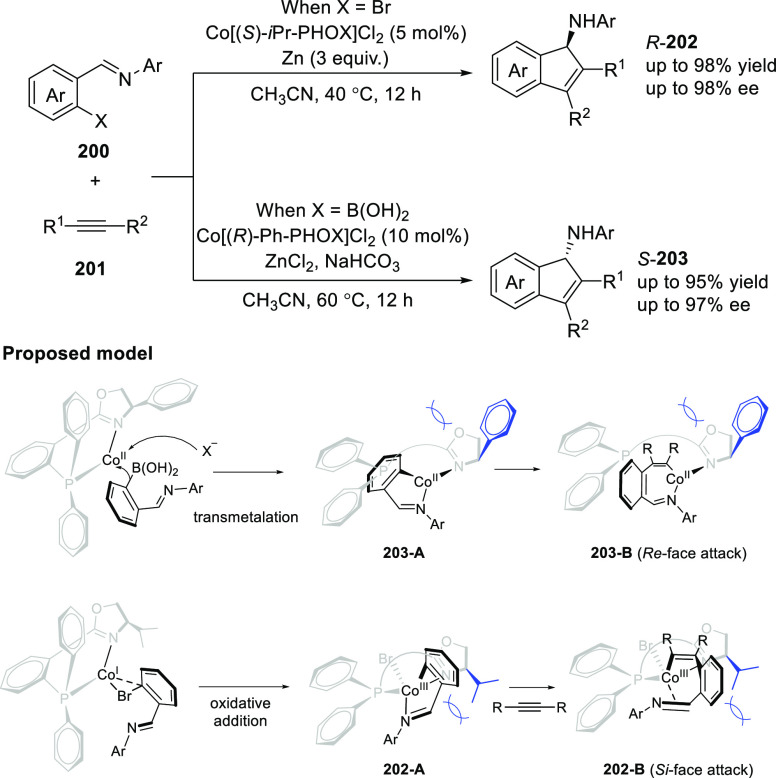
Enantioselective [3 + 2] Annulation Reaction

Cheng reported the synthesis of chiral 1-aminoindenes (**202/203**) through an intramolecular reaction between *o*-imodoylarylboronic acids or aryl halides or pseudohalides (**200**) with alkynes (**201**) using a Co/chiral PHOX-catalyzed enantioselective [3 + 2] annulation reaction (Scheme 55).^69^ This reaction achieved a controlled synthesis of both enantiomers of variety of 1-aminoindenes (**202/203**) in high yields and good to excellent enantioselectivities (up to 98% yield and 98% *ee*). The unsymmetrical alkynes reacted regioselectively affording single stereoisomers in high yield and excellent *ee* values. The role of ZnCl_2_ was found to be crucial as it dramatically enhanced the yield without any negative effect on enantioselectivity. The configuration of the desired product is dominated and controlled by the steric bulk of the substituent attached to the nitrogen-adjacent carbon on the oxazoline ring. As demonstrated in the proposed model, the phenyl group of complex **203-A** forces the R group to be far away from the oxazoline moiety and leads the subsequent intramolecular addition of imine from the *Re*-face, while the isopropyl group of complex **202-A** leads to *Si*-face insertion.

**Scheme 56:**
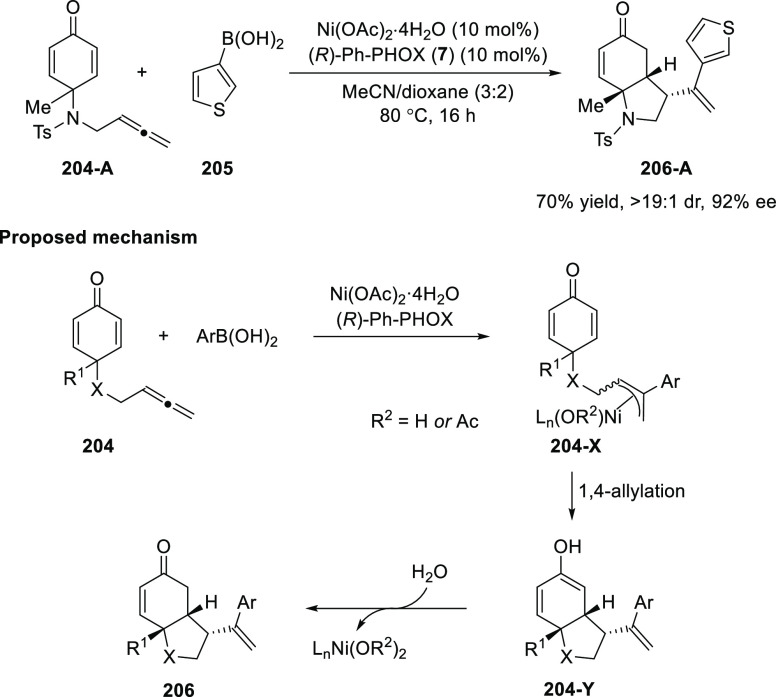
Enantioselective Ni-Catalyzed Desymmetrization of Allenyl Cyclohexa-2,5-dienones

Lam and co-workers earlier discovered that the Ni/(*R*)-Ph-PHOX (**7**) complex is highly effective in promoting enantioselective anticarbometallative cyclizations of alkynyl electrophiles with arylboronic acids.^66−68^ They hypothesized that Ni/(*R*)-Ph-PHOX (**7**) complex could also promote a catalytic enantioselective intramolecular 1,4-allylation of substrates containing an allene tethered to an electron-deficient alkene. Considering these facts Lam 2018 described the enantioselective Ni-catalyzed arylative desymmetrization of allenyl cyclohexa-2,5-dienones (**204-A**) by using arylboronic acids (**205**) (Scheme 56).^70^ A variety of hexahydroindol-5-ones and hexahydrobenzofuran-5-ones with three contiguous stereocenters were synthesized in moderate to good yield with high diastereo- and enantioselectivities. The proposed mechanism begins with the Ni/(*R*)-Ph-PHOX (**7**)-catalyzed addition of an arylboronic acid to an allenyl cyclohexa-2,5-dienone (**204**). The resulting allylnickel species **204-X** would undergo intramolecular 1,4-allylation to give nickel enolate **204-Y**, which upon protonation would liberate the Ni(II) catalyst and a *cis*-fused hexahydroindol-5-one or hexahydrobenzofuran-5-one (**206**).

**Scheme 57:**
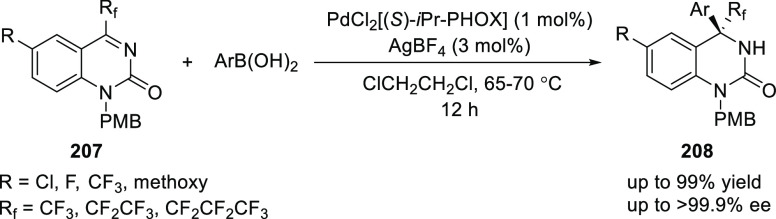
Pd-Catalyzed Asymmetric Arylation

In 2016, Hayashi reported a Pd-catalyzed asymmetric arylation of fluoroalkyl-substituted 2-quinazolinone derivatives (**207**) with arylboronic acids using (*S*)-*i*Pr-PHOX (**4**) ligand (Scheme 57).^71^ A variety of trifluoromethylated and perfluoromethylated 2-quinazoline (**208**) products possessing quaternary carbon stereocenters were synthesized in excellent enantioselectivities (>99% *ee*).

**Scheme 58:**
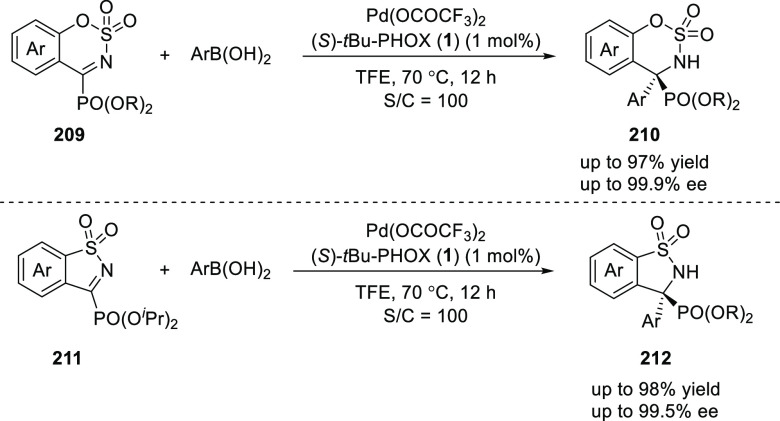
Enantioselective Addition of Arylboronic Acids to Cyclic α-Ketiminophosphonates

Later, Zhou employed a Pd-catalyzed enantioselective addition of arylboronic acids to five- and six-membered cyclic α-ketiminophosphonates (**209/211**) using (*S*)-*t*Bu-PHOX (**1**) ligand (Scheme 58).^72^ The method provided an elegant and efficient route to access chiral α-aminophosphonates (**210/212**) possessing a quaternary carbon stereocenter in high yields and excellent enantioselectivities (up to 99.9% *ee*). The use of low catalytic loading and a wide substrate scope are the highlights of this arylation methodology.

**Scheme 59:**
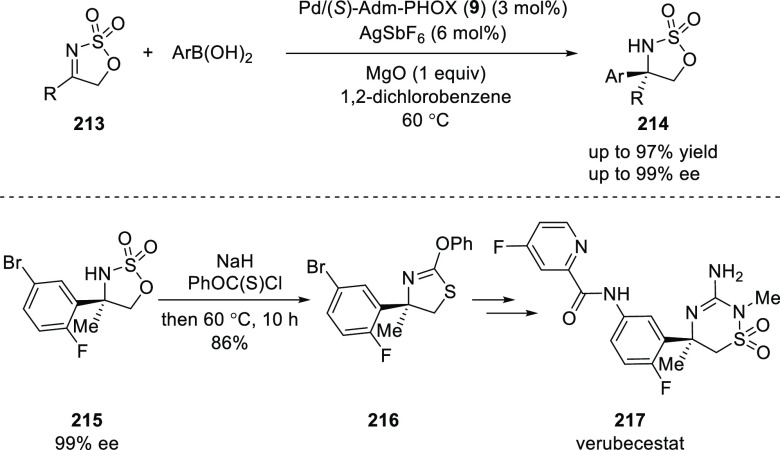
Enantioselective Addition of Arylboronic Acids to Cyclic Iminosulfates

Chen reported a Pd-catalyzed enantioselective addition of arylboronic acids to cyclic iminosulfates (**213**) using an adamantyl-substituted phosphinooxazoline ligand **9** (Scheme 59).^73^ This enantioselective arylation tolerates a wide variety of arylboronic acids as well as cyclic iminosulfates to provide cyclic sulfamidates (**214**) in high yields with excellent *ee*s (up to 97% yield and up to 99% *ee*). The methodology was applied to synthesize verubecestat (**217**), a compound under clinical evaluation for the treatment of Alzheimer’s disease.

##### 2.1.1.5 Application of PHOX in Asymmetric Borylation of Alkenes

**Scheme 60:**
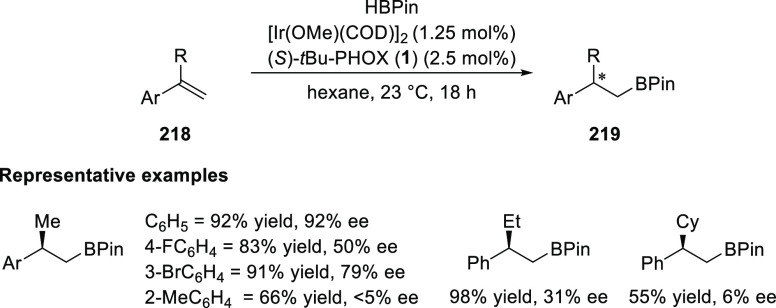
Enantioselective Hydroboration of Terminal Olefins

In 2011, Mazet developed an Ir-catalyzed regio- and enantioselective hydroboration of terminal olefins (**218**) using the *t*Bu-PHOX (**1**) ligand (Scheme 60).^74^ The reaction is highly dependent on sterics as changing the R group from methyl to ethyl or cyclohexyl or by replacing the phenyl ring with an *o*-tolyl group radically decreased the enantioselectivity of the expected hydroboration products.

##### 2.1.1.6 Application of PHOX in Asymmetric α-Arylation

**Scheme 61:**
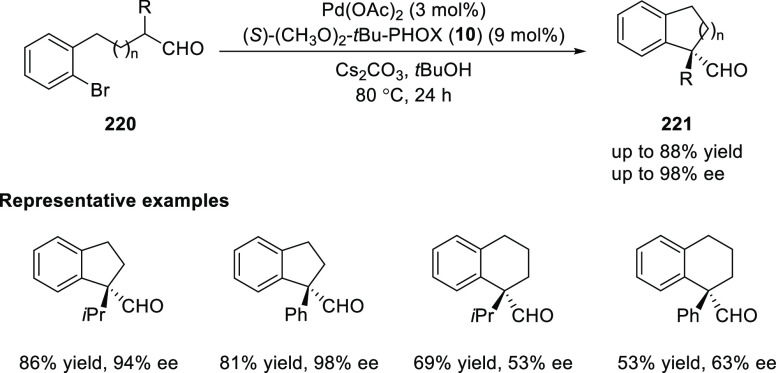
Enantioselective α-Arylation of Aldehydes

Buchwald reported a Pd-catalyzed α-arylation of aldehydes (**220**) forming all-carbon-substituted asymmetric centers in high yields and enantioselectivities (Scheme 61).^75^ Generally, substrates with α-aryl substituents gave rise to products with higher optical purity than these with α-alkyl analogues. The efficiency of the method is excellent for substrates forming five-membered rings, but it dropped significantly for substrates forming a six-membered ring; tetrahydronaphthalene derivatives were prepared in moderate to good yields with moderate levels of enantioselectivity.

##### 2.1.1.7 Application of PHOX in Asymmetric [2 + 2] Cycloaddition

**Scheme 62:**
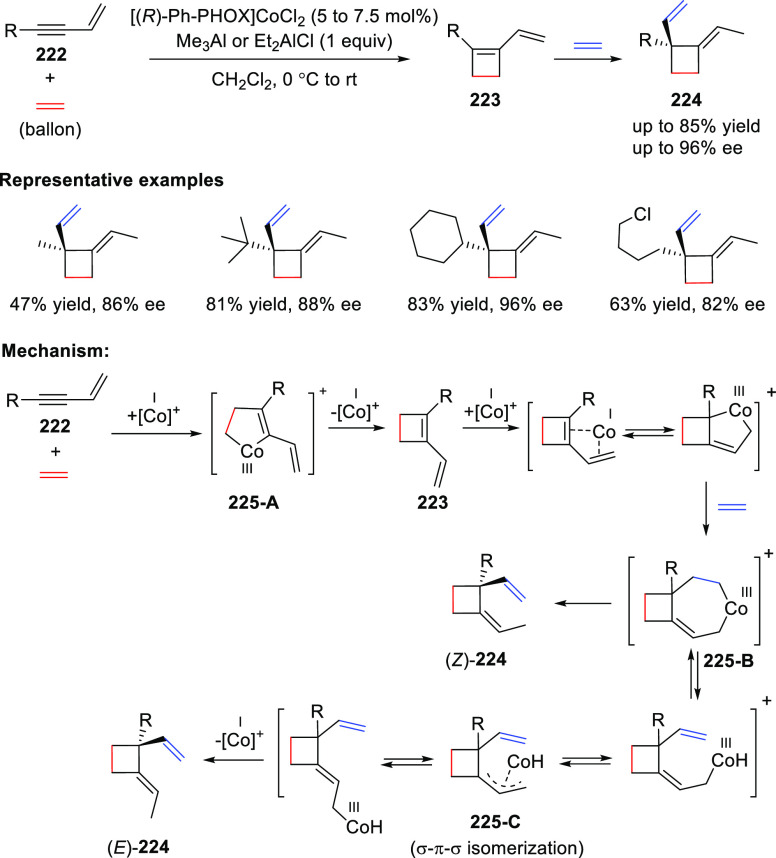
Asymmetric [2 + 2] Cycloaddition between 1,3-Enynes and Ethylene

In 2018, Rajanbabu reported a Co/(*R*)-Ph-PHOX (**7**)-catalyzed [2 + 2] cycloaddition between 1,3-enynes (**222**) and ethylene followed by an enantioselective hydrovinylation of the resulting vinylcyclobutene (**223**) to give highly functionalized cyclobutanes (**224**) with an all-carbon quaternary stereocenter, as the (*E*)-isomer (Scheme 62).^76^ The reaction proceeds in a tandem fashion to form three highly selective C–C bonds in one pot using a single chiral Co catalyst. The use of an activator, Et_2_AlCl, was important to promote this cycloaddition reaction. The cycloaddition initiates with an oxidative dimerization of ethylene and the enyne (**222**) in the coordination sphere of an activated Co(I) species to afford a metallacyclopentene **225-A**, which after reductive elimination provides cyclobutene **223**. This diene **223** further undergoes oxidative dimerization with ethylene to deliver the metallacycloheptene **225-B**. Sterically congested **225-B**, (*Z*)-allylcobalt(III)-hydride undergoes (*Z*)/(*E*)-isomerization through an η^3^-(allyl) intermediate **225-C**, which upon β-hydrogen elimination and reductive elimination finally leads to the (*E*)-**224** isomer as the major product. The ratio of (*Z*)- and (*E*)-isomers depended on the nature of the R substituent and the nature of the ligand used.

##### 2.1.1.8 Application of PHOX in Asymmetric Heck Reaction

**Scheme 63:**
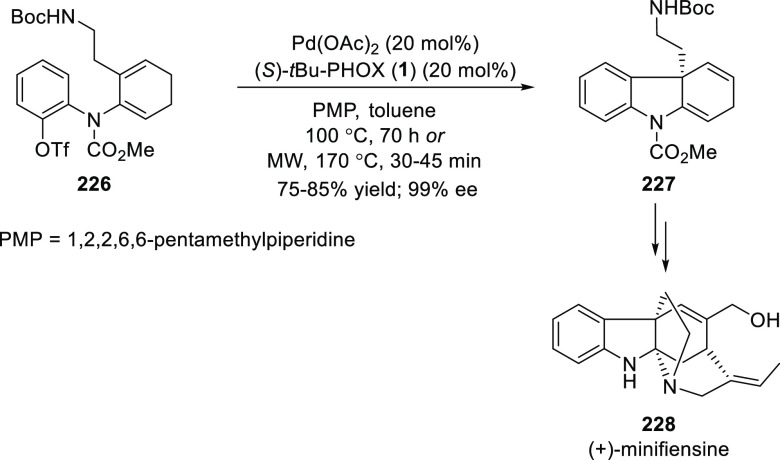
Asymmetric Heck-Cyclization of Dienyl Aryl Triflate **226**

Overman and Wrobleski reported a synthesis of (+)-minifiensine (**228**), which involves an asymmetric Heck-cyclization of dienyl aryl triflate **226** using a catalytic amount of Pd and (*S*)-*t*Bu-PHOX (**1**) as the key step to provide dihydrocarbazole **227** in 99% *ee* (Scheme 63).^77^ It is noteworthy that microwave heating (170 °C) was employed to accelerate this catalytic transformation.

**Scheme 64:**
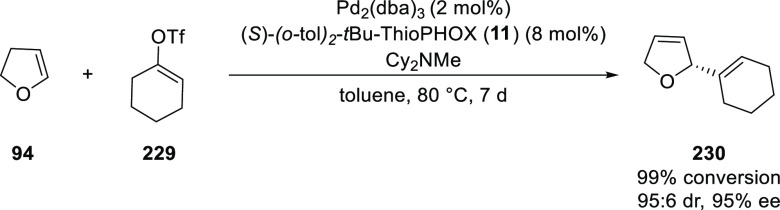
Intermolecular Asymmetric Heck Reaction

In 2009, Guiry reported a Pd-catalyzed intermolecular asymmetric Heck reaction between 2,3-dihydrofuran (**94**) and a range of triflates using HetPHOX ligands (Scheme 64).^78^ The HetPHOX ligand derived from *tert*-leucinol and di-*o*-tolylphosphine, (*S*)-(*o*-tol)_2_-*t*Bu-ThioPHOX (**11**) proved the most effective affording *ee* values of up to 96, 95, and 94% in the phenylation, cyclohexenylation (using triflate (**229**)), and naphthylation, respectively, of 2,3-dihydrofuran.

**Scheme 65:**
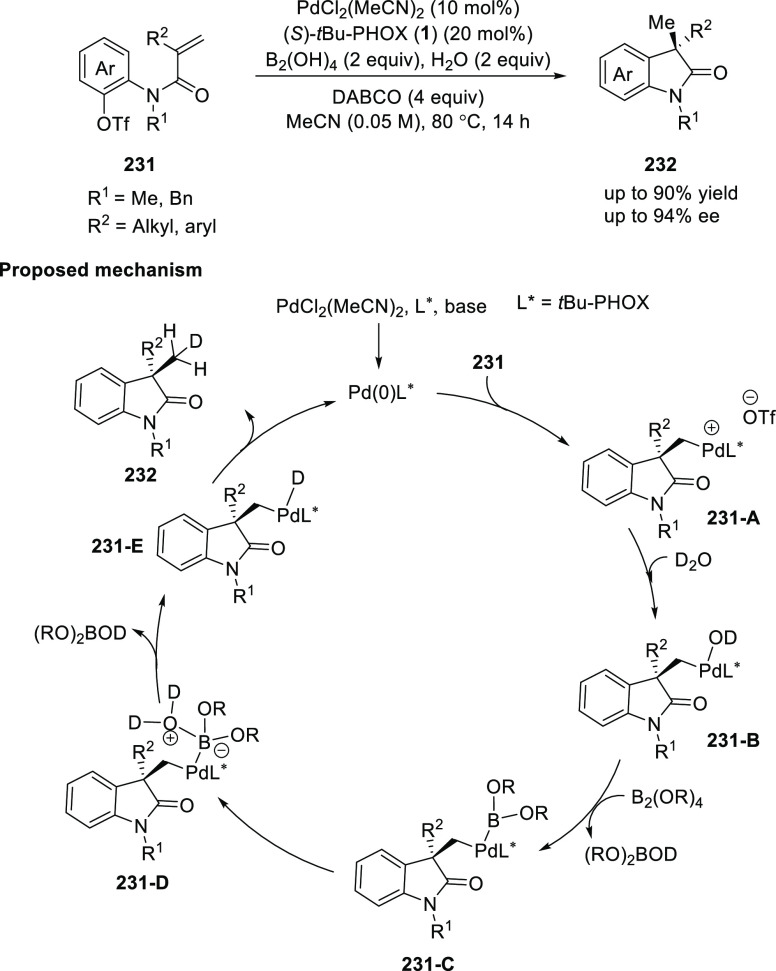
Asymmetric Reductive Heck Reaction

Zhu reported an asymmetric reductive Heck reaction using a catalytic amount of Pd, (*S*)-*t*Bu-PHOX (**1**) and diboron-H_2_O as a hydride source (Scheme 65).^79^ This method was applied to synthesize a library of enantioenriched oxindoles (**232**) possessing a C3-quaternary stereocenter in excellent yields and enantioselectivities. Alkenes possessing aryl and alkyl groups at R^2^ were compatible. The use of D_2_O instead of H_2_O as a D-donor enabled the synthesis of CH_2_D substituted oxindoles. The catalytic cycle initiates with oxidative addition of the *t*Bu-PHOX (**1**) ligated Pd(0) to aryl triflate **231**, followed by intramolecular carbopalladation, generating a cationic alkyl-Pd(II) intermediate **231-A** which reacts with B_2_(OH)_4_ and H_2_O in a series of steps to afford alkyl-Pd(II)-H species **231-E** Finally, reductive elimination from **231-E** provides the desired product **232** with simultaneous regeneration of the Pd(0)/*t*Bu-PHOX (**1**) catalyst.

**Scheme 66:**
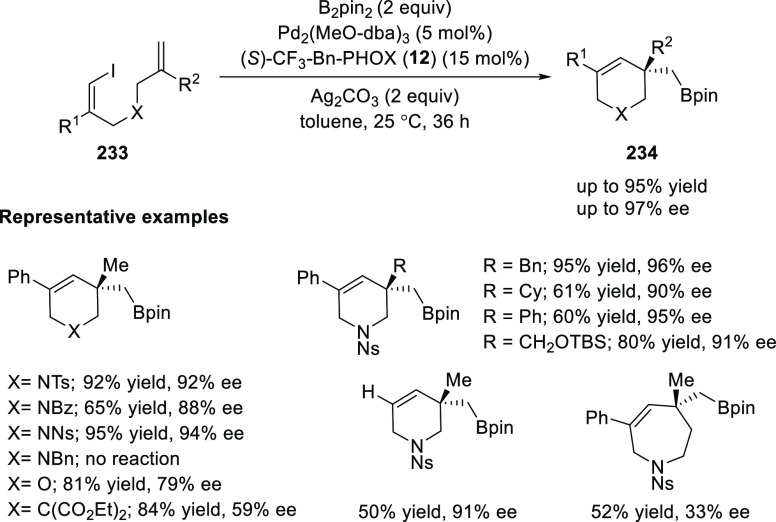
Asymmetric Vinylborylation

Tong developed a Pd-catalyzed asymmetric vinylborylation of (*Z*)-1-iodo-diene (**233**) with B_2_Pin_2_ using (*S*)-CF_3_–Bn-PHOX (**12**) (Scheme 66).^80^ This method allowed them to synthesize a variety of 3,3-disubstituted tetrahydropyridines (**234**) derivatives in good yields and high enantioselectivities. The choice of the tether between vinyl iodide and the alkene had a strong effect on the reaction outcome, as a tether with a strong coordinating atom with Pd had a positive effect and this protocol was limited to the synthesis of a 6-membered cyclic product. This reaction begins by the oxidative addition of Pd(0) to vinyl iodide followed by a Ag-mediated transmetalation with B_2_Pin_2_ to form a vinyl-Pd(II)-Bpin species which, upon intramolecular enantioselective carbopalladation followed by reductive elimination, furnished the expected product. Mechanistic studies revealed that the transmetalation step occurs before the alkene insertion, which was complementary to the previous understanding of a similar reaction.

**Scheme 67:**
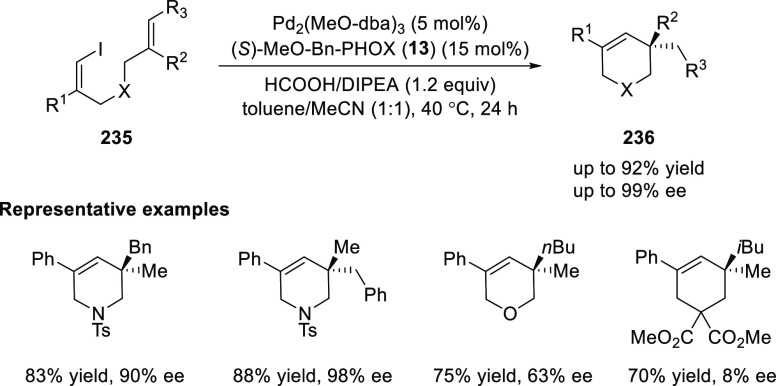
Asymmetric Reductive Heck Cyclization

In 2017, Tong developed an asymmetric reductive Heck cyclization of (*Z*)-1-iodo-1,6-dienes (**235**) using Pd_2_(dba-OMe)_3_ and (*S*)-MeO-Bn-PHOX (**13**) as the ligand (Scheme 67).^81^ This method provided a facile access to chiral quaternary tetrahydropyridines (**236**) with good to excellent yields and enantioselectivities. Additionally, this method tolerates susceptible β-hydrogen elimination and a substrate bearing a trisubstituted alkene. The linker between the vinyl iodides and alkenes as well as substituent R^1^, R^2^, and R^3^ had a strong influence on the yield and enantioselectivity of product.

##### 2.1.1.9 Application of PHOX in Asymmetric Alkylation

**Scheme 68:**
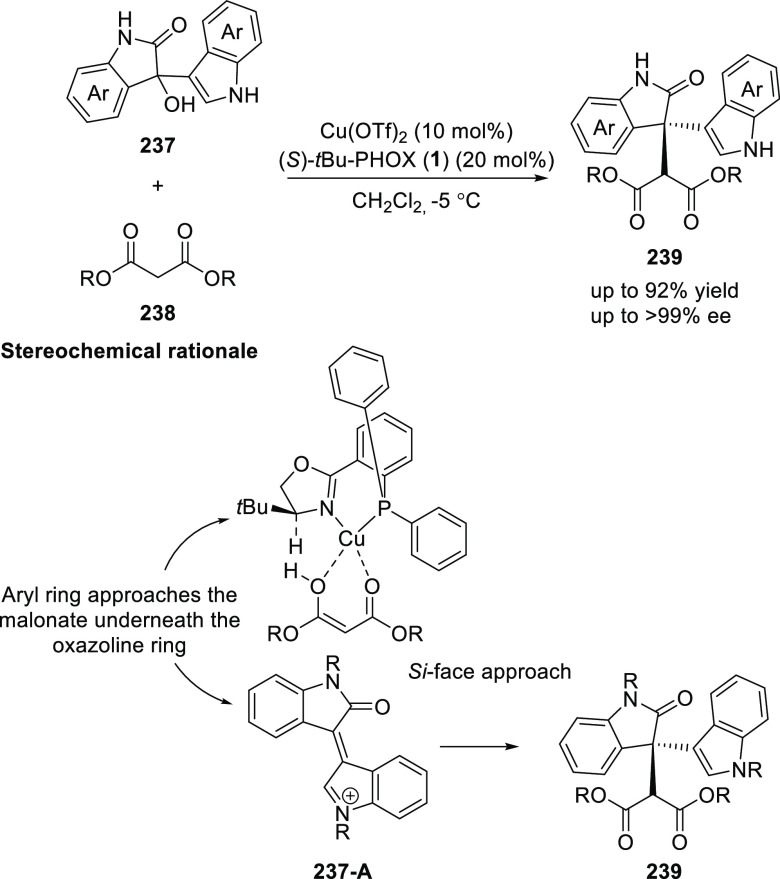
Asymmetric Alkylation of 3-Hydroxy-2-oxindole

In 2018, Bisai reported a Cu-catalyzed asymmetric alkylation of 3-hydroxy-2-oxindole (**237**) with a variety of malonates (**238**) using the *t*Bu-PHOX (**1**) ligand (Scheme 68).^82^ The presence of an indole moiety at C3 is critical for success and no reaction occurred when indole was replaced with an alkyl group. This observation suggested *in situ* formation of a highly reactive intermediate **237-A** followed by an enantioselective malonate addition. The mechanistic studies confirmed that the reaction is reversible in nature and the involvement of a Cu(II)-complex with a distorted trigonal bipyramid (TBP) geometry.

**Scheme 69:**
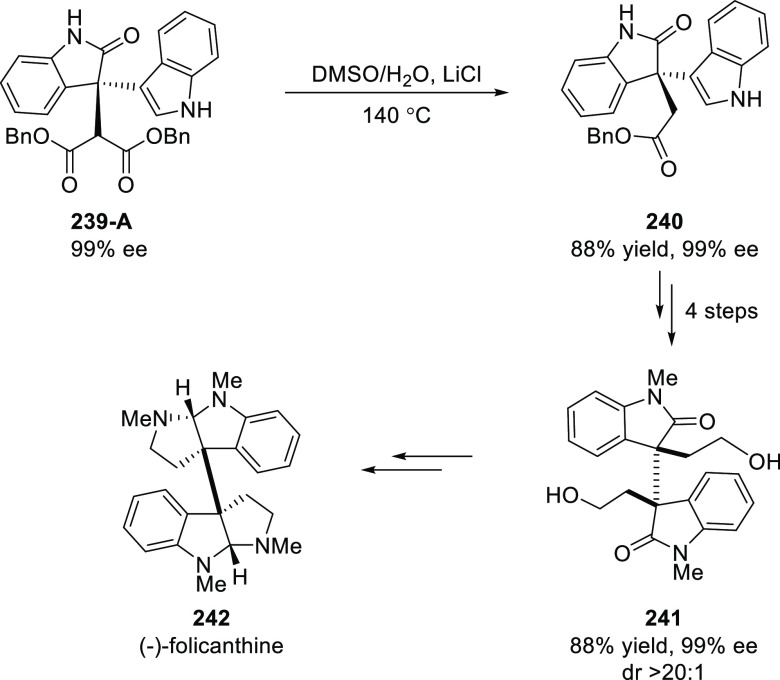
Synthesis of (−)-Folicanthine

The application of the method was illustrated in the formal total synthesis of (−)-folicanthine (**242**) by synthesizing a C_2_-symmetric dimeric 2-oxindole **241** possessing an all-carbon quaternary stereocenter in 5 steps (Scheme 69).

##### 2.1.1.10 Application of PHOX in Desymmetrizing Cross-Coupling

**Scheme 70:**
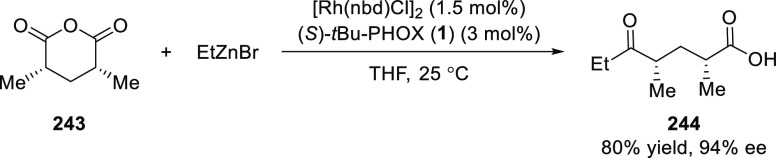
Desymmetrizing Cross-Coupling

Rovis reported a Rh/*t*Bu-PHOX (**1**)-catalyzed cross-coupling between sp^3^ organozinc reagents and 3,5-dimethylglutaric anhydride (**243**) (Scheme 70).^83^ A variety of *syn*-deoxypolypropionates (**244**) were synthesized in excellent yields and good to excellent enantioselectivities. This catalytic system worked well with alkyl and benzyl zinc reagents possessing various functionalities, however it was less efficient with phenyl and isopropyl zinc reagents.

##### 2.1.1.11 Application of PHOX in Asymmetric Conjugate Addition

**Scheme 71:**
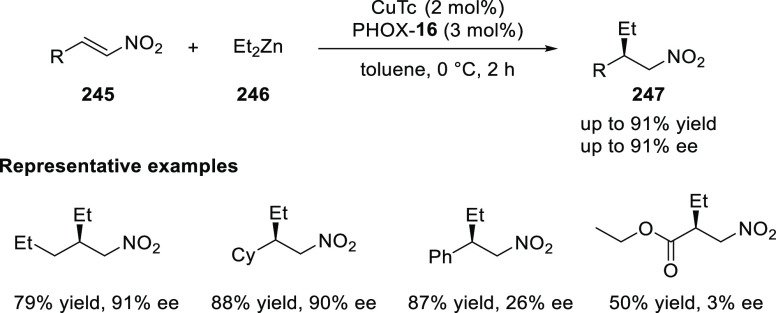
Asymmetric Conjugate Addition

Jung reported a Cu-catalyzed asymmetric conjugate addition of alkylzinc reagents to nitroalkenes (**245**) and cyclic enones by using the newly synthesized *P*,*N*-ligand **16** (Scheme 71).^84^ A variety of chiral nitroalkanes (**247**) and β-alkyl cyclic ketones were synthesized in good yields and good enantioselectivity (up to 92% yield and 95% *ee*). Computational studies suggested that the chiral substituents in the oxazoline moiety of *P*,*N*-ligand **16** are not crucial for the selectivity whereas the other alkyl substituent in the bridging moiety is involved in the steric repulsion during the transition state.

Metal complexes of PHOX ligands with one stereocenter (**1**–**17**) have been extensively studied in the past decade. These complexes have been successfully applied in various asymmetric transformations. Palladium complexes were excellent for (I) decarboxylative allylic alkylation and protonation, (II) Heck reaction, (III) hydroamination and alkylation of diene, (IV) [3 + 2] cycloaddition, (V) Meerwein-Eschenmoser and Saucy-Marbet Claisen rearrangement, (VI) aryl boronic acid addition to various imines. Iridium complexes were mostly utilized in hydrogenation of diverse types of alkenes and imines wheras nickel complexes showed excellent results when employed in annulation reactions between aryl boronic acids and alkynes, allenes and imines. Cobalt complexes were specifically successful in (I) intramolecular reactions between *o*-imodoylarylboronic acids or aryl halides or pseudohalides with alkynes, and (II) [2 + 2] cycloadditions between 1,3-enynes and ethylene. Copper complexes were successfully employed in (I) asymmetric alkylations of 3-hydroxy-2-oxindole, and (II) asymmetric conjugate additions of alkylzinc reagents to nitroalkenes while rhodium complexes found their application in desymmetrizations of 3,5-dimethylglutaric anhydride with alkyl zinc bomides. It is evident from the literature covered in this section of the review that PHOX ligands with only one stereocenter are adaptable with various metals and hence researchers should seek to continue to expand their utilization in asymmetric catalysis.

#### 2.1.2 Phosphinooxazoline Ligands with Stereoaxis or Stereocenter

**Figure 3:**
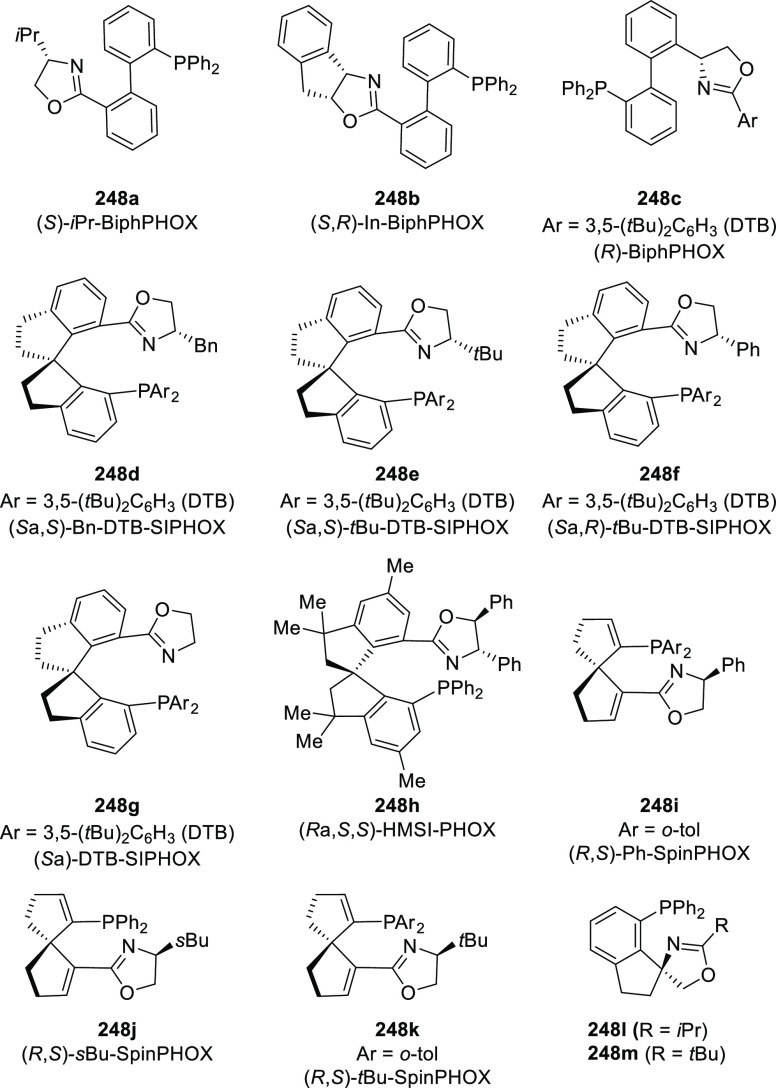
Phosphinooxazoline ligands with a stereocenter or stereoaxis.

This section deals with the application of phosphinooxazoline ligands (**248a**–**m**) possessing a stereoaxis or stereocenter in metal-catalyzed enantioselective reactions (Figure 3).

##### 2.1.2.1 BiphPHOX Ligands

BiphPHOX ligands (**248a**–**b**) possess a chiral oxazoline ring and exist as an equilibrium mixture of diastereomers in solution due to rotation around the internal bond of the biphenyl groups. Interestingly, when these ligands coordinate to Pd or Ir, only one of two possible diastereomeric complexes are formed. The Zhang group extensively utilized the metal-complexes of BiphPHOX ligands (**248a**–**b**) in the Ir-catalyzed asymmetric hydrogenation of variably substituted olefins and in the Ni-catalyzed arylation of cyclic aldimines and ketimines.

**Scheme 72:**
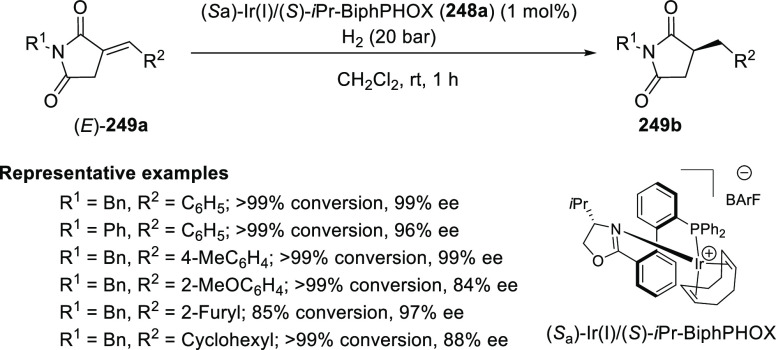
Asymmetric Hydrogenation of α-Alkylidene Succinimides

In 2013, Zhang reported the first asymmetric hydrogenation of α-alkylidene succinimides (**249a**) using an Ir/(*S*)-*i*Pr-BiphPHOX (**248a**) complex (1 mol %) to afford hydrogenated products (**249b**) in excellent yields (>99%) and enantioselectivities (up to 99% *ee*) under 20 bar H_2_ (Scheme 72).^85^ The reactions performed under reduced catalytic loading (0.05 mol %) and reduced pressure (1 bar) were successful although at the expense of a prolonged reaction time. The *i*Pr substituent on the oxazoline ring had a strong impact on the enantioselectivity (99% *ee*). The α-alkylidene succinimide (**249a**) with (*E*)-configuration was essential for the success of hydrogenation whereas the *N*-protecting group did not affect the yield and enantioselectivity of the reaction.

**Scheme 73:**
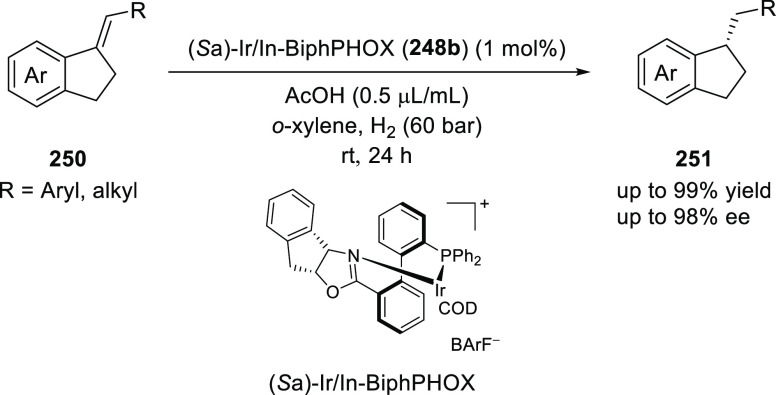
Asymmetric Hydrogenation of Unfunctionalized Exocyclic Alkenes

In a subsequent study, Zhang described the first asymmetric hydrogenation of unfunctionalized exocyclic C=C bonds (**250**) with Ir and In-BiphPHOX (**248b**) delivering the expected chiral 1-benzyl-2,3-dihydro-1H-indene products (**251**) in up to 98% *ee* (Scheme 73).^86^ The use of coordinating solvents like THF and dioxane dramatically decreased the conversion by deactivating the Ir catalyst while the additive acetate ion plays a crucial role in improving the enantioselectivity.

**Scheme 74:**
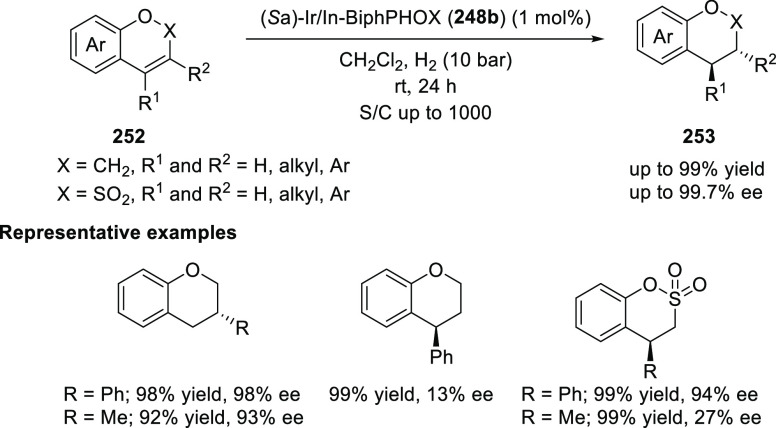
Asymmetric Hydrogenation of Substituted 2*H*-Chromenes and Benzo[*e*][1,2]oxathiine 2,2-Dioxides

Afterward, Zhang reported the asymmetric hydrogenation of substituted 2*H*-chromenes and substituted benzo[*e*][1,2]oxathiine 2,2-dioxides (**252**) (Scheme 74).^87^ A variety of 2*H*-chromenes possessing 3-aryl/alkyl substituents were hydrogenated to the corresponding chiral 3-substituted chromanes (**253**) in high yields (92–99%) with excellent enantioselectivities (>99% *ee*). The 4-phenyl substituent on 2*H*-chromenes also gave the corresponding product in excellent yield but with poor enantioselectivity. Interestingly, benzo[*e*][1,2]oxathiine 2,2-dioxides with phenyl and methyl substituents produced the desired products in excellent yields but with contrasting enantioselectivity for phenyl (94% *ee*) and methyl substituents (27% *ee*).

**Scheme 75:**
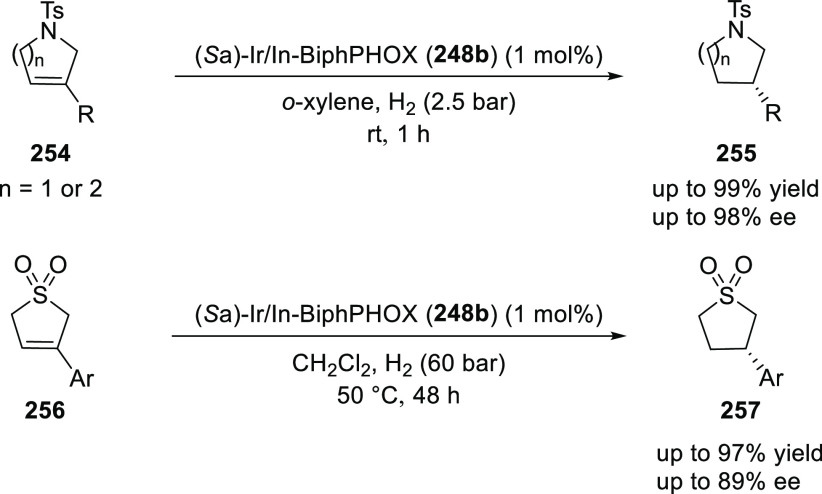
Asymmetric Hydrogenation of 3-Substituted 2,5-Dihydropyrroles and 2,5-Dihydrothiophene 1,1-Dioxides

Similarly, Zhang extended their catalytic system for the asymmetric hydrogenation of 3-substituted 2,5-dihydropyrroles (**254**) and 3-substituted 2,5-dihydrothiophene 1,1-dioxides (**256**) (Scheme 75).^88^ The hydrogenation of 3-substituted 2,5-dihydrothiophene 1,1-dioxides was more challenging compared to the 3-substituted 2,5-dihydropyrroles and hence, a higher temperature and hydrogen pressure were required to achieve full conversions.

**Scheme 76:**
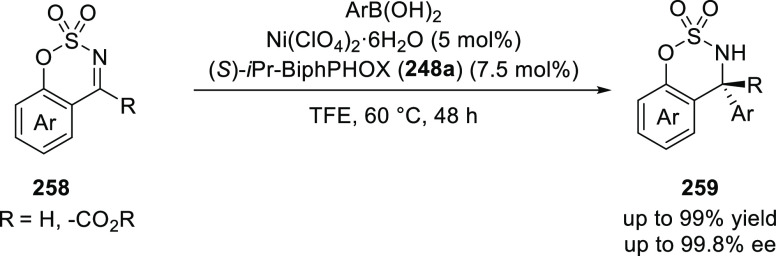
Asymmetric Arylation of Cyclic Aldimines and Ketimines

Later on, Zhang employed *i*Pr-BiphPHOX (**248a**) in a Ni(II)-catalyzed asymmetric addition of arylboronic acids to cyclic aldimines and ketimines (**258**) furnishing products with excellent yields (up to 99%) and enantioselectivities (>99% *ee*) (Scheme 76).^89^ Ligand *i*Pr-BiphPHOX (**248a**) coordinates with Ni(II) to form a complex with (*S*)-axial chirality, while the complex with (*R*)-axial chirality was disfavored because of the steric hindrance of the *i*Pr group and the anion coordinated to Ni(II). Crystallographic studies confirmed the (*S*)-configuration and tetrahedral geometry of the Ni(II) complex. Interestingly, DFT calculations suggested that in solution the configuration of the Ni(II) complex changes from tetrahedral to planar.

Ligand **248c** is architecturally very similar to BiphPHOX (**248a**–**b**) and it has been applied in the asymmetric hydrogenation of unsaturated acids.

**Scheme 77:**
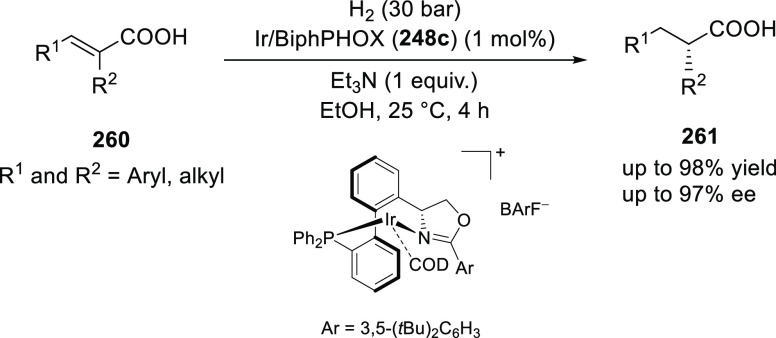
Asymmetric Hydrogenation of α,β-Unsaturated Acids

In 2017, Zhang developed a series of highly modular phosphinooxazoline ligands from readily available (*S*)-(+)-2-phenylglycinol. These ligands were utilized in the Ir-catalyzed asymmetric hydrogenation of α,β-unsaturated carboxylic acids (**260**) to provide chiral α-substituted carboxylic acids (**261**) (up to 97% *ee*, 98% yield, 2000 TON) (Scheme 77).^90^ Ligand **248c** possessing a 3,5-(*t*Bu)_2_C_6_H_3_ substituent on the oxazoline was found to be optimal. Changing the 3,5-(*t*Bu)_2_C_6_H_3_ substituent to a phenyl or a *t*Bu drastically reduced the reactivity and enantioselectivity in hydrogenation. The role of Et_3_N as an additive was crucial as the reactivity dropped dramatically without it.

##### 2.1.2.2 SIPHOX Ligands

SIPHOX ligands (**248d**–**g**) have been employed in the Ir-catalyzed asymmetric hydrogenation of olefins, Pd-catalyzed Narasaka-Heck cyclization, Pd-catalyzed enantioselective formal [6 + 4] cycloaddition of vinyl oxetanes with azadienes and Ni-catalyzed enantioselective arylation of cyclic *N*-sulfonyl imines.

**Scheme 78:**
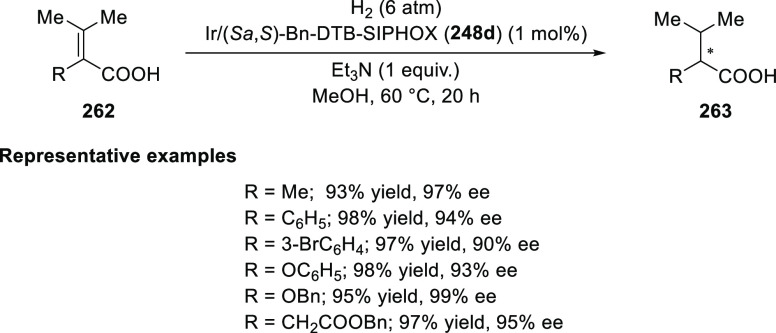
Asymmetric Hydrogenation Reaction of α,β-Unsaturated Carboxylic Acids

Zhou reported the asymmetric hydrogenation of α,β-unsaturated carboxylic acids (**262**) possessing tetra-substituted olefins using Ir and chiral *P*,*N*-ligands (**248d**) based on the spiro-backbone (Scheme 78).^91^ This method offers a direct approach to chiral carboxylic acids possessing an α-stereocenter having aryl, alkyl, aryloxy, and alkyloxy substituents. The substituents on both phosphorus (3,5-di-*t*-butylphenyl) and the oxazoline ring of **248d** had a strong effect on the outcome of the reaction. A chiral induction model was proposed which showed that the (*R*)-aryloxy or (*R*)-alkyloxy-β,β-dimethyl acrylic acids **262** prefer to coordinate to catalyst (*S*a,*S*)-**248d** with *Re*-facial selectivity and consequently generates products **263** preferably with (*S*)-configuration.

**Scheme 79:**
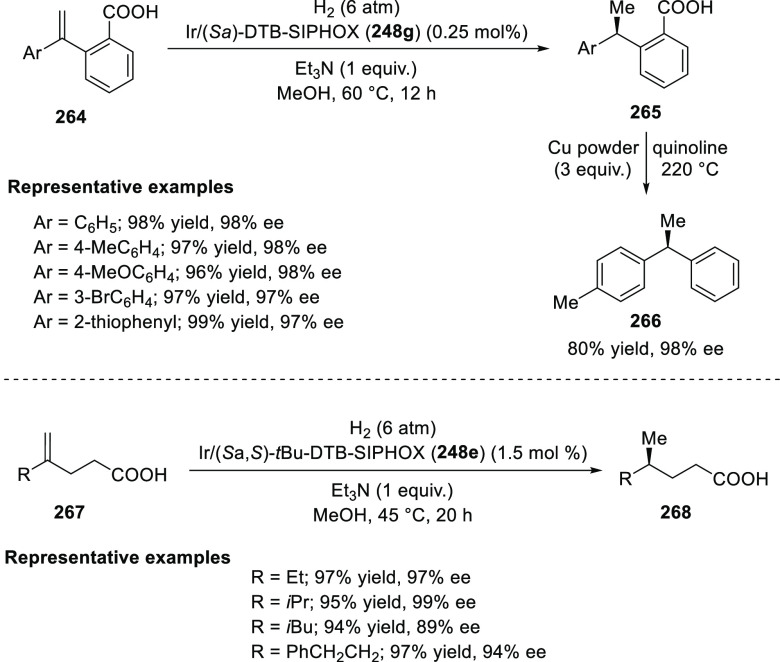
Enantioselective Hydrogenation

In 2013, Zhou extended the Ir-catalyzed enantioselective hydrogenation strategy to 1,1-diarylethenes (**264**) and 1,1-dialkylethenes (**267**) by using a carboxylic acid as a directing group (Scheme 79).^92^ The carboxylic acid group is essential for the success of this method as no reaction was observed without it or with the corresponding ester. The diphenylethene substrates having a carboxylic acid group either at the *meta*- or *para*-position could not be hydrogenated under these reaction conditions. Importantly, no reaction occurred in the absence of the base triethylamine. These experiments revealed that the carboxylic acid reacts with a base and acts as an anchor by coordinating with the Ir catalyst. This enables the catalyst to discriminate between the prochiral faces of the substrates and catalyze their hydrogenation with high levels of enantioselectivity.

**Scheme 80:**
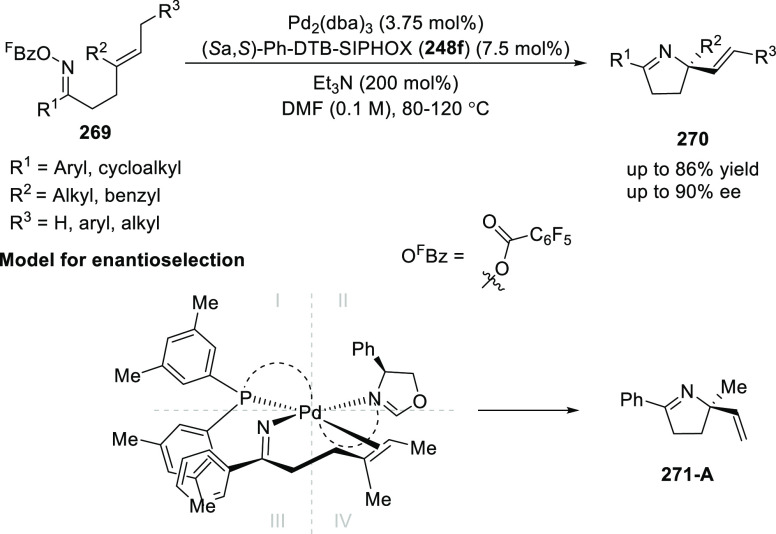
Enantioselective Narasaka–Heck Cyclization

In 2017, Bower developed a Pd-catalyzed highly enantioselective Narasaka-Heck cyclization starting from oxime esters with tethered trisubstituted alkenes (**269**) using Spinol-derived chiral ligands **248f** (Scheme 80).^93^ This method provides access to dihydropyrrole derivatives (**270**) with a nitrogen-containing stereocenter in up to 86% yield and 90% *ee*. It is important to note that the alkene geometry is criticial for reaction success, as cyclization with the (*Z*)-isomer proceeds with considerably lower *ee*s and yields compared to the corresponding (*E*)-isomer. Additionally, alkenes with 1,2-disubstitution resulted in a mixture of dihydropyrrole and pyrrole products due to competing β-hydride elimination. The proposed reaction mechanism suggested the enantioselective migratory insertion of alkenes into Pd–N bond was the key step.

**Scheme 81:**
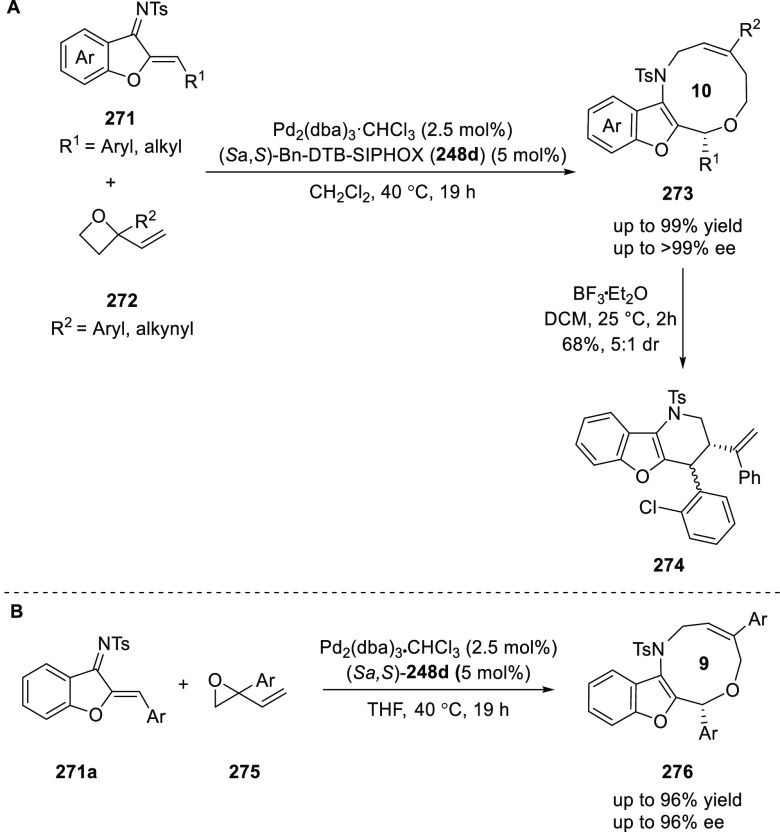
Enantioselective Formal [6 + 4] Cycloaddition of Vinyl Oxetanes with Azadienes

Zhao reported a highly enantioselective synthesis of benzofuran/indole-fused ten-membered heterocycles (**273**) via a formal [6 + 4] cycloaddition of vinyl oxetanes (**272**) with azadienes (**271**) (Scheme 81A).^94^ A wide range of benzofuran- and indole-fused heterocycles were accessed in excellent yields and enantioselectivities. The use of aryl- and alkynyl-substituted vinyl oxetanes as substrates was successful whereas an alkyl-substituted vinyl oxetane failed to furnish the desired product, possibly due to the difficulty in the formation of the Pd-π-allyl intermediate. Furthermore, a unique fragmentation of these ten-membered heterocycles (**273**) was achieved under Lewis acid catalysis to furnish a thermodynamically more favorable six-membered ring (**274**). Additionally, this method was also employed to generate nine-membered heterocycles (**276**) by reacting azadiene (**271a**) with vinyl epoxide (**275**) via a formal [5 + 4] cycloaddition reaction (Scheme 81B).

**Scheme 82:**
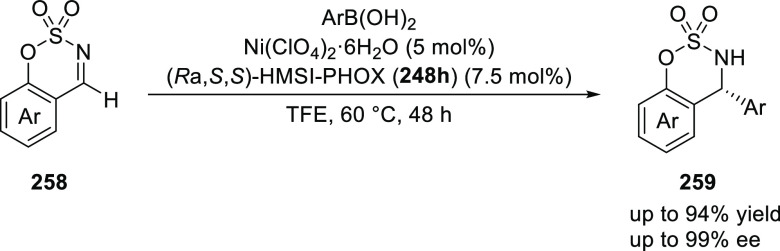
Enantioselective Arylation of Cyclic *N*-Sulfonyl Imines

Lin reported a Ni-catalyzed enantioselective arylation of cyclic *N*-sulfonyl imines (**258**) with arylboronic acids (Scheme 82).^95^ A newly developed chiral phosphinooxazoline ligand, (*Ra*,*S*,*S*)-**248h**, based on the hexamethyl-1,1′-spirobiindane backbone (HMSI-PHOX), which is a derivative of SIPHOX, provided optically active amines in high yields (up to 94%) and enantioselectivities (up to 99% *ee*).

##### 2.1.2.3 SpinPHOX Ligands

SpinPHOX ligands (**248i**–**k**) have been employed in the Ir-catalyzed asymmetric hydrogenation of α-aryl-β-substituted acrylic acids, exocyclic α,β-unsaturated carbonyl compounds, and 3-ylidenephthalides.

**Scheme 83:**
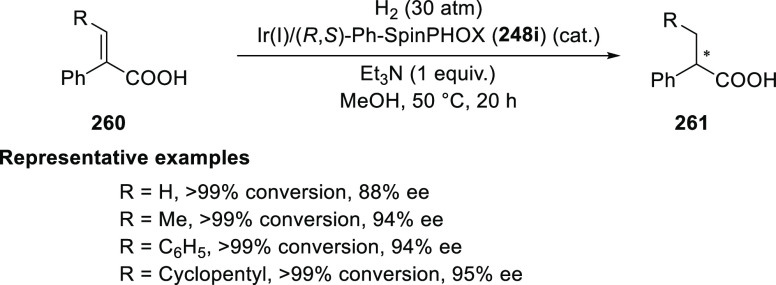
Enantioselective Hydrogenation α-Aryl-β-Substituted Acrylic Acids

Ding reported the enantioselective hydrogenation of a series of α-aryl-β-substituted acrylic acids (**260**) by using an Ir-(*R*,*S*)-SpinPHOX (**248i**) based catalytic system (Scheme 83).^96^ A series of biologically interesting α-aryl acetic acids (**261**) were synthesized in excellent yields and enantioselectivity (up to 96% *ee*). The addition of triethylamine as a basic additive was essential for both conversion and enantioselectivity due to the poor solubility and potential impact of the carboxylate group on the catalysis. Additionally, hydrogenation of the (*Z*)-isomer with (*R*,*S*)-SpinPHOX (**248i**) was not as successful as the conversion to products was too low. It was found that the chirality at the spiro backbone of (*R*,*S*)-SpinPHOX (**248i**) had a significant impact on the asymmetric induction of the reaction. The (*R*)-configuration in the spiro backbone and an (*S*)-configuration on the oxazoline component was found to be the matched ligand whereas the corresponding diastereomeric ligand (*S*,*S*)-SpinPHOX gave lower conversion and enantioselectivity for the opposite enantiomer.

**Scheme 84:**
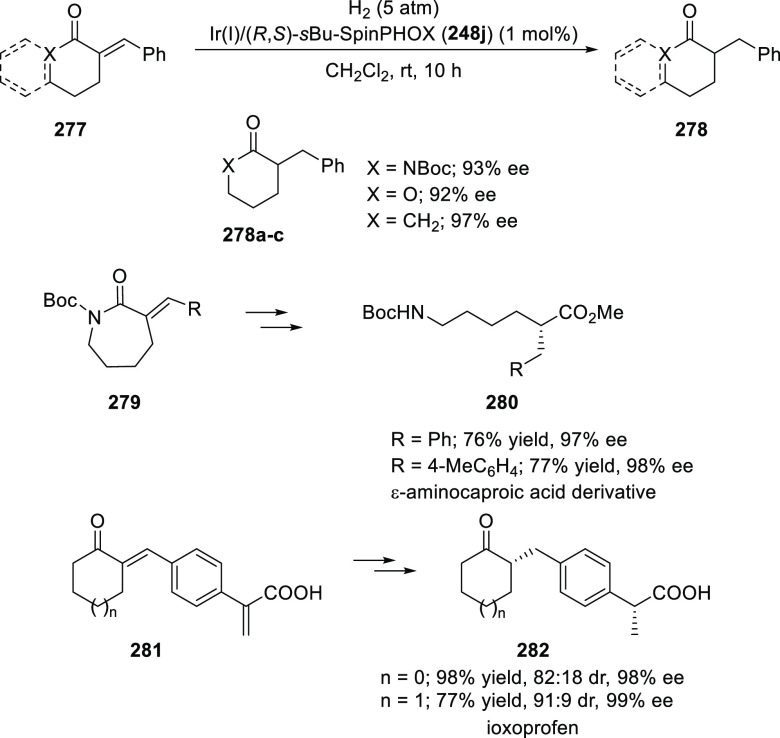
Enantioselective Hydrogenation of Exocyclic α,β-Unsaturated Carbonyl Compounds

The Ir/SpinPHOX (**248j**) catalytic system was extended to the enantioselective hydrogenation of six- and seven-membered exocyclic α,β-unsaturated carbonyl compounds (**277**) with a focus on the challenging lactam derivatives (Scheme 84).^97^ A series of optically active carbonyl compounds (**278**) such as lactams, lactones and cyclic ketones with an α-chiral carbon stereocenter was prepared in excellent enantiomeric excess (up to 97%). An excellent stereoselectivity was observed when either (*R*,*S*)-**248j** or (*S*,*S*)-**248j** were employed in the reaction and opposite enantiomers were formed preferentially, which indicated that the spiro chirality of the ligand primarily controls the sense of asymmetric induction while the chirality of the oxazoline moiety might have some influence on the level of enantioselectivity. Additionally, the application of this methodology was shown by synthesizing an ε-aminocaproic acid derivative (**280**) and the nonsteroidal anti-inflammatory drug loxoprofen (**282**).

**Scheme 85:**
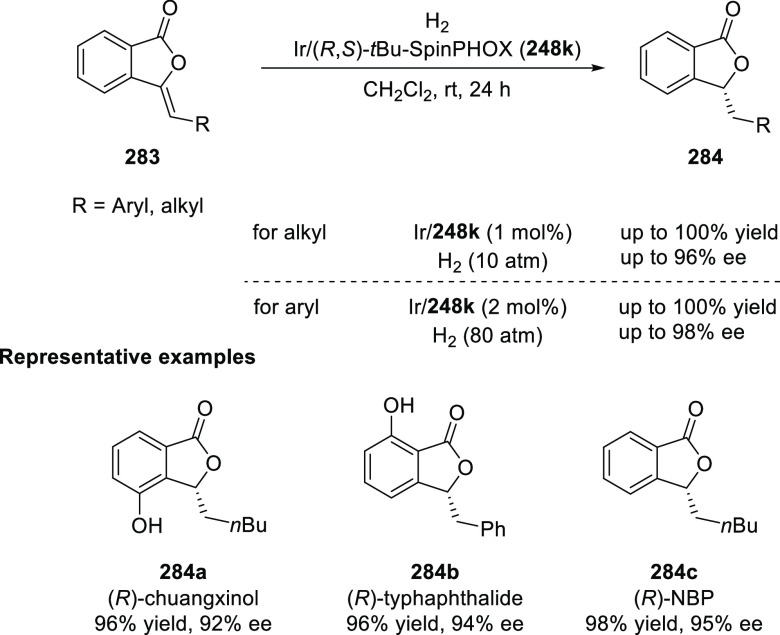
Enantioselective Hydrogenation of 3-Ylidenephthalides

In 2018, Ding reported the enantioselective hydrogenation of 3-ylidenephthalides (**283**) by using an Ir/SpinPHOX (**248k**) catalyst (Scheme 85).^98^ This method provides a straightforward approach to a wide variety of 2-substituted chiral phthalides (**284**) in high yields with excellent enantioselectivities (up to >99% yield and 98% *ee*). The application of this methodology was demonstrated in the synthesis of chiral drugs and natural products such as (*R*)-chuangxinol (**284a**), (*R*)-typhaphthalide (**284b**), and (*S*)-3-*n*-butylphthalide (NBP) (**284c**), a constituent of celery seed oil.

**Scheme 86:**
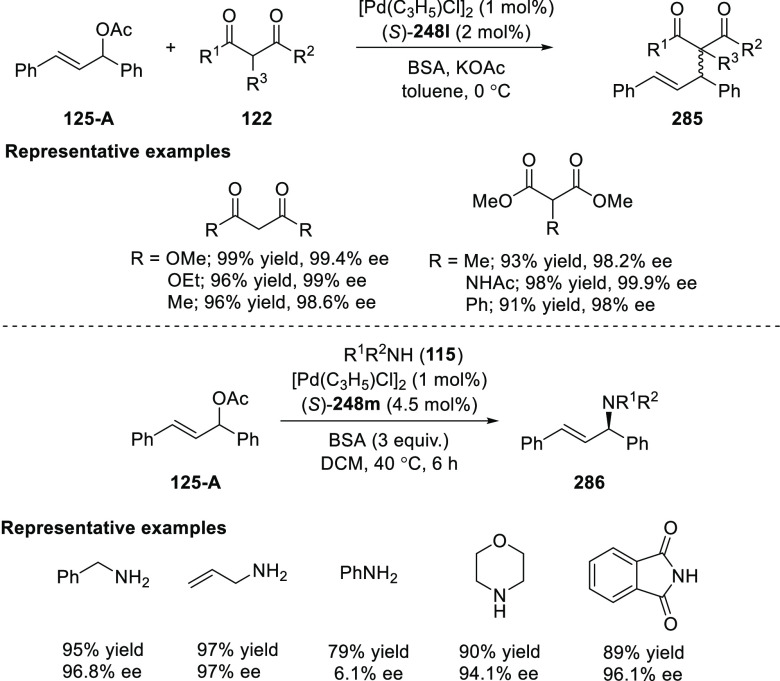
Pd-Catalyzed Asymmetric Allylic Alkylation and Amination

In 2018, Teng reported a new rigid phosphinooxazoline ligand (**248l,m**) possessing a chiral spiro core and it was applied in Pd-catalyzed asymmetric allylic alkylation and amination with *ee*s up to 99% (Scheme 86).^99,100^

To conclude this section, metal-complexes of PHOX ligands with Stereoaxis or Stereocenter (**248a**–**m**) have been employed in various asymmetric transformations. Iridium complexes were successful in the hydrogenation of a variety of alkenes whereas nickel complexes were excellent when used in the addition of aryl boronic acids to imines. Palladium complexes found their application in (I) Narasaka-Heck cyclization, (II) [6 + 4] cycloadditions of vinyl oxetanes with azadienes, and (III) allylic alkylation and amination.

#### 2.1.3 Phosphinooxazoline Ligands with a Stereoplane

**Figure 4:**
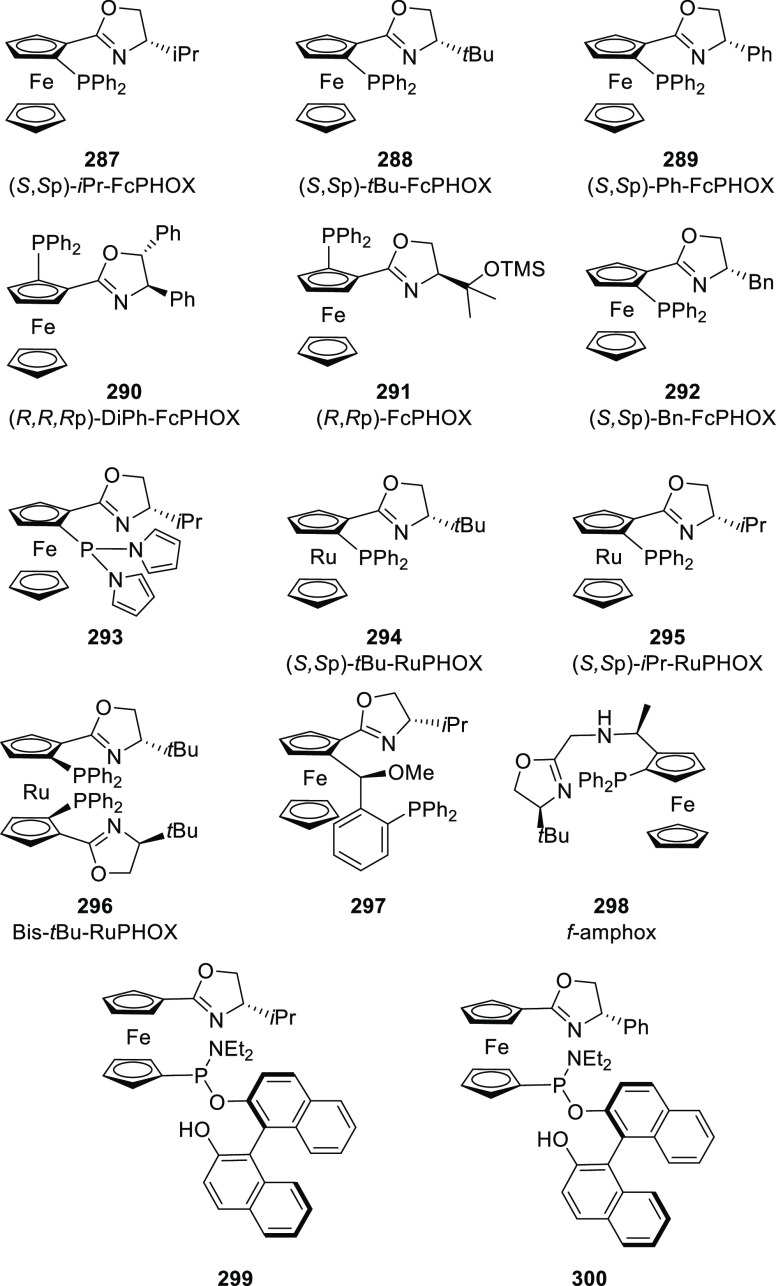
FcPHOX ligands.

Ferrocene phosphinooxazoline (FcPHOX) ligand (other acronyms include FOXAP, Phosferrox, FOX) is the major contributor in this category of *P*,*N*-ligands (Figure 4). This ligand was first reported by Richards and Uemura in 1993 for Pd-catalyzed asymmetric allylic alkylation and since then has been employed in a myriad of metal-catalyzed asymmetric transformations.^101^

##### 2.1.3.1 FcPHOX Ligands for Asymmetric Cycloaddition Reactions

**Scheme 87:**
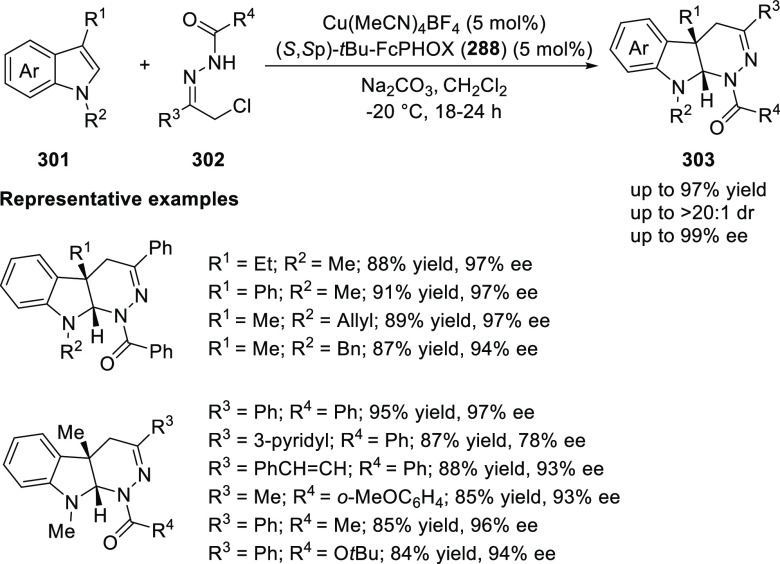
Asymmetric Inverse-Electron-Demand *aza*-Diels–Alder Reaction of Indoles

In 2014, Wang developed a Cu/(*S*,*S*p)-*t*Bu-FcPHOX (**288**)-catalyzed asymmetric inverse-electron-demand *aza*-Diels–Alder reaction of indoles (**301**) with *in situ* formed azoalkenes (Scheme 87).^102^ This method provided access to a variety of biologically important [2,3]-fused indoline tetrahydropyridazine heterocycles (**303**) in good yields (up to 97%) with high regioselectivity and diastereoselectivity (>20:1 dr), and excellent enantioselectivity (up to 99% *ee*). The success of this reaction lies with the coordination of the chiral Cu/FcPHOX (**288**) complex to the *in situ* formed azoalkene.

**Scheme 88:**
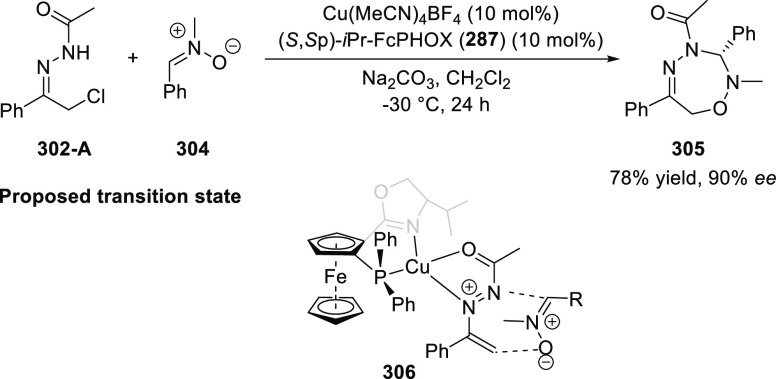
Asymmetric 1,3 Dipolar [3 + 4] Cycloaddition with Nitrones

Later, Wang successfully employed azoalkenes in an unprecedented Cu/(*S*,*S*p)-*i*Pr-FcPHOX (**287**)-catalyzed asymmetric 1,3-dipolar [3 + 4] cycloaddition with nitrones (**304**) to generate rapid access to biologically important 1,2,4,5-oxatriazepanes (**305**) in excellent regioselectivity and enantioselectivity (Scheme 88).^103^ Interestingly, the [3 + 2] cycloadducts which could have potentially been formed were not observed. Alkyl-substituted nitrones resulted in only the racemic products and *ortho*-substituted aryl hydrazones and alkyl α-chloro-*N*-acyl hydrazone were not suitable substrates for this reaction. A proposed transition state **306** depicts that the *in situ* generated azoalkene coordinates with Cu through nitrogen and oxygen. The bulky isopropyl group of the oxazoline ring blocks the backside of the coordinated azoalkene, and hence the nitrone approaches the R group to avoid unfavorable steric congestion between the diphenylphosphine group in the chiral ligand and the R group of the nitrone.

**Scheme 89:**
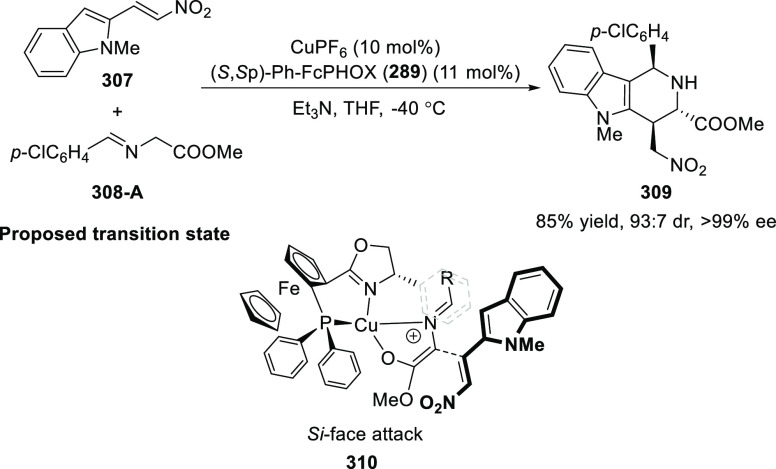
Asymmetric [3 + 3] Cycloaddition of Azomethine Ylides with 2-Indolylnitroethylenes

Deng reported the first Cu/(*S*,*S*_p_)-Ph-FcPHOX (**289**)-catalyzed asymmetric [3 + 3] cycloaddition of azomethine ylides (**308-A**) with 2-indolylnitroethylenes (**307**) which afforded the highly substituted tetrahydro-γ-carboline derivatives (**309**) in moderate to high yields, and excellent levels of stereoselectivity (up to >98:2 dr, > 99% *ee*) (Scheme 89).^104^ This reaction is highly chemoselective toward the formation of the [3 + 3] cycloadduct over the [3 + 2] cycloadduct (up to 94:6 ratio). An alkyl-substituted azomethine ylide afforded the [3 + 2] cycloadduct in 87% yield instead of the expected [3 + 3] cycloadduct, presumably due to the less-reactive nature of the aliphatic imine. Because of the steric effects of the bulkier phenyl group in the oxazoline ring and the diphenylphosphine group of the ligand, the reaction is proposed to involve *Si*-face attack of nucleophilic complex **310**, generated *in situ* from the chiral Cu/FcPHOX (**289**) and azomethine ylide (**308-A**).

During the Michael addition, the carbanion generated adjacent to the nitro group is stable enough at lower temperature and therefore suppresses the Mannich cyclization which gives the [3 + 2] cycloaddition product. Also, similar levels of stereoselectivity were observed for both the [3 + 3] and [3 + 2] cycloadducts suggesting that the Michael addition should be the crucial step for asymmetric induction.

**Scheme 90:**
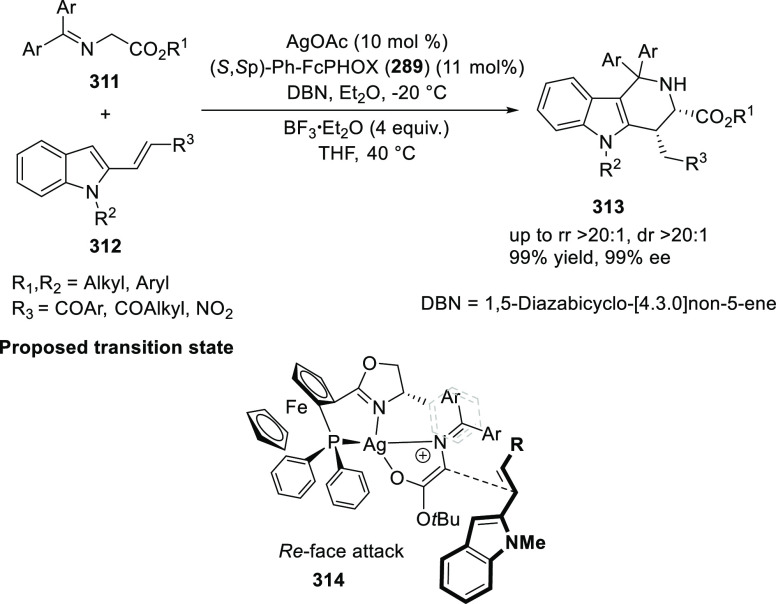
Regioselective and Stereoselective [3 + 3] Annulation of Ketone-Derived Azomethine Ylides with 2-Indolylethylenes

Following their earlier work, Deng described the first example of the Ag(I)/(*S*,*S*_p_)-Ph-FcPHOX (**289**)-catalyzed regioselective and stereoselective [3 + 3] annulation of ketone-derived azomethine ylides (**311**) with 2-indolylethylenes (**312**) (Scheme 90).^105^ This tandem method involved a Michael addition followed by a BF_3_·Et_2_O-promoted Friedel–Crafts reaction. Like their earlier report, the traditional [3 + 2] cycloaddition was prevented by using sterically hindered ketone-derived azomethine ylides (>20:1 rr). A wide variety of highly substituted tetrahydro-γ-carboline derivatives (**313**) were obtained in high yields (up to 99%) with excellent stereoselectivities (up to >20:1 dr, up to 99% *ee*). Remarkably, the stereochemistry for the [3 + 3] annulation of the ketone-derived azomethine ylides was different from that of aldehyde-derived azomethine ylides although the same ligand Ph-FcPHOX (**289**) was used. This was due to the favored *Re*-face attack of the nucleophilic ketone-derived azomethine ylide to 2-indolylethylenes because of the steric effect generated by the two bulky phenyl groups in the nucleophile.

**Scheme 91:**
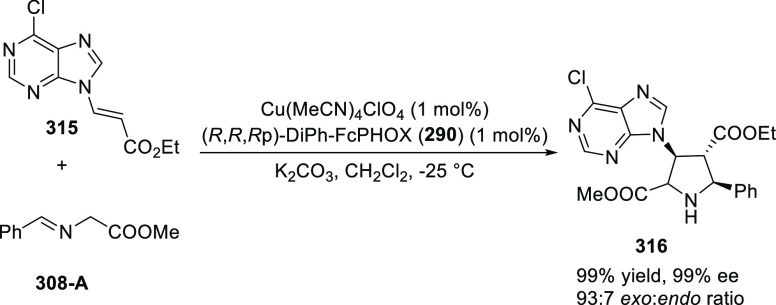
Highly *exo*- and Enantioselective 1,3-Dipolar Cycloaddition of Azomethine Ylides with β-Nucleobase Substituted Acrylates

In 2014, Guo reported a synthetic method to access chiral azacyclic nucleoside analogues (**316**) *via* a Cu/(*S*,*S*,*R*_p_)-diPh-FcPHOX (**290**)-catalyzed highly *exo*- and enantioselective 1,3-dipolar cycloaddition of azomethine ylides (**308-A**) with β-nucleobase substituted acrylates (**315**) (Scheme 91).^106^ A variety of azacyclic nucleoside analogues (**316**) were synthesized in high yields (up to 99%), excellent *exo*- (up to 93:7) and enantioselectivities (>99% *ee*). The use of dipolarophiles including pyrimidine-, benzimidazole-, imidazole-, benzotriazole-, and indole-substituted acrylates, afforded the desired pyrrolidine derivatives with excellent results. It is important to note that the (*E*)-geometry of β-nucleobase acrylate is essential since the reaction of the (*Z*)-isomer of β-nucleobase acrylate **315** furnished the expected products in low conversion along with poor enantioselectivity.

**Scheme 92:**
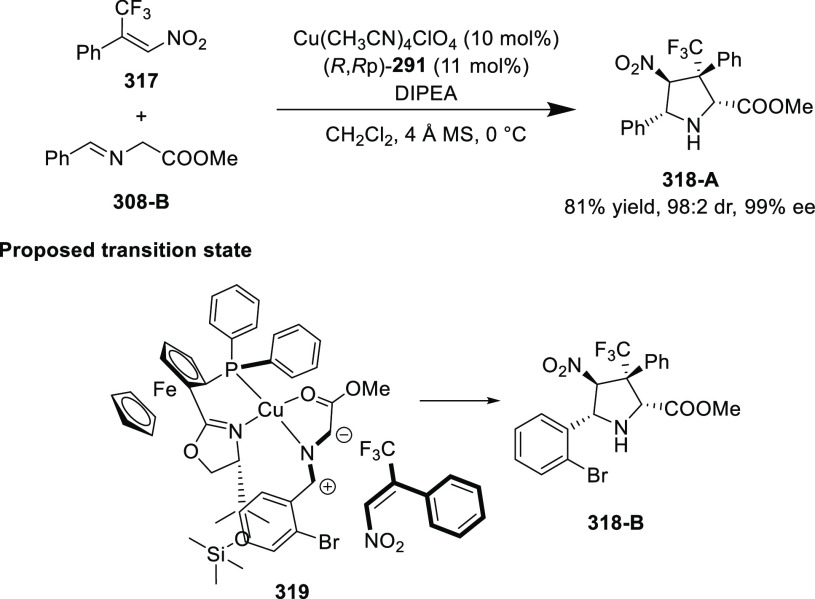
[3 + 2] Cycloaddition between Azomethine Ylides and β-Trifluoromethyl β,β-Disubstituted Nitroalkenes

In 2016, Zhou developed the first catalytic asymmetric synthesis of pyrrolidines (**318-A**) possessing a trifluoromethylated quaternary stereogenic center at the C-3 position of the pyrrolidine ring using the Cu/FcPHOX (**291**)-catalyzed 1,3-dipolar [3 + 2] cycloaddition between azomethine ylides (**308-B**) and β-trifluoromethyl β,β-disubstituted nitroalkenes (**317**) (Scheme 92).^107^ This *exo*-selective method provided different pyrrolidine derivatives (**318-A**) with high diastereoselectivities (up to >98:2 dr) and excellent enantioselectivities (up to >99.9 *ee*). Azomethine ylides bearing an alkyl-substituent and an *ortho*-hydroxy substituent on the aromatic ring failed to give any product. Additionally, azomethine ylides derived from alanine did not react under this catalytic protocol.

The proposed transition state **319** shows that the position of the substituents on the aromatic ring of azomethine ylides affects the result through their steric impact. This rationalizes the higher enantioselectivities observed for *ortho*-substituted azomethine ylides than the corresponding *para*- and *meta*-analogues whereas the substituents on the aromatic rings of β-trifluoromethyl nitroalkenes had a limited effect on the enantioselectivity of the reaction.

**Scheme 93:**
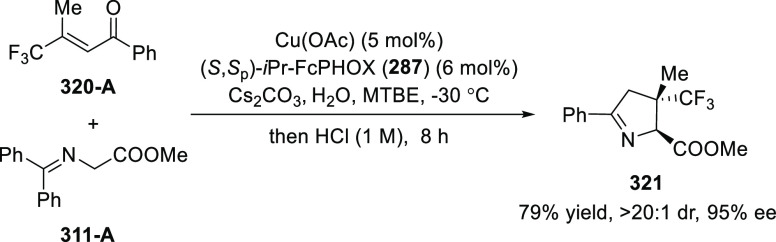
Asymmetric Michael Addition of Ketone-Derived Azomethine Ylide to β-Trifluoromethyl β,β-Disubstituted Enones

In 2017, Liu and Zhang reported a Cu/(*S*,*S*_p_)-*i*Pr-FcPHOX (**287**)-catalytic asymmetric Michael addition of ketone-derived azomethine ylides (**311-A**) to β-trifluoromethyl β,β-disubstituted enones (**320-A**) and subsequent hydrolytic cyclization to generate 1-pyrrolines (**321**) (Scheme 93).^108^ This method offers a facile entry to highly functionalized 1-pyrrolines (**321**) bearing two contiguous stereocenters in excellent stereoselectivities (up to >20:1 dr, 98% *ee*). Remarkably, the addition of trace amounts of water is essential for the chemoselective formation of 1-pyrrolines, rather than pyrrolidines (formed via direct 1,3-dipolar cycloaddition). The addition of 6 equiv of water changed the ratio between 1-pyrroline to pyrrolidine from 9:1 to >100:1. Similar levels of chemoselectivity were achieved when ethanol was used instead of water.

**Scheme 94:**
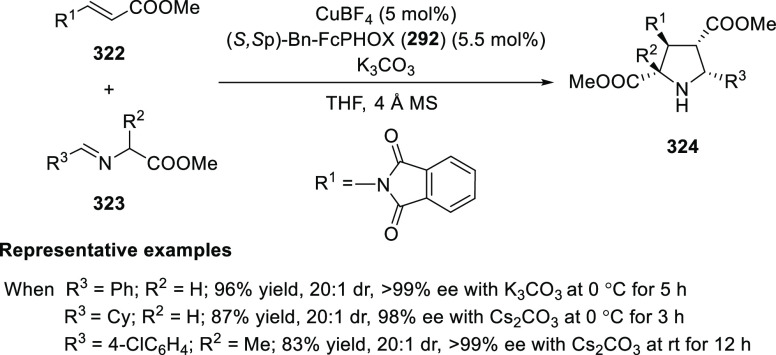
1,3-Dipolar Cycloaddition of Azomethine Ylides with β-Phthaliminoacrylate Esters

In 2017, Deng reported the first example of a Cu/(*S*,*S*)-Bn-FcPHOX (**292**)-catalyzed 1,3-dipolar cycloaddition of azomethine ylides (**323**) with β-phthaliminoacrylate esters (**322**) to generate pyrrolidine β-amino esters (**324**) in high yields with excellent diastereo- and enantioselectivities (up to 98%, > 20:1 dr, > 99% *ee*) (Scheme 94).^109^ It was noted that less reactive aliphatic-substituted azomethine ylides and azomethine ylides (**323**) derived from alanine were well tolerated in the reaction furnishing the corresponding pyrrolidines (**324**) in excellent stereoselectivities.

**Scheme 95:**
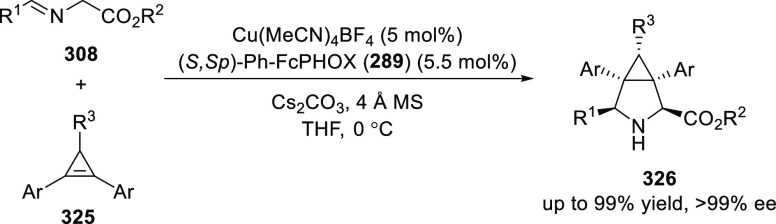
Stereoselective Synthesis of Chiral Bicyclic 3-Azabicyclo[3.1.0]hexanes

In 2018, Yang and Deng reported the first example of a highly efficient and stereoselective synthesis of chiral bicyclic 3-azabicyclo[3.1.0]hexanes (**326**) using a Cu/(*S*,*S*)-Ph-FcPHOX (**289**)-catalyzed asymmetric [3 + 2] cycloaddition of azomethine ylides (**308**) with trisubstituted cyclopropenes (**325**) (Scheme 95).^110^ With this new desymmetrization process, the asymmetric construction of 3-azabicyclo[3.1.0]hexane derivatives (**326**) possessing five contiguous stereogenic centers and two bridgehead quaternary stereogenic centers was achieved in high yields (up to 99%) and enantioselectivities (up to 99% *ee*). A variety of functionalities (CO_2_R, CN, CONMe_2_) on the cyclopropane ring and aliphatic-substituted azomethine ylides were well tolerated.

**Scheme 96:**
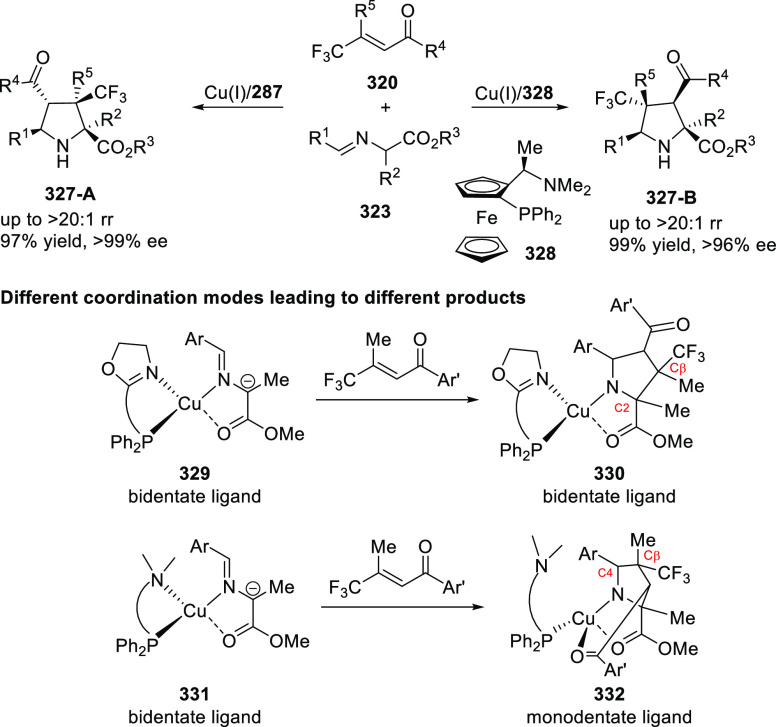
Asymmetric Intermolecular [3 + 2] Cycloaddition of α-Substituted Iminoesters

In 2018, Liu and Zhang reported an unprecedented ligand-controlled regiodivergent Cu(I)/chiral *P*,*N*-ligand (**287/328**)-catalyzed asymmetric intermolecular [3 + 2] cycloaddition of α-substituted iminoesters (**323**) with β-fluoromethyl β,β-disubstituted enones (**320**) (Scheme 96).^111^ This novel method allowed for the enantioselective regiodivergent synthesis of pyrrolidines (**327 A-B**) bearing two adjacent quaternary stereocenters or two discrete quaternary stereocenters, in high yields (up to >99%), and high regio- (up to >20:1 rr), diastereo- (up to >20:1 dr), and enantioselectivity (up to >99% *ee*). Mechanistic studies provided insights into the ligand controlled origins of the regioselective control of the cycloaddition. The phosphorus and nitrogen atoms of (*S*,*S*)-*i*Pr-FcPHOX (**287**), remain coordinated to the Cu(I) throughout the whole catalytic process, and **327-A** was obtained as the main product due to a combination of the electron distribution across the complex, steric hindrance effects, and a π–π interaction between the two aryl rings of the iminoester **323** and enone **320**. In contrast ligand **328** acts as a pseudobidentate ligand. The formation of an O–Cu bond with the carbonyl oxygen atom of the enone **320** and dissociation of the amine from the Cu(I) center occurs which makes ligand **328** monodentate and results in switching the regioselectivity of the reaction to form product **327-B**.

**Scheme 97:**
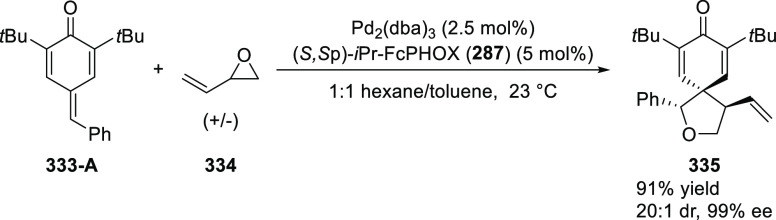
Formal [3 + 2] Cycloaddition of *p*-Quinone Methides

In 2016, Zhao developed an unprecedented formal [3 + 2] cycloaddition of *p*-quinone methides (**333-A**) with vinyl epoxides (**334**) under Pd/(*S*,*S*_p_)-*i*Pr-FcPHOX (**287**) catalysis (Scheme 97).^112^ A wide range of spiro[4.5]decanes (**335**) were obtained in high efficiency and stereoselectivity. It is noteworthy that the bulky *t*-butyl substituents on the *para*-quinone methide structure were important for the high diastereoselectivity and enantioselectivity of this catalytic transformation. Changing the *t*-butyl to the smaller isopropyl or methyl groups led to a drastically reduced dr and *ee* of the reaction (up to 5:1 dr, up to 30% *ee*). Importantly, a variety of aryl and alkyl-substituted *p*-quinone methides were tolerated as substrates.

**Scheme 98:**
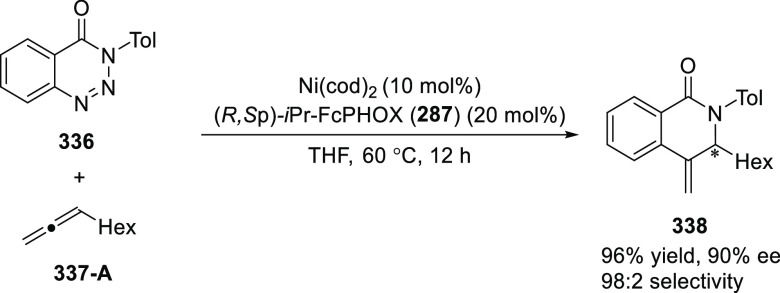
Denitrogenative Annulation of 1,2,3-Benzotriazin-4(3*H*)-ones with Allenes

In 2010, Murakami reported a Ni/(*S*,*Sp*)-*i*Pr-FcPHOX (**287**)-catalyzed denitrogenative annulation of 1,2,3-benzotriazin-4(3*H*)-ones (**336**) with allenes (**337-A**) (Scheme 98). A variety of substituted 3,4-dihydroisoquinolin-1(2*H*)-ones (**338**), found in a wide variety of plant alkaloids and bioactive compounds, were synthesized in high regio- and enantioselectivity (up to 99% yield and 97% *ee*).^113^ This process tolerates a variety of functionalities and worked well with sterically and electronically different *N*-aryl substituents. The regioselectivity was significantly affected by the sterics of the allene substituent as changing the hexyl group to a cyclohexyl group led to a drop in regioselectivity from 98:2 to 73:27, whereas the enantioselectivity remained unaltered.

**Scheme 99:**
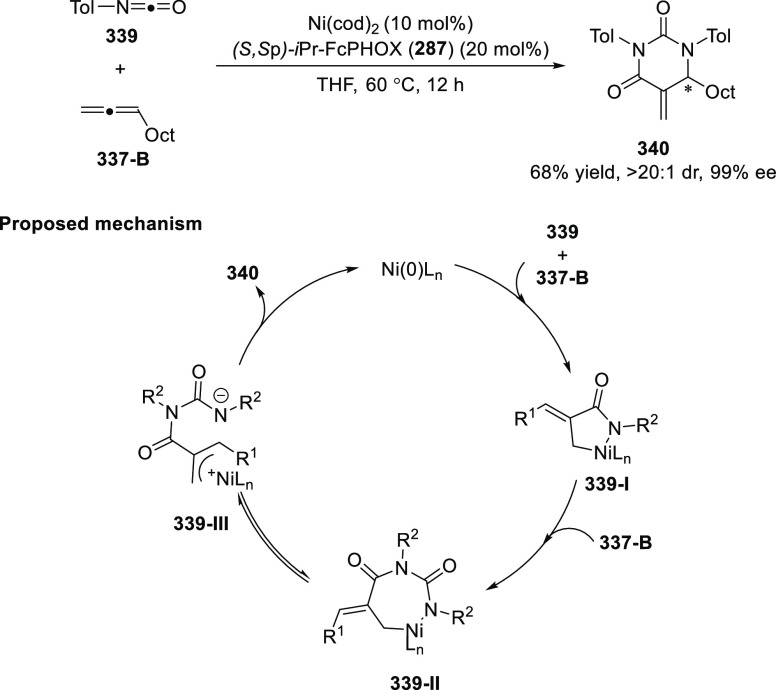
Enantioselective Cycloaddition between Isocyanate and Allene

In 2010, Murakami developed a Ni/(*S*,*Sp*)-*i*Pr-FcPHOX (**287**)-catalyzed enantioselective cycloaddition between isocyanate (**339**) and allene (**337-B**) (Scheme 99).^114^ This process involves the [2 + 2 + 2] cycloaddition of two molecules of isocyanate and one molecule of allene, providing an efficient access to enantiomerically enriched dihydropyrimidine-2,4-diones (**340**). This method is limited to aryl isocyanates since various alkyl isocyanates failed to undergo this cycloaddition. Surprisingly, electron-rich aryl isocyanates furnished products with higher regioselectivity compared to electron-deficient aryl isocyanates. A mechanistic cycle involves intermolecular Ni(0) oxidative cyclization between the allene (**337-B**) and isocyanate (**339**), followed by the addition of one more molecule of isocyanate to generate a zwitterionic π-allylnickel species (**339-III**) which, upon cyclization at the more substituted carbon of the allyl moiety, affords product **340** along with Ni(0).

**Scheme 100:**
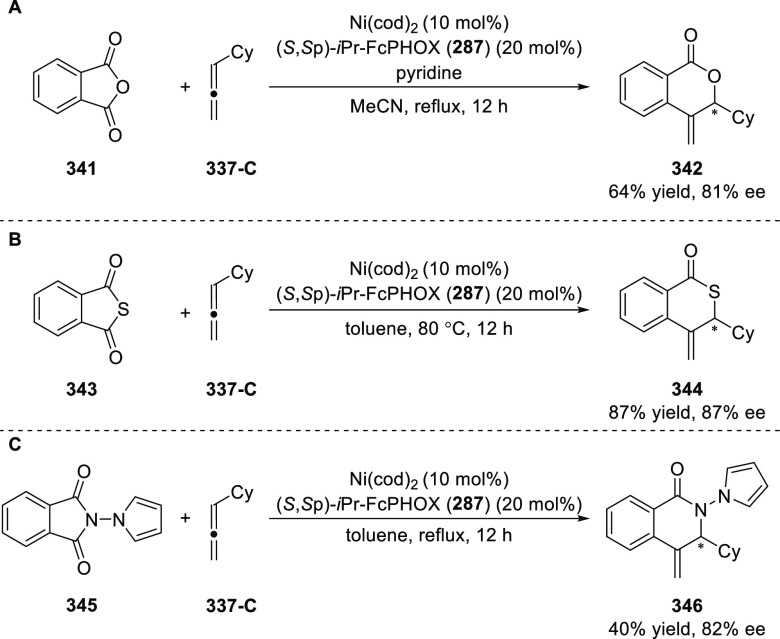
Decarbonylative Cycloaddition of Phthalic Anhydride with Allenes

In 2011, Matsubara developed a decarbonylative cycloaddition of phthalic anhydride (**341**) with allenes (**337-C**) to give δ-lactone derivatives (**342**) (Scheme 100A).^115^ The reaction proceeds via asymmetric insertion of a carbon–carbon double bond into a carbon–oxygen bond. The use of (*S*,*S*_p_)-*i*Pr-FcPHOX (**287**) afforded chiral δ-lactone derivatives (**342**) in moderate yields and enantioselectivities of up to 81%. This decarbonylation was also extended to thiophthalic anhydride (**343**) and *N*-pyrrole-substituted phthalimide (**345**), which furnished the corresponding chiral cycloadducts (**344/346**) in high yield and enantioselectivities of up to 87% and 82% *ee*, respectively (Scheme 100B,C).

**Scheme 101:**
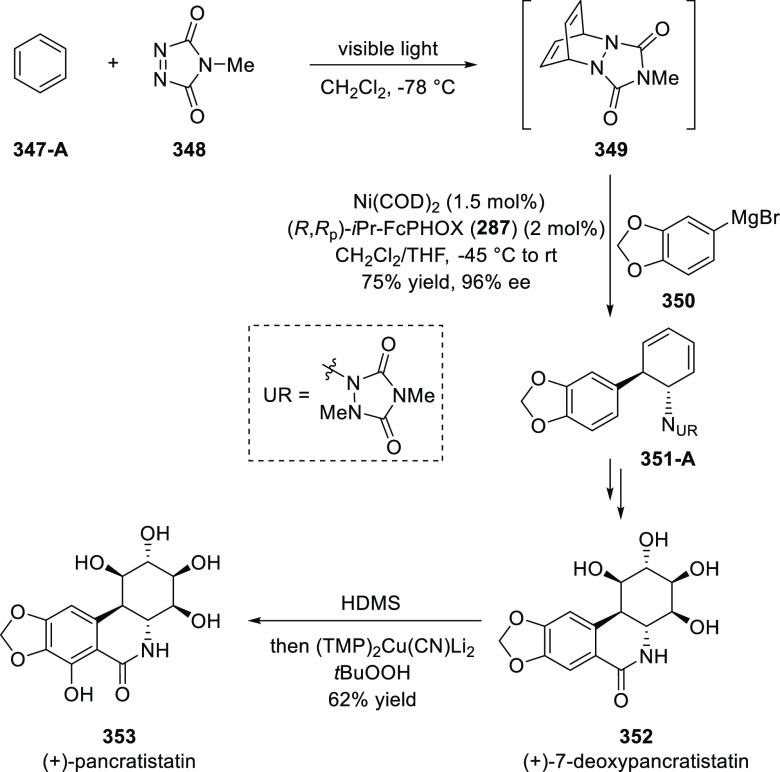
Synthesis of (+)-Pancratistatin and (+)-7-Deoxypancratistatin

In 2017, Sarlah reported a concise synthesis of (+)-pancratistatin (**353**) and (+)-7-deoxypancratistatin (**352**) from benzene (**347-A**) using a dearomative 1,2-transcarboamination approach in six and seven steps in 19% and 12% overall yields, respectively (Scheme 101).^116^ The key step was to install the first two vicinal stereocenters which involved visible light-promoted *para*-cycloaddition of the benzene with the N–N arenophile, *N*-methyl-1,2,4-triazoline-3,5-dione (MTAD, **348)**, to generate the MTAD-benzene cycloadduct **349**. The cycloadduct **349** was further reacted with aryl Grignard reagent **350** using a catalytic amount of Ni/(*R*,*R*p)-*i*Pr-FcPHOX (**287**) to generate the desired dearomatized product **351-A** in 75% yield as a single diastereoisomer (*trans*) with high enantioselectivity (96% *ee*) on a decagram scale. The diene intermediate **351-A** was further used to complete the synthesis of the (+)-pancratistatin core.

The cycloadduct **349** comprises bis-allylic bridgehead positions bearing an electron-deficient urazole and hence it is prone for oxidative addition. Mechanistically, the catalytic process involves anti-π-coordination of the diene to the metal complex, oxidative addition, transmetalation to generate a symmetric η^5^-complex which, upon enantiodiscrimination of the enantiotopic termini of the cyclohexadienyl system, yields the product enantioselectively.

**Scheme 102:**
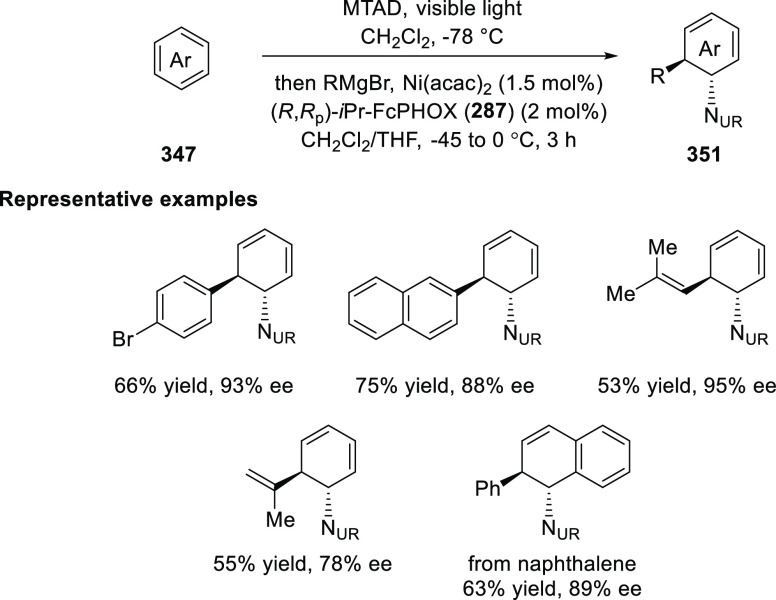
Dearomative *trans*-1,2-Carboamination

In 2018, Sarlah applied their novel dearomative *trans*-1,2-carboamination strategy to a range of aromatic precursors (**347**) including naphthalene and a series of Grignard reagents using an air-stable Ni(acac)_2_ and (*R*,*R*_p_)-*i*Pr-FcPHOX (**287**) to obtain diene products (**351**) with exclusive 1,2-*trans* selectivity, and high enantioselectivity (Scheme 102).^117^ Interestingly, the methodology was also feasible with different vinyl Grignard reagents which allowed for the enantioselective installation of an alkene moiety.

**Scheme 103:**
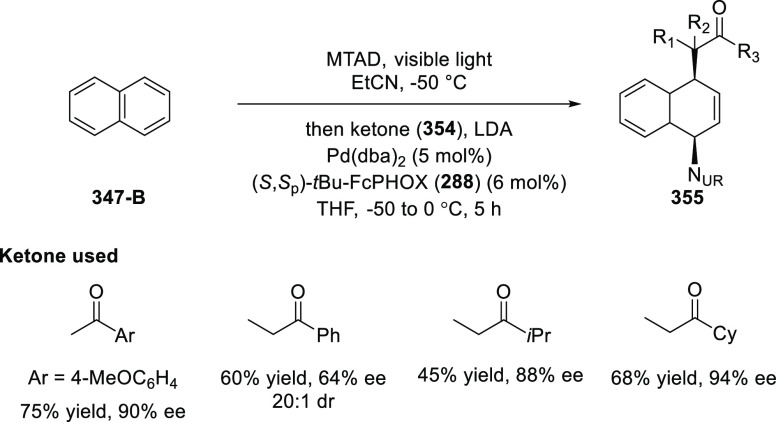
Enantioselective Ring-Opening Reaction

In subsequent studies, Sarlah developed a Pd/(*S*,*S*_p_)-*t*Bu-FcPHOX (**288**)-catalyzed enantioselective ring-opening reaction of the cycloadduct derived from naphthalene (**347-B**) and MTAD using lithium enolates derived from ketone (**354**) (Scheme 103).^118^ The method is highly step- and atom-economical and delivers products with exclusive *syn*-1,4-selectivity and high enantioselectivity (up to 94% *ee*). Compared to the Ni-catalyzed process, the present protocol is limited to specific substrates. Additionally, enantioselective ring-opening with ester-derived lithium enolates was also achieved by using a [Pd(allyl)Cl]_2_ and (*R*)-DTBM-SEGPHOS combination.

**Scheme 104:**
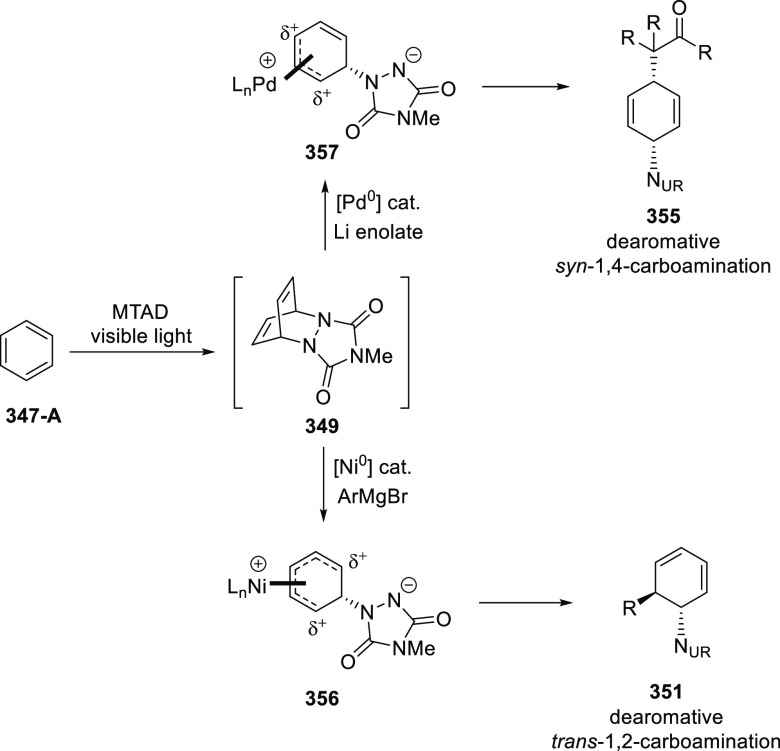
Mechanistic Details

The complementary selectivity observed in Pd and Ni catalysis is likely due to the result of the inner-sphere delivery of the Grignard reagent in the case of cationic Ni η^5^-complex **356** and outer sphere attack of the enolate on Pd η^3^-intermediate **357** (Scheme 104).

##### 2.1.3.2 FcPHOX Ligands for Asymmetric 1,4- and 1,2-Addition

**Scheme 105:**
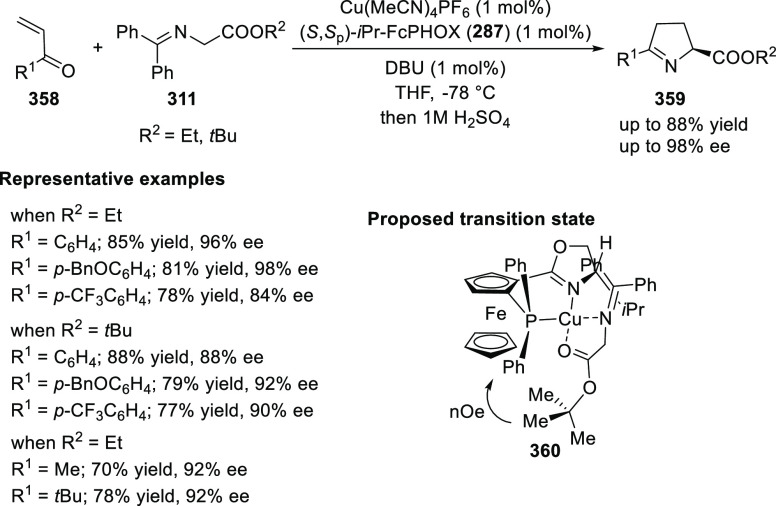
Enantioselective Reaction between Glycine Derivatives and α,β-Unsaturated Ketones

In 2011, Zajac reported an enantioselective reaction between glycine derivatives (**311**) and α,β-unsaturated ketones (**358**) using a Cu complex of (*S*,*S*_p_)-*i*Pr-FcPHOX (**287**) ligand (Scheme 105).^119^ The chiral Michael adducts (**359**) formed were directly transformed into the cyclized product upon acid/base workup. The use of acrylonitrile, methyl acrylate or phenylvinyl sulfone as Michael acceptors afforded cycloaddition products exclusively instead of the expected chiral Michael addition adducts. The mechanistic studies performed showed that structure **360** retains a distorted tetrahedral geometry around Cu. It is believed that the phenyl groups of the diphenylphosphine and the imine in **311** occupy a coplanar conformation due to possible π-stacking, with the ester *t*-butyl group underneath the complex. This orientation requires the electrophilic, α,β-unsaturated ketone (**358**) to approach the nucleophile from the *Si*-face and eclipse the phenyl groups.

**Scheme 106:**
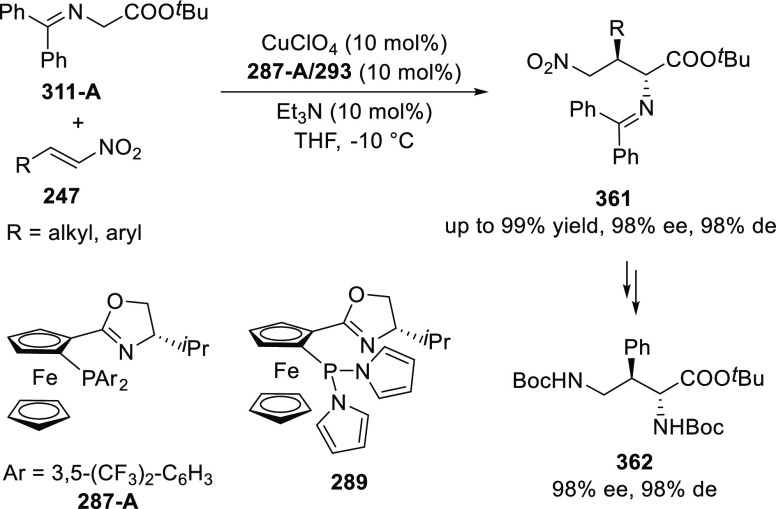
Asymmetric Michael Addition of Glycine Derivatives to Nitroalkenes

In 2010, Hou reported a Cu-catalyzed asymmetric Michael addition of glycine derivatives (**311-A**) to nitroalkenes (**247**) using a derivative of the (*S*,*S*_p_)-*i*Pr-FcPHOX (**287-A**) ligand (Scheme 106).^120^ This method provided a route to prepare a variety of β-substituted-α,γ-diaminobutyric acid derivatives (**361**) in high diastereo- and enantioselectivities (up to 98% de, 98% *ee*). The *ortho*-substituted nitrostyrene derivatives provided much lower enantioselectivities (76 to 89% *ee*) with the (*S*,*S*_p_)-*i*Pr-FcPHOX ligand. FcPHOX **289** with a P-bound pyrrole group possessing strong π-acceptor and weak σ-donor properties dramatically improved the enantioselectivity (up to 93% *ee*) for this class of substrates.

**Scheme 107:**
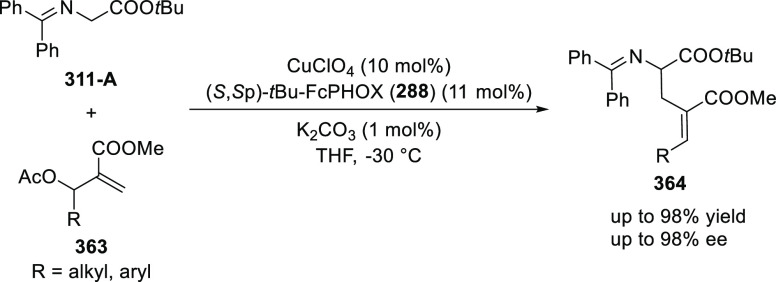
Enantioselective Synthesis of 4-Alkylidenylglutamic Acid Derivatives

In 2009, Pu reported a Cu/(*S*,*S*_p_)-*t*Bu-FcPHOX (**288**)-catalyzed enantioselective synthesis of 4-alkylidenylglutamic acid derivatives (**364**) from the reaction of a glycinate Schiff base (**311-A**) with activated alkyl- and aryl-substituted allylic acetates (**363**) (Scheme 107).^121^ The presence of electron-withdrawing groups was essential, as no product was formed when glycine *t*-butyl ester (**311-A**) reacted with simple allyl acetate. This suggested that the reaction proceeds *via* a tandem conjugate addition–elimination pathway.

**Scheme 108:**
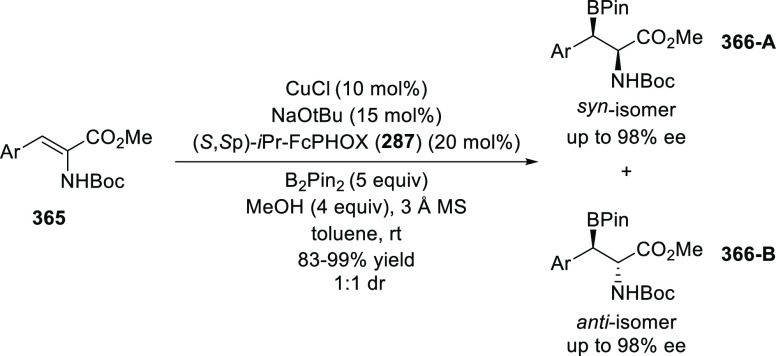
Asymmetric Borylation of β-Substituted α-Dehydroamino Acid Derivatives

In 2014, Lin reported the Cu/(*S*,*S*p)-*i*Pr-FcPHOX (**287**)-catalyzed asymmetric borylation of β-substituted α-dehydroamino acid derivatives (**365**) using B_2_Pin_2_ to furnish enantioenriched *syn*- and *anti*-β-boronate-α-amino acid derivatives (**366**) in a 1:1 ratio (Scheme 108).^122^ The double bond geometry in the β-phenyl-substituted substrate was very important since the use of the (*E*)-isomer did not afford any product under these reaction conditions. Changing an aryl group to an alkyl group in the starting alkene afforded the product in high yield but with poor enantioselectivity (<30%) for both regioisomers.

**Scheme 109:**
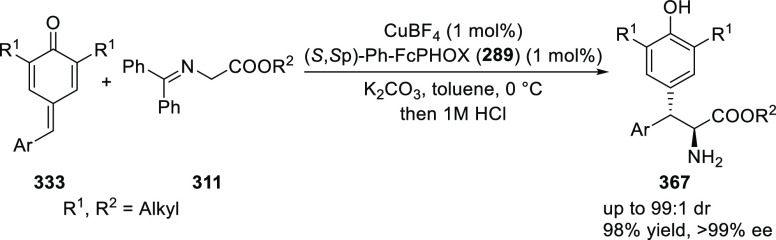
Enantioselective 1,6-Conjugate Addition of Glycine Schiff Bases

In 2016, Deng described a Cu/(*S*,*S*_p_)-Ph-FcPHOX (**289**)-catalyzed highly enantioselective catalytic 1,6-conjugate addition of glycine Schiff bases (**311**) to *p*-quinone methides (**333**) (Scheme 109).^123^ A series of *p*-quinone methides (**333**) derived from aryl aldehydes reacted with glycine Schiff bases to provide nonracemic β-Ar,Ar′-α-amino acid derivatives (**367**) with two contiguous stereogenic centers in high yields with excellent diastereoselectivities (up to 99:1) and enantioselectivities (up to >99% *ee*).

**Scheme 110:**
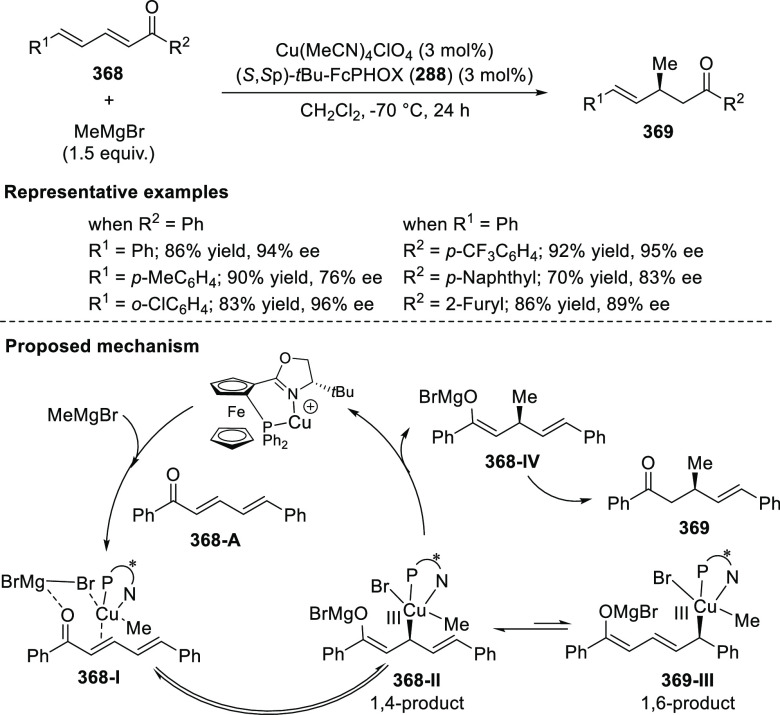
Regio- and Enantioselective 1,4-Conjugate Addition Reaction of Alkyl Grignard Reagents

In 2013, Zhang developed a Cu/(S,S_p_)-*t*Bu-FcPHOX (**288**)-catalyzed regio- and enantioselective 1,4-conjugate addition reaction of alkyl Grignard reagents to α,β,γ,δ-unsaturated ketones (**368**) (Scheme 110).^124^ A series of γ,δ -unsaturated ketones (**369**) were obtained in high yields with high enantioselectivity (up to 92% yield and 96% *ee*). The increase in the steric hindrance of the Grignard reagent had a negative impact on enantioselectivity as well as on reaction yield. The mechanism is proposed to involve the formation of π-complex **368-I** between Cu–ligand, MeMgBr and substrate **368-A**. Later, Cu(III) σ-complex **368-II** forms via oxidative addition, which is stabilized by the diconjugated system. This stability accounts for the preference of the 1,4-selectivity over 1,6-selectivity. Finally, the reductive elimination of **368-II** delivers Cu-FcPHOX and the 1,4-addition product **369**.

**Scheme 111:**
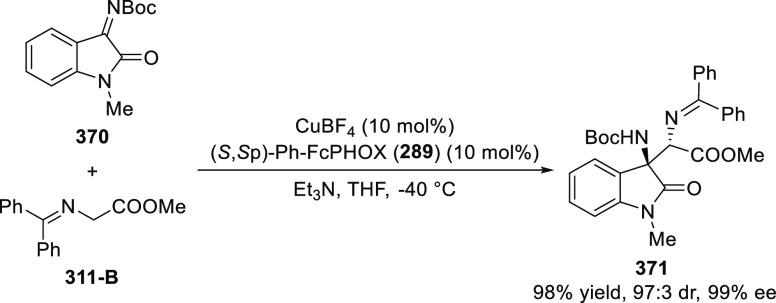
Diastereo- and Enantioselective Mannich Reaction of Glycine Schiff Bases

In 2018, Deng developed the Cu(I)/(*S*,*S*_p_)-Ph-FcPHOX (**289**)-catalyzed diastereo- and enantioselective Mannich reaction of glycine Schiff bases (**311-B**) with isatin *N*-Boc ketimines (**370**) (Scheme 111).^125^ A wide range of 3-substituted 3-aminooxindole compounds (**371**) were obtained in high yields (up to 98%) with excellent enantioselectivities (>99% *ee* in most cases) and moderate to high diastereoselectivities (up to 97:3 dr). The reactivities and stereoselectivities of the Mannich reaction were greatly reliant on the steric hindrance of the ester groups employed. The methyl and ethyl glycine esters were most suitable, whereas the diastereoselectivities decreased when isopropyl or benzyl esters were tested. The *t*-butyl glycine ester Schiff base was found to be unreactive. The *N*-protection of isatin is crucial as no reaction occurred with isatin possessing a free NH. The imine derived from *N*-methyl isatin was found to be the best substrate whereas changing to either an *N*-benzyl or an *N*-acetyl isatin produced disappointing diastereoselectivities.

##### 2.1.3.3 FcPHOX Ligands for Asymmetric Allylic Alkylation

**Scheme 112:**
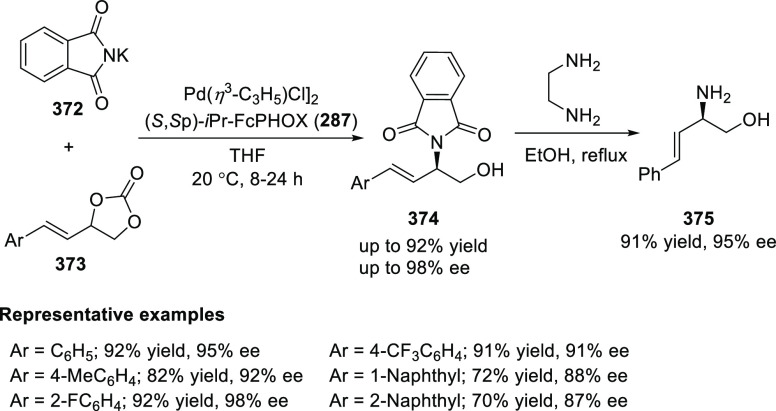
Asymmetric Allylic Amination of 4-Aryl-1,3-dioxolan-2-ones

In 2013, Zhang reported a Pd/(*S*,*S*_p_)-*i*Pr-FcPHOX (**287**)-catalyzed asymmetric allylic amination of 4-aryl-1,3-dioxolan-2-one (**373**) to synthesize chiral β-aryl-α,β-unsaturated amino alcohols (**375**) (Scheme 112).^126^ A wide variety of chiral amino alcohols (**374**) were obtained in good to excellent yields (up to 92%) and enantioselectivities (up to 98% *ee*) under mild reaction conditions.

**Scheme 113:**
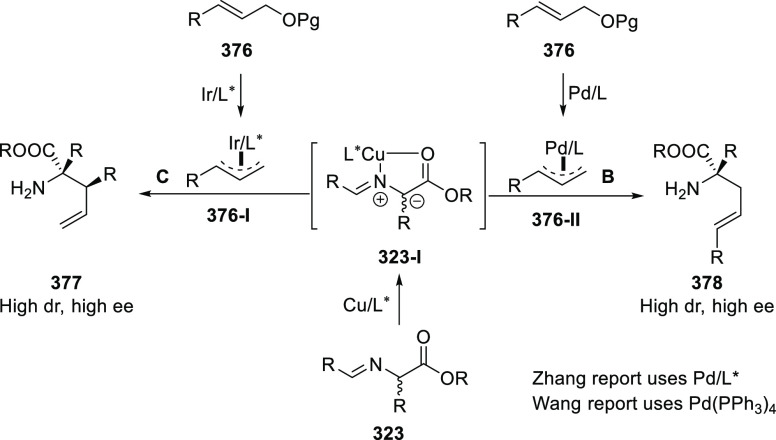
Asymmetric α-Allylation of Schiff Base Activated Amino Acids

In 2017 and 2018, Zhang and Wang independently published a series of papers based on a Pd/Cu dual catalyst system for the asymmetric α-allylation of Schiff base activated amino acids (Scheme 113).^127−133^

**Scheme 114:**
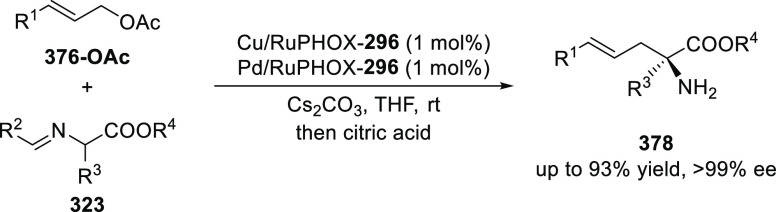
Cu/Pd-Catalyzed Asymmetric α-Allylation of Schiff Base

Zhang reported a Pd/Cu cooperative catalytic system for the asymmetric α-allylation of Schiff bases derived from imine esters (**323**) (Scheme 114).^127^ A dimeric *t*Bu-RuPHOX (**296**) was found to be a common ligand of choice. A wide variety of α,α-dialkyl α-amino acid derivatives (**378**) were synthesized in high yields and with excellent enantioselectivities (up to >99% *ee*). The method was easily extended to the asymmetric allylation of a range of small peptides. The catalytic system utilizes chiral complexes of Cu and Pd with the chiral ligand, *t*Bu-RuPHOX (**296**), and the use of the same ligand on both metals avoids the possible problem of ligand exchange and also makes it practically simple. The catalytic cycle involves the simultaneous generation of a five-membered *N*,*O*-bidentate metalated nucleophilic species **323-A** between Cu and the Schiff base and the electrophilic π-allyl Pd intermediate **376-II**. This intermediate further reacts to give allylated product in high selectivity. Both studies results suggested that the rigid structure of the five-membered *N*,*O*-bidentate metalated azomethine ylide **323-A** enables the asymmetric induction from the chiral ligand (Scheme 113).

**Scheme 115:**
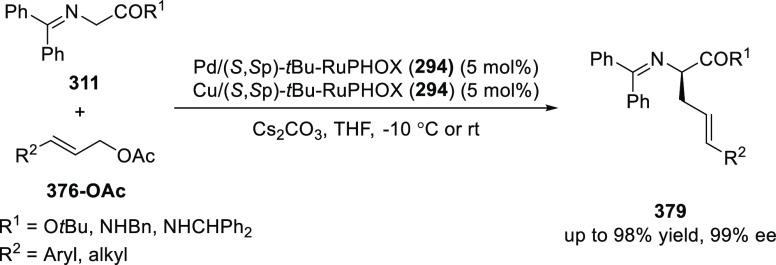
Cu/Pd-Catalyzed Asymmetric α-Allylation of Schiff Bases

Zhang found that the aldehyde-derived glycine esters (**323**) always furnished bisallylated products. This limitation was solved in the subsequent studies involving asymmetric allylation of ketone-derived prochiral glycine ester and amide derivatives (**311**) using the catalytic Cu/Pd cooperative system (Scheme 115).^128^ This time ligand (*S*,*S*_p_)-*t*Bu-RuPHOX (**294**) performed better compared to the earlier investigated dimeric ligand *t*Bu-RuPHOX (**296**). A range of α-substituted α-amino acid derivatives (**379**) were efficiently synthesized in high yields and with excellent enantioselectivities under mild conditions (up to 98% yield and 99% *ee*).

**Scheme 116:**
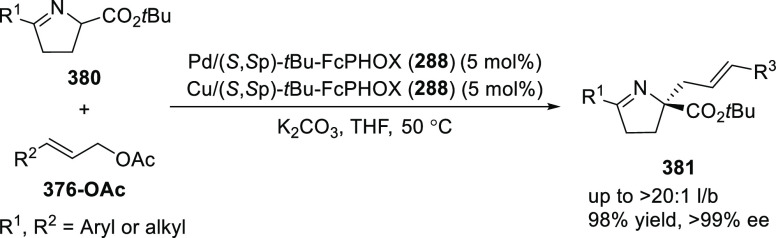
Asymmetric Allylation to 1-Pyrroline-5-carboxylic Esters

Later, Zhang expanded the scope of this asymmetric allylation to 1-pyrroline-5-carboxylic esters, cyclic imino esters (**380**) to generate a variety of 3,4–2*H*-pyrrole (**381**) derivatives bearing a quaternary stereogenic center in high yields and excellent enantioselectivities (up to 98% yield and >99% *ee*) (Scheme 116).^129^ Similar to earlier studies, mechanistic investigations uncovered that the synergistic action of the two chiral metal complexes is responsible for its high reactivity and excellent enantioselectivity; the steric hindrance and electronic factors of the electrophiles and the nucleophiles are crucial for the formation of the linear products.

**Scheme 117:**
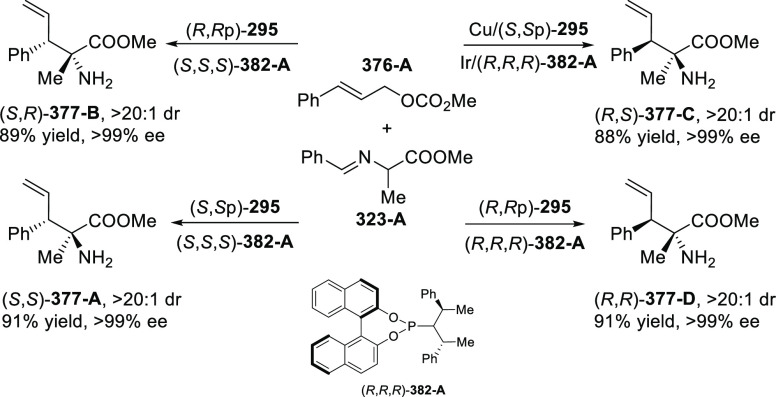
Ir/Cu-Catalyzed Allylic Alkylation of Imine Esters

In a recent report, Zhang introduced a synergistic Ir/Cu-catalyzed allylic alkylation of imine esters to construct a range of α,α-disubstituted α-amino acids (**377-A–D**) bearing two vicinal stereocenters with excellent levels of enantio- and diastereoselectivity (up to >99% *ee* and >20:1 dr) (Scheme 117).^130^ The (*S*,*S*_p_)-*i*Pr-RuPHOX (**295**) ligand on Cu and Feringa’s chiral phosphoramidite ligands (**382-A**) on Ir were necessary for the success of the reaction. Significantly, the two chiral catalysts allowed complete control over the absolute and relative configuration, affording all stereoisomers of the desired products. This methodology was applied to synthesize dipeptides and analogues of bioactive molecules bearing vicinal stereocenters in a stereodivergent manner. The mechanism is proposed to involved the generation of a five-membered *N*,*O*-bidentate metalated nucleophilic species (**323-I**) using the Cu/*i*Pr-RuPHOX (**295**) complex followed by interception of the *in situ* formed reactive allyl Ir intermediate (**376-I**) to generate enantiopure α,α-disubstituted α-amino acid derivatives (**377**) bearing vicinal stereocenters (Scheme 113). It is important to note that the well-defined geometry of intermediates **323-I** and **376-I** allows for the control of the configurations of both stereocenters.

**Scheme 118:**
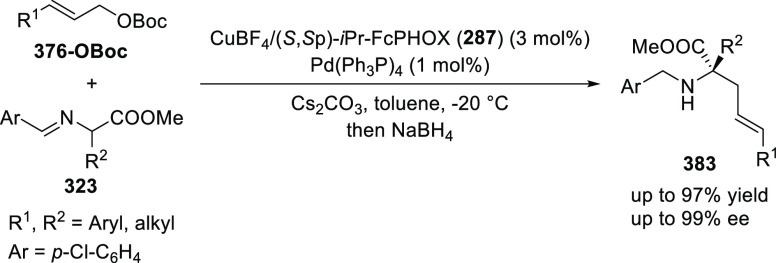
Pd/Cu-Catalyzed Allylic Alkylation of Imine Esters

At the same time, Wang independently reported a Pd/Cu dual catalyst system for the asymmetric α-allylation of Schiff bases derived from imine esters (**323**) to synthesize a variety of α,α-dialkyl α-amino acid derivatives (**383**) in high yields and with excellent enantioselectivities (up to 97% yield, > 99% *ee*) (Scheme 118).^131^ Similar to Zhang’s report, the presence of Cu and Pd is indispensable to this reaction as no product was observed in the absence of either. Wang’s catalytic system involves Cu(I) and chiral ligand **287** which *in situ* generates the chiral complex (**323-A**) from the Schiff base of imine ester (Scheme 116). Unlike Zhang’s report, the achiral ligand Ph_3_P was used with Pd to generate the achiral π-allylpalladium intermediate **376-II** (Scheme 113). Besides this, the ^31^P NMR spectroscopic analysis confirmed that either negligible or no ligand scrambling was observed from Cu to Pd. This observation confirmed that the rigid structure of the five-membered *N*,*O*-bidentate chiral Cu complex **323-A** controls the stereoselectivity of the allylation.

**Scheme 119:**
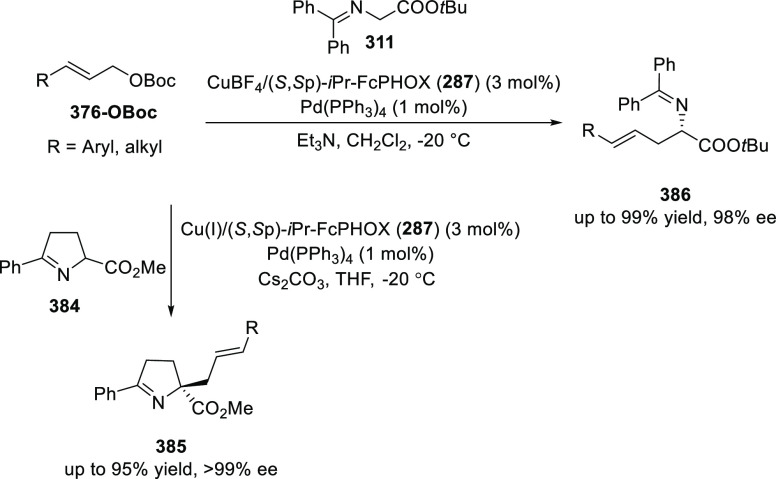
Stereoselective α-Allylation of Acyclic and Cyclic Ketimine Esters

Wang further employed this synergistic Cu/Pd catalytic system for the stereoselective α-allylation of both acyclic (**311-A**) and cyclic ketimine esters (**384**) (Scheme 119).^132^ The combination of the Cu/(*S*,*S*_p_)-*i*Pr-FcPHOX (**287**) complex and an achiral palladium complex delivered an array of biologically important α-allyl α-amino acid derivatives (**386**) and 2*H*-pyrrole derivatives (**385**) in excellent yields and enantioselectivities (up to 99% yield, >99% *ee*).

**Scheme 120:**
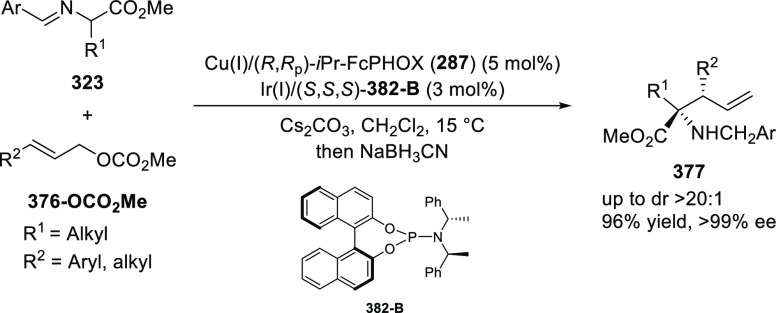
Stereodivergent Synergistic Cu/Ir Catalyzed α-Allylation of Aldimine Esters

Like Zhang, Wang established a stereodivergent synergistic Cu/Ir catalyzed α-allylation of aldimine esters to generate a series of α-amino acid derivatives (**377**) possessing vicinal stereocenters in high yield and excellent stereoselectivity (Scheme 120).^133^ For the success of reaction, the use of (*S*,*S*_p_)-*i*Pr-FcPHOX (**287**) ligand on Cu and Feringa’s chiral phosphoramidite ligands **382-B** on Ir was necessary. All four stereoisomers of **377** were accessed in good yield (up to 96%) with excellent enantioselectivity (>99% *ee*) and exclusive diastereoselectivity (>20:1 dr) from the same set of starting materials with the current dual Cu/Ir catalysis by simply selecting a pairwise combination of two chiral catalysts. This outcome suggested that the two distinct metal catalysts exert almost absolute control over the corresponding stereogenic centers, respectively. Additionally, the dual catalysts can be prepared *in situ* from a mixture of Cu(MeCN)_4_BF_4_, [Ir(cod)Cl]_2_, (*S*,*S*_p_)-*i*Pr-FcPHOX (**287**), and (*R*,*R*,*R*)-**382-B** in a one-pot protocol to furnish the expected allylated products (**377**) in comparable levels of yield and stereoselectivity.

##### 2.1.3.4 FcPHOX Ligands for Asymmetric Intramolecular Arylcyanation/Diarylation of Alkenes

**Scheme 121:**
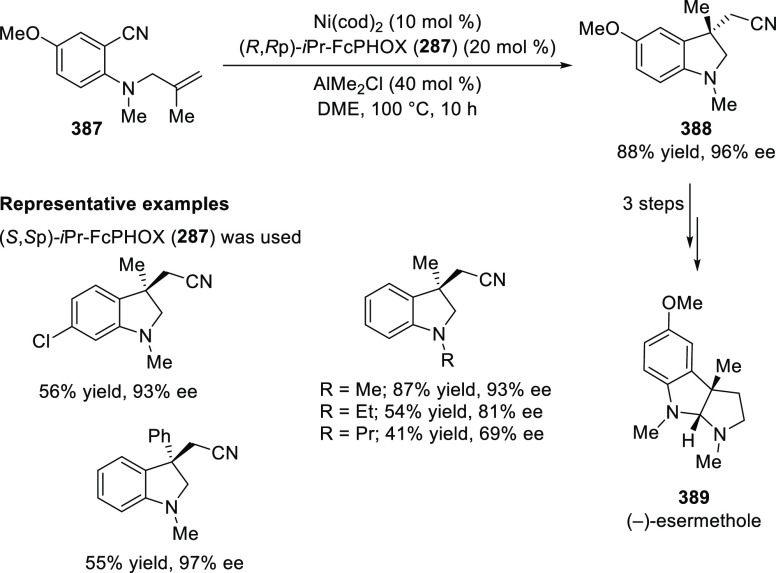
Asymmetric Intramolecular Arylcyanation of Alkenes

In 2008, Nakao described an intramolecular arylcyanation of alkenes to synthesize a range of synthetically interesting nitriles possessing a benzylic quaternary carbon stereocenter (Scheme 121).,^134^ The combination of Ni(cod)_2_ along with the chiral ligand, (*R*,*R*)-*i*Pr-FcPHOX (**287**) and the Lewis acid, AlMe_2_Cl, efficiently formed product **388** in 96% *ee* and 88% yield, which is a key intermediate in the subsequent synthesis of (−)-esermethole (**389**). Later in 2010, the scope of the methodology was expanded by synthesizing a variety of enantiomerically enriched 3,3-disubstituted indoline derivatives.^135^ It was observed that the electron density on the benzene ring slightly affected the enantioselectivity, possibly through altering the ability of the nitrogen or olefin to coordinate to Ni. Surprisingly, aryl–halogen bonds were tolerated whereas activation of C–CN bonds was achieved exclusively. Additionally, the size of the *N*-substituents significantly affected both the chemical yield and enantioselectivity as the bulkier substituents on the nitrogen slow down oxidative addition of the Ar–CN bond as well as coordination of the double bond to Ni. Mechanistic studies revealed that the AlMe_2_Cl promoted η^2^-nitrile complex formation by coordinating with the cyano nitrogen.

**Scheme 122:**
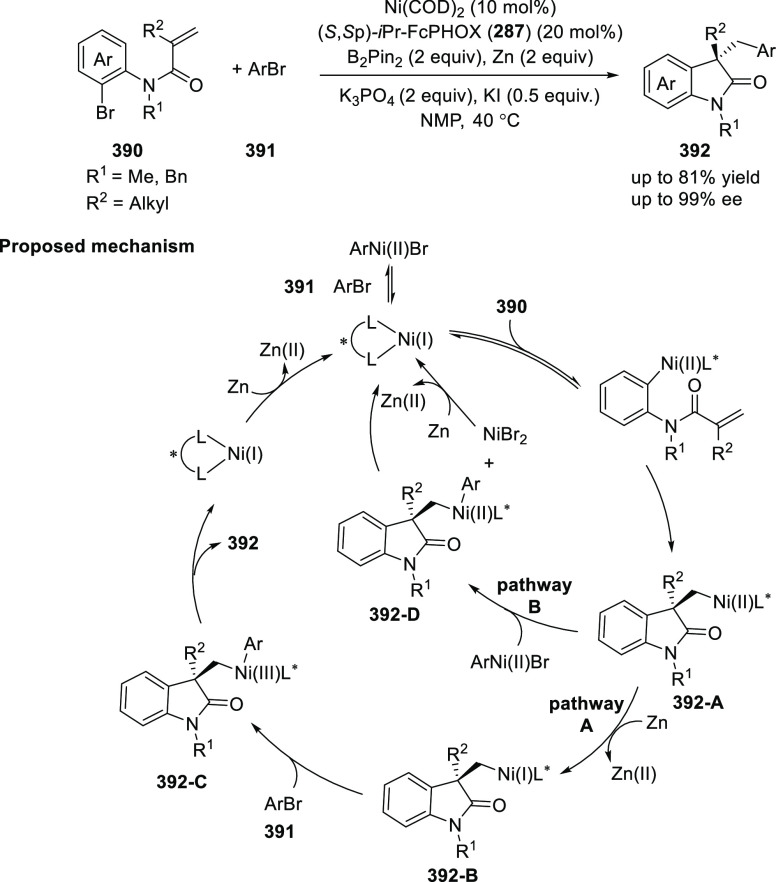
Enantioselective Diarylation of Activated Alkenes

An enantioselective diarylation of activated alkenes was reported by Kong wherein structurally distinguishable aryl bromides react together *via* a domino Heck cyclization/reductive cross coupling process using a catalytic amount of Ni/(*S*,*S*)-*i*Pr-FcPHOX (**287**) (Scheme 122).^136^ This method allowed direct access to various bis-heterocycles **392** bearing an all-carbon quaternary center in moderate to good yields (up to 81%) with excellent enantioselectivity (up to 99% *ee*). Experimental evidence suggested that (i) no intermediacy of arylboronic reagent instead B_2_Pin_2_ acts as a coreducing agent; (ii) the enantioselective-determining step of the reaction is the migratory insertion, and it should be irreversible; (iii) the cyclization process is not the turnover-limiting step. A plausible mechanism of the reaction involved two reaction pathways. Initial oxidative addition of aryl bromide **391** to the Ni(0) species followed by an intramolecular carbonickelation results in the formation of a σ-alkyl-Ni(II)Br species **392-A**. In pathway A, **392-A** was then reduced by stoichiometric Zn/Pin_2_B_2_ to generate σ-alkyl-Ni(I)Br species **392-B**, which further undergoes a second oxidative addition with aryl bromide **391** to form the σ-alkyl-Ni(III)ArBr species **392-C**. Finally, reductive elimination of **392-C** affords the desired product **392** and regenerates the Ni(0) catalyst upon reduction with Zn/Pin_2_B_2_. Pathway B involves transmetalation between both Ni(II) centers **392-A** and ArNi(II)Br to form the σ-alkyl-Ni(II)ArBr species **392-D**, which generates product **392** following reductive elimination.

##### 2.1.3.5 FcPHOX Ligands for Ir-Catalyzed Asymmetric Hydrogenation

**Scheme 123:**
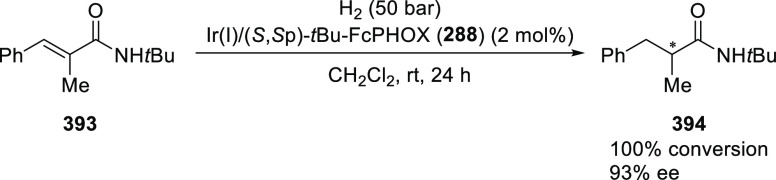
Asymmetric Hydrogenation of α,β-Unsaturated Amides

In 2009, Hou developed an asymmetric hydrogenation of α,β-unsaturated amides (**393**) using an Ir/(*S*,*S*_p_)-*t*Bu-FcPHOX (**288**) catalytic system to produce amides (**394**) with an α-chiral center in high yield and excellent enantioselectivity (Scheme 123).^137^ The presence of hydrogen on the amide nitrogen is imperative for the enantioselectivity of the reaction. Interestingly, amides having an aliphatic substituent at the β-position were also suitable substrates.

**Scheme 124:**
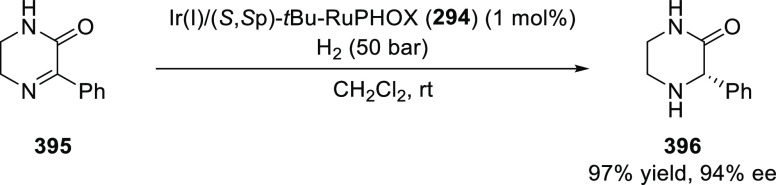
Asymmetric Hydrogenation of 5,6-Dihydropyrazin-2(1H)-ones

In 2017, Zhang developed a Ir/(*S*,*S*_p_)-*t*Bu-RuPHOX (**294**)-catalyzed asymmetric hydrogenation of 5,6-dihydropyrazin-2(1H)-ones (**395**) to synthesize chiral piperazin-2-ones (**396**) in excellent yields and with moderate to high *ee*s (up to 97% yield, up to 94% *ee*) (Scheme 124).^138^ This asymmetric hydrogenation was easily extended to protected and unprotected amide substrates.

**Scheme 125:**
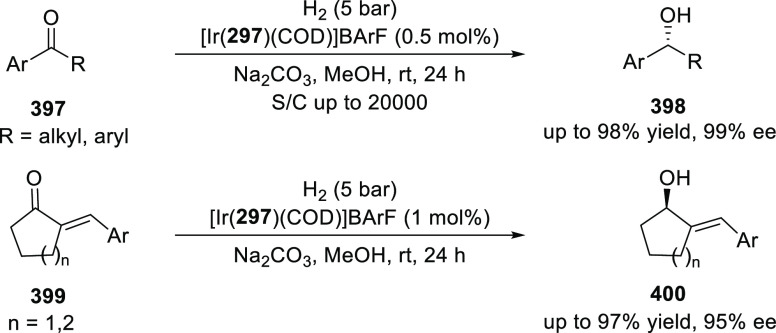
Asymmetric Hydrogenation of Ketones

Zhang reported the Ir-catalyzed asymmetric hydrogenation of simple ketones (**397**) and *exo*-α,β-unsaturated cyclic ketones (**399**) using a novel planar chiral ferrocene phosphinooxazoline ligand **297** (Scheme 125).^139^ A variety of ketones (**397/399**) were converted into their corresponding secondary alcohols (**398/400**) in up to 98% yield and 99% *ee*. The Ir-**297** complex was very stable and displayed high catalytic activity (0.005 mol %, S/C = 20 000) during the asymmetric hydrogenation of acetophenone on a 4.80 g scale providing alcohol product in 95% yield and 96% *ee*. Deuterium labeling studies confirmed that the reaction proceeds with hydrogen gas rather than via transfer hydrogenation with the alcoholic solvent.

**Scheme 126:**
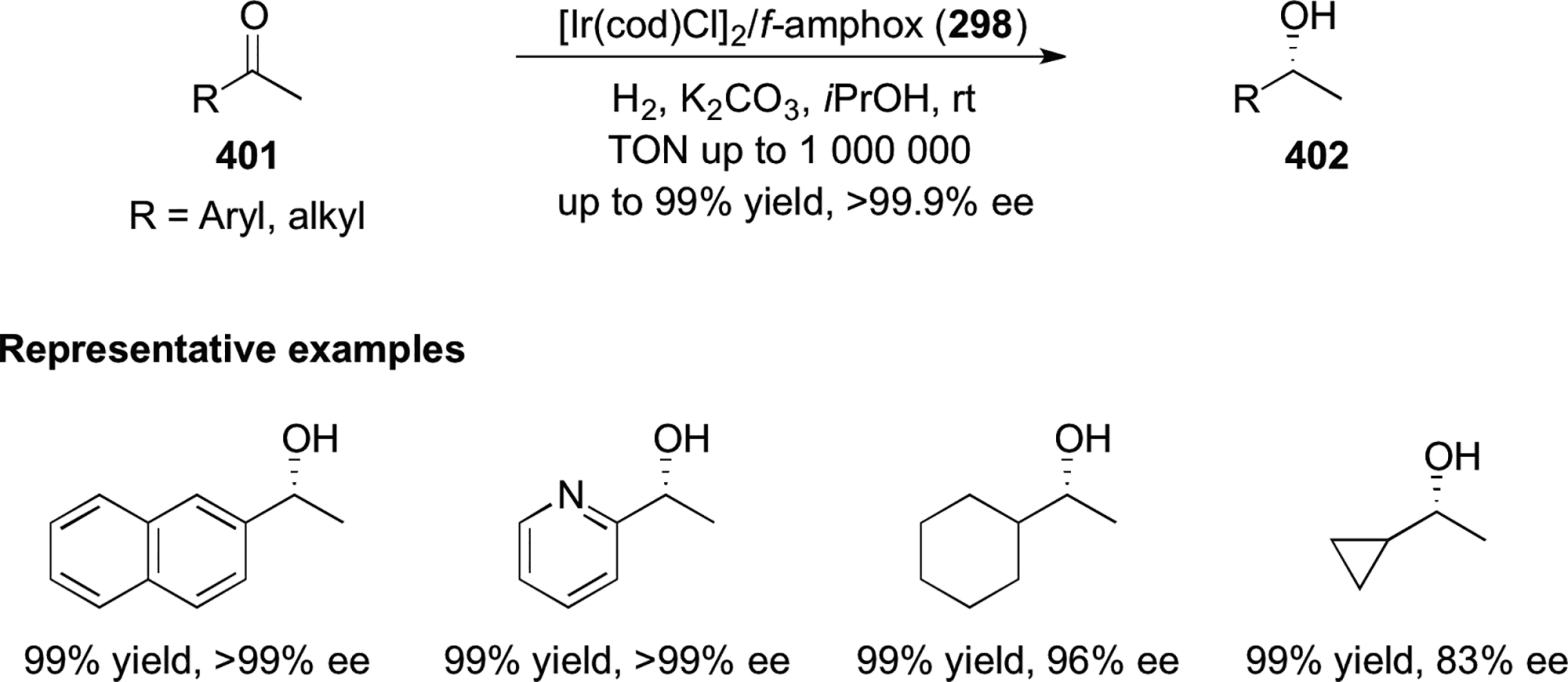
Asymmetric Hydrogenation of Methylketones

**Scheme 127:**
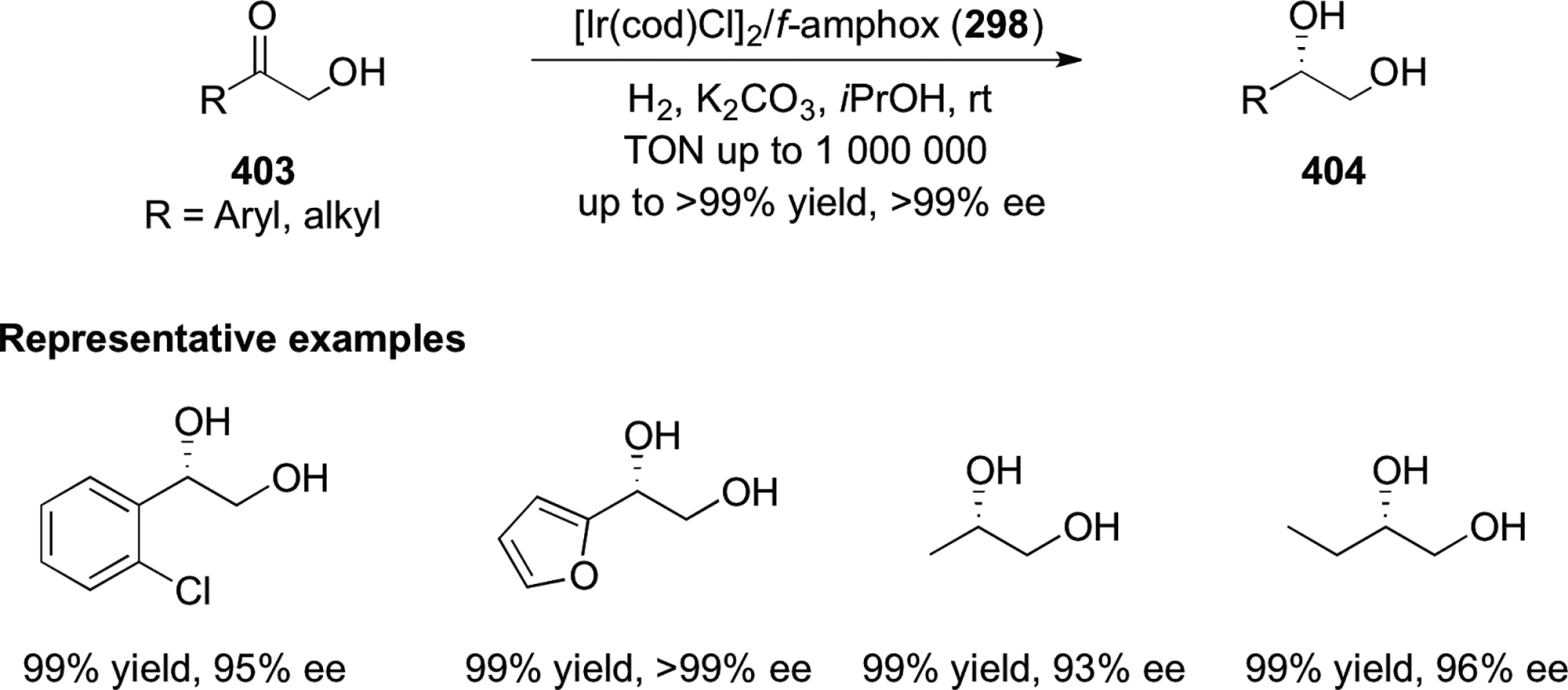
Asymmetric Hydrogenation of α-Hydroxy Ketones

**Scheme 128:**
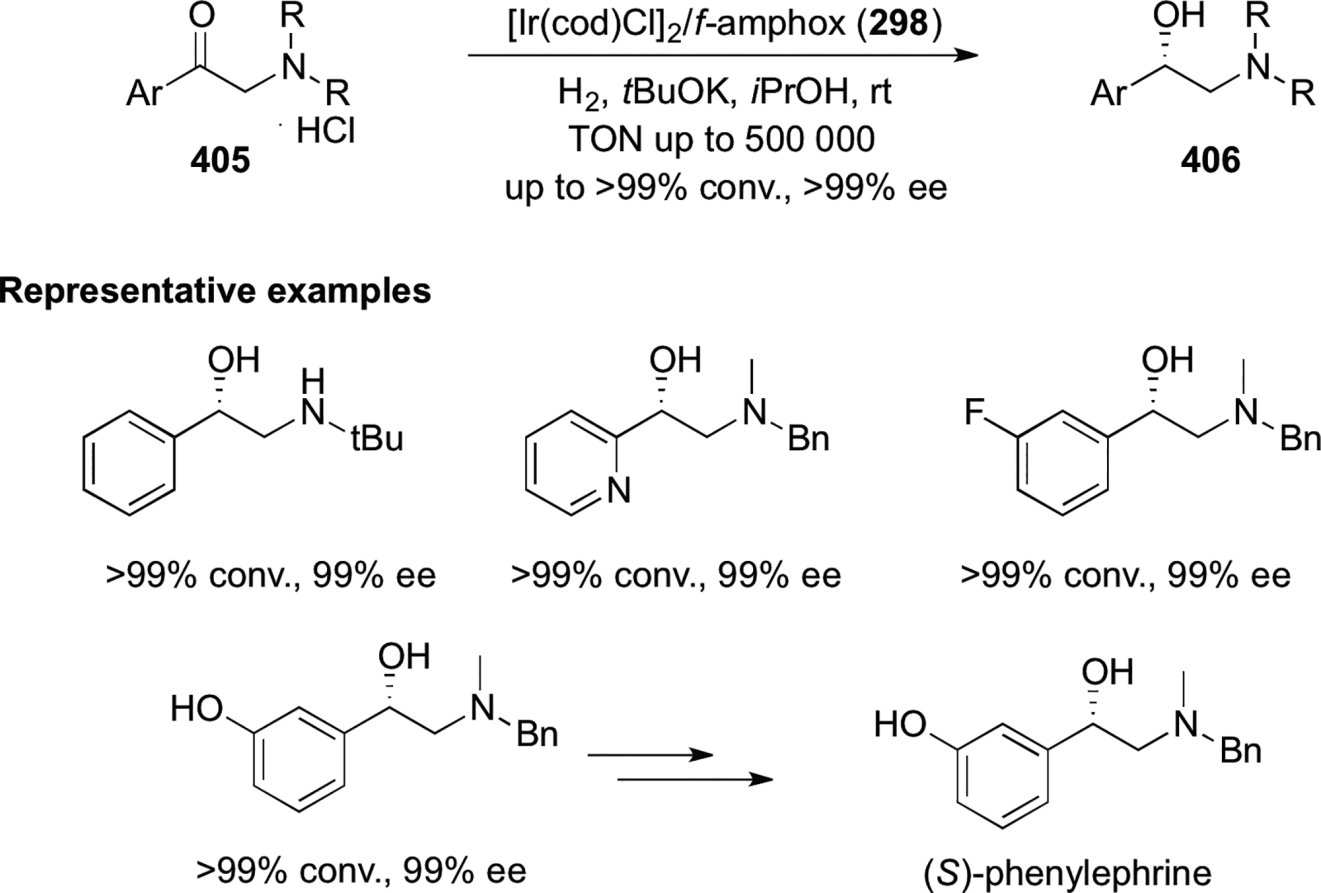
Asymmetric Hydrogenation of α-Amino Ketones

**Scheme 129:**
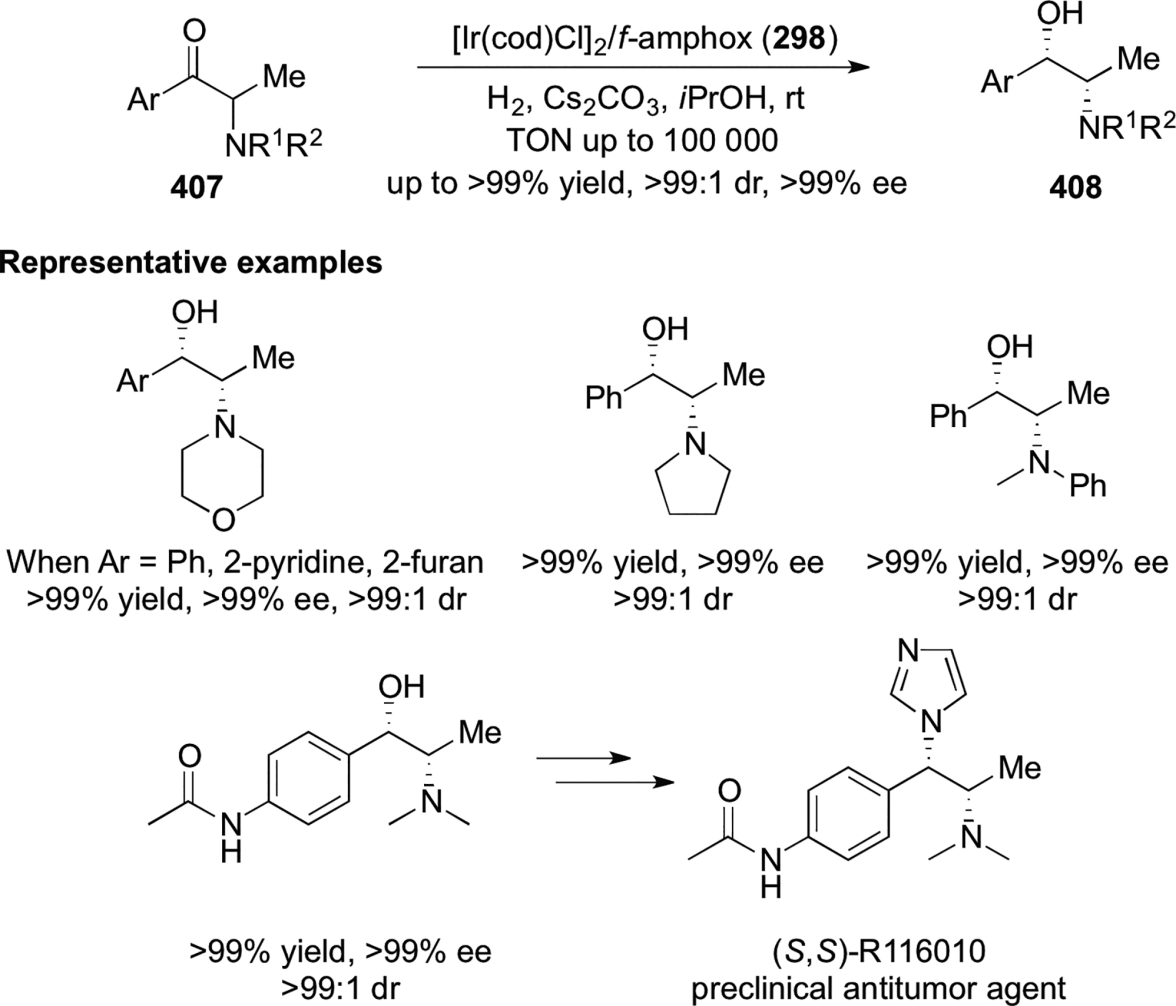
Asymmetric Hydrogenation of α-Amino Ketones *via* Dynamic Kinetic Resolution

**Scheme 130:**
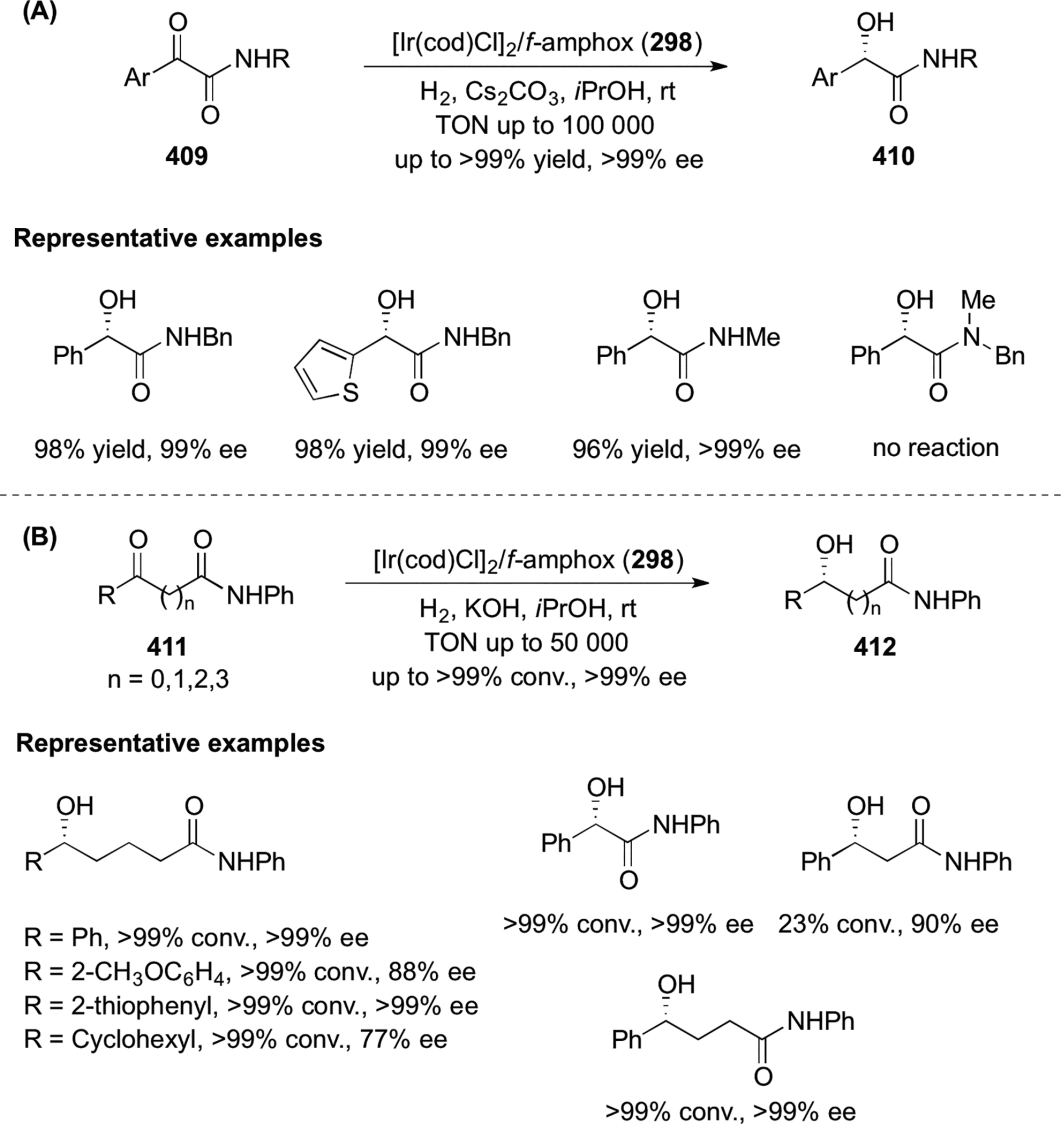
Asymmetric Hydrogenation of Ketoamides

**Scheme 131:**
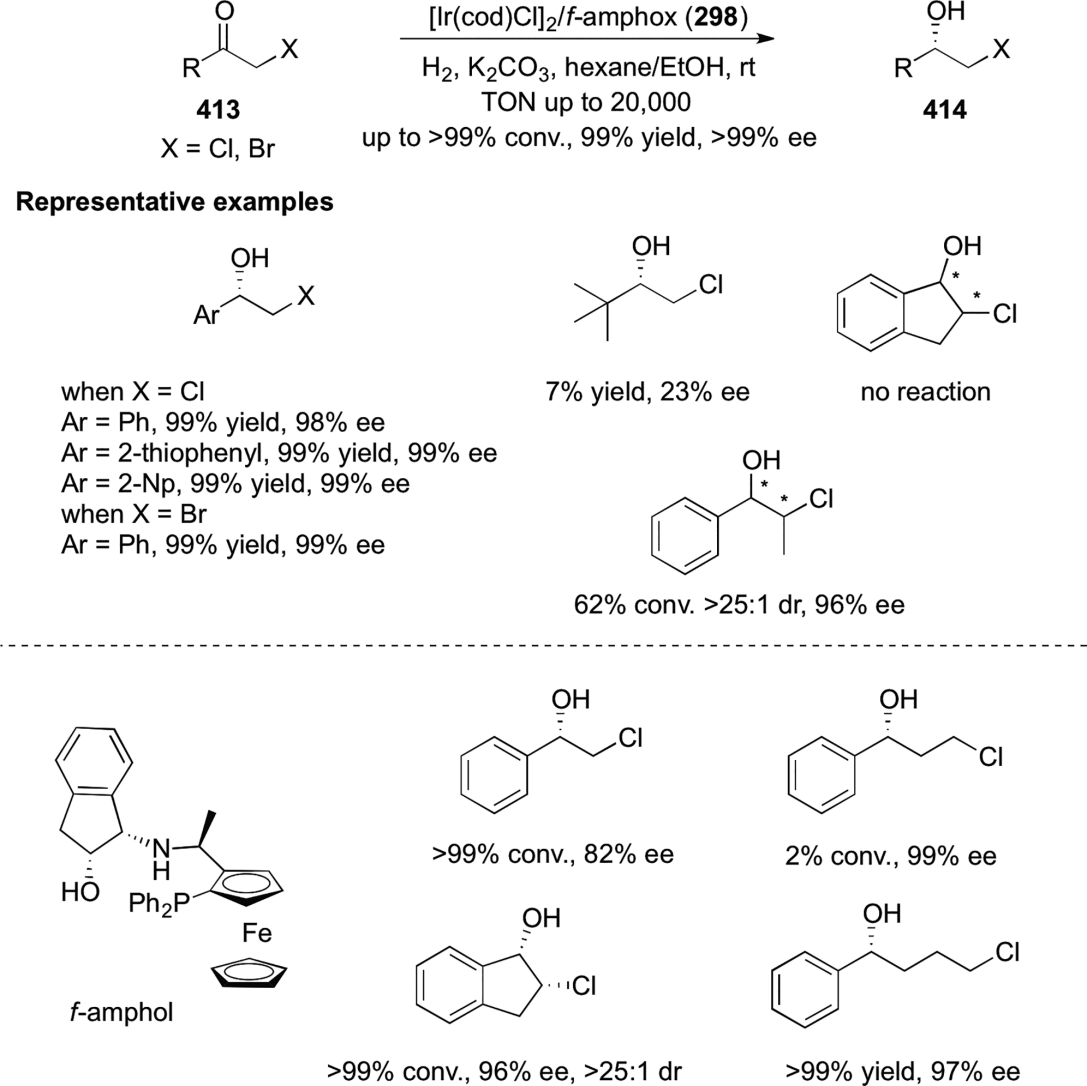
Asymmetric Hydrogenation of Halogenated Ketones

Zhang, Dong and Lan reported a novel modular electron-donating tridentate ligand having a chiral ferrocenylphosphine motif linked to the oxazoline unit through a secondary amine (Figure 4). The highly active and air-stable ferrocene aminophosphooxazoline (*f*-amphox, **298**) ligands were applied in the Ir-catalyzed asymmetric hydrogenation of various ketones to generate alcohol derivatives in excellent yields and enantioselectivities (Scheme 126-131).^140−146^

The initial application of this Ir-*f*-amphox catalyst in the asymmetric hydrogenation of simple ketones (**401**) provided the corresponding secondary alcohols (**402**) in excellent enantioselectivities (up to 99.9% *ee*) and activities (up to 1 000 000 TON) (Scheme 126).^140^ It was observed that the (*S*_c_,*S*_c_,*R*_Fc_) configuration of the *f*-amphox is very critical for enantioselectivity and reactivity.

Later, various α-hydroxy ketones (**403**) were converted to the corresponding chiral vicinal 1,2-diols (**404**) with excellent outcomes (TON up to 1 000 000, up to 99% yield, >99% *ee*) (Scheme 127).^141^

Similarly, the asymmetric hydrogenation of α-amino ketones (**405**) afforded the corresponding chiral 1,2-amino alcohols (**406**) in excellent activities and enantioselectivities (TON was up to 500 000, >99% conversion, >99% *ee*). The hydrogenated product possessing 3-hydroxy functionality on benzene ring is the key intermediate of (*S*)-phenylephrine, an enantiomer of an important α-adrenergic receptor agonist (Scheme 128).^142^

Asymmetric hydrogenation of a series of racemic α-amino β-unfunctionalized ketones (**407**) via a dynamic kinetic resolution (DKR) process allowed for the construction of a series of chiral 1,2-amino alcohols (**408**) bearing vicinal stereocenters with excellent results (all products >99% *ee* and >99:1 dr, TON up to 100 000). The success of this DKR was due more to the base-mediated rapid racemization of the substrate than hydrogenation. As expected, no reaction occurred without a base. The catalytic asymmetric hydrogenation with the process of DKR provided a powerful synthetic strategy for the synthesis of a key chiral intermediate of the preclinical antitumor agent (*S*,*S*)-R116010 (Scheme 129).^143^

Further, the asymmetric hydrogenation of a wide range of prochiral α-, β-, γ-, and δ-keto amides (**409**/**411**) was achieved to deliver a range of chiral hydroxy amides (**410**/**412**) with excellent results (TON was up to 100 000, >99% conversion and >99% *ee*). A DFT study suggested that noncovalent interactions between the ligand and substrate play an important role in achieving high enantioselectivity (Scheme 130A,B).^144,145^

Finally, the asymmetric hydrogenation of prochiral halogenated ketones (**413**) was also achieved to produce chiral halohydrins (**414**) with excellent results (TON up to 20 000, up to 99% yield and >99% *ee*) (Scheme 131).^146^

**Scheme 132:**
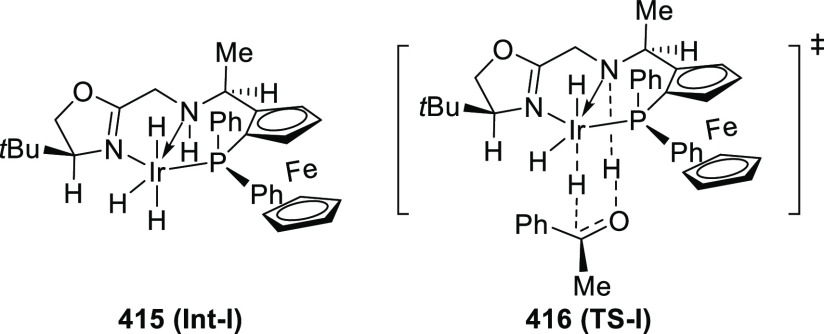
Mechanistic Studies for Asymmetric Hydrogenation of Acetophenone with *f*-Amphox

Mechanistic studies for the asymmetric hydrogenation of acetophenone indicated that the formation of intermediate **415** is kinetically and thermodynamically favored (Scheme 132). Then, the hydrogenation of acetophenone with intermediate **415** takes place via transition state **416** which forms the major product **402**. Studies showed that in **415** the right-hand side is occupied by the ligand’s cyclopentadiene moiety and an equatorial phenyl group, which blocks the right-hand side during hydrogenation and hence acetophenone is thought to approach the Ir-center of **415** by placing the larger group at the unencumbered left-hand side.

##### 2.1.3.6 Asymmetric Allylation with 1,1′-*P*,*N*-Ferrocene Ligand

**Scheme 133:**
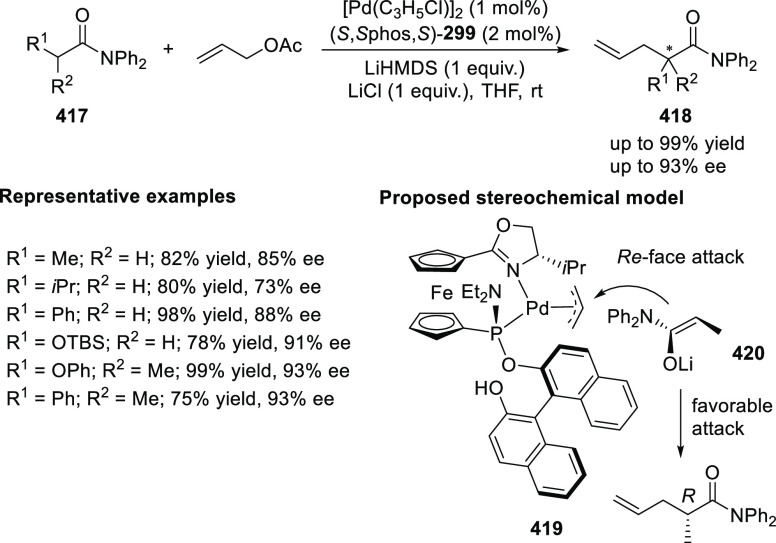
Asymmetric Allylic Alkylation of Acyclic Amides

In 2008, Hou developed a Pd-catalyzed asymmetric allylic alkylation of acyclic amides (**417**) using the 1,1′-*P*,*N*-ferrocene ligand (**299**) (Scheme 133).^147^ Among the various ligands tested, (*S*_c_,*S*_phos_,*S*_a_)-**299** afforded the allylated products in high yields (up to 99%) with excellent enantioselectivities (up to 93% *ee*). The nature of the substituents on the amide nitrogen and the use of allyl acetate instead of the corresponding carbonate or phosphonate had a significant influence on the efficiency and enantioselectivity of the reaction. The application of (*S*_c_,*S*_phos_,*S*_a_)-**299** without a stereogenic center in the oxazoline decreased the yield (65%) and *ee* of the product (55%) suggesting its importance. Also, the product configuration was determined by the stereochemistry of the Binol component rather than that of the phosphorus atom. The presence of a lithium counterion and the exact amount of base was crucial for the success of the reaction. In the proposed stereochemical model, the Binol subunit and the nucleophile occupies the same side of the π-allyl complex which favors *Re* face attack to provide the product with (*R*)-configuration.

**Scheme 134:**
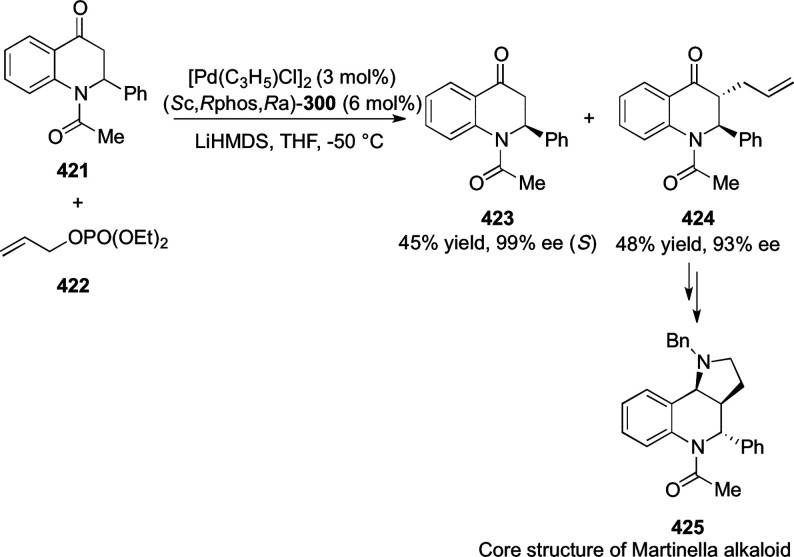
Kinetic Resolution of 2,3-Dihydro-2-substituted 4-Quinolones

Later in 2009, Hou reported the highly efficient kinetic resolution of 2,3-dihydro-2-substituted 4-quinolones (**421**) using a Pd/(*S*,*R*_phos_,*R*)-**300**-catalyzed asymmetric allylic alkylation (AAA) (Scheme 134).^148^ This method provided the allylated products, 2,3-disubstituted 2,3-dihydro-4-quinolones (**424**), with *trans*-selectivity in 37–48% yields and 83–93% *ee* along with 37–47% yields of recovered starting materials (**423**) in 87–99% *ee* (*S*-factor of 40–145). It is noteworthy that this method incorporates chiral centers at both α- and β-positions of the ketone. The nitrogen substituent of **421** also played a role in the reaction, as for example, when the acetyl group of 4-quinolone was replaced by H or Boc, both the allylated product and recovered substrates were obtained in lowered *ee*. The application of the methodology was demonstrated in the synthesis of pyrrolo[3,2-*c*]quinoline (**425**), a core structural motif of biologically active *Martinella* alkaloids.

**Scheme 135:**
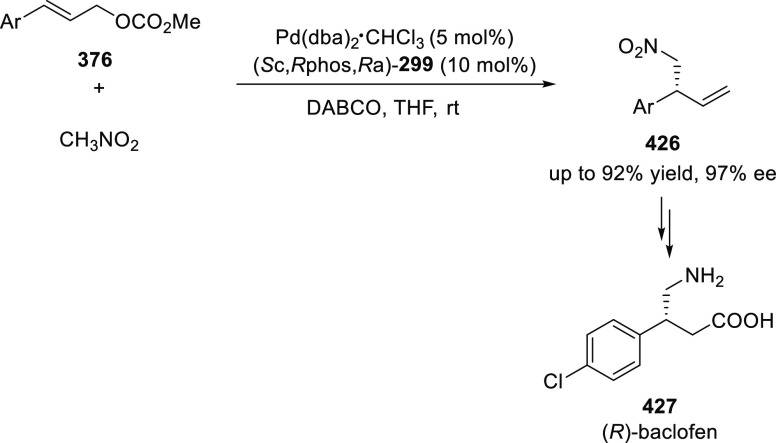
Asymmetric Allylic Alkylation of Nitromethane

In 2012, Hou reported a Pd/(*S*_c_,*R*_phos_,*R*_a_)-**299**-catalyzed asymmetric allylic alkylation (AAA) between allylic carbonate (**376**) and nitromethane (Scheme 135).^149^ Among the various ligands tested, (*S*_c_,*R*_phos_,*R*_a_)-**299** afforded branched allylated products (**426**) in high yields with excellent regio- and enantioselectivities. The synthetic application of the method was shown by preparing (*R*)-baclofen **427**, an antispasmodic and (*R*)-rolipram, an anti-inflammatory agent and antidepressant.

**Scheme 136:**
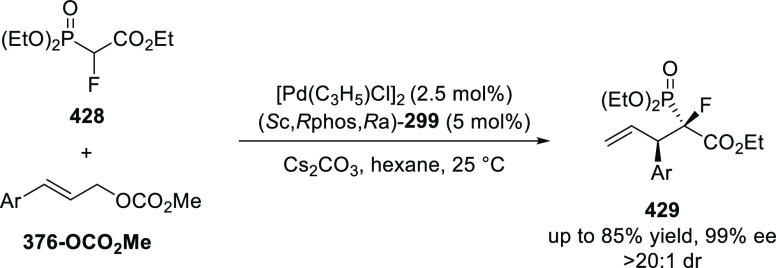
Asymmetric Allylic Alkylation of α-Fluoro-alkylphosphonates

In 2014, Hou reported a Pd/(*S*_c_,*R*_phos_,*R*_a_)-**299**-catalyzed asymmetric allylic alkylation of α-fluoro-alkylphosphonates (**428**) with monosubstituted allylic substrates (**376**) to afford allylated products (**429**) with two chiral centers in high regio-, diastereo- and enantioselectivities (Scheme 136).^150^ The reaction was highly diastereoselective for all allyl substrates tested possessing either electron-donating or electron-withdrawing substituents on the phenyl ring whereas the regioselectivity was sensitive to the substituent on the aryl group of the carbonates (**376**). The reaction of cinnamyl carbonate furnished the expected product with >20:1 branch to linear ratio, whereas any other substituent on the phenyl ring of carbonates (**376**) lowered the b/l ratio to 7:1. Additionally, highly functionalized products with two adjacent stereogenic centers and three functional groups were easily elaborated to more complex products.

To conclude this section, metal-complexes of PHOX ligands (**287**–**300**) with a stereoplane have been employed in various asymmetric cycloaddition reactions. Copper and nickel complexes were extensively employed in (I) [4 + 2], [4 + 3], [3 + 3], [2 + 2 + 2] cycloadditions, (II) 1,2- and 1,4-additions. Copper complexes in combination with palladium were excellent for asymmetric α-allylation of Schiff base activated amino acids. Nickel complexes were also used in intramolecular arylcyanation and diarylation of alkenes whereas iridium complexes were successful in alkene, imine and ketone hydrogenations.

#### 2.1.4 Phosphinooxazoline with Phosphorus Bonded to *N*- and *O*-Atom

**Figure 5:**
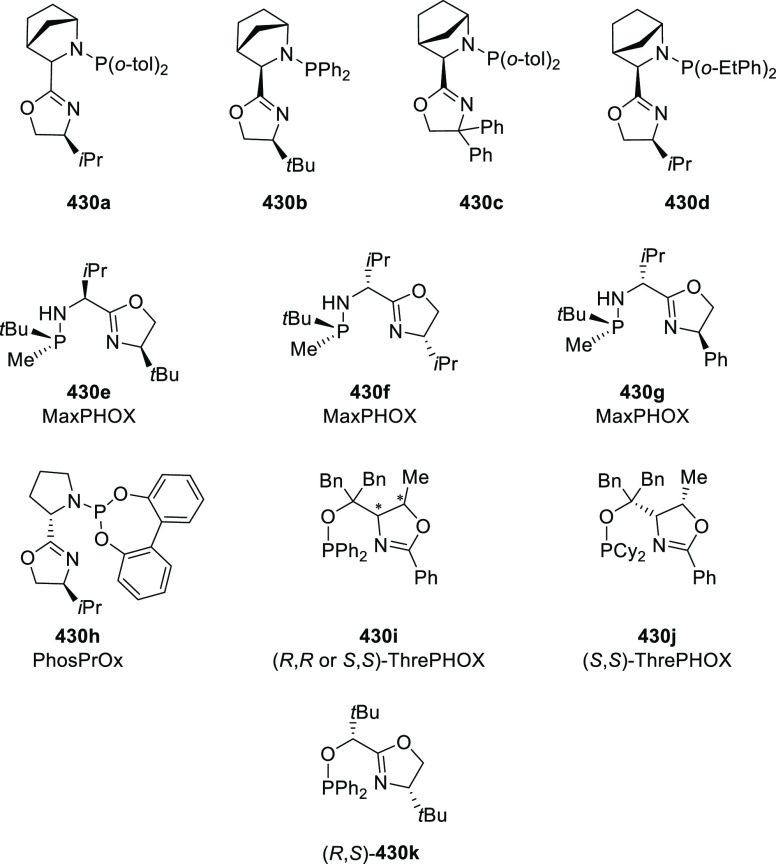
Phosphino-oxazoline ligands with Phosphorus Bonded to *N*- and *O*- Atom.

This section deals with the application of phosphino-oxazoline ligands with phosphorus bonded to *N*- and *O*- atom (**430a**–**k**) in Ir-catalyzed asymmetric hydrogenations. The phosphino-oxazoline ligands discussed in this section are shown in Figure 5.

**Scheme 137:**
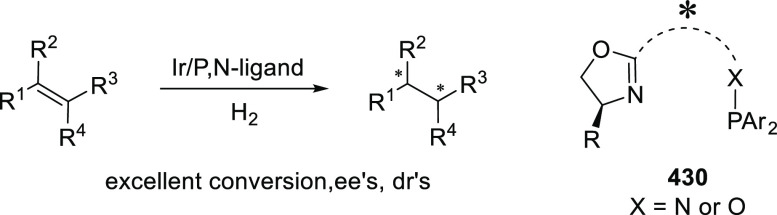
Ir-Catalyzed Asymmetric Hydrogenation of Olefins

The Andersson group reported a series of papers on the applications of *P*,*N*-ligands of type **430a**–**d** in the Ir-catalyzed asymmetric hydrogenation of various types of olefins (Scheme 137).

**Scheme 138:**
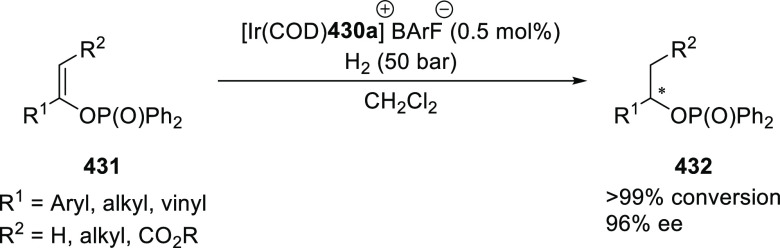
Ir-Catalyzed Asymmetric Hydrogenation of Di- and Trisubstituted Enol Phosphinates

In 2008, Andersson described the Ir-catalyzed asymmetric hydrogenation of a variety of di- and trisubstituted enol phosphinates (**431**) using ligand **430a** (Scheme 138).^151,152^ A range of olefin substrates possessing both aromatic and aliphatic substituents afforded hydrogenated products (**432**) with excellent enantioselectivities (up to >99% *ee*). Trisubstituted enol phosphinates were hydrogenated in better yields and selectivities than the related 1,1-disubstituted compounds. More importantly, enol phosphinates were stable toward degradation under the hydrogenation reaction conditions employed.

**Scheme 139:**
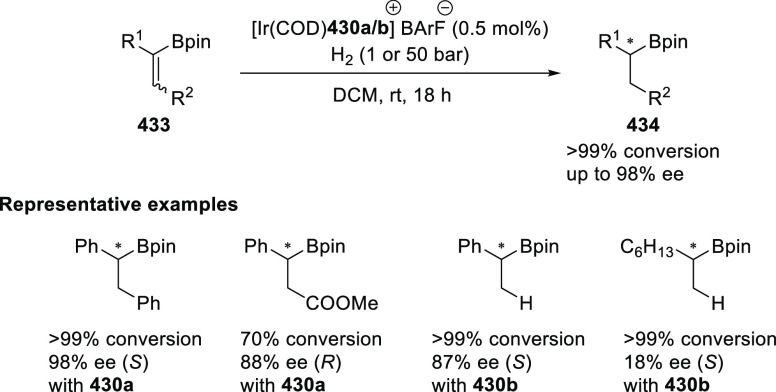
Ir-Catalyzed Asymmetric Hydrogenations of Vinyl Boronates

In 2009, the first Ir-catalyzed asymmetric hydrogenations of vinyl boronates (**433**) was developed using ligand **430a**/**b** under low catalyst loadings (0.5 mol %) and pressure (as low as 1 bar) (Scheme 139).^153^ For most of the vinyl boronates, the selectivity was either decreased or even reversed with decreased hydrogen pressure suggesting the possibility of different mechanisms at play at different concentrations of hydrogen. Vinyl boronates having aromatic or polarizing groups gave good results (98% *ee*), whereas aliphatic substituents on vinyl boronates were less successful as they gave hydrogenated products with <50% *ee*.

**Scheme 140:**
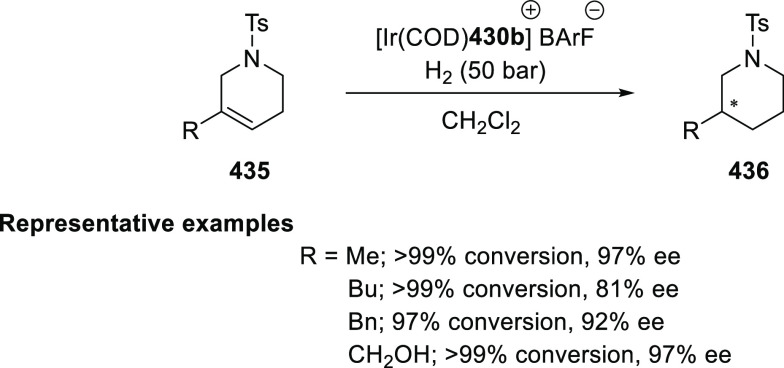
Ir-Catalyzed Asymmetric Hydrogenation of Cyclic Allylic Amines

Andersson subsequently employed ligand **430b** for the Ir-catalyzed asymmetric hydrogenation of cyclic allylic amines (**435**) thereby synthesizing chiral piperidines (**436**) in good to excellent enantioselectivities (Scheme 140).^154^

**Scheme 141:**
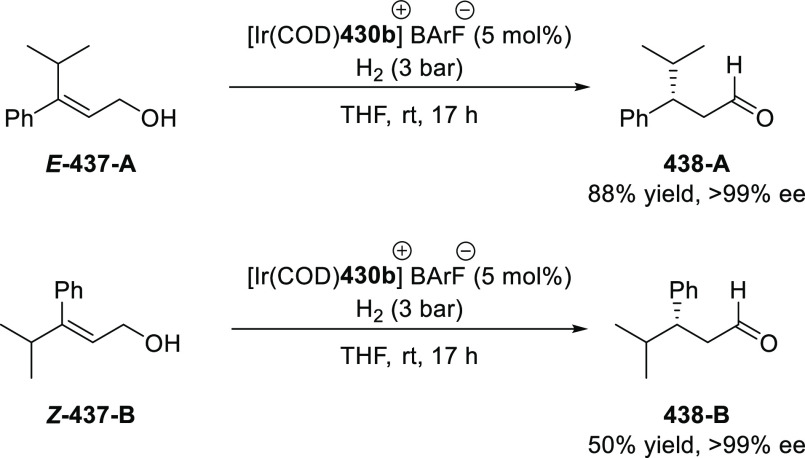
Asymmetric Isomerization of (*E*)- and (*Z*)-Trisubstituted Allylic Alcohols

In 2011, Andersson reported the enantioselective synthesis of β,β-disubstituted aldehydes (**438**) using the Ir-catalyzed asymmetric isomerization of (*E*)- and (*Z*)-trisubstituted allylic alcohols (**437**) (Scheme 141).^155^ The Ir complex of the bulkier ligand **430b** was successfully employed in the isomerization of a broad range of primary allylic alcohols. The yield of product was significantly impacted by the size of the substituent on the allyl alcohols. (*E*)-Cinnamyl alcohols with Cy and *i*Pr substituents reacted smoothly (86% yield, >99% *ee* and 88% yield, >99% *ee*). For ethyl and methyl substituents, the enantioselectivities were only marginally affected (97% *ee* and 91% *ee*), but the aldehyde product was recovered in poor yields (21% and <5%). Compared to the (*E*)-trisubstituted allylic alcohols, the catalytic system was less sensitive to steric effects in the isomerization of (*Z*)-trisubstituted allylic alcohols.

**Scheme 142:**
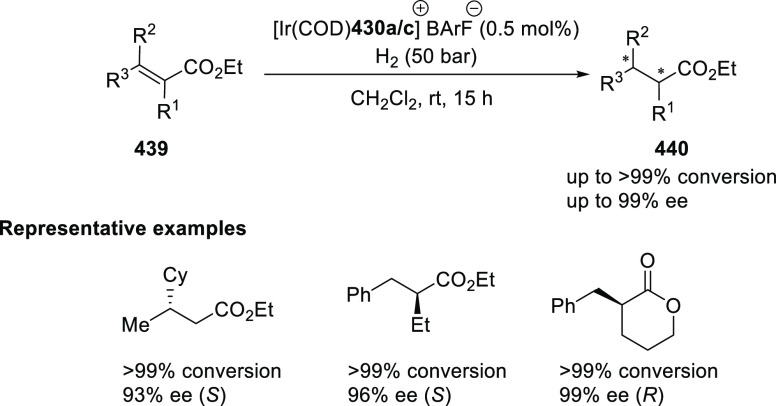
Asymmetric Hydrogenation of α,β-Unsaturated Esters

Later in 2012, the same catalytic system was extended to the asymmetric hydrogenation of α,β-unsaturated esters (**439**) (Scheme 142).^156^ A wide variety of ester substrates (**439**) with both aromatic and aliphatic substituents on the prochiral carbon were hydrogenated with excellent enantioselectivities (up to 99%).

**Scheme 143:**
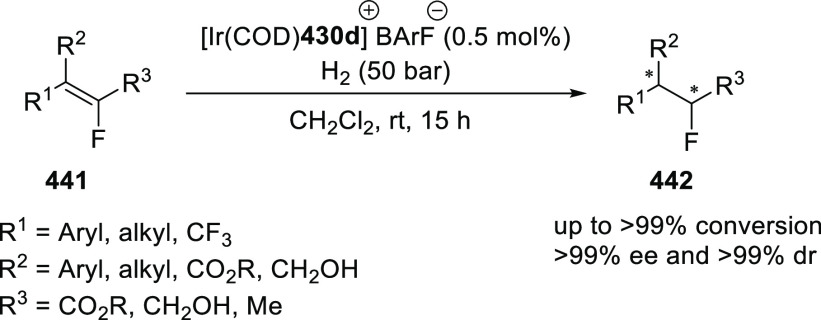
Asymmetric Hydrogenation of Tetra-Substituted Vinyl Fluorides

More recently, Andersson reported a highly stereoselective synthesis of chiral fluorine-containing compounds (**442**) with two vicinal stereogenic centers via the asymmetric hydrogenation of tetra-substituted vinyl fluorides (**441**) (Scheme 143).^157^ The Ir complex of the newly developed ligand (**430d**) was determined to be the most effective catalyst. Several aromatic, aliphatic, and heterocyclic substrates with a variety of functional groups were tolerated to provide chiral fluoroalkanes in excellent yield, diastereoselectivity, and enantioselectivity. Moreover, this catalytic hydrogenation system substantially overcomes the problem of defluorination.

**Scheme 144:**
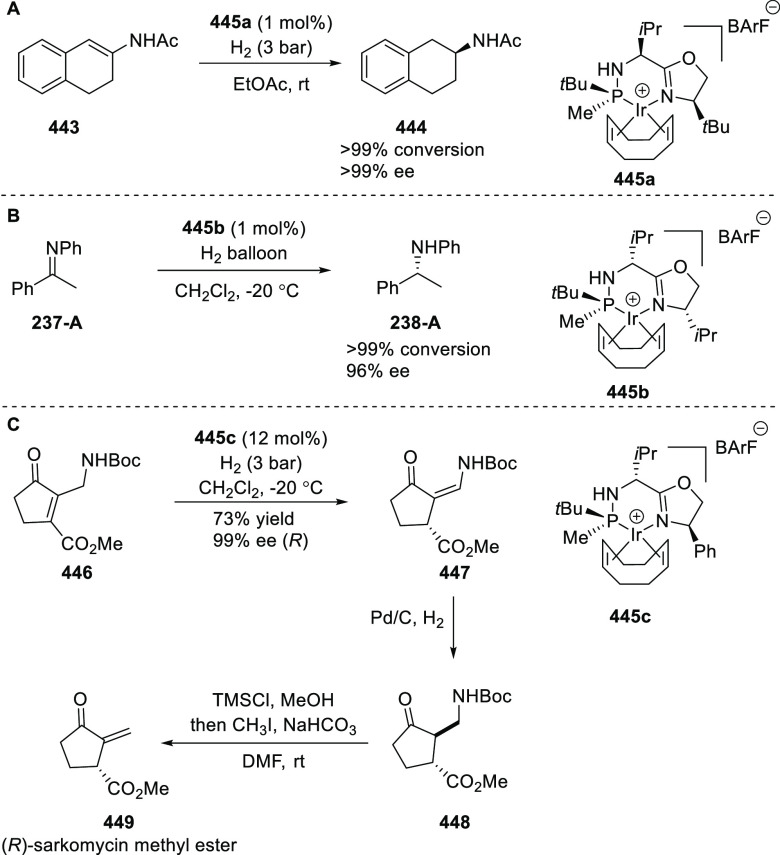
Ir-MaxPHOX Catalyzed Asymmetric Hydrogenation

Riera and Verdaguer developed a novel ligand, MaxPHOX (**430e**–**g**), which contains three stereogenic centers that can be introduced from three separate and simple building blocks. Initially, Ir-complexes of MaxPHOX-**445a** were applied in the asymmetric hydrogenation of cyclic enamides derived from α- and β-tetralones (**443**) (Scheme 144A).^158,159^ The enantioselectivities observed with Ir-MaxPHOX were much better than those with Ru and Rh catalysts. It was noticed that the enantioselectivity was pressure dependent as reducing the hydrogen pressure to 3 bar increased the enantioselectivity. The X-ray crystallographic study of Ir-**445a** complex showed that the six-membered metallacycle adopts a boat-like conformation. The phosphorus atom and the bulky *t*-butyl group on the oxazoline ring are *syn* to each other on the same face of the metallacycle. In addition to this the catalytic activity observed in coordinating solvents like EtOAc and MeOH suggested bidentate binding of the substrate to the cationic Ir-**445a** complex. It was believed that the directing amide group binds to an axial position away from the bulky *t*-butyl group, while the alkene binds equatorially *trans* to phosphorus. Finally, the asymmetric induction dependence on the hydrogen pressure indicated that hydrogen is involved in the enantioselectivity-determining step.

A further application of Ir-MaxPHOX-**445b** complexes was shown in the asymmetric hydrogenation of *N*-aryl imines (Scheme 144B).^160^ Ir-MaxPHOX-**445b** possessing an *i*Pr group both on the oxazoline ring and in the backbone with a P-stereogenic center was capable of efficient imine hydrogenation. The chirality on phosphorus has a significant impact on the catalyst activity as the non-P-stereogenic equivalent of Ir-MaxPHOX-**445b** gave rise to lower selectivity. Also, the nature of the counterion considerably affects the reaction outcome; smaller counterions (e.g., BF_4_) reduced the enantiomeric excess and slowed down the reaction.

Subsequently, the catalytic system was also employed in the synthesis of the antibiotic (*R*)-sarkomycin methyl ester (**449**) (Scheme 144C).^161^ The key step was an unprecedented asymmetric isomerization of cyclic enone **446** catalyzed by the Ir-MaxPHOX-**445c** complex resulting in the formation of exocyclic enamine in good yield (73%) and excellent enantioselectivity (99% *ee*). The exocyclic enamine **447** was formed because of stabilization due to the conjugation and formation of a strong intramolecular hydrogen bond. The exclusive formation of the enamide (*Z*)-isomer **447** also supported strong hydrogen bonding stabilization. The exocyclic enamine was subsequently reduced, the amine deprotected followed by spontaneous elimination to provide the desired antibiotic **449** in 45% yield and 98% *ee*.

**Scheme 145:**
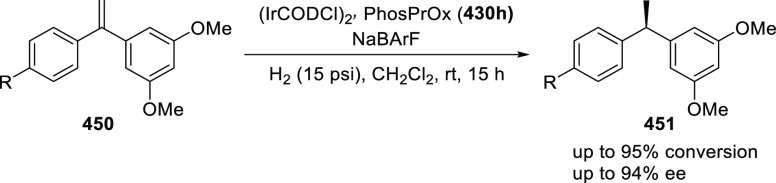
Ir-Catalyzed Asymmetric Hydrogenation of 1,1-Diarylalkenes

In 2013, Sigman developed a novel phosphoramidite ligand, Phos-PrOx (**430h**) and applied it in the Ir-catalyzed asymmetric hydrogenation of 1,1-diarylalkenes (**450**) (Scheme 145).^162^ The presence of a coordinating group on the *meta*-position of one of the phenyl rings was essential for the success of the reaction. It was proposed that the 3,5-dimethoxy substitution acts as a directing group by precoordinating the substrate alkenes to Ir. This coordination orients one alkene face toward Ir, poising the substrate for subsequent alkene coordination and hydrogenation to deliver 1,1-diarylmethines in moderate to good levels of enantioselectivity.

**Scheme 146:**
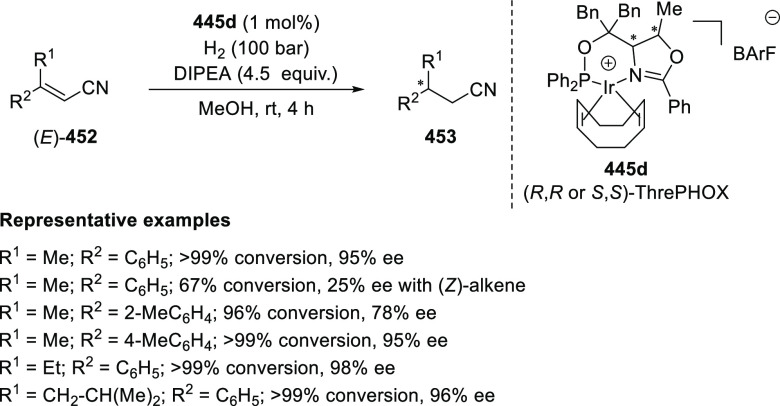
Ir-Catalyzed Asymmetric Hydrogenation of α,β-Unsaturated Nitriles

Pfaltz developed the first Ir-catalyzed asymmetric hydrogenation of α,β-unsaturated nitriles (**452**) using the chiral *P*,*N*-ligand, ThrePHOX (**430i**) and *N*,*N*-diisopropylethylamine (DIPEA) (Scheme 146).^163^ The Ir-**430i** complex (**445d**) was inactive without the addition of DIPEA. Slight variation in the ligand structure, changing the double bond geometry from *trans* to *cis* and the amount of DIPEA used all had a strong impact on the reactivity and selectivity of hydrogenation. Alkenes lacking a conjugated cyano group did not react in the present system which rendered the possibility of selectively reducing the cyano-substituted C=C bond of an α,β-unsaturated nitrile, while leaving the less electrophilic C=C bonds intact. The authors proposed the generation of a reactive neutral Ir(I) monohydride *via* base-mediated deprotonation of an Ir-dihydride complex due to its pronounced Brønsted acidity. The newly generated neutral Ir(I) monohydrate is expected to be less electrophilic which means easy release of nitrile from the Ir-metal center, thus opening the free coordination site required for the hydrogenation reaction. Also, the hydride of neutral Ir (I) monohydrate would be more nucleophilic than hydrides in a cationic dihydride complex, thus aiding hydride transfer to the electrophilic C=C bond of the α,β-unsaturated nitrile.

**Scheme 147:**
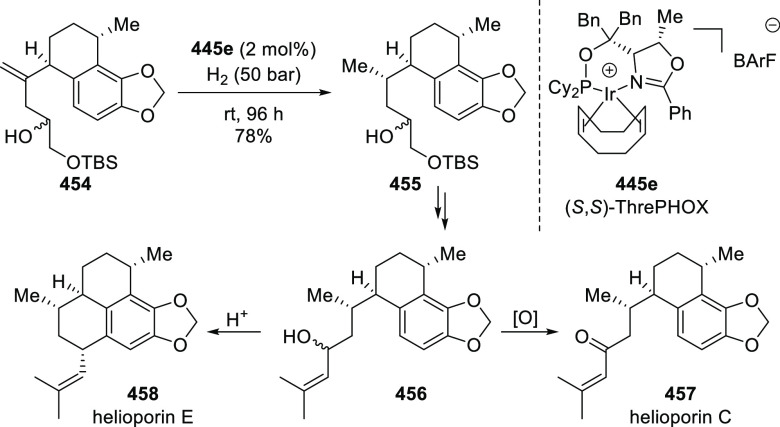
Total Synthesis of Helioporins C and E

The application of the ThrePHOX ligand (**430j**) developed by Pfaltz was demonstrated by Schmalz in a short total synthesis of helioporins C (**457**) and E (**458**) (Scheme 147).^164^ The Ir-catalyzed asymmetric hydrogenation of the exocyclic double bond was established for setting up a homobenzylic stereocenter in a highly diastereoselective manner. The hydrogenated product after subsequent desilylation, oxidative cleavage and vinyl Grignard addition afforded the allylic alcohol **456**. This allylic alcohol underwent oxidation to afford helioporins C (**457**) and acid-catalyzed cyclization to give rise to helioporins E (**458**).

**Scheme 148:**
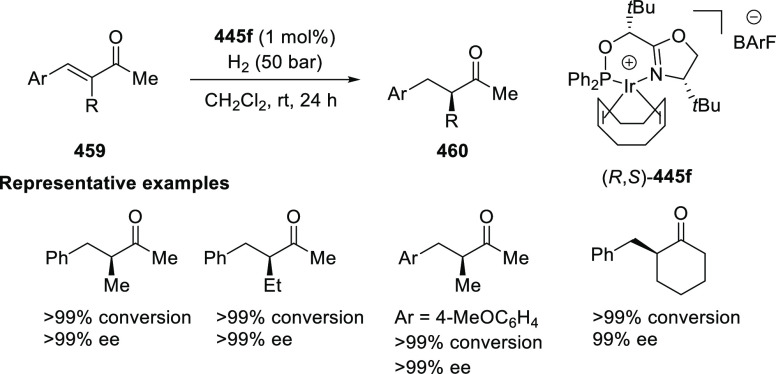
Ir-Catalyzed Asymmetric Hydrogenations of β-Arylated α,β-Unsaturated Ketones

Kazmaier described a new efficient *P*,*N*-ligand **430k** for Ir-catalyzed asymmetric hydrogenations of β-arylated α,β-unsaturated ketones (**459**) (Scheme 148).^165^ By using Ir-complex (**445f**) derived from ligand **430k**, various linear as well as cyclic α,β-unsaturated ketone substrates were hydrogenated successfully to the corresponding ketones (**460**) in excellent yields (>99%) and enantioselectivities (>99% *ee*).

To conclude this section, iridium complexes of PHOX ligands (**430a**–**k**) with phosphorus bonded to *N*- and *O*- atoms have been mainly employed in asymmetric alkene hydrogenation.

### 2.2 Mono(oxazoline) *N*,*N*-Ligands

**Figure 6:**
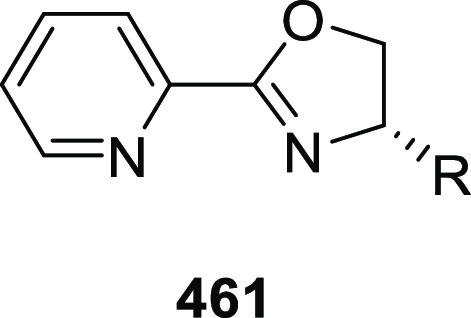
PyOx ligand.

Mono(oxazoline) bidentate *N*,*N*-ligands are also widely used in asymmetric catalysis. PyOx **461**, which contains a pyridine-oxazoline backbone is the most used ligand in this class (Figure 6). These ligand class will not be discussed here since Yang and Zhang recently reviewed the area since Brunner’s original synthesis in 1986.^166,167^

### 2.3 Mono(oxazoline) *N*,*S*-Ligands

**Scheme 149:**
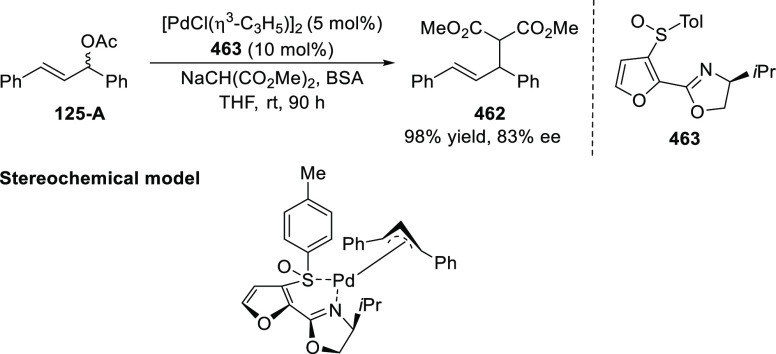
Pd-Catalyzed Allylic Alkylation with (Sulfinyl)furyl Oxazoline Ligand

In 2008 Arai developed a series of novel chiral (sulfinyl)furyl oxazoline-containing ligands **463** and applied them in the Pd-catalyzed allylic alkylation of 1,3-diphenylpropenyl acetate **125-A**. After optimization, which included changing the solvent from dichloromethane to THF, it was shown that the product **462** was formed in up to 98% yield and 83% *ee*. It is thought that the oxazoline nitrogen acts as a π-donor while the sulfur is a π-acceptor leading to the reactive intermediate shown (Scheme 149).^168^

**Scheme 150:**
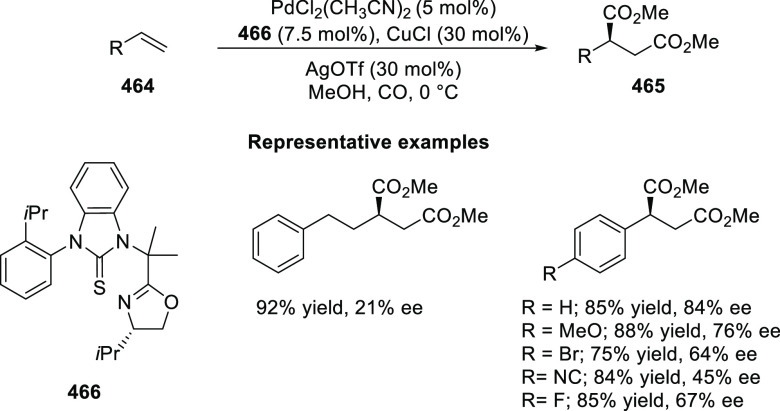
Pd-Catalyzed Esterification of Styrenes

In 2010 Huang reported the synthesis of axially chiral thiourea-oxazoline ligands in the Pd-catalyzed enantioselective bis(methoxycarbonylation) of styrenes **464**. When applied in this catalytic transformation ligand **466** afforded the product **465** in up to 85% yield and 84% *ee* (Scheme 150). A variety of electronically different styrenes **464** were successfully transformed to the product **465**, although when nonstyrenyl alkenes were used, the enantioselectivity dropped dramatically to 21% *ee* although still in 92% yield.^169^

**Scheme 151:**
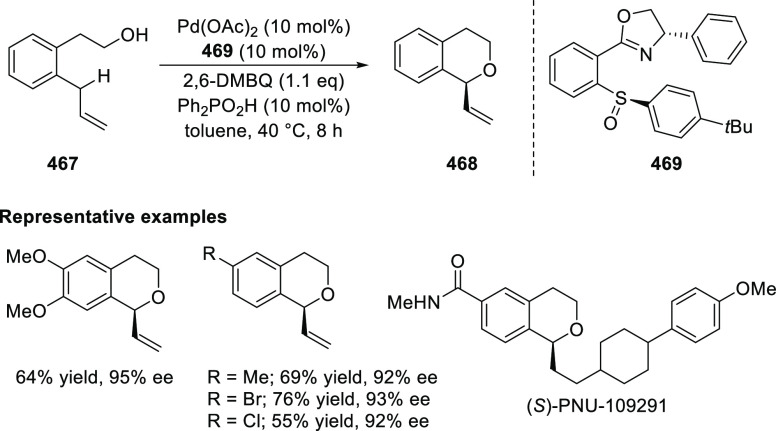
Pd-Catalyzed Synthesis of Isochroman Motifs

In 2016 White developed a novel set of aryl sulfoxide-oxazoline ligands **469** and applied them in the Pd-catalyzed synthesis of isochroman motifs **468**. A series of oxazoline substituents were screened and the ligand containing the phenyl group gave rise to the desired product in up to 64% yield and 95% *ee* (Scheme 151). The substrate scope included a range of electronically diverse isochroman motifs with 92% *ee* being the lowest obtained for all substrates. This methodology was subsequently applied in the synthesis of PNU-109291 a potent 5HT_1D_ agonist avoiding the costly chiral resolution used in its previous synthesis.^170^ In a subsequent publication White further explored the ligand scaffolds performance in the Pd-catalyzed asymmetric allylic alkylation of pyrazole-5-ones.^171^

**Scheme 152:**
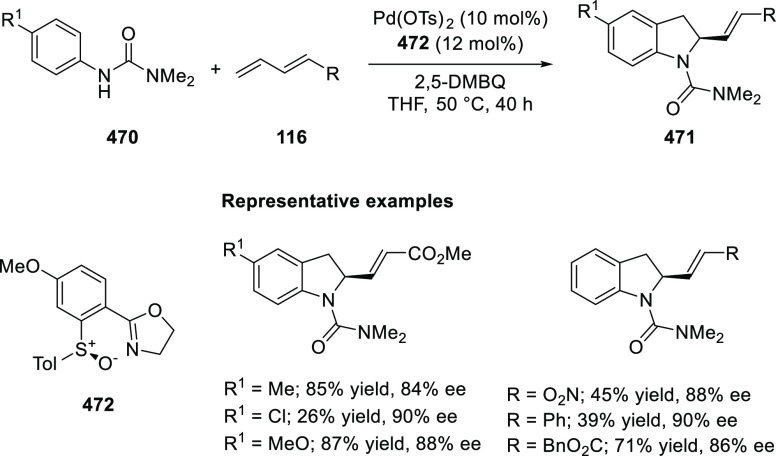
Pd-Catalyzed Asymmetric Intramolecular Allylation of Aryl Ureas

Han reported the asymmetric intramolecular allylation of aryl ureas **470** to indolines **471** using a novel sulfoxide-oxazoline ligand **472**. A variety of dienes **116** were applied in this transformation with all substituents furnishing the products **471** in a range of 80 to 90% *ee* with yields proving highly variable on the substrate used. Interestingly, this ligand does not possess a chiral oxazoline motif and instead relies on asymmetric induction from the chiral sulfoxide unit. When initial conditions were screened the BOX ligand **600c** afforded the product in only trace amounts. In the initial screen, benzoquinone was used as the oxidant furnishing the product in 77% yield and 85% *ee*, and when 2,5-dimethylquinone 2,5-(DMBQ) was used it improved the results moderately to 79% yield and 88% *ee* (Scheme 152).^172^

**Figure 7:**
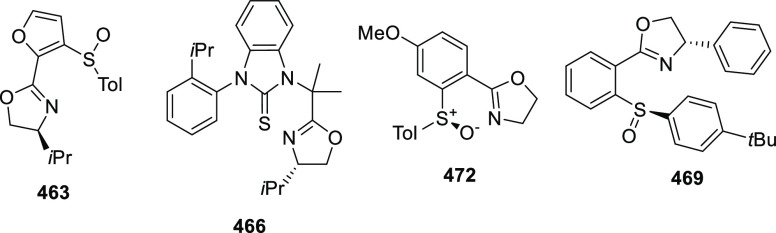
Summary of mono(oxazoline) *N*,*S*-ligands.

Interestingly, the mono(oxazoline) *N*,*S*-ligands mentioned in this section (Figure 7) are all used in Pd-catalyzed asymmetric reactions with unsaturated systems. While the chemistry is synthetically useful it appears this class of ligand is currently limited in their application. Utilizing these ligands in a wider range of chemistry with inexpensive metals could further develop this area greatly.

### 2.4 Mono(oxazoline) *N*,*O*-Ligands

**Figure 8:**
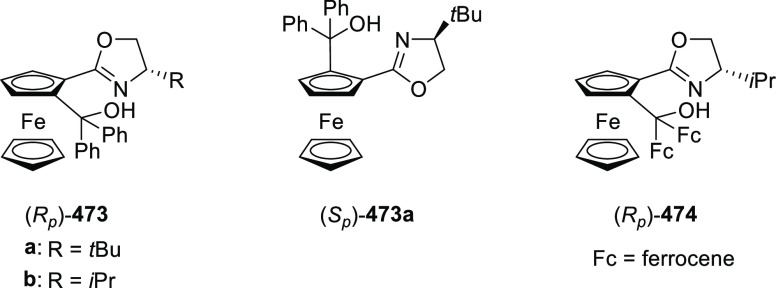
*N*,*O*-Ferrocenyloxazoline ligands **473** and **474**.

*N*,*O*-Ferrocenyloxazoline ligands (*R*_*p*_)- and (*S*_*p*_)-**473a** were originally synthesized by Bolm in 1997 (Figure 8).^173^ Since then, Butenschön has described the synthesis of (*R*_*p*_)-**473b** and triferrocenylmethane **474** and applied ligands (*R*_*p*_)-**473a**–**b** and **474** in the diethylzinc addition to aromatic and alkenyl aldehydes, achieving enantioselectivities of up to 97% *ee*.^174^

In almost all of the examples reported, triferrocenylmethane **474** gave the best enantioselectivity. To exemplify, the results of each ligand in the diethylzinc addition to benzaldehyde **475** are summarized in Table 1. The reaction with Bolm’s *t*Bu-**473a** afforded the chiral alcohol **476** in a higher enantiomeric excess (93% *ee*) than Butenshön’s *i*Pr-**473b** (83% *ee*), but Butenschön’s triferrocenyl *i*Pr-**474** gave the best result (97% *ee*). The increased steric bulk beside the alcohol moiety counteracts the decrease in steric bulk going from *t*Bu to *i*Pr on the oxazoline.

**Table 1:**
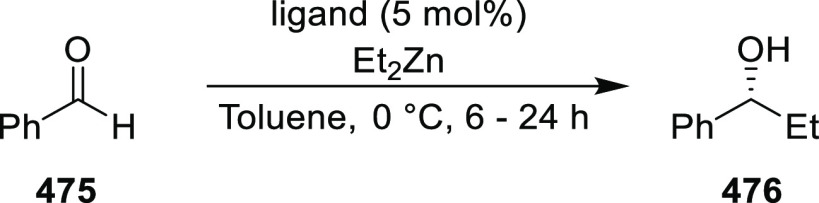
Comparison of Bolm and Butenschön’s *N*,*O*-Ferrocenyloxazoline Ligands in Asymmetric Diethylzinc Addition to Benzaldehyde **475**

entry	ligand	yield **478** (%)	% *ee*
1	(*R*_*p*_)-**473a**	83	93
2	(*R*_*p*_)-**473b**	97	83
3	(*R*_*p*_)-**474**	95	97

**Scheme 153:**
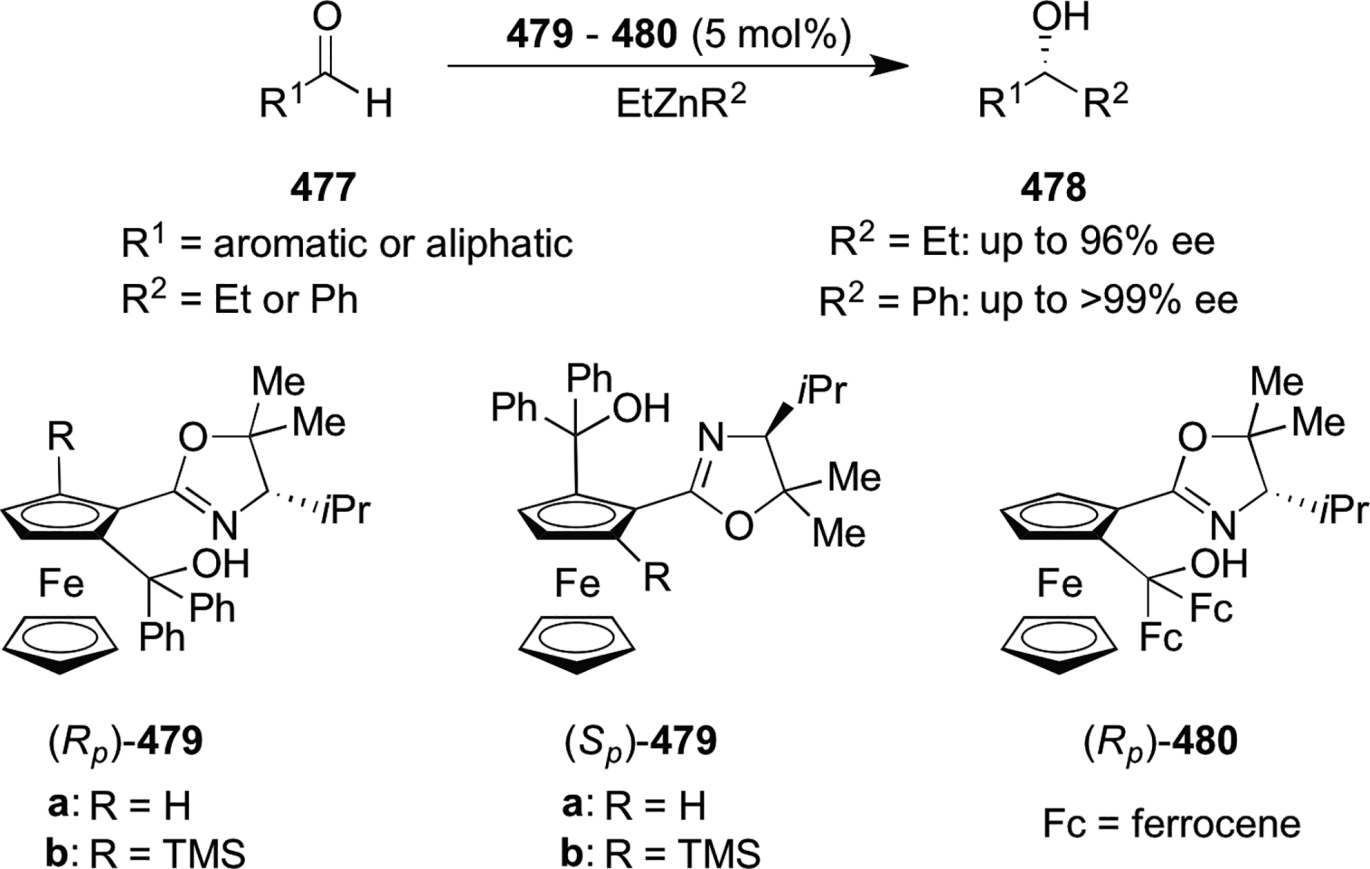
Application of Guiry’s *gem*-Dimethyl *N*,*O*-Ferrocenyloxazoline Ligands in Diethylzinc Addition to Various Aldehdyes **477**

More recently, Guiry has studied the *gem*-dimethyl effect in these *N*,*O*-ferrocenyloxazoline ligand systems, describing the synthesis of ligands (*R*_*p*_)- and (*S*_*p*_)-**479a**–**b** and (*R*_*p*_)-**480**, applying them in the diethyl- and diphenylzinc addition to aromatic and aliphatic aldehydes **479**, with enantiomeric excesses up to >99% (Scheme 153).^175^

The results of the diethylzinc additions to benzaldehyde **475** with each ligand are summarized in Table 2. In the case of benzaldehyde, the triferrocenylmethane ligand (**480**) performed best, giving the chiral alcohol **476** in 93% *ee* (entry 5). However, for different aldehydes, different ligands performed better. This study shows that the *gem*-dimethyl effect can be applied to ligands of this class to reach similar levels of enantioselectivity that can be achieved with less economical *t*Bu-ligands, with (*R_p_*)-**479a** giving the chiral alcohol **476** in 88% *ee* (compared to 93% *ee*) (entry 1). Second, trisubstituted ferrocene derivatives (**479b**) perform worse than the corresponding disubstituted ferrocene derivatives, with (*R*_*p*_)-**479b** giving the chiral alcohol **478** in 82% *ee* (entry 3). Finally, in this chiral ligand scaffold, planar chirality has a dominant effect over the stereoselectivity induced, with the (*S_p_*)-ligands giving lower levels of enantioselectivity in the diethylzinc additions, while reversing the enantioselectivity in the diphenylzinc additions (entries 2 and 4).

**Table 2:**
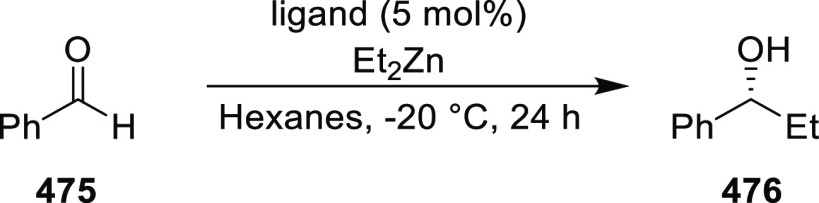
Comparison of Guiry’s *gem*-Dimethyl *N*,*O*-Ferrocenyloxazoline Ligands in Asymmetric Diethylzinc Addition to Benzaldehyde **475**

entry	ligand	yield **478** (%)	% *ee*
1	(*R*_*p*_)-**479a**	93	88
2	(S_*p*_)-**479a**	32	33
3	(*R*_*p*_)-**479b**	54	82
4	(S_*p*_)-**479b**	11	–5
5	(*R*_*p*_)-**480**	79	93

**Figure 9:**
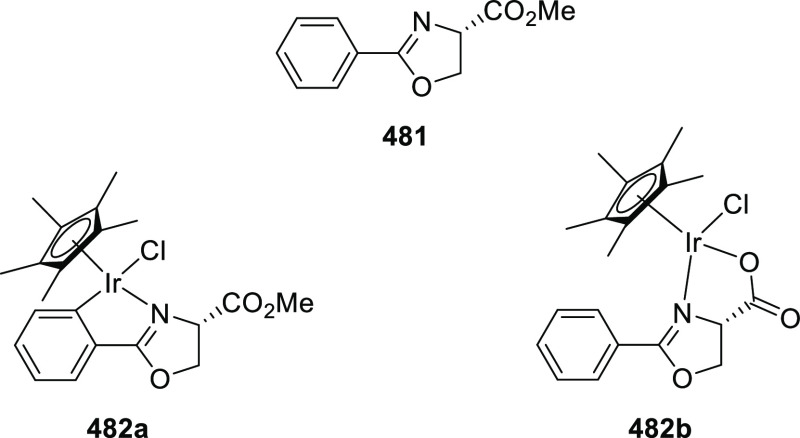
Xiao and Chen’s *N*,*O*-ligand **481** and Ir(**481**)Cp*Cl **482a** and **482b**.

Xiao and Chen reported the synthesis of *N*,*O*-ligand **481** bearing a phenyl group at the *C*-1 position of the oxazoline and an ester at the *C*-4 stereocenter (Figure 9).

**Scheme 154:**
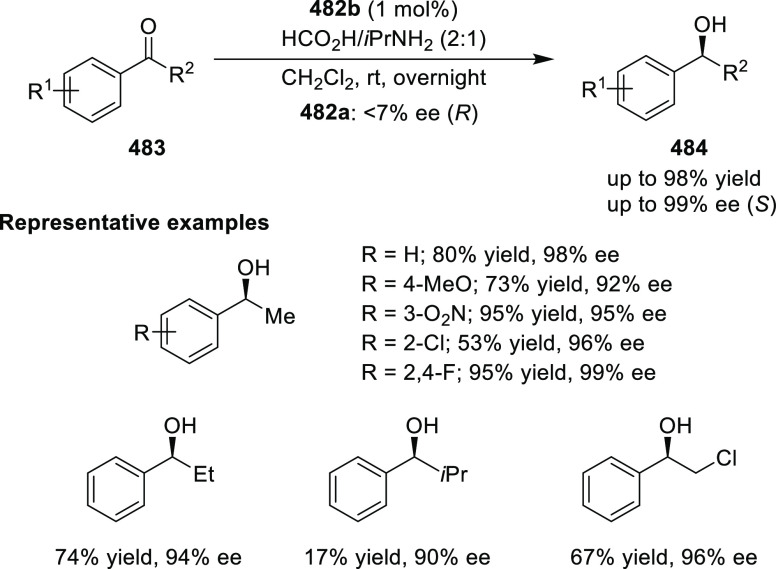
Ir-Complex **482b**-Catalyzed Asymmetric Transfer Hydrogenation of Aryl Ketones **483**

They applied this ligand in the asymmetric transfer hydrogenation of aryl ketones **483**, having prepared both the *N*,*C*- and *N*,*O*-chelated Ir complexes **482a** and **482b**. It was observed that **482a** did not induce appreciable levels of enantioselectivity in this reaction, while the reaction with **482b** gave the corresponding secondary alcohols **484** in up to 98% yield and with up to >99% *ee* (Scheme 154).^176^

**Figure 10:**
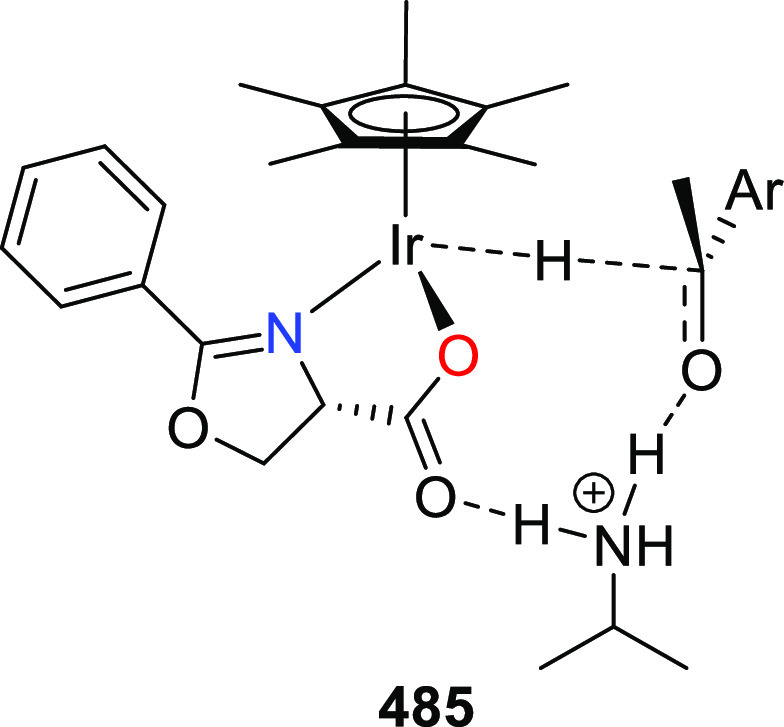
Plausible transition state for the asymmetric transfer hydrogenation of ketones **483** with Ir-complex **482b**.

A plausible transition state **485** for this process was proposed in which the hydride is delivered to the *Re*-face of the ketone, with the ammonium cation H-bonding to the *N*,*O*-ligand and the ketone (Figure 10).

**Figure 11:**
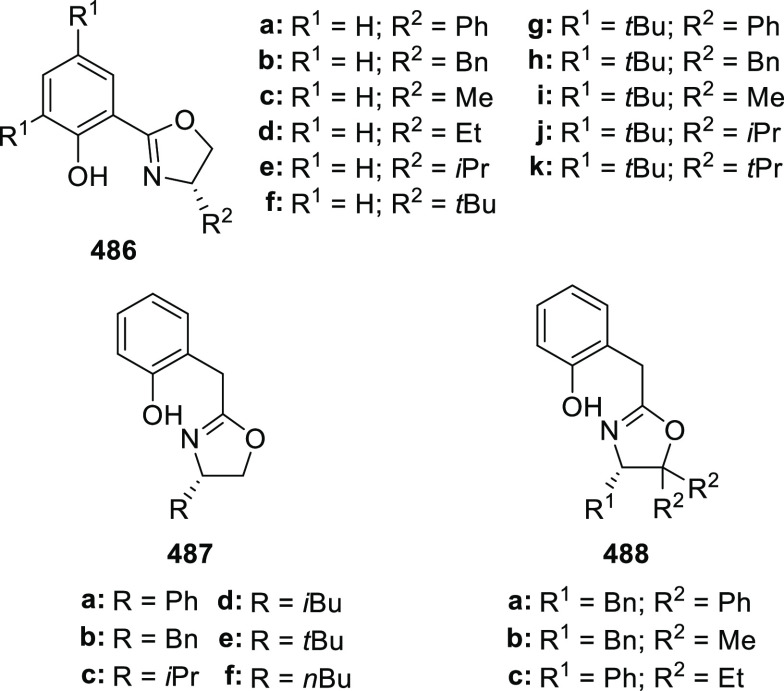
Box–OH ligands **486**–**488**.

In recent years, Wang has significantly contributed to the development of *N*,*O*-analogues of PHOX-type ligands, describing the synthesis of Box–OH ligands **486** – **488** and their application in various enantioselective Mg(II)-catalyzed transformations (Figure 11).

**Scheme 155:**
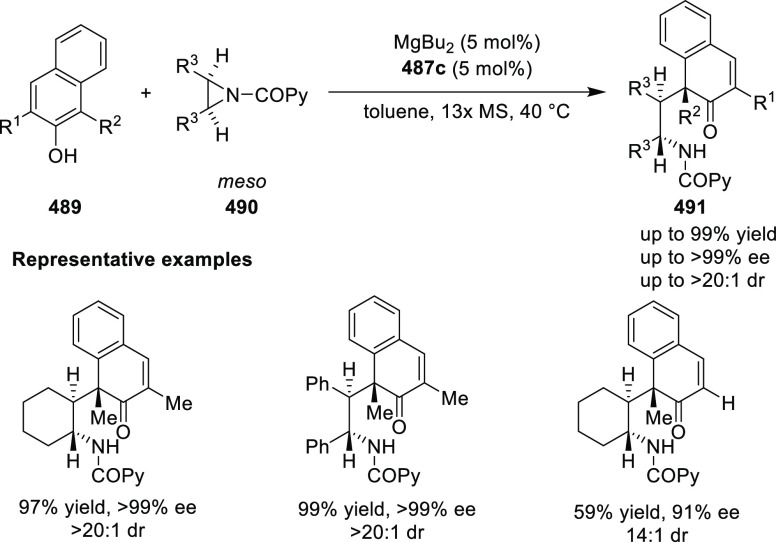
MgBu_2_/**487c**-Catalyzed Asymmetric Dearomatization of β-Naphthols **489** with *meso*-Aziridines **490**

Wang first utilized the Box–OH ligands in the Mg-catalyzed asymmetric dearomatization of β-naphthols **489** with *meso*-aziridines **490**. Following optimization, ligand **487c** was applied in the synthesis of a range of ring-opened products **491** bearing three contiguous stereocenters in up to 99% yield, with up to >20:1 dr and >99% *ee* (Scheme 155). Ligands of the type **487**, with a methylene spacer between the phenol and the oxazoline, performed better than ligands **486** without the spacer. Changes to the β-naphthols **489** or the *meso*-aziridines **490** did not have a significant effect on the outcome of the reactions, all of which proceeded with excellent levels of enantioselectivity (all but one example >97% *ee*).^177^

**Scheme 156:**
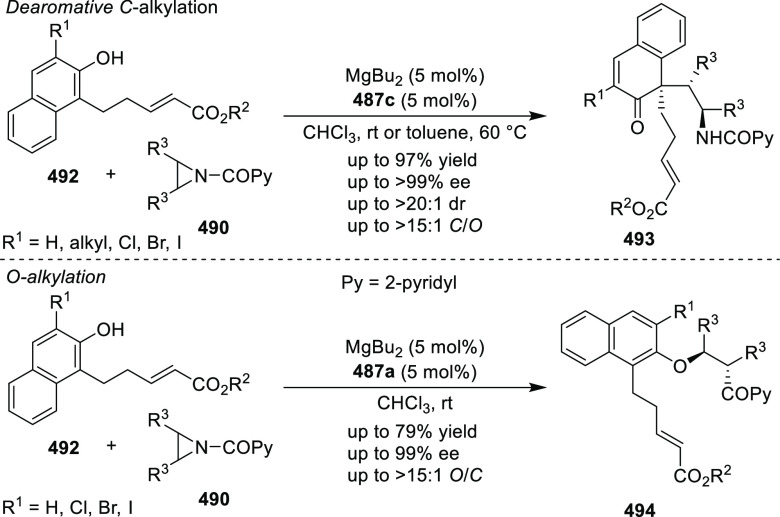
MgBu_2_/**487c**-Catalyzed Asymmetric Dearomatization and MgBu_2_/**487a**-Catalyzed Asymmetric *O*-Alkylation of Naphthols **494**

Wang further developed Box–OH/Mg-mediated transformations of naphthols with aziridines, describing two distinct processes for the dearomatization or *O*-alkylation of naphthols **492**. The *i*Pr-**487c**/MgBu_2_-catalyzed reaction of **492** with aziridines **490** gives access to the dearomatized products **493** in up to 97% yield, with up to >20:1 dr and >99% *ee* (Scheme 156). Conducting the reaction of *C*3-H and *C*3-halogenated naphthols **492** in the presence of Ph-**487a** (in place of **487c**) gives access to the *O*-alkylated products **494** in up to 79% yield and with up to 99% *ee*. So far there is no concrete explanation for the remarkable chemoselectivity observed in these processes. The reaction is certainly substrate dependent as the nature of R^1^ has the largest effect on chemoselectivity. The ligand plays an important role, and the authors suggest this could be due to π—π or π—anion interactions in the transition state of the reaction with ligand Ph-**487a**. Finally, the presence of a long alkyl chain at *C*-1 is essential for *O*-alkylation to occur, and the nature of this alkyl chain has a slight effect over the *C*/*O* ratio (the catenulate ester appears to be optimal).^178^

**Scheme 157:**
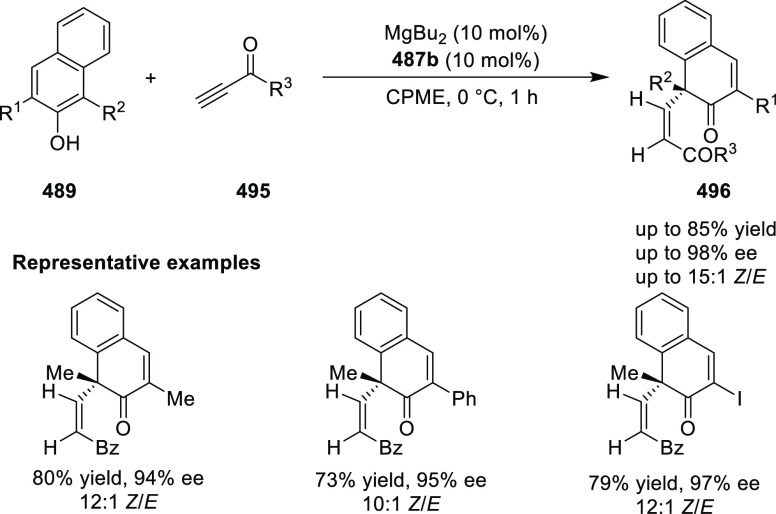
MgBu_2_/**487b**-Catalyzed Dearomative Conjugate Addition of Naphthols **489** to Alkynyl Ketones **495**

Wang has also reported the **487b**/Mg-catalyzed dearomative conjugate addition of naphthols **489** to alkynyl ketones **495** to give the corresponding alkenes **496** in up to 85% yield, with up to 15:1 *Z*/*E* selectivity and up to 98% *ee*, using cyclopentyl methyl ether (CPME) as the solvent (Scheme 157).^179^ Later, the scope of this reaction was extended to dialkyl acetylenedicarboxylates.^180^

**Scheme 158:**
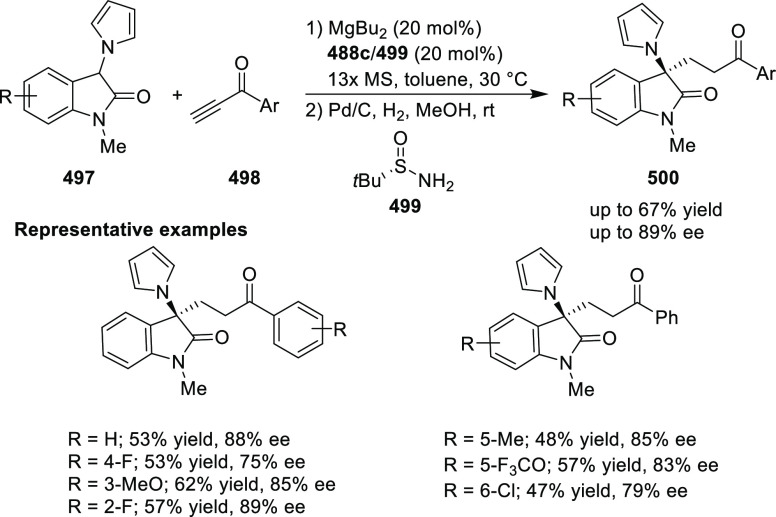
MgBu_2_/**488b**-Catalyzed Dearomative Conjugate Addition of Oxindoles **497** to Alkynyl Ketones **498**

Wang extended the Box–OH/Mg-catalyzed conjugate addition reaction to include *C*3-pyrrolyl-oxindoles **497** with alkynyl ketones **498**. By developing a **499**/**488c** bis-ligand system, a range of oxindoles **500**, bearing quaternary stereocenters were accessed via the Mg-catalyzed process, followed by reduction of the alkene with Pd/C and H_2_ in up to 67% yield and with up to 89% *ee* (Scheme 158). The reaction with sulfoxide **409** as a coligand only slightly improved the enantioselectivity of the process, compared to the reaction with just **488c**, under the exact same reaction conditions (without molecular sieves). The authors did not report the result of the reaction with just ligand **488c** under the final optimized conditions (including molecular sieves). The best *E*/*Z* selectivity achieved during optimization, without the reduction of the double bond, was 3:1.^181^

**Scheme 159:**
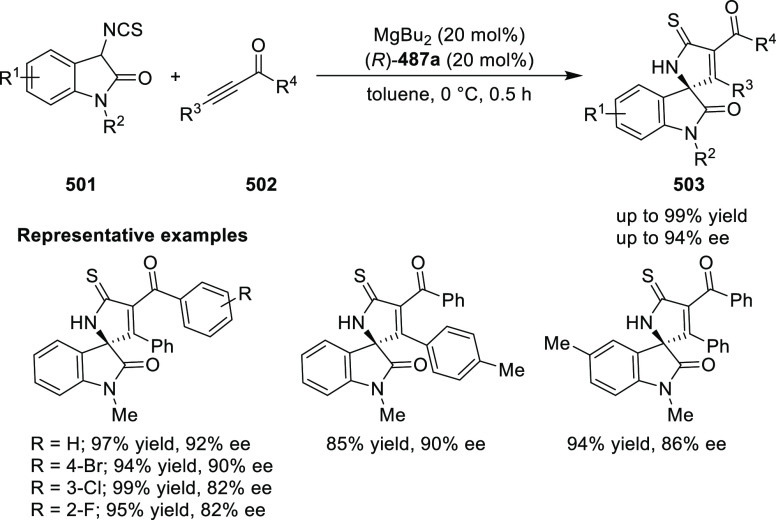
MgBu_2_/**487a**-Catalyzed Asymmetric [3 + 2] Cyclization of 3-Isothiocyanato Oxindoles **501** with Alkynyl Ketones **502**

Wang has also applied Box–OH/Mg-catalysts in the asymmetric [3 + 2] cyclization of 3-isothiocyanato oxindoles **501** with alkynyl ketones **502**. Utilizing (*R*)-Ph-**487a** as the chiral ligand, a range of polycyclic oxindoles **503** were synthesized in up to 99% yield and with up to 94% *ee* (Scheme 159).^182^

Overall, mono(oxazoline) *N*,*O*-ligands have been applied in a very limited range of asymmetric transformations in the past decade. In particular, they have been successfully applied in the asymmetric alkyl or aryl zinc addition to aldehydes, and in asymmetric Mg-catalyzed addition reactions to alkynyl ketones.

### 2.5 Miscellaneous Mono(oxazoline) Ligands

#### 2.5.1 Mono(oxazoline)-Sulfonamide Ligands

**Figure 12:**
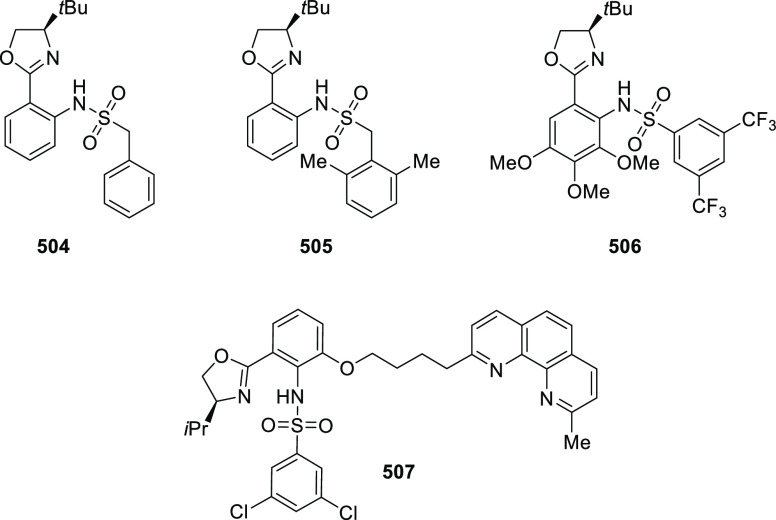
Chiral sulfonamide ligands.

Kishi and co-workers reported a library of chiral sulfonamide ligands (Figure 12) and they were successfully employed in the enantioselective Cr-catalyzed allylation, propargylation and vinylation of aldehydes, a reaction famously known as the Nozaki-Hiyama-Kishi (NHK) reaction.

**Scheme 160:**
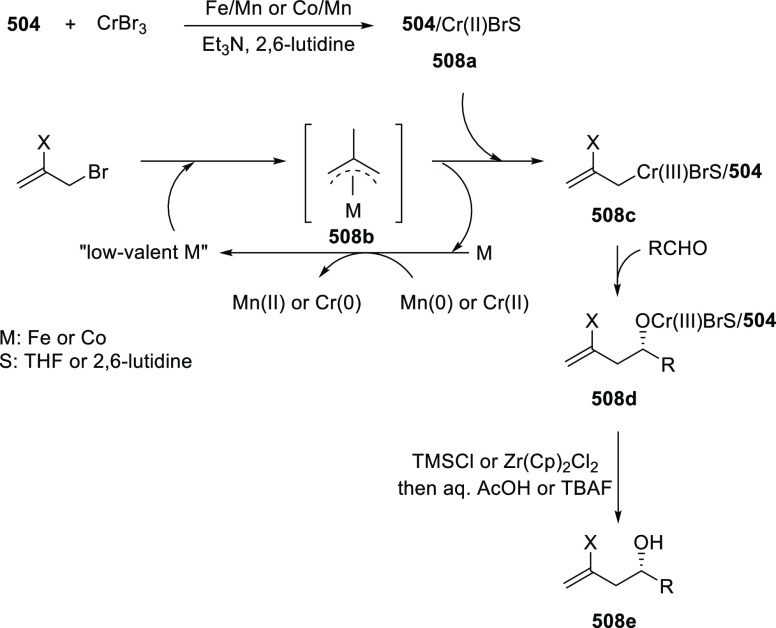
Proposed Mechanism for Cr-Catalyzed Allylation of Aldehydes

In 2004, Kishi developed a novel Cr-catalyzed enantioselective allylation of aldehydes using chiral sulfonamide ligands **504**–**507** (Scheme 160).^183^ A Cr-**504**–**507** complex generated from a sulfonamide **504**–**507** and CrCl_3_/CrBr_3_ in the presence of Et_3_N and Mn as a reducing agent of Cr and TMSCl or Zr(Cp)_2_Cl_2_ as a Cr alkoxide dissociating reagent. TMSCl and Zr(Cp)_2_Cl_2_ reagents upon hydrolysis breaks complex **508d** to alcohol **508e**. Interestingly, the addition of 2,6-lutidine was found to improve asymmetric inductions significantly by acting as an acid scavenger. Low valent Co phthalocyanine (CoPc) or Fe(TMHD)_3_ (iron tris(2,2,6,6-tetramethyl-3,5-heptanedione)) were used as an activator since they facilitate radical formation from allyl halides. The proposed mechanism for the Cr-catalyzed allylation of aldehydes is depicted in Scheme 160. The initial reaction between Cr(III) and chiral sulfonamide **504** in the presence of Et_3_N, 2,6-lutidine, Co/Fe and Mn generates Cr(II)-complex **508a**. Metalloallyl species (**508b**), formed from the allyl bromide and Co/Fe, undergoes transmetalation with Cr(II)-complex (**508a**) to generate the Cr(III)-allyl complex **508c**. This complex would undergo the addition to aldehydes through a six-membered transition state to form complex **508d** which further reacts with TMSCl or Zr(Cp)_2_Cl_2_ to form the alcohol product **508e**.

**Scheme 161:**
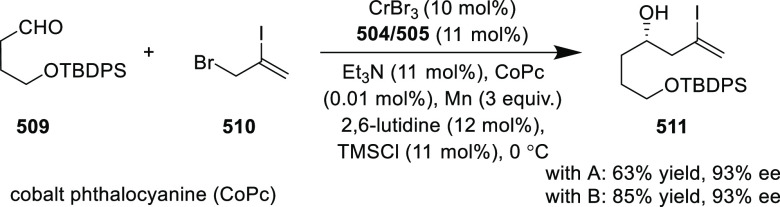
Synthesis of Chiral Alcohol **511**

The Cr-complex of ligand **504/505** was applied in the synthesis of chiral alcohol **511** (93% *ee*), a key building block in the halichondrin (a polyether macrolide) synthesis (Scheme 161).^184^ Ligand **505** exhibited exceptional crystallinity compared to ligand **504** and therefore it was easier to recover when used on a large scale reaction.

**Scheme 162:**
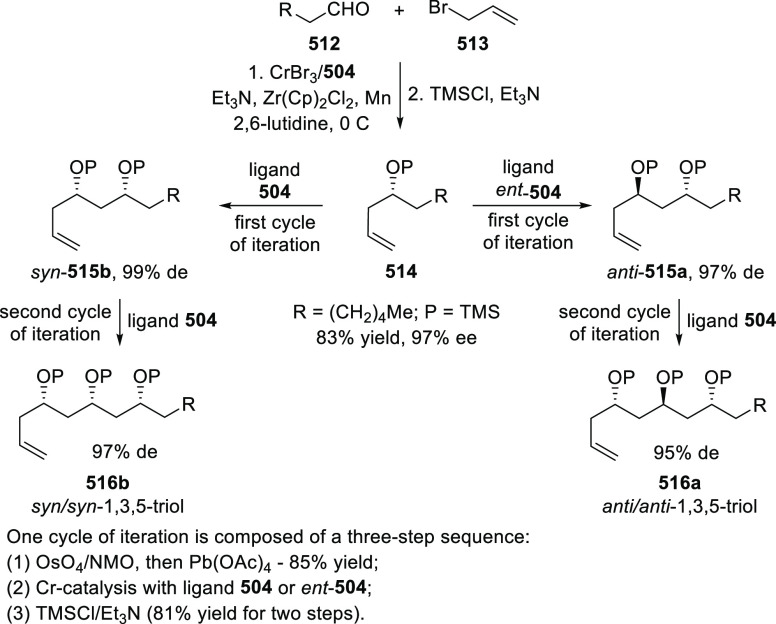
Iterative Asymmetric Allylation

Furthermore, iterative use of Cr(III)/**504**-catalyzed asymmetric allylation of aldehyde **512** provided an easy access to stereocontrolled 1,3-polyols, *syn*/*syn*- and *anti*/*anti*-1,3,5-triols (**516a**–**b**) (Scheme 162).^185^ One iteration cycle is composed of a three-step sequence: (i) oxidative cleavage of the olefin to form an aldehyde; (ii) catalytic asymmetric allylation; and (iii) protection of the resultant alcohol.

**Scheme 163:**
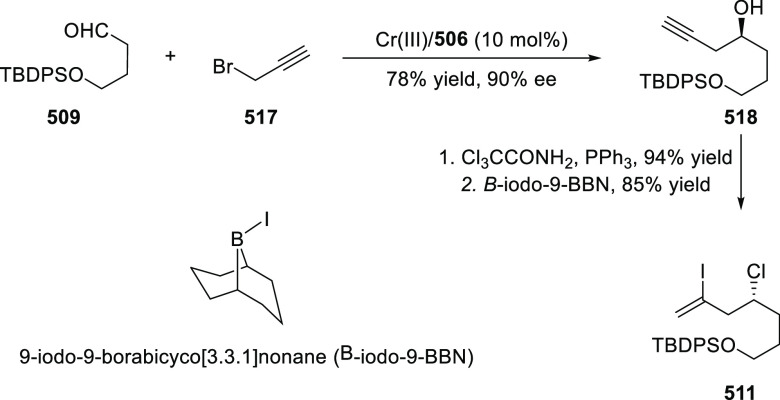
Asymmetric Propargylation of Aldehyde

In 2009, an asymmetric propargylation of aldehydes was developed by using Cr(III) bromide and (*R*)-sulfonamide **506** (Scheme 163).^186^ An optically pure homopropargyl alcohol **518** was obtained in 78% yield with 90% *ee*. Alcohol **518** was further converted to **511**, a building block of halichondrins and E7389.

Cr catalysts derived from different chiral sulfonamides were effectively utilized by Kishi to couple various aldehydes with allyl and vinyl halides to construct diverse C–C bonds during their halichondrin synthesis.^187−189^

**Scheme 164:**
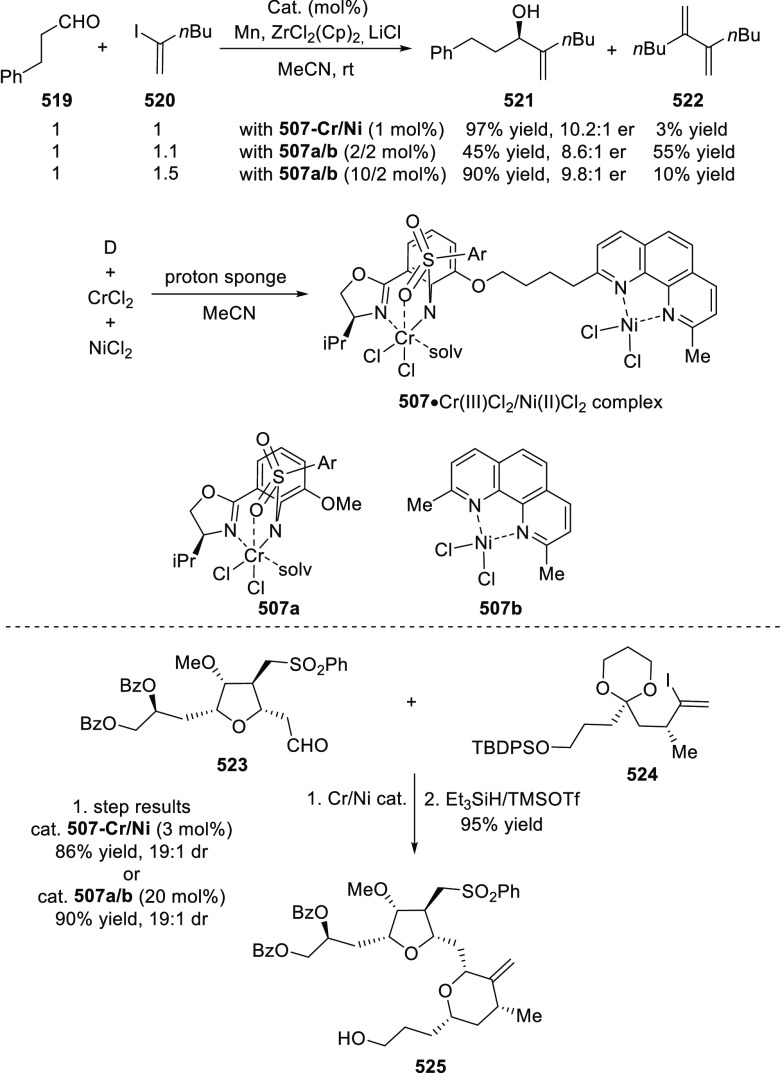
Asymmetric Vinylation of Aldehydes

In 2009, Kishi designed tethered ligand **507** which then was used to prepare heterobimetallic catalysts with Cr and Ni coordinated to the sulfonamide and phenanthroline moieties, respectively. The Ni/Cr heterobimetallic catalysts performed exceptionally well in the catalytic asymmetric vinylation of aldehydes (**519**) (Scheme 164).^190^ The catalyst highlights include low catalytic loading (1 mol%), the formation of a negligible amount of dimer **522**, a byproduct formed through the alkenyl Ni species, use of a 1:1 molar ratio of coupling partners aldehyde **519** and vinyl iodide **520**, the asymmetric induction was similar to that obtained in the coupling with the Cr/**507a** and Ni/**507b**.

The application of this new catalyst was again demonstrated in C–C bond-forming reactions from the synthesis of halichondrin/E7389. The coupling between polyfunctional aldehyde **523** (1.0 equiv) and vinyl iodide **524** (1.2 equiv) in the presence of Cr/Ni catalyst **507** (3 mol%) furnished the desired allylic alcohol in 86% yield with a 19:1 dr whereas the Cr-**507a**/Ni-**507b** catalyst (20 mol%) delivered allylic alcohol **525** in 90% yield with a 19:1 dr.

Overall, chiral monooxazoline sulfonamide ligands have found major success in Cr- and Ni-catalyzed allylation, vinylation, and propagylation of aldehydes. The major challenge remains the extension of this methodology to ketones.

#### 2.5.2 Tridentate Mono(oxazoline) Ligands

This section summarizes the progress made in the design and application of tridentate mono(oxazoline) ligands in metal-catalyzed asymmetric catalysis.

**Figure 13:**
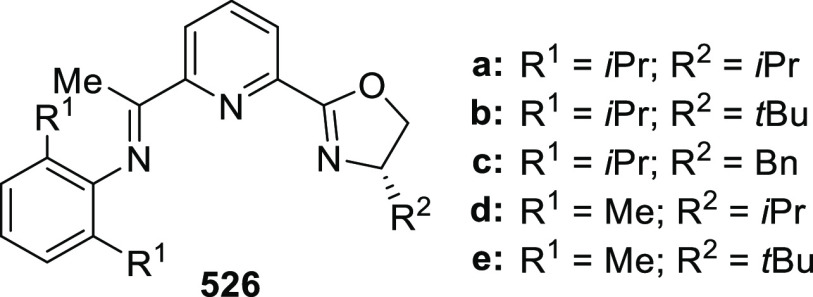
Tridentate iminopyridine oxazoline (IPO) ligands.

A variety of tridentate iminopyridine oxazoline (IPO) ligands (**526**) (Figure 13) have been applied in asymmetric catalytic transformations.

**Scheme 165:**
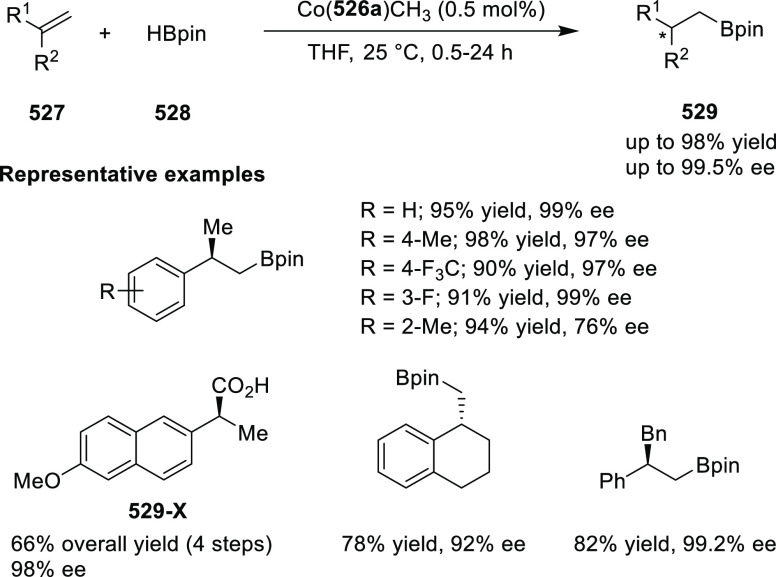
Asymmetric Co-Catalyzed Hydroboration of 1,1-Disubstituted Aryl Alkenes

Huang described the synthesis of novel IPO ligands **526a**–**c** and applied them in the asymmetric Co-catalyzed hydroboration of 1,1-disubstituted aryl alkenes **527**. Following optimization, a range of alkenes **527** were successfully subjected to the hydroboration catalyzed by the Co(**526a**) CH_3_ complex to give the enantioenriched products **529** in high yields up to 98% and with excellent enantioselectivities up to 99.5% *ee* (Scheme 165). This methodology was applied to the enantioselective synthesis of Naproxen **529-X** in four synthetic steps, in 66% overall yield and with 98% *ee*.^191^

**Scheme 166:**
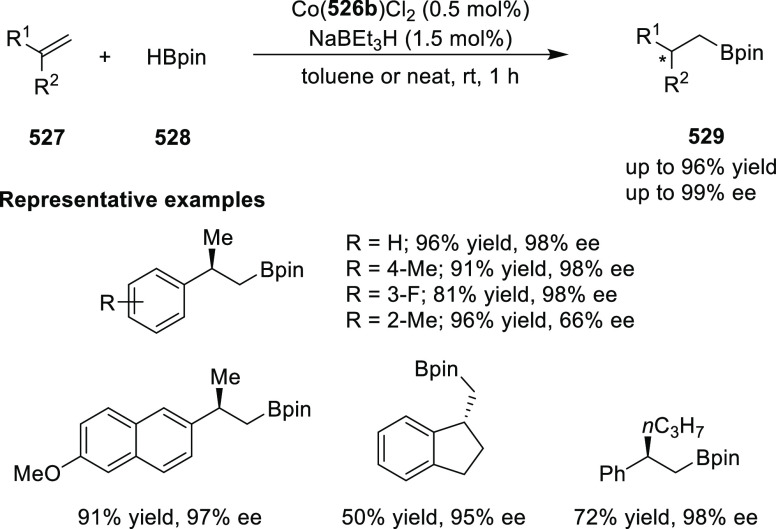
Hydroboration of 1,1-Disubstituted Aryl Alkenes

Lu independently reported the application of the same IPO ligands in a very similar Co-catalyzed hydroboration of 1,1-disubstituted aryl alkenes **527** (Scheme 166). In this case, the optimized conditions for the hydroboration utilized Co(**526b**)Cl_2_ as the catalyst to give the enantioenriched products **529** in up to 96% yield and with up to 99% *ee*, with very similar results to Huang. Interestingly, this catalyst was tested by Huang to give the model product in 91% *ee*, compared to Lu’s 98% *ee*. Both groups used similar reaction conditions during their optimization studies (including the addition of NaBEt_3_H); however, Lu’s reaction conditions were much more concentrated (5.0 M in toluene, or neat) than Huang’s (0.25 M in THF), leading to different levels of enantioselectivity. Huang excluded NaBEt_3_H from the reaction mixture and used the Co-CH_3_ complex (in place of the CoCl_2_ complex) as the catalyst in their final optimized reaction conditions.^192^ Lu subsequently described a Bn-IPO Fe(**526c**)Cl_2_-catalyzed hydroboration of 1,1,-disubstiuted alkenes with very similar results, giving the enantioenriched products in up to 97% *ee*.^193^

**Scheme 167:**
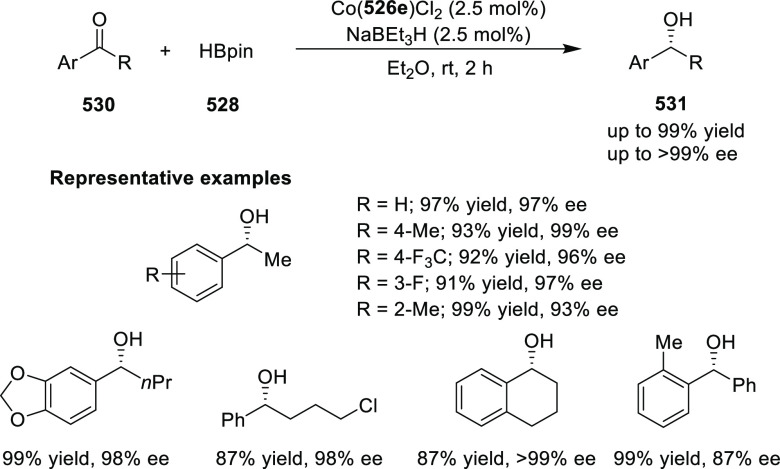
Asymmetric Hydroboration of Aryl Ketones

Lu has also applied IPO ligands in the Co(**526e**)Cl_2_-catalyzed hydroboration of aryl ketones **530** to give the enantioenriched alcohols **531** in moderate to high yields up to 99% and with excellent enantioselectivities up to >99% *ee* (Scheme 167). The reaction also works with the IPO-FeCl_2_ complexes, but with lower levels of enantioselectivity.^194^

**Scheme 168:**
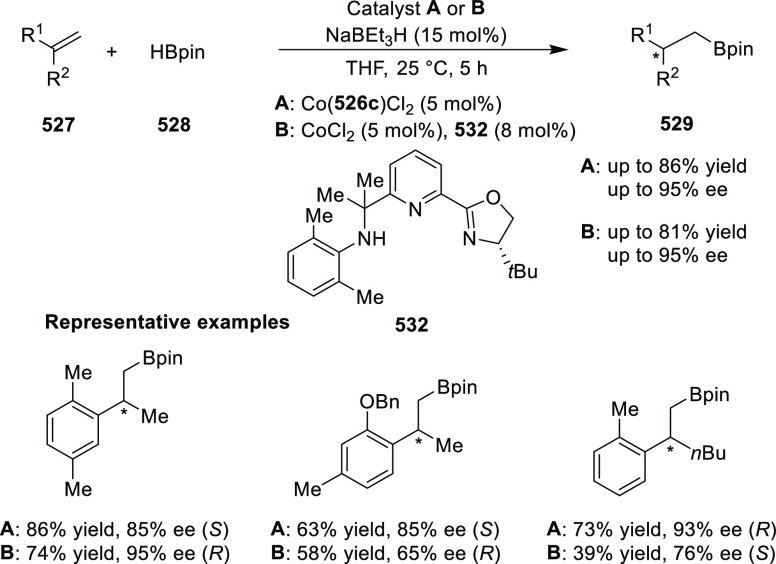
Asymmetric Hydroboration of 1,1-Disubstituted Aryl Alkenes

Lu has further expanded on the Co-catalyzed asymmetric hydroboration of 1,1-disubstituted aryl alkenes **527**, reporting a dual stereocontrolled reaction to give access to both enantiomers of the products **529**. Utilizing Bn-IPO ligand **526c**, the enantioenriched products **529** were accessed in up to 86% yield and with up to 95% *ee* (Scheme 168). Under the same reaction conditions, but with novel aminopyridine oxazoline (APO) ligand **532** (8 mol%) and CoCl_2_ (5 mol%) as the catalytic system, the opposite enantiomers of the products **529** were obtained in up to 81% yield and with up to 95% *ee*. While the IPO system gave higher yields across the board, both the IPO and APO systems gave better levels of enantioselectivity with different substrates. Preliminary deuterium labeling experiments suggested that the reaction with the IPO ligand operated under a different mechanism than the APO ligand, and this could explain the stereochemical outcomes of the two reactions.^195^

**Scheme 169:**
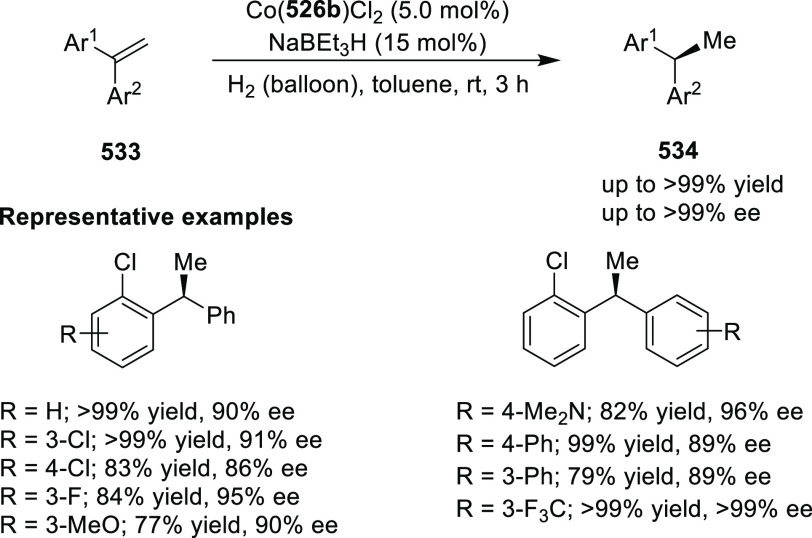
Asymmetric Hydrogenation of 1,1-Diarylethenes

Co(**526b**)Cl_2_ has also been used by Lu in the asymmetric hydrogenation of 1,1-diarylethenes **533**. A range of enantioenriched diarylethane derivatives **534** were accessed in up to >99% yield and with up to >99% *ee* (Scheme 169). In all examples, one of the two aryl groups of the substrates **533** had an *ortho*-substituent.^196^

**Scheme 170:**
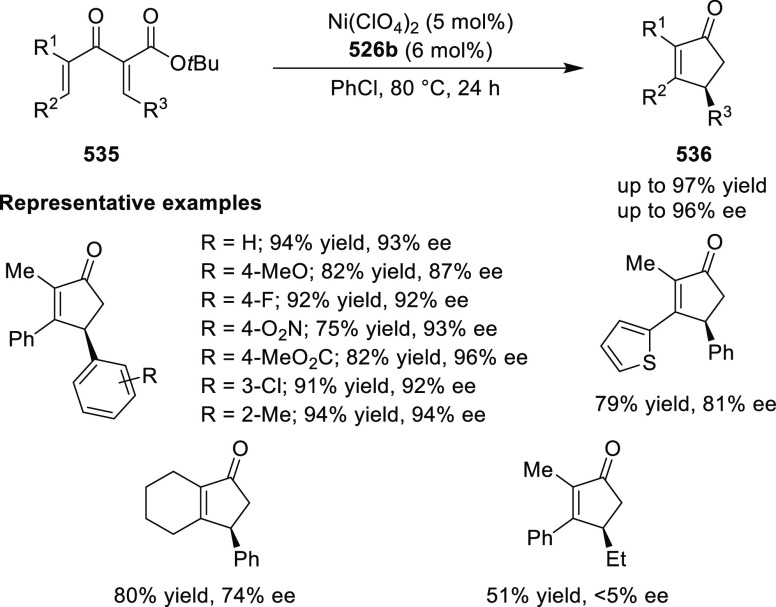
Ni-Catalyzed Asymmetric Nazarov Cyclization/Decarboxylation

Lu has also reported a sequential Ni-catalyzed asymmetric Nazarov cyclization/decarboxylation of divinylketones **535** bearing a *t*Bu-ester group to give enantioenriched cyclopentanones **536** in up to 97% yield and with up to 96% *ee* (Scheme 170). The reaction tolerated a range of electronically and sterically different aryl groups at R^2^ and R^3^, although aliphatic groups at R^3^ were not tolerated.^197^

**Scheme 171:**
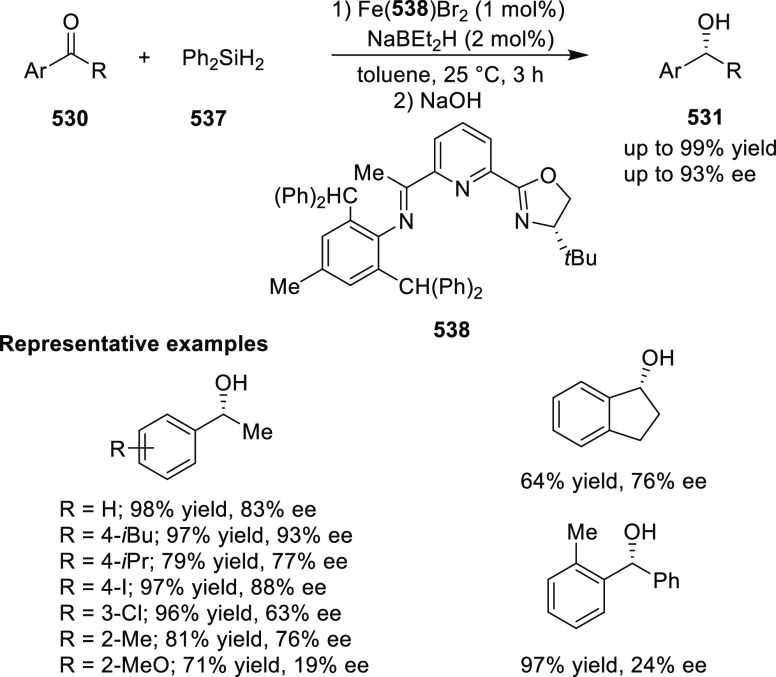
Fe-Catalyzed Hydrosilylation of Aryl Ketones

Huang has reported a highly hindered IPO ligand **538** for use in the Fe-catalyzed hydrosilylation of aryl ketones **530** (Scheme 171). The hindered Fe(**538**)Br_2_ catalyst showed high activity for accessing a range of chiral secondary alcohols **531** in up to 99% yield and with moderate to high enantioselectivities up to 93% *ee*.^198^

**Figure 14:**
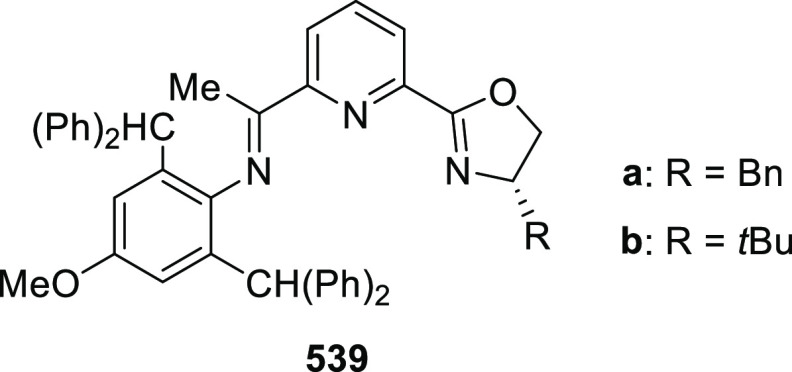
IPO ligands **539a**–**b**.

Lu later described derivatives of IPO ligands **538**, **539a**, and **539b** (Figure 14), for the Co- and Fe-catalyzed hydrosilylation of alkenes, respectively.

**Scheme 172:**
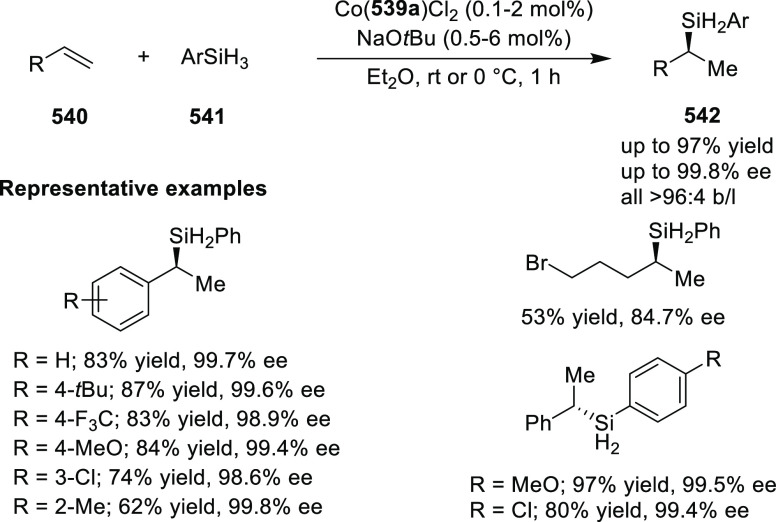
Co-Catalyzed Hydrosilylation of Alkenes with Phenylsilane

First, Lu developed the Co-catalyzed hydrosilylation of alkenes with phenylsilane **541**. During optimization, they found that the sterically hindered ligand **539a** performed better than the less hindered ligands **526a** and **526d**, giving the model product in 98.5% *ee* under those conditions, compared to 72% *ee* (**526a**) and 88% *ee* (**526d**). Under optimized conditions with Bn-IPO **539a** as the chiral ligand, a range of silylated products **542** were synthesized in up to 97% yield, with >96:4 b/l and with excellent levels of enantioselectivities up to 99.8% *ee* (Scheme 172). Styrenes were hydrosilylated with excellent levels of enantioselectivity while aliphatic alkenes gave <88% *ee*.^199^

**Scheme 173:**
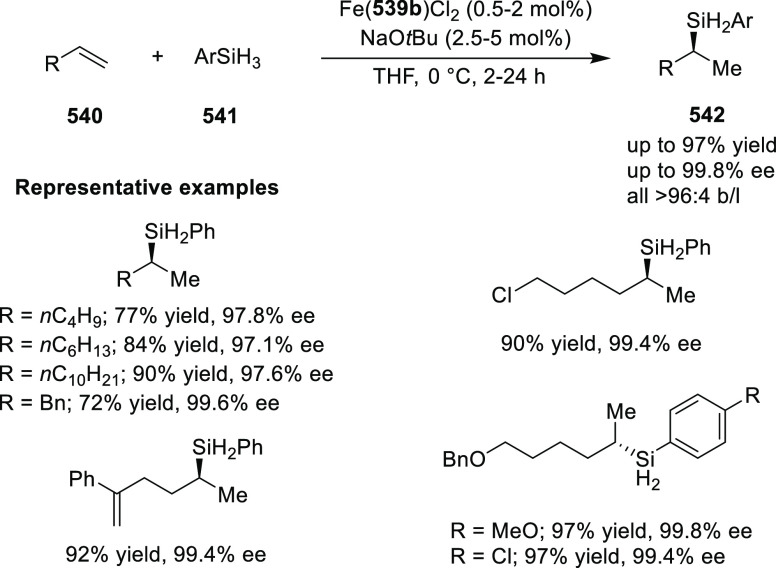
Fe-Catalyzed Hydrosilylation of Aliphatic Alkenes

Lu subsequently developed the Fe(**539b**)Cl_2_-catalyzed hydrosilylation of aliphatic alkenes **540** under similar reaction conditions to the previous Co-catalyzed report (ligand **539a** was not reported). A range of silylated products were isolated in up to 97% yield, with >96:4 b/l and with excellent levels of enantioselectivities up to 99.8% *ee* (Scheme 173). These reports offer complementary systems, with the same family of chiral ligands, for the enantioselective hydrosilylation of styrenes and aliphatic alkenes in similarly high stereoselectivities.^200^

**Figure 15:**
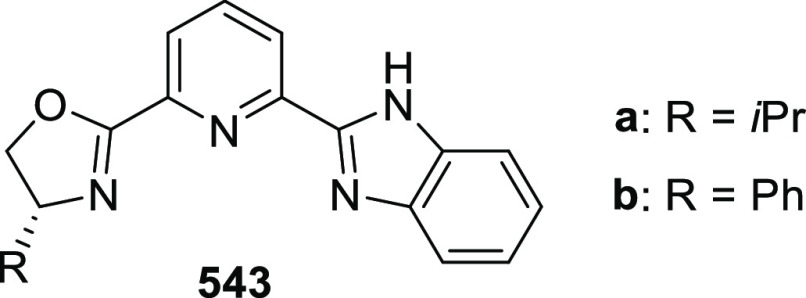
*C*_1_-symmetric benzimidazole-pyridyl oxazoline ligands.

Other unsymmetrical tridentate pyridine-oxazoline-containing ligands have been applied in asymmetric catalytic transformations. Yu has described the design of *C*_1_-symmetric benzimidazole-pyridyl oxazoline ligands **543a** and **543b** (Figure 15).

**Scheme 174:**
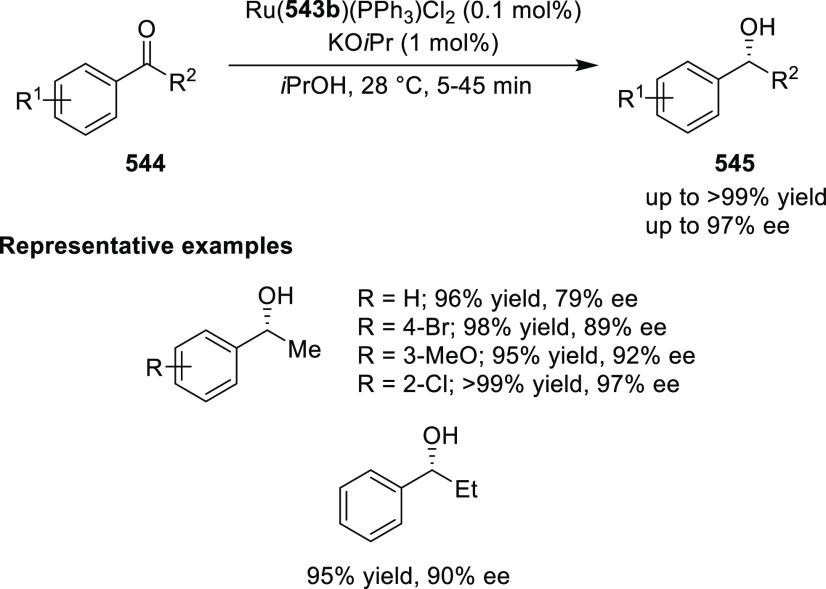
Asymmetric Transfer Hydrogenation of Ketones

Yu observed that the Ru-complex Ru(**543b**)(PPh_3_)Cl_2_ was a highly active catalyst for the asymmetric transfer hydrogenation of ketones **544**, giving the corresponding chiral alcohols in up to >99% yield and with up to 97% *ee* (Scheme 174).^201^

**Figure 16:**
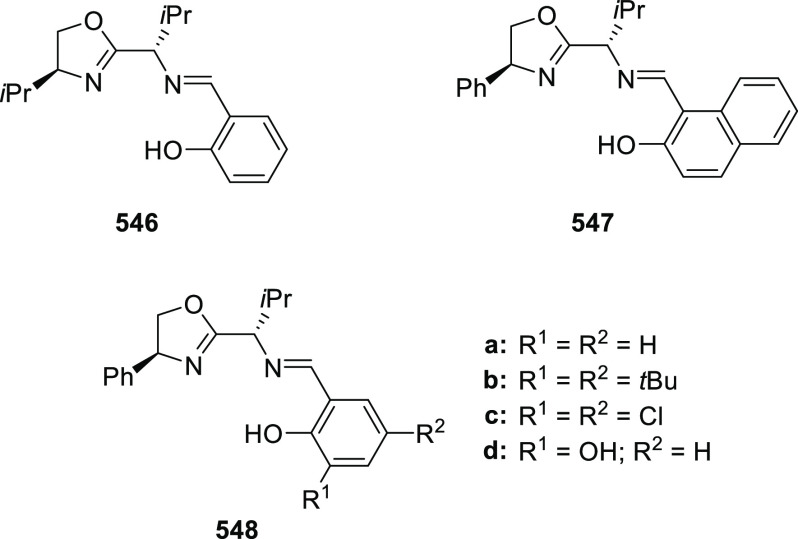
Novel Schiff base oxazoline-containing ligands.

Wang and Shi have reported the synthesis of novel Schiff base oxazoline-containing ligands **546**–**548** (Figure 16) and their applications in the asymmetric α-chlorination of β-keto esters. The denticity of the ligands was not determined, but the *ortho*-hydroxy groups were observed to be essential for generating products with appreciable levels of enantioselectivity.

The results of a screen of ligands **546**–**548** in the enantioselective Cu-catalyzed α-chlorination of β-keto ester **549** are summarized in Table 3. The reaction with bulkier *i*Pr-oxazoline ligand **546**, which bears no substitution on the *ortho*-phenol, gave the chlorinated product **550** with only 47% *ee* (entry 1). The reaction with Ph-oxazoline *ortho*-naphthol **547** gave **550** in 99% yield and with a much improved 78% *ee*. A screen of other Ph-oxazoline ligands **548a**-**d** showed that ligand **548a**, with no substitution on the *ortho*-phenol, gave **550** in 99% yield and with 78% *ee* (entries 3–6). A temperature screen was conducted in the reaction with ligand **548a** (entries 7–9). The reaction conducted at 0 °C gave **550** with the highest level of enantioselectivity of 83% *ee* (entry 7).^202^

**Table 3:**
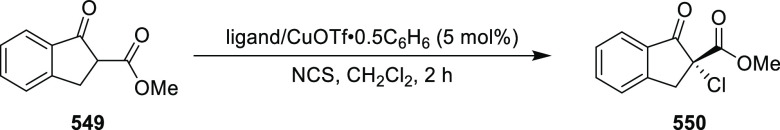
Enantioselective Cu-Catalyzed α-Chlorination of β-Keto Ester

entry	ligand	temp (°C)	yield (%)	% *ee*
1	**546**	rt	99	47
2	**547**	rt	99	78
3	**548a**	rt	99	78
4	**548b**	rt	95	50
5	**548c**	rt	99	54
6	**548d**	rt	99	68
7	**548a**	0	99	83
8	**548a**	–20	99	72
9	**548a**	–78	99	6

#### 2.5.3 Mono(oxazoline) Carbene Ligands

In the past decade, limited progress has been made in the design and application of mixed oxazoline-carbene ligands in asymmetric catalysis.

**Figure 17:**
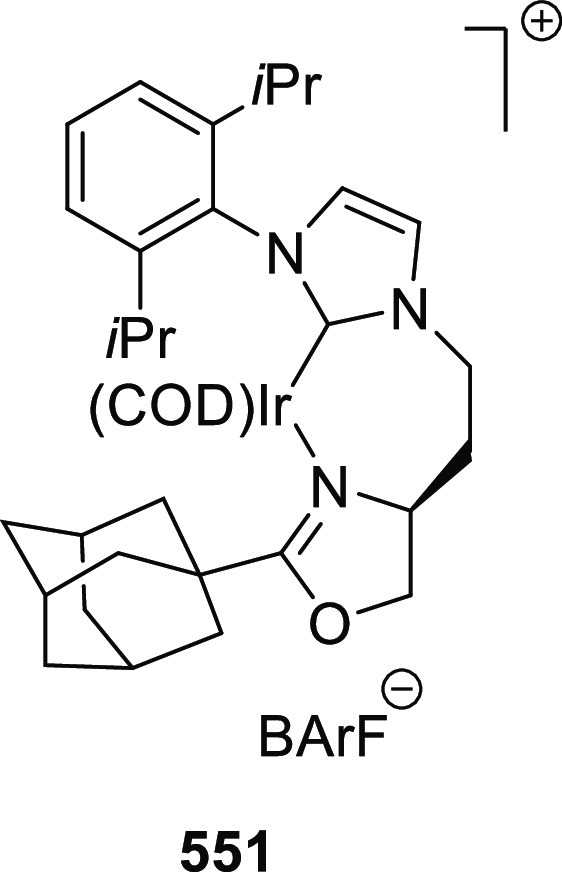
Mixed oxazoline-carbene ligands.

Burgess has applied the Ir-complex **551** (Figure 17) in the asymmetric catalytic hydrogenation of vinyl ethers.

**Scheme 175:**
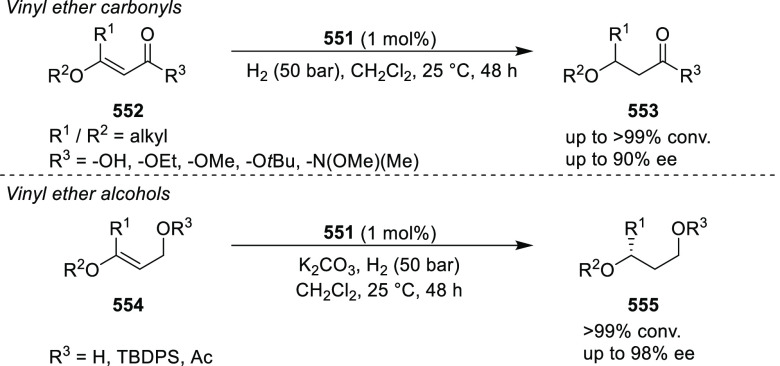
Asymmetric Hydrogenation of Vinyl Ether Alcohols

Under optimized conditions, vinyl ether esters **552** were successfully hydrogenated, under 50 bar of H_2_, to give the corresponding chiral esters **553** with up to >99% conversion and with mostly moderate to good enantioselectivities of up to 90% *ee* (Scheme 175). Ir-complex **551** was then applied in the asymmetric hydrogenation of vinyl ether alcohols with better results, under slightly altered reaction conditions. The chiral alcohols **555** were formed with >99% conversion and with very high enantioselectivities of up to 98% *ee*.^203^

**Figure 18:**
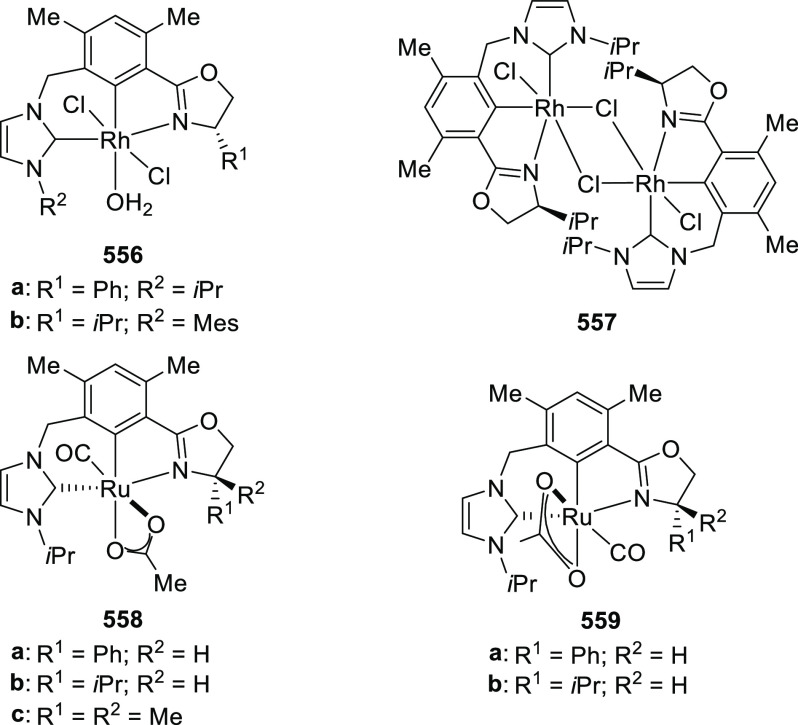
Mixed carbene-oxazoline Rh- and Ru-complexes.

Ito and Nishiyama reported a set of mixed carbene-oxazoline Rh- and Ru-complexes 556–559(Figure 18), which are analogous to PheBOX complexes (covered in section 2.5.4). The applications of the Rh-complexes in asymmetric conjugate reduction and the Ru-complexes in the asymmetric catalytic hydrogenation and asymmetric transfer hydrogenation of ketones, led to low or moderate enantioselectivities of the isolated products in all cases.

For example, the asymmetric transfer hydrogenation of 9-acetylanthracene was conducted with **558a**–**c** and **559b** as the catalysts (Table 4). Interestingly, the reactions with Ru-catalysts **558a**, **558b**, and **559b** selectively formed secondary alcohols **561a** and **561b** in which the ketone and part of the anthracene ring are reduced (entries 1–3). The reaction with **559b** gave the alcohol **561a** with the highest stereoselectivity of 60% *ee*. Achiral Ru-catalyst **558c** gave remarkable selectivity in the formation of **561a**. These results suggest that further development of these mixed-carbene-oxazoline systems could be useful for the selective reduction of extended aromatic-ring-systems.^204^

**Table 4:**
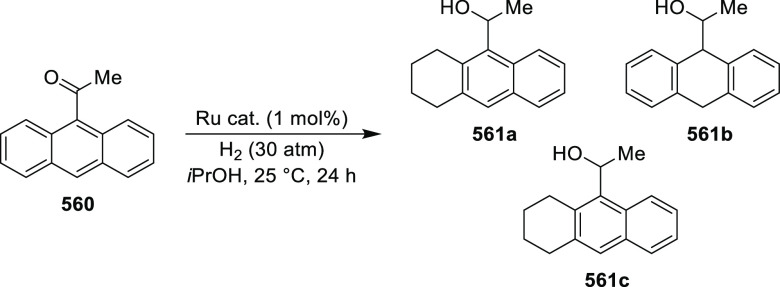
Asymmetric Transfer Hydrogenation of 9-Acetylanthracene

entry	cat	yield (%)	**561a**/**b**/**c**	% *ee* (**561a**)	% *ee* (**561b**)
1	**558a**	99	60:40:0	9 (*R*)	18 (*R*)
2	**558b**	99	51:49:0	31 (*R*)	5 (*R*)
3	**559b**	97	60:40:0	60 (*R*)	28 (*R*)
4	**558c**	90	93:<1:7		

**Figure 19:**
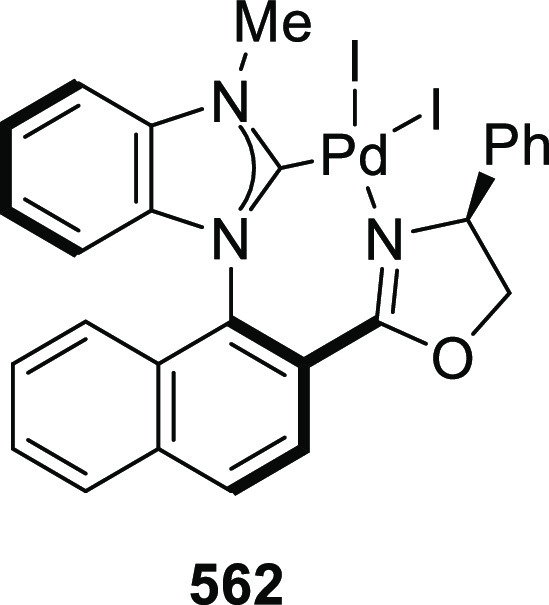
Axially chiral mixed carbene-oxazoline ligand.

Wang and Shi have applied Pd-complex **562** (Figure 19), bearing an axially chiral mixed carbene-oxazoline ligand, in an asymmetric allylic arylation for the kinetic resolution of Morita-Baylis-Hillman (MBH) adducts **563**.

**Scheme 176:**
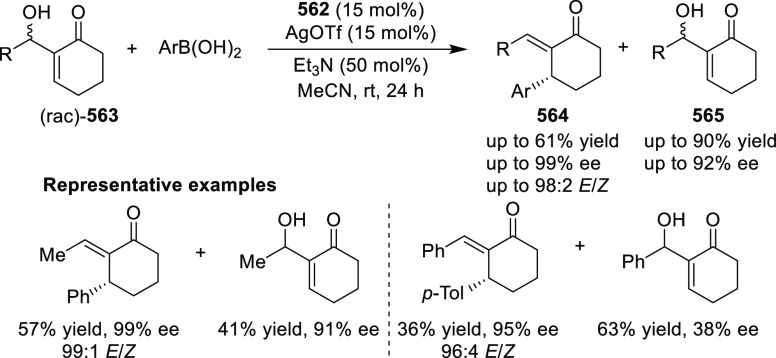
Kinetic Resolution of Morita–Baylis–Hillman (MBH) Adducts

Reacting racemic MBH adducts **563** with aryl boronic acids in the presence of **562**, the corresponding arylated products **564** were isolated in up to 61% yield, with up to 99:1 *E*/*Z* selectivity and >99% *ee* (Scheme 176). The enantioenriched alcohols **565** were recovered in up to 37% yield and with the best enantioselectivity of 92% *ee*. This process is clearly a better method for synthesizing the arylated products **564** enantioselectively than it is for resolving the MBH adducts **563**.^205^

**Figure 20:**
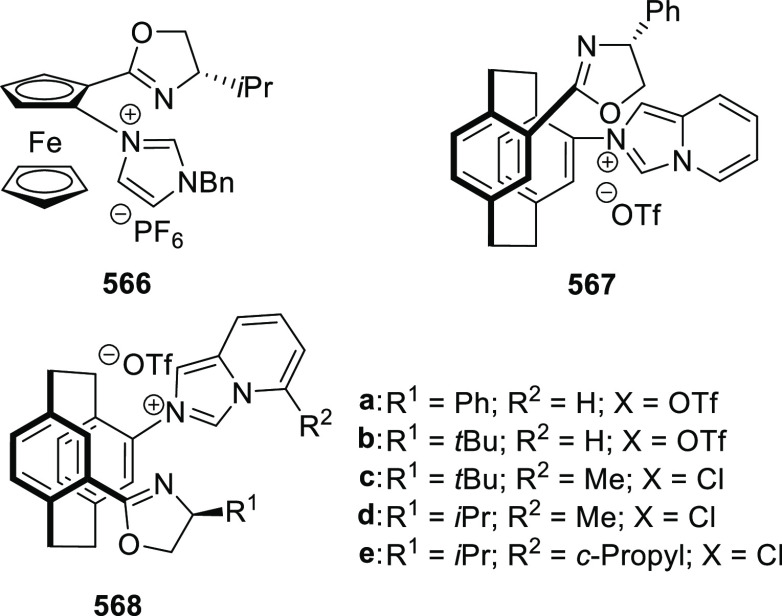
Novel planar chiral mixed carbene-oxazoline ligands.

Ma and Jiang have independently developed novel planar chiral mixed carbene-oxazoline ligands for use in asymmetric catalysis (Figure 20). Jiang has reported the ferrocene-based ligand precursor **566**, while Ma has reported the cyclophane-based ligand precursors like **567** and **568**. Jiang has applied the Rh^I^(**566**)(cod) complex in the asymmetric hydrosilylation of ketones, obtaining only low to moderate enantioselectivities of 39–56% *ee*.^206^

**Scheme 177:**
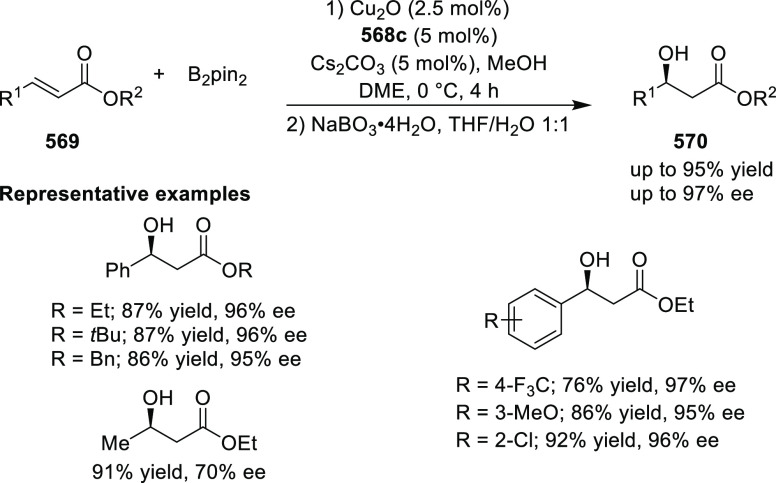
Cu-Catalyzed Conjugate Borylation of α,β-Unsaturated Esters

In Ma’s initial report, the cyclophane-containing ligands were applied in the Cu-catalyzed conjugate borylation of α,β-unsaturated ketones, with the products isolated with up to 84% *ee* when ligand precursor *t*Bu-**568b** was used.^207^ The pseudo-*ortho*-cyclophanes **568** generally perform better than the pseudo-*geminal*-cyclophanes, like **567** in Ma’s reports. Ma extended the substrates from α,β-unsaturated ketones to α,β-unsaturated esters in the conjugate borylation of **569**. Under optimized conditions, a range of esters **569** were borylated with B_2_pin_2_, utilizing Cu_2_O/*t*Bu-**568c** as the catalytic system, followed by treatment with sodium perborate, to yield the corresponding chiral alcohols **570**. The enantioenriched products **570** were generated in up to 95% yield and with high enantioselectivities up to 97% *ee* (almost all examples >90% *ee*) (Scheme 177).^208^

**Scheme 178:**
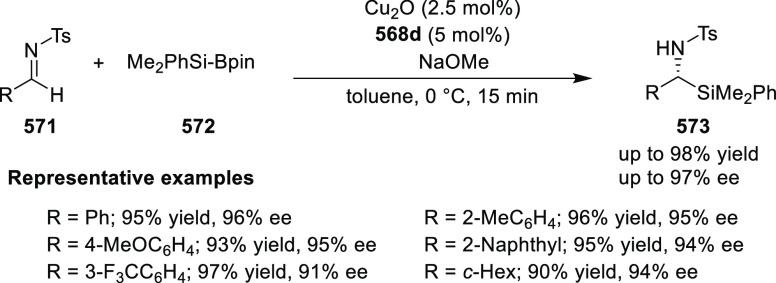
1,2-Silylation of *N*-Tosylaldimines

Ma has also developed a Cu_2_O/*i*Pr-**568d**-catalyzed 1,2-silylation of *N*-tosylaldimines **571**, generating the silylated amines **573** in up to 98% yield and with excellent enantioselectivities of up to 97% *ee* (Scheme 178).^209^

#### 2.5.4 Ruthenium(II)/Phenyloxazoline (Ru(II)-Pheox)

**Figure 21:**
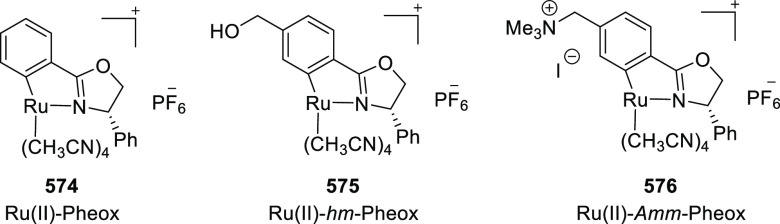
Chiral Ru(II)/phenyloxazoline (Ru(II)-Pheox) complexes.

Iwasaa designed and synthesized novel chiral Ru(II)/phenyloxazoline (Ru(II)-Pheox) complexes (Figure 21).^210^ This catalyst system showed excellent reactivity and enantioselectivity in inter- and intramolecular cyclopropanation reactions.

**Scheme 179:**
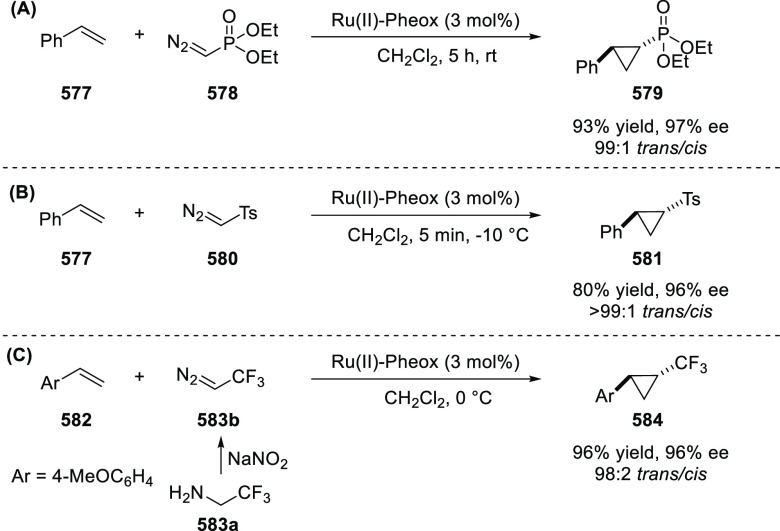
Highly Stereoselective Cyclopropanation of Alkenes with Various Diazo Precursors

Iwasaa employed Ru(II)-Pheox complex (**574**) for the highly stereoselective cyclopropanation of alkenes (**577**) with various diazo precursors (**578**/**580**/**583a**) (Scheme 179A–C).^211−213^ The asymmetric cyclopropanation between diethyl diazomethylphosphonate (**578**) and a series of alkenes including styrene derivatives, α,β-unsaturated esters, ketones, and amides was carried out to provide the corresponding cyclopropylphosphonate products (**579**) in high yields and with excellent diastereoselectivity (up to 99:1 dr) and enantioselectivity (up to 97% *ee*) (Scheme 179A).^211^ Similarly, diazosulfones (**580**) reacted with various alkenes including vinyl ethers, vinyl amines, and vinyl carbamates to furnish chiral cyclopropyl sulfones (**581**) in high yields (up to 80%) with excellent *trans*-selectivity and enantioselectivity (up to 96% *ee*) (Scheme 179B).^212^ An asymmetric synthesis of various trifluoromethyl cyclopropanes (**584**) was achieved in high yields with excellent diastereoselectivity (up to 98:2 dr) and enantioselectivity (up to 96% *ee*) by reacting *in situ* generated CF_3_CHN_2_ (**583b**) with a range of alkenes including vinyl ferrocene, vinyl ethers, vinyl amines, vinyl carbamates, and dienes (Scheme 179 C).^213^

**Scheme 180:**
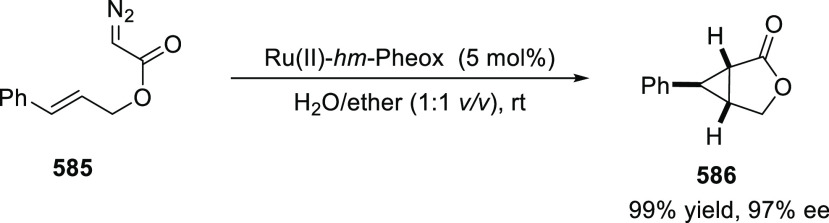
Enantioselective Intramolecular Cyclopropanation

In 2012, Iwasaa described the highly enantioselective intramolecular cyclopropanation of *trans*-allylic diazoacetates (**585**) and alkenyl diazoketones using a water-soluble Ru(II)/hydroxymethyl (phenyl)oxazoline catalyst, Ru(II)-*hm*-Pheox (**575**) (Scheme 180).^214^ The polar protic functionality of the Ru catalyst was vital to give rise to high yields, due to its ability to form strong hydrogen bonds with water. The catalyst could be reused at least five times without substantial decline in reactivity or enantioselectivity.

**Scheme 181:**
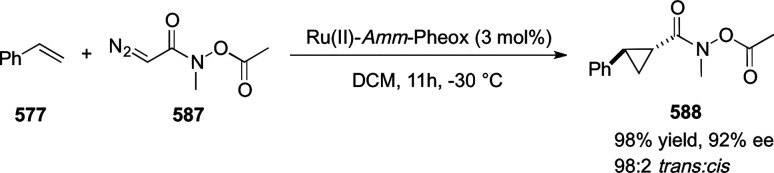
Enantioselective Intermolecular Cyclopropanation of Alkenes with Diazo Weinreb Amides

Later in 2016, chiral Ru(II)–*Amm*–Pheox complex (**576**) containing an internal quaternary ammonium unit was first employed in the stereoselective intermolecular cyclopropanation of diazo Weinreb amides (**587**) with alkenes (**577**) to furnish the corresponding chiral cyclopropyl Weinreb amides (**588**) in high yields (up to 98%) with excellent diastereoselectivities (up to 98:2 dr) and enantioselectivities (up to 92% *ee*) (Scheme 181).^215^ Additionally, the use of acetoxy-functionalized diazoacetamide (AMD) as the carbene source was found to be crucial for the high *trans*-selectivity of the cyclopropanation.

**Scheme 182:**
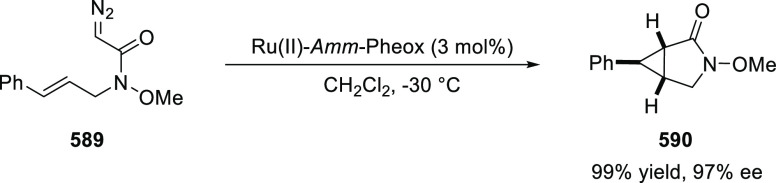
Enantioselective Intramolecular Cyclopropanation of *trans*-Allylic Diazo Weinreb Amide Derivatives

In subsequent studies, the intramolecular stereoselective cyclopropanation of a series of *trans*-allylic diazo Weinreb amide derivatives (**589**) was developed using the chiral Ru(II)-*Amm*-Pheox catalyst (**576**) to afford the corresponding chiral bicyclic cyclopropyl products (**590**) in excellent yields (up to 99%) with excellent enantioselectivity (up to 97% *ee*) (Scheme 182).^216^

Overall, chiral Ru(II)-Pheox complexes have found their applications in inter and intramolecular asymmetric cyclopropanation reactions between various alkenes with diazo derivatives.

#### 2.5.5 Cobalt Oxazoline Palladacycle (COP)

**Figure 22:**
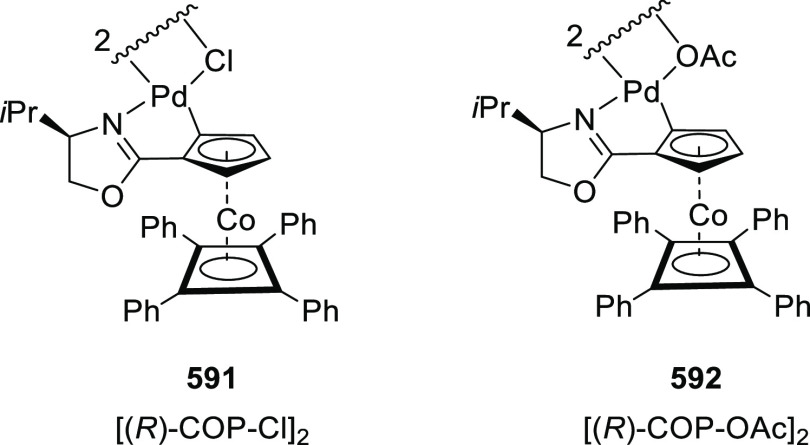
COP catalysts.

The enantioselective chiral cobalt oxazoline palladacycle (COP) catalyst (**591**/**592**) (Figure 22) was first reported by Richards and Overman in 2003 for the enantioselective rearrangement of allylic *N*-arylimidates.^217^ A detailed review on Pd(II)-catalyzed enantioselective reactions using the COP catalyst was published by Overman in 2016.^218^

#### 2.5.6 Pentaphenylferrocene Oxazoline Palladacycle [PPFOP]

**Scheme 183:**
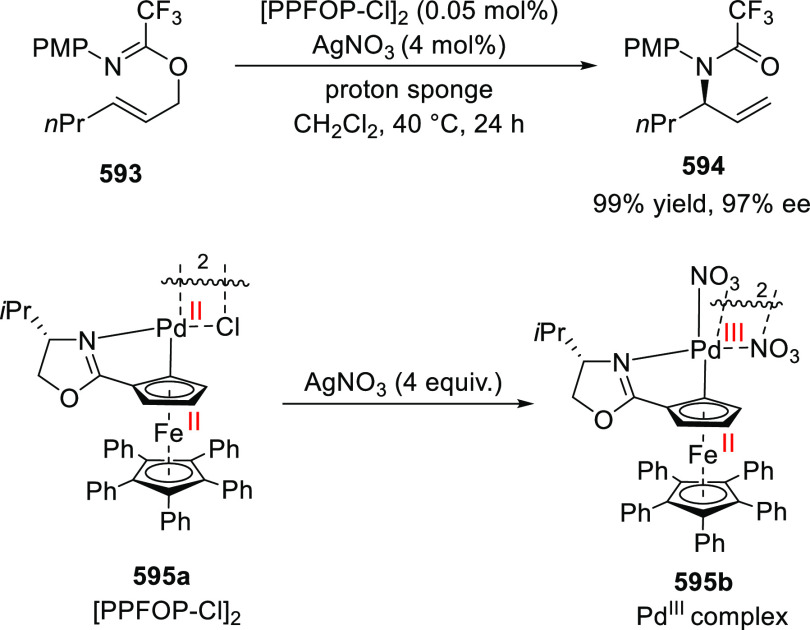
Novel Pd(III)-Catalyst for Asymmetric Rearrangement of Allylic Trifluoroacetimidates

Peters reported^219,220^ the novel Pd(III)-catalyst (**595b**) for the asymmetric rearrangement of allylic trifluoroacetimidates (**593**) (Scheme 183). Various spectroscopic and electrochemical methods demonstrated that the Pd-catalyst (**595b**) unexpectedly had an activated oxidized form of a Pd(III) center bound to a ferrocene core which remained unchanged (Fe(II)) during the oxidative addition. The dimeric paramagnetic ferrocene-derived palladacycle (**595b**) contains two Pd(III) centers without involving a Pd–Pd bond. The Pd(III)-catalyst was formed by activating the diamagnetic dimeric chloride-bridged pentaphenylferrocene oxazoline palladacycle precatalyst, [PPFOP-Cl]_2_(**595a**), by treatment with AgNO_3_ (4 equiv). Two equivalents of the AgNO_3_ were required for a Cl-counterion exchange while another 2 equiv oxidized the complex to deliver the dark red-brown paramagnetic species (**595b**). Pd-complex (**595b**) was found to be the most efficient enantioselective catalyst for the rearrangement of allylic trifluoroacetimidates in terms of catalyst TON, scope and enantioselectivity. Previously it was assumed that the reaction between [PPFOP-Cl]_2_ and AgNO_3_ resulted in the oxidation of Fe(II) to Fe(III) in a ferrocenium core which would decrease the electron density of the Pd(III) center thus generating a more Lewis acidic Pd(III) center.^219^

**Scheme 184:**
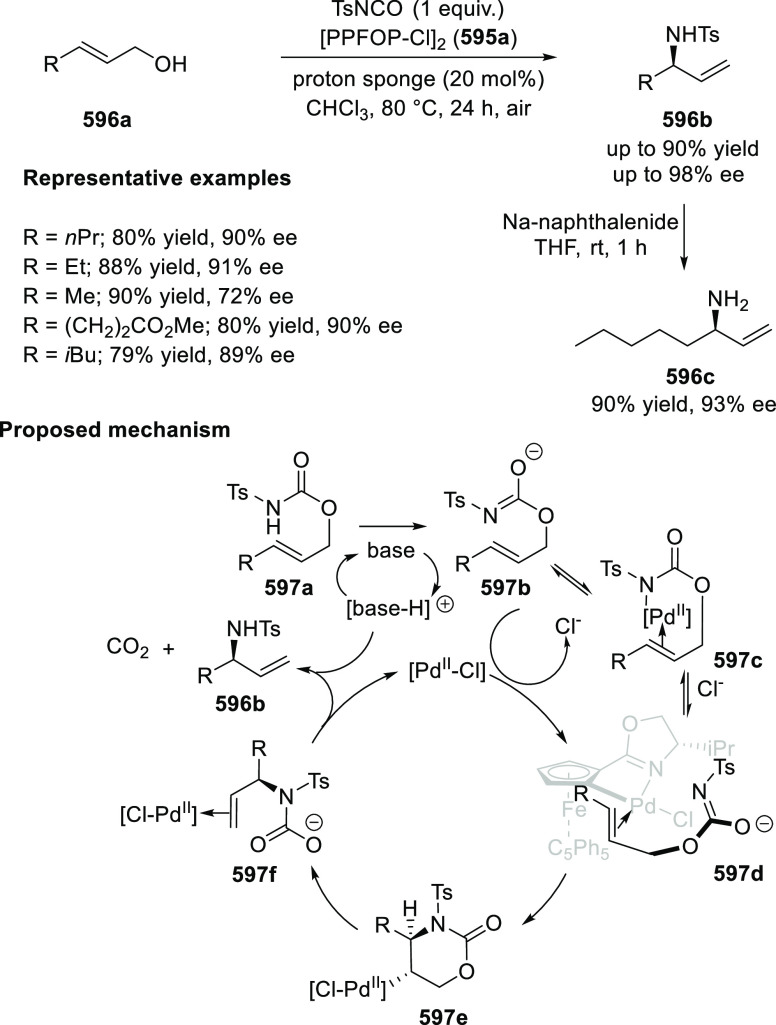
Regio- and Enantioselective Synthesis of Sulfonyl-Protected Chiral Allylic Amines

Peters reported the regio- and enantioselective synthesis of sulfonyl-protected chiral allylic amines (**596b**) from achiral allylic alcohols (**596a**) by using a catalytic ferrocene palladacycle, [PPFOP-Cl]_2_ (**595a**) and a tertiary amine (proton sponge -1,8-bis(*N*,*N*-dimethylamino)naphthalene) (Scheme 184).^221,222^ This reaction was highly step-economic and proceeded with no inert-gas atmosphere or catalyst activation by a silver salt. The preference for the branched allylic product was due to the Pd(II)-catalyzed cyclization-induced decarboxylative [3,3]-rearrangement of *in situ*-generated allylic carbamate intermediates. The allylic alcohol with an aliphatic group formed products in good to high yields and with high regio- and enantioselectivity whereas aromatic substituents were not well tolerated. The proposed mechanism of the Pd(II)/base-catalyzed decarboxylative allylic carbamate **597a** rearrangement is shown in Scheme 184. The deprotonated carbamate **597b** reacts with ferrocene palladacycle **595a** to form a chelate complex **597c**, the resting state, in which both the olefin and the anionic carbamate moiety are coordinated to the Pd center thus replacing the anionic ligand, Cl. Further dissociation of the carbamate *N*-atom in complex **597c**, which might be triggered by the reversible recoordination of the anionic ligand, Cl, generates complex **597d** which subsequently undergoes outer-sphere attack of the nucleophilic deprotonated *N*-center to the coordinated olefin to form intermediate **597e**. The cyclic aminopalladated intermediate **597e** featuring a σ-alkyl-Pd bond undergoes a ring-opening and β-hydride elimination to form the deprotonated carbaminic acid derivative **597f**. Decomplexation, decarboxylation, and protonation of **597f** leads to the formation of *N*-sulfonylated allylic amine **596b** and regenerates the Pd(II) and base catalysts for the next turnovers.

To conclude this section, chiral PPFOP ligands are successfully applied in the synthesis of chiral allyl amines via the asymmetric [3,3] rearrangement of allylic amides.

#### 2.5.7 Olefin-mono(oxazoline) Ligands

**Scheme 185:**
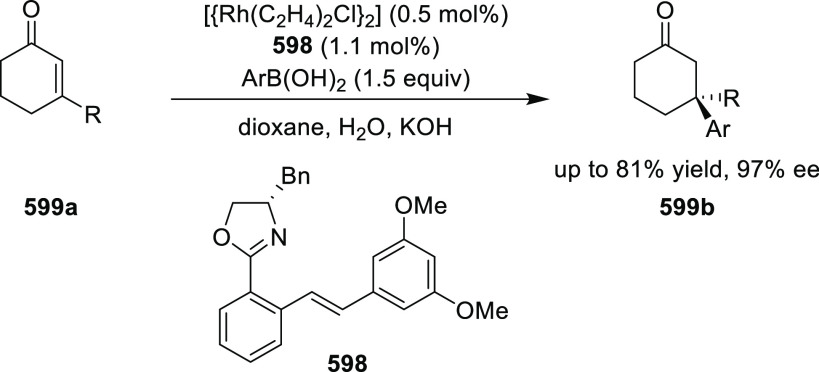
Rhodium Catalyzed Conjugate Addition of Phenylboronic Acids to Cyclohexanones

In 2010, Glorius developed the η^2^-binding ligand OlefOx **598** and applied it in the conjugate addition of aryl boronic acids to cyclohexanones **599a** (Scheme 185). The ligand **598** had a modular synthesis which allowed for fine-tuning of its structure, furnishing the substituted cyclohexanone product **599b** in up to 97% *ee* and 81% yield. It was thought that the η^2^-binding mode allowed for alternative coordination geometries to traditional η^1^-oxazoline ligand motifs.^223^

## 3 Bis(oxazoline) Ligands

### 3.1 Bis(oxazoline) Ligands with One Carbon Separating the Oxazoline Rings

#### 3.1.1 Parent Bis(oxazoline) Ligands with One Carbon Separating the Oxazoline Rings

For *C*_2_-symmetric bis(oxazoline) ligands, only examples reported after 2010 will be described in this section. For applications of these ligands prior to 2010, see the 2011 review by Desimoni and Jørgensen.^224^

**Figure 23:**
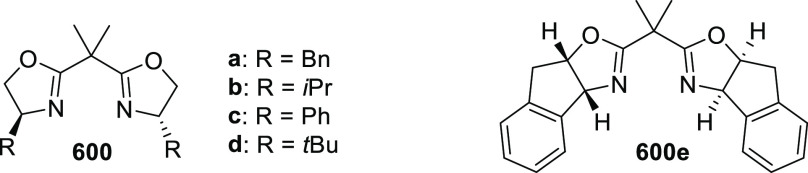
Parent BOX ligands.

**Scheme 186:**
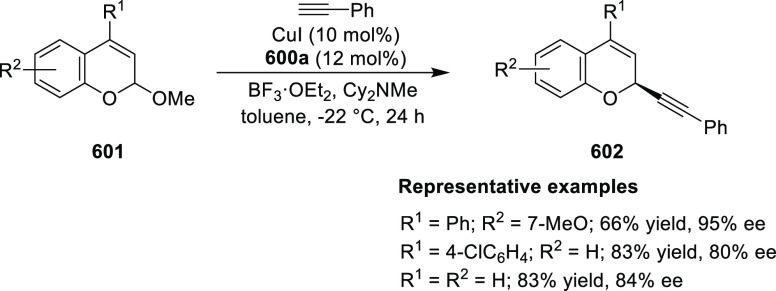
Cu-Catalyzed Asymmetric Alkynylation of Benzopyranyl Acetals

Watson has applied **600a** (Figure 23) in the alkynylation of benzopyranyl acetals **601** using chiral cuprates (Scheme 186). The reaction proceeded smoothly giving enantiomeric excesses of up to 95% and yields up to 90%. Regarding substrates, the enantioselectivity was enhanced to 95% *ee* when a methoxy was placed in the 7-position of the chromene acetal. Nonaromatic alkynes were shown to be detrimental to enantioselectivity with results dipping to 70% *ee* and 49% yield.^225^

**Scheme 187:**
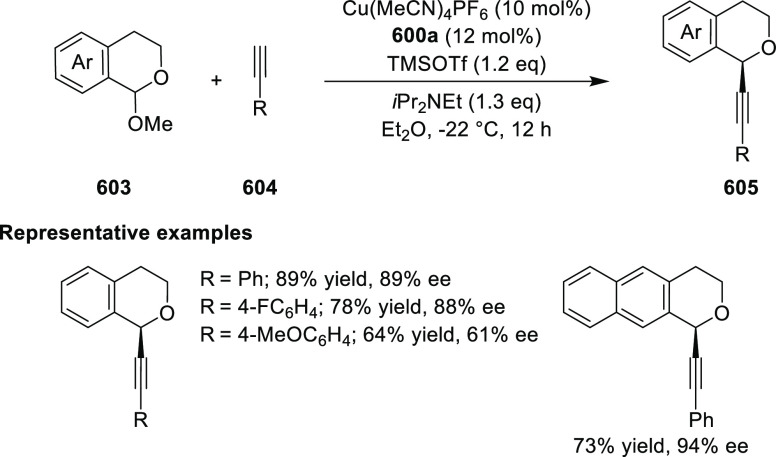
Cu-Catalyzed Asymmetric Addition of Alkynes

Watson developed an asymmetric Cu-catalyzed addition of terminal alkynes **604** to a TMSOTf-generated oxocarbenium ion (Scheme 187). By treating isochroman acetals **603** with TMSOTf, this generates an oxocarbenium ion that can be attacked by the chiral [Cu(MeCN)_4_]PF_6_-**600a** acetylene complex generating the product in up to 94% *ee* and good yields. It was shown that the *p*-methoxy electron-donating group on the alkyne lowered the enantioselectivity dramatically to 61% *ee*.^226^

**Scheme 188:**
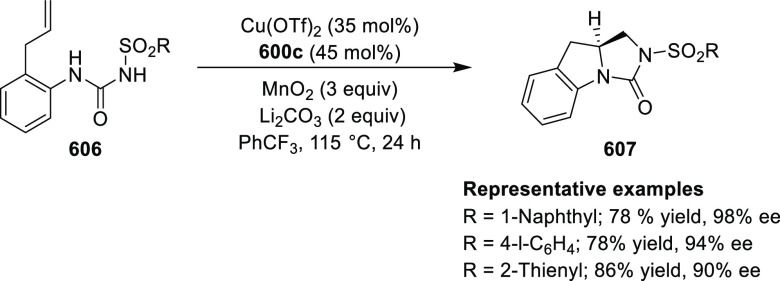
Cu-Catalyzed Intramolecular Cyclization of *N*-Alkenylureas

Zeng reported the application of **600c** (Figure 23) in the Cu-catalyzed intramolecular cyclization of *N*-alkenylureas **606** (Scheme 188). It allowed for the generation of chiral vicinal diamino bicyclic heterocycles in good yields and high enantioselectivities of up to 98% *ee*. Substituted aryl and heteroaryl systems were well tolerated with this catalysis with substrates possessing 2-thienyl substituted sulfonyl groups affording the corresponding product in 90% *ee* and 86% yield.^227^

**Scheme 189:**
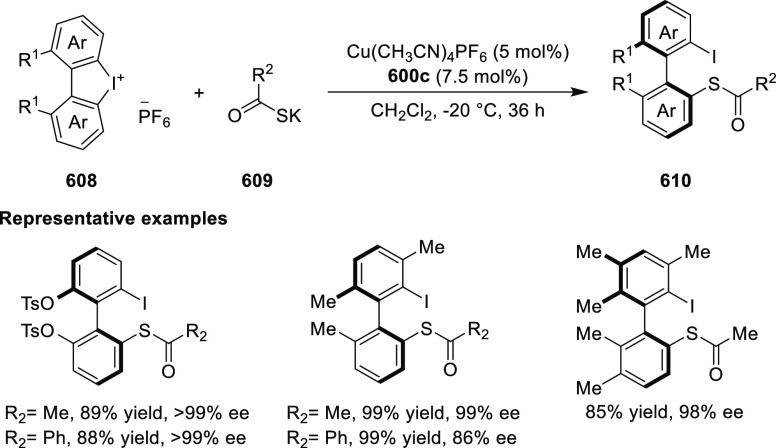
Cu-Catalyzed Ring Opening of Diaryliodonium Salts

Gu utilized Cu complexes of ligand **600c** in the thiolative ring opening of five-membered cyclic diaryliodonium salts **608** in order to synthesize atropoisomeric products and various axially chiral P,S ligands (Scheme 189). The best results were obtained using Cu(MeCN)_4_PF_6_ as the Cu(I) source, which provided high yields and enantioselectivities up to >99% *ee*. While most substrates were tolerated, bulky aryl thioates **609** appeared to reduce the enantioselectivity to as low as 86% *ee*.^228^

**Scheme 190:**
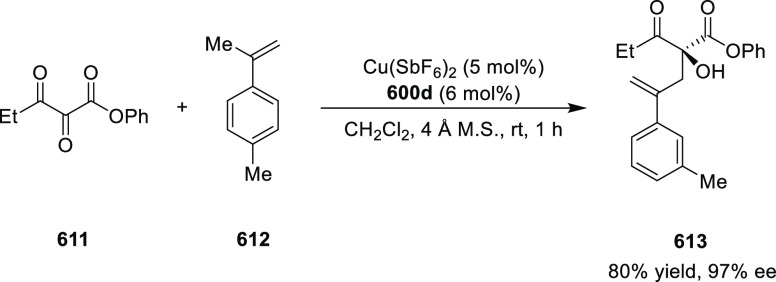
Enantioselective Carbonyl-Ene Reaction of 2,3-Diketo Esters

Doyle applied ligand **600d** (Figure 23) in the enantioselective carbonyl-ene reactions of 2,3-diketo esters **611** (Scheme 190). The best results were obtained with Cu(SbF_6_)_2_ as the Lewis acid and loadings as little as 1 mol% which furnished yields up to 94% and enantioselectivities up to 97% *ee*. This reaction was also performed on gram-scale while maintaining high yields and excellent enantioselectivities.^229^

**Scheme 191:**
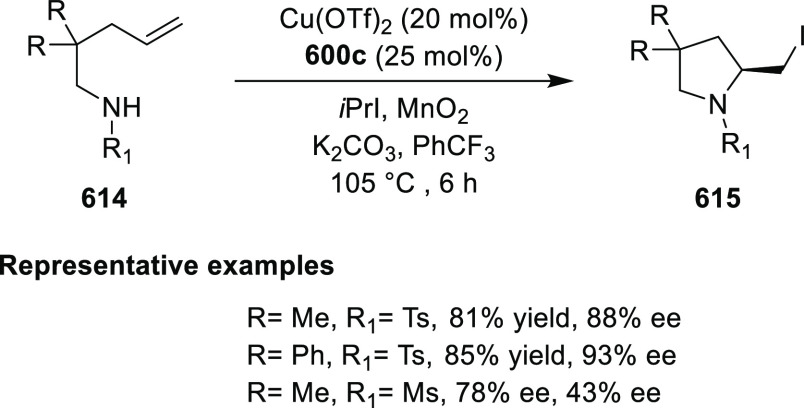
Cu-Catalyzed Asymmetric Aminohalogenation

Chemler applied ligand **600c** in the Cu-catalyzed aminohalogenation/cyclization reaction of 2-allylaniline **614** (Scheme 191). Isopropyl iodide was used as the halide source providing 2-iodomethylindoline products **615** in 93% *ee* and high yields. It was also noted that mesyl-protecting groups were not tolerated in this reaction with enantioselectivities lowered to 43% *ee*.^230^

**Scheme 192:**
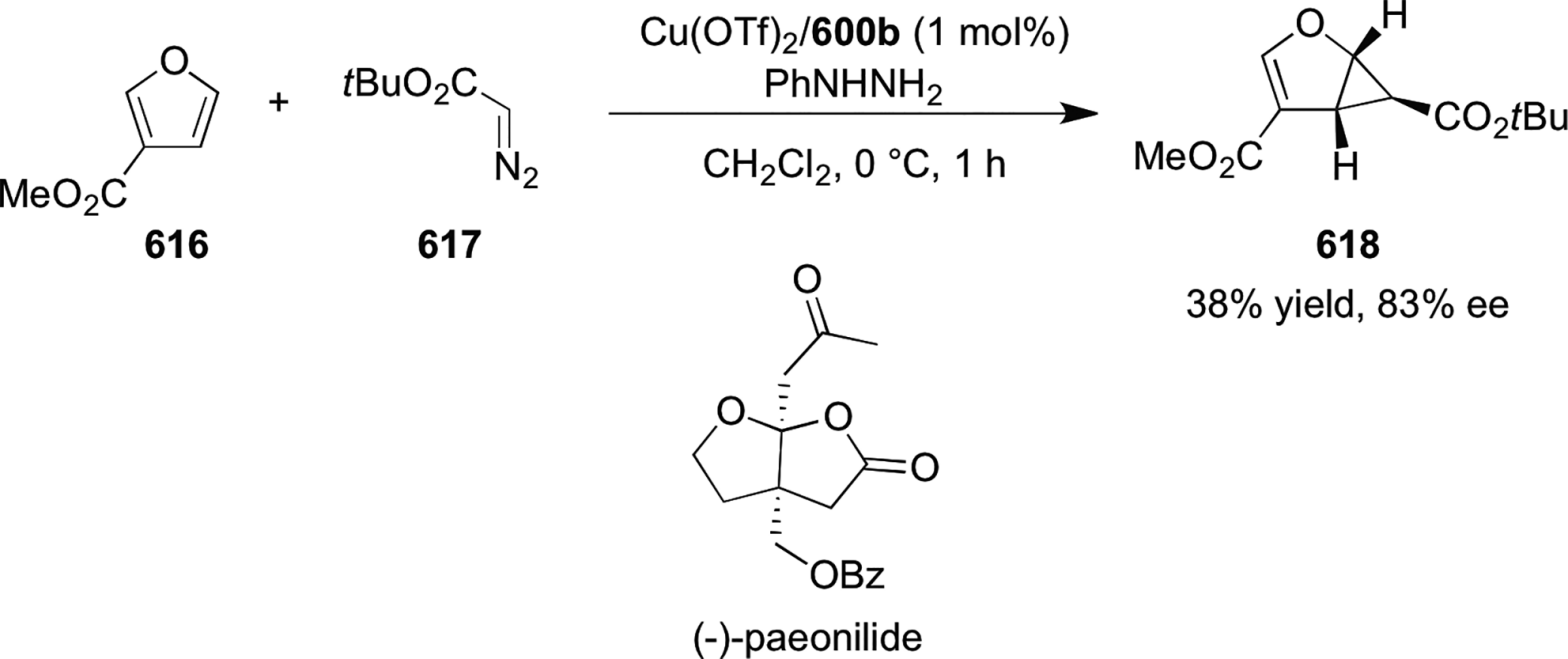
Cu-Catalyzed Asymmetric Cyclopropanation of Furans

Reiser applied ligand **600b** (Figure 23) in the Cu-catalyzed cyclopropanation of furans **616** in moderate yields and enantioselectivities of up to 83% *ee* (Scheme 192). This protocol was subsequently applied in the synthesis of (−)-paeonilide on a 20 g scale but this methodology was limited by the fact that a second cyclopropanation would occur if it was allowed to run to completion.^231^

**Scheme 193:**
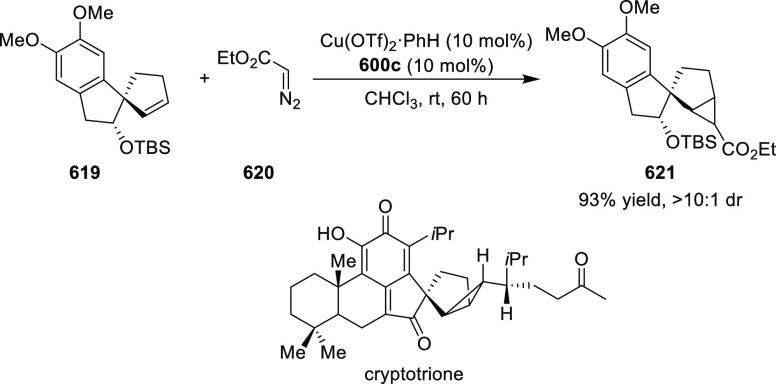
Cu-Catalyzed Asymmetric Synthesis of Cryptotrione Core

Shi also employed a similar cyclopropanation using a catalyst derived from (CuOTf)_2_·PhH and ligand **600c** achieving 93% yield and a diastereomeric ratio of >10:1 in the synthesis of Cryptotrione (Scheme 193). It was shown that slow addition of ethyl diazoacetate **620** via syringe pump was critical to obtaining the higher yields and enantioselectivities.^232^

**Scheme 194:**
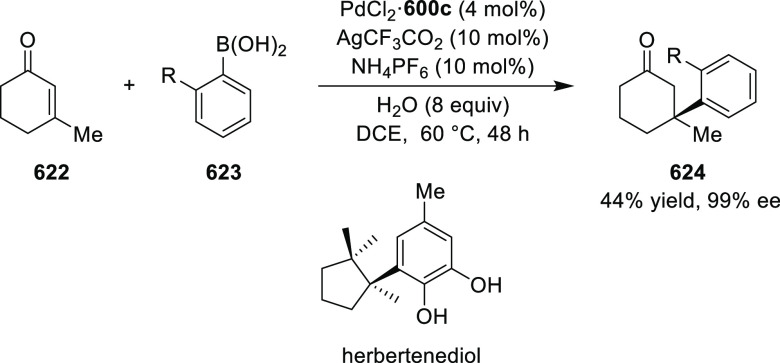
Pd-Catalyzed Michael Addition and Synthesis of Herbertenediol

Minnaard developed a Pd-catalyzed Michael addition of boronic acids to β,β-disubstituted cyclic enones **622** using chiral ligand **600c** affording products **624** with up to 99% *ee* (Scheme 194). The moderate yields were attributed to significant protodeboronation of the arylboronic acid. It was shown that larger *ortho*-substituents such as aldehydes, nitro groups, trifluoromethyl groups as well as di-*ortho* substitution were not tolerated on the aryl boronic acid **623**. The developed methodology was then applied in the enantioselective synthesis of sesquiterpenes such as herbertenediol.^233^

**Scheme 195:**
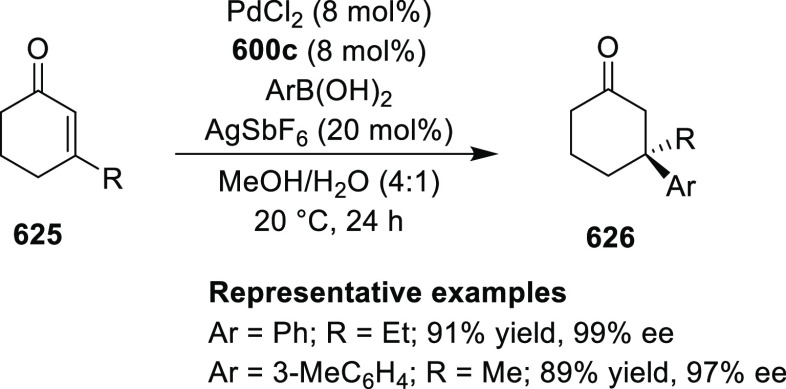
Pd-Catalyzed Asymmetric Conjugate Addition of Arylboronic Acids

Minnaard detailed an efficient Pd-catalyzed conjugate addition of arylbronoic acids to form β-substituted carbocyclic, heterocyclic and acyclic ketones. A PdCl_2_-**600c** complex provided the optimal results of up to 91% yield and 99% *ee*; however, the use of AgBF_4_ was needed to promote *in situ* dehalogenation to increase the conversion (Scheme 195). It was shown that the enantioselectivities dramatically dropped for acyclic enones with 60% *ee* being the highest reported.^234^

**Scheme 196:**
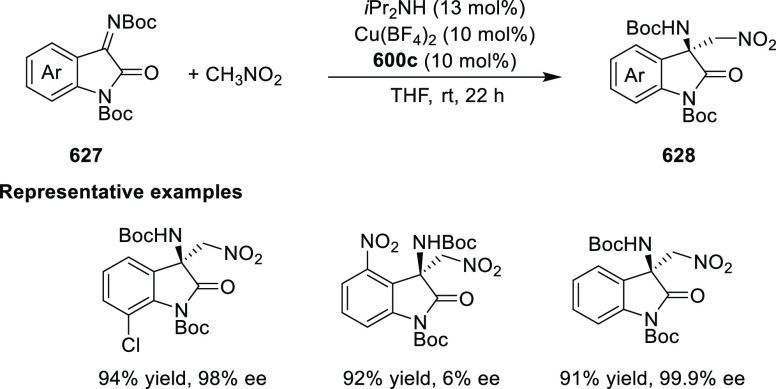
Cu-Catalyzed Asymmetric Henry Reaction of Isatin *N*-Boc Ketimines

Pedro applied ligand **600c** in the Cu-catalyzed aza-Henry reaction with isatin *N*-Boc ketimines **627** achieving yields up to 99% and outstanding *ee*s of 99.9% (Scheme 196). The reaction proceeds smoothly with or without protection of the isatin NH. The use of less acidic Cu(II) salts avoided imine hydrolysis but affected the enantioselectivity with CuCl_2_ affording the product in 97% yield and 27% *ee*. The best result was obtained with Cu(II) tetrafluoroborate hydrate and ligand **600c**. It was also shown that having a nitro group in the 7-position of the isatin significantly reduced the enantioselectivity to 6% *ee*.^235^

**Scheme 197:**
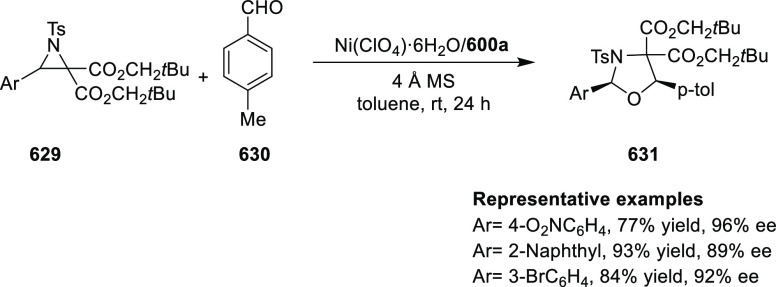
Ni-Catalyzed Asymmetric Cycloaddition of *N*-Tosylaziridines

Zhang applied ligand **600a** in the Ni-catalyzed cycloaddition of *N*-tosylaziridines **629** leading to very high yields of up to 99% and enantioselectivities up to 96% *ee* (Scheme 197). A variety of aziridines were subjected to the optimized reaction conditions, most of which achieved enantioselectivities of greater than 92% *ee*. Naphthyl-substituted aziridines however led to a reduction in enantioselectivity (89% *ee*).^236^

**Scheme 198:**
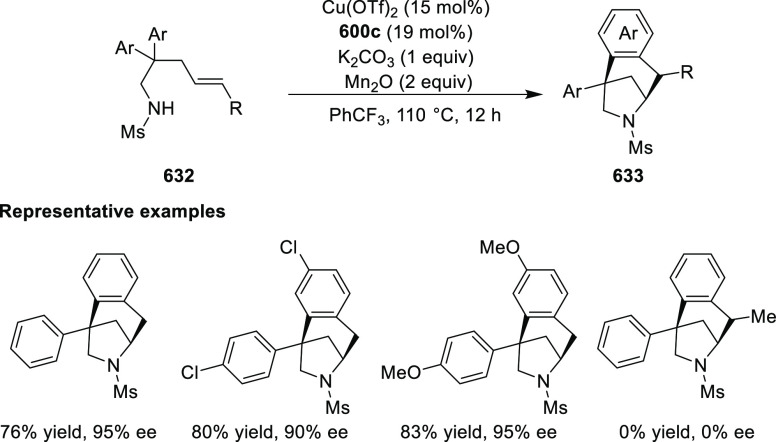
Cu-Catalyzed Synthesis of 6-Azabicyclo[3.2.1]octanes

Chemler utilized a **600c**-Cu complex in the synthesis of 6-azabicyclo[3.2.1]octanes **633** via an enantioselective alkene carboamination (Scheme 198). This process allowed for the rapid synthesis of bridged bicyclic rings containing nitrogen in good yields and up to 95% *ee*. Two new stereocenters are formed in the reaction, and the C–C bond-forming arene addition is a net C–H functionalization. Electron-donating and withdrawing arenes were tolerated in this reaction. However, this reaction was limited in the substitution of the alkene. If the alkene was 1,1 disubstituted the reaction proceeded smoothly but when 1,2-disubstituted the reaction halted completely, not giving any product.^237^

**Scheme 199:**
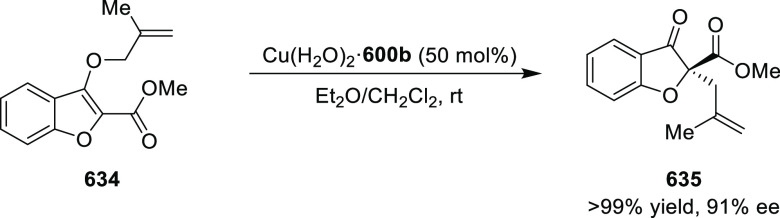
Cu-Catalyzed Asymmetric Synthesis of Dihydrofuran-3-ones

Tang developed an efficient synthesis of dihydrofuran-3-ones **634** and then applied them as substrates in an asymmetric Claisen rearrangement catalyzed by the Cu complex of ligand **600b**. This furnished the corresponding product **635** in quantitative yield and high enantioselectivities up to 91% *ee* (Scheme 199).^238^

**Scheme 200:**
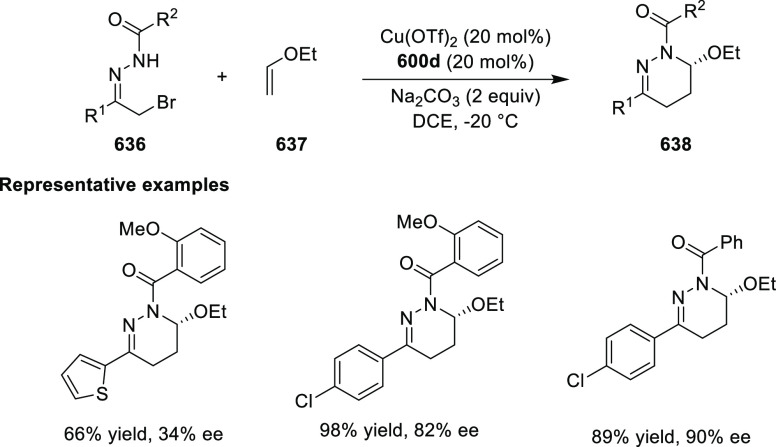
Cu-Catalyzed Synthesis of Chiral Pyridazine Derivatives

Xiao employed ligand **600d** in the Cu-catalyzed inverse electron-demand hetero-Diels–Alder reaction between α-halo-*N*-acylhydrazones **636** with enol ethers **637**. The reaction exhibits high enantioselectivity of up to 90% *ee* and provides densely substituted chiral pyridazine derivatives **638** in good yields (Scheme 200). It was shown that ester and thienyl-substituted hydrazones led to a drop in enantioselectivity to 34% *ee*.^239^

**Scheme 201:**
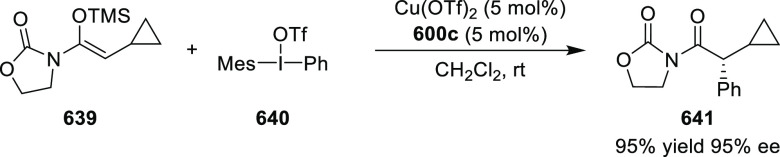
Cu-Catalyzed Asymmetric α-Arylation of *N*-Acyloxazolidinones

Gaunt applied chiral Cu-(II)-bisoxazoline complexes derived from ligand **600c** in the enantioselective α-arylation of *N*-acyloxazolidinones **639** by diaryliodonium salts **640** (Scheme 201). Cu(II) triflate was used as the Cu source providing the arylated product in up to 95% *ee* in excellent yields under mild conditions.^240^

**Scheme 202:**
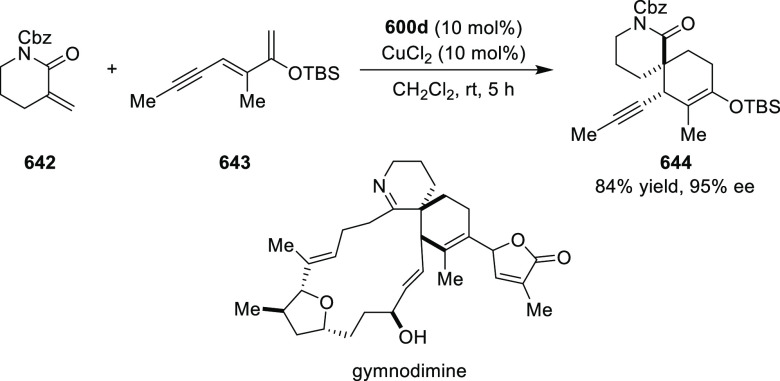
Cu-Catalyzed Synthesis of Gymnodimine Core

Romo employed a Cu-catalyzed Diels–Alder reaction to synthesize an intermediate of spirocyclic imine marine toxin (−)-Gymnodimine and an unnatural epimer. It provided the spirolactam product **644** in 84% yield and 95% *ee* (Scheme 202).^241^

**Scheme 203:**
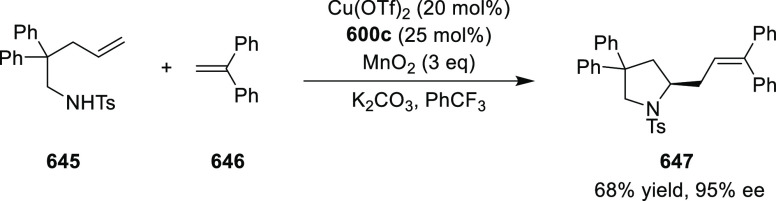
Cu-Catalyzed Asymmetric Amination of γ-Alkenylsulfonamides

Ligand **600c** was applied successfully in the intramolecular amination/Heck-type coupling of γ-alkenylsulfonamides **645** by Chemler. Using Cu(OTf)_2_ as the Cu source and **600c** as the ligand, enantioselectivities of up to 95% *ee* and 68% yield were observed for bulky alkenylsulfonamides (Scheme 203).^242^

**Scheme 204:**
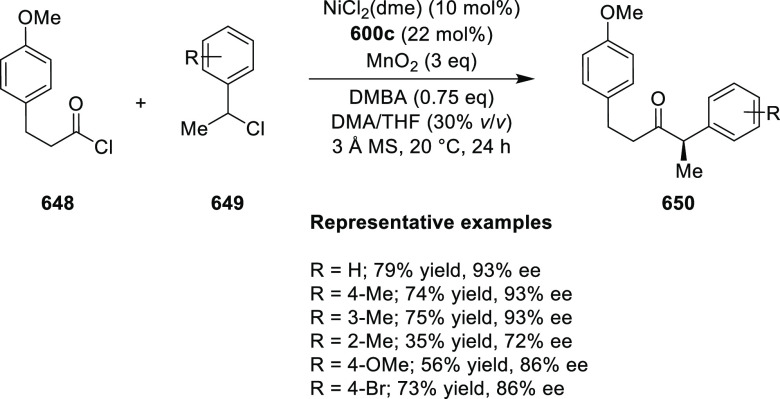
Ni-Catalyzed Asymmetric Synthesis of α,α-Disubstituted Ketones

Reisman utilized **600c** in the Ni-catalyzed synthesis of α,α-disubstituted ketones **650** from acyl chlorides **648** and racemic benzyl halides **649** (Scheme 204). The best results were obtained by using MnO_2_ as a stoichiometric reductant and DMBA (7,12-dimethylbenz[a]anthracene) to avoid homocoupling with 79% yield and *ee*s up to 93%. A wide variety of substrates with a range of different substitutions were tolerated, however it was shown that *ortho*-substituted benzyl chlorides were poor substrates providing products in only 35% yield and 72% *ee*. It was also noted that an unusual solvent mixture (30% v/v DMA/THF) was needed. It was shown that higher *ee*s but poor yields were obtained in THF while homocoupling issues remained when using pure DMA.^243^

**Scheme 205:**
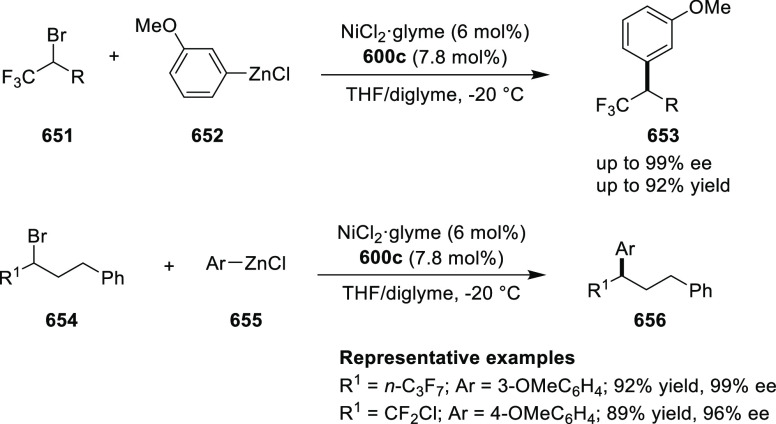
Ni-Catalyzed Asymmetric Cross-Electrophile Coupling

Fu showed that a catalyst combination of ligand **600c** and bench-stable NiCl_2_·glyme were able to synthesize tertiary stereocenters bearing a CF_3_ group in a Negishi arylation reaction in up to 92% yield and 99% *ee* (Scheme 205). It was shown that a variety of functional groups were compatible with the reaction conditions, including silyl ether, primary alkyl chloride, primary alkyl bromide, primary alkyl tosylate, aryl ether, ketone, aryl iodide, carbamate, ester and a furan with all of the above groups maintaining enantioselectivities of greater than 95% *ee*. It was also shown that a range of electron-withdrawing fluorinated groups could be applied to this methodology.^244^

**Scheme 206:**
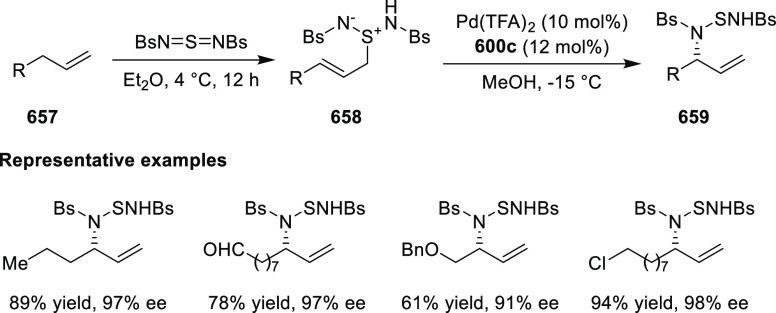
Enantioselective Allylic Amination of Terminal Olefins

Tambar developed a novel methodology for the enantioselective allylic amination of terminal olefins **657** by using a Pd(TFA)_2_-**600c** catalyst system which promotes the rearrangement of their stable ene-adducts **658** (Scheme 206). This furnished the product **659** in up to 98% *ee* with good to excellent yields. Also notable is the ability to accommodate a wide variety of functional groups, although carboxylic acids and free alcohols were not compatible in this methodology due to competing addition to the ene-adducts.^245^

**Scheme 207:**
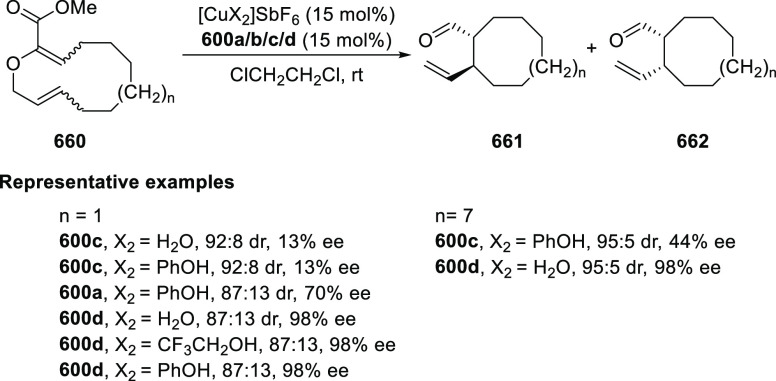
Cu-CAtalyzed [1,6]-Transannular Gosteli–Claisen Rearrangement of Cyclic Allyl Vinyl Ethers

Hiersemann reported a Cu-catalyzed [1,6]-transannular Gosteli–Claisen rearrangement of cyclic allyl vinyl ethers **660**. Throughout their study they showed that enantioselectivity and diastereoselectivity varied significantly depending on the ligand system used (Scheme 207). When diastereoselectivity was high the enantioselectivity dropped dramatically and *vice versa* when the enantioselectivity was high. For eight-membered ring systems the reaction had outstanding *trans*-selectivity and reached up to 98% *ee* and 87:13 dr with ligand **600d**. This methodology was extended to larger ring systems and displayed a switch to *cis*-selectivity affording the 12-membered ring product in up to 98% *ee* and 95:5 dr once again using chiral ligand **600d**.^246^

**Scheme 208:**
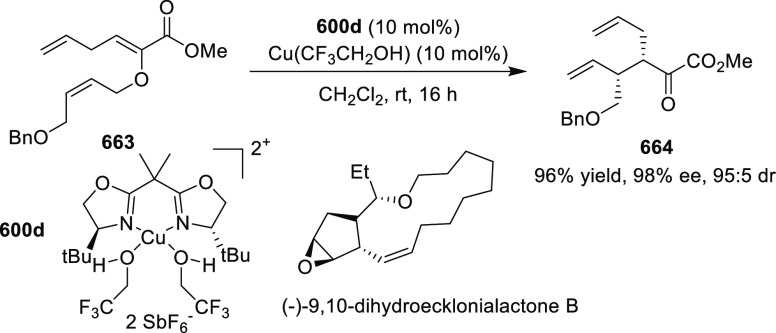
Cu-Catalyzed Claisen Rearrangement of Gosteli-type Allyl Vinyl Ethers

Hiersemann employed a **600d**-Cu(II) complex to prepare a key intermediate in the synthesis of (−)-9,10-dihydroecklonialactone B. This Claisen rearrangement of Gosteli-type allyl vinyl ethers **663** produced the key keto ester **664** in 96% yield, 95:5 dr and 98% *ee* (Scheme 208). It was shown that a modified catalyst-complex using bis(trifluoroethanol) was more effective for the Claisen rearrangement than the traditionally used bis(aqua) Cu(II) complex. This key intermediate was then converted in eight steps to (−)-9,10-dihydroecklonialactone B.^247^

**Scheme 209:**
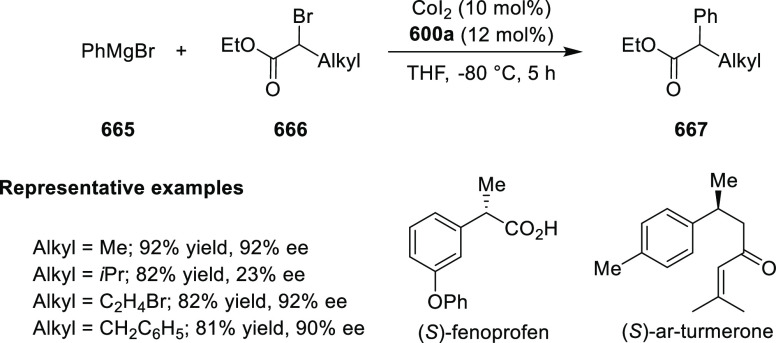
Co-Catalyzed Asymmetric Arylation of α-Bromo Ketones

Walsh reported the asymmetric arylation of α-bromo esters **666** using a CoI_2_ and **600a** catalyst complex with up to 96% yield and 97% *ee*. The reaction was tolerant of different cyclic/acyclic and aromatic esters. The scope for the alkyl chain showed tolerance for halides/alkenes/aromatic/heteroaromatic/esters while maintaining excellent enantioselectivities (Scheme 209). Interestingly *i*Pr- and cyclopentyl-containing α-bromo esters showed a significant drop in *ee*. The scope for the aryl-Grignard reagent **665** showed very little variability in *ee* regardless of sterics or electronics. This methodology was then employed in the synthesis of (*S*)-fenoprofen and (*S*)-ar-turmerone without erosion of *ee* in subsequent steps.^248^

**Scheme 210:**
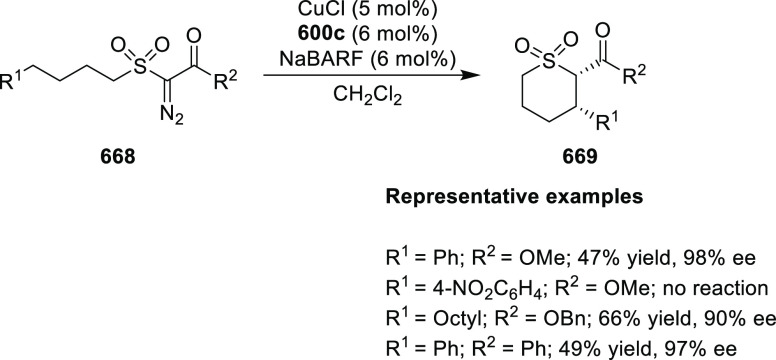
Cu-Catalyzed Synthesis of Thiopyrans

Maguire highlighted the use of CuCl and **600c** as a potent catalyst for intramolecular C–H insertion in the synthesis of a series of thiopyrans **669** (Scheme 210). The optimal catalyst complex in conjunction with NaBARF showed good yields and enantioselectivities of up to 98% *ee*. Interestingly the electron deficient nitrophenyl substrate did not yield any product. It was also shown that these reactions could be completed in significantly less time using microwave heating, but this came with a cost of reduced enantioselectivity. Further detailed investigations were also carried out by Maguire.^249−252^

**Scheme 211:**
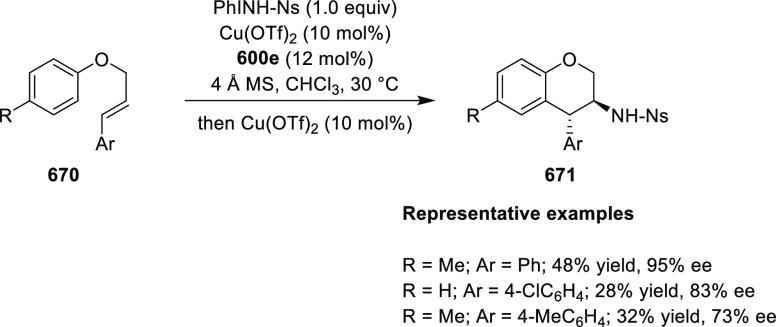
Cu-Catalyzed Asymmetric Aziridoarylation

Copper triflate and **600e** was utilized in the aziridoarylation of aryl cinnamyl ethers **670** for the synthesis of 3-amino-4-arylchromans **671** (Scheme 211). This one-pot, two-step reaction uses the catalyst mixture of **600e** and Cu(OTf)_2_ to effect a stereoselective aziridination, following additional Cu(OTf)_2_ the arylative ring-opening of the aziridine furnishes substituted chromans in moderate yields and enantioselectivities of up to 95% *ee*.^253^

**Scheme 212:**
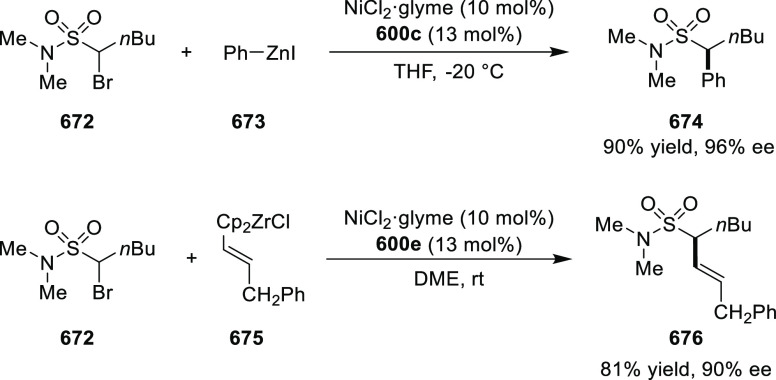
Ni-Catalyzed Asymmetric Negishi Coupling of Sulfonamides

Fu reported the use of NiCl_2_-glyme and ligand **600c** as an effective catalyst for the asymmetric Negishi coupling of sulfonamides and sulfones **672** with organozinc reagents **673** in good yields and reaching up to 96% *ee* (Scheme 212). A wide range of substrates including aromatic, cyclic and acyclic sulfonamides were investigated, although for sulfones there was a more limited scope which proceeded with high enantioselectivity of 90% *ee*. The alkenylation of α-bromosulfonamides using zirconium reagents **675** as the nucleophile and **600e** as the ligand of choice proceeded in 81% yield and 90% *ee*.^254^

**Scheme 213:**
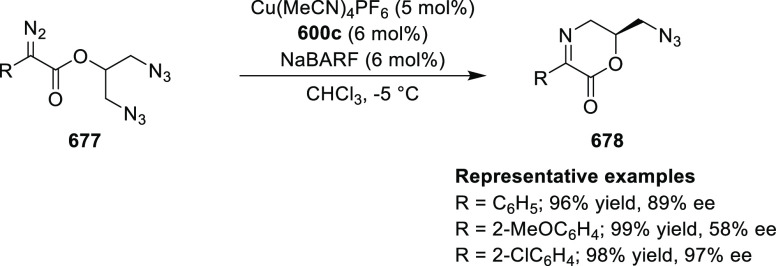
Cu-Catalyzed Synthesis of Cyclic Iminoesters

Gu developed an enantioselective desymmetrization of 1,3-diazoisopropyl diazo(aryl)acetates **677** catalyzed by a Cu complex with ligand **600c** to furnish imino esters **678** in very high yields and enantioselectivities of up to 97% *ee* (Scheme 213). The best results were obtained when using the large noncoordinating counterion, NaBARF, which is often employed in these reactions to increase the enantioselectivity. The substituents on the aromatic ring slightly affected the enantioselectivity. The *ortho*-groups showed the largest effect on enantioselectivities with electron-donating groups greatly diminishing the enantioselectivity to 58% *ee*.^255^

**Scheme 214:**
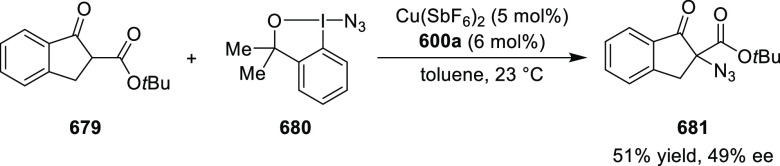
Cu-Catalyzed Asymmetric Azidation of β-Keto Esters

Waser developed the azidation of β-keto esters **679** and silyl enol ethers using a benziodoxole reagent **680** (Scheme 214). This methodology was expanded upon but not fully optimized with an initial test reaction using ligand **600a** and a Cu salt showing moderate enantioselectivities of 49% *ee*.^256^

**Scheme 215:**
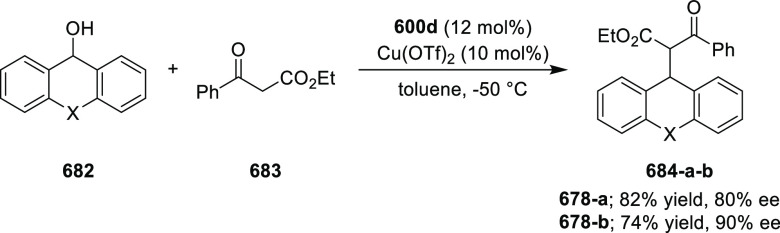
Cu-Catalyzed Alkylation of β-Keto Esters

Nájera disclosed a Cu-catalyzed asymmetric alkylation of β-keto esters **683** using ligand **600d** and benzylic alcohols **682** as the alkylating agents (Scheme 215). Xanthydrol and thioxanthydrol were the model alkylating reagents while aryl, alkyl and lactone-based β-keto esters were all shown to be good substrates for this reaction, and good yields with enantioselectivities of up to 90% *ee* were observed.^257^

**Scheme 216:**
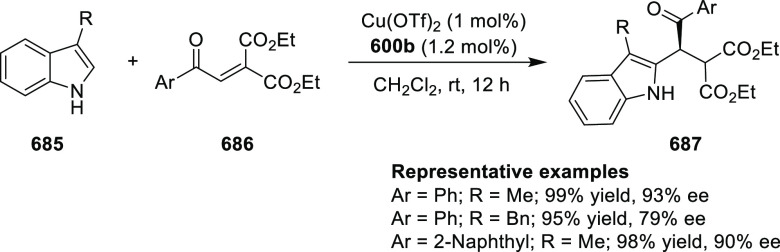
Cu-Catalyzed Friedel–Crafts Alkylation of Indoles

Jia performed the asymmetric Friedel–Crafts alkylation of 3-substituted indoles **685** using Cu(OTf)_2_ and ligand **600b** as the optimal catalyst and ligand system achieving excellent yields and enantioselectivities of up to 93% *ee* (Scheme 216). This reaction was performed with α,β-unsaturated esters **686** and was sensitive only to bulky substituents on the indole ring.^258^

**Scheme 217:**
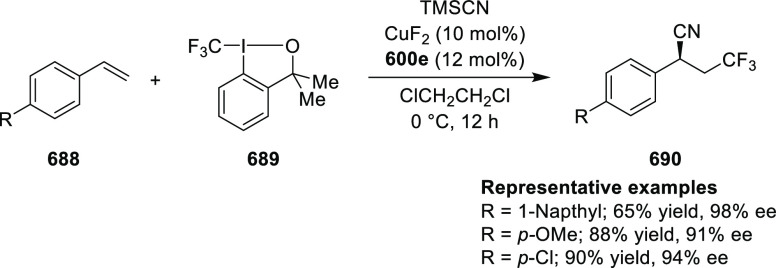
Cu-Catalyzed Cyano-trifluoromethylation of Styrenes

Pan disclosed an asymmetric cyano-trifluoromethylation of styrenes **688** using Togni’s reagent **689** as the electrophilic source of CF_3_ and **600e** as the chiral ligand (Scheme 217). CuF_2_ was shown to be the ideal combination with ligand **600e** and the products were formed in good yields and enantioselectivites of up to 98% *ee*. Electron-withdrawing and donating styrenes were tolerated remarkably well with *p*-methoxystyrene giving the lowest yield of 88% with 91% *ee*.^259^

**Scheme 218:**
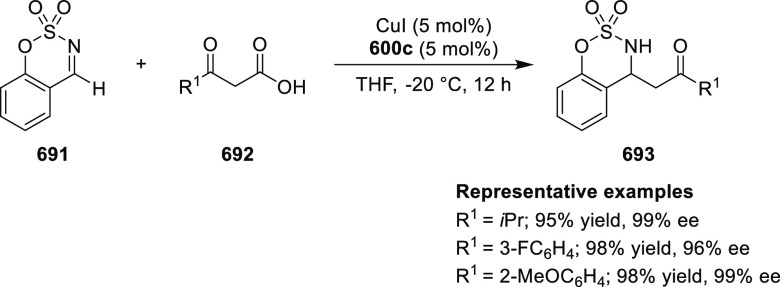
Cu-Catalyzed Decarboxylative Mannich Reaction

Ma disclosed a highly enantioselective decarboxylative Mannich reaction of β-ketoacids **692** and cyclic aldimines **691** (Scheme 218). Using ligand **600c** and CuI excellent yields and enantioselectivities of up to 99% *ee* were achieved. No substrate dipped below 90% *ee* with a wide variety of electronically different substrates being tolerated by the reaction conditions.^260^

**Scheme 219:**
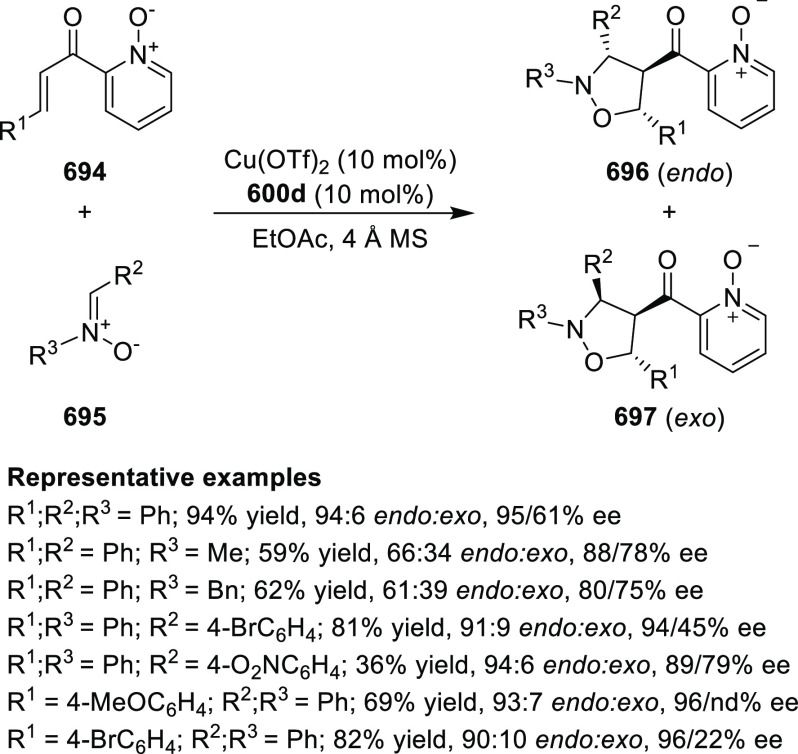
Cu-Catalyzed Cycloaddition of Nitrones and Pyridine *N*-Oxides

Pedro reported the cycloaddition between nitrone **695** and 2-alkenoyl pyridine *N*-oxides **694** to give the isooxazolidine **696**/**697** products in high *ee* and de (Scheme 219). It was shown that *N*-phenyl nitrones performed much better in catalysis than *N*-methyl or *N*-benzyl nitrones which gave a much higher mixture of *endo*:*exo*. The nitrone derived from *p*-nitrobenzaldehyde formed the product in poor yields although the enantioselectivities remained high at 89% *ee*. The scope for the 2-alkenoyl pyridine *N*-oxides was remarkably tolerant of varying electronic properties achieving high *endo*:*exo* ratios while the enantioselectivities remained high.^261^

**Scheme 220:**
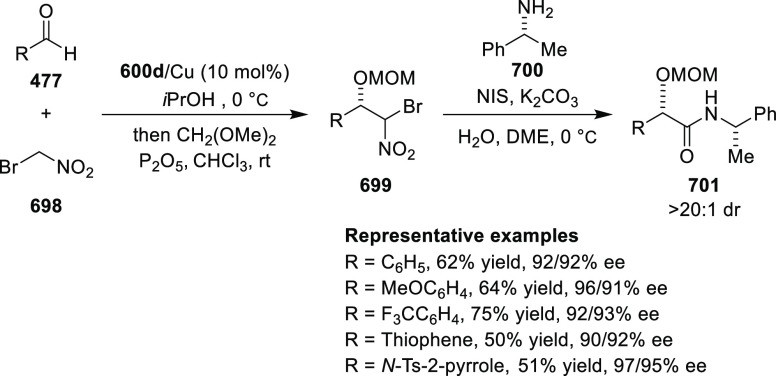
Cu-Catalyzed Asymmetric Umpolung Amide Synthesis

Johnston utilized ligand **600d** in a three-step sequence for an umpolung amide synthesis (Scheme 220). Aldehydes **477** were reacted with bromonitromethane **698** in an enantioselective Henry addition using Cu(II) *ortho*-iodo benzoic acid as the catalyst, followed by MOM protection of the corresponding alcohol. This gave the MOM-protected alcohols **699** in good yields and with high enantioselectivities of up to 99% *ee*. The MOM-protected alcohols **699** were then subjected to the umpolung amide synthesis using enantioenriched (>99% *ee*) α-methyl benzyl amine **700** and these products **701** were used to provide confirmation that the two diastereomers of **699** are homochiral at the benzylic carbon (differing at the nitro-α-carbon) and remained enantioenriched throughout the three-step sequence (Henry/MOM-protection/umpolung amide synthesis).^262^

**Scheme 221:**
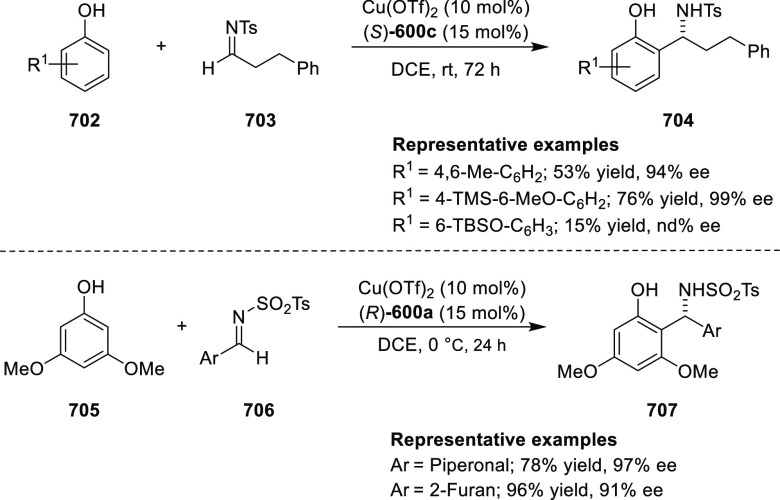
Cu-Catalyzed Asymmetric Aza-Friedel–Crafts Reaction

Chemler developed a Cu-catalyzed aza-Friedel–Crafts reaction between phenols **702** and aldimines **703** that provides chiral secondary benzylamines **704** with enantioselectivities up to 99% *ee* using **600c** as the chiral ligand (Scheme 221). With regards to the phenol scope most substrates were well tolerated, however some substrates such as 1,3,5-trimethoxyphenol and 5-OTBS phenol gave poor conversions as low as 15% yield and enantioselectivites of <5% *ee*. When the aryl imine scope was attempted the conditions had to be reoptimized using (*R*)-**600a** as the ligand and lower temperatures to provide better enantioselectivity of up to 97% *ee*.^263^

**Scheme 222:**
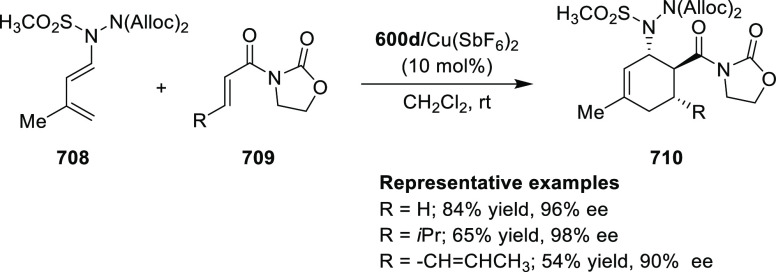
Cu-Catalyzed Asymmetric Diels–Alder of 1-Hydrazinodienes

Sorensen reported the asymmetric Diels–Alder cycloaddition of 1-hydrazinodienes **708** and *N*-acryloyl oxazolidinones **709** using ligand **600d** and Cu(SbF_6_)_2_ as the catalyst complex (Scheme 222). The scope of the reaction was varied and tolerated different substituents on the substituted *N*-acryloyl oxazolidinones and the lowest enantioselectivities were seen when *p*-Cl/Br/CF_3_ substituted aryl rings were used all affording 90% *ee*. When changing the 1-hydrazinodienes there was little to no drop in enantioselectivity with 97% *ee* being the lowest recorded.^264^

**Scheme 223:**
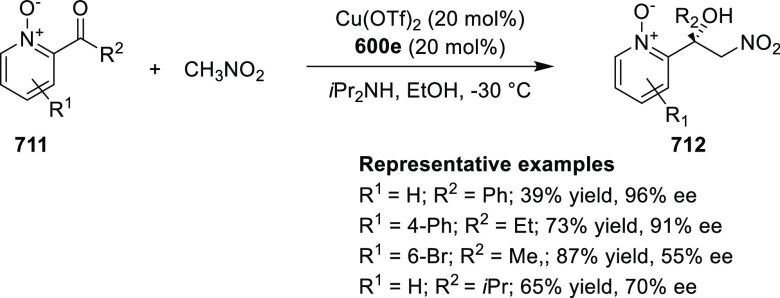
Cu-Catalyzed Asymmetric Addition to 2-Acylpyridine *N*-Oxides

An asymmetric addition of nitromethane to 2-acylpyridine *N*-oxides **711** was reported by Pedro in 2014. Using **600e** and Cu(OTf)_2_ as the catalyst complex and ethanol as the solvent enantioselectivities up to 96% *ee* were achieved (Scheme 223). Ethanol was chosen as the solvent as it stopped competitive background reactions from occurring. Substrates substituted on the 6-position had a disastrous effect on the enantioselectivity which dropped to as low as 48% *ee*.^265^

**Scheme 224:**
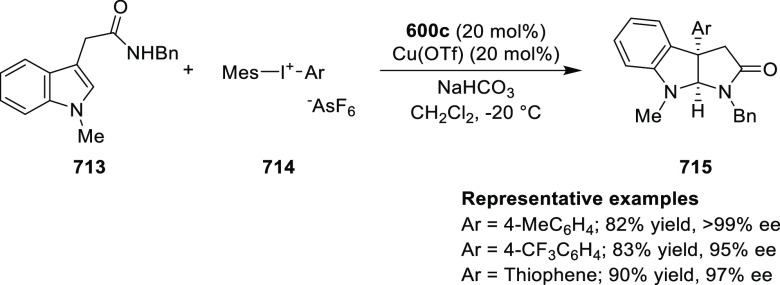
Copper-Catalyzed Asymmetric Synthesis of Pyrroloindolines

MacMillan developed an enantioselective cascade arylation-cyclization using Cu(OTf), **600c** and aryl iodonium salts **714** to synthesize aryl pyrroloindolines **715** (Scheme 224). In general, the reaction was highly enantioselective with *ee*’s up to >99% *ee*. Unprotected indole starting material has the largest negative impact on the catalytic transformation as enantioselectivity was lowered to 90% *ee*.^266^

**Scheme 225:**
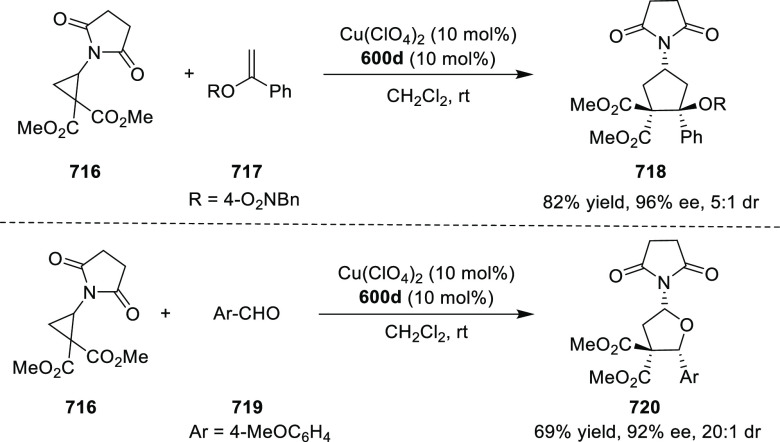
Dynamic Kinetic Asymmetric Annulation of Aminocyclopropanes

Waser reported a dynamic kinetic asymmetric annulation of aminocyclopropanes **716** with enol ethers **717** and aldehydes furnishing up to 96% *ee* and 92% *ee*, respectively (Scheme 225). The best enantioselectivities were achieved with Cu(ClO_4_)_2_-**600d** as the catalyst system and succinimide as the nitrogen-containing component of the aminocyclopropanes with up to 96% *ee* being obtained. It was also shown that the counterion had a large influence on the *ee*, hexafluoroantimonate led to the highest dr’s but when perchlorate was chosen it provided the highest enantioselectivities.^267^

**Scheme 226:**
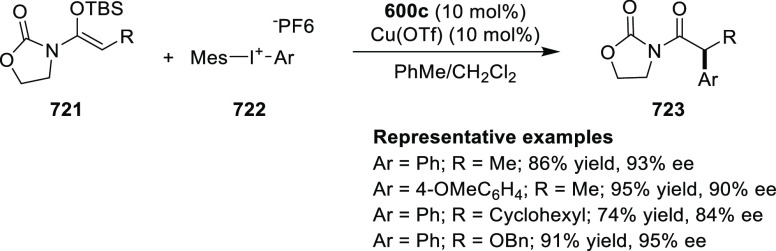
Cu-Catalyzed Enantioselective α-Arylation of Carbonyls

MacMillan published the enantioselective α-arylation of carbonyls using aryliodonium salts **722** with ligand **600c** and Cu(OTf) as the active catalyst system (Scheme 226). Using silyl enol ethers **721** as the silylated nucleophile they attacked the Cu-oxazoline iodonium salt complex to give good yields and enantioselectivities up to 95% *ee*. It was shown that using the PF_6_ counterion and a mixed solvent system of toluene/CH_2_Cl_2_ at 0 °C produced the best yields. A diverse range of aryl and heteroaryl rings were tolerated well in catalysis. As well as this, arenes, olefins, ethers, esters, and carbamates were all tolerated for the silyl enol ether scope.^268^

**Scheme 227:**
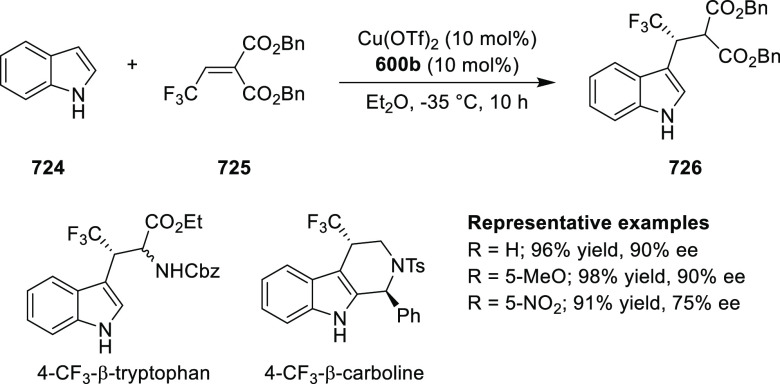
Cu-Catalyzed Friedel–Crafts Alkylation of Indoles

Lu developed a Cu-catalyzed Friedel–Crafts alkylation of indoles **724** with trifluoroethylidene malonates **725** to construct a tertiary stereocenter bearing a trifluoromethyl group (Scheme 227). Using Cu(OTf)_2_ and **600b** as the catalyst system excellent conversions and enantioselectivities of up to 90% *ee* were achieved. This protocol was then utilized in the synthesis of β-CF_3_-tryptophan and 4-CF_3_-β-carboline.^269^

**Scheme 228:**
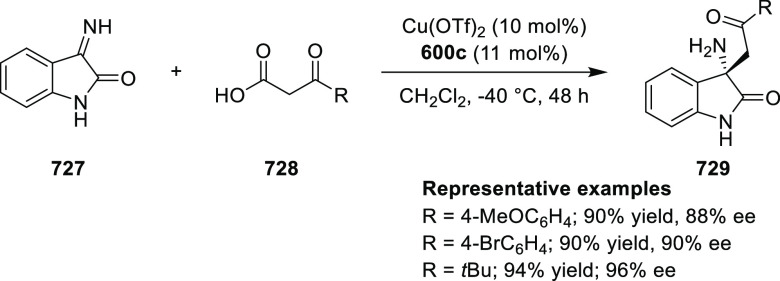
Cu-Catalyzed Enantioselective Decarboxylative Mannich Reaction of Unprotected Isatin-Derived Ketimines

Ohshima reported an enantioselective decarboxylative Mannich-type reaction of unprotected isatin-derived ketimines **727** (Scheme 228). By utilizing a β-keto acid **728** as the nucleophile this led to an irreversible decarboxylation addition process. Using Cu(OTf)_2_ and **600c** as the ligand the desired product was formed in up to 96% *ee* and very high yields (of up to 90%). With regards to the β-keto acids this reaction was also tolerant of heteroaryl systems such as furan/thiophene and naphthyl with good enantioselectivities (85–92% *ee*) being obtained.^270^

**Scheme 229:**
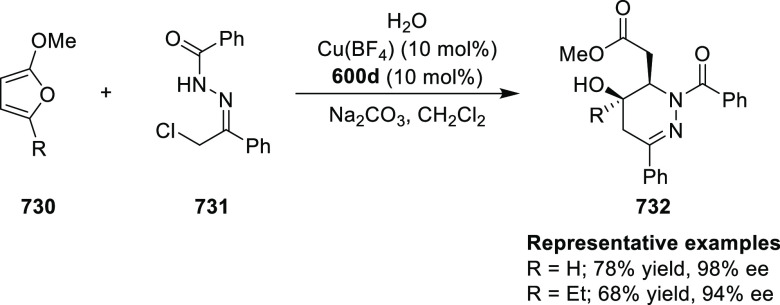
Cu-Catalyzed Inverse Aza-Diels–Alder Cascade Reaction

Wang disclosed a multicomponent cascade inverse electron-demand Aza-Diels–Alder/nucleophilic addition/ring-opening reaction using 2-methoxyfurans **730** as dienophiles (Scheme 229). This reaction was envisaged to synthesize a bicyclic heterocycle, but water does a nucleophilic addition following a ring opening leading instead to a series of tetrahydropyridazine derivatives **732**. With this methodology in place a series of substituted tetrahydropyridazines were synthesized. Electron-donating and electron-withdrawing groups were all well tolerated with enantioselectivities in the 92–98% *ee* range for this Cu-catalyzed process.^271^

**Scheme 230:**
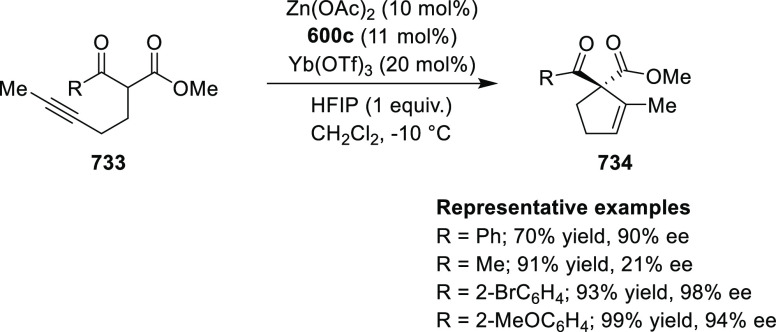
Dual-Catalytic Asymmetric Cyclization

Shibata developed a Zn(OAc)_2_/Yb(OTf)_3_ cocatalyst system to induce an intramolecular 5-*endo*-*dig* cyclization of β-keto esters **733** and alkynes to produce enantioenriched cyclopentene products of type **734** (Scheme 230). The best results were achieved using **600c** as the ligand and 1 equiv of hexafluoroisopropanol (HFIP). It was proposed that the effect of HFIP was to improve catalytic turnover by releasing the products from the catalytic cycle or assist initial enolization. It was believed that the generally high *ee*s were due to the π–π stacking interactions of aromatic ketones with the phenyl groups of the ligand **600c**. This hypothesis was backed up by the fact that the reaction with nonaromatic ketones led to poor enantioselectivities as low as 21% *ee*.^272^

**Scheme 231:**
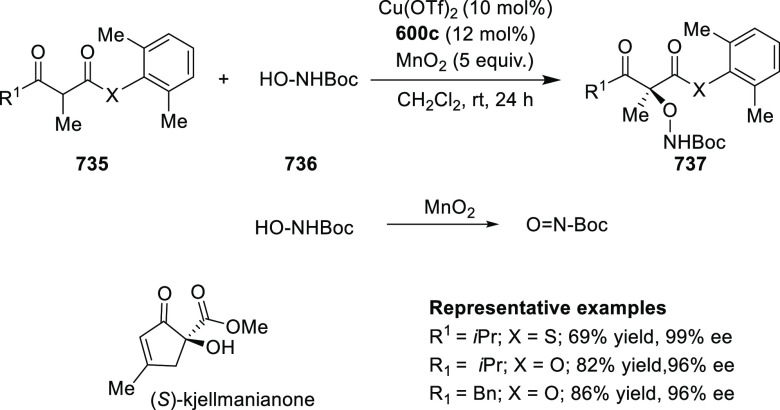
Cu-Catalyzed Asymmetric Nitrosocarbonyl Aldol Reaction

Yamamoto reported an asymmetric synthesis of α-hydroxy-β-keto esters **737** in a Cu-catalyzed *O*-nitrosocarbonyl aldol reaction (Scheme 231). Using ligand **600c** and Cu(OTf)_2_ as the catalyst complex high levels of enantioselectivities up to 99% *ee* were observed. MnO_2_ was chosen as the oxidizing reagent as it was found to be a mild method for the generation of nitrosocarbonyl compounds which avoided overoxidation of the products. This catalysis was shown to be particularly flexible as both oxo-ester and thioesters **735** can be tolerated while retaining high levels of enantioselectivity. This methodology was then applied to the synthesis of antibacterial agent (*S*)-Kjellmanianone.^273^

**Scheme 232:**
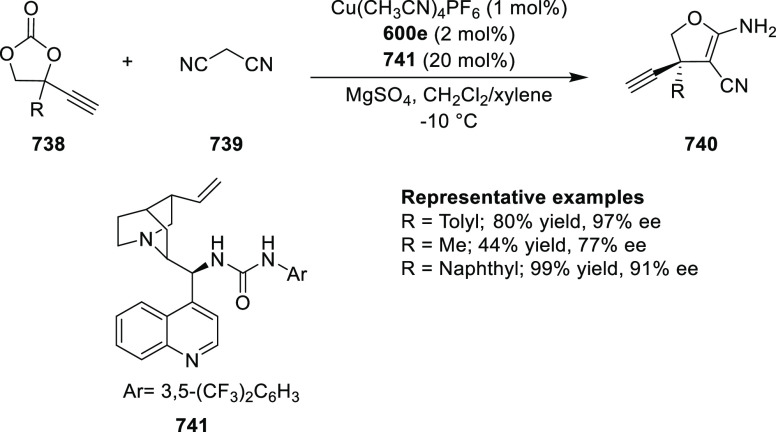
Bicatalytic [3 + 2] Cycloaddition Reaction of Ethynylethylene Carbonates

Song used ligand **600e** and Cu(CH_3_CN)_4_PF_6_ in combination with cinchona alkaloid **74** to promote a [3 + 2] cycloaddition reaction of ethynylethylene carbonates **738** and malononitrile **739** (Scheme 232). This system relied on cinchona alkaloid **741** for activation of the malononitrile and a Cu-mediated decarboxylation of a carbonate followed by a cycloaddition. The ligand **600e** was not fully responsible for enantioselectivity as the choice of alkaloid was also shown to have an impact on the enantioselectivity. Good yields and enantioselectivities up to 97% *ee* were achieved using this copper/organocatalytic system.^274^

**Scheme 233:**
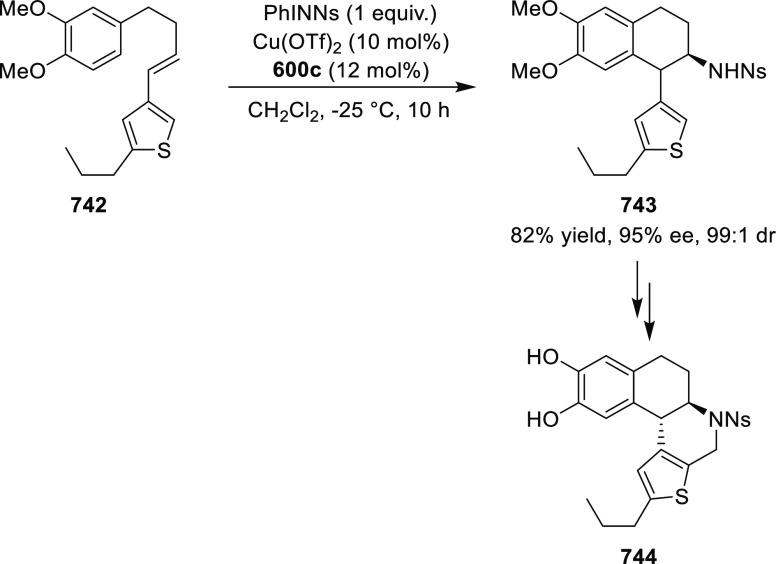
Cu-Catalyzed Asymmetric Synthesis of Dopamine D1 Agonist

Bar utilized a one-pot- aziridination/Friedel–Crafts cyclization in the synthesis of dopamine D1 agonist A-86929 **744** (Scheme 233). Using ligand **600c** in combination with Cu(OTf)_2_ it was shown to be an effective catalyst for the asymmetric aziridination and subsequent Friedel–Crafts cyclization. This provided the key intermediate in good yields, > 99:1 dr and 95% *ee*.^275^

**Scheme 234:**
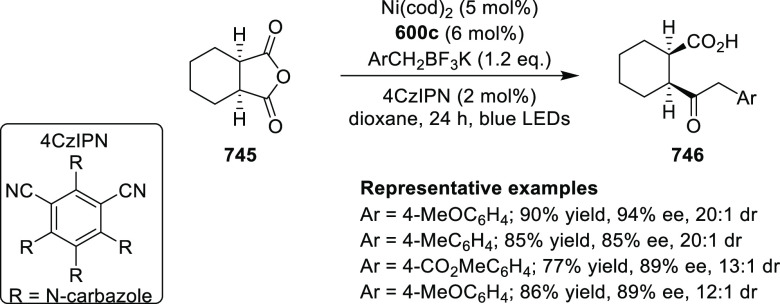
Ni-Catalyzed Asymmetric Desymmetrisation of Cyclic *meso*-Anhydrides

Doyle and Rovis reported a dual Ni- and photoredox-catalyzed enantioselective desymmetrization of cyclic *meso*-anhydrides **745** (Scheme 234). Using **600c**, Ni(cod)_2_ and photocatalyst 4CzIPN, enantioselectivities up to 94% *ee* were achieved, with dr values of >20:1. A range of electrophiles were tested such as cyclopentyl, cyclobutyl and cyclopropyl succinic anhydrides, all of which were suitable. However, anhydrides bearing β-substitution provided moderate enantioselectivities of 36% *ee*. Both electron-deficient and electron-neutral trifluoroborates reacted smoothly. Some *ortho*-substituted nucleophiles led to a lower diastereoselectivity of 6:1 dr.^276^

**Scheme 235:**
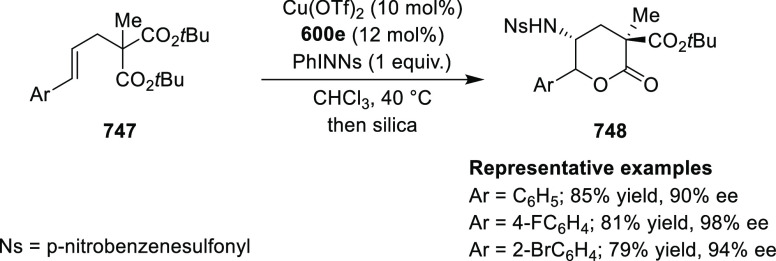
Cu-Catalyzed Asymmetric Aminolactonization

Aziz developed an asymmetric aminolactonization of 1,2 disubstituted alkenoic acid ester **747** via an aziridination-cyclization reaction protocol (Scheme 235). Using Cu(OTf)_2_ and ligand **600e** the aziridination proceeds smoothly and upon treatment with additional Cu(OTf)_2_ or silica gel the aziridine successfully cyclizes to produce the desired aminolactone **748** in up to 98% *ee*.^277^

**Scheme 236:**
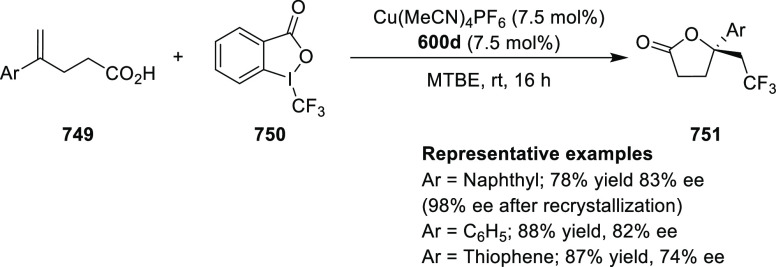
Cu-Catalyzed Synthesis of Enantioenriched CF_3_-Containing Lactones

Buchwald developed an efficient Cu-catalyzed synthesis of enantioenriched CF_3_-containing lactones **751** (Scheme 236). Using **600d** as the chiral ligand and[Cu(MeCN)_4_]PF_6_ as the Cu source enantioselectivities up to 98% *ee* after recrystallization were achieved in good yields. Togni’s reagent **750** proved to be a competent radical coupling reagent which promoted an asymmetric cyclization. Mechanistic studies supported a redox radical addition mechanism. This reaction showed a good functional group compatibility with a variety of aryl and heteroaryl systems being tolerated.^278^

**Scheme 237:**
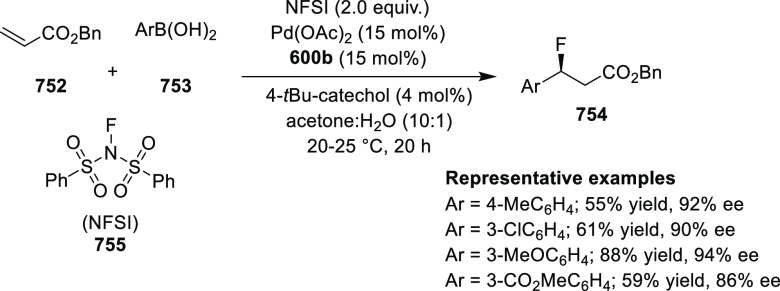
Pd-Catalyzed Three-Component Coupling of Deactivated Alkenes

Fustero and Toste documented a three-component coupling of deactivated alkenes **752**, aryl boronic acids **753** and *N*-fluorobenzenesulfonimide **755** (Scheme 237). This asymmetric Pd-catalyzed functionalization of α,β-unsaturated systems afforded good yields and high enantioselectivities using a Pd(OAc)_2_ and ligand **600b** catalyst system. The reaction goes through a high-valent Pd(IV) intermediate. This methodology is amenable to a wide range of aryl boronic acids without a large drop in enantioselectivity (84–94% *ee*).^279^

**Scheme 238:**
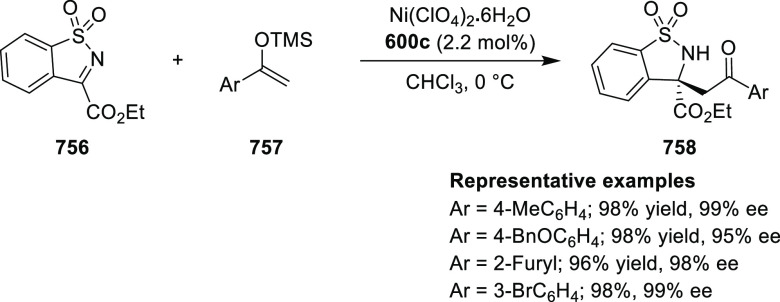
Ni-Catalyzed Asymmetric Synthesis of Benzosultam

Fu reported an asymmetric Mukaiyama–Mannich reaction of cyclic *N*-sulfonyl α-ketiminoesters **756** and silyl enol ethers **757** which synthesized a range of sultams **758** (Scheme 238). The Ni(ClO_4_)_2_·6H_2_O-**600c** complex promoted the reaction very efficiently, giving the desired product in good yields and up to 99% *ee*. The reaction appeared to tolerate most substitution on the *N*-sulfonyl α-ketiminoester substrates in high enantioselectivities. Silyl enol ethers furnished high levels of enantioselectivity regardless of the aromatic/heteroaromatic group attached (97–99% *ee*). However, the α-difluorinated acetophenone derived silyl enol ether gave the corresponding benzosultam **758** in only 21% yield with a 30% *ee* value.^280^

**Scheme 239:**
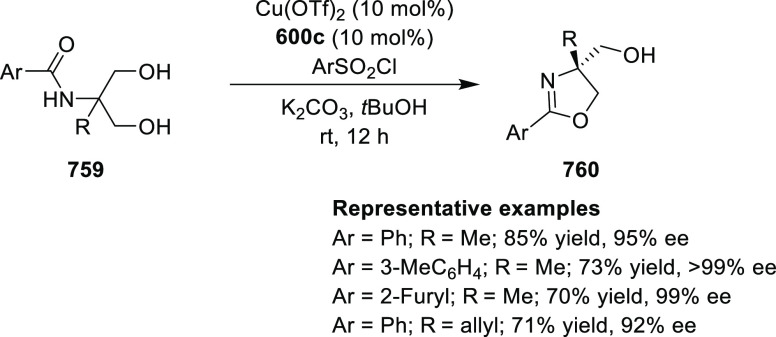
Cu-Catalyzed Synthesis of Chiral Oxazoline Derivatives

Onomura developed a catalytic synthesis of chiral oxazoline derivatives **760** via asymmetric desymmetrization of 1,3-diols **759** (Scheme 239). This method employed Cu(OTf)_2_ and ligand **600c** in *t-*butanol affording good yields and enantioselectivities of up to >99% *ee*. For the substrate scope of 2-(*N*-acylamino)-1,3-propanediols, the enantioselectivity remained high regardless of the electronic nature of Ar be it aryl or heteroaryl (87–99% *ee*). When investigating the R substituents the enantioselectivities remained high (89–97% *ee*) but the yields were lowered (20–71% yield).^281^

**Scheme 240:**
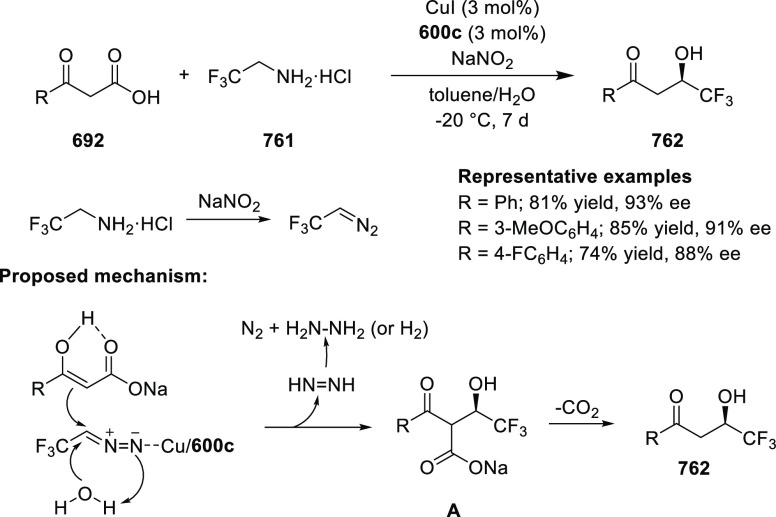
Cu-Catalyzed Denitrogenative-Decarboxylative Coupling

Ma reported a novel Cu-catalyzed one-pot cross-coupling of β-ketoacids **692** with *in situ* generated trifluorodiazoethane **761** to access trifluoromethylated aldol products **762** (Scheme 240).^282^ Initially the reaction was examined racemically with CuI being the best Lewis acid to promote the formation of the aldol product **762**. To move to an asymmetric system the reaction had to be reoptimized with the temperature being lowered and the reaction time extended. Ligand **600c** was shown to be optimal giving up to 93% *ee* in good yields. The proposed reaction mechanism involves nucleophilic attack of the enol form of the ketoacid to activated trifluorodiazoethane and simultaneous termination with water to form intermediate **A** with the release of diazene which rapidly undergoes a disproportionation reaction to form nitrogen gas and hydrazine (or H_2_). Finally, decarboxylation leads to trifluoromethylated aldol product **762**.

**Scheme 241:**
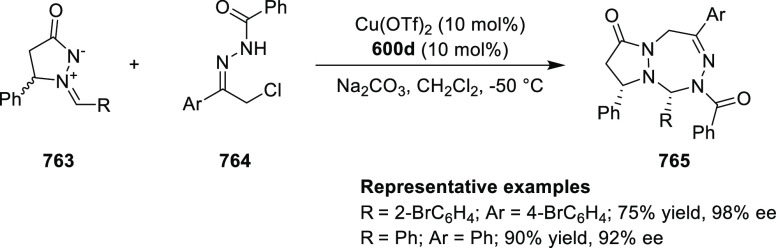
Cu-Catalyzed Cycloaddition of Azomethine Imines

Wang described an asymmetric 1,3-dipolar [3 + 4] cycloaddition of azomethine imines **763** and azoalkanes **764** (Scheme 241). Using **600d** as the ligand and Cu(OTf)_2_ as the metal, enantioselectivities up to 98% *ee* were obtained in good yields. Azomethine imines **763** bearing electron-donating, electron-neutral or electron-withdrawing groups at *para*-, *meta*-, or *ortho*-positions of the aryl ring were well tolerated, giving the corresponding products in up to 90% yield and 98% *ee*. Naphthyl, heteroaromatic, and alkyl azomethine imines were also tolerated with up to 92% yield and 96% *ee*. Unfortunately, only racemic product was obtained when an alkyl-substituted hydrazone was employed.^283^

**Scheme 242:**
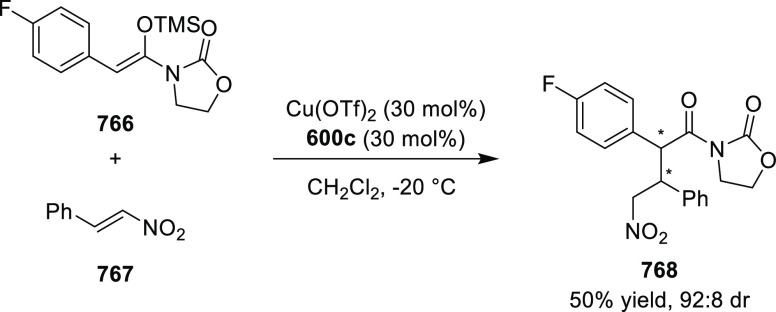
Cu-Catalyzed Asymmetric Michael Reaction

Sen detailed a diversity orientated synthesis (DOS) strategy toward the synthesis of optically active quinolizidinones, piperidinones, and pyrrolidinones. Ligand **600c** was applied in the Cu-catalyzed asymmetric Michael reaction of a silyl keteneimide **766** and nitrostyrene **767** to generate valuable intermediates in high diastereomeric ratios and good yields (Scheme 242).^284^

**Scheme 243:**
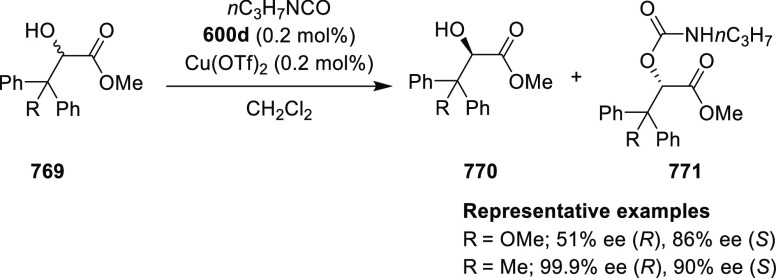
Cu-Catalyzed Kinetic Resolution of α-Tert-alkyl-α-hydroxy Esters

Ohkuma performed a kinetic resolution of sterically hindered racemic α-tert-alkyl-α-hydroxy esters **769***via* an enantiomer-selective carbamoylation reaction (Scheme 243). Using ligand **600d** and Cu(OTf)_2_ as the catalyst and carrying out the reaction at 0 °C enantioselectivities up to >99.9% *ee* and selectivity factors of up to 261 were achieved. The racemic α-tert-alkyl-α-hydroxy esters **769** reacted smoothly with 0.5 equiv of isocyanate to give the enriched starting-material **770** and carbamate **771**, respectively. Regarding substrates, most were tolerated to give high selectivity and enantioselectivity. However, it was shown that replacing the β-methyl group with a β-methoxy group had a coordinative interaction with the catalyst and this reduced the enantioselectivity to as low as 51%ee.^285^

**Scheme 244:**
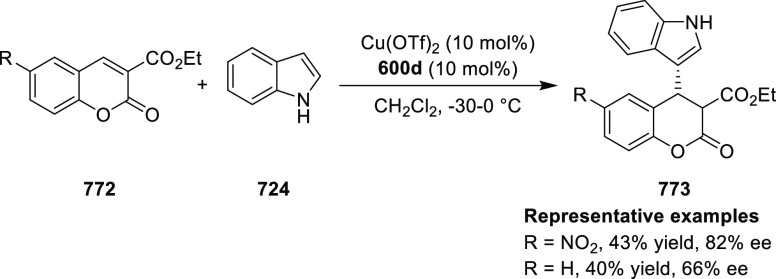
Cu-Catalyzed Asymmetric Friedel–Craft Alkylation of Coumarin-3-carbonylates

Beletskaya reported an asymmetric Friedel–Crafts reaction of indoles **724** with coumarin-3-carbonylates **772** in good yields and enantioselectivities (Scheme 244). Using ligand **600d** and Cu(OTf)_2_ up to 82% *ee* and good yields were achieved. Groups with contrasting electronics in the 5-position of indole did not influence the yield or enantioselectivity, but the introduction of a methoxy group in the 4-position halted the reaction. Electron-withdrawing groups in the 5-position of coumarin greatly increased the enantioselectivities up to 82% *ee*.^286^

**Scheme 245:**
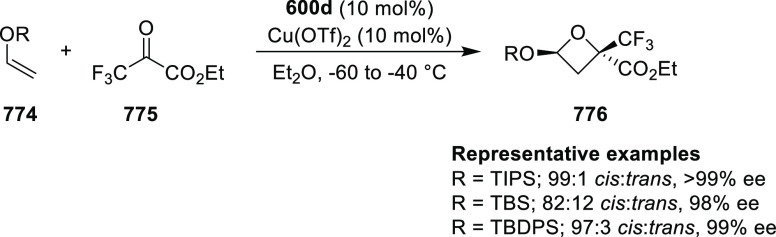
Cu-Catalyzed Asymmetric Synthesis of Oxetane Derivatives

Mikami carried out an enantioselective Cu-catalyzed [2 + 2] cycloaddition of silyl enol ethers **774** with trifluoropyruvate **775** in the synthesis of oxetane derivatives **776** (Scheme 245). Using **600d** as the ligand and Cu(OTf)_2_ as the catalyst, excellent *cis*/*trans* ratios and enantioselectivites up to >99% *ee* were achieved.^287^

As shown above parent BOX ligands have been widely applied in asymmetric catalysis to great success. While there are few shortcomings for parent BOX ligands in catalysis, structural modifications are used to modulate the bite angles, electronics and sterics of ligands to gain enhanced activity.

#### 3.1.2 Bis(oxazoline) Ligand with Modified Oxazoline Ring Substitution

**Figure 24:**
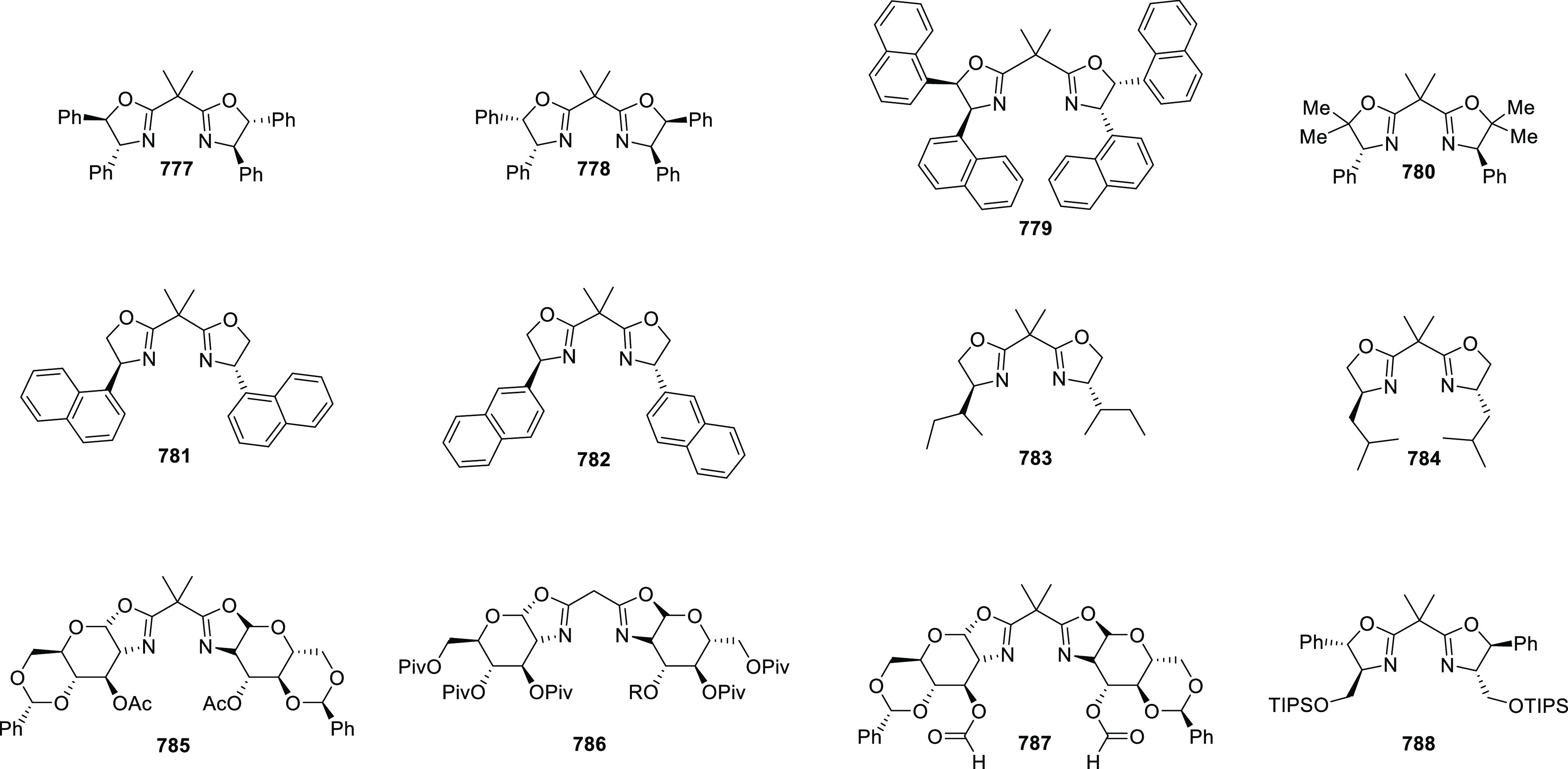
Summary of Bis(oxazoline) ligands with modified oxazoline ring substitution.

**Scheme 246:**
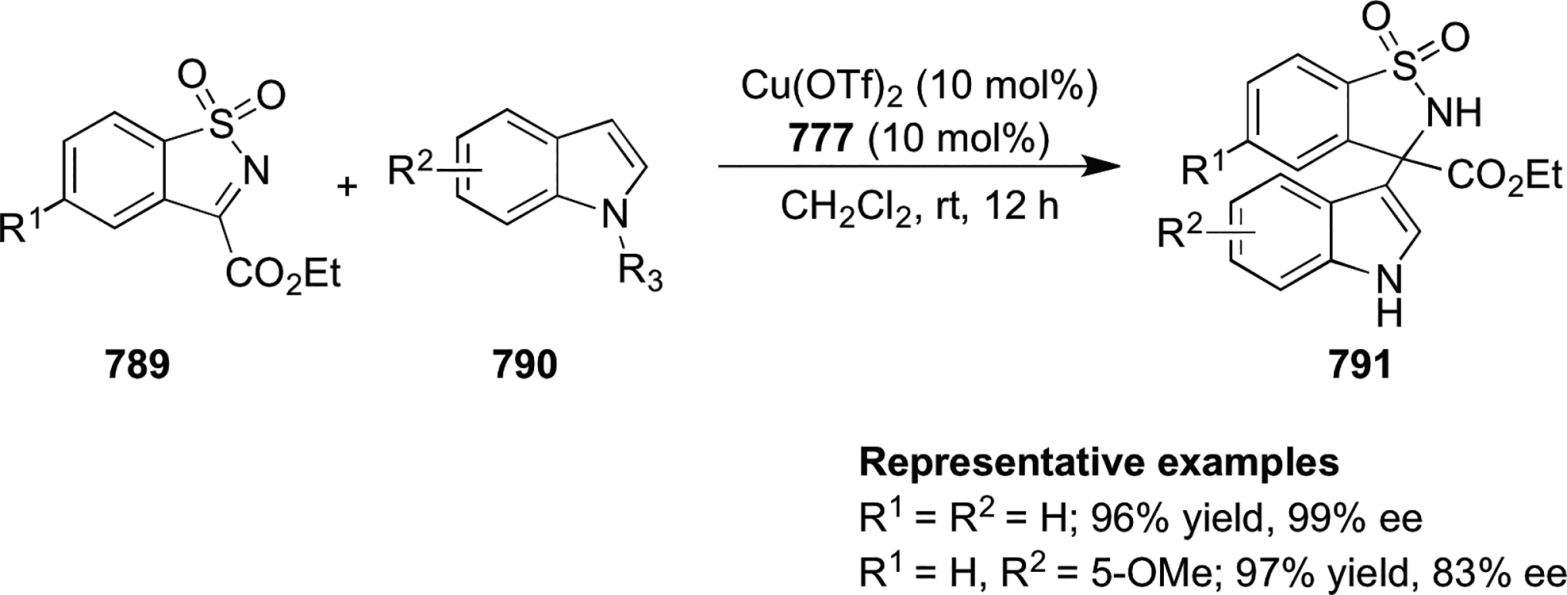
Cu-Catalyzed Friedel–Crafts Alkylation of Indoles and *N*-Sulfonyl Ketimino Esters

Jia reported the construction of cyclic indolyl α-amino esters *via* a Cu-catalyzed asymmetric Friedel–Crafts alkylation reaction (Scheme 246). Using **777** as the ligand (Figure 24) and Cu(OTf)_2_ as the Lewis acid excellent yields and up to 99% *ee* were furnished. A range of *N*-sulfonyl ketimino ester derivatives **789** and indoles **790** reacted smoothly to give the product in up to 96% yield and >99% *ee*. This process was then extended to pyrrole and *N*,*N*-dimethylaniline while maintaining enantioselectivities up to 98% *ee*. At the same time indole substrates were very well tolerated with only 5-methoxyindole causing a drop in enantioselectivity to 83% *ee*.^288^

**Scheme 247:**
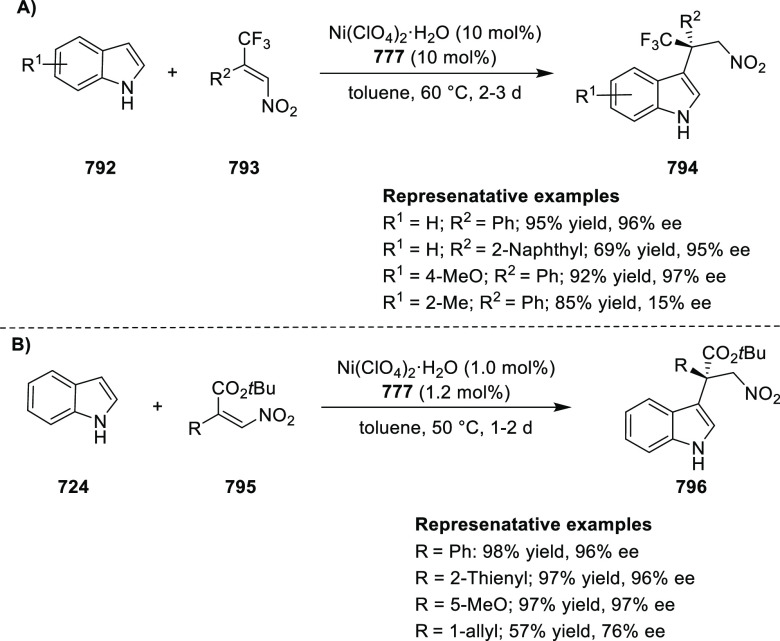
Ni-Catalyzed Asymmetric Friedel–Crafts of Indoles and β-CF_3_-β-Disubstituted Nitroalkenes

Jia once again utilized **777** as the ligand but this time in the Ni-catalyzed Friedel–Crafts alkylation of indoles **792** with β-CF_3_-β-disubstituted nitroalkenes **793** to produce trifluoromethylated all-carbon quaternary stereocenters with enantioselectivities up to 97% *ee* and good yields (Scheme 247A). Most nitroalkenes furnished the products **784** in 88–97% *ee* while benzyl substituted nitroalkenes provided the corresponding product in 78% yield and 33% *ee*. For the indole substrate scope a range of electron-donating and -withdrawing substrates were proven successful (90–97% *ee*), only methyl substitution in the 1- and 2-positions provided poor results with trace amounts of product being formed.^289^

This methodology was extended to nitroacrylates **795** and indoles **724** garnering access to β-2,2-amino acid derivatives **796** in excellent yields and enantioselectivities up to 97% *ee* (Scheme 247B). In this case the nitroacrylate scope was very tolerant with different electronic patterns being widely accepted while maintaining high enantioselectivities (88–97% *ee*). The substitution on the indole ring had a much larger effect on the enantioselectivity. Substrates with 2-Me, 1-Me or 1-allyl all proved to be challenging substrates with enantioselectivities dropping as low as 54% *ee*.^290^

**Scheme 248:**
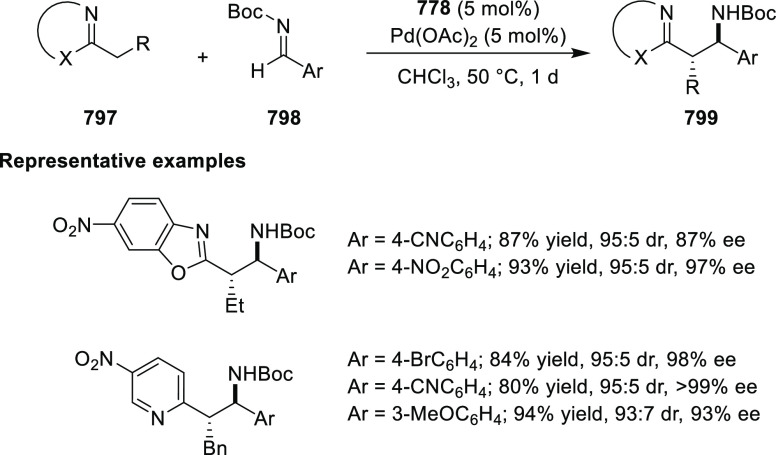
Pd-Catalyzed Asymmetric Addition to *N*-Boc Aldimines

Lam reported the use of **778** in a Pd-catalyzed asymmetric addition of alkylazaarenes **798** to *N*-Boc aldimines **797** to produce Boc-protected α-stereogenic amines **799** (Scheme 248). Addition of the nitro group was integral to activating the alkylazaarenes **797** such as nitrobenzoxazoles and 3-nitropyridines were shown to be appropriate starting materials. A range of electron-donating and withdrawing groups were well tolerated in the optimized conditions achieving good yields, diastereomeric ratios and enantioselectivities (up to 95:5 dr and >99% *ee*). However, moving the nitro groups to the 4-position of the nitrobenzooxazole was shown to be disastrous for reactivity and enantioselectivity due to co-ordination of Pd and the nitro group furnishing the product in 38% yield, 18% *ee* and 83:17 dr. Nitroalkenes **245** were shown to be good substrates with similarly impressive enantioselectivities being achieved of up to >99% *ee* and 95:5 dr.^291^

**Scheme 249:**
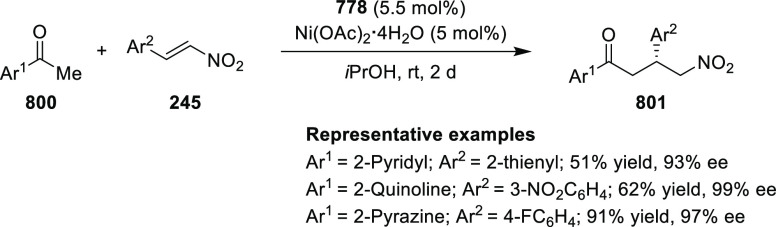
Ni-Catalyzed Asymmetric Friedel–Crafts Alkylation between Nitrostyrenes and Aryl Ketones

In a follow up publication by Lam, the scope of nitroalkenes **245** was investigated extensively, affording good yields and enantioselectivities up to 99% *ee* (Scheme 249). Regarding the 2-acetylazaarenes **800** scope, substrates containing quinoline, pyrazine, thiazole, benzothiazole, or *N*-methylimidazole all reacted smoothly to produce the desired product **801** in yields up to 95% and with high levels of enantioselectivity (up to 99%). It was also shown that β-alkyl-substituted nitroalkenes were not optimal substrates even after reoptimization with enantioselectivities reaching a maximum of 82% *ee*.^292^

**Scheme 250:**
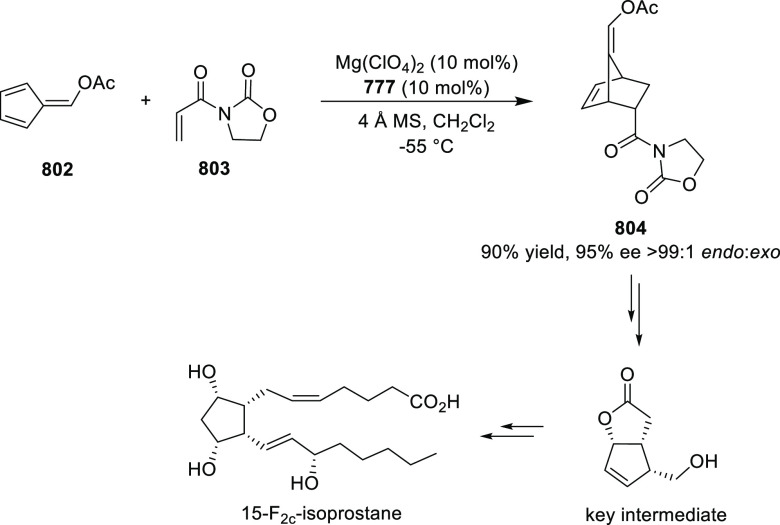
Mg-Catalyzed Asymmetric Synthesis of 15-F_2c_-Isoprostane Intermediate

Zanoni performed an enantioselective Mg-catalyzed Diels–Alder cycloaddition of acetoxyfulvene **802** and 3-acryloyl-1,3-oxazolidin-2-one **803**. The resulting cycloadduct **804** was further transformed into a key building block for the formal synthesis of isoprostanoids (Scheme 250). Using Mg(ClO_4_)_2_ and **777** the cycloadduct product was isolated in 95% *ee* and with an *endo*/*exo* ratio of >99:1.^293^

**Scheme 251:**
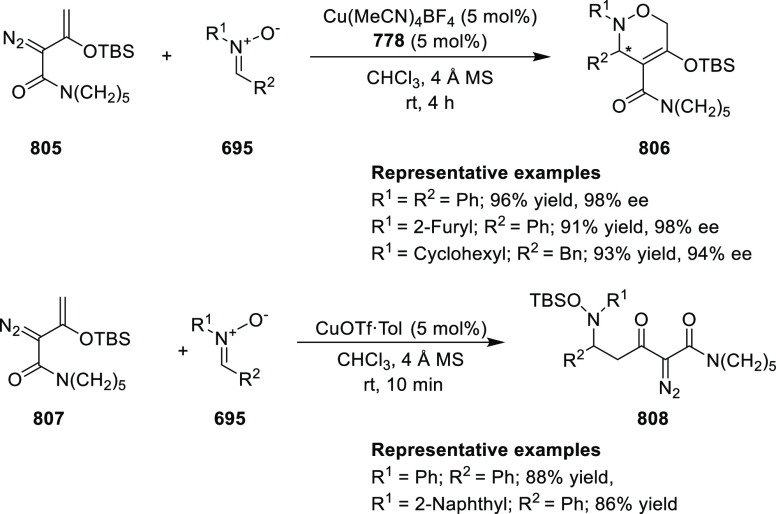
Cu-Catalyzed Asymmetric Enoldiazoacetamide Cyclization

Doyle reported a catalyst divergent reaction of enoldiazoacetamides **805** with nitrones **695** (Scheme 251). When using the achiral catalyst of Cu(OTf).Tol, the Mannich addition products **808** were observed in up to 98% yield. However, when using Cu(MeCN)_4_BF_4_ a [3 + 3] cyclization product was observed. Exploiting this catalyst system using **778** as the ligand enantioselectivities up to 98% *ee* were obtained with excellent yields. Regarding the substrate scope a range of aryl, heteroaryl and bulky alkyl substrates were tolerated with enantioselectivities and yields remaining high throughout (94–98% *ee*).^294^

**Scheme 252:**
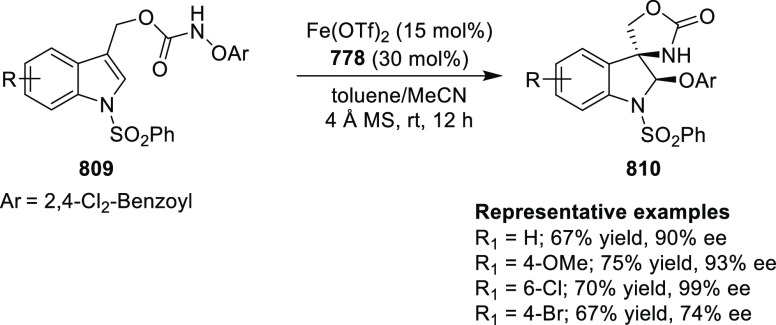
Fe-Catalyzed Asymmetric Intramolecular Aminohydroxylation

Xu developed an Fe-catalyzed asymmetric intramolecular aminohydroxylation of indoles **809** using Fe(OTf)_2_ and ligand **778** achieving up to 99% *ee* and >20:1 dr (Scheme 252). A scope of substituted indoles was attempted, and all were shown to be excellent substrates. Electron donating groups and electron withdrawing groups in the 5-, 6-, and 7-positions were well tolerated (86–99% *ee*), although 4-bromo-substituted indole caused a substantial drop in enantioselectivity to 74% *ee* and moderate yields.^295^

**Scheme 253:**
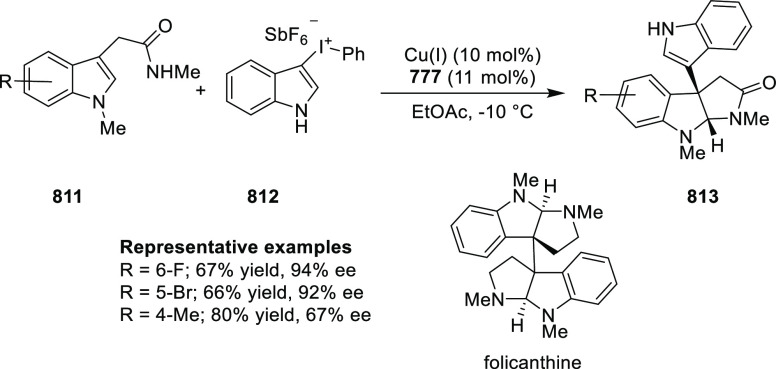
Formal Synthesis of Folicanthine

You reported the asymmetric dearomatization of indole acetamides **811** with 3-indolylphenyliodonium salts **812** catalyzed by a Cu(I) **777** complex (Scheme 253). This process was then applied by carrying out the formal synthesis of folicanthine. Using [Cu(CH_3_CN)_4_PF_6_] and ligand **777** in ethyl acetate enantioselectivities up to 94% *ee* were achieved with good yields. Electron-withdrawing groups were well tolerated regardless of the position with the 6-fluoro substrate giving 94% *ee*. It was also shown that placing a methyl group in the 4- and 5-positions led to products formed in 67% *ee* and 82% *ee*, respectively.^296^

**Scheme 254:**
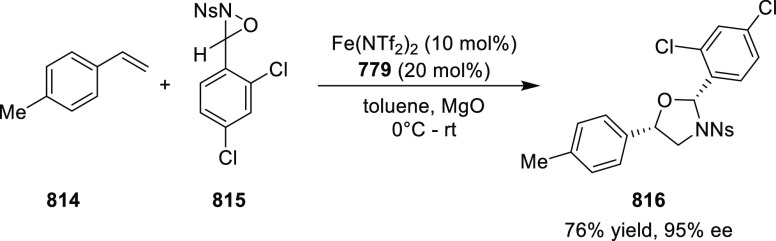
Fe-Catalyzed Aminohydroxylation of Alkenes

Yoon applied ligand **779** in the Fe-catalyzed aminohydroxylation of alkenes **814** using oxaziridines **815** (Scheme 254). Using Fe(NTf)_2_ and ligand **779** enantioselectivities up to 95% *ee* and 76% yield were obtained. A variety of aryl alkenes were tested in the reaction providing high enantioselectivities throughout with *p*-bromostyrene giving 63% yield and 85% *ee* as the lowest enantioselectivity. It was also shown that aliphatic alkenes and *trans*-β-methylstyrene were not tolerated in this reaction providing no product whatsoever.^297^

**Scheme 255:**
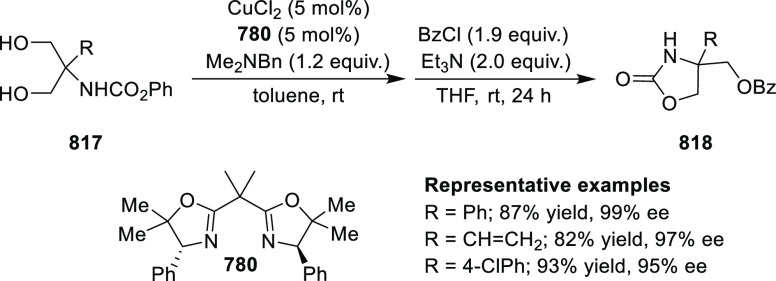
Cu-Catalyzed Synthesis of Chiral Oxazolidinones

Kang reported the dual-functional Cu-catalyzed desymmetrization and subsequent kinetic resolution of serinols **817** to produce enantioenriched oxazolidinones (Scheme 255). Using ligand **780** and a CuCl_2_ complex enantioselectivities were high across the board tolerating a range of alkyl, aryl and alkenes (94–99% *ee*).^298^

**Scheme 256:**
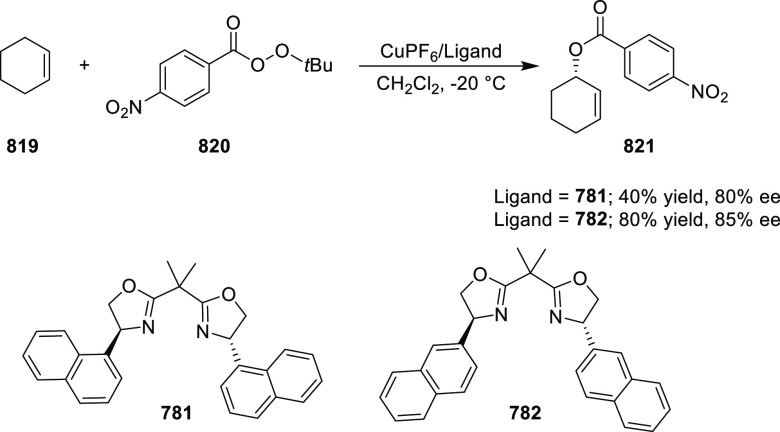
Cu-Catalyzed Allylic Oxidation of Cyclohexene

Andrus and Zhou prepared two regioisomers of naphthyl-substituted BOX ligands (**781** and **782**) and screened them in the allylic oxidation of cyclohexene **819** (Scheme 256). Using ligand **781** and CuPF_6_ the corresponding product was isolated in 40% yield and 80% *ee*, while use of ligand **782** gave the product in 80% yield and 85% *ee*.^299^

**Scheme 257:**
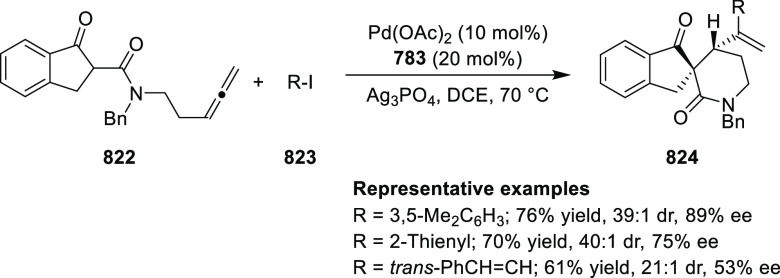
Pd-Catalyzed Asymmetric Carbocyclization Cascade

Dixon reported a Pd-catalyzed arylative and vinylative allene carbocyclization cascade. Using silver phosphate and a Pd(OAc)_2_.**783** (*s*-butyl-substituted BOX ligand), the allene-linked ketoamide **822** pro-nucleophile and aryl iodides **823** were cyclized in good yields and up to 39:1 dr and 89% *ee* (Scheme 257). While a range of substituted aryl and *cis*-styrenyl iodides were well tolerated, *trans*-styrenyl iodides were shown to cause a large drop in enantioselectivity to 61% yield, 21:1 dr, 53% *ee*.^300^

**Scheme 258:**
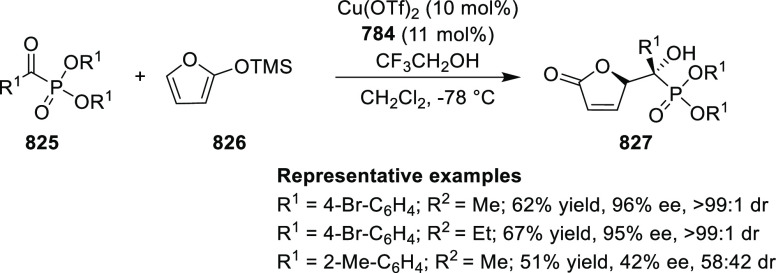
Cu-Catalyzed Asymmetric Mukaiyama Aldol Reaction of α-Keto Phosphonates

Miao developed an asymmetric Cu-catalyzed vinylogous Mukaiyama aldol reaction of α-keto phosphonates **825** and 2-TMSO furan **826** (Scheme 258). Using the ligand **784** with Cu(OTf)_2_ and 2,2,2-trifluoroethanol as an additive, the aldol products **827** were obtained in enantioselectivities up to 96% *ee* and diastereoselectivities up to 99:1 dr. It was shown that the size of the phosphonate ester **825** group had little to no effect on the enantioselectivity or diastereoselectivity as both the methyl and ethyl esters gave the same results (95% *ee* and >99:1 dr). Electron-withdrawing and electron-donating groups were shown to have no effect on the results all giving enantioselectivities in the range of 94–98% *ee*. However, phosphonates that possessed an *ortho*-methyl substituent were less reactive giving enantioselectivities as low as 42% *ee* and diastereoselectivities as low as 58:42 dr.^301^

##### 3.1.2.1 Bis(oxazoline) Ligands with Glucose Derived Oxazoline Ring Substitution

**Figure 25:**
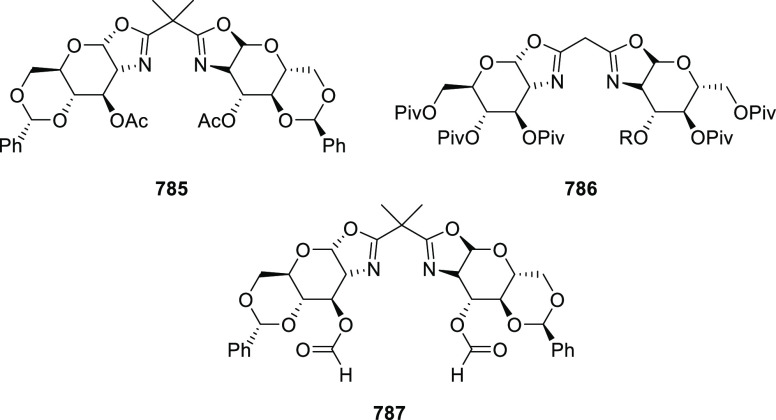
Glucose-derived BOX ligands.

**Scheme 259:**
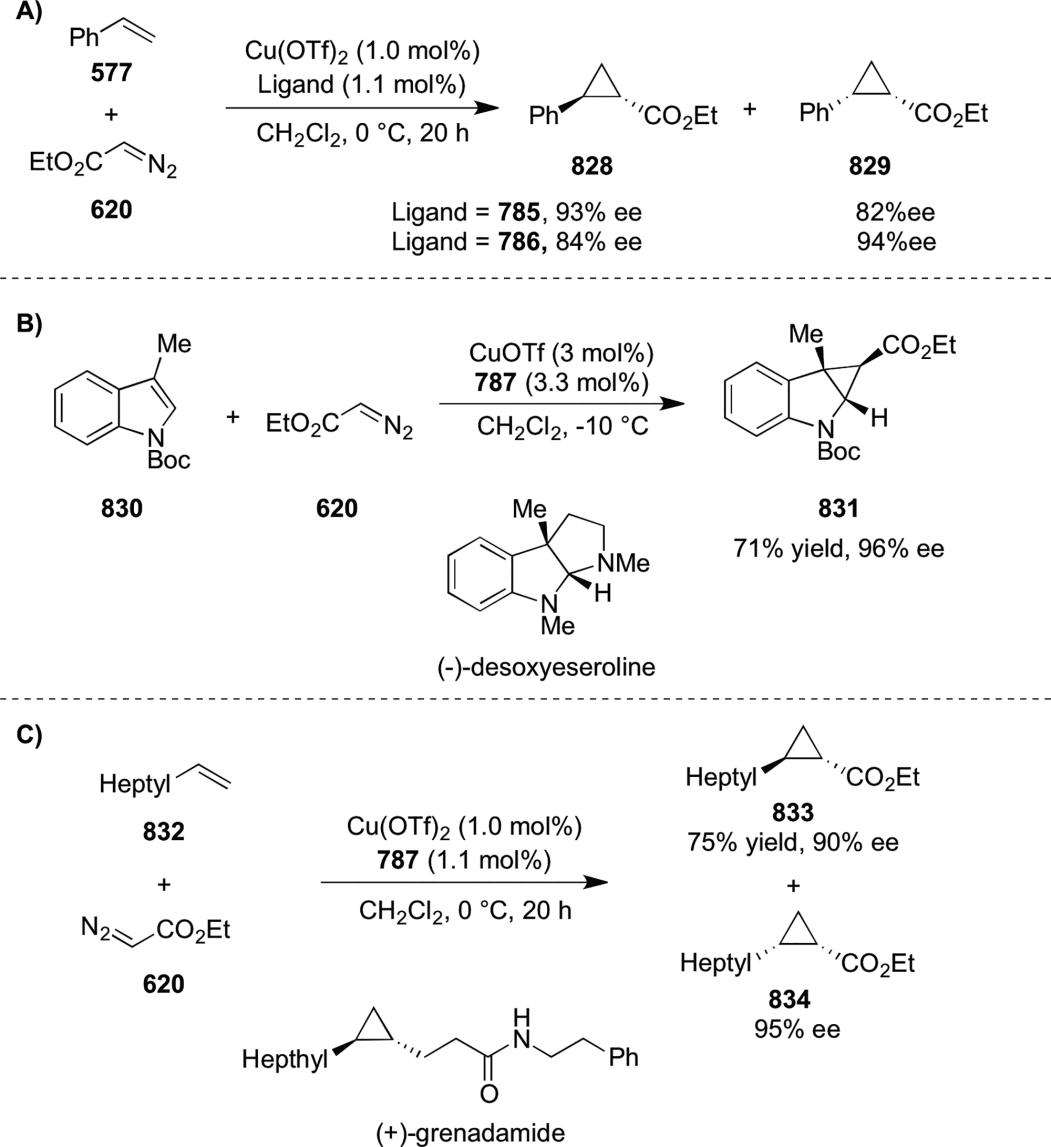
Cu-Catalyzed Asymmetric Cyclopropanation

Boysen developed a series of glucose-derived ligands (Figure 25) and applied them in Cu-catalyzed cyclopropanations. Initially both ligands **785** and **786** were applied in the cyclopropanation of styrene **577** giving up to 93% *ee* for the *trans*-product **828** and 94% *ee* for the *cis*-product **829**, respectively (Scheme 259A).^302^ Following on from this initial report, ligand **785** was subsequently applied in the cyclopropanation of **830** affording the product in up to 71% yield and 96% *ee* (Scheme 259B). The subsequent product was then applied in the synthesis of (−)-desoxyeseroline. A similar strategy was employed using ligand **787** to provide intermediate **833** in 75% yield and 90% *ee* in the synthesis of (+)-grenadamide (Scheme 259C).^303,304^

**Scheme 260:**
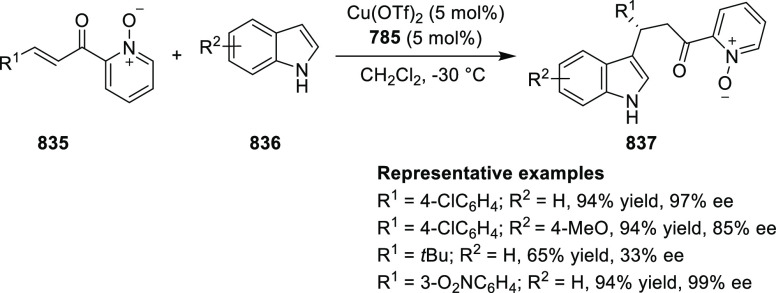
Cu-Catalyzed Asymmetric Friedel–Crafts Reaction of 2-Enoylpyridine-*N*-oxides

Reddy reported the use of ligand **785** in the Cu-catalyzed asymmetric Friedel–Crafts reaction of 2-enoylpyridine-*N*-oxides **835** with indoles **836** (Scheme 260). Using ligand **785** and Cu(OTf)_2_ enantioselectivities up to 99% *ee* were obtained in excellent yields. The indole substitution pattern had a large influence on the enantioselectivity, for example, when the electron-donating 4-methoxyindole was used the enantioselectivity dropped to 85% *ee* although the yield was still high at 94%. For the 2-enoylpyridine-*N*-oxide substrate scope it was shown the aryl and heteroaryl were suitable substrates with the enantioselectivities remaining high at 90–99% *ee*. When alkyl substituents were tested the enantioselectivity dropped dramatically with *t-*butyl affording the product in 65% yield and 33% *ee*, while the cyclohexyl-containing substrate did not give rise to product.^305^

##### 3.1.2.2 Bis(oxazoline) Ligands with Silyl-Protected Oxazoline Ring Substitution

**Scheme 261:**
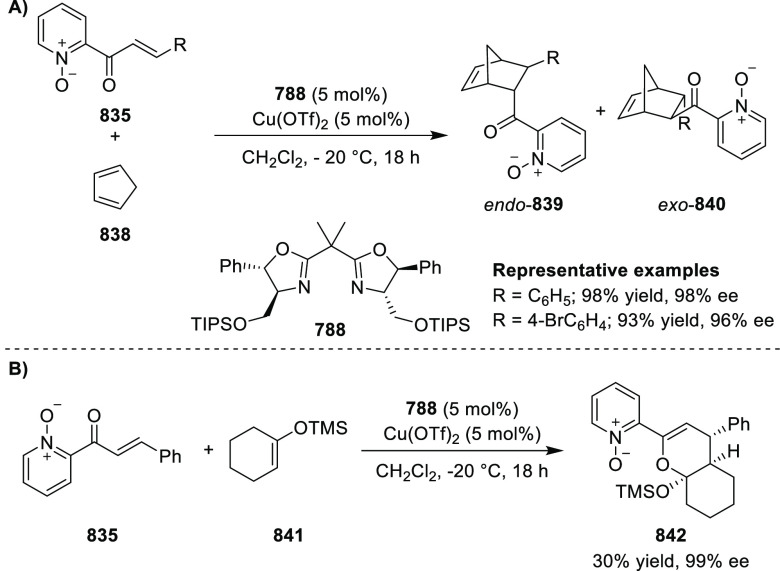
Cu-Catalyzed Cycloaddition of 2-Alkenoylpyridine-*N*-oxides and Dienes/Enol Silyl Ethers

Desimoni and Faita disclosed the Cu-catalyzed cycloaddition of 2-alkenoylpyridine-*N*-oxides **835** and dienes **838** (Scheme 261A). Using ligand **788** and Cu(OTf)_2_ excellent enantioselectivities up to 98% *ee* and *endo*:*exo* ratios up to 99:1 were observed for the products **839**/**840**. Eletron-withdrawing arylidenepyridine-*N*-oxides were also attempted and showed to have no effect on the enantioselectivity. In a subsequent publication, ligand **788** was applied in the cycloaddition between enol silyl ethers **841** and 2-alkenoylpyridine-*N*-oxides **835** forming products **842** in poor yield (30%) and with high enantioselectivity (99% *ee*) (Scheme 261B).^306,307^

The BOX ligands with modified oxazoline ring substitutions are widely applied in Lewis acid catalysis to great affect as shown above. While many of the modifications rely on costly non-natural amino alcohols more cost-effective ligands have been developed using carbohydrates for their chiral information.

#### 3.1.3 Bis(oxazoline) Ligands with Backbone Modifications

**Figure 26:**
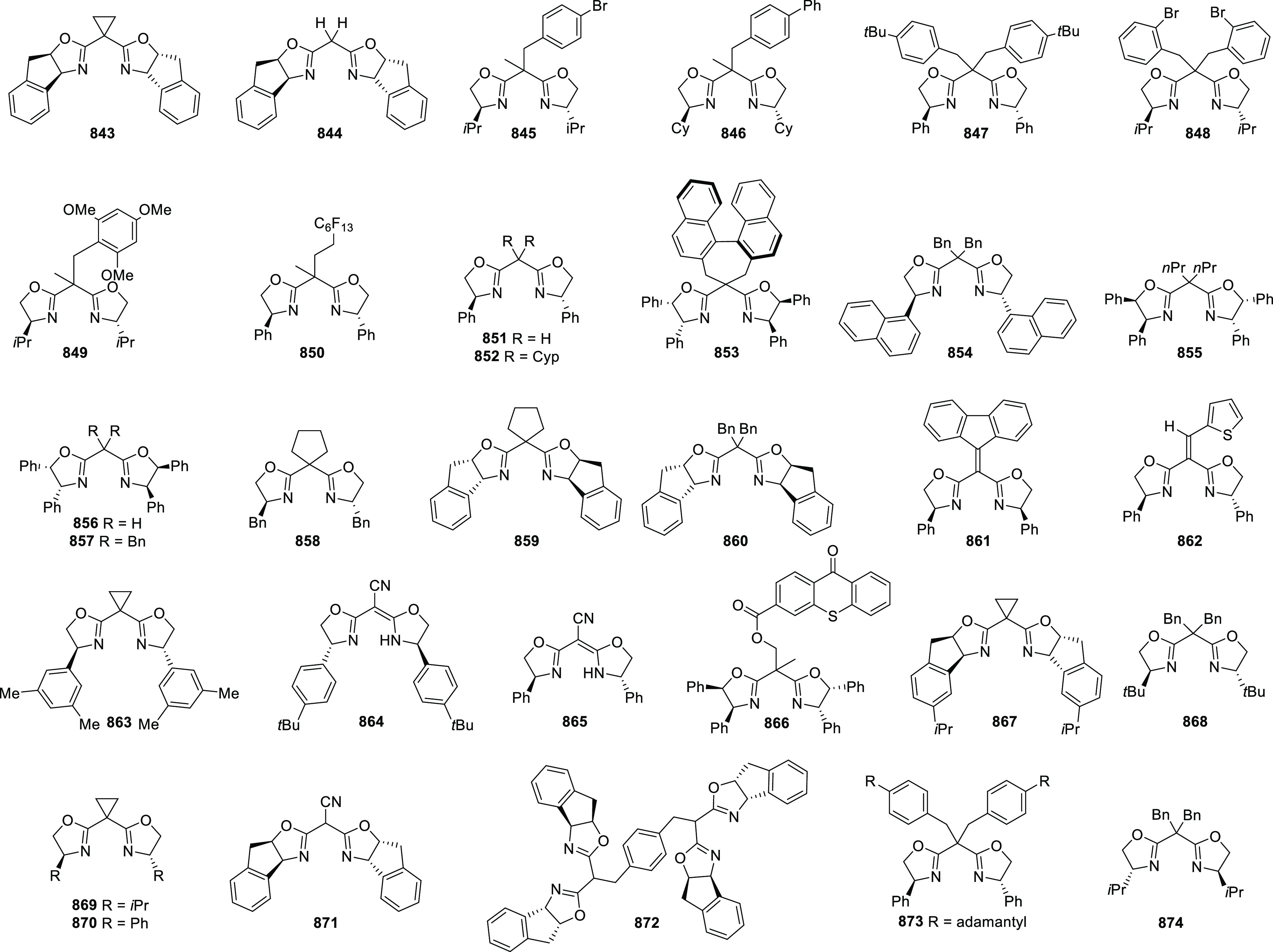
BOX ligand with backbone modifications.

**Scheme 262:**
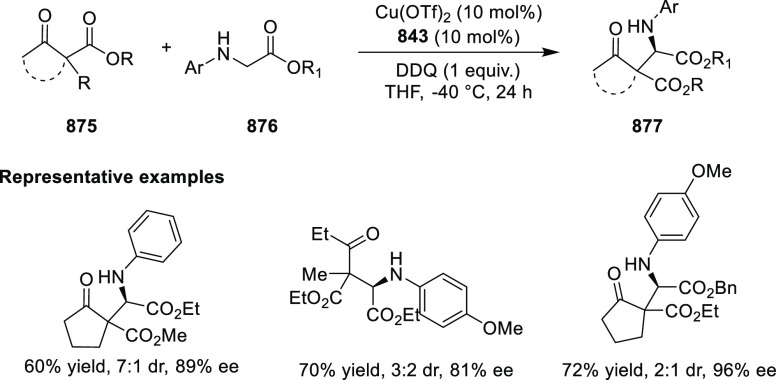
Cu-Catalyzed Asymmetric Coupling of α-Substituted-β-keto Esters and Glycine Esters

Wang reported the Cu-catalyzed asymmetric cross coupling of α-substituted-β-keto esters **875** with *N*-substituted glycine esters **876** (Scheme 262). Using a Cu(OTf)_2_ and ligand **843** complex (Figure 26) gave the product **877** in good yields and up to 96% *ee* in a 2:1 dr. A variety of α-substituted-β-keto esters **875** were screened affording the products **877** in up to 72% yield and 96% *ee*. Acyclic α-substituted-β-keto esters however led to a drop in enantioselectivity as low as 81% *ee* in a 3:2 dr.^308^

**Scheme 263:**
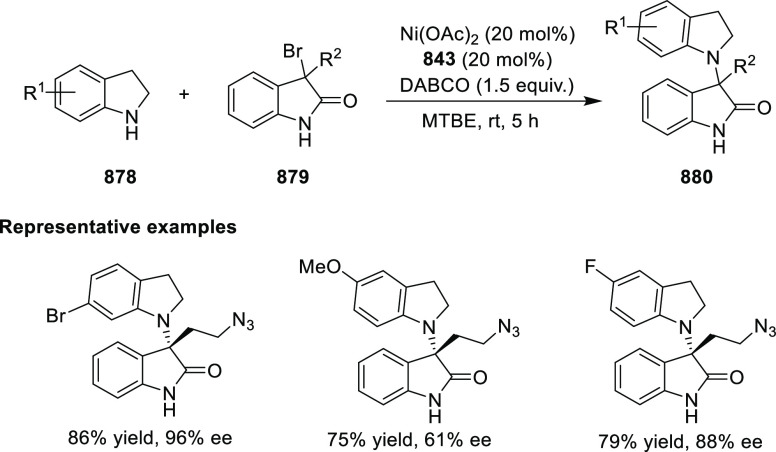
Ni-Catalyzed Asymmetric Amination of 3-Bromooxindoles

**Figure 27:**
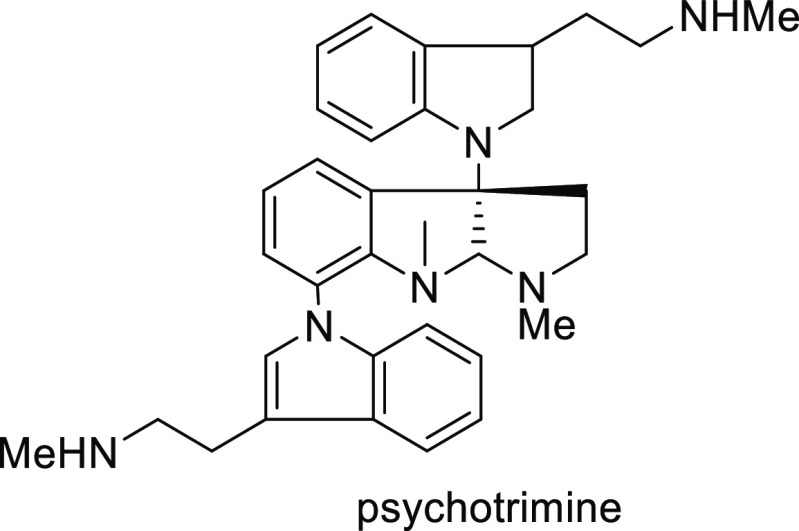
Psychotrimine.

Wang developed a Ni-catalyzed asymmetric amination of 3-bromooxindoles **879** with indolines **878** (Scheme 263), a synthetic methodology that was then applied in the formal synthesis of Psychotrimine (Figure 27). Using ligand **843** and Ni(OAc)_2_ as the optimized catalyst complex enantioselectivities up to 96% *ee* were obtained. Indolines and oxindoles with a variety of halides in the 4-, 5-, and 6-positions afforded the product in high enantiosleectivities (86–96% *ee*). However, placing electron-donating substituents (methyl or methoxy) in the 5-position led to a significant drop in the enantioselectivity, to 61% *ee* and 74% *ee*, respectively.^309^

**Scheme 264:**
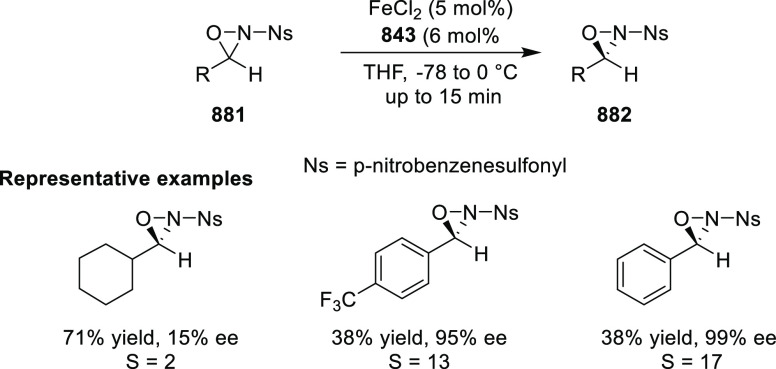
Fe-Catalyzed Kinetic Resolution of *N*-Sulfonyl Oxaziridines

Yoon applied ligand **850** in the Fe-catalyzed kinetic resolution of *N*-sulfonyl oxaziridines **881** achieving up to 99% *ee* and with selectivity factors up to 30 (Scheme 264). A variety of C-aryl oxaziridines **881** were screened in this reaction with most substrates obtaining >90% *ee*. A major limitation of this method was that C-alkyl oxaziridines were not compatible with 15% *ee* being the highest enantioselectivity obtained.^310^

**Scheme 265:**
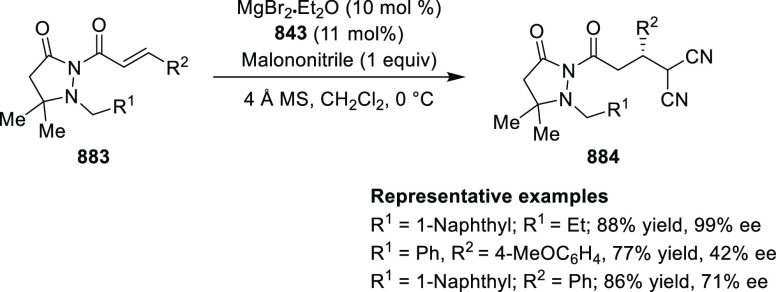
Mg-Catalyzed Asymmetric Conjugate Addition to Pyrazolidone Derivatives

Sibi developed a Mg-catalyzed asymmetric conjugate addition of malononitrile to pyrazolidone-derived enoates **883** (Scheme 265). Using MgBr_2_.Et_2_O and ligand **843** as the catalyst enantioselectivites up to 99% *ee* and good yields were obtained. It was also shown that when 4 Å MS were taken out of the optimized reaction conditions the enantioselectivity dropped as low as 44% *ee*. It was shown that the substituents on the enone component of the substrate greatly affected the enantioselectivity with electron donating *p-*methoxyphenyl groups resulting in enantioselectivities as low as 42% *ee*.^311^

**Scheme 266:**
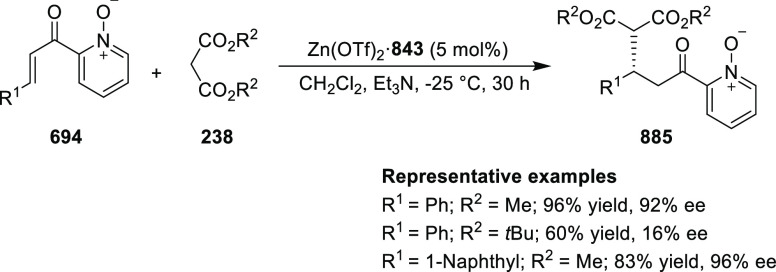
Zn-Catalyzed Asymmetric Michael Addition of Malonates to 2-Enoylpyiridine *N*-Oxides

Singh reported an asymmetric Zn-catalyzed Michael addition of malonates **238** to 2-enoylpyiridine *N*-oxides **694** (Scheme 266). Using Zn(OTf)_2_.**843** as the catalyst good yields and enantioselectivities of up to 96% *ee* were observed. It was shown that bulky malonates led to low enantioselectivities, such as di-*t*-butylmalonate which furnished the product in 60% yield and 16% *ee*. Interestingly, while many aromatic and heteroaromatic β-substituted 2-enoylpyridine *N*-oxides were suitable substrates in this reaction, the sterically hindered *t*-butyl β-substituted 2-enoylpyridine *N*-oxide did not give rise to any product.^312^

**Scheme 267:**
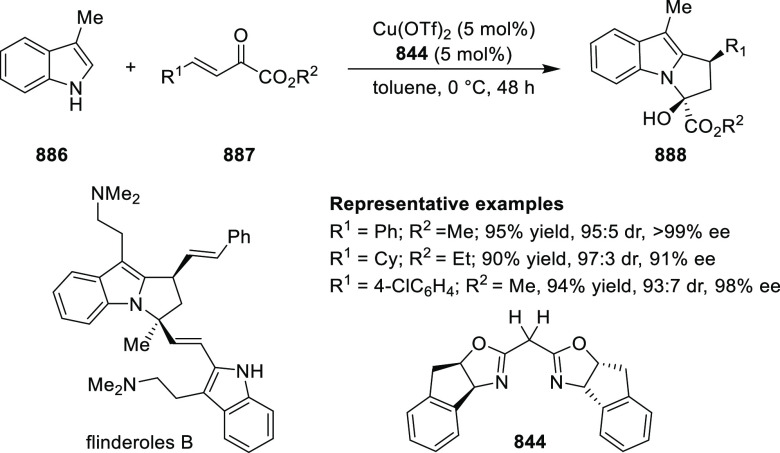
Cu-Catalyzed Asymmetric Friedel–Crafts Alkylation/*N*-Hemiacetalization Cascade Reaction of Indoles

Xiao developed a Cu-catalyzed Friedel–Crafts alkylation/*N*-hemiacetalization cascade reaction of indoles **886** with β,γ-unsaturated α-keto esters **887** (Scheme 267). Using Cu(OTf)_2_ and the indanol-derived chiral ligand **844** as the catalyst enantioselectivities up to >99% *ee* and 97:3 dr were obtained. This methodology was then applied in the formal synthesis of flinderole B analogues. A series of β,γ-unsaturated α-keto ester substrates **887**, regardless of bulkiness or electronic-nature, resulted in high levels of enantioselectivities of 91 to >99% *ee*.^313^

##### 3.1.3.1 Bis(oxazoline) Ligands with Benzyl Substituted Backbone Modifications

**Figure 28:**
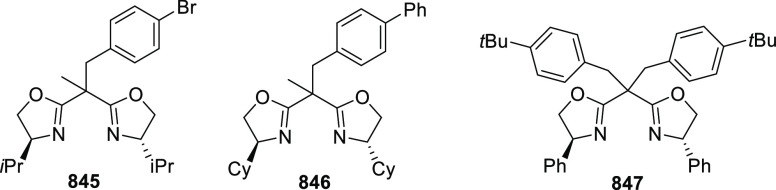
BOX ligands with substituted benzyl backbones.

**Scheme 268:**
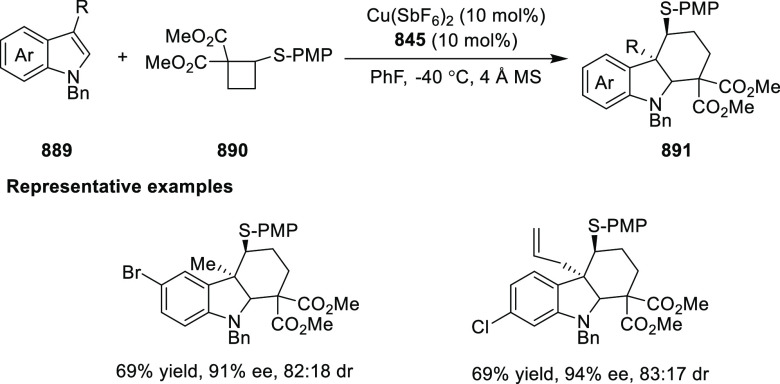
Cu-Catalyzed Asymmetric Synthesis of Cyclohexyl-Fused Indolines

**Figure 29:**
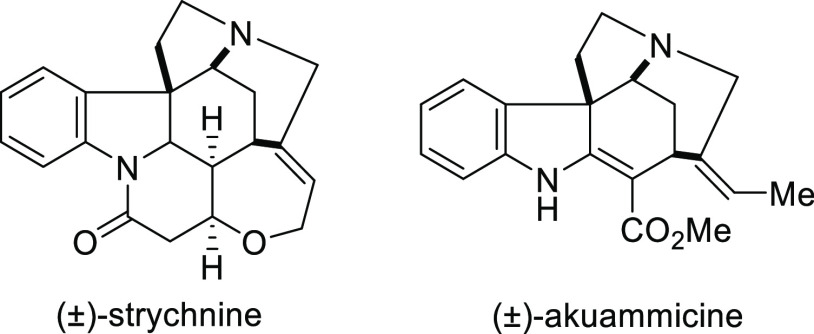
(±)-Strychnine and (±)-akuammicine.

Tang developed a Cu-catalyzed [4 + 2] cycloaddition of indoles **889** and cyclobutanes **890** to yield cyclohexyl-fused indolines **891** (Scheme 268). This reaction was initially developed using Cu(SbF_6_)_2_ in CH_2_Cl_2_ in a racemic manner and was then further developed using the chiral ligand **845** (Figure 28) affording up to 94% *ee* and 83:17 dr improving upon the parent BOX ligand **600b**. This methodology was then applied to the formal total synthesis of (±)-strychnine and the total synthesis of (±)-akuammicine (Figure 29).^314^

**Scheme 269:**
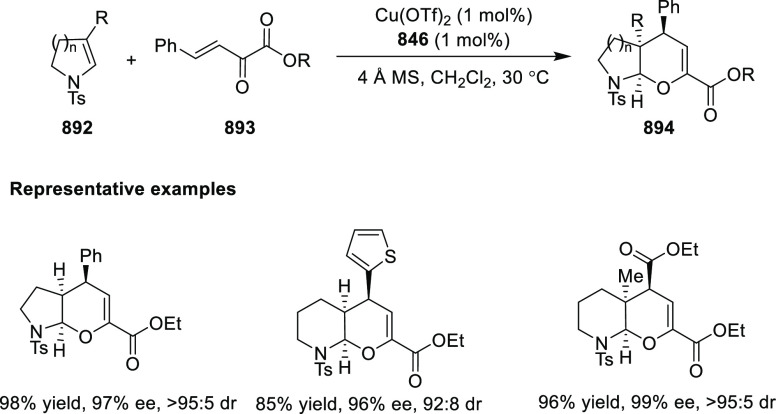
Cu-Catalyzed Asymmetric Synthesis of *N*,*O*-Acetals

Tang applied ligand **846** in the Cu-catalyzed enantioselective synthesis of bicyclic *N*,*O*-acetals **892** via a hetero-Diels–Alder reaction (Scheme 269). Catalyzed by a Cu(OTf)_2_.**846** complex, β,γ-unsaturated α-keto esters **893** reacted with cyclic enamines **892** efficiently, affording the desired products **894** in excellent yields with excellent stereoselectivities (up to 99% yields up to >95:5 dr; and 95–99% *ee*). Aryl and heteroaryl moieties were tolerated in the β,γ-unsaturated α-keto ester **893** substrate scope without any deterioration in the enantioselectivity observed (95–99% *ee*).^315^

**Scheme 270:**
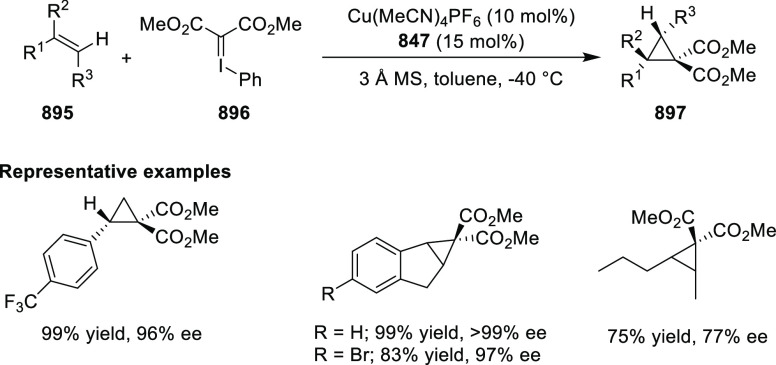
Cu-Catalyzed Enantioselective Cyclopropanation of Alkenes

Tang applied ligand **847** in the asymmetric Cu-catalyzed cyclopropanation of alkenes **895** to yield the 1,1-cyclopropane diesters **897** (Scheme 270). Using phenyliodonium ylides **896** as the carbene transfer reagent, [Cu(CH_3_CN)_4_]PF_6_ as the catalyst and **847** as the chiral ligand, enantioselectivities of up to >99% *ee* were observed. A variety of terminal alkenes were very well tolerated in the reaction most of which gave enantioselectivities in the range of 87–96% *ee*. When nonterminal alkenes were subjected to the optimized reaction conditions, 5-, 6- and 7-membered cyclic alkenes yielded the product in up to >99% *ee*. One limitation of this reaction appeared to be aliphatic-substituted alkenes afforded the product in enantioselectivities as low as 77% *ee*. In 2018 Tang further developed the asymmetric cyclopropanation of trisubstituted olefins using modified bis(oxazoline) ligands.^316,317^

**Scheme 271:**
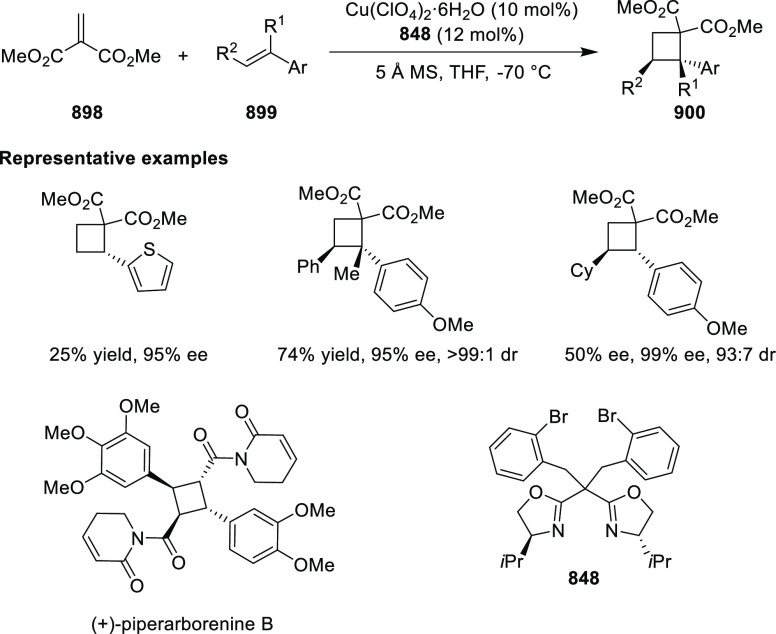
Cu-Catalyzed Asymmetric Synthesis of Cyclobutanes

Tang also reported the Cu-catalyzed asymmetric construction of cyclobutanes **900** from methylidenemalonate **898** and styrenes **899** (Scheme 271). Using ligand **848** and Cu(ClO_4_)_2_·6H_2_O as the catalyst, enantioselectivities up to >99% *ee* and >99:1 dr were obtained. A range of styrenes **899** (aryl and heteroaryl) were well tolerated with all enantioselectivities observed ranging between 95 and 99% *ee*. This methodology was then applied to the synthesis of (+)-piperarborenine B a potential anticancer agent.^318^

**Scheme 272:**
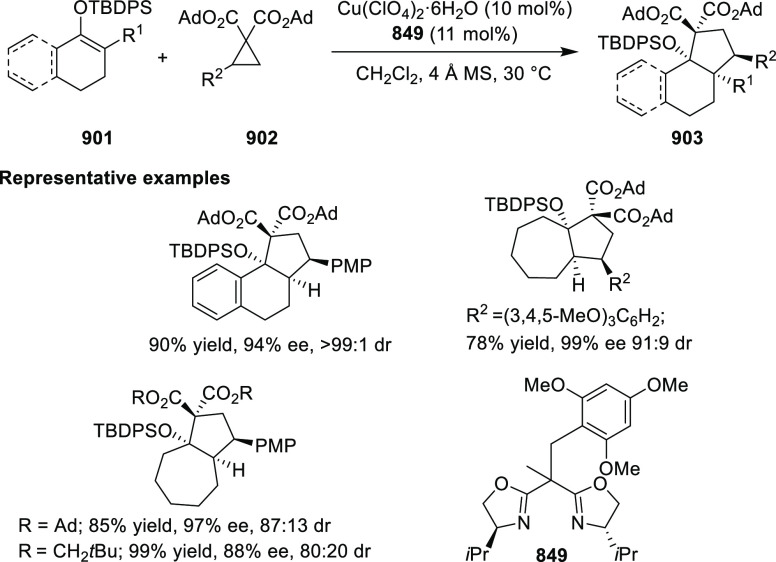
Cu-Catalyzed [3 + 2] Annulation of Cyclic Enol Silyl Ethers and Cyclopropanes

Building on this work, Tang developed a Cu-catalyzed enantioselective [3 + 2] annulation of cyclic enol silyl ethers **901** and cyclopropanes **902** (Scheme 272). Using a catalyst derived from Cu(ClO_4_)_2_·6H_2_O and ligand **849**, enantioselectivities of up to 99% *ee* and >99:1 dr were observed. Bulky adamantyl groups on the cyclopropyl esters were deemed necessary as less bulky ester groups resulting in lower enantioselectivities (94% *ee*). A range of electronically variable cyclopropanes were tested with optimized conditions showing no significant drop in enantioselectivity (91–99% *ee*).^319^

**Scheme 273:**
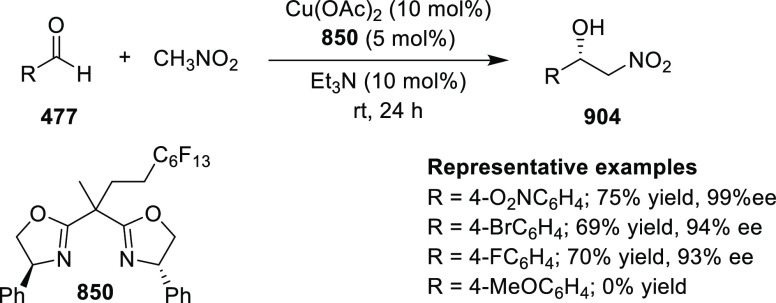
Cu-Catalyzed Asymmetric Henry Reaction

**Scheme 274:**
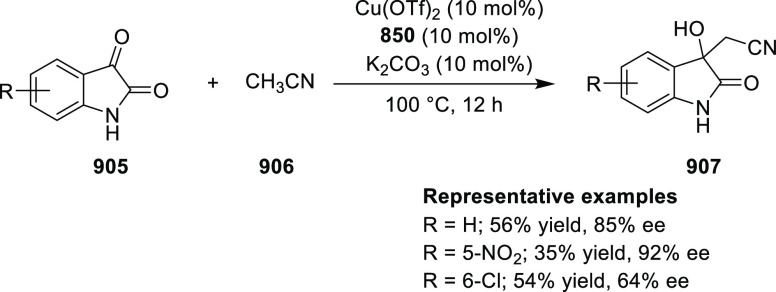
Cu-Catalyzed Asymmetric Synthesis of 3-Hydroxy-2-oxindoles

Cai utilized ligand **850**, a fluorinated derivative of ligand **600c**, in the Henry reaction between aldehydes **477** and nitromethane (Scheme 273). Using Cu(OAc)_2_ and ligand **850** enantioselectivities of up to 99% *ee* were achieved. While most aldehydes **477** performed well in the catalysis (90–99% *ee*), electron rich aldehydes such as *p*-methoxybenzaldehyde provided no product whatsoever. Cai further extended this methodology to the Cu-catalyzed asymmetric addition of acetonitrile **906** to isatins **905** to produce a series of 3-hydroxy-2-oxindoles **907** (Scheme 274). Using Cu(OTf)_2_ and ligand **850** enantioselectivities up to 92% *ee* were obtained. Regarding the substrate scope placing the electron-withdrawing chloro-group in the 6-position of the isatin led to lower enantioselectivities of 64% *ee*.^320,321^

**Scheme 275:**
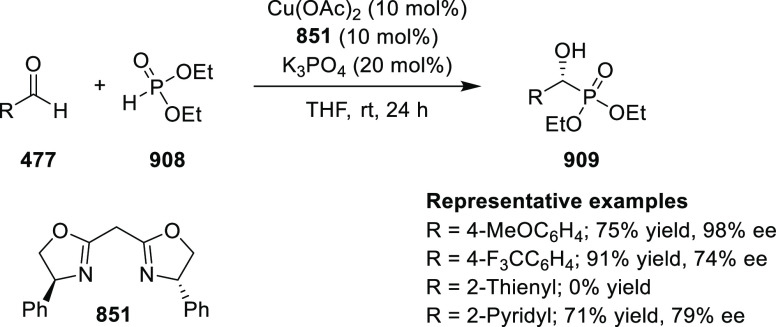
Cu-Catalyzed Asymmetric Hydrophosphonylation of Aldehydes

Cai reported the use of ligand **851** in the Cu-catalyzed hydrophosphonylation of aldehydes **477** to give the corresponding products **909** in good yields and with enantioselectivities of up to 98% *ee* (Scheme 275). Various electron-withdrawing and donating aldehydes **477** were screened in this reaction and these formed the products **909** in moderate to high levels of enantioselectivity (74–98% *ee*). The highly electron-withdrawing *p*-trifluoromethylbenzaldehyde gave the lowest enantioselectivity with 74% *ee* albeit in good yields (91% yield). Heteroaromatic systems largely were not compatible in this reaction with 2-thienyl- and 2-furyl-aldehydes not affording any product; the use of pyridine-2-carboxyladehyde as substrate was more successful, with the product formed in 71% yield albeit with modest enantioselectivity (79% *ee*).^322^

**Figure 30:**
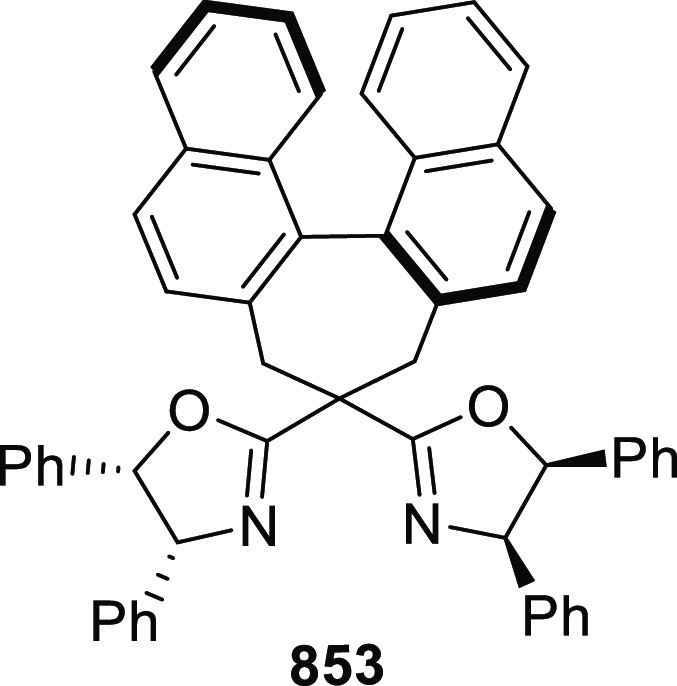
BINOL-derived BOX ligand **853**.

**Scheme 276:**
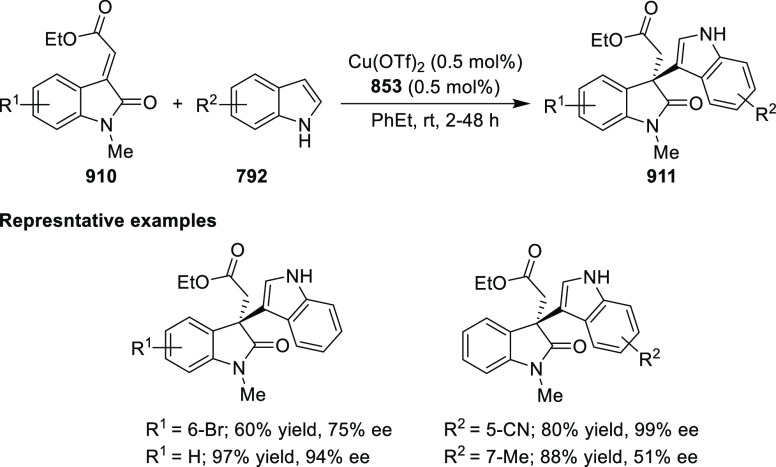
Cu-Catalyzed Asymmetric Friedel–Crafts Alkylation of Indoles and Isatin-Derived β,γ-Unsaturated α-Keto Esters

Wang reported a Cu-catalyzed Friedel–Crafts reaction of indoles **792** with isatin-derived β,γ-unsaturated α-keto esters **910** (Scheme 276). Using the BINOL-derived ligand **853** (Figure 30), the products **911** were formed in in good yields and enantioselectivities of up to 99% *ee* while using relatively low catalyst loadings of 0.5 mol %. Regarding the scope of this reaction a wide variety of isatin substrates **910** were well tolerated with most products being formed in 82–99% *ee*. Interestingly 6-bromoisatin gave rise to the corresponding product in 60% yield and a low enantioselectivity of 75% *ee*. The scope for indole substrates **792** was similarly impressive with a variety of functional groups being tolerated in good to excellent levels of enantioselectivity (82–99% *ee*). 7-Methylindole however formed the product in 88% yield and 51% *ee*.^323^

**Scheme 277:**
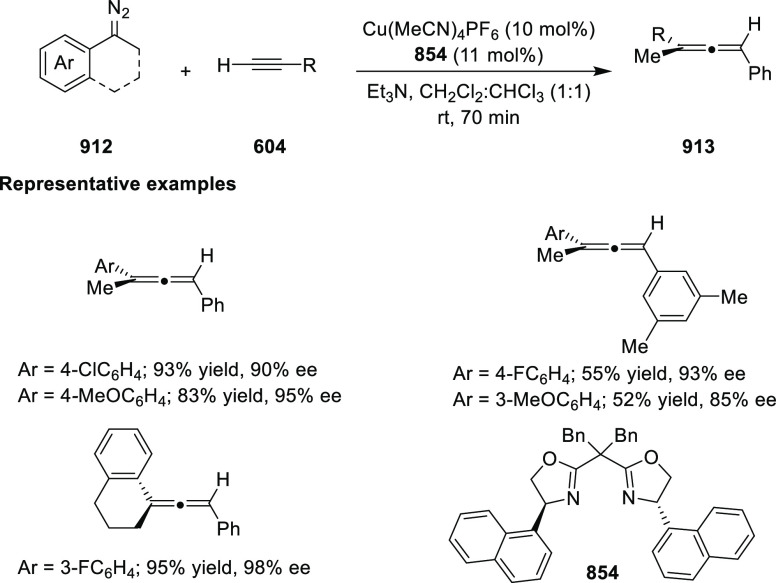
Cu-Catalyzed Asymmetric Synthesis of Trisubstituted Allenes

Wang developed an enantioselective synthesis of trisubstituted allenes **913** from a Cu-catalyzed cross-coupling of diazoalkanes **912** and terminal alkynes **604** (Scheme 277). The optimal catalyst complex was a combination of Cu(MeCN)_4_PF_6_ and the naphthyl-containing ligand **854** which gave the product in good yields and up to 98% *ee*. A series of aryldiazoalkanes **912** bearing electron-deficient or -rich aromatic substituents were smoothly reacted with phenylacetylene to give the corresponding trisubstituted allenes **913** in high yields (78–95% yield) and very high levels of enantioselectivity (84–95% *ee*). The scope of the terminal alkynes afforded the product in good enantioselectivities (92–98% *ee*) although the yields were lowered (46–60%)^324^

##### 3.1.3.2 Bis-Phenyl BOX Ligands with Backbone Modifications

**Figure 31:**
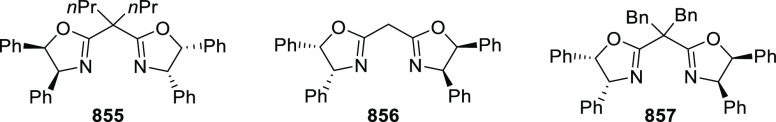
Bis-phenyl BOX ligands with backbone modifications.

**Scheme 278:**
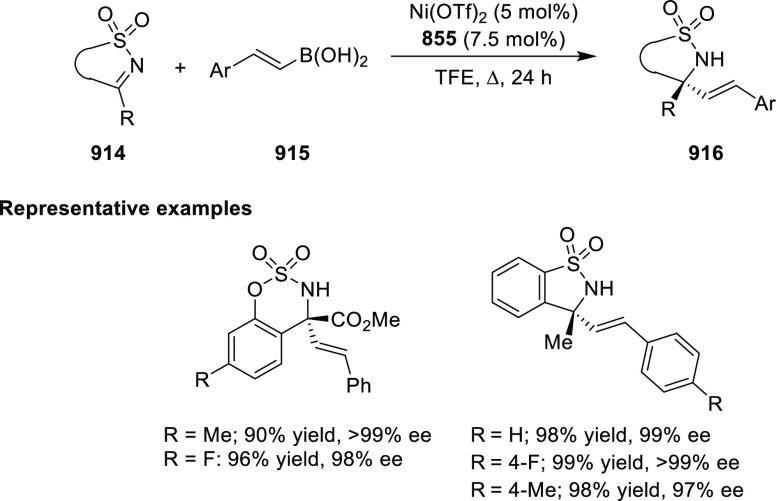
Ni-Catalyzed Asymmetric Alkenylation of Ketimines

**Scheme 279:**
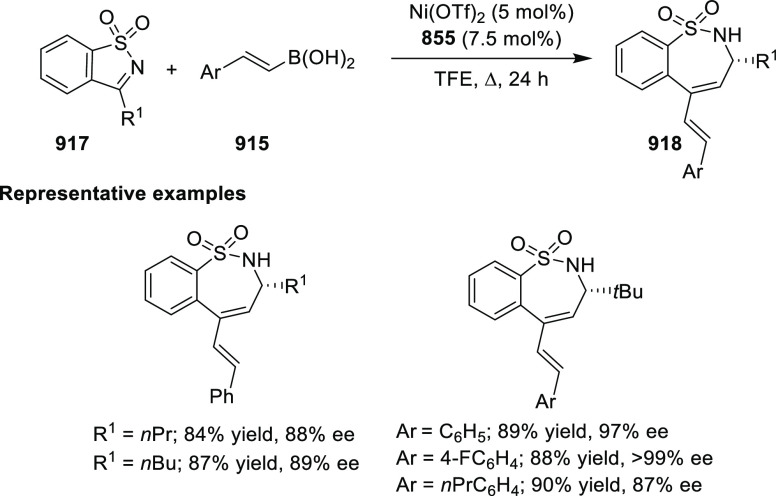
Ni-Catalyzed Asymmetric Ketimine Alkenylation/Ring-Expansion

Zhang reported the asymmetric Ni-catalyzed alkenylation of ketimines **914** using alkenylboronic acids **915** (Scheme 278). The optimal catalyst combination of ligand **855** (Figure 31) and Ni(OTf)_2_ proved to be an extremely effective system providing the product in yields up to >99% and enantioselectivities of up to >99% *ee*. The initial ketimine **914** scope gave high to excellent enantioselectivities irrespective of the nature of the ketimine (88 to >99% *ee*). The alkenylboronic acid **915** scope showed tolerance of both alkyl and styrenylboronic acids with enantioselectivities remaining very high (93% to >99% *ee*). This methodology was then expanded to the π-conjugation-controlled site-selective asymmetric ketimine-alkenylation/ring-expansion (Scheme 279). Once again, this methodology proved robust with routinely high enantioselectivities being obtained (up to >99% *ee*).^325^ This methodology was further employed in the allylation of cyclic ketimines.^326^

**Scheme 280:**
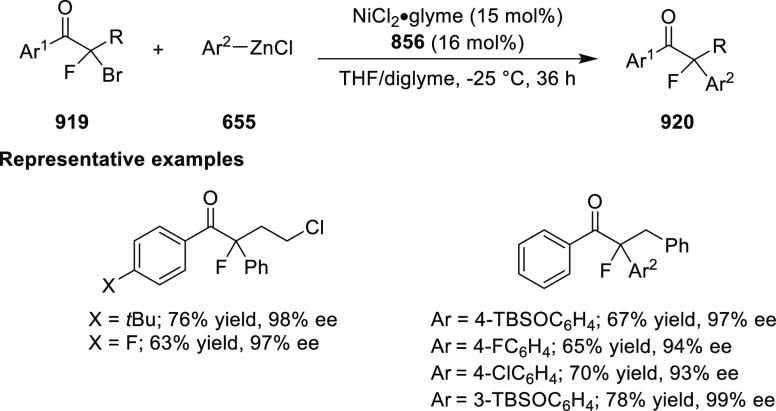
Ni-Catalyzed Asymmetric Negishi Cross-Coupling of α,α-Dihaloketones

Fu reported the development of an asymmetric Ni-catalyzed Negishi cross-coupling of α,α-dihaloketones **919** to give the corresponding products **920** containing quaternary-fluorinated stereocenters (Scheme 280). Using NiCl_2_·glyme and ligand **856** enantioselectivities of up to 99% *ee* were obtained. The structure of the ketone **919** had no major effect on the enantioselectivity with most substrates furnishing the product in high levels of enantioselectivities (92% to 98% *ee*). In a similar fashion the nature of the nucleophilic zinc reagent **655** had little impact on the enantioselectivity, all giving high enantioselectivities (91 to 99% *ee*). This methodology was further applied by Futo in the Negishi phenylation of racemic α-bromonitriles and the Kumada cross-coupling of racemic α-bromoketones^327−329^

**Scheme 281:**
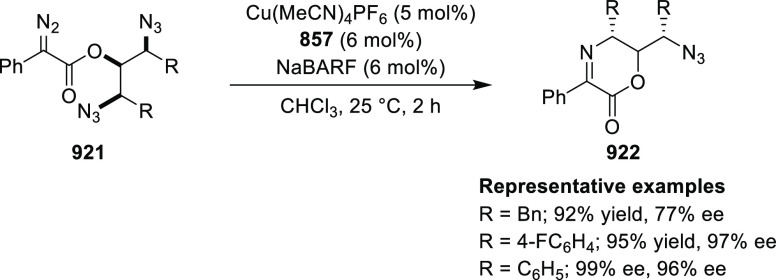
Cu-Catalyzed Desymmetrisation of *meso*-α,α′-Diazido Alcohols

An asymmetric Cu-catalyzed desymmetrization of *meso*-α,α′-diazido alcohols **921** to produce cyclic α-imino esters **922** was reported by Gu (Scheme 281). The optimized conditions utilized CuPF_6_(MeCN)_4_ and ligand **857** as the catalyst complex and NaBARF as the noncoordinating counterion. This combination furnished the corresponding products **922** in up to 97% *ee*, which was carried out on gram scale. Aryl-substituted *meso*-α,α′-diazido alcohol substrates **921** afforded the products in very high levels of enantioselectivity (92 to 97% *ee*). When alkyl-containing substrates were used, the enantioselectivity dropped as low as 77% *ee*.^330^

##### 3.1.3.3 Bis(oxazoline) Ligands with Cyclopentyl Backbone Modifications

**Figure 32:**
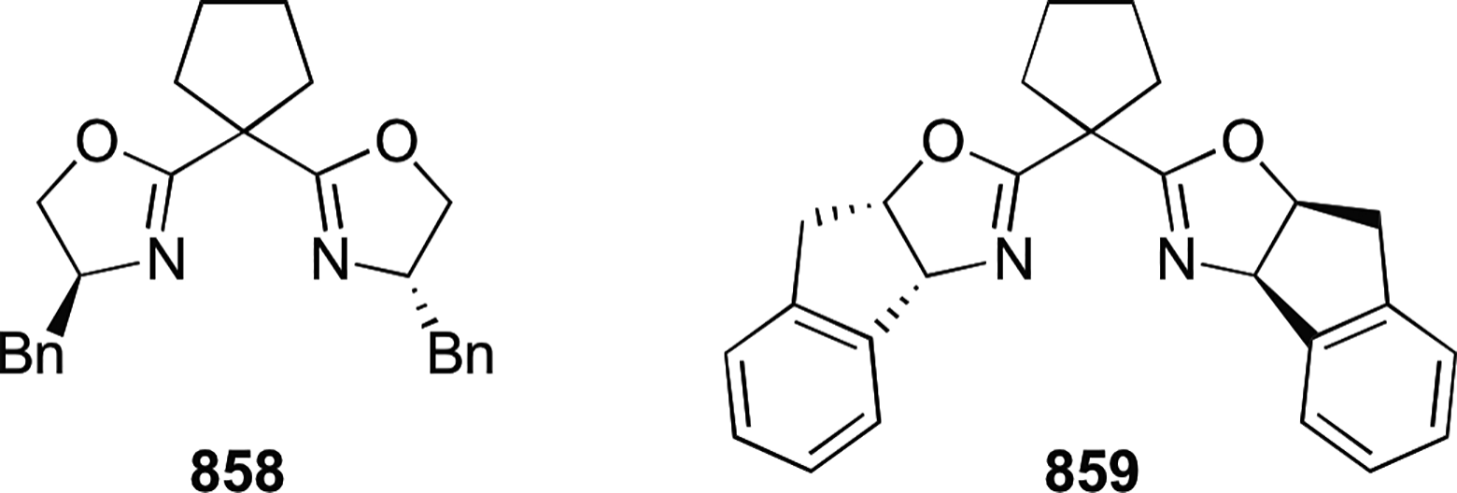
BOX ligands with cyclopentyl backbone modification.

**Scheme 282:**
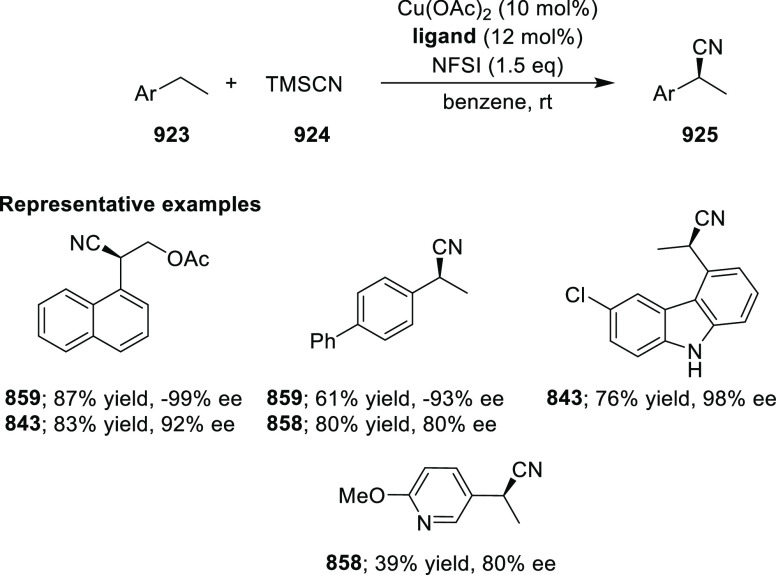
Cu-Catalyzed Asymmetric Synthesis of Benzylic Nitriles

Liu developed an asymmetric Cu-catalyzed cyanation by C–H activation of benzylic C–H bonds for the synthesis of benzylic nitriles **925** (Scheme 282). This reaction was catalyzed by a Cu(OAc)_2_·BOX complex using NFSI (*N*-fluorobenzenesulfonylimide) as the oxidant. Enantioselectivities up to 99% *ee* were obtained but each substrate had to screened for the optimal ligand, with BOX ligand **843** and the modified BOX ligands **858** and **859** found to be the ligands of choice (Figure 32). Alkyl-naphthylenes, alkyl arenes and alkyl heteroarenes all performed well under the optimized conditions with the lowest enantioselectivities obtained being 80% *ee*.^331^

**Scheme 283:**
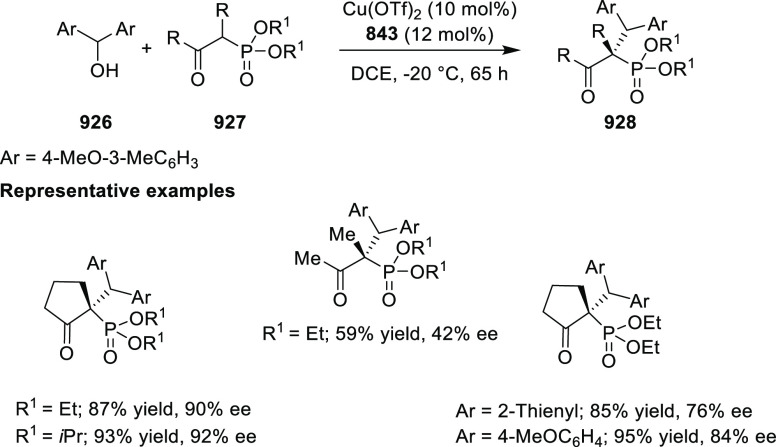
Cu-Catalyzed Asymmetric Alkylation of β-Keto Phosphonates

Nishibayashi developed the Cu-catalyzed enantioselective alkylation of β-keto phosphonates **927** using diaryl methanols **926** as the electrophiles (Scheme 283). Cu(OTf)_2_ and ligand **843** was shown to be the optimal catalyst giving the alkylated product **928** in up to 92% *ee*. Cyclic phosphonates were shown to be compatible with this catalysis affording the product in up to 92% *ee*, while acyclic phosphonates furnished the product in as low as 42% *ee*. Regarding the diarylmethanol the electronic nature of the aryl groups had little effect on the enantioselectivity (84–90% *ee*), heteroaryl substrates however produced the largest drop in enantioselectivity to 76% *ee*.^332^

**Scheme 284:**
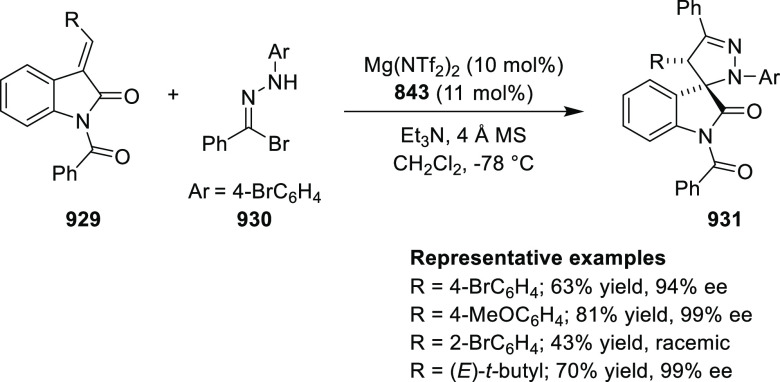
Mg-Catalyzed Asymmetric Cycloaddition of Nitrile Imines with Methyleneindolines

Stanley reported the Mg-catalyzed asymmetric cycloaddition of nitrile imines **930** with methyleneindolinones **929** to produce enantioenriched spirocyclic products **931** in up to 99% *ee* employing ligand **843** (Scheme 284). A range of methyleneindolinones were screened in the reaction with electron withdrawing and donating substituents giving rise to the product in up to 99% *ee*. Interestingly, an *ortho*-bromo substituent on the methyleneindolinone substrate led to racemic product.^333^

**Scheme 285:**
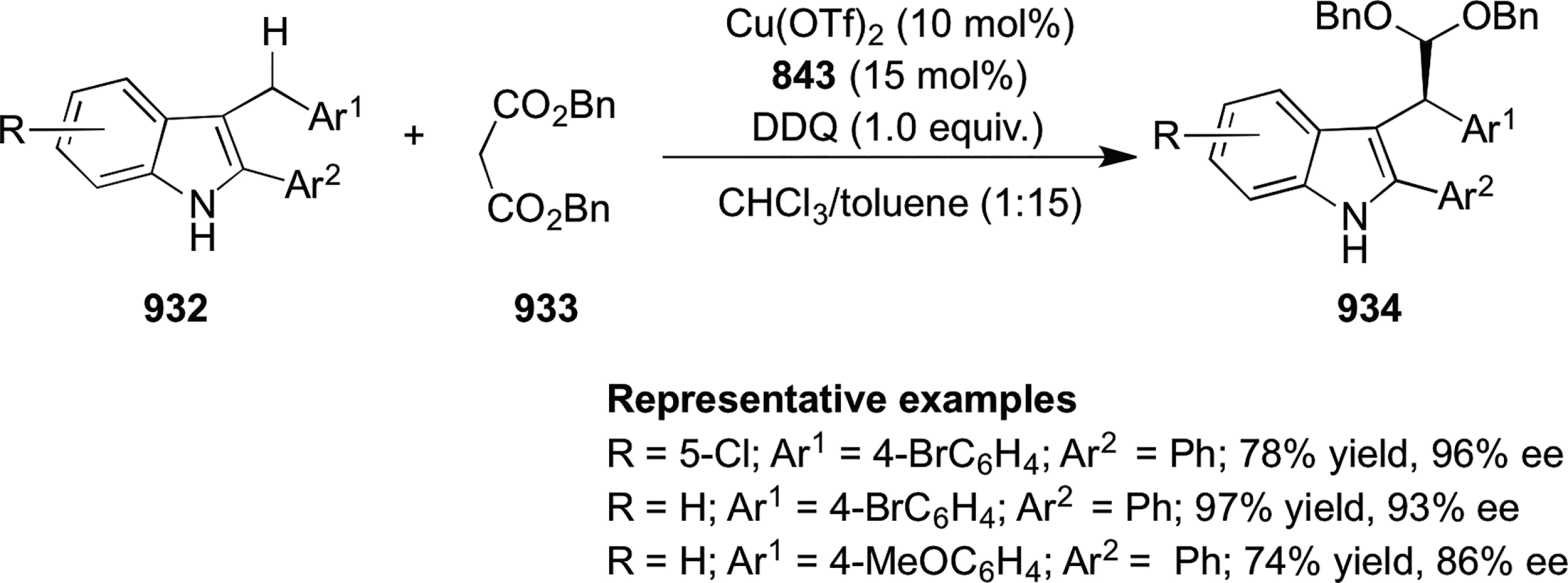
Cu-Catalyzed Asymmetric C–H Activation of Indole Derivatives

Gong developed an asymmetric Cu-catalyzed cross-coupling of 3-indoylmethyl C–H bonds **932** with diaryl malonates **933** (Scheme 285). With Cu(OTf)_2_ as the Lewis acid, **843** as the chiral ligand and DDQ (2,3-dichloro-5,6-dicyano-1,4-benzoquinone) as the oxidant, the product was formed in up to 99% yield and 96% *ee*. The substrate scope included a range of 3-arylmethylindole derivatives **932** which proved successful for both electronically rich or poor 3-arylmethyl substituted substrates (up to 99% yield and 94% *ee*). The electronic properties of the aryl substituent had little effect on the enantioselectivity (86 to 94% *ee*) The introduction of substituents onto the indole moiety afforded the product in high enantioselectivity (78% yield and 96% *ee*).^334^

**Scheme 286:**
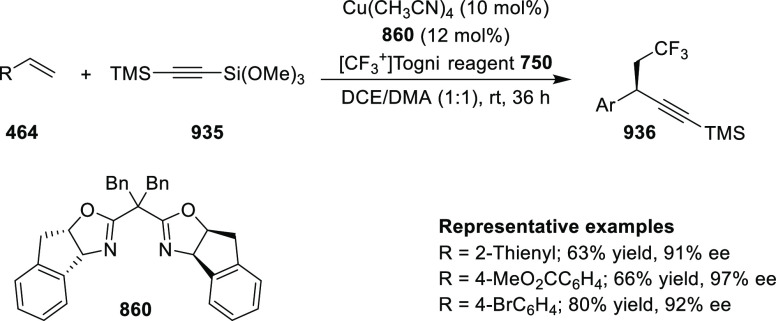
Cu-Catalyzed Asymmetric Trifluoromethylalkynylation of Alkenes

Liu reported the trifluoromethylalkynylation of alkenes **464** via Cu-Catalyzed radical relay producing chiral CF_3_-containing propargylic compounds **936** in up to 97% *ee* (Scheme 286). Using a complex formed from Cu(CH_3_CN)_4_PF_6_ and ligand **860** as the catalyst complex and Togni’s reagent **750** as the CF_3_ source, a wide range of products were accessed in good yields (up to 88% yield). The substrate scope for the alkene **464** coupling partner included a series of structurally diverse alkenes with electron-rich and poor styrenes affording the product in enantioselectivities up to 97% *ee*. Hetereoaryl alkenes also yielded the product in high enantioselectivities (87 to 95% *ee*).^335^

**Scheme 287:**
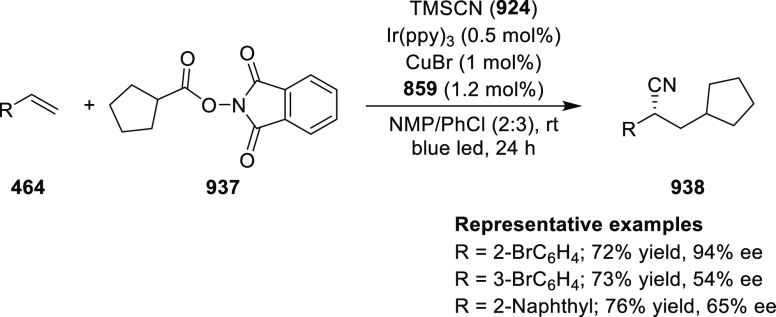
Photoredox Cyanoalkylation of Akenes

Pan reported the photoredox cyanoalkylation of akenes **464** using redox active *N*-hydroxy-phthalimide esters **937**. By merging Ir-catalyzed photocatalysis and Cu-catalysis the products were formed in up to 94% *ee* when using CuBr and ligand **859** (Scheme 287). A range of styrenes **464** were tested using the optimized reaction conditions, most of which gave the product in high enantioselectivities (84 to 94% *ee*). Large *p*-*t*-butylstyrene or 2-naphthylstyrene led to a drop in enantioselectivity (76% *ee* and 65% *ee*, respectively). *m*-Bromostyrene as substrate led to the most substantial drop in enantioselectivity with the product being obtained in 54% *ee*.^336^

**Scheme 288:**
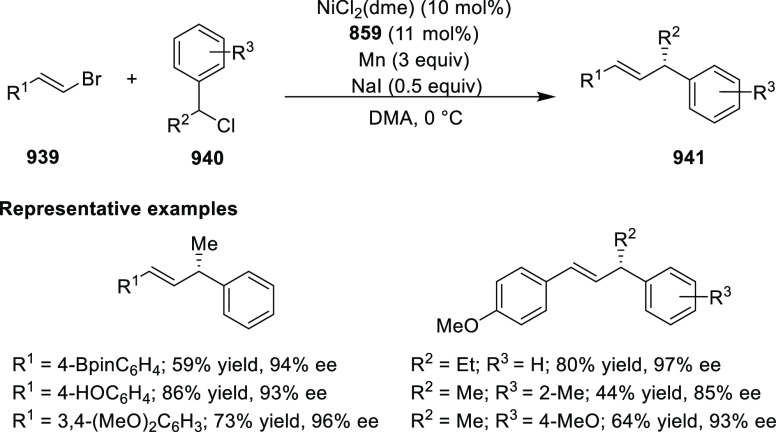
Ni-Catalyzed Asymmetric Cross-Electrophile Coupling of Vinyl and Benzyl Electrophiles

Reisman reported the Ni-catalyzed cross-electrophile reductive coupling between vinyl and benzyl electrophiles (Scheme 288). Using NiCl_2_(dme) as the catalyst and the chiral ligand **859**, enantioselectivities up to 97% *ee* were observed. Both the bromostyrene **939** and benzyl chloride **940** substrate scope had little effect on the enantioselectivity (as low as 85% *ee*) with pinacol boronate and free phenol functional groups being compatible with the reaction.^337^

##### 3.1.3.4 Bis(oxazoline) Ligands with Unsaturated Backbone Modifications

**Figure 33:**
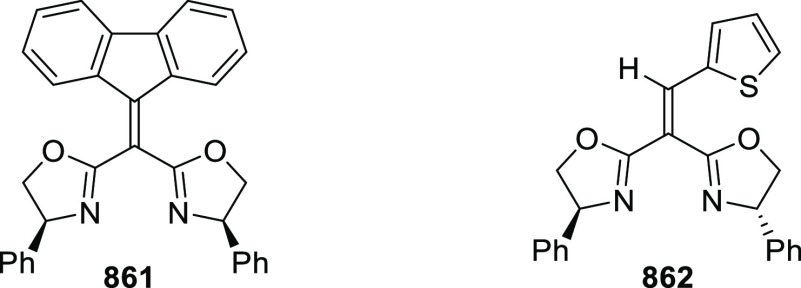
BOX ligands with unsaturated backbone modifications.

**Scheme 289:**
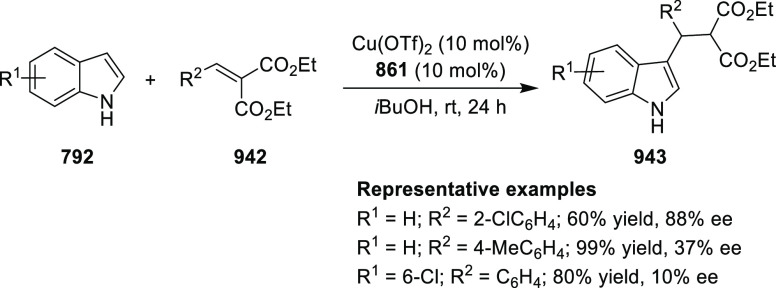
Cu-Catalyzed Friedel–Crafts Alkylation of Indoles and Alkylidene Malonates

Fu detailed the synthesis of the novel ligand **861** (Figure 33) and its application in the Cu-catalyzed Friedel–Crafts alkylation of indoles **792** by alkylidene malonates **942** (Scheme 289). The optimal reaction conditions utilized a combination of Cu(OTf)_2_ and ligand **861**, which yielded the product in up to 88% *ee*. The substrate scope probed electronic properties of the alkylidene malonates with *p*-substituted substrates leading to a significant drop in enantioselectivities (37% *ee*). Substitution on the indole ring also played a large effect with 6-chloroindole forming the product in just 10% *ee*.^338^

**Scheme 290:**
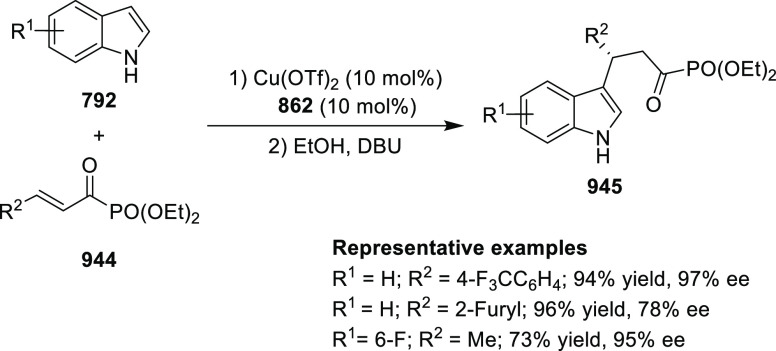
Cu-Catalyzed Conjugate Addition of Indoles to β-Substituted Unsaturated Acyl Phosphonates

Fu reported the Cu-catalyzed conjugate addition of indoles **792** to β-substituted unsaturated acyl phosphonates **744** to produce 3-indolyl adducts in good yields (Scheme 290). Heteroarylidene-tethered bis(oxazoline) ligand **862** and Cu(OTf)_2_ were used as the optimized catalytic conditions forming products in modest to very high yields (73–96%) and achieving very high levels of enantioselectivities of up to 97% *ee*. Regarding the substrate scope it appears that esters, nitrile, halides and ethers are all well tolerated on the indole ring with enantioselectivities remaining high (90–96% *ee*). Regarding the scope of the acyl phosphonates **744** a range of aryl and heteroaryl substrates were tested with 2-furyl providing the product in the lowest enantioselectivity (78% *ee*).^339^

**Figure 34:**
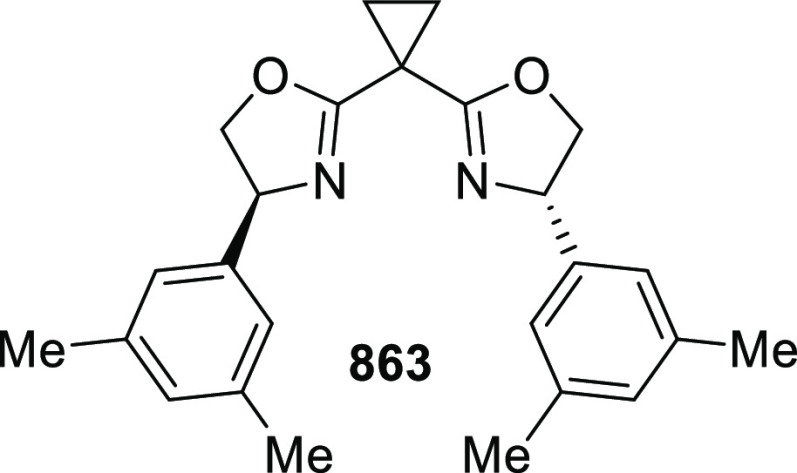
BOX ligand with xylene-derived substituents.

**Scheme 291:**
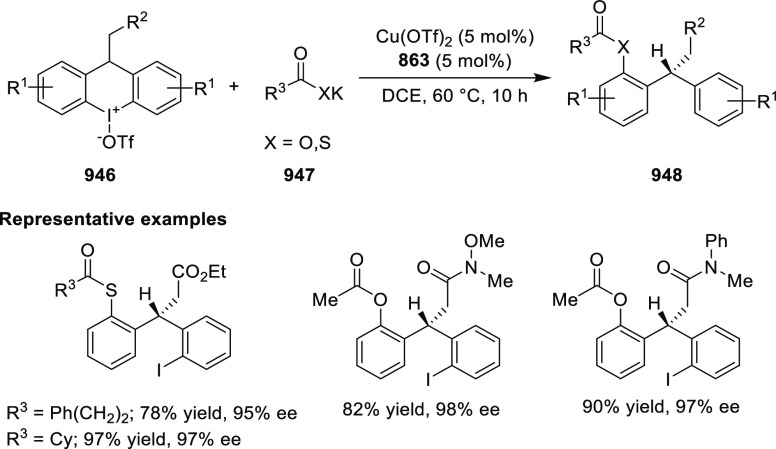
Cu-Catalzyed Asymmetric Synthesis of Diarylmethanes

Gu applied ligand **863** (Figure 34) in the Cu-catalyzed ring opening of diaryliodonium salts **946** in the synthesis of chiral diarymethanes **948** (Scheme 291). Using thiolates and carboxylates **947** as nucleophiles the asymmetric Cu(OTf)_2_-catalyzed ring opening gave the products **948** in good yields (up to 99% yield) and enantioselectivities up to 98% *ee*. Substituted thioacetates appeared to have a small effect on the enantioselectivity with most substrates ranging between 91 to 97% *ee*. The substrate scope for the carboxylates gave high enantioselectivity once again (93 to 98% *ee*) but the reaction time was extended from 10 to 48 h.^340^

##### 3.1.3.5 Application of Semicorrin Derived Ligands

**Figure 35:**
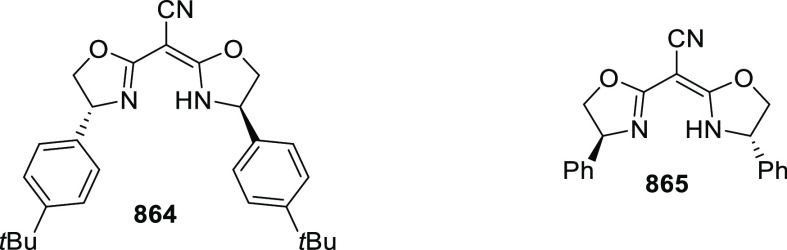
Semicorrin derived BOX ligands **864**–**865**.

**Scheme 292:**
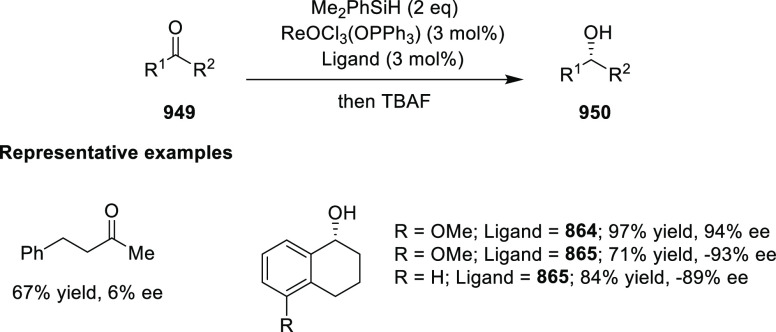
Re-Catalyzed Asymmetric Reduction of Ketones

**Scheme 293:**
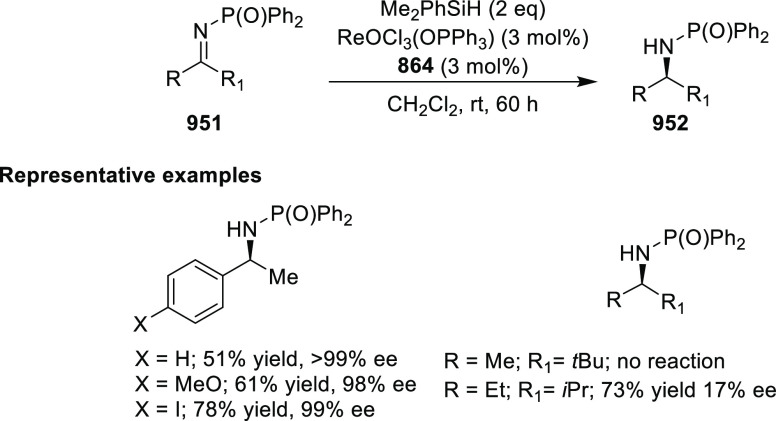
Re-Catalyzed Asymmetric Reduction of α-Aryl Phosphinyl Imines

Toste developed the Re-catalyzed asymmetric reduction of ketones **949** and imines **951** using Semicorrin ligands **864** and **865** developed by Pfaltz.^341a,341c^ When carrying out the asymmetric hydrosilylation of ketones **949** both ligands **864** and **865** (Figure 35) were screened reaching up to 94% *ee* and 93% *ee*, respectively, for substituted tetralones (Scheme 292). This methodology appeared to work only with aryl ketone substrates with 4-phenyl-2-butanone furnishing the product in 67% yield and 6% *ee*. This methodology was then expanded to the reduction of aryl phosphinyl imines **951** which furnished the product in up to >99% *ee* for some ary and heteroaryl imines (Scheme 293). Similarly, this reaction was limited as alkyl imines furnished the products **952** in lower enantioselectivities (0–32% *ee*).^341d^

##### 3.1.3.6 Application of Bi-Functional BOX Ligand with Photosensitizer Backbone Modifications

**Figure 36:**
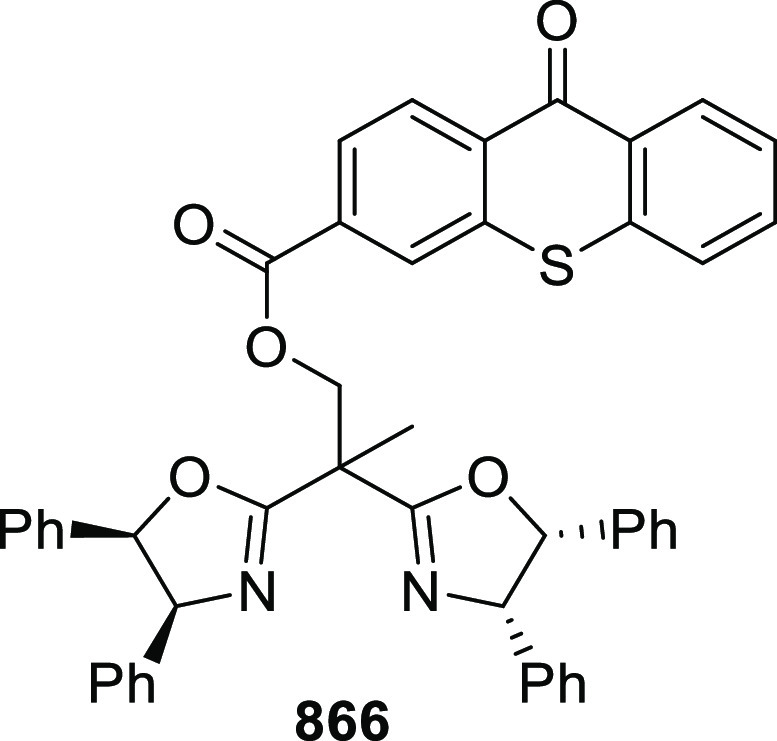
Bifunctional BOX ligand **963** with photosensitizer.

**Scheme 294:**
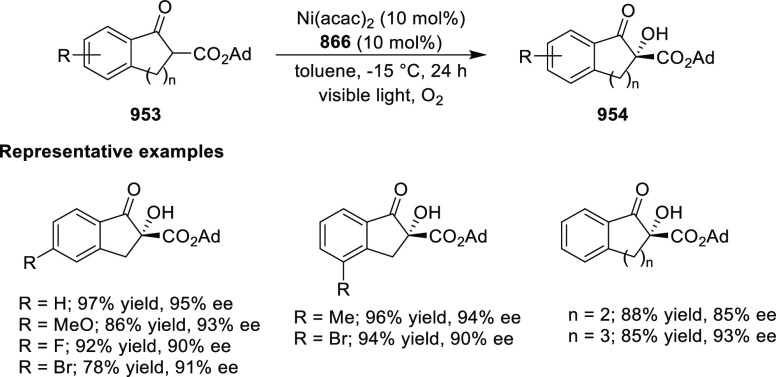
Ni-Catalyzed Asymmetric Oxidation of β-Keto Esters

Xiao developed a bifunctional box derived ligand **866** (Figure 36), which has a built-in diarylketone photosensitizer which allows the ligand to function as a chiral catalyst and a photocatalyst. Using ligand **866** and Ni(acac)_2_ the oxidation of β-keto esters **953** afforded the desired products **954** in up to 95% *ee* (Scheme 294). Regarding the substrate scope a range of cyclohexanone-derived β-keto esters **953** formed the products in high enantioselectivity (88–95% *ee*). Cycloheptanone-derived β-keto esters also formed the products in up to 93% *ee*.^342^

##### 3.1.3.7 Bis(oxazoline) Ligands with Miscellaneous Backbone Modifications

**Figure 37:**
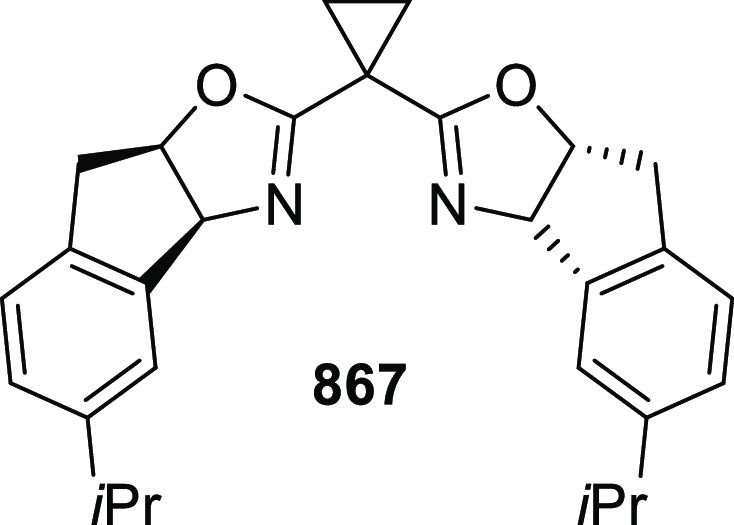
Functionalized indene-derived ligand **867**.

**Scheme 295:**
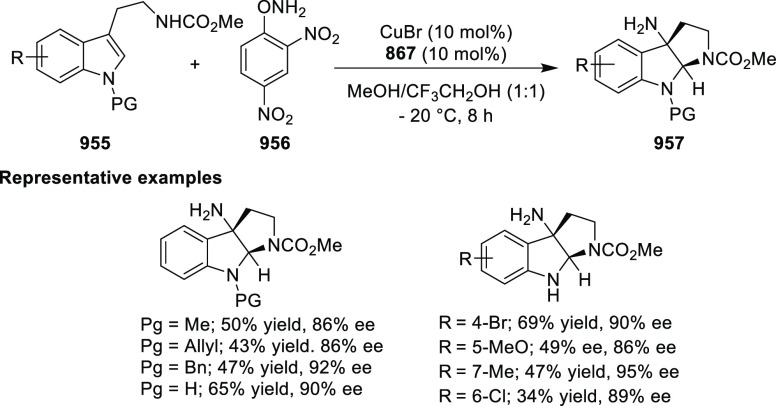
Cu-Catalyzed Enantioselective Synthesis of 3a-Amino-pyrroloinolines

**Figure 38:**
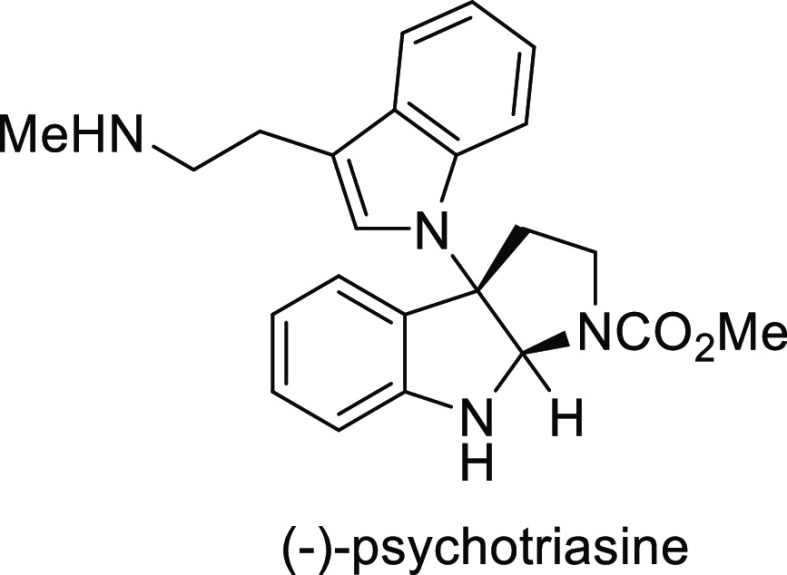
(−)-Psychotriasine.

You applied ligand **867** in the Cu-catalyzed dearomative amination of tryptamines **955**. Using ligand **867** (Figure 37), CuBr and *O*-(2,4-dinitrophenyl)hydroxylamine **956**, an efficient electrophilic aminating reagent a range of 3-amino-pyrroloindolines **957** were synthesized in up to 95% *ee*. A range of indole protecting groups were attempted with allyl, methyl and benzyl all affording the product in high enantioselectivities (86–92% *ee*) (Scheme 295). An indole substrate scope was carried out with a variety of electron withdrawing and donating groups in the 4–7 positions providing the products in high enantioselectivities (82–95% *ee*). This methodology was then applied to the formal synthesis of tryptophan-derived alkaloids (−)-psychotriasine (Figure 38).^343^

**Scheme 296:**
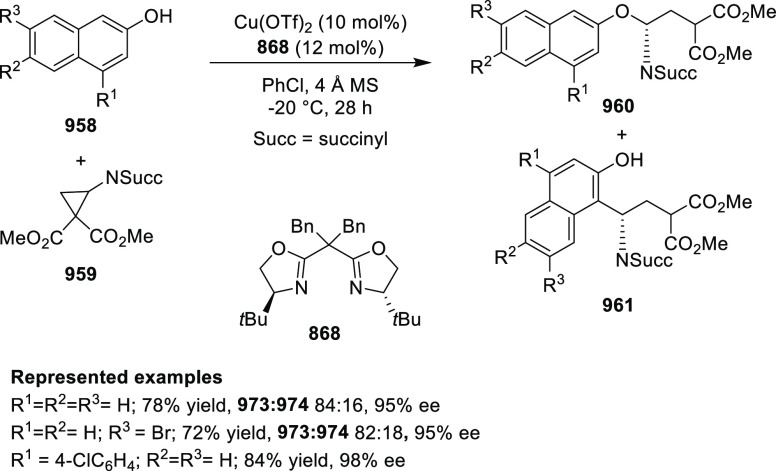
Cu-Catalyzed Asymmetric Friedel–Crafts Alkylation of β-Naphthols and Aminocyclopropanes

Guo reported the Cu-catalyzed Friedel–Crafts alkylation of β-naphthols **958** and aminocyclopropanes **959** to make γ-aminobutyric acid-like structures **961**. Using the chiral ligand **868** and Cu(OTf)_2_ the desired Friedel–Crafts products were isolated in up to 98% *ee* (Scheme 296). The undesired *O*-alkylation product **960** was seen when less sterically demanding β-naphthols were used. Besides the chemoselectivity issue presented by the *O*-alkylation product a large number or electronically diverse β-naphthols were applied to this catalysis forming the products in high levels of enantioselectivity (90–98% *ee*).^344^

**Figure 39:**
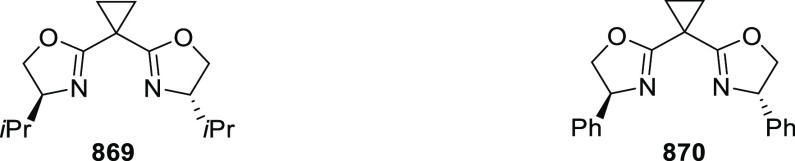
BOX ligand with cyclopropyl-modified backbone.

**Scheme 297:**
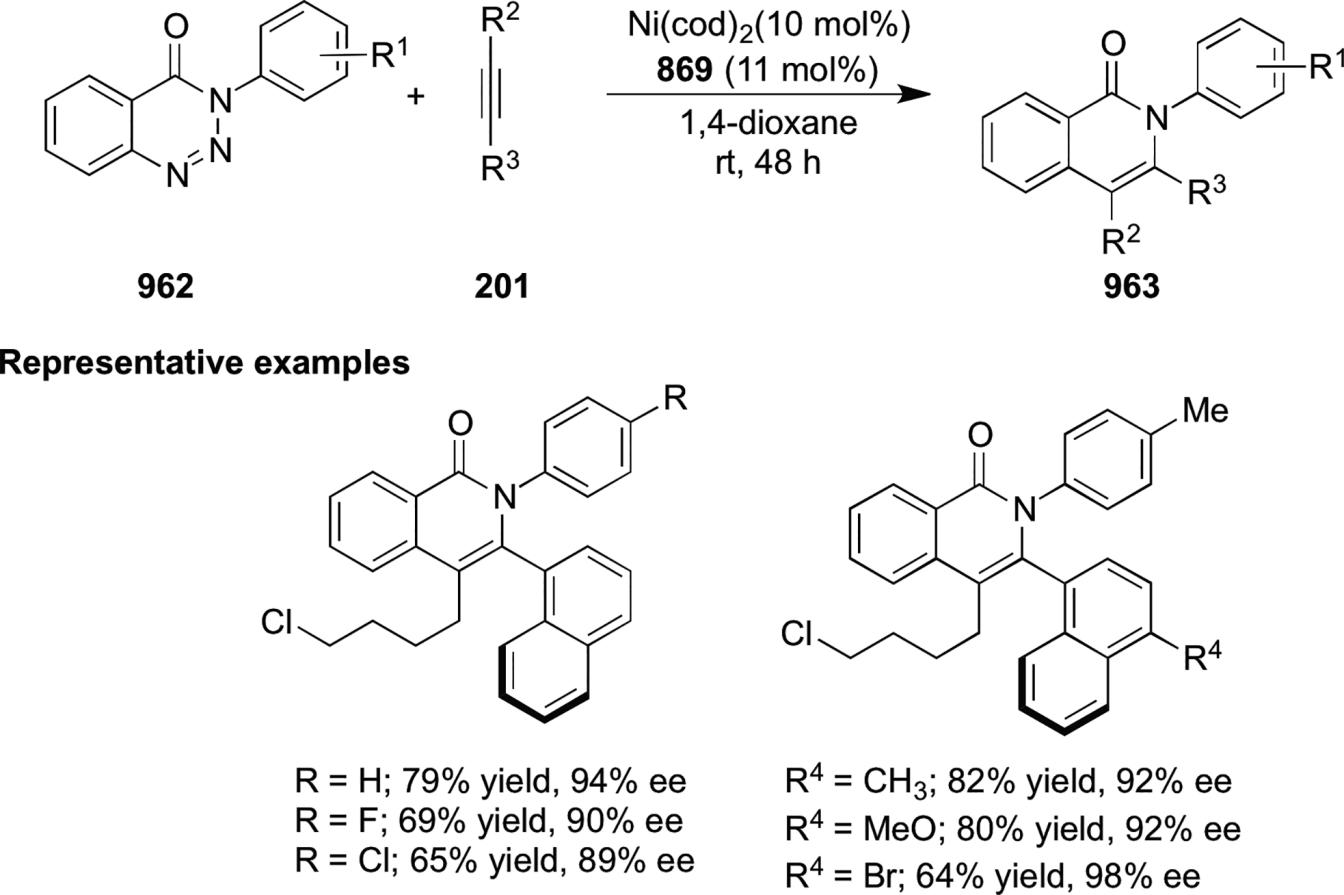
Ni-Catalyzed Enantioselective Denitrogenative Transannulation

Liu reported the synthesis of axially chiral isoquinolones **963** via a Ni-catalyzed denitrogenative transannulation (Scheme 297). Ni(cod)_2_ and ligand **869** (Figure 39) proved to be the optimal conditions for the denitrogenative transannualtion of 1,2,3-benzotriazin-4(3H)-ones **962** and bulky internal alkynes **201**, to give the product **963** in good yields and enantioselectivities of up to 98% *ee*. This reaction is amenable to both electron-withdrawing and electron donating groups at the 4-position of the naphthalene ring with 4-bromonaphthalene giving the highest *ee* of 88%. The stability of these axially chiral products **963** was tested by refluxing them in toluene for 1 day. No erosion of enantioselectivity was observed demonstrating the high barrier to rotation about the naphthyl–isoquinolone bond.^345^

**Scheme 298:**
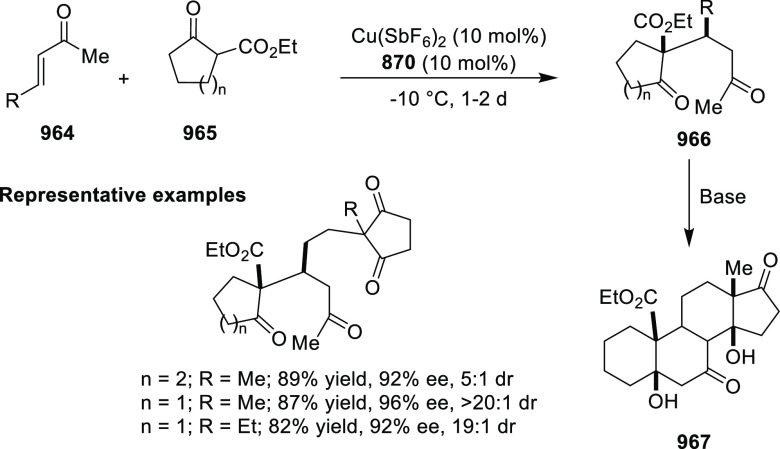
Cu-Catalyzed Asymmetric Synthesis of Oxygenated Steroid Derivatives

Nagorny developed a concise Cu-catalyzed synthesis of oxygenated steroids *via* a sequential Michael-addition/intramolecular aldol cyclization protocol (Scheme 298). Using ligand **870** (Figure 39) and Cu(SbF_6_)_2_ to catalyze the Michael addition of β,β′-enones **964** and substituted β,β′-keto esters **965** resulted in Michael adducts which can undergo base-promoted aldol cascade reactions resulting in steroid skeletons **967**. Regarding the substrate scope of the Michael addition, five-membered β-keto esters **965** proceeded with high levels of diastereocontrol (up to >20:1 dr and up to 96% *ee*). Six-membered β-keto esters **965** proceeded in high enantioselectivities (up to 94% *ee*) but the diastereocontrol dropped to as low as 4:1 dr. Once treated with base the double aldol cyclization occurred to form the steroidal backbone.^346^

**Figure 40:**
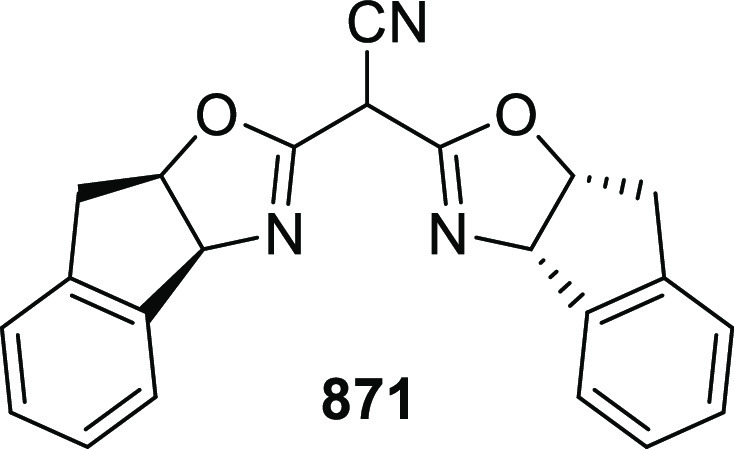
Indene BOX ligand **871** with nitrile backbone modification.

**Scheme 299:**
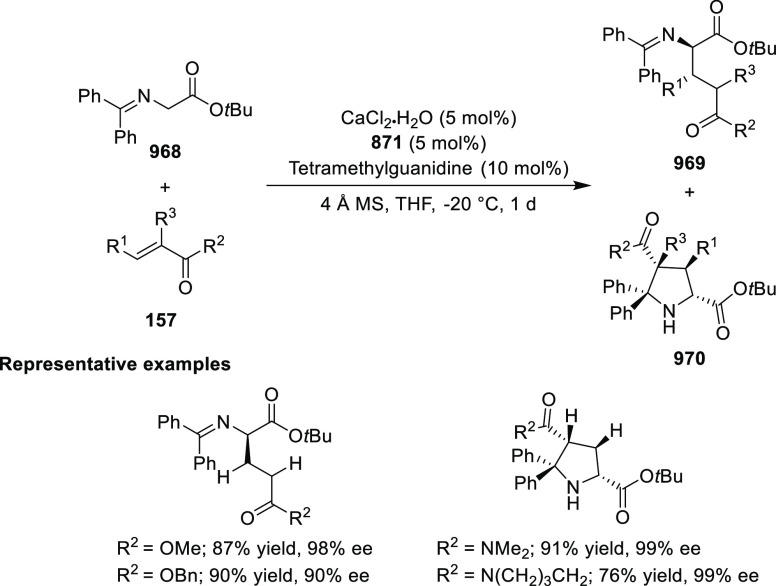
Calcium-Catalyzed Asymmetric Synthesis of Pyrrolidine Derivatives

Kobayashi applied ligand **871** (Figure 40) in the Ca-catalyzed 1,4-addition to form product **969** and a [3 + 2] cycloaddition toward the synthesis of pyrrolidine derivatives **970** (Scheme 299). Using CaCl_2_ and ligand **871** as the optimized catalyst the tandem reaction sequence between imines **968** and enones **157** proceeded in up to 99% *ee* and in good yields (up to 98% yield). Imines **968** containing amides appeared to give complete conversion to the pyrrolidine products **970** while ester-containing imines underwent the 1,4-addition with complete selectivity to form **969**.^347^

**Figure 41:**
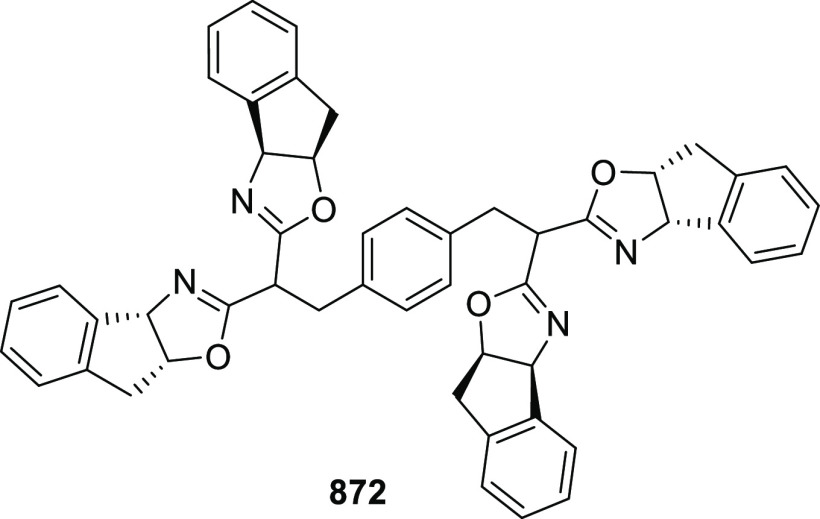
Recyclable BOX ligand **872**.

**Scheme 300:**
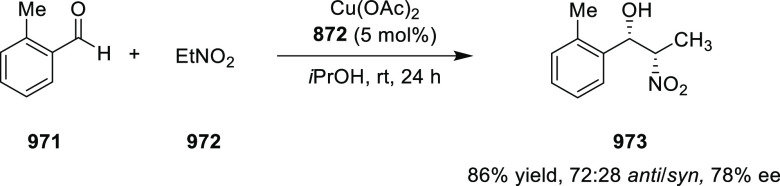
Cu-Catalyzed Enantioselective Henry Reaction with a Non-supported Recyclable Ligand

Miñana reported the application of the recyclable ligand **872** (Figure 41) in the Cu-catalyzed Henry reaction of aldehydes **971**. Using Cu(OAc)_2_ and ligand **872** enantioselectivities up to 78% *ee* were observed with *anti*/*syn* ratios of up to 72:28 (Scheme 300). This ligand avails of a release-capture technique which can be recycled after a run of catalysis without the need for a heterogeneous support. After the reaction solvent is removed the ligand is precipitated by washing with hexane/ether removing it from the product solution. The precipitated ligand is dried and reused in another reaction. The viability of **872** as a recyclable ligand was tested by placing it through 14 subsequent runs of catalysis after recovery. This showed a slight decrease in enantioselectivity to 72% *ee* after 14 cycles in catalysis.^348^

**Figure 42:**
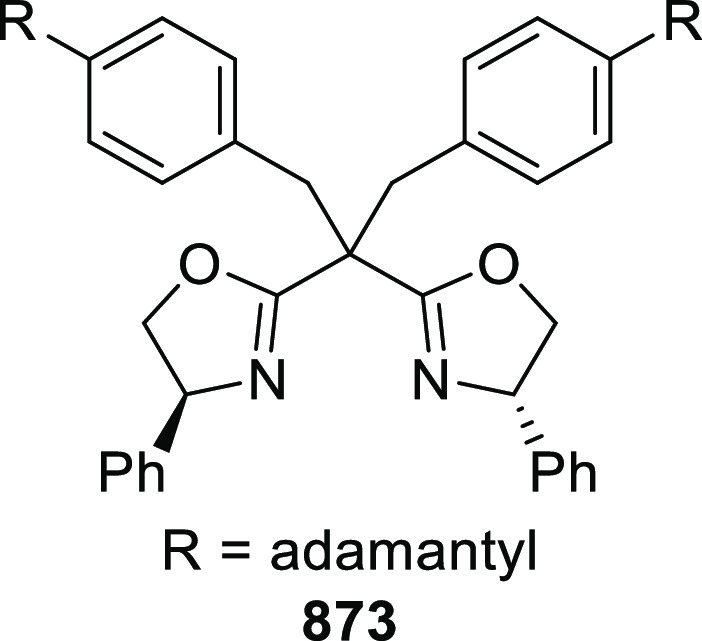
BOX ligand **873** with adamantyl backbone modification.

**Scheme 301:**
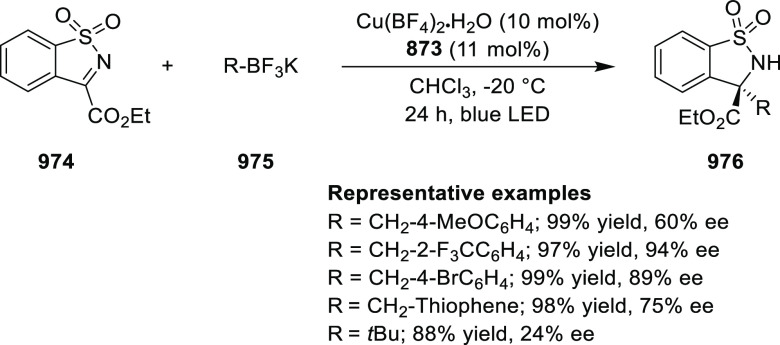
Cu-Catalyzed Asymmetric Alkylation of Imines

**Scheme 302:**
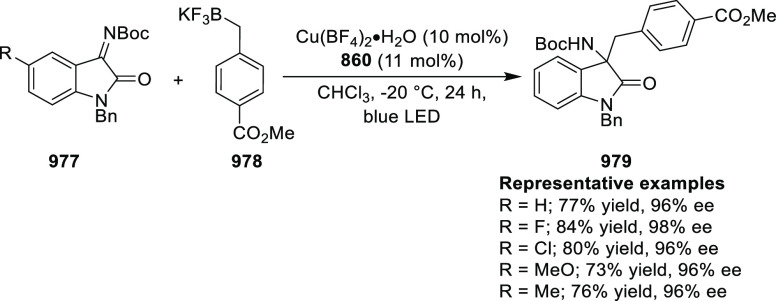
Cu-Catalyzed Enantioselective Alkylation of Isatin-Derived Ketimines

Gong reported the Cu-catalyzed asymmetric alkylation of imines **974** driven by light. The light promotes radical formation of the trifluoroborates and acts as a reducing agent for the copper catalyst. Using ligand **873** (Figure 42), a range of *N*-sulfonylimines **974** were alkylated using alkyl trifluoroborates **975** in high yields and enantioselectivities of up to 94% *ee* (Scheme 301). The scope of this reaction investigated various electron-withdrawing and donating alkyl trifluoroborates and all were found to maintain high enantioselectivities (81–94% *ee*). Lower enantioselectivities were observed when using *p*-methoxyphenyl, heteroaryl and bulky *t*-butyl trifluoroborates (as low as 24% *ee*). This methodology was then applied to the benzylation of isatin-derived ketimines with **860** as the ligand. A variety of isatin-derived ketimines were attempted furnishing the product in up to 98% *ee* (Scheme 302).^349^

**Scheme 303:**
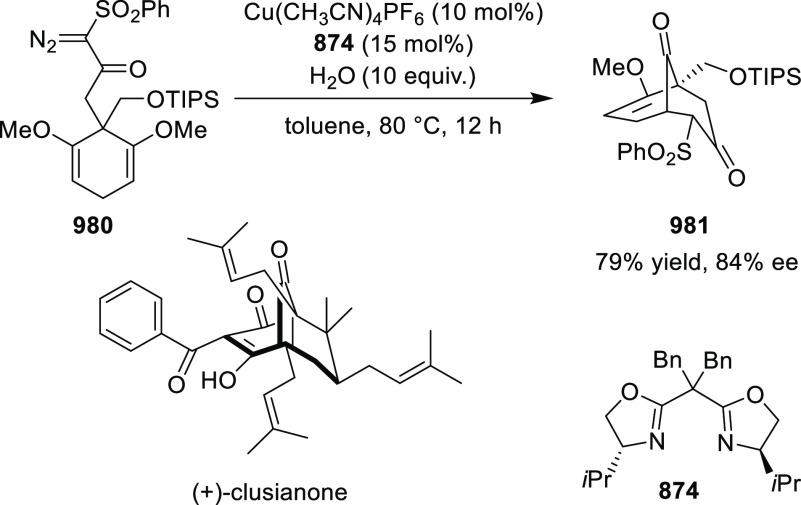
Cu-Catalyzed Asymmetric Intramolecular Cyclopropanation of α-Diazo Ketones

Nakada reported the Cu-catalyzed catalytic asymmetric intramolecular cyclopropanation of α-diazo ketones **980** in the synthesis of polycyclic polyprenylated acylphloroglucinols (Scheme 303). Using ligand **874** with Cu(CH_3_CN)_4_ the cyclopropylated product is formed and undergoes a subsequent rearrangement with water present to furnish the diketone **981** (79% yield, 84% *ee*), a valuable intermediate in the formal synthesis of (+)-clusianone.^350a^

**Scheme 304:**
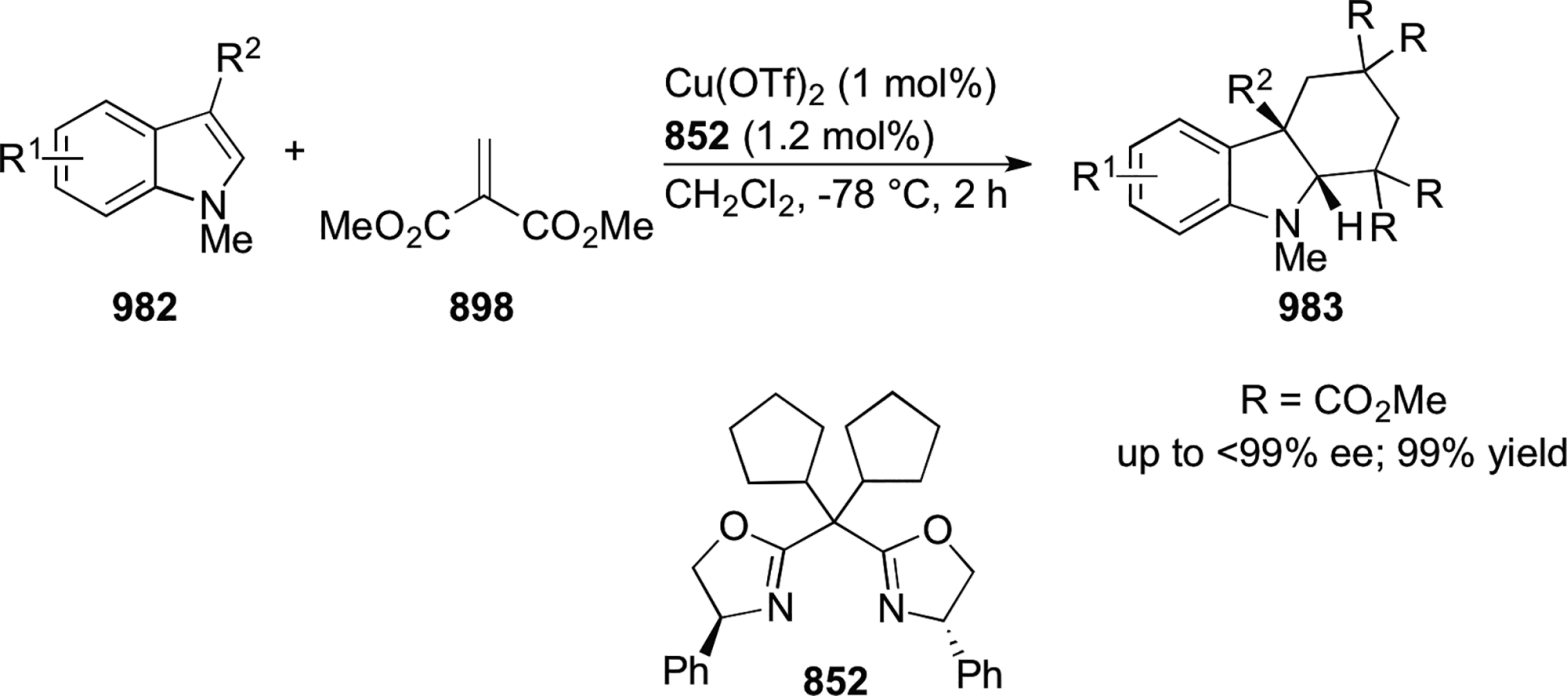
Tandem [2 + 2 + 2] Asymmetric Reaction of Indole with Methylenemalonate

In 2017, Tang reported the synthesis of Wing-BOX ligand **852** and it is application in the synthesis optically active hexahydrocarbazoles **983** in up to <99% *ee* and 99% yield (Scheme 304). Ligand **852** exploits the Thorpe–Ingold effect using the cyclopently groups on the backbone to enhance enantioselectivity (the parent bis(oxazoline) ligand **600c** furnished the product in 45% *ee* and 76% yield).^350b^

It has been shown that BOX ligands with backbone modification are used largely within Lewis acid catalysis and cross-coupling to great effect. On multiple occasions they are employed in a scenario where parent BOX ligands achieve moderate enantioselectivity and are used to fine-tune the bite angles, electronics and achieve excellent enantioselectivity. From this section of the review, it is plain to see the far-reaching applications of BOX ligands and modified BOX ligands in asymmetric catalysis. A large proportion of this catalysis entails Lewis acid activation and inherently inexpensive metals such as Zn and Cu. While traditional enantioselective cross-couplings with Pd catalysts are still popular the increased use of inexpensive metals such as Ni or Co has been noted. One key reason for the Nickel’s rise to prominence is its’ use in asymmetric cross-electrophile coulpling with simple coupling partners such as pseudohalides and halides. It is envisaged that the increased research activity in photochemistry and electrochemistry will further enhance and compliment the asymmetric methods developed and discussed above.

### 3.2 Bis(oxazoline) Ligands with Other Linkers

#### 3.2.1 Bis(oxazoline) Ligands Directly Connected at *C*_1_

**Figure 43:**
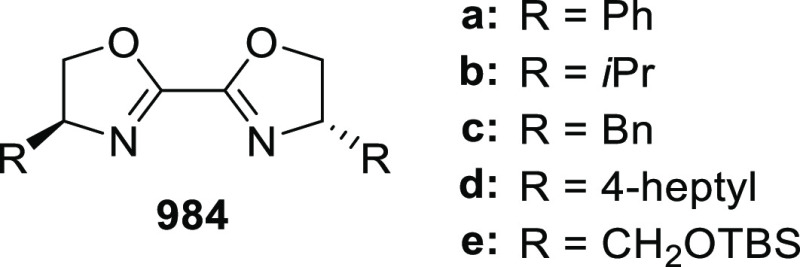
BiOX ligands.

Bioxazoline (BiOX) ligands **984a**–**e** are bis(oxazoline) ligands with the oxazoline moieties directly attached to each other (Figure 43). They have emerged as a useful class of chiral ligand, especially in asymmetric Ni-catalyzed cross-coupling reactions.

**Scheme 305:**
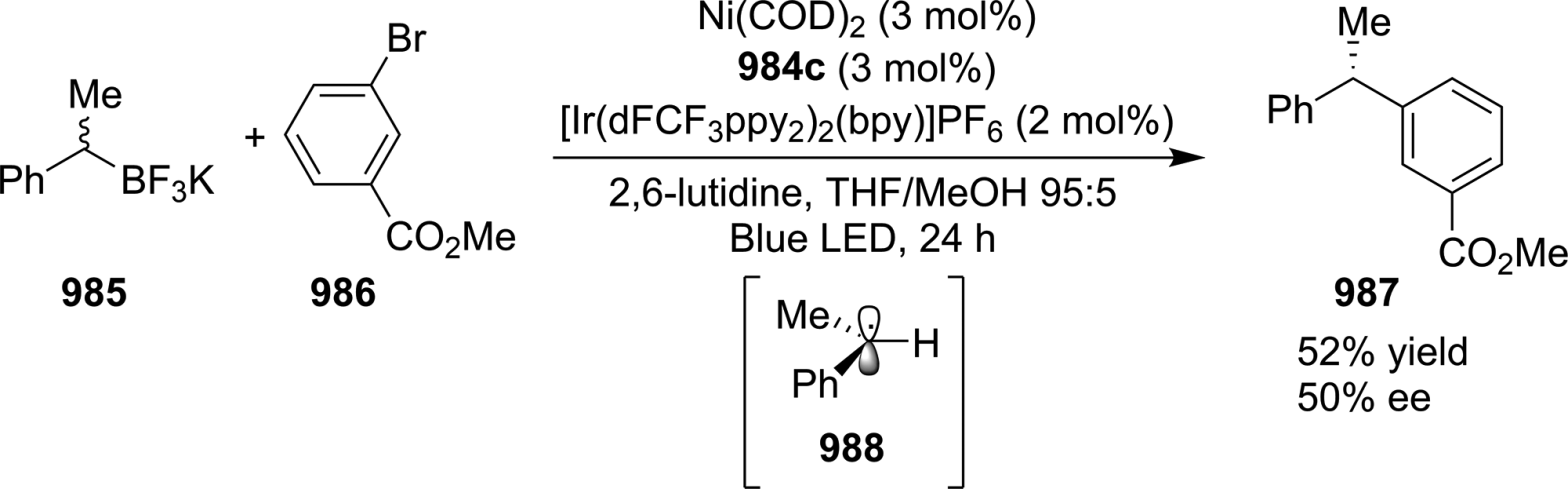
Dual Photoredox/Ni-Catalyzed Cross-Coupling Reaction of Benzyltrifluoroborates with Aryl Bromides

Molander reported a dual photoredox/Ni-catalyzed cross coupling reaction of benzyltrifluoroborates with aryl bromides. They showed that the application of BiOX ligand **984c** in the cross coupling of secondary benzyltrifluoroborate **985** with aryl bromide **986** led to the isolation of the enantioenriched product **987** in 52% yield and with 50% *ee* (Scheme 305). The stereoconvergent single-electron transmetalation event in this reaction (unlike traditional stereospecific transmetalation) allows for facial discrimination of the prochiral alkyl radical **988** by a chiral ligand.^351^

**Scheme 306:**
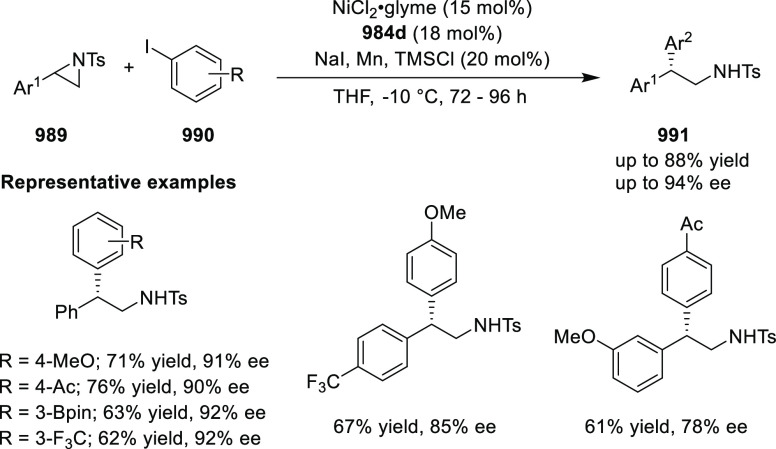
Enantioselective Ni-Catalyzed Reductive Cross-Coupling of Styrenyl Aziridines with Aryl Iodides

Doyle and Sigman have applied BiOX ligand **984d** in an enantioselective Ni-catalyzed reductive cross-coupling of styrenyl aziridines **989** with aryl iodides **990**. A range of racemic aryl aziridines **989** were subjected to the reaction with multiple aryl iodides **990** to give the enantioenriched amines **991** in good yields up to 88% and high enantioselectivities up to 94% *ee* (Scheme 306). Switching from the initial nonchiral ligand (bpp) for the racemic transformation led to a decrease in the yield of the cross-coupling reaction, however the addition of NaI and catalytic TMSCl was found to increase the yields.^352^

**Scheme 307:**
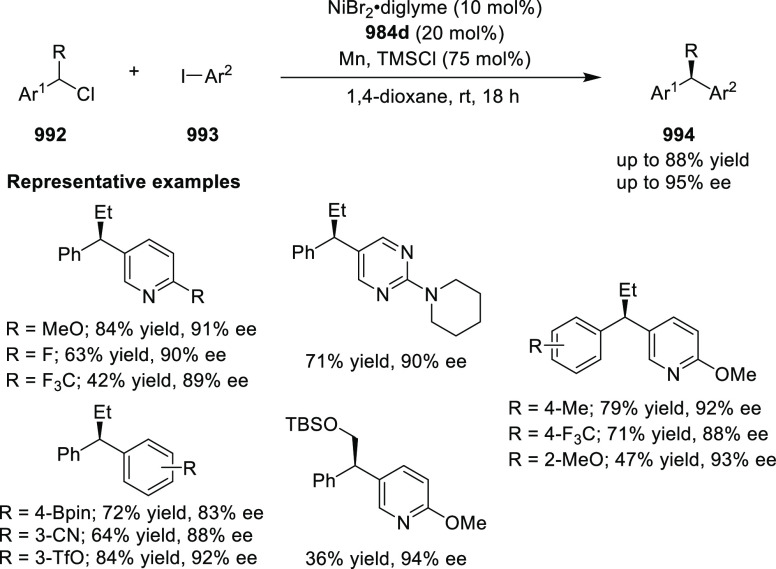
Enantioselective Ni-Catalyzed Reductive Cross-Coupling Reaction of Aryl Iodides with Secondary Benzyl Chlorides

Contemporaneously, Reisman reported an enantioselective Ni-catalyzed reductive cross-coupling reaction of aryl iodides **993** with secondary benzyl chlorides **992**, utilizing 4-heptyl BiOX ligand **984d**. A range of aryl and heteroaryl iodides **993** were well tolerated in the reaction, as were a range of secondary benzyl chlorides **992**, giving the diaryl alkanes **994** in moderate to good yields up to 88% and with high enantioselectivities up to 95% *ee* (Scheme 307). Mn^0^ was found to be an essential reductant and TMSCl an essential activator for the outcome of the reaction which, unlike the previous example, could be run at ambient temperature.^353^

**Scheme 308:**
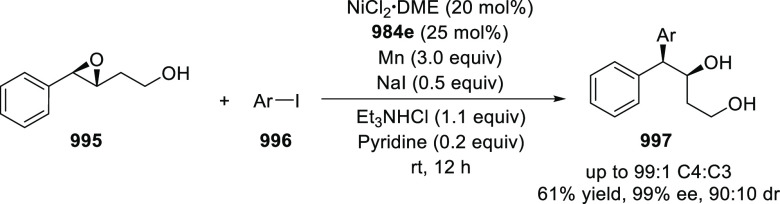
Regio- and Diastereoselective Ni-Catalyzed Reductive Cross-Coupling between Epoxide and Aryl Iodides

Yamamoto has reported a regio- and diastereoselective Ni-catalyzed reductive cross-coupling of enantioenriched-3,4-epoxyalcohols **995** with aryl iodides **996**, utilizing BiOX ligand **984e** (Scheme 308). This general protocol furnishes a new type of enantioenriched 4,4-diaryl alkane **997**, which also incorporates an additional 1,3-diol, in up to 61% yield and with up to 99% *ee*. The diol can be easily transformed to a variety of functional groups.^354^

**Scheme 309:**
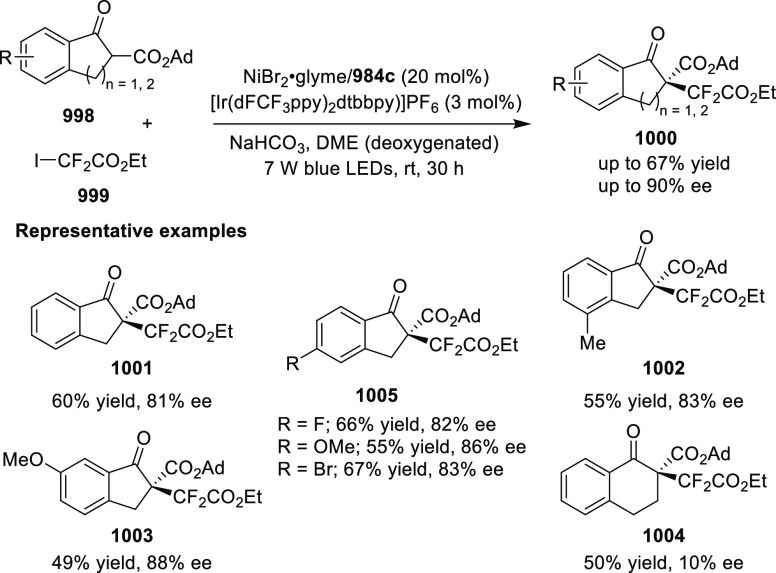
Enantioselective Difluoroalkylation of β-Keto Esters by Dual Photoredox/Ni-Catalysis

Lu and Xiao have described an enantioselective difluoroalkylation of β-keto esters by dual photoredox/Ni-catalysis. Employing Bn-BiOX ligand **984c**, a range of indanone- and tetralone-β-keto esters **998** were successfully difluoroalkylated with iodofluoroacetates **999** to give the corresponding indanones and one tetralone **1000** bearing α-quaternary stereocenters in moderate yields up to 67% and with high enantioselectivities up to 90% *ee* (Scheme 309). The reaction was found to work with bromofluoroacetates in a slightly lower yield and preliminary studies showed it could also be applied to perfluoroalkylation with iodofluoro compounds under altered reaction conditions.^355^

**Scheme 310:**
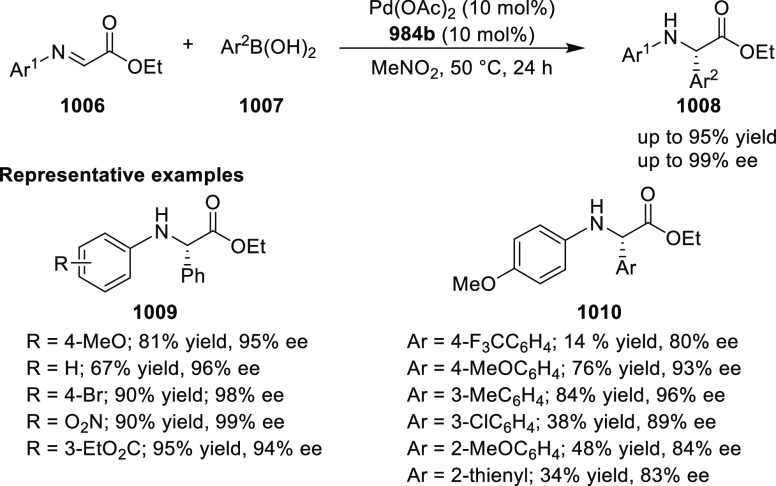
Asymmetric Pd-Catalyzed Arylation of α-Imino Esters with Aryl Boronic Acids

BiOX ligands of the type **984** have also found application in asymmetric Pd-catalyzed transformations. For example, Zeng has reported an asymmetric Pd-catalyzed arylation of α-imino esters **1006** with aryl boronic acids **1007**. A range of enantioenriched α-diaryl amino esters **1008** were accessed in low to high yields up to 95% and with excellent enantioselectivities up to 99% *ee*, utilizing *i*Pr-BiOX ligand **984b** as the chiral ligand (Scheme 310). Electron-withdrawing groups on the α-imino ester **1006** were well tolerated while electron-donating groups led to a decrease in reaction yield. Variation of the aryl boronic acid **1007** had a more dramatic effect on the outcome of the reaction, with electron-withdrawing and -donating groups decreasing the reaction yield.^356^ Yang later reported a related C–H oxidative cross-coupling, in which the same α-imino esters are formed by *in situ* oxidation with 2,2,6,6-tetramethylpiperidine-1-oxoammonium tetrafluoroborate, which enters the same catalytic cycle as in Zeng’s report.^357^

**Scheme 311:**
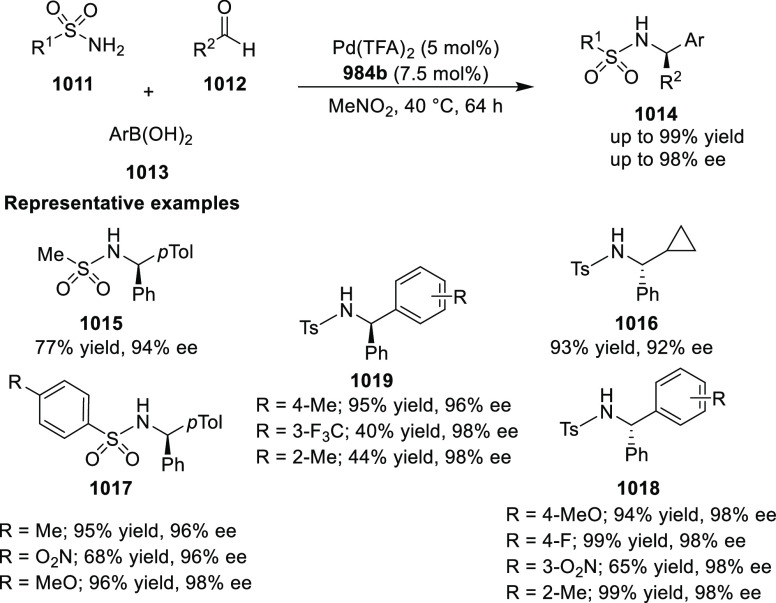
Enantioselective Pd-Catalyzed Three-Component Synthesis of α-Substituted Amines

Manolikakes reported a related enantioselective Pd-catalyzed three-component synthesis of α-substituted amines. An array of aryl or alkyl sulfonamides **1011** and aldehydes **1012** were reacted together with aryl boronic acid **1013** in the presence of Pd(TFA)_2_ and *i*Pr-BiOX ligand **984b** to give enantioenriched α-substituted amines **1014** in up to 99% yield and with excellent enantioselectivities up to 98% *ee* (almost all >95% *ee*) (Scheme 311). The reaction was found to be insensitive to air and moisture.^358^ Later, Manolikakes reported the same reaction with glyoxylic acid derivatives in place of regular aldehydes giving enantioenriched arylglycines with similar results.^359^

**Scheme 312:**
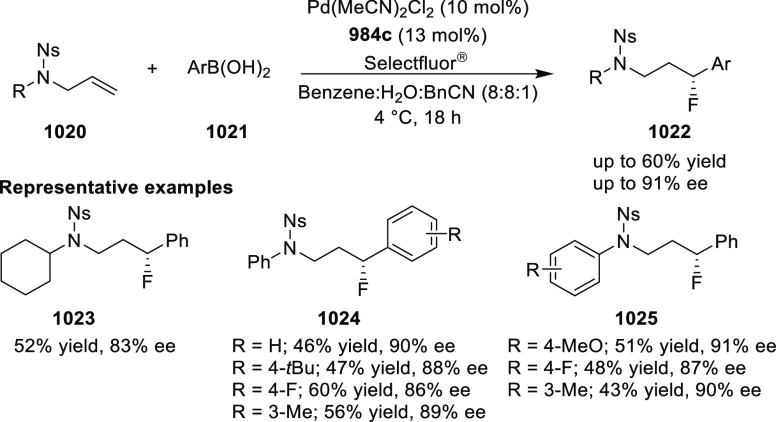
Enantioselective Pd-Catalyzed 1,1-Fluoroalkylation of Amino Alkenes with Aryl Boronic Acids and Selectfluor

Toste has reported an enantioselective Pd-catalyzed 1,1-fluoroalkylation of amino alkenes **1020** with aryl boronic acids **1021** and Selectfluor. Utilizing Bn-BiOX ligand **984c**, a range of fluorinated amines **1022** were synthesized in moderate yields up to 60% and with high enantioselectivities up to 91% *ee* (Scheme 312). It was found that the exclusion of water from the racemic reaction mixture led to no observable product formation, while the addition of acetonitrile dramatically increased the yield of the product. Interestingly, in the asymmetric reaction, the removal of the nitrile led to trace amounts of product formation, while the use of benzonitrile, in place of acetonitrile, led to the highest levels of enantioselectivity.^360^

**Scheme 313:**
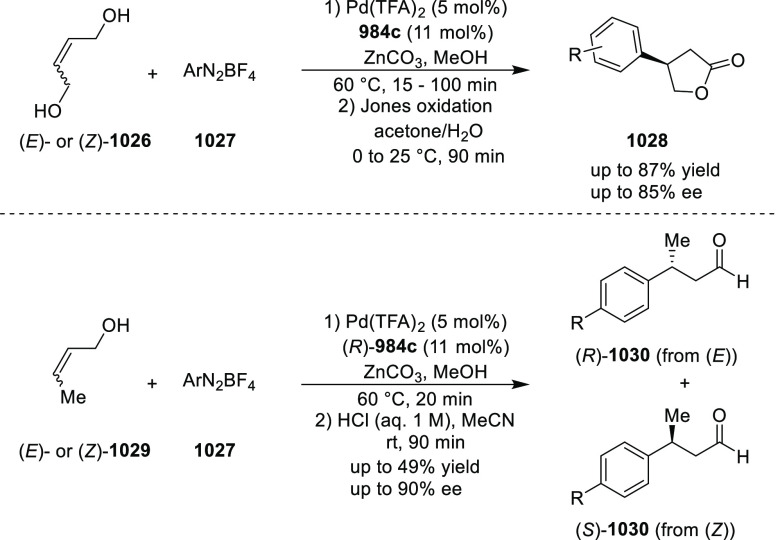
Enantioselective Heck–Matsuda Arylation of Allyl Alcohols with Aryl Diazonium Tetrafluoroborates

Correia has developed an enantioselective Heck–Matsuda arylation of (*Z*)-allyl alcohols with aryl diazonium tetrafluoroborates. When diols **1026** are reacted with diazonium tetrafluoroborates **1027** in the presence of Pd(TFA)_2_ and Bn-BiOX ligand **984c**, followed by a Jones oxidation, the corresponding enantioenriched α-aryl lactones **1028** were isolated in up to 87% yield and with up to 85% *ee* (Scheme 313). The Heck–Matsuda arylation gives the corresponding lactols, which are then converted to the lactones. The lactol intermediates can be further derivatized by other means to give a range of enantioenriched products. The reaction was found to be stereoconvergent and similar results were obtained when an (*E*)-allyl diol was used. A small range of allyl alcohols **1029** were subjected to the reaction to give dimethyl acetal intermediates which, following acid hydrolysis, gave the corresponding β-aryl aldehydes **1030** in up to 49% yield and with up to 90% *ee*. As expected, (*Z*)- and (*E*)-allyl alcohols gave opposite enantiomers of the product aldehydes **1030**.^361^

**Scheme 314:**
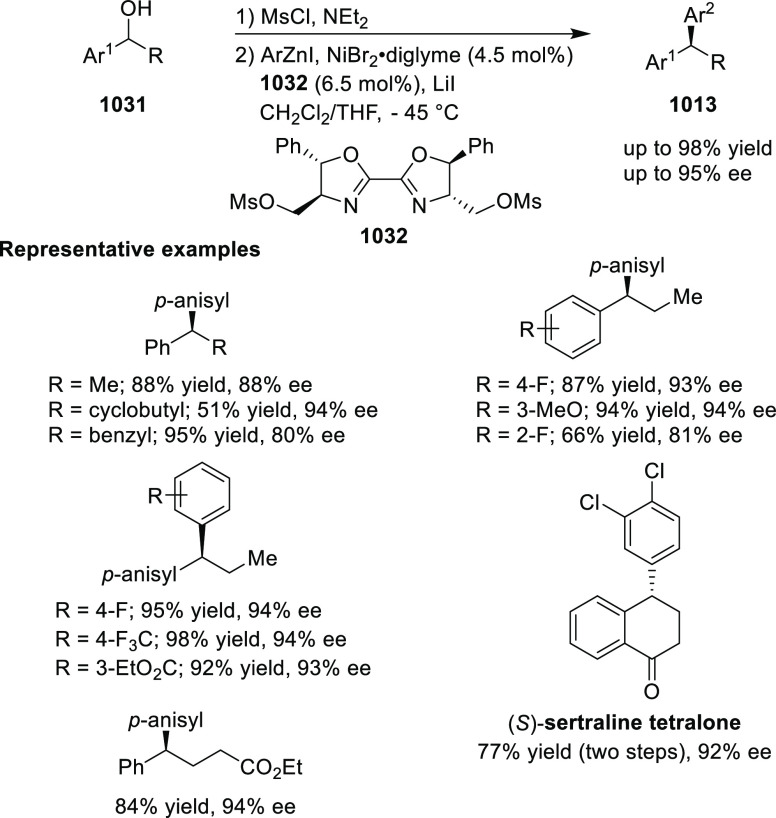
Ni-Catalyzed Negishi Cross-Coupling of Benzyl Mesylates

Fu has developed a Ni-catalyzed Negishi cross-coupling of benzyl mesylates. Employing a disubstituted BiOX ligand **1032** in the reaction between the benzyl alcohols **1031** (which are first converted into the corresponding mesylates) and arylzinc iodides, a wide range of diaryl alkanes **1013** were accessed in good yields up to 98% and with high levels of enantioselectivity up to 95% *ee* (Scheme 314). This transformation was then applied to the asymmetric synthesis of (*S*)-sertraline tetralone, a precursor to the antidepressant drug Zoloft.^362^

Overall, BiOX ligands have been applied in a limited range of Pd- and Ni-catalyzed processes in the past decade. Researchers should seek to continue to expand the use of oxazoline-containing ligands in asymmetric 3d-transition-metal-catalyzed processes, and BiOX ligands have shown great potential for asymmetric induction in new Ni-catalyzed transformations.

#### 3.2.2 Bis(oxazoline) Ligands Directly Connected at *C*_4_

**Figure 44:**
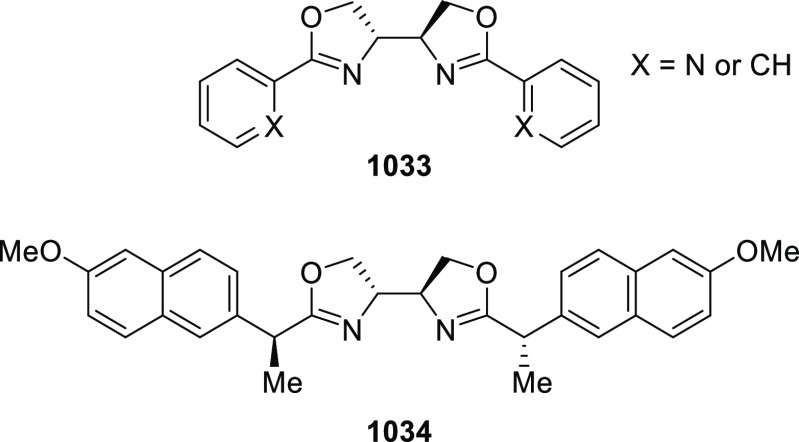
Bis(oxazoline) ligands joined at the *C*_4_-stereocenter.

As is abundantly clear from the numerous examples shown in this Review, bis(oxazoline) ligands most commonly contain amino alcohol derived stereocenters at the *C*_4_-position of the ring. In 1997, Lee reported the synthesis of the novel (*L*)-tartaric acid-derived bioxazoline ligands **1033**, joined at the *C*_4_-chiral center, but these have found limited application in asymmetric catalysis as the chiral information is facing away from the coordination sphere (Figure 44). Kesavan has developed novel bioxazoline ligand **1034**, which is based on Lee’s design, but contains a second chiral center near the coordination sphere, leading to more effective transfer of chiral information. While variations on this design have also been tested, **1034** has emerged as the best ligand in each application reported thus far.

**Scheme 315:**
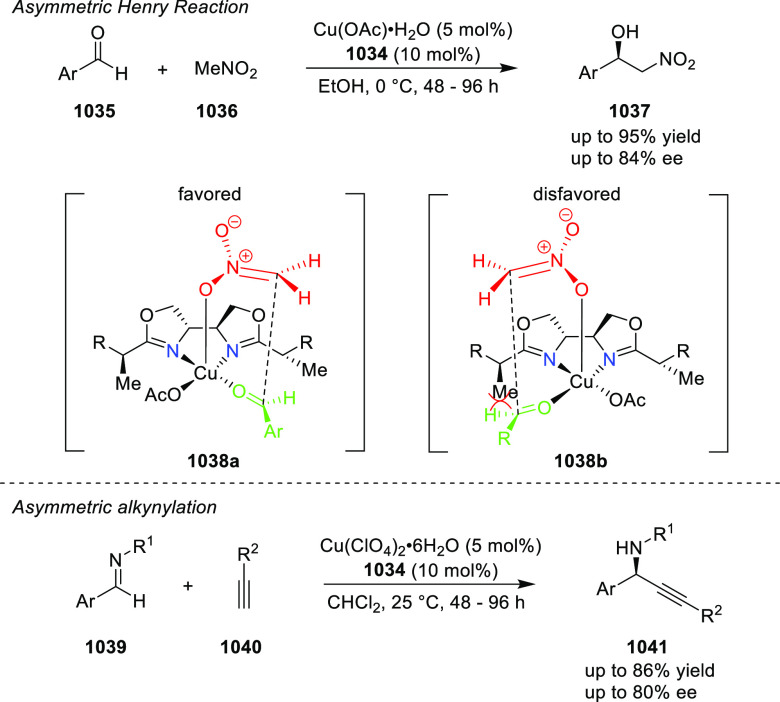
Asymmetric Transformations Mediated by Chiral Bis(oxazoline) Ligand **1034**

Kesevan first applied ligand **1034** in the enantioselective Cu-catalyzed Henry reaction^363^ and the enantioselective Cu-catalyzed alkynylation of imines,^364^ achieving enantiomeric excesses of up to 84% *ee* and 80% *ee*, respectively (Scheme 315). The stereochemical outcome of the asymmetric Henry reaction is described by the two transition states **1038a** and **1038b**. The latter is thought to be disfavored due to the steric interaction between the aldehyde and one of the methyl groups of the ligand.

**Scheme 316:**
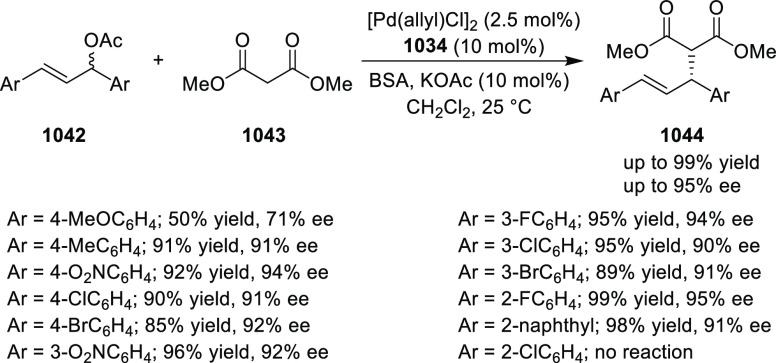
Asymmetric Pd-Catalyzed Alkylation of Allyl Acetates

Kesevan then reported the use of ligand **1034** in the asymmetric Pd-catalyzed alkylation of allyl acetates **1042** by malonate **1043**. A range of symmetrical allyl acetates **1042** bearing different aryl groups were subjected to the reaction leading to the isolation of enantioenriched products **1044** in up to 99% yield and with up to 95% *ee* (Scheme 316). An electron-donating group on the allyl acetate **1042** led to a decrease in reaction yield and enantioselectivity, while various electron-withdrawing groups were well tolerated in all positions of the aryl ring. Subjecting nonsymmetrical allyl acetates to the reaction almost always led to a 1:1 regiomeric mixture of products, with mostly high enantioselectivities.^365^ The scope of this reaction was later extended to include 3-OBoc-oxindoles as the nucleophile with slightly lower levels of enantioselectivity.^366^

The development of bis(oxazoline) ligands connected at the *C*_4_-chiral center remains a significantly underdeveloped area. It is clear from the reports described in this Review that these ligands have the potential to induce high levels of stereoselectivity when a second element of chirality is introduced into the scaffold, and as such, these ligands should be considered by researchers in the future.

#### 3.2.3 Bis(oxazoline) Ligands with Pyrimidine and Pyrazine Linkers

**Figure 45:**
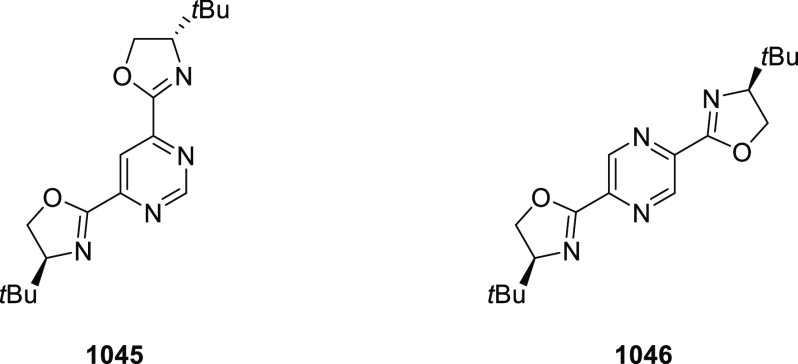
Bis(oxazoline) ligands **1045** and **1046**.

Building on their previous work into the Heck–Matsuda arylation (Scheme 313), Correia and Pfaltz have reported the development of two new classes of bis(oxazoline) ligands, based on pyrimidine- (**1045**) and pyrazino-oxazoline (**1046**) motifs, for use in this transformation (Figure 45). With the aim of using the Heck–Matsuda arylation for the enantioselective synthesis of (*R*)-verapamil, they screened a number of ligands to improve the regioselectivity of the initial arylation reaction. In Correia’s previous report, arylation occurs on a symmetrical allyl diol, giving the lactol intermediate as a single regioisomer, which is then converted to the corresponding lactone by a Jones oxidation. In this report, the diol **1047** is asymmetrical, so an alternative chiral ligand was sought to control the regioselectivity of the process.

**Scheme 317:**
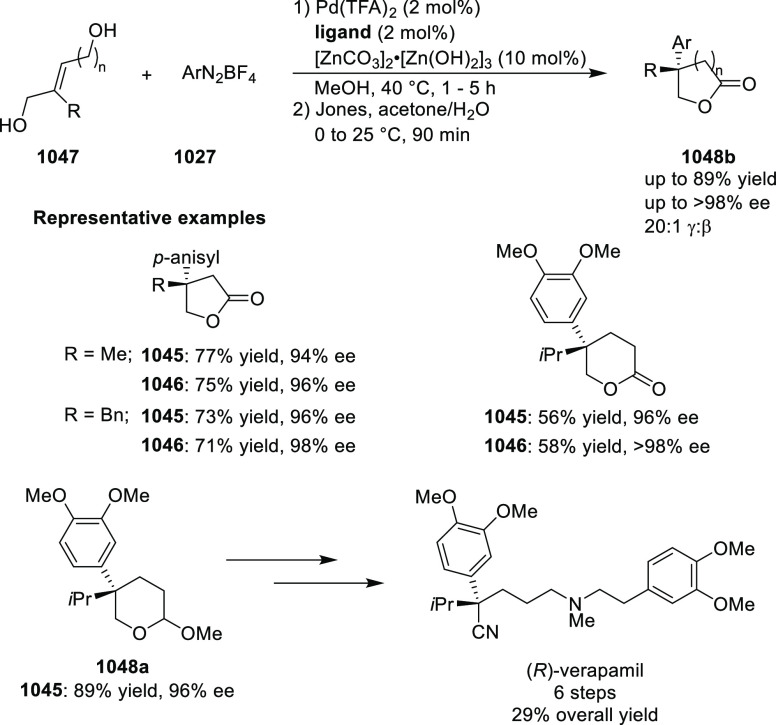
Heck–Matsuda Arylation for Enantioselective Synthesis of (*R*)-Verapamil

Following a screen of ligands, **1045** and **1046** were found to induce high levels of stereo- and regioselectivity, giving the corresponding lactones **1048b** in good yields of up to 89% yield, with high enantioselectivities up to >98% *ee* and with regioselectivities >20:1 γ/β in all cases (Scheme 317). *O*-Methyl lactol **1048a** (the product of the reaction before the Jones oxidation) was synthesized in 89% yield and with 96% *ee*, utilizing pyrimidine ligand **1045**. This was taken forward to synthesize (*R*)-verapamil in 29% overall yield (six steps). Experimental results obtained for the Heck–Matsuda arylation with the mononuclear Pd(TFA)_2_(**1045**) complex and the dinuclear Pd_2_(TFA)_4_(**1045**) complex suggested that the active species in this process is mononuclear.^367^

**Scheme 318:**
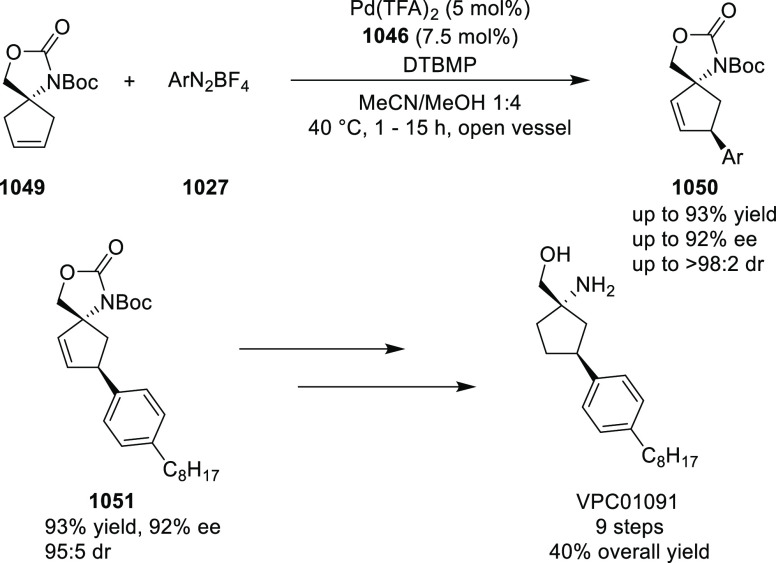
Heck–Matsuda Arylation of Spirocyclic Pyrrolidinones with Aryl Diazonium Tetrafluoroborates

Correia later developed a Heck–Matsuda arylation of spirocyclic pyrrolidinones **1049** with aryl diazonium tetrafluoroborates **1027** generating the corresponding enantioenriched pyrrolidinones **1050** in good yields up to 93%, with high enantioselectivities up to 92% *ee* and with high diastereoselectivities up to >98:2 dr, when PyraBOX ligand **1046** was used (Scheme 318). Pyrrolidinone **1050** was then taken forward to the synthesize S1P1 agonist VPC01091 in 40% overall yield (9 steps) and with 94% *ee*.^368^

**Scheme 319:**
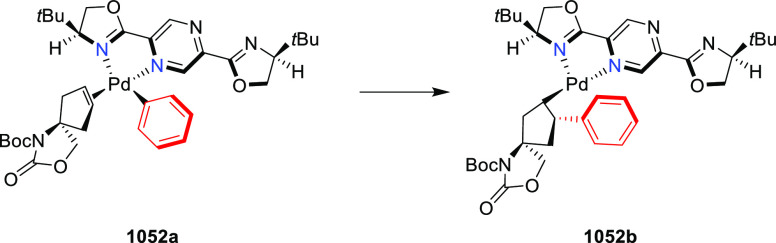
Stereochemical Model for Heck–Matsuda Arylation with Ligand **1046**

The stereochemical outcome of the transformation is explained by the stereoselective migratory insertion of one face of the alkene in **1052a** into the Pd–Ar bond to give **1052b** (Scheme 319). It is proposed that the alkene coordinates to Pd on the opposite face to the carbamate due to the steric bulk of the −*N*Boc group, giving the intermediate **1052a** with the aryl group on the opposite side to the carbamate. PyraBOX **1046** has also been applied to the Heck–Matsuda reaction of five-membered cyclopentene rings containing *S*- and *P*-stereogenic centers with similarly high levels of enantioselectivity.^369^

**Scheme 320:**
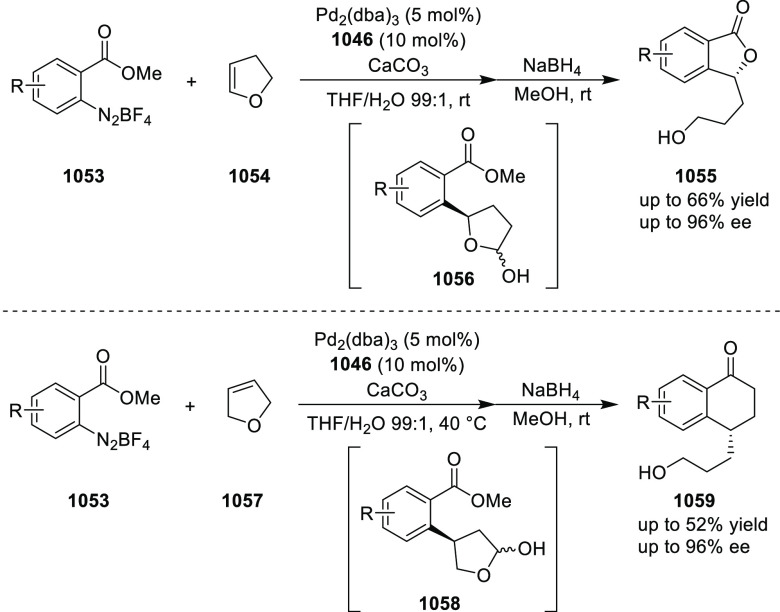
Heck–Matsuda Arylation of Dihydrofurans

Correia has also developed an enantioselective phthalide and isochromanone synthesis via a Heck–Matsuda arylation of dihydrofurans. When 2,3-dihydrofuran **1054** is subjected to the arylation with a range of aryl diazonium tetrafluoroborates **1053**, the corresponding lactols **1056** can then be subjected to NaBH_4_ reduction/cyclization to form enantioenriched phthalides **1055** in moderate yields up to 66% and with high enantioselectivities up to 96% *ee* (Scheme 320). When 3,4-dihydrofurans **1057** are subjected to the same reaction sequence, the corresponding isochromanones **1059** are isolated in up to 52% yield and with up to 96% *ee*.

In recent years, the use of chiral pyrimidine- and pyrazine-linked bis(oxazoline) ligands has been spearheaded by Correia. It is clear that these ligands can be used to induce high levels of enantioselectivity in Pd-catalyzed processes. In future, these unique ligands could be further developed for other transition-metal-catalyzed asymmetric transformations. In particular, the binding mode of these ligands could be suitable for developing chiral nickel catalysts.

#### 3.2.4 Bis(oxazoline) Ligands with Spirocyclic Linkers

**Figure 46:**
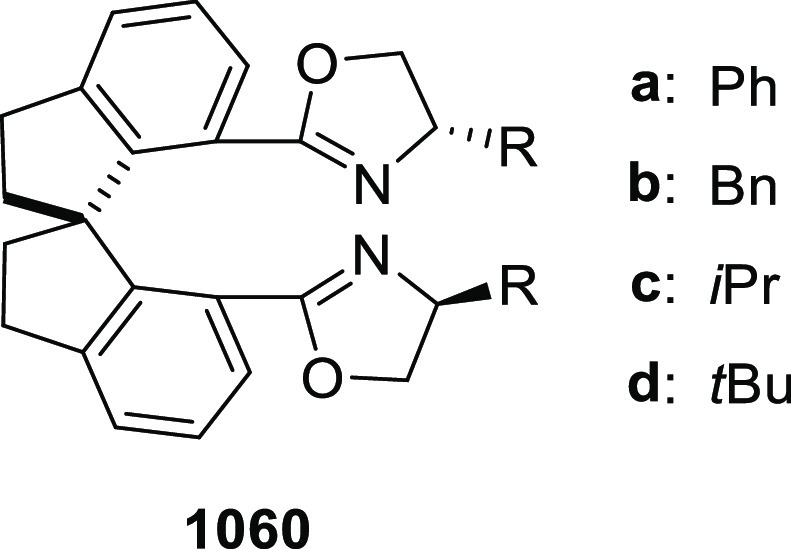
SpiroBOX ligands **1060**.

The SpiroBOX ligands **1060** have been developed and applied in the asymmetric insertion of carbenoids into O–H bonds by Zhou (Figure 46).

**Scheme 321:**
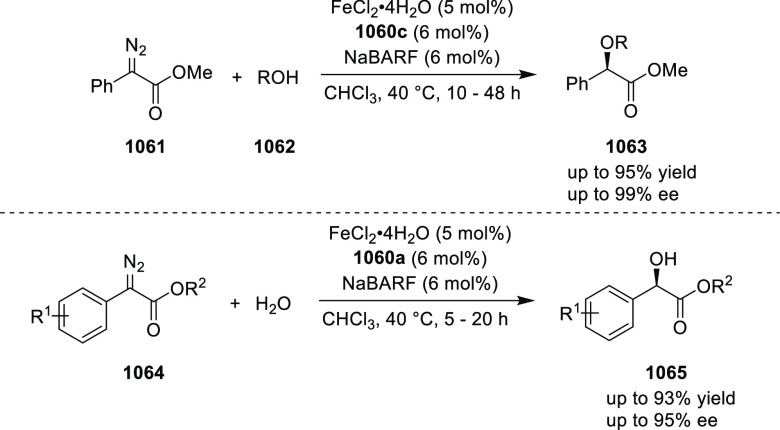
Asymmetric Fe-Catalyzed O–H Insertion

By utilizing a chiral bis(oxazoline) ligand with a highly rigid backbone, Zhou thought that tighter chelation would give access to successful Fe-catalyzed processes. Subjecting diazo compound **1061** to optimized conditions for Fe-catalyzed O–H insertion, with a range of alcohols **1062**, led to the isolation of enantioenriched benzyl ethers **1063** in up to 95% yield and with up to 99% *ee*, with *i*Pr-SpiroBOX **1060c** as the chiral ligand (Scheme 321). Subjecting a range of diazo compounds **1064** to the reaction with water led to the isolation of enantioenriched benzyl alcohols **1065** in up 93% yield and with up to 95% *ee*, when Ph-SpiroBOX **1060a** was used as chiral ligand. Almost all examples are >90% *ee*, with *ortho*-substituted diazo compounds giving lower levels of enantioselectivity.^370^

**Scheme 322:**
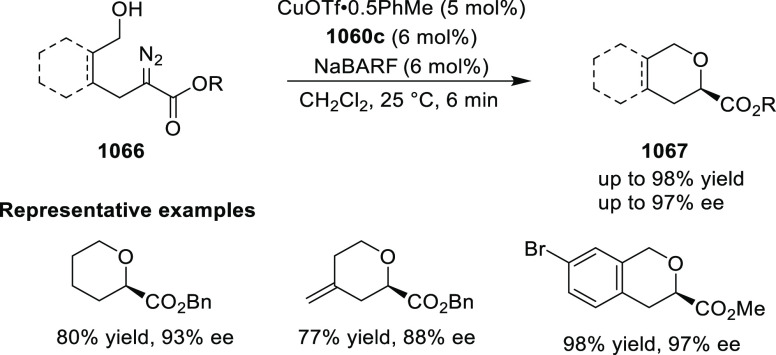
Asymmetric Cu-Catalyzed Carbenoid O–H Insertion

Zhou has applied their Cu-catalyzed O–H insertion chemistry to the enantioselective synthesis of 2-carboxy cyclic ethers **1067**. Employing *i*Pr-SpiroBOX ligand **1060c**, enantioenriched 2-carboxy cyclic ethers **1067** can be accessed by an intramolecular carbenoid O–H insertion of diazo compounds **1066** in up to 98% yield and with up to 97% *ee* (Scheme 322).^371^ Zhou has applied the SpiroBOX ligands **1060** to asymmetric Cu-catalyzed α-diazo phosphonate carbenoid O–H insertions^372^ and Pd-catalyzed carbenoid insertions into phenol O–H bonds.^373^

**Scheme 323:**
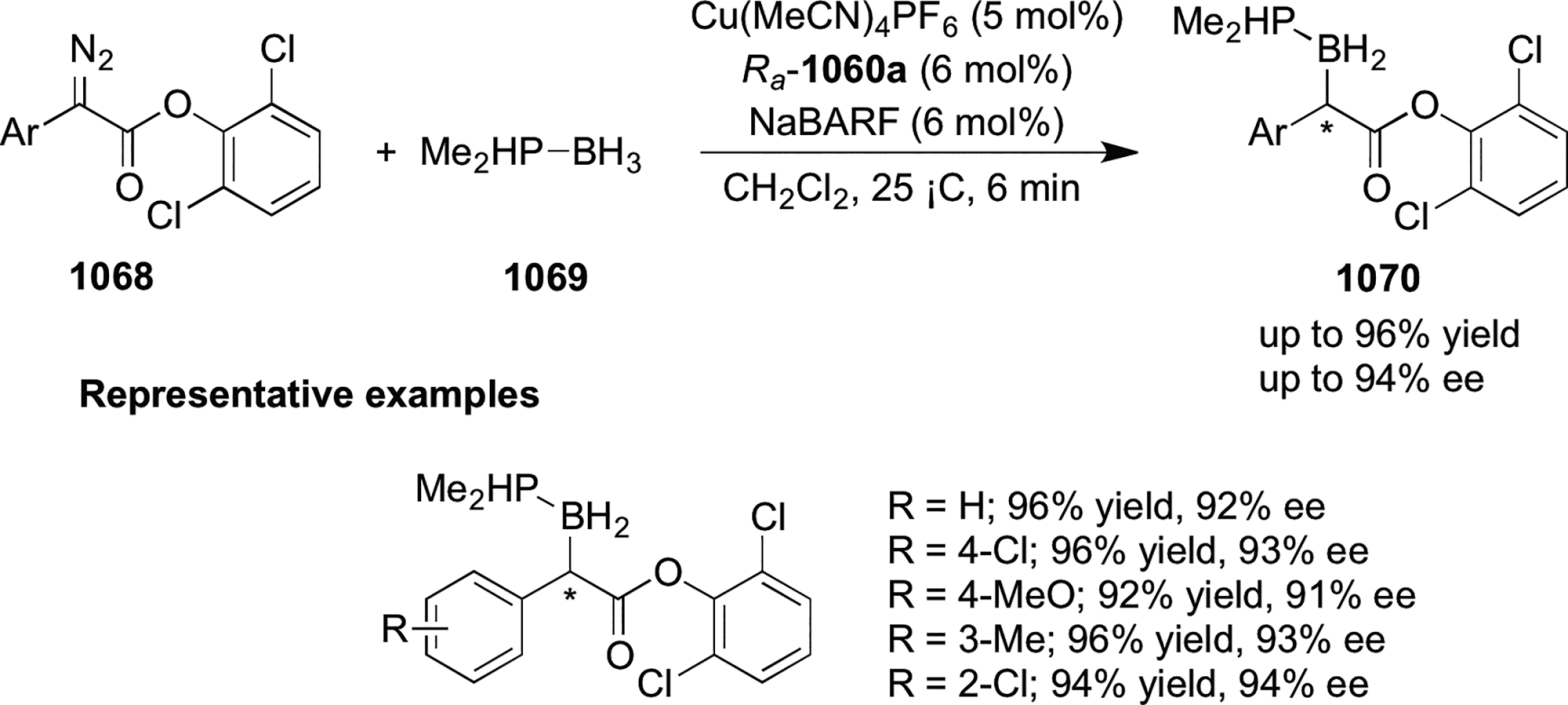
Cu-Catalyzed Carbenoid β–H Insertion

Zhou has also extended the application of the SpiroBOX ligands **1060** to include other X-H bonds. Subjecting the dimethylphosphorus-borane adduct **1069** to a Cu-catalyzed carbenoid β-H insertion, utilizing ligand (*R*_*a*_)*-***1060a**, a range of enantioenriched phosphorus-borane adducts were accessed in high yields up to 96% and with high enantioselectivities up to 94% *ee* (Scheme 323). The stereochemical outcome of the reaction required very specific 2,6-dichlorophenyl α-diazo esters **1068**.^374^

**Scheme 324:**
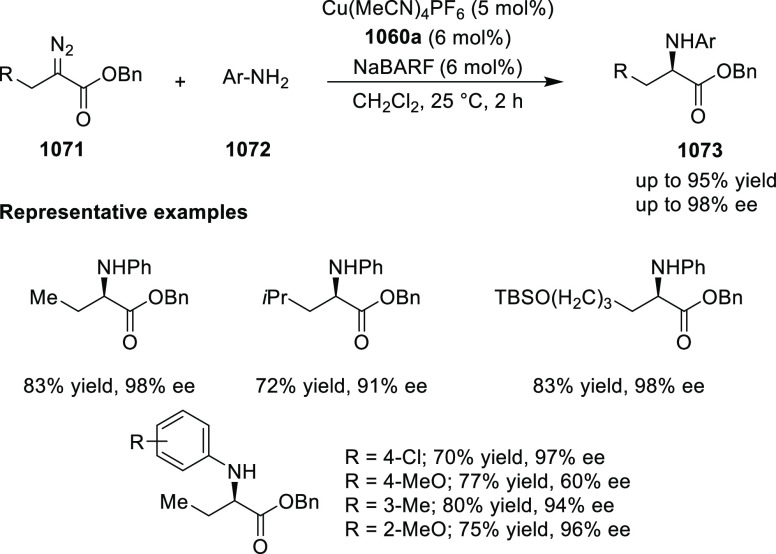
Asymmetric Cu-Catalyzed Carbenoid *N*–H Insertion

Zhou extended the enantioselective Cu-catalyzed carbenoid insertion reaction to include *N*-H bonds. A range of α-alkyl-α-diazo esters **1071** were reacted with aniline derivatives **1072** to access a range of enantioenriched secondary amines **1073** in up to 95% yield and with up to 98% *ee* (Scheme 324). Most of the aniline derivatives performed well in the reaction except *p*-anisidine which gave the product with only 60% *ee*. The active catalytic species was found to be a binuclear [Cu_2_(**1060a**)_2_] species.^375^

**Scheme 325:**
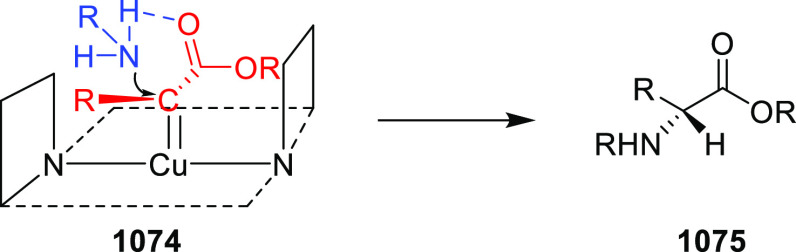
Stereochemical Model for Asymmetric Cu-Catalyzed *N*–H Insertion with Ligand **1060a**

They proposed a stereochemical model based on this dinuclear Cu-species with a perfect *C*_2_-symmetric pocket (Scheme 325).

Zhou has also applied an intramolecular Cu/**1060a**-catalyzed carbenoid *N*-H insertion reaction to the enantioselective synthesis of 2-carboxytetrahydroquinolines.^376^

**Scheme 326:**
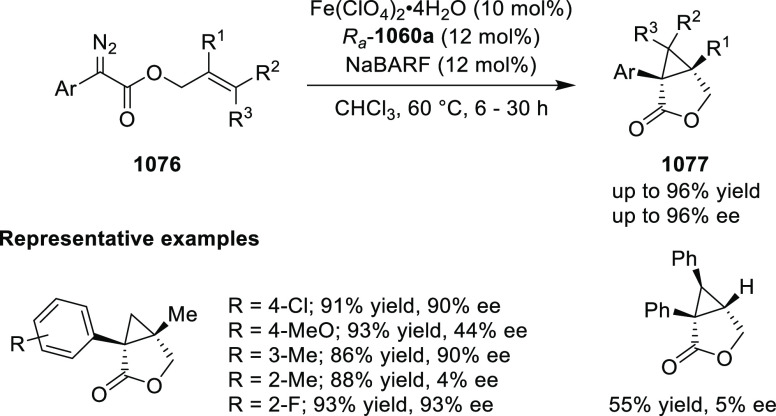
Intramolecular Fe-Catalyzed Asymmetric Cyclopropanation of Alkenes

Zhou has described an intramolecular Fe-catalyzed asymmetric cyclopropanation of alkenes with carbenoids. Applying **1060a** in this reaction, a range of enantioenriched cyclopropanes **1077** were accessed in high yields up to 96% and with low to high enantioselectivities up to 96% *ee* (Scheme 326).^377^

**Figure 47:**
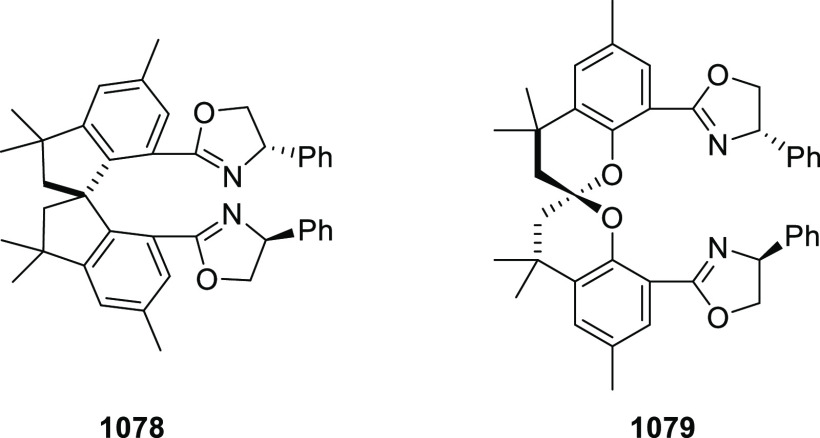
HMSI-BOX **1078** and SpanBOX **1079**.

They have also reported an Fe/**1060a**-catalyzed carbenoid indole *C*-H insertion, but with only low to moderate enantioselectivities.^378^ Other bis(oxazoline) ligands with spirocyclic backbones, like HMSI-BOX **1078** and SpanBOX **1079** have been reported (Figure 47).

**Scheme 327:**
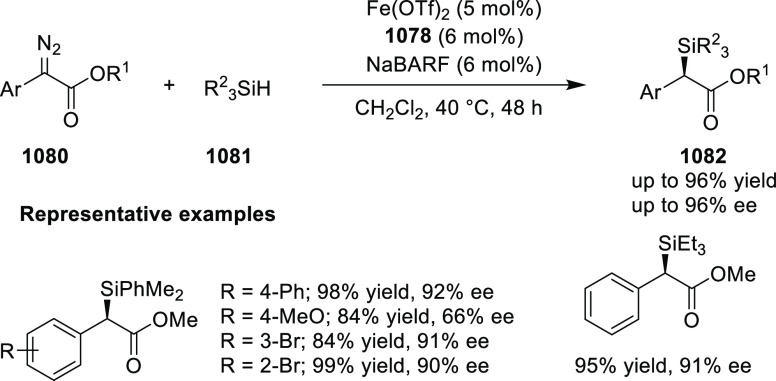
Asymmetric Fe-Catalyzed Carbenoid *Si*–H Insertion

Xie and Lin have reported the use of spirocyclic HMSI-BOX ligand **1078** in the Fe-catalyzed carbenoid *Si*-H insertion. A range of silanes **1081** were reacted with α-aryl-α-diazo esters **1080** to give the corresponding enantioenriched silanes in up to 99% yield and with up to 96% *ee* (Scheme 327). During optimization, **1078** gave the product of the model reaction with 91% *ee*, compared to SpiroBOX ligand (*R*_*a*_)-**1060a**, which gave the product in 84% *ee*. As in the other carbenoid insertion reactions, 4-methoxy-substitution on the α-aryl-α-diazo esters **1080** leads to a dramatic decrease in enantioselectivity, in this case to 66% *ee*.^379^

**Scheme 328:**
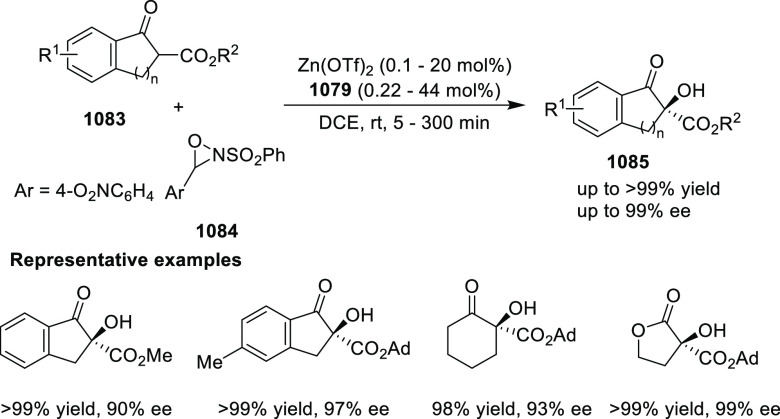
Asymmetric Zn-Catalyzed α-Hydroxylation of β-Keto Esters

Ding has applied SpanBOX **1079** in the Zn-catalyzed α-hydroxylation of β-keto esters **1083** with racemic oxaziridines **1084** to give the enantioenriched α-hydroxy-β-keto esters **1085** in up to >99% yield and with up to 99% *ee* (Scheme 328). A range of substituted β-keto esters were well tolerated with all reported transformations proceeding with >98% yields and >90% *ee*. The majority of substrates were successfully hydroxylated using 1 mol % Zn(OTf)_2_ and 2.2 mol% of ligand **1079**.^380^ Ding also reported the Zn- and Cu-catalyzed α-chlorination of the same β-keto esters, utilizing **1079** with similar results.^381^

Overall, bis(oxazoline) ligands with spirocyclic backbones are clearly an optimal choice for asymmetric transformations of carbenoids with both iron and copper catalysts. Researchers developing these types of transformations in the future should consider spirocyclic bis(oxazoline) ligands. More importantly, these ligands could be used to develop new asymmetric Fe-catalyzed processes.

#### 3.2.5 Bis(oxazoline) Ligands with One Boron Separating the Oxazoline Rings

**Figure 48:**
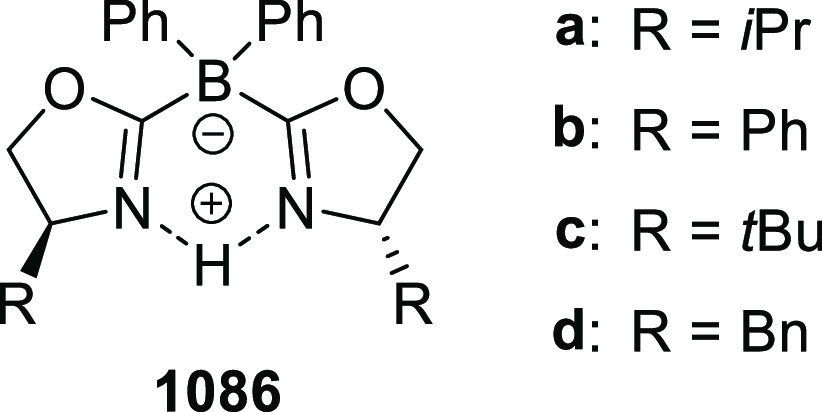
BoraBOX ligands **1086**.

Pfaltz pioneered the use of bis(oxazoline) ligands linked by a boron atom, like the BoraBOX ligands **1086a**–**d**, in asymmetric catalysis (Figure 48).^382,383^

**Figure 49:**
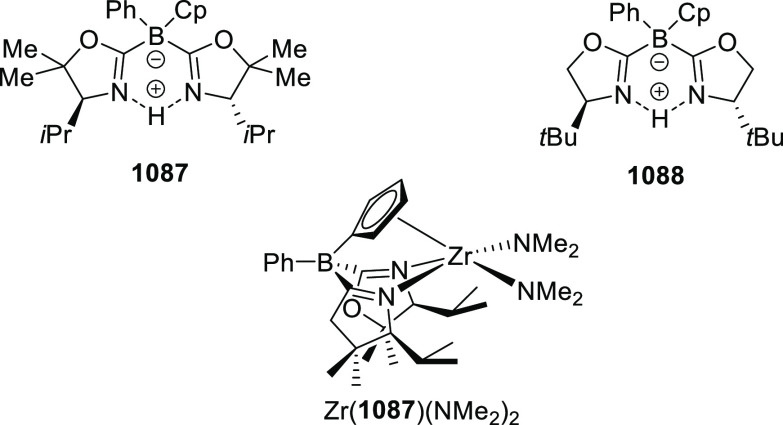
Boron-bridged bis(oxazoline) ligandsand Zr(**1087**)(NMe_2_)_2_.

More recently, Sadow has developed boron-bridged bis(oxazoline) ligands **1087** and **1088** for use in enantioselective transition metal-catalyzed hydroaminations of alkenes (Figure 49). The former ligand exploits the gem-dimethyl effect which forces the *i*Pr-containing ligand to behave like a *t*Bu-substituted ligand. The ligands form complexes like Zr(**1087**)(NMe_2_)_2_, in the case of Zr, in which the Cp ring participates in bonding to the metal center.

Sadow has reported detailed studies on the effects of different catalysts, based on Zr, Y, Ti and Hf, which incorporate these BoraBOX ligands.^384−386^ As an example, a comparison of different BoraBOX precatalysts on the outcome of the hydroamination/cyclization of aminoalkene **1089** to give enantioenriched pyrrolidine **1090** is presented in Table 5. Both the Zr(**1087**)(NMe_2_)_2_ and Hf(**1087**)(NMe_2_)_2_ complexes were found to be efficient catalysts in this transformation, giving the product **1090** in 95% and 98% yield, and with 93% and 97% *ee*, respectively, with the Hf-catalyst reaction performed at 0 °C (entries 1 and 4). Interestingly, Ti(**1087**)(NMe_2_)_2_ was found to operate at a much lower rate of reaction, and gave the product **1090** in much lower enantioselectivity of 76% *ee* (entry 5). Therefore, the metal center affects the rate of the reaction, such that Zr > Hf ≫ Ti. It is also apparent that the ancillary ligand **1087** or **1088** has a positive effect on the rate of the reaction, such that Zr(**1087**)(NMe_2_)_2_ (1.25 h) > Zr(**1088**)(NMe_2_)_2_ (18 h), but not the enantioselectivity of the transformation (entry 6). Interestingly, Y(**1088**)(CH_2_SiMe_3_) proved to be an effective catalyst, giving the opposite enantiomer (*S*) in 100% yield and 94% *ee* in only 10 min (entry 7).

**Table 5:**
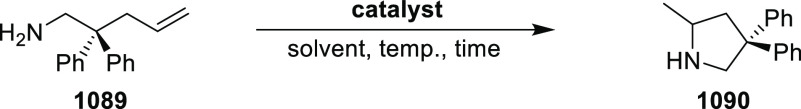
Asymmetric Hydroamination/Cyclization of Aminoalkene **1089** with Ligands **1087** and **1088**

entry	precatalyst^a^	solvent	temp (°C)	time	yield (%)^b^	*ee* (%)
1	Zr(*S*-**1087**)(NMe_2_)_2_	C_6_H_6_	25	1.25 h	95	93 (*R*)
2	Zr(**1087**)(NMe_2_)_2_	C_7_H_8_	–30	5 d	98	98 (*R*)
3	Zr(*R*-**1087**)(NMe_2_)_2_	C_6_H_6_	25	1.25 h	95	93 (*S*)
4	Hf(**1087**)(NMe_2_)_2_	C_7_H_8_	0	15 h	98	97 (*R*)
5	Ti(**1087**)(NMe_2_)_2_	C_6_D_6_	25	5 d	93	76 (*R*)
6	Zr(**1088**)(NMe_2_)_2_	C_6_H_6_	25	18 h	95	93 (*R*)

a For Zr, Hf and Ti: 10 mol % precatalyst, for Y: 5 mol % precatalyst.

b Determined by ^1^H NMR spectroscopic analysis of the crude reaction mixture.

**Scheme 329:**
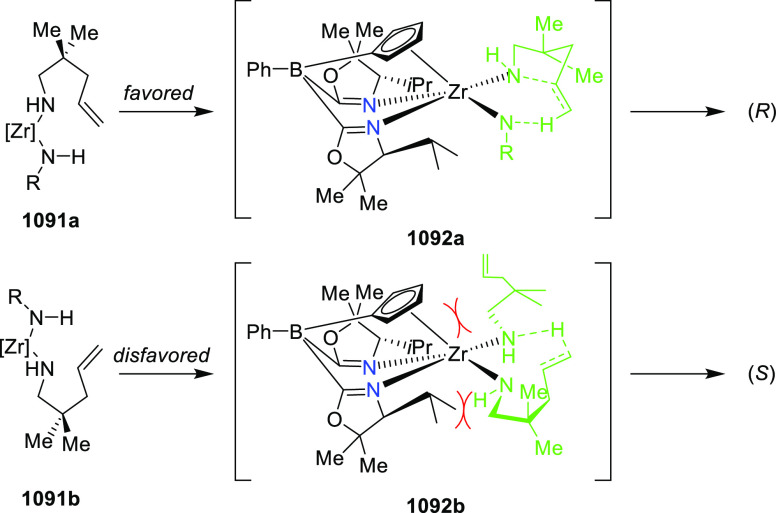
Stereochemical Model to Explain the Outcome of Cyclization Catalyzed by Zr(**1087**)(NMe_2_)_2_ Precatalyst

Sadow proposed a model to explain the stereochemical outcome of the cyclization catalyzed by the Zr(**1087**)(NMe_2_)_2_ precatalyst (Scheme 329). From their experimental results, they proposed that optimal levels of enantioselectivity could be obtained by using a 2:1 substrate/catatlyst ratio. The coordination of the substrates to the Zr-catalyst occurs, and the cyclization of one Zr-amide is facilitated by the other. Because of the steric interactions in the transition states depicted below, only one of the amides cyclizes to selectively give the (*R*)-product.

The application of oxazoline-containing ligands in asymmetric Zr-catalyzed transformations is limited. Clearly, the boron-linked bis(oxazoline) ligands described in this Review represent a foundation for the development of more Zr-catalyzed asymmetric transformations. The unique structure of these ligands could also be useful for developing novel chemistry with other metals.

#### 3.2.6 Bis(oxazoline) Ligands with One Nitrogen Separating the Oxazoline Rings

**Figure 50:**
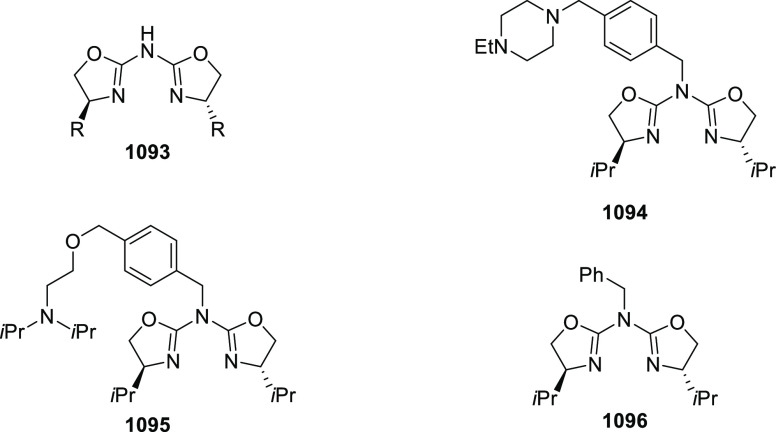
AzaBOX ligands.

Reiser has developed a related class of bis(oxazoline) ligand, azaBOX, in which there is a central bridging amine (Figure 50).^387^ The standard azaBOX ligand **1093** has a simple secondary amine bridge, but this amine is an excellent functional handle for further derivatization, and it has been used to create polymer-supported azaBOX ligands, but this is outside the scope of this Review.

The central amine has been used by Hong to develop a base-functionalized azaBOX ligand **1094** for use in bifunctional catalysis. Hong compared the activity of benzyl-substituted azaBOX **1096** and bifunctional piperazine-substituted azaBOX **1094** in the Cu-catalyzed Henry reaction of 2-anisaldehyde **1097** with nitromethane **1036** for the asymmetric synthesis of alcohol **1098**. In the reaction with **1096** as the chiral ligand, 1-benzyl-4-ethyl-piperazine **1099** was added as a base. When 2 mol% of **1099** was used with 2 mol% **1096** and 2 mol% CuTC, the alcohol **1098** was furnished in 56% yield and with 95% *ee* (Table 6, entry 1). Increasing the amount of base was found to increase the yield of the reaction but slightly decrease the enantioselectivity (30 mol % **1099**: 85% yield, 90% *ee*) (entry 2). When 2 mol% of **1094** was used with 2 mol% CuTC, a significant rate acceleration was observed, furnishing the alcohol **1098** 90% yield and with 92% *ee* (entry 3).

**Table 6:**
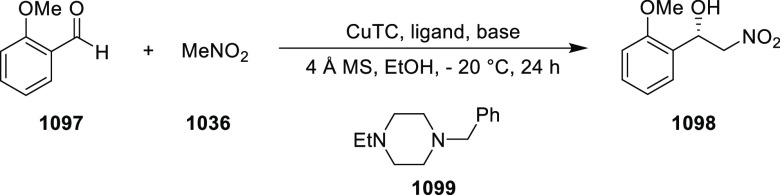
Asymmetric Cu-Catalyzed Henry Reaction of 2-Anisaldehyde **1097** with Nitromethane **1036**

entry	CuTC mol %	ligand (mol %)	base (mol %)	yield (%) **1098**	% *ee*
1	2 mol %	**1096** (2 mol %)	**1099** (2 mol %)	56	95
2	2 mol %	**1096** (2 mol %)	**1099** (30 mol %)	85	90
3	2 mol %	**1094** (2 mol %)		90	92

Applying the **1096**/**1099** system in the Cu-catalyzed Henry reaction of 2-anisaldehyde **1097** with nitroethane **1100** led to the isolation of alcohol **1101** in 54% yield, 1.3:1 *syn*/*anti*, and with 72% *ee* (*syn*)/96% *ee* (*anti*) (Table 7, entry 1). The same reaction with piperazine-azaBOX **1094** gave alcohol **1101** in 81% yield, 1.3:1 *syn*/*anti*, and with 92% *ee* (*syn*)/97% *ee* (*anti*), an overall significant improvement in the yield and enantioselectivity (entry 3). A di-*iso*-propylamine-functionalized azaBOX ligand **1095** was also applied in this reaction, giving alcohol **1101** in 99% yield, 1.5:1 *syn*/*anti*, and with 92% *ee* (*syn*)/96% *ee* (*anti*), these results indicated that ligand **1095** is an overall more efficient ligand than **1094** for this transformation. However, neither bifunctional ligand **1094** nor **1095** have a significant advantage over the combination of unfunctionalized azaBOX **1096** with DIPEA in this reaction, which gave alcohol **1101** in 96% yield, 1.6:1 *syn*/*anti*, and with 94% *ee* (*syn*)/95% *ee* (*anti*) (entry 2). While the bifunctional ligand systems do not give largely improved outcomes for the Henry reaction over the unfunctionalized ligand/base systems, they are excellent additions to the library of bis(oxazoline) ligands for the application in new enantioselective catalytic transformations.^388^

**Table 7:**
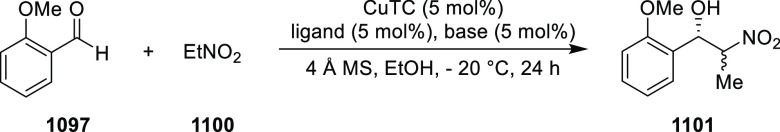
Asymmetric Cu-Catalyzed Henry Reaction of 2-Anisaldehyde **1097** with Nitromethane **1036**

entry	ligand	base	yield (%) **1101**	*syn*/*anti*	% *ee* (*syn*/*anti*)
1	**1096**	**1099**	54	1.3:1	72/96
2	**1096**	DIPEA	96	1.6:1	94/95
3	**1094**		81	1.3:1	92/97
4	**1095**		99	1.5:1	92/96

**Scheme 330:**
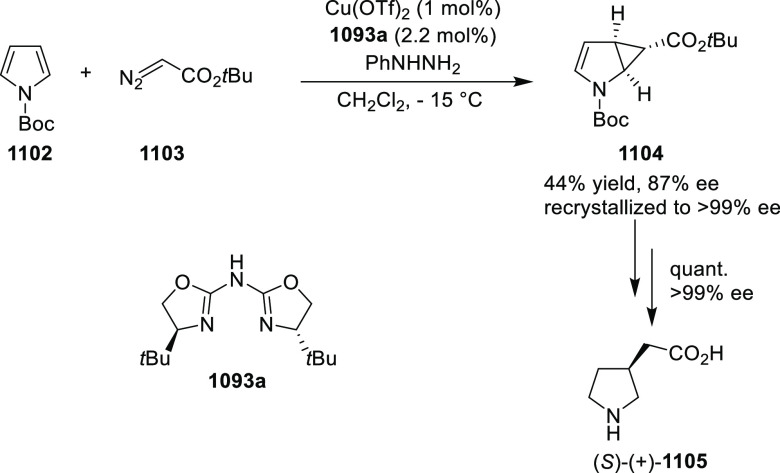
Enantioselective Synthesis of GABA Uptake Inhibitor (+)-Homo-β-proline **1105**

Reiser has achieved an enantioselective synthesis of the GABA uptake inhibitor (+)-homo-β-proline **1105** via a key Cu-catalyzed pyrrole cycloproponation. Utilizing *t*Bu-azaBOX **1093a**, cyclopropane **1104** was accessed in 44% yield and with 87% *ee*, which can be recrystallized up to >99% *ee* (Scheme 330). (+)-Homo-β-proline **1105** was then synthesized in three further synthetic steps, all quantitative with regards to the reaction yield, and with >99% *ee*.^389^

Overall, azaBOX ligands have not received significant attention in the past decade. Clearly, they can be used for inducing high levels of enantioinduction, and the free-amine has been shown to be a useful functional handle for accessing not only novel ligand structures but bifunctionality as well.

#### 3.2.7 *C*_2_-Symmetric Bis(oxazoline) Ligands with Diphenylamine Linkers

**Figure 51:**
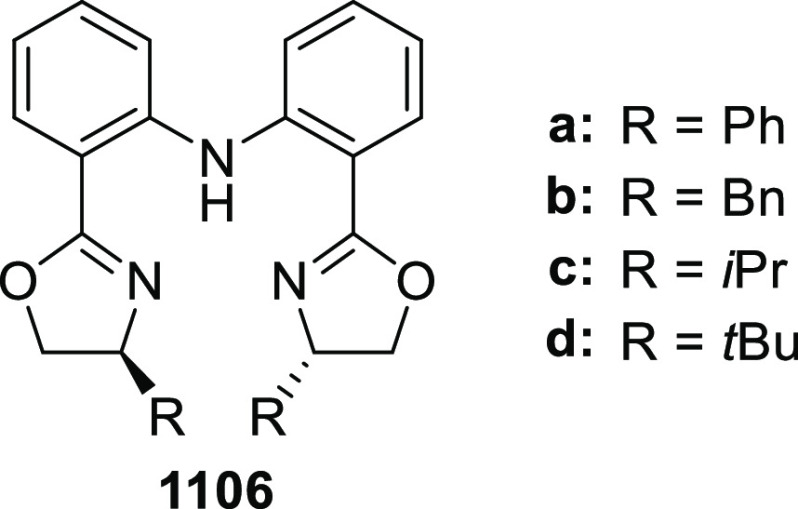
Diphenylamine-linked bis(oxazoline) ligands **1106**.

Since they were first developed by Guiry, diphenylamine-linked bis(oxazoline) ligands of the type **1106a**–**d** have been applied in a diverse range of asymmetric transformations (Figure 51).^390^ Ligands **1106** can be both bidentate (through the oxazoline moieties) and tridentate (through the oxazoline moieties and the bridging amine), depending on what metal ion the ligand is coordinated to.

**Scheme 331:**
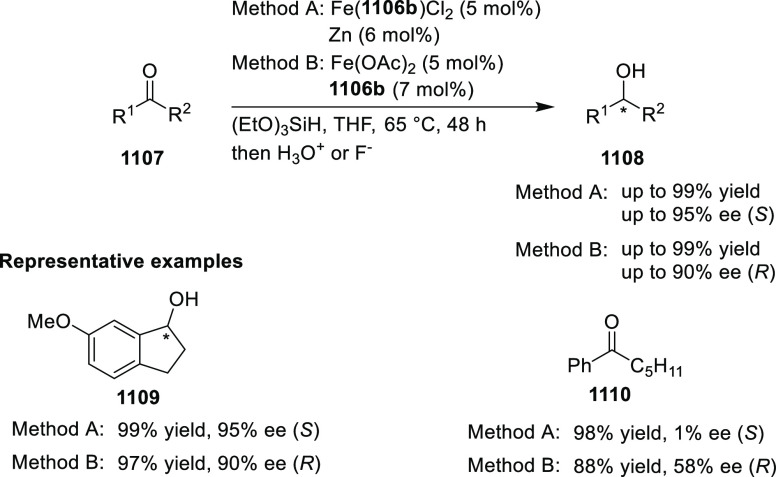
Asymmetric Fe-Catalyzed Hydrosilylation of Ketones

Nishiyama has applied Bn-ligand **1106b** in the Fe-catalyzed hydrosilylation of ketones **1107** for the asymmetric synthesis of secondary alcohols **1108**. Under two sets of optimized conditions, a range of enantioenriched alcohols **1108** were accessed in up to 99% yield and with up to 95% *ee* (*S*) or up to 99% yield and 90% *ee* (*R*) (Scheme 331). The stereoselectivity varied depending on whether the preprepared Fe(**1106b**)Cl_2_ catalyst and Zn were used in the reaction (method A), or the free ligand **1106b** was precomplexed with Fe(OAc)_2_ directly in the reaction mixture without any Zn (method B). The major difference is the presence of Zn in method A. Both methods gave better levels of enantioselectivity depending on the substrate. For example, in the formation of alcohol **1109**, method A gave **1109** in 95% *ee* (*S*) as compared to method B, which gave **1109** in 90% *ee* (*R*). However, in the case of **1110**, method A gave essentially racemic alcohol **1110** (1% *ee* (*S*)) while method B gave **1110** in 58% *ee* (*R*). Analytical results for the complexes with and without the Zn additive suggested that the Zn was acting to reduce Fe(III) to Fe(II), however no active species has been characterized as of yet.^391^ Nishiyama has also applied Bn-ligand **1106b** and *i*Pr-ligand **1106c** in the Fe-catalyzed and Co-catalyzed hydrosilylation of ketones and Co-catalyzed hydrosilylation of enones.^392^

**Scheme 332:**
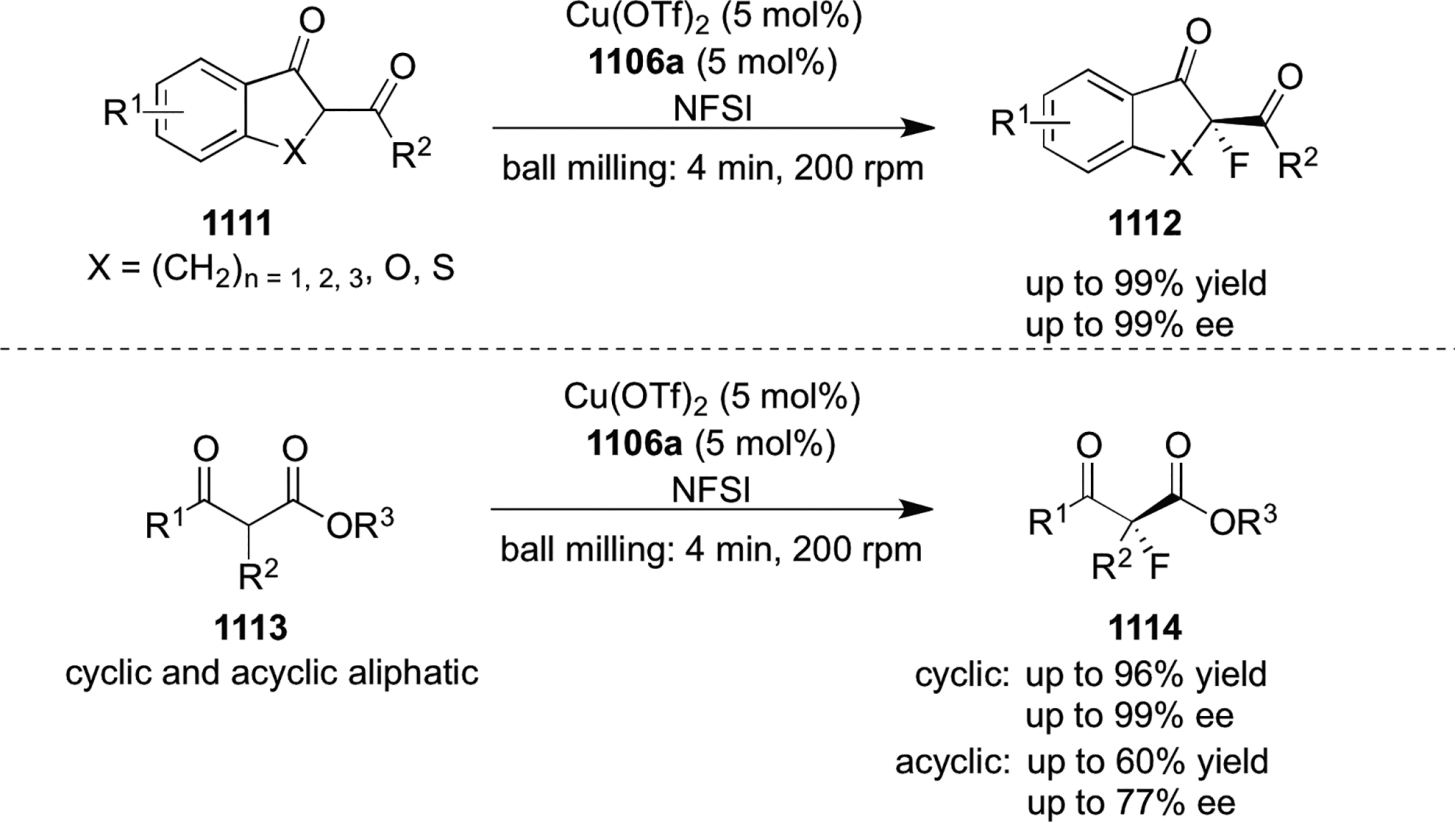
Asymmetric Cu-Catalyzed Solvent-Free α-Fluorination of β-Keto Esters/Amides

A Cu/**1106a**-catalyzed solvent-free α-fluorination of β-keto esters/amides **1111** with NSFI, mediated by ball-milling, has been reported by Xu. A range of indanone-, tetralone-, benzosuberone-, benzofuran-3-(2*H*)-one-, and benzothiophen-3-(2*H*)-one-based β-keto esters/amides were successfully fluorinated to give the enantioenriched products **1112** in up to 99% yield and with up to 99% *ee* (Scheme 332). Cyclic aliphatic β-keto esters **1113** were also subjected to the α-fluorination to give the products **1114** in up to 96% yield and 99% *ee*, while acyclic aliphatic β-keto esters gave the products with lower levels of enantioselectivity up to 75% *ee*.^393^

**Scheme 333:**
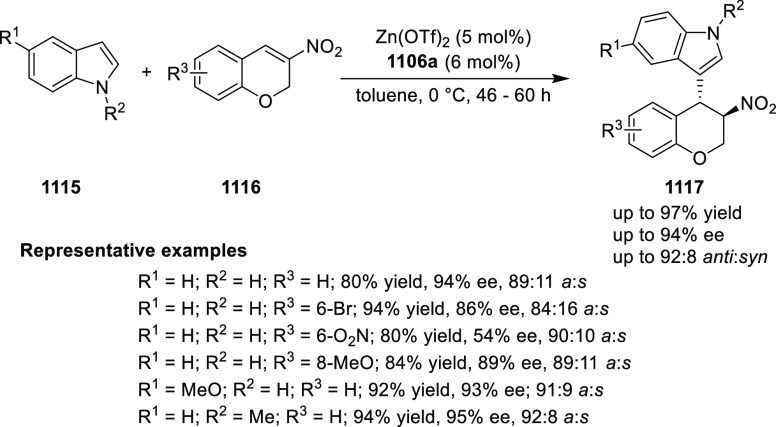
Asymmetric Zn-Catalyzed Friedel–Crafts Alkylation of Indoles with 3-Nitro-2*H*-chromenes

Du has described the asymmetric Zn-catalyzed Friedel–Crafts alkylation of indoles **1115** with 3-nitro-2*H*-chromenes **1116**, utilizing the Ph-ligand **1106a**. The products **1117** were isolated in up to 97% yield, with up to 94% *ee* and with up to a 92:8 *anti*/*syn* ratio (Scheme 333).^394^

**Scheme 334:**
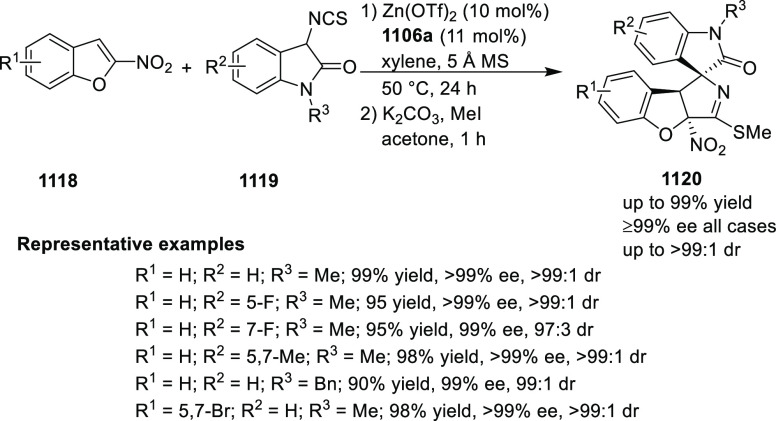
Asymmetric Zn-Catalyzed Dearomative [3 + 2] Cycloaddition of 2-Nitrobenzofurans and 3-Isocyanato Oxindoles

Yuan has reported an asymmetric Zn-catalyzed dearomative [3 + 2] cycloaddition of 2-nitrobenzofurans **1118** and 3-isocyanato oxindoles **1119**. Employing Ph-ligand **1106a** in the process, followed by treatment with MeI in acetone, a range of spirocyclic products **1120**, containing three contiguous stereocenters, were accessed in up to 99% yield, with ≥99% *ee* in all cases and with up to >99:1 dr. (Scheme 334).^395^ Yuan later extended the scope of this reaction to include 2-nitroindoles.^396^

**Scheme 335:**
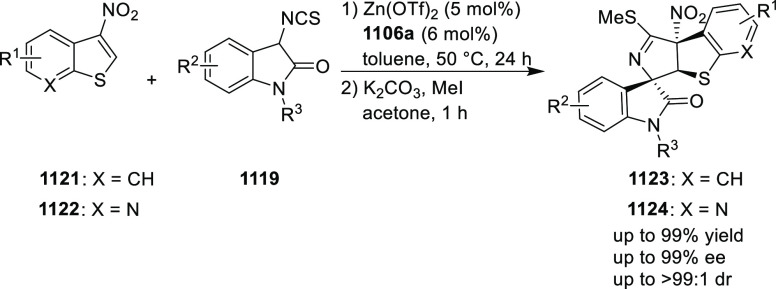
Enantioselective Zn-Catalyzed Dearomative [3 + 2] Cycloaddition of 3-Nitrobenzothiophenes/3-Nitrothiopheno[2,3-*b*]pyridines with 3-Isocyanato Oxindoles

Yuan has also described an enantioselective Zn-catalyzed dearomative [3 + 2] cycloaddition of 3-nitrobenzothiophenes **1121** and 3-nitrothiopheno[2,3-*b*]pyridines **1122** with 3-isocyanato oxindoles **1119**. Again, utilizing Ph-ligand **1106a** under similar optimized conditions to the previous reactions, a range of spirocyclic products **1123**/**1124** containing three contiguous stereocenters were accessed in up to 99% yield, with up to >99% *ee* and with up to >99:1 dr (Scheme 335).^397^

**Figure 52:**
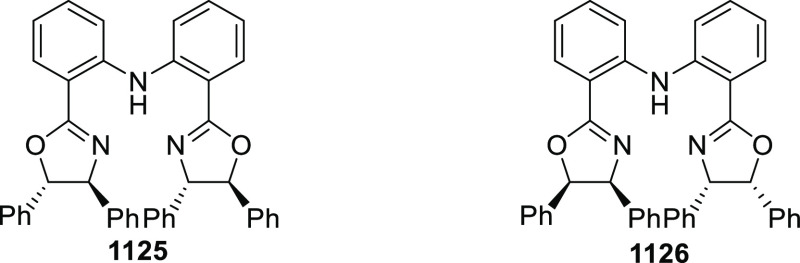
DiPh-Diphenylamine-Linked Bis(oxazoline) Ligands.

Xu and Yuan have reported the application of diPh-ligand (*R*,*R*)-**1125** in the asymmetric Zn-catalyzed dearomative [3 + 2] cycloaddition of 3-nitroindoles **1127** with 3-isocyanato oxindoles **1119** (Figure 52).

**Scheme 336:**
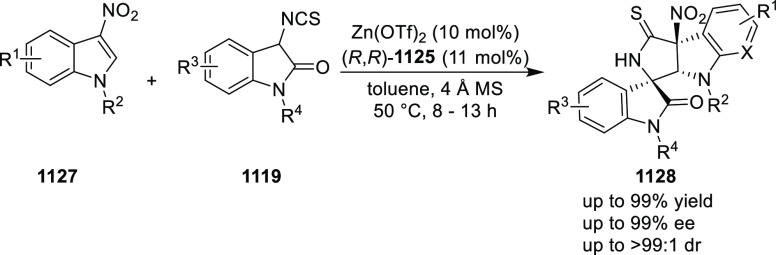
Asymmetric Zn-Catalyzed Dearomative [3 + 2] Cycloaddition of 3-Nitroindoles with 3-Isocyanato Oxindoles

The spirocyclic products **1128** were isolated in up to 99% yield, with up to 99% *ee* and with up to 99:1 dr (Scheme 336).^398^

**Scheme 337:**
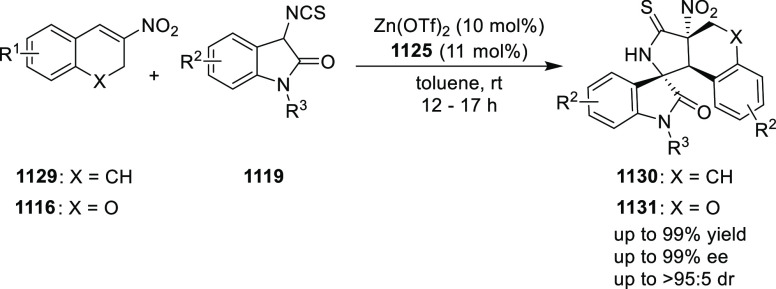
Enantioselective [3 + 2] Cycloaddition of 3-Nitro-2*H*-chromenes with 3-Isocyanato Oxindoles

Chen and Xiao have also applied ligand **1125** in the enantioselective [3 + 2] cycloaddition of 3-nitro-2*H*-chromenes **1129**/**1116** with 3-isocyanato oxindoles **1119** to give the spirocyclic products **1130**/ **1131** in up to 99% yield, with up to 99% *ee* and >99:5 dr (Scheme 337).^399^

**Scheme 338:**
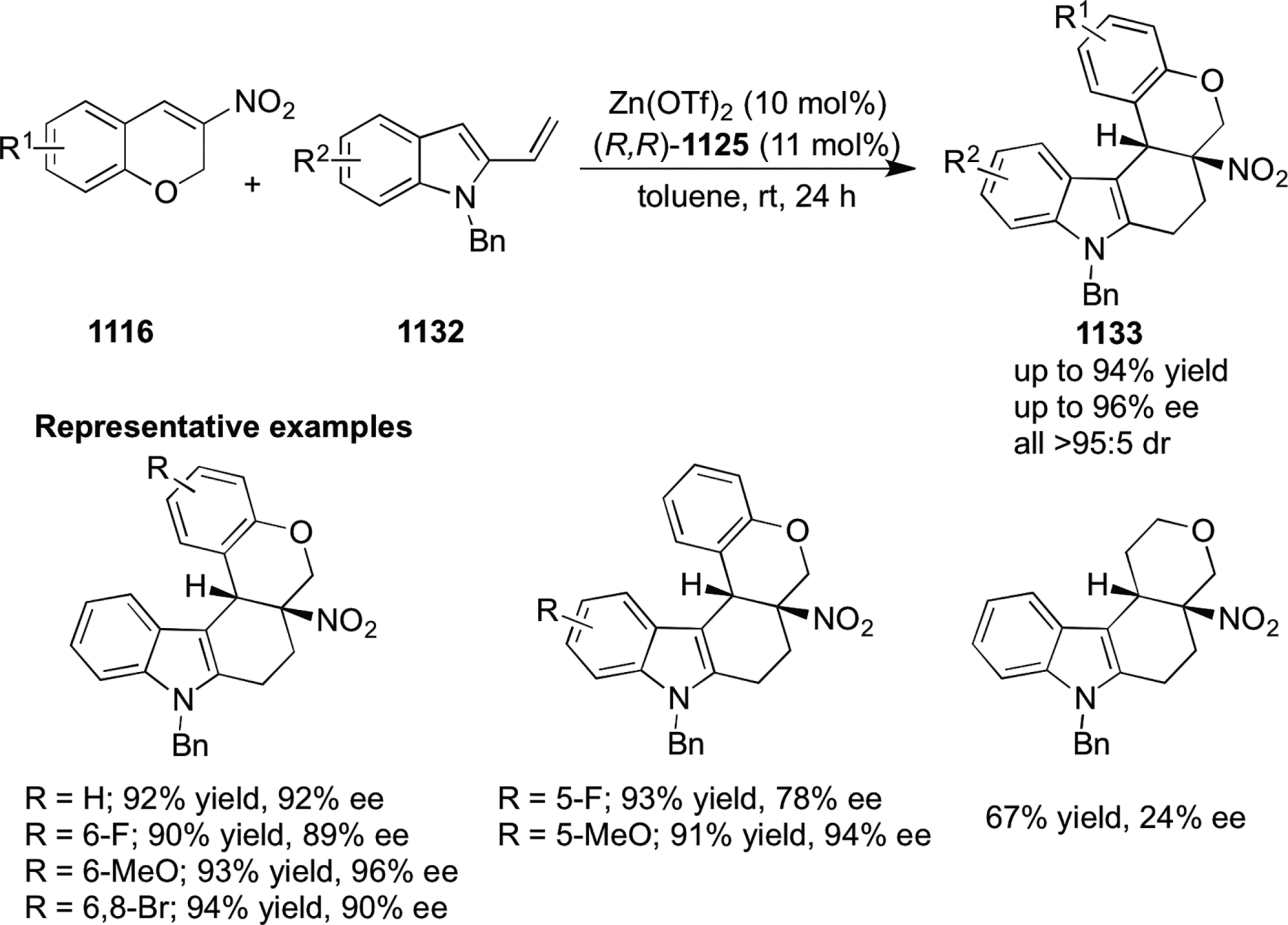
Asymmetric Zn-Catalyzed [4 + 2] Cycloaddition of 2-Vinyl Indoles with 3-Nitro-2*H*-chromenes

Ding and Xiao have described an asymmetric Zn-catalyzed [4 + 2] cycloaddition of 2-vinyl indoles **1132** with 3-nitro-2*H*-chromenes, utilizing **1125** as the chiral ligand. The tetracyclic products **1133**, containing two contiguous stereocenters were isolated in up to 94% yield, with up to 96% *ee* and >99:5 dr (Scheme 338).^400^

**Scheme 339:**
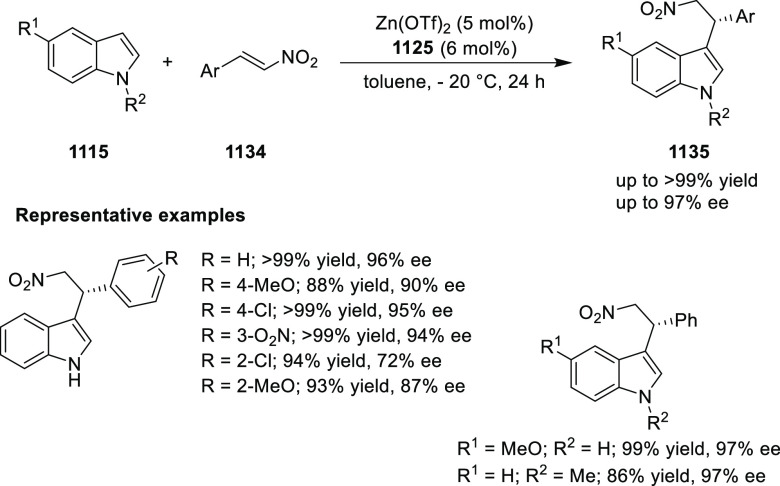
Zn-Catalyzed Asymmetric Friedel–Crafts Alkylation of Indoles with *trans*-β-Nitrostyrenes

Ligand **1125** has been applied in the Zn(OTf)_2_-catalyzed asymmetric Friedel–Crafts alkylation of indoles **1115** with *trans*-β-nitrostyrene derivatives **1134**, for the synthesis of enantioenriched nitroalkanes **1135** in high yields up to >99% and with excellent enantioselectivities up to 97% *ee* (Scheme 339). Substituients on the *trans*-β-nitrostyrene derivatives **1134** were well tolerated in terms of steric and electronic changes, except *ortho*-substitution, for example, in the reaction of indole with 2-chloro-*trans*-β-nitrostyrene, the product was isolated with only 72% *ee*, compared to 95% *ee* for the reaction with 4-chloro-*trans*-β-nitrostyrene. In the same report, ligand **1106a** was applied to the asymmetric Friedel–Crafts alkylation of pyrrole with *trans*-β-nitrostyrene derivatives, but the products were isolated with low levels of enantioselectivity of 11–91% *ee*.^401^

**Scheme 340:**
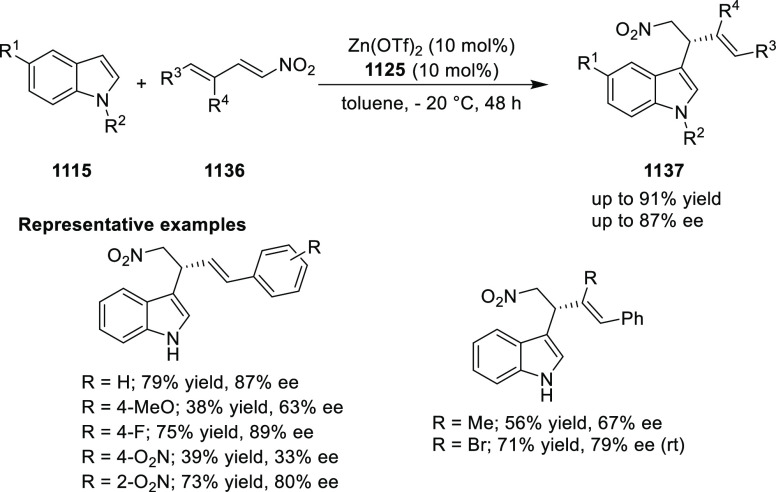
Zn-Catalyzed Asymmetric Friedel–Crafts Alkylation of Indoles with Nitrodienes

Du showed that the scope of the reaction of indoles with *trans*-β-nitrostyrene derivatives, utilizing ligand **1125**, could be extended to include 2-propargyloxy-β-nitrostyrenes, giving the products with more inconsistent levels of enantioselectivity of 36–93% *ee*. In the same report, Du explored the Zn(**1125**)-catalyzed asymmetric Friedel–Crafts alkylation of indoles **1115** with nitrodienes **1136**, giving the enantioenriched products **1137** in up to 91% yield and with lower levels of enantioselectivity compared to *trans*-β-nitrostyrenes **1134**, of up to 87% *ee* (Scheme 340).^402^

**Scheme 341:**
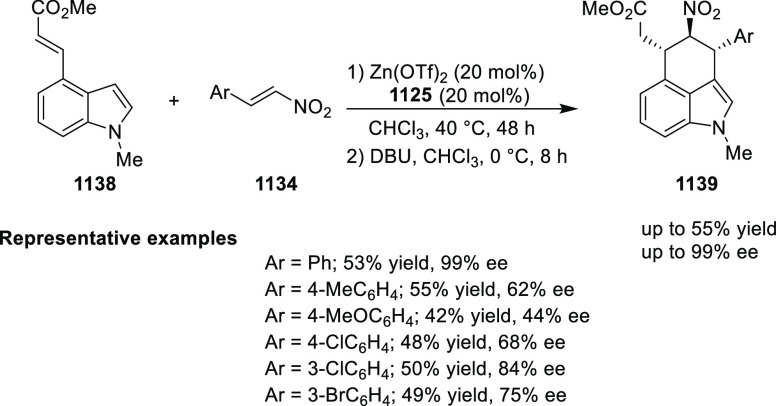
One-Pot/Two-Step Asymmetric Zn-Catalyzed Friedel–Crafts Alkylation/Michael Addition of 4-(Methylacrylato)indole with *trans*-β-Nitrostyrenes

Guiry has described a one-pot/two-step Zn/**1125**-catalyzed Friedel–Crafts alkylation/Michael addition sequence of 4-(methylacrylato)indole **1138** with *trans*-β-nitrostyrene derivatives **1134** for the enantioselective synthesis of tricyclic indoles **1139**, representing the *C*_4_-substituted core structure of the ergoline derivatives. Subjecting the indole **1138** to the Zn(OTf)_2_/**1125**-catalyzed reaction with a range of *trans*-β-nitrostyrene derivatives **1134**, followed by the addition of DBU, the tricyclic products **1139** were isolated as the *anti*–*anti* diastereomers in up to 55% yield and with up to 99% *ee* (Scheme 341). The reaction was very sensitive to changes on the aryl group of the *trans*-β-nitrostyrene derivative **1134**.^403^

**Scheme 342:**
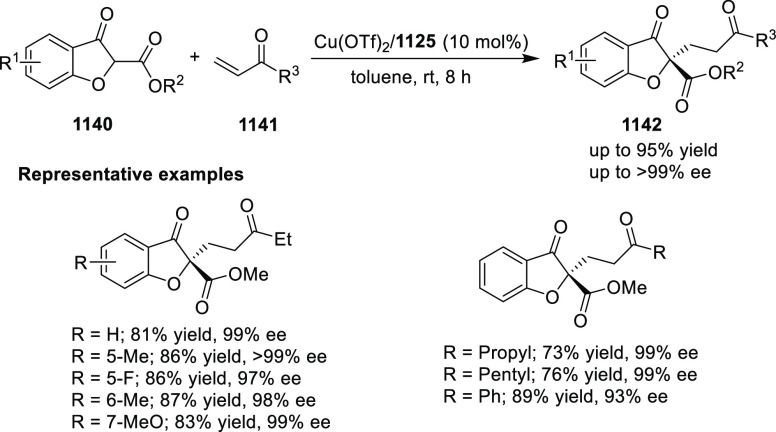
Asymmetric Cu-Catalyzed Conjugate Addition of 2-Substituted Benzofuran-3-2*H*-onesto α,β-Unsaturated Ketones

Wang and Xu have applied ligand **1125** in the Cu-catalyzed conjugate addition of 2-substituted benzofuran-3–2*H*-ones **1140** to α,β-unsaturated ketones **1141** to give the corresponding enantioenriched β-substituted-β-keto esters **1142** in up to 95% yield and with high enantioselectivities up to >99% *ee* (Scheme 342).^404^

**Figure 53:**
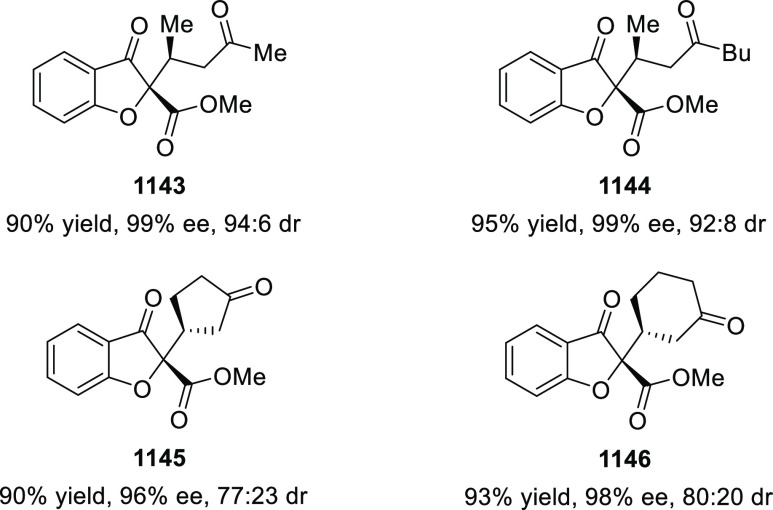
Products of the asymmetric Cu-catalyzed conjugate addition of 2-substituted benzofuran-3–2*H*-onesto β-substituted enones and cyclic enones.

In the same report, the substrate scope was extended to include linear β-substituted and cyclic enones, giving the linear products **1143** and **1144** with excellent enantio- and diastereoselectivities, while the cyclic products **1145** and **1146** were isolated in excellent enantioselectivities but low diastereoselectivities (Figure 53).

Diphenylamine-linked bis(oxazoline) ligands have been successfully applied in a range of Zn-, Cu- and Fe-catalyzed processes. The future for these ligands probably lies in new asymmetric transformations catalyzed by these metals. In particular, new applications of these ligands in Fe-catalysis should be explored.

#### 3.2.8 *C*_1_-Symmetric Bis(oxazoline) Ligands with Diphenylamine Linkers

Most commonly, bis(oxazoline) ligands are designed to incorporate *C*_2_-symmetry. This leads to simpler synthetic routes (utilizing a single amino alcohol in one cyclization step) and decreases the amount of transition states possible in the asymmetric transformation, thus increasing the enantioselectivity of the process. However, *C*_1_-symmetric bis(oxazoline) ligands have been applied to asymmetric catalytic transformations with excellent results.^405^

**Figure 54:**
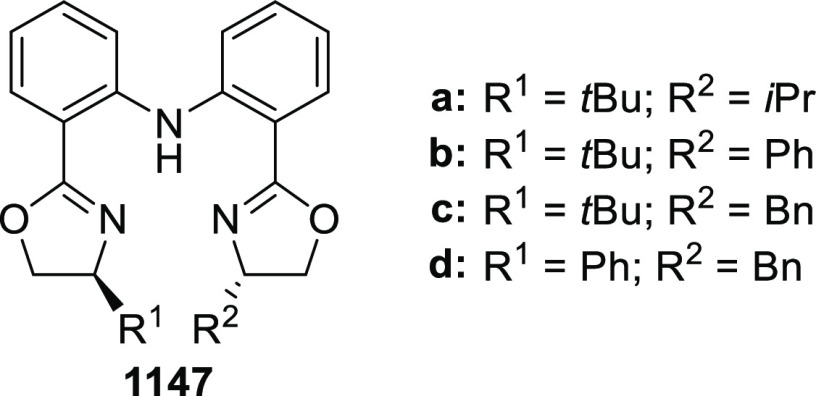
*C*_1_-symmetric diphenylamine-linked bis(oxazoline) ligands **1147**.

Guiry introduced diphenylamine-linked bis(oxazoline) ligands of the type **1106** by developing a modular synthesis, in which the two oxazoline moieties are linked by a Pd-catalyzed aryl amination. This allowed for the synthesis of both *C*_1_-(**1147)** and *C*_2_-symmetric ligands which have been applied in asymmetric catalysis (Figure 54).

Guiry has reported that *C*_1_-symmetric ligands of the type **1147a**–**d**, where R^1^ ≠ R^2^, are excellent at controlling the regio- and enantioselectivity in the enantioselective Cr-catalyzed homoallenylation of aldehydes, NHK reaction. In the reaction of allene **1149** with benzaldehyde **1148**, in the presence of CrCl_3_, Mn, TMSCl and the appropriate chiral ligand, *C*_1_-symmetric ligand **1147a** was found to give the allenyl alcohol **1150**, following acid hydrolysis, with perfect regioselectivity (over the diene **1151**) and excellent enantioselectivity of 96% *ee* (Table 8, entry 3). The *C*_2_-symmetric ligand **1106c** gave the allenyl alcohol **1150** with much lower regioselectivity of 71:29 and almost no enantioselectivity at all (entry 1). The *C*_2_-symmetric ligand **1106d** gave the allenyl alcohol **1150** with perfect regioselectivity, high enantioselectivity of 84% *ee*, but with low conversion (entry 2). The methodology was applied to a range of aryl, alkenyl and aliphatic aldehydes, generating allenyl alcohols in moderate to good yields up to 71%, with perfect regioselectivity in all but one case (naphthaldehyde) and with high enantioselectivities of up to 98% *ee*. This was the first example of a regio- and enantioselective homoallenylation of aldehydes.^406^

**Table 8:**
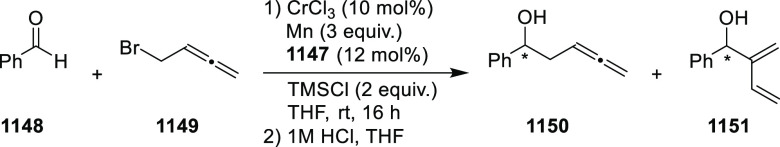
Enantioselective Cr-Catalyzed Homoallenylation of Benzaldehyde

entry	ligand	conv (%)	yield (%)	**1150**/**1151**	% *ee*
1	**1106c**	35	16	100:0	84 (*R*)
2	**1106d**	80	39	71:29	8 (*S*)
3	**1147a**	87	71	100:0	96 (*R*)
4	**1147b**	85	40	84:16	49 (*R*)
5	**1147c**	45	23	100:0	91 (*R*)
6	**1147d**	93	33	74:26	11 (*R*)

**Figure 55:**
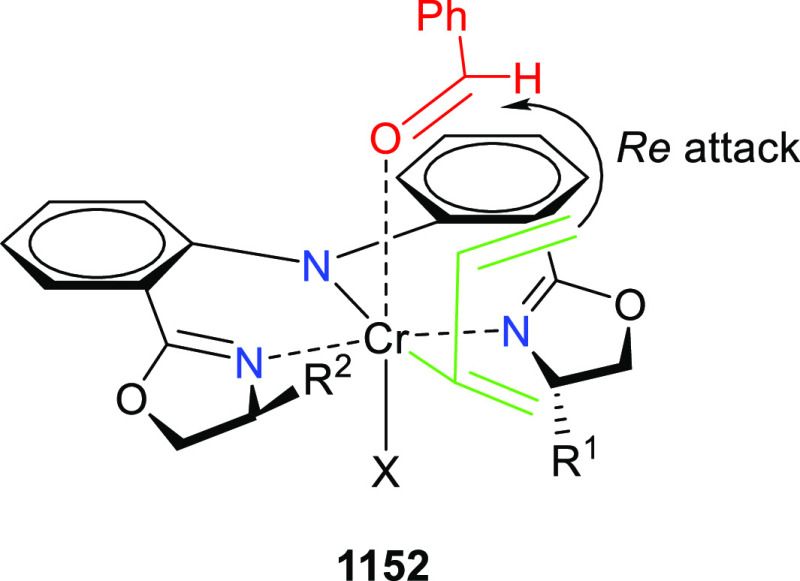
Stereochemical model for the enantioselective Cr-catalyzed homoallenylation of benzaldehyde utilizing ligand **1147a**.

A possible transition state, to explain the stereochemical outcome for this transformation with ligand **1147a**, is depicted in **1152** (Figure 55). The diene sits in the equatorial position, while the aldehyde coordinates in the apical position in an *anti*-geometry to avoid a steric interaction between the oxazoline *i*Pr-group (R^2^). This leads to *Re*-face attack to yield the (*R*)-enantiomer. The oxazoline *t*Bu-group (R^1^) might prevent the aldehyde to coordinate from the opposite apical position.

**Figure 56:**
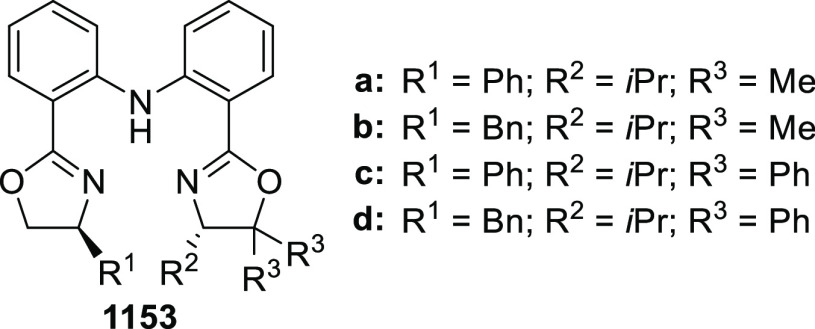
*C*_1_-symmetric *gem*-dimethyl diphenylamine-linked bis(oxazoline) ligands **1153**.

Guiry later explored the *gem*-disubstitution effect in the application of *C*_1_-symmetric ligands of the type **1153** (Figure 56) in the asymmetric Friedel–Crafts alkylation of indole **1154** and 2-methoxyfuran **1157** with *trans*-β-nitrostyrene **1155**. In a number of transformations, one of the oxazoline moieties must be *t*Bu-substituted to achieve high levels of enantioselectivity. However, l-*tert*-leucine, the parent amino alcohol of these oxazolines, is very expensive, with d-*tert*-leucine being prohibitively expensive. They exploited the *gem*-disubstitution effect with *C*_5_-*i*Pr-substituted oxazoline-containing ligands to give the products of the Friedel–Crafts alkylations with enantioselectivities better than the normal *i*Pr-substituted ligands and approaching that of the normal *t*Bu-substituted ligands.

**Scheme 343:**
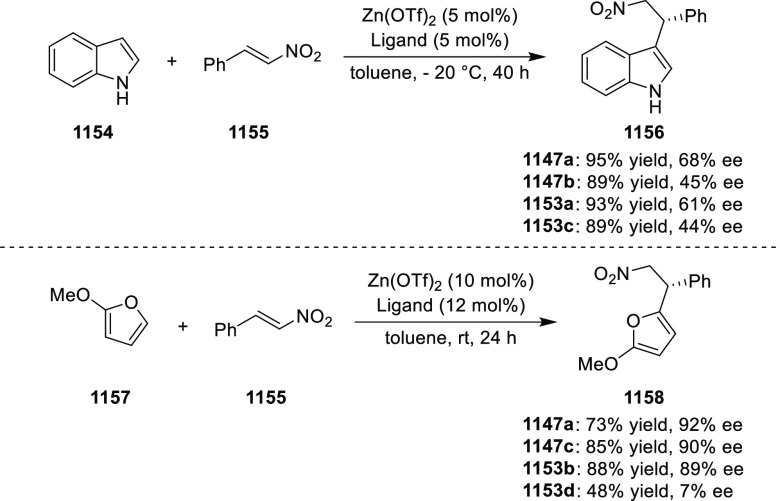
Asymmetric Friedel–Crafts Alkylation of Indole and 2-Methoxyfuran with *trans*-β-Nitrostyrene

In the Friedel–Crafts reaction of indole **1154**, the enantioenriched nitroalkane **1156** was isolated in 95% yield and 68% *ee*, when the reaction was conducted with *C*_2_-symmetric-*t*Bu-ligand **1106d** (Scheme 343). *gem*-Dimethyl-*i*Pr/Ph-ligand **1153a** gave the product **1156** in 93% yield and 61% *ee*, which is a slightly lower enantiomeric excess than **1106d**, but actually higher than *C*_1_-symmetric-*t*Bu/Ph-ligand **1147b**, which gave the product in 89% yield and 45% *ee*. In the Friedel–Crafts reaction of 2-methoxyfuran **1157**, the reaction with *C*_2_-symmetric-*t*Bu-ligand **1106d** gave the product **1158** in 73% yield and 92% *ee*, while *C*_1_-symmetric-*t*Bu/Bn-ligand **1147c** gave the product **1158** in 85% yield and 90% *ee*. The reaction with *gem*-dimethyl-*i*Pr/Bn-ligand **1153b** gave comparable results, giving product **1158** in 88% yield and 89% *ee*. In the reactions of both substrates, the *gem*-diphenyl ligands gave poor levels of enantioselectivity compared to the *gem*-dimethyl ligands.^407^

**Figure 57:**
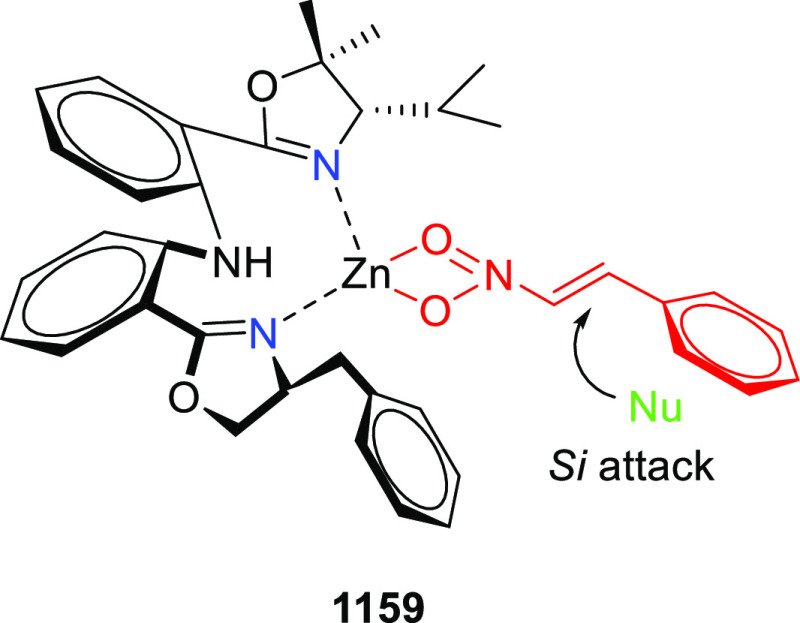
Stereochemical model for the two Friedel–Crafts alkylation reactions utilizing ligand **1153b**.

A transition state **1159**, to explain the stereochemical outcome of the two Friedel–Crafts alkylation reactions, was proposed (Figure 57). The bis(oxazoline) ligand coordinates to the Zn-center in a bidentate fashion (based on X-ray crystallographic structures for Zn(**1153**)Cl_2_ complexes), with the nitro-group coordinated to Zn in a bidentate manner through the oxygen atoms. Because of the influence of the oxazoline substituents, the appropriate nucleophile (Nu) preferentially attacks the *Si*-face of the nitrostyrene, to yield the (*R*)-enantiomer of the product **1156** in the case of the indole nucleophile and the (*S*)-enantiomer of the product **1158** in the case of the 2-methoxyfuran nucleophile.

**Figure 58:**
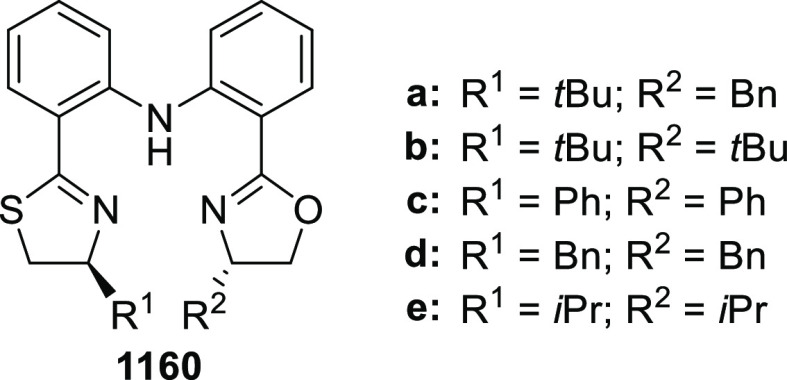
*C*_1_-symmetric thiazoline-oxazoline hybrid ligands **1160**.

Guiry has also developed *C*_1_-symmetric thiazoline-oxazoline hybrid ligands of the type **1160** (Figure 58). While these ligands are not bis(oxazoline) ligands, they are more comparable to bis(oxazoline) ligands than mono(oxazoline) ligands; therefore, they have been included in this section of the Review.

**Scheme 344:**
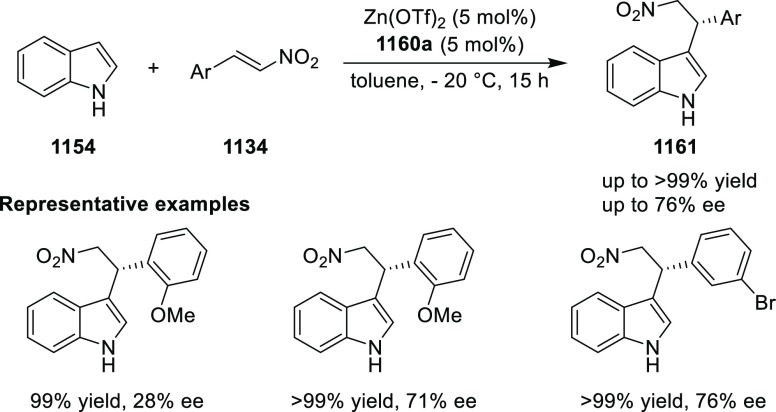
Zn-Catalyzed Asymmetric Friedel–Crafts Reaction of Indole with *trans*-β-Nitrostyrene Derivatives

The thiazoline-oxazoline ligands **1160** were successfully applied in the Zn-catalyzed asymmetric Friedel–Crafts reaction of indole with *trans*-β-nitrostyrene derivatives, with the reaction using *t*Bu/Bn-ligand **1160a** as the chiral ligand, yielding the corresponding enantioenriched nitroalkanes **1161** in up to >99% and with moderate levels of enantioselectivity up to 76% *ee* (Scheme 344).^408^

Thazoline-oxazoline ligands **1160** were also applied by Guiry in the Cr-catalyzed asymmetric Nozaki-Hiyama-Kishi (NHK) reaction of allyl bromide **1162** with benzaldehyde **1148**. A range of these ligands were tested in this reaction, a selection of which are shown in Table 9, which affords, following acidic hydrolysis, the corresponding alcohol **1163**. The nature of the substituents on the thiazoline (R^1^) and oxazoline (R^2^) moieties of ligands **1160** has a dramatic effect over the enantioselectivity of the reaction, with some ligands giving the (*R*)-product **1162** (entries 1–2) and some giving the (*S*)-product **1163** (entries 3–5). As in the Friedel–Crafts reaction, the reaction with *t*Bu/Bn-ligand **1160a** gave the best result, yielding alcohol **1163** in 84% yield and with 85% *ee* (*R*) (entry 1).^409^

**Table 9:**
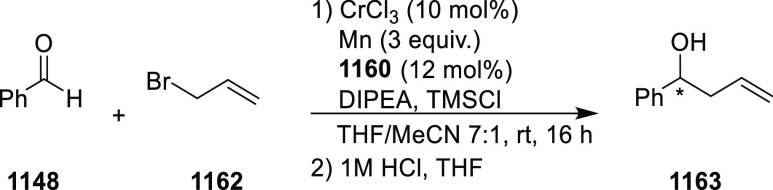
Asymmetric NHK Reaction of Allyl Bromide with Benzaldehyde

entry	ligand	conv (%)	yield (%) **1163**	% *ee*
1	**1160a**	100	84	85 (*R*)
2	**1160b**	100	87	10 (*R*)
3	**1160c**	100	81	31 (*S*)
4	**1160d**	85	67	6 (*S*)
5	**1160e**	94	78	39 (*S*)

**Figure 59:**
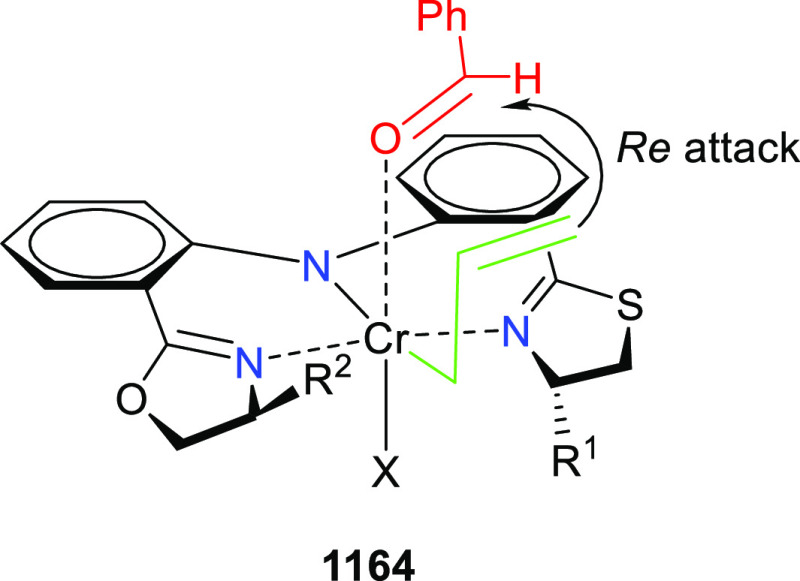
Stereochemical model for the asymmetric NHK reaction utilizing ligand **1160a**.

A possible transition state **1164**, to explain the stereochemical outcome for this transformation with ligand **1160a**, is based on the X-ray crystallographic structure obtained for a tridentate Fe(**1160**)Cl_2_ complex (Figure 59). This transition state **1164** is like the transition state **1152**, used to explain the stereochemical outcome in the Cr-catalyzed homoallenylation with ligand **1147a**. In this case, the allyl moiety binds to chromium in the equatorial position, while the aldehyde binds in the apical position in the *anti*-geometry to avoid a steric interaction between the aldehyde Ph-group and the oxazoline-benzyl (R^2^) Ph-group. The allyl moiety then attacks the aldehyde from the *Re*-face to give the (*R*)-enantiomer. As in the homoallylation transition state **1152**, the thiazoline *t*Bu group (R^1^) might block the aldehyde from coordinating from the opposite apical position.

While conventional wisdom suggests that *C*_2_-symmetry is important for applying chiral bis(oxazoline) ligands in highly enantioselective transformations, the reports described in this Review show that there is a place for *C*_1_-symmetric diphenylamine-linked bis(oxazoline) ligands. Significant progress has been made in the Cr-catalyzed asymmetric NHK reaction and in particular, the asymmetric and regioselective homoallenylation of aldehydes. This chemistry requires the use of chemical reductants, and a potential future direction could be the application of electrochemistry to develop more environmentally sustainable processes.

#### 3.2.9 *C*_1_-Symmetric Bis(oxazoline) Ligands with Modified Diphenylamine Linkers

**Figure 60:**
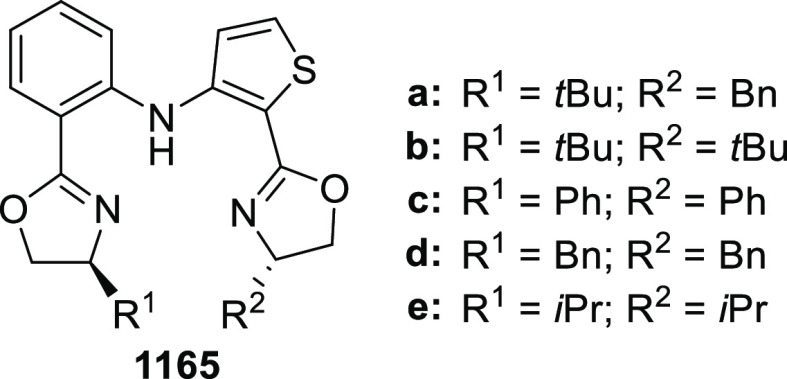
*C*_1_-Symmetric phenyl-thiophene-amine-linked bis(oxazoline) ligands **1165**.

Guiry has also developed related *C*_1_-symmetric bis(oxazoline) ligands **1165** based on a phenyl-thiophene-amine backbone (Figure 60).

These ligands **1165** were applied in the same Cr-catalyzed NHK reaction of allyl bromide **1162** with benzaldehyde **1148** to yield the enantioenriched alcohol **1163**. As with the previous report with *C*_1_-symmetric ligands **1160** (Table 9), the reaction with the *t*Bu/Bn-substituted ligand (**1165a**) gave the best result, yielding the alcohol **1163** in 83% and with 73% *ee* (*R*) (Table 10, entry 1). As before, the same trend in the formation of the (*R*)- or (*S*)-enantiomer of the product **1163** was observed for the different substituents on the oxazoline moieties.^410^

**Table 10:**
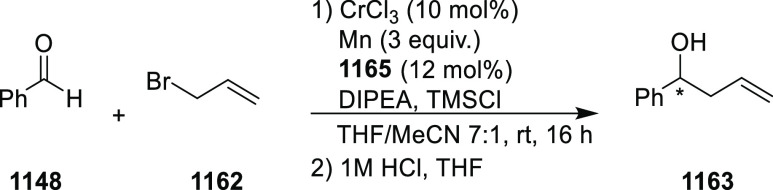
Asymmetric NHK Reaction of Allyl Bromide with Benzaldehyde

entry	ligand	conv (%)	yield (%) **1163**	% *ee*
1	**1165a**	88	83	73 (*R*)
2	**1165b**	83	79	22 (*R*)
3	**1165c**	80	72	38 (*S*)
4	**1165d**	80	78	8 (*S*)
5	**1165e**	82	78	63 (*S*)

#### 3.2.10 Bis(oxazoline) Ligands with Carbazole Linkers

**Figure 61:**
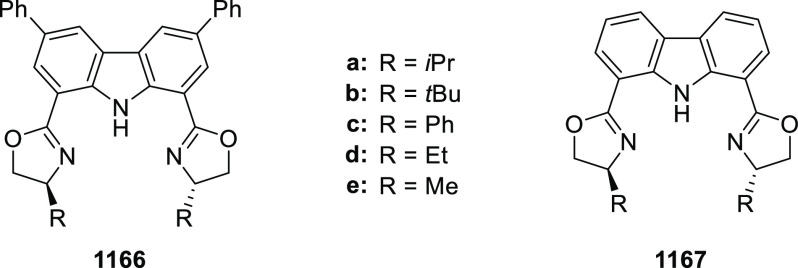
Carbazole-linked bis(oxazoline) ligands.

Developed by Nakada, carbazole-linked bis(oxazoline) ligands **1166** and **1167** are tridentate ligands with a more rigid backbone than their diphenylamine-linked counterparts (Figure 61).^411^ They have mostly been applied to Cr-catalyzed NHK-type transformations, with some exceptions.

**Scheme 345:**
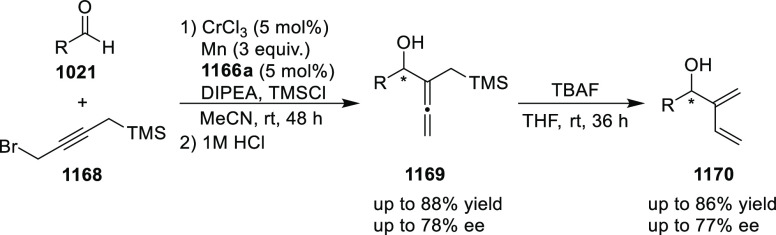
Enantioselective Cr-Catalyzed Addition of (4-Bromobut-2-ynyl)-trimethylsilane to Aldehydes

Connell has reported the enantioselective Cr-catalyzed addition of (4-bromobut-2-ynyl)-trimethylsilane **1168** to aldehydes **1021** for the asymmetric synthesis of (1,3-butadien-2-yl)methanols **1170** via [1-(silylmethyl)allenyl]methanols **1169**. Using *i*Pr-ligand **1166a** as the chiral ligand, a small range of [1-(silylmethyl)allenyl]methanols **1169** were accessed in up to 88% yield and with up to 78% *ee* (Scheme 345). These were converted to (1,3-butadien-2-yl)methanols **1170** by treating with TBAF in up to 86% yield and with up to 77% *ee*, with a slight decrease in the enantiomeric excess (for R = Ph, 78% *ee* to 70% *ee*).^412^

**Scheme 346:**
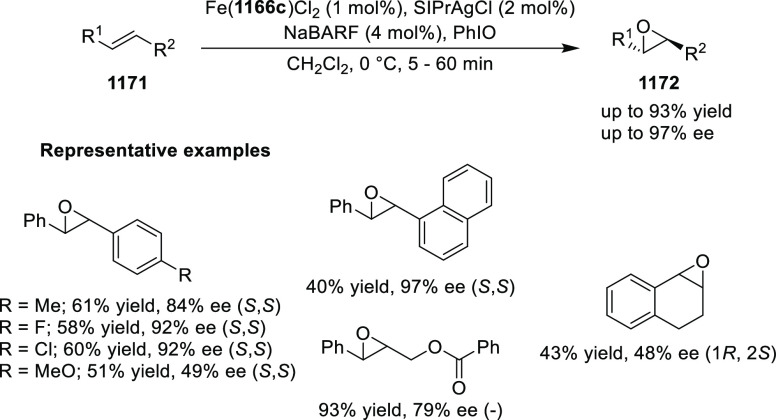
Asymmetric Fe-Catalyzed Epoxidation of *trans*-Alkenes

Nakada has reported that the Fe(III) complex of Ph-ligand **1166c** Fe(**1166c**)Cl_2_ catalyzes the asymmetric epoxidation of *trans*-alkenes **1171** in up to 93% yield and with up to 97% *ee* (Scheme 346). *Trans*-stilbene derivatives and cinnamyl alcohol derivatives performed well in the reaction, although substrates bearing an electron-rich arene gave the epoxides with only moderate levels of enantioselectivity. The only cyclic alkene tested (1,2-dihydronaphthalene) gave the epoxide product with only 48% *ee*. The diphenylamine-linked ligand **1106a** did not catalyze the epoxidation, indicating that the π–π conjugation system of the carbazole ligand **1166c** is essential to the catalytic activity of the Fe(III) complex in this transformation.^413^

**Scheme 347:**
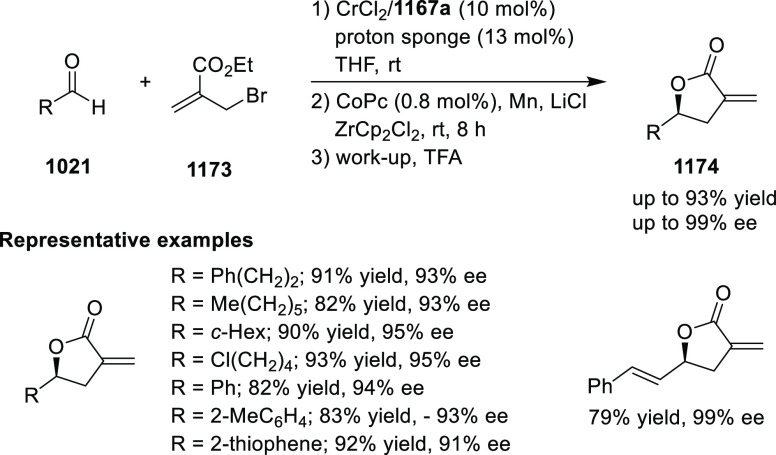
Cr-Catalyzed Enantioselective Synthesis of α-*exo*-γ-Butyrolactones

Zhang has reported the use of ligands **1167** in a variety of Cr-catalyzed processes, like the enantioselective synthesis of α-*exo*-γ-butyrolactones **1174** via a sequential 2-(alkoxycarbonyl)allylation/lactonization process. Ethyl 2-(bromomethyl) acrylate **1173** was reacted with a variety of aldehydes **1021** in the presence of 10 mol % CrCl_2_ and *i*Pr-ligand **1167a**, followed by acidic workup or treatment with K_2_CO_3_ overnight to give the enantioenriched lactones **1174** in up to 93% yield and with up to 99% *ee* (Scheme 347). The diphenylamine-linked bis(oxazoline) ligand **1106c** also catalyzed the model reaction but with a lower enantiomeric excess (79% *ee* compared to 93% *ee*).^414^

**Figure 62:**
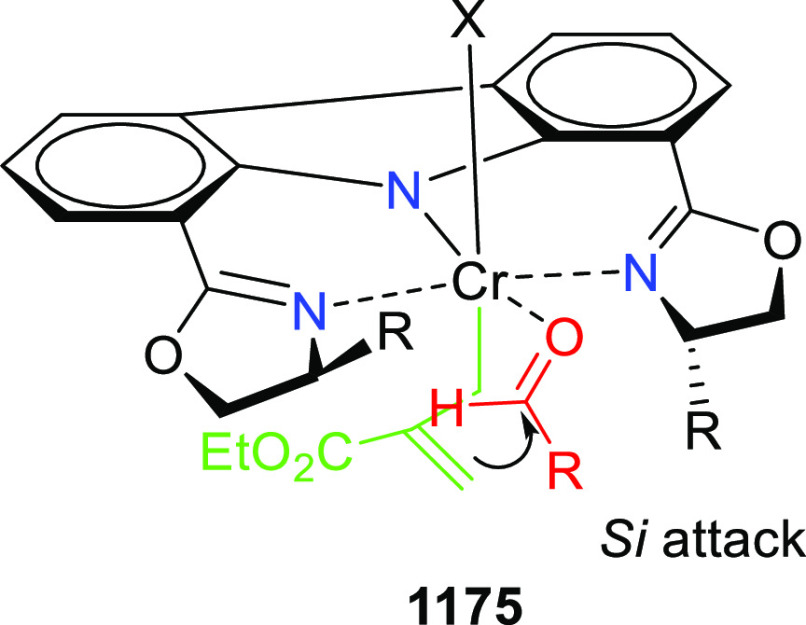
Stereochemical model for the sequential 2-(alkoxycarbonyl)allylation/lactonization process utilizing ligand **1167a**.

A possible transition state **1175**, similar to the other Cr-catalyzed processes utilizing these ligands and diphenyl-amine-linked bis(oxazoline) ligands, but invoking tridentate chelation, was proposed to explain the formation of the (*S*)-enantiomer of the product with the *i*Pr-ligand **1167a** (Figure 62).

**Scheme 348:**
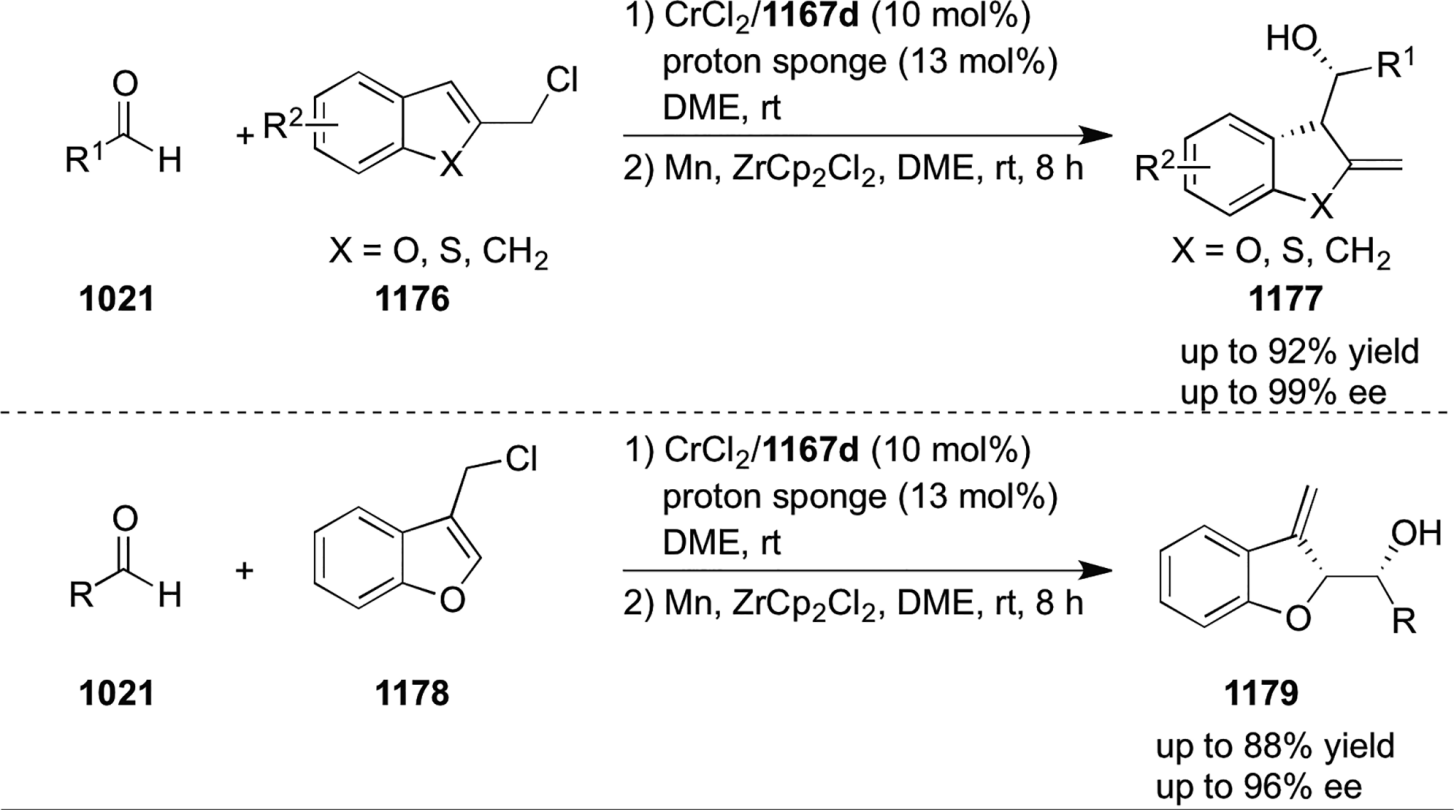
Asymmetric Cr-Catalyzed Dearomative Addition of Chloro(methyl) Heteroarenes to Aldehydes

The asymmetric Cr-catalyzed dearomative addition of halomethylarenes **1176** to aldehydes **1021** for the asymmetric synthesis of secondary benzylic alcohols **1177** was also reported by Zhang, employing Et-ligand **1167d**. 2-(Chloromethyl)benzofurans, -benzothiophenes, and -indenes **1176** were successfully subjected to the reaction conditions with a range of aryl, alkenyl and aliphatic aldehydes **1021** to give the corresponding benzylic alcohols in up to 92% yield and with enantiomeric excesses up to 99% *ee* (Scheme 348). 3-(Chloromethyl)benzofuran **1178** was also subjected to the reaction to yield the corresponding secondary benzylic alcohols **1179** in up to 88% yield and with up to 96% *ee*.^415^ The scope of this process was extended to include bromomethylnaphthalenes,^416^ bromomethyl- and chloromethyloxazoles, and bromomethyl- and chloromethylindoles^417^ with similar results.

**Scheme 349:**
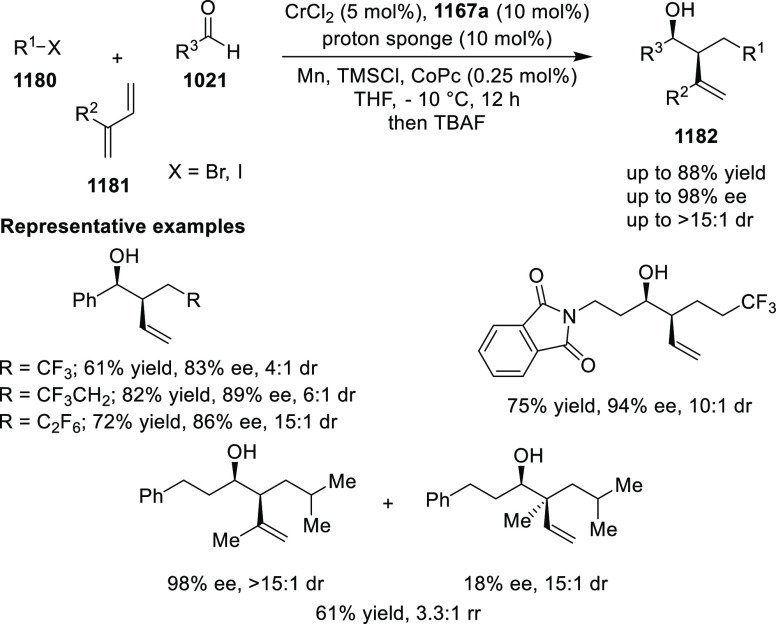
Asymmetric Cr/Co-Bimetallic Alkylation/Carbonyl Allylation

An enantioselective 1,2-difunctionalization of 1,3-butadienes **1181** via an asymmetric Cr/Co-bimetallic alkylation/carbonyl allylation sequence was achieved by Zhang, with the *i*Pr-ligand **1167a**. A range of 1,3-butadienes **1181** were difunctionalized with various alkyl bromides and iodides **1180**, and aliphatic and aryl aldehydes **1021**. The enantioenriched alcohols **1182** were isolated in good yields up to 88%, with high levels of enantioselectivity up to 98% *ee* and with high diastereoselectivities up to >15:1 dr (Scheme 349). A range of fluorinated and nonfluorinated alkyl iodides/bromides were successfully applied in this transformation.^418^

Like the diphenylamine-linked bis(oxazoline) ligands, carbazole-linked bis(oxazoline) ligands have been successfully applied in enantioselective Cr-catalyzed processes. The carbazole backbone is rigid compared to the flexible diphenylamine backbone; as a result, both ligand families have their own unique properties.

#### 3.2.11 Miscellaneous Bis(oxazoline) Ligands with Free-Amine-Based Linkers

**Scheme 350:**
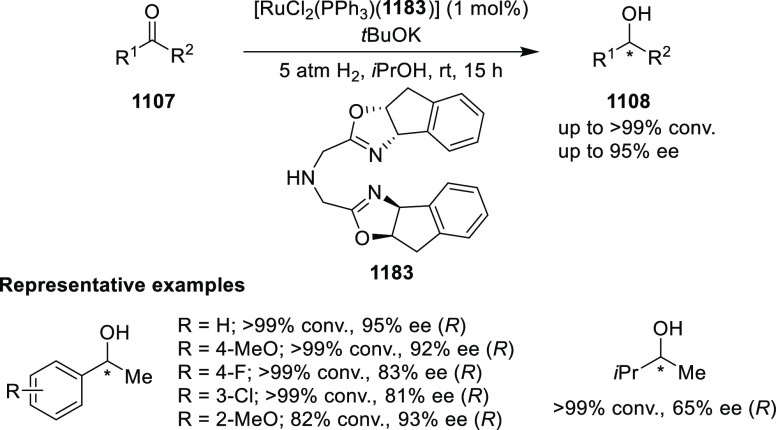
Asymmetric Ru-Catalyzed Hydrogenation of Ketones

Zhang has reported the asymmetric Ru-catalyzed hydrogenation of ketones **1107** with a tridentate, bifunctional indaBOX ligand **1183** for the synthesis of enantioenriched alcohols **1108** with up to >99% conversion and up to 95% *ee* (Scheme 350). Monoaryl and cyclohexyl ketones performed well in the reaction, yielding the secondary alcohols with enantiomeric excesses >80%, but linear aliphatic ketones gave the corresponding alcohols in moderate enantioselectivities 42–65% *ee*.^419^

**Figure 63:**
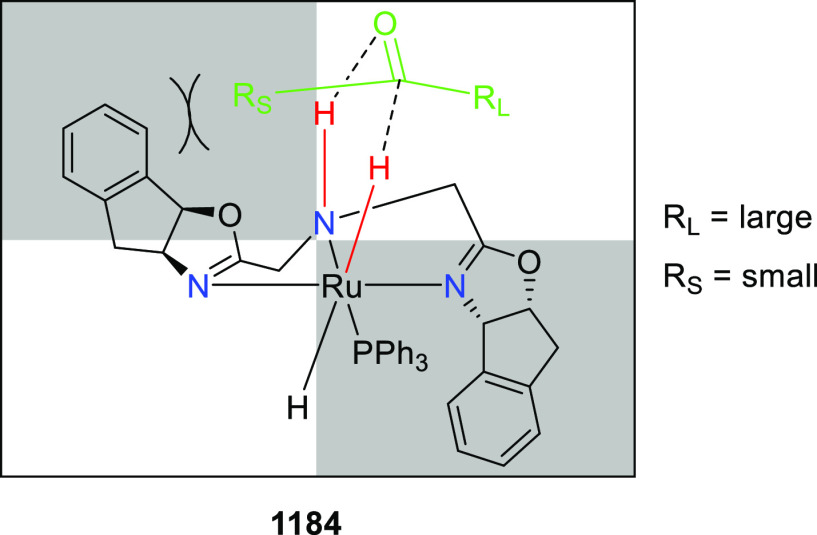
Stereochemical model for the asymmetric Ru-catalyzed hydrogenation utilizing ligand **1183**.

A possible transition state **1184** was proposed to explain the stereochemical outcome of the transformation, highlighting the suggested ligand-assisted mechanistic pathway (Figure 63).

**Figure 64:**
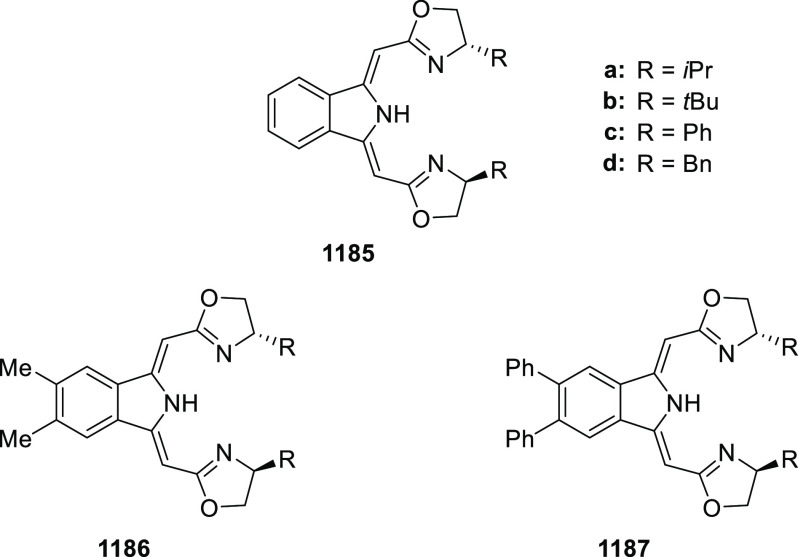
Boxmi ligands.

Gade has developed a new class of chiral, planar, rigid pincer-type ligand, bis(oxazolinylmethylidene)isoindolines **1185**– **1187** (boxmi), which contain a phthalimide-based linker in the backbone (Figure 64).

**Scheme 351:**
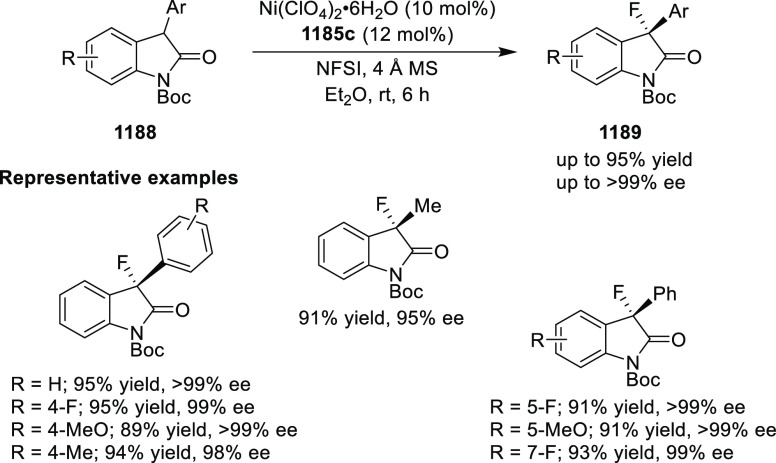
Ni-Catalyzed Asymmetric α-Fluorination of Oxindoles

Gade first applied these ligands in the Ni-catalyzed asymmetric α-fluorination of oxindoles **1188** with NFSI. A range of α-aryl-α-fluorooxindoles **1189** (and one α-methyl example) were accessed in up to 95% yield and with up to >99% *ee*, using opitimized reaction conditions with Ph-boxmi **1185c** as the chiral ligand (Scheme 351). They also extended the scope to include indanone-based β-keto esters with enantioselectivities up to 97% *ee*.^420^

In same report, Gade applied the ligands in the NHK-reaction of allyl bromide **1062** with benzaldehyde **1021** and Table 11 summarizes the results of this study. The reaction with *i*Pr-ligand **1186a** gave the best outcome, giving alcohol **1063** in 92% yield and with 86% *ee* (*S*) (entry 4). Unlike the diphenylamine- and carbazole-linked bis(oxazoline) ligands, all ligands tested in the boxmi series led to the formation of the (*S*)-alcohol.

**Table 11:**
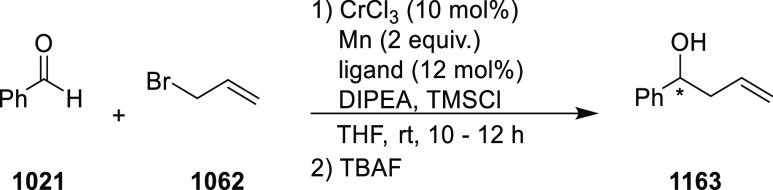
Asymmetric NHK Reaction of Allyl Bromide with Benzaldehyde

entry	ligand	time (h)	yield (%) **1063**	% *ee*
1	**1185a**	12	92	83 (*S*)
2	**1185b**	10	91	73 (*S*)
3	**1185c**	12	91	84 (*S*)
4	**1186a**	10	92	86 (*S*)
5	**1186b**	10	93	68 (*S*)
6	**1186c**	10	89	54 (*S*)
7	**1186d**	12	92	73 (*S*)
8	**1187a**	12	91	83 (*S*)
9	**1187b**	12	92	63 (*S*)
10	**1187c**	12	93	79 (*S*)

**Scheme 352:**
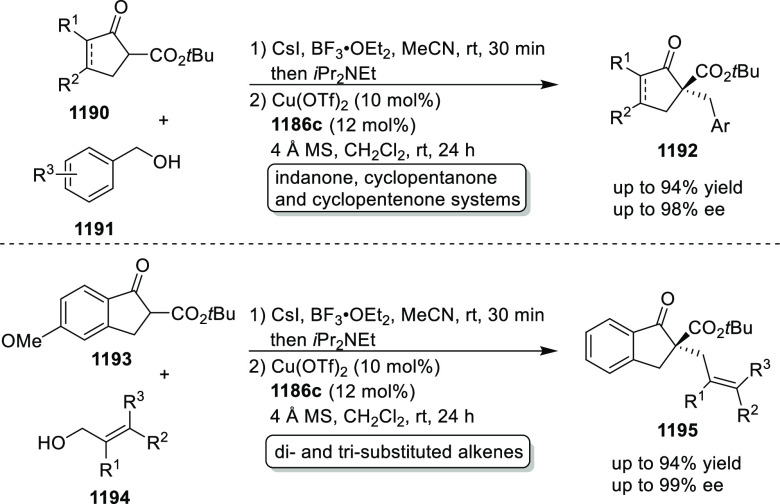
Asymmetric Cu-Catalyzed α-Alkylation/Allylation of β-Keto Esters

Gade has developed an asymmetric Cu/**1186c**-catalyzed α-alkylation of β-keto esters **1190** with benzyl alcohols **1191**, for the enantioselective synthesis of enantioenriched β-keto esters **1192** in up to 94% yield and with 98% *ee* (Scheme 352). A variety of indanone-, cyclopentanone- and cyclopentenone-based β-keto esters **1192** were tolerated in the reaction. The initial step in the reaction converts the alcohols **1191** to the corresponding iodides with CsI, and the iodides then participate in the alkylation reaction in the second step. Benzyl alcohols **1191** bearing different substitutions were well tolerated; however, no heteroaromatic substrates were reported. The reaction was then extended to include allyl alcohols **1194** as the alkylating agent. Both di- and trisubstituted alkenes were tolerated in the reaction, giving the enantioenriched β-keto esters **1195** in up to 94% yield and with up to 99% *ee*.^421^

**Scheme 353:**
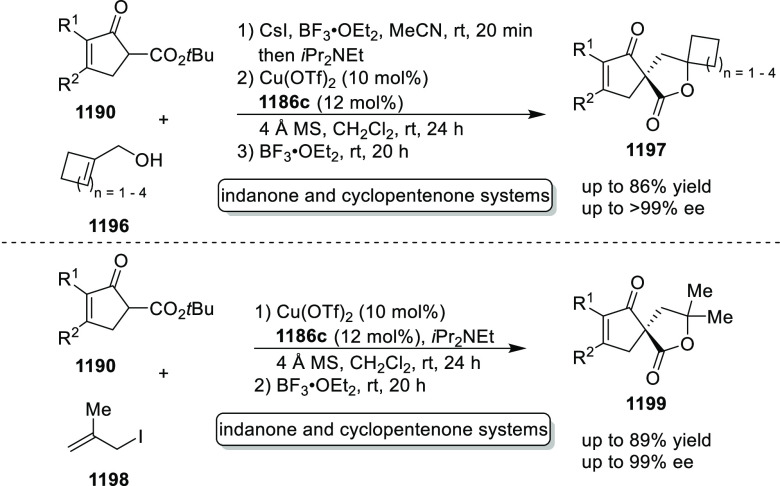
Asymmetric Synthesis of Spirolactones

In the same report, Gade reported that treating the allylated-β-keto ester products from the reaction with cycloalkenes **1196** with BF_3_·OEt_2_, the allylation could be further extended to the synthesis of bispirolactones **1197** in up to 86% yield and with >99% *ee* (Scheme 353). When 3-iodo-2-methylpropene **1198** (which cannot be prepared from the corresponding alcohol by the CsI method) was used directly in the allylation/spirolactonization sequence, spirolactones **1199** were isolated in up to 89% yield and with 99% *ee*.

**Scheme 354:**
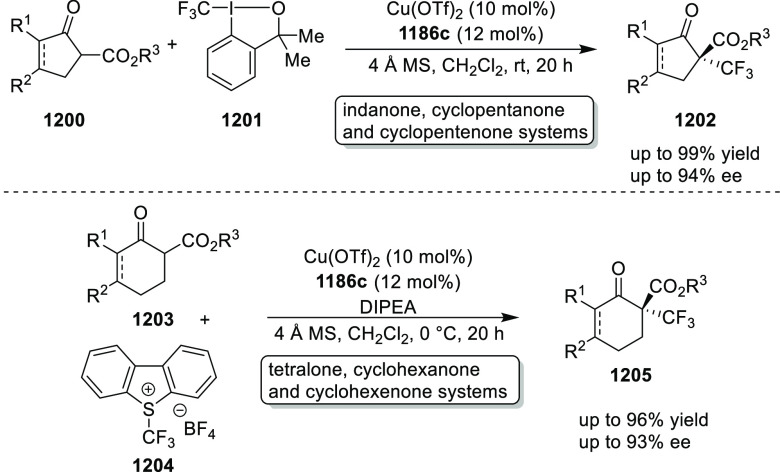
Asymmetric Cu-Catalyzed Trifluoromethylation of β-Keto Esters

Gade later reported an asymmetric Cu/**1186c**-catalyzed trifluoromethylation of β-keto esters **1200** and **1203**. The 5-membered-ring-based β-keto esters **1200** were trifluoromethylated under optimized conditions with Togni’s reagent **1201** to give the α-trifluoromethyl-β-keto esters **1202** in up to 99% yield and with up to 94% *ee* (Scheme 354). The 6-membered-ring-based β-keto esters **1203**, with a more enolizable ketone were found to undergo the trifluoromethylation with higher levels of enantioselectivity with Umemoto’s reagent **1204** and DIPEA in place of Togni’s reagent **1201**. The 6-membered-ring-based products **1205** were isolated in up to 96% yield and with up to 93% *ee*.^422^ Gade subsequently developed a very similar methodology for a highly enantioselective Cu/**1185c**-catalyzed trifluoromethylthiolation of β-keto esters with the -SCF_3_ analogue of Togni’s reagent.^423^

**Scheme 355:**
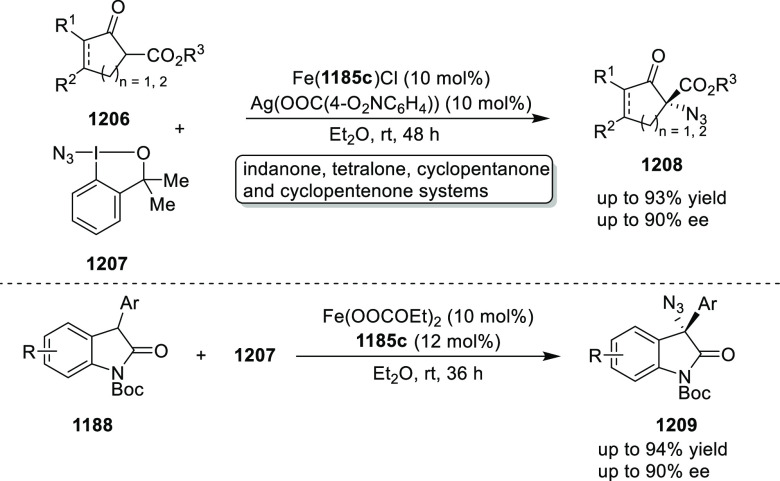
Asymmetric Fe-Catalyzed Azidation of β-Keto Esters and Oxindoles

Further expanding on their use of boxmi-ligands in the α-functionalization of oxindoles and β-keto esters, Gade described an asymmetric Fe-catalyzed azidation of β-keto esters **1206** and oxindoles **1188** with an azide-analogue of Togni’s reagent **1207** (Scheme 355). During optimization they found that iron carboxylates gave the highest levels of enantioselectivity in the reaction. A screen of silver carboxylates in combination with the [Fe(**1185c**)Cl] complex led to the best result being obtained with silver *p*-nitrobenzoate. Using the optimized conditions, a range of 5- and 6-membered-ring-based β-keto esters **1206** were azidated to give the enantioenriched products **1208** in up to 93% yield and with up to 90% *ee*. A range of α-aryl-oxindoles **1188** were azidated to give the products in up to 94% yield and with up to 90% *ee*, utilizing Fe(OOCOEt)_2_/**1185c** and no silver salts.^424^

**Scheme 356:**
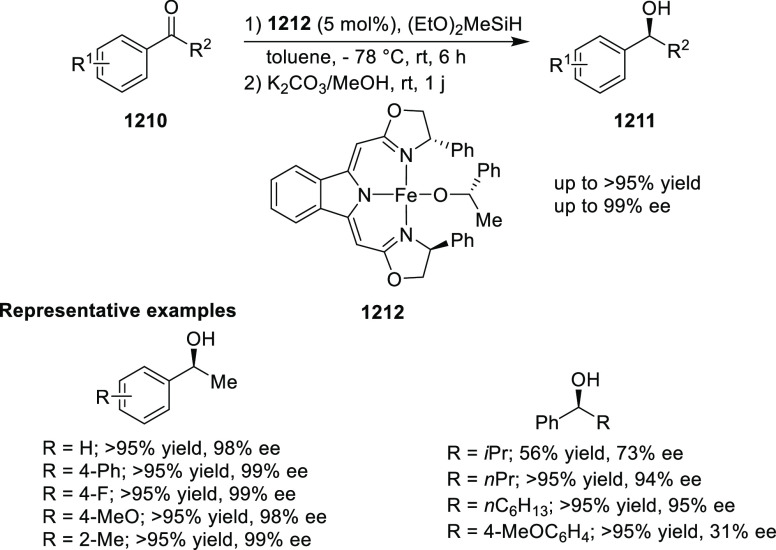
Asymmetric Fe-Catalyzed Hydrosilylation of Ketones

Gade has furthered the application of boxmi ligands in Fe-catalysis by developing a highly reactive Fe/**1185c** system for the hydrosilylation of ketones **1210**. Under optimized conditions, Fe-catalyst **1212** successfully catalyzed the hydrosilylation of a range of ketones **1210**, the first Fe-catalyst to achieve >95% *ee* in this reaction and the first Fe-catalyst to operate at such relatively low temperatures. Monoarylated alcohols **1211** were isolated in up to >95% yield and with up to 99% *ee*, whereas diaryl ketones gave lower levels of enantioselectivity due to less discrimination between the two groups (Scheme 356).^425^

Tridentate bis(oxazoline) ligands bearing free-amine linkers have been shown to be useful in a range of transformations. In particular, Gade has pioneered the use of Boxmi ligands in Fe-catalysis. These ligands can be used to form very active Fe-catalysts, and future developments in this area are expected.

#### 3.2.12 Bis(oxazoline) Ligands with Pyridine Linkers and Monosubstituted Oxazoline Rings

**Figure 65:**
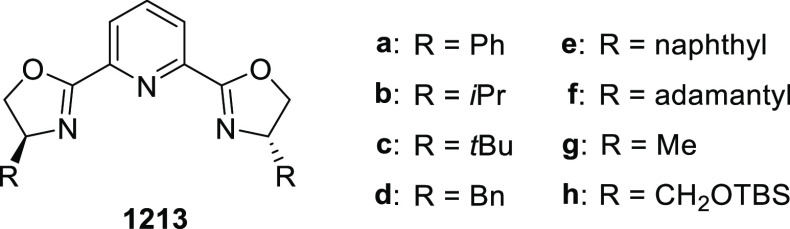
PyBOX ligands.

PyBOX ligands **1213a**–**h** are an extensively used class of pyridine-based tridentate bis(oxazoline) ligand (Figure 65).

Bolm has applied ligand **1213a** in the Fe-catalyzed enantioselective transfer of nitrenes to sulfides to afford chiral sulfimides (nitrogen analogues of sulfoxides) in excellent yields and good enantiomeric excesses of up to 90% *ee*. Only Ph-PyBOX **1213a** was found to induce good levels of stereoinduction, while the other PyBOX ligands tested **1213b**-**c** gave almost racemic products. The Fe(III) source was found to have a dramatic effect on the stereoselectivity of the reaction, with only Fe(III) acetylacetonate derivatives yielding the product in good enantioselectivity (Table 12). Ultimately, the catalyst derived from [Fe(dmhdCl)_3_] was found to be optimal giving sulfimide **1216** in 98% yield and 86% *ee* (entry 6). Only sulfides **1214** bearing at least one nonaliphatic group facilitated nitrene transfer with good stereoselectivity. For example, when the benzyl analogue of sulfide **1214** was subjected to the reaction, the product was isolated in 55% yield and 10% *ee*, the only example with an enantiomeric excess of less than 60% *ee*.^426^

**Table 12:**
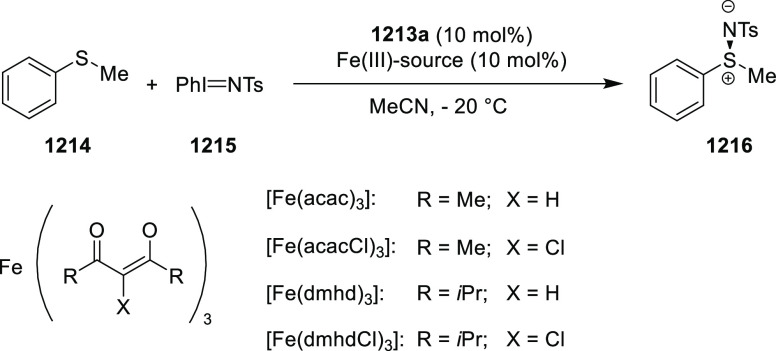
Fe(III) Source Effect on Stereoselectivity of the Reaction

entry	Fe source	ligand	time	yield	% *ee*
1	[Fe(acac)_3_]	(*S*,*S*)-**1213a**	14 h	75%	50%
2	[Fe(acacCl)_3_]	(*S*,*S*)-**1213a**	1 h	97%	64%
3	[Fe(dmhd)_3_]	(*S*,*S*)-**1213a**	6 h	99%	68%
5^a^	[Fe(dmhdCl)_3_]	(*S*,*S*)-**1213a**	2 h	99%	82%
6^a^	[Fe(dmhdCl)_3_]	(*R*,*R*)-**1213a**	16 h	98%	–86%

a Acetone instead of MeCN.

**Scheme 357:**
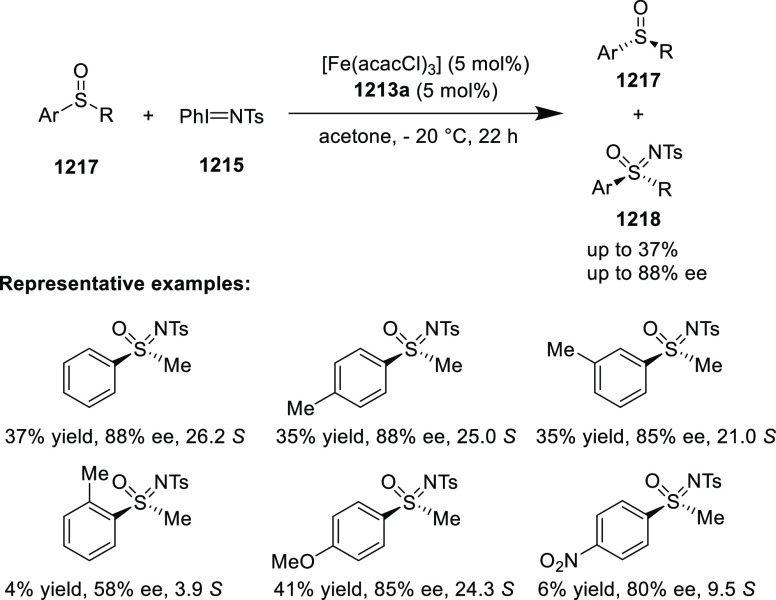
Fe(III)-Catalyzed Imidative Kinetic Resolution of Racemic Sulfoxides

Bolm applied the same ligand **1213a** in the Fe(III)-catalyzed imidative kinetic resolution of racemic sulfoxides, accessing the sulfamidates in moderate to good yields and enantioselectivities. When racemic sulfoxides **1217** are subjected to the reaction conditions, sulfamidates **1218** were obtained in up to 37% yield and 88% *ee*, with an *S* factor of up to 26.2 (Scheme 357). The reaction outcome, especially with regard to the yield of the reaction, was sensitive to steric and electronic modifications on the aryl ring of the sulfoxide **1217**. For example, the 2-methyl and 4-nitro-substituted examples gave the corresponding products in 4% and 6% yield with enantiomeric excesses of 58% and 80% *ee*, respectively.^427^

**Scheme 358:**
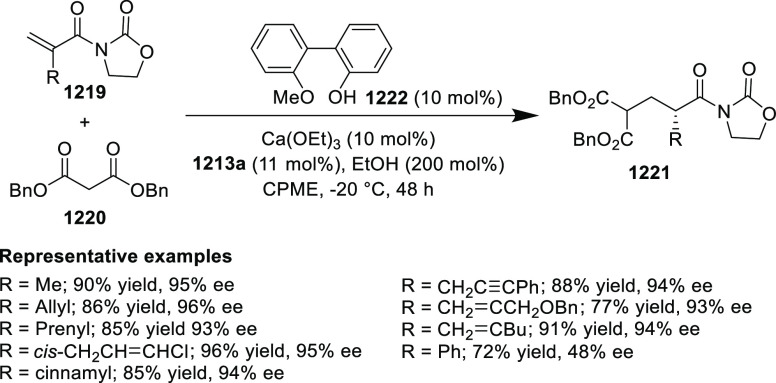
Asymmetric Protonation of Chiral Calcium Enolates

Ligand **1213a** was applied by Kobayashi in the asymmetric protonation of chiral calcium enolates formed from the 1,4-addition of malonates to oxazolidinone-based Michael acceptors. Following addition of the malonate **1220** to the Michael acceptor **1219**, the chiral Ca(II)-PyBOX complex coordinates to and rigidifies the enolate, controlling its geometry, yielding enantiomerically enriched **1221** after protonation (Scheme 358). Only Ph-PyBOX **1213a** was found to induce good stereoselectivity and interestingly, best results were obtained when 10 mol% phenol **1222** and 200 mol% EtOH were used as additives in cyclopentyl methyl ether (CPME) at −20 °C. The products **1221** were formed in excellent yields and enantioselectivities of up to 91% and 96% *ee*, respectively, with a variety of alkyl groups. In the case of an aryl group (R = Ph) a diminished enantiomeric excess of 42% was achieved.^428^

**Scheme 359:**
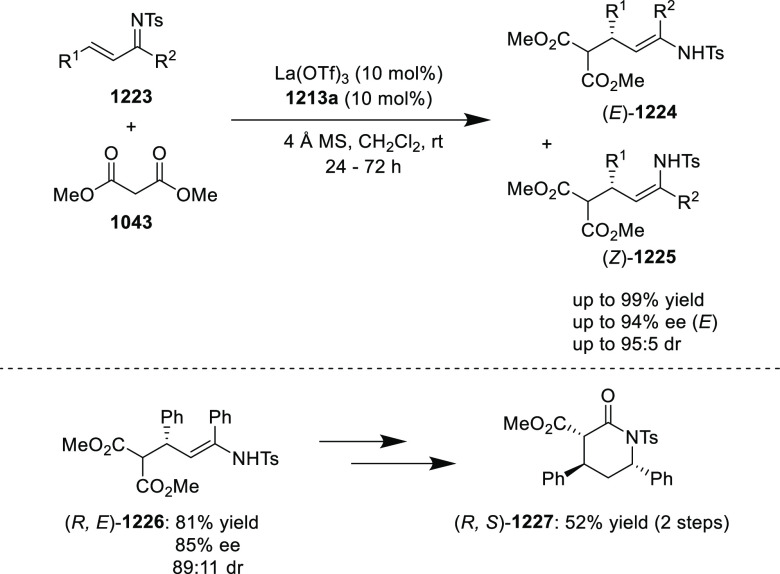
La(III)-Catalyzed Asymmetric Conjugate Addition of Malonate Esters to α,β-Unsaturated *N*-Sulfonyl Imines

Ligand **1213a** was employed by Blay and Pedro in the La(III)-catalyzed asymmetric conjugate addition of malonate esters to α,β-unsaturated *N*-sulfonyl imines **1223**. Of the ligands tested, only the Ph-PyBOX **1213a** gave appreciable levels of enantiomeric excess, with a range of (*E*)-enamine products **1224** isolated in moderate to excellent yields, moderate to high *E*/*Z* selectivities (up to 95:5 *E*/*Z*) and good enantioselectivities up to 94% *ee* (Scheme 359). The enamine products can be used to synthesize chiral δ-aminoesters and lactams with good efficiency, for example, optically pure lactam (*R*,*S*)-**1227** was accessed in 2 steps and 52% yield from (*R*,*E*)-enamine **1226**.^429^

**Scheme 360:**
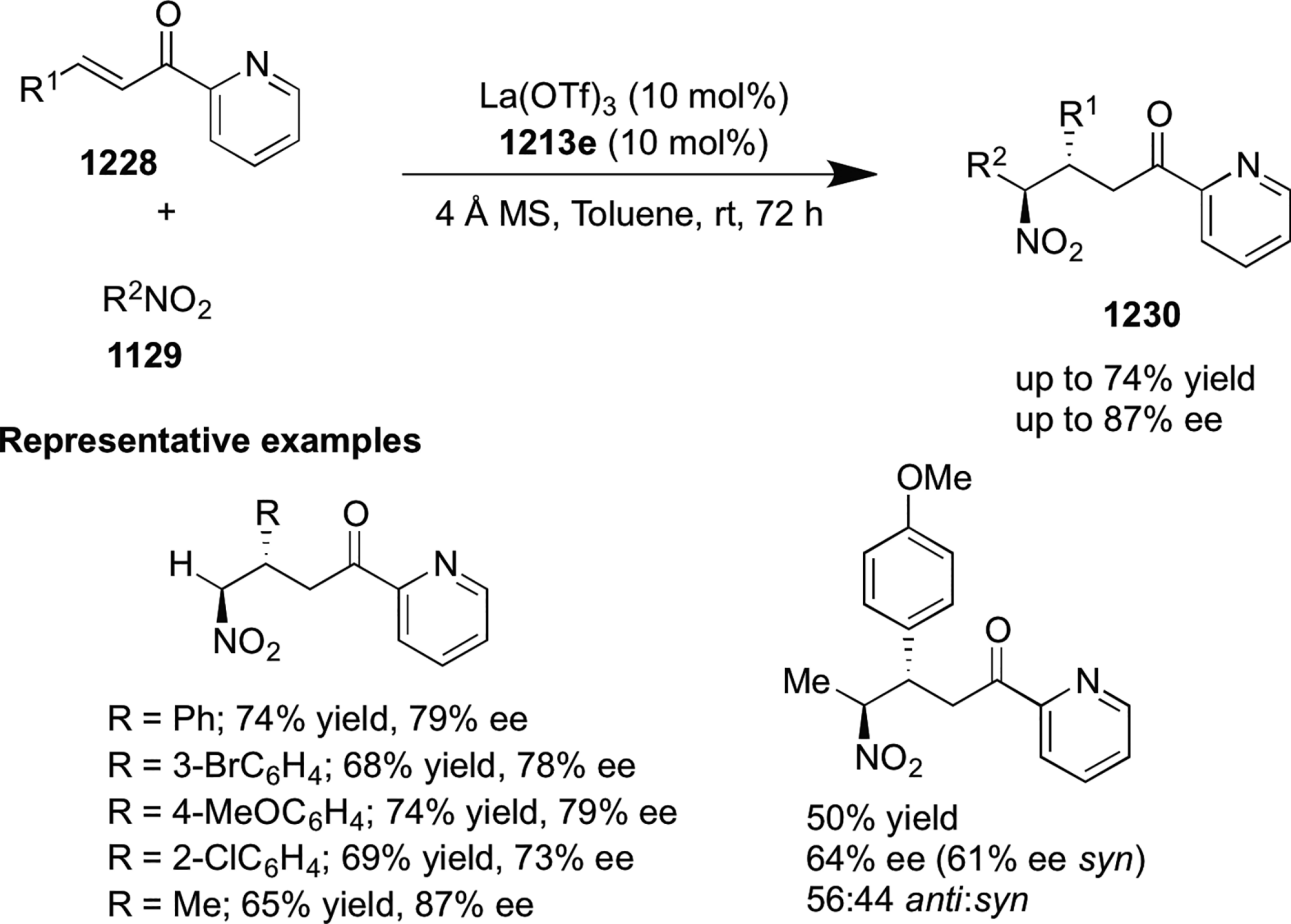
La(III)-Catalyzed Asymmetric Conjugate Addition of Nitroalkanes to (*E*)-2-Azachalcones

Pedro and Blay described a new naphthyl-PyBOX ligand **1213e** in the related La(III)-catalyzed conjugate addition of nitroalkanes to (*E*)-2-azachalcones **1228**, providing the nitro-Michael product **1230** in up to 74% yield and moderate to good enantioselectivities of up to 87% *ee* for a range of substrates (Scheme 360). The reaction proceeded well when R^1^ was either aryl or alkyl, and R^2^ was methyl. Where R^2^ = ethyl or propyl, the dr of the corresponding products was often low.^430^

**Scheme 361:**
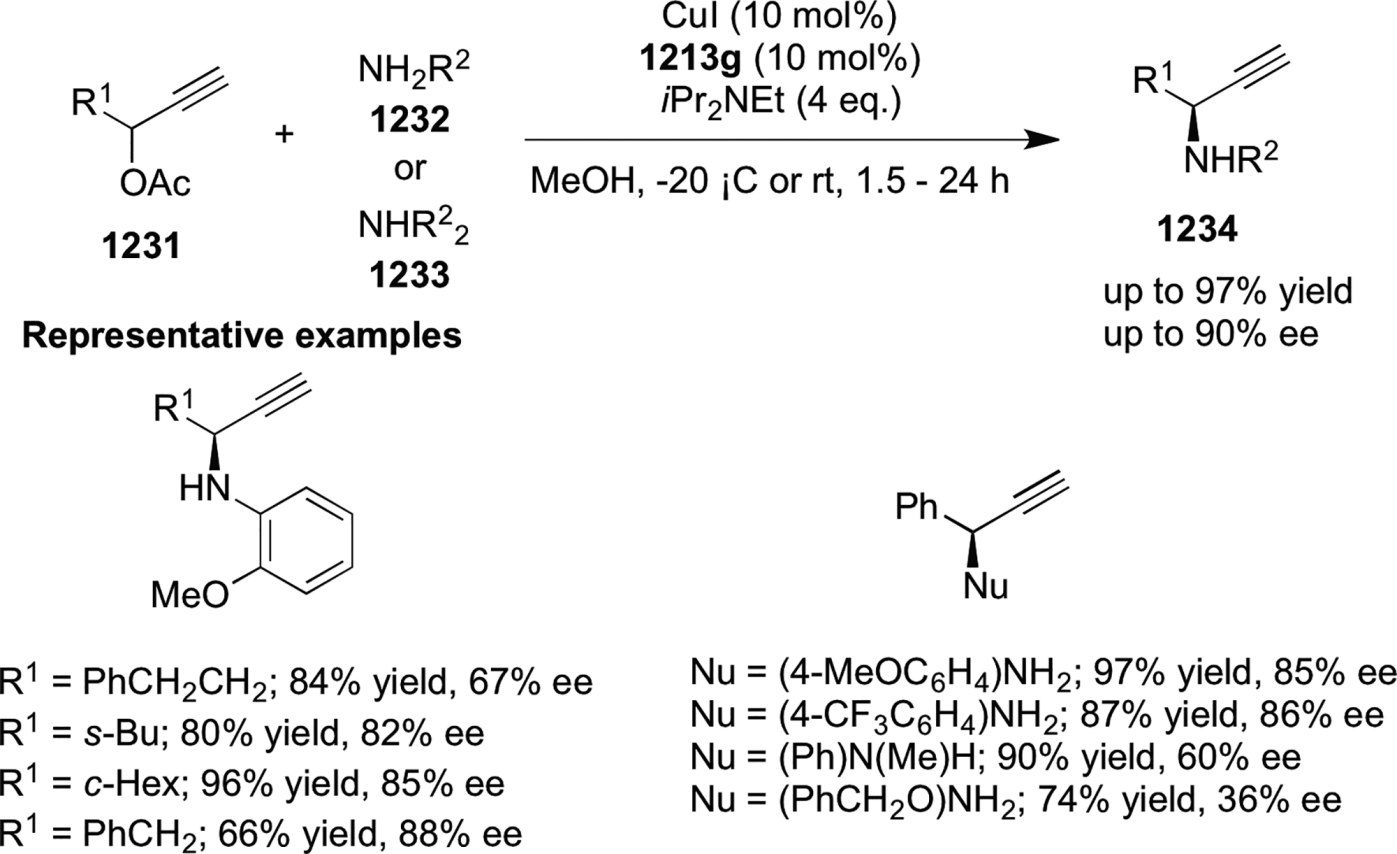
Asymmetric Cu-Catalyzed Propargylation of Amines

Maarseveen prepared a range of chiral propargylic amines via a Cu-**1213g**-catalyzed propargylation of both aromatic and nonaromatic amines with propargylic esters **1231**. Using a range of primary (**1232**) and secondary amines (**1233**), several propargylic amines **1234** were synthesized in up to 97% yield and 90% *ee* (Scheme 361). Aniline derivatives performed best as nucleophiles, giving the corresponding products in high yields and enantioselectivities, while other amines generally performed poorly. A number of *C*-nucleophiles were also tested, with indole derivatives providing the products in up to 91% yield and 98% *ee*. Some of the propargylic amines synthesized by this protocol were converted into α-amino acid derivatives and further elaborated to provide formal total syntheses of the biologically active compounds (+)-anisomycin and (−)-cytoxazone.^431^

**Scheme 362:**
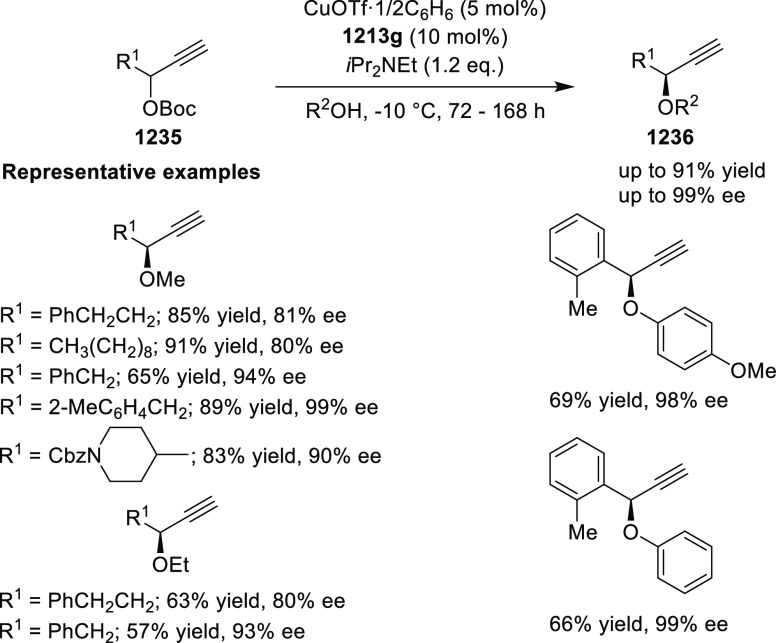
Asymmetric Cu-Catalyzed Propargylation of Alcohols

Nishibayashi employed a Cu-**1213g** complex in the enantioselective propargylic etherification of propargylic esters **1235** with alcohols (Scheme 362). Initially, a range of PyBOX ligands were tested in the reaction, giving low enantioselectivities when the reaction was performed at rt. Performing the reaction at a lower temperature of −10 °C for a longer reaction time (72 h) gave the propargyl ether products in generally high yields (57–91%) and enantioselectivities (79–99% *ee*) when using Me-PyBOX **1213g** as the chiral ligand. For reactions with MeOH and EtOH, the alcohol can be used as the reaction solvent. For reactions with phenol derivatives, 2 equiv of the corresponding phenol can be used in MeOH as the reaction solvent. A dimeric [Cu_2_(**PyBOX**)_2_][OTf] species was proposed as the active catalytic agent in the reaction based on experimental evidence.^432^

**Scheme 363:**
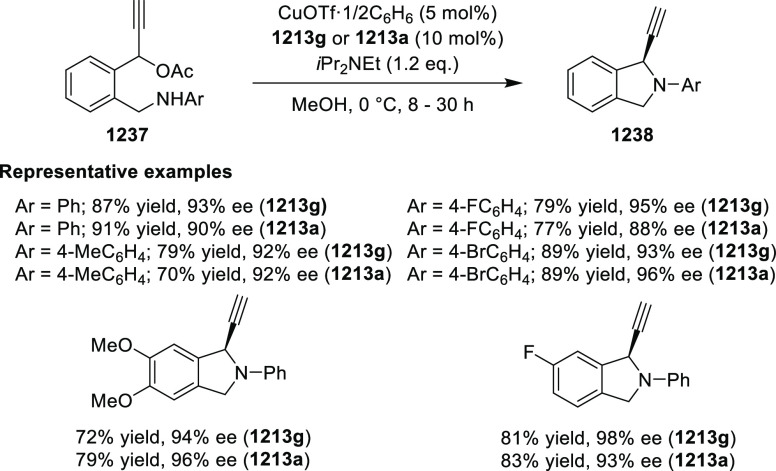
Asymmetric Cu-Catalyzed Intramolecular Amination of Propargylic Acetates

Nishibayashi employed Ph- and Me-PyBOX ligands **1213a** and **1213g** in a related Cu-catalyzed intramolecular amination of propargylic acetates **1237** to yield nitrogen-containing heterocycles **1238** bearing ethynyl groups in the α-position. A range of PyBOX ligands bearing both mono- and disubstitution on the oxazoline ring were tested, with both **1213a** and **1213g** performing well. A range of nitrogen heterocycles were successfully synthesized in good yields and high enantioselectivities of up to 98% *ee* (Scheme 363).^433^

**Scheme 364:**
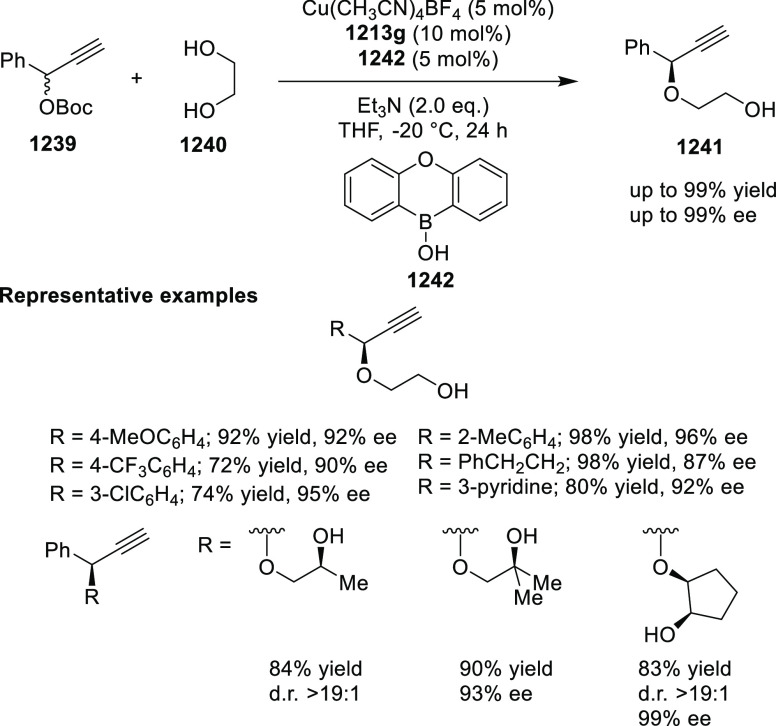
Asymmetric Cu-Catalyzed Desymmetrization of Diols with Propargylic Acetates

A Cu/boronic acid dual catalyzed enantioselective propargylation of polyols was reported by Niu and applied to the desymmetrization of *meso* 1,2-diols **1240** to furnish products with up to three stereocenters in one operation. Generally, aliphatic alcohols are not nucleophilic enough to engage in Cu-catalyzed propargylation reactions unless they are used as the reaction solvent, thus limiting the scope of the reaction to simple alcohols like MeOH and EtOH. Niu addressed this issue by taking advantage of the increased nucleophilicity of boronate complexes formed between diols and boronic acids. Subjecting propargylic esters **1239** to the reaction with various diols **1240** in the presence of [Cu(MeCN)_4_]PF_6_ and chiral Me-PyBOX ligand **1213g** the desymmetrized propargylic ethers **1241** were obtained in yields up to 99% and enantioselectivities up to 99% *ee* (Scheme 364). The authors suggested the presence of a dinuclear Cu species as the active catalyst in the reaction, based on Nishibayashi’s reports.^434^

**Scheme 365:**
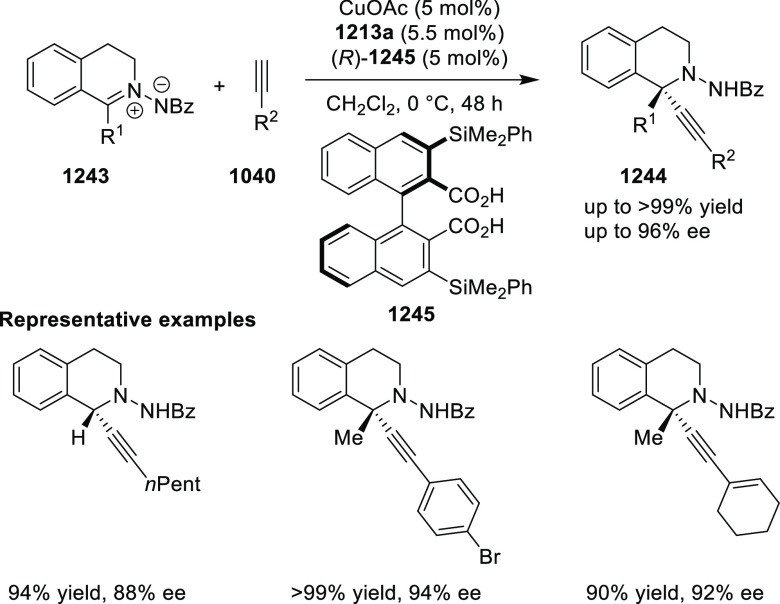
Cu/chiral Bronsted Acid-Catalyzed Asymmetric Alkynylation of *C*_1_-Substituted *C*,*N*-Cyclic Azomethine Imines

Maruoka has applied **1213a** in the catalytic asymmetric alkynylation of *C*_1_-substituted *C*,*N*-cyclic azomethine imines **1243** with a Cu(I)/chiral Bronsted acid cocatalyst system (Scheme 365). This is the first example of an asymmetric direct alkyne addition to a *CN* double bond to give tetrasubstituted carbon centers in high stereoselectivity. A range of nitrogen heterocyclic products **1244** with tertiary and quaternary alkyl-substituted stereocenters were generated in excellent yields up to >99% and excellent enantioselectivities up to 96% *ee*. The tertiary stereocenters were formed in excellent yields and enantioselectivities without the need for a chiral Bronsted acid additive **1245**, however in the case of products containing a quaternary stereocenter, the additive dramatically improved the stereoselectivity of the process.^435^

**Scheme 366:**
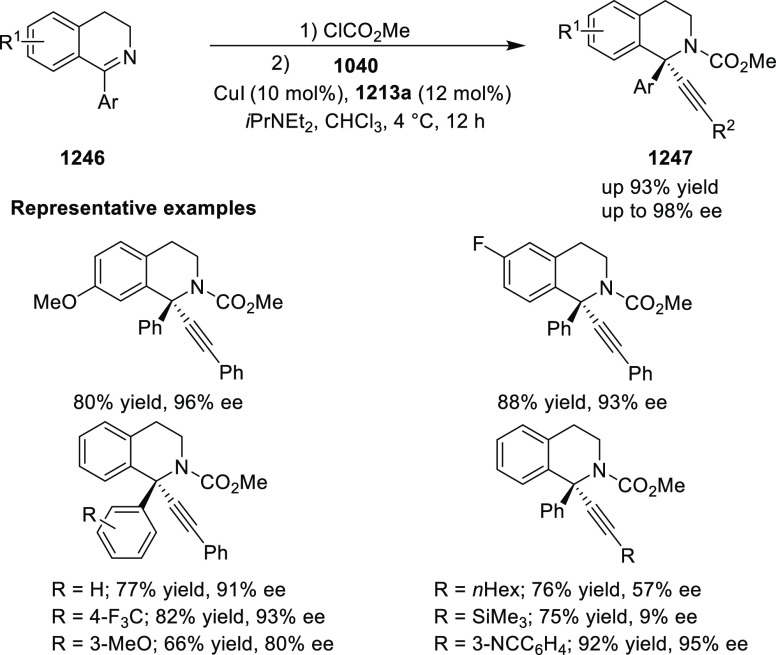
Cu-Catalyzed Asymmetric Alkynylation of Diaryl Ketimines

Watson later reported a similar Cu-catalyzed asymmetric alkynylation of diaryl ketimines **1246** (Scheme 366). Utilizing Ph-PyBOX ligand **1213a**, diaryl ketimines **1246** were first reacted with ClCO_2_Me to form an iminium ion, followed by the CuI/**1213a**-catalyzed addition of an alkyne to form a range of *C*_1_-tetrasubstituted nitrogen heterocyclic products **1247** in generally excellent yields up to 93% and enantioselectivities up to 98% *ee*. The reactions were run under basic conditions without the need for a chiral Bronsted acid additive. A wide range of aryl groups were tolerated on the ketimine **1246**; however, no heteroaryl groups were reported. The alkyne was found to tolerate a range of substituted aryl groups, however alkyl and silyl groups were detrimental to the stereoselectivity of the process.^436^

**Scheme 367:**
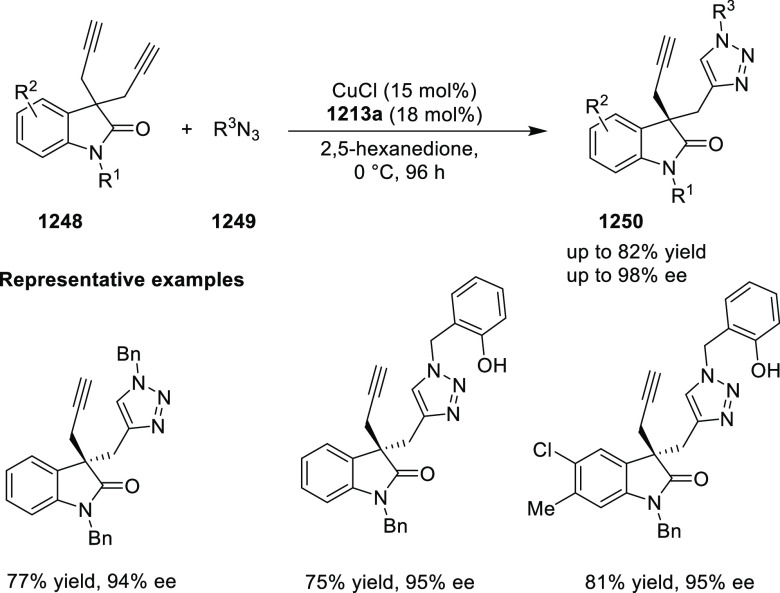
Enantioselective Cu-Catalyzed Azide–Alkyne Cycloaddition *via* Desymmetrization of Oxindole-Based 1,5-Heptadiynes

Zhou has applied ligand **1213a** in the first highly enantioselective Cu-catalyzed azide–alkyne cycloaddition (CuAAC) via desymmetrization of oxindole-based 1,5-heptadiynes **1248** to furnish chiral quaternary oxindoles **1250** bearing a 1,2,3-triazole moiety (Scheme 367). A range of azides and oxindoles were demonstrated to perform well in the reaction providing the products in good yields of up to 82% and excellent enantioselectivities of up to 98% *ee*, with the construction of all-carbon quaternary stereogenic centers.^437^

**Scheme 368:**
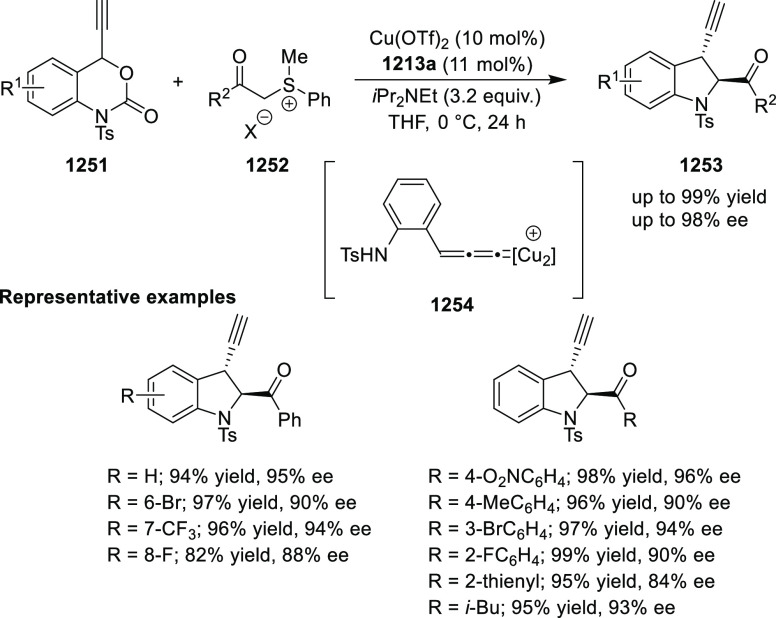
Asymmetric Decarboxylative [4 + 1] Cycloaddition of Propargylic Carbamates with Sulfur Ylides

Li and Xiao reported the first decarboxylative [4 + 1] cycloaddition of propargylic carbamates **1251** with sulfur ylides (Scheme 368). A range of ethynyl benzoxazinanones **1251** with varying aryl substitution patterns were subjected to the reaction with sulfonium salts **1252**, bearing different ketonic moieties, in the presence of Cu(OTf)_2_ and chiral Ph-PyBOX ligand **1213a** to yield chiral indolines **1253** in high yields of up to 99% and with excellent enantioselectivities of up to 98% *ee*. The proposed mechanism for the reaction involves a key Cu-allenylidene intermediate **1254**, which undergoes a formal [4 + 1] cycloaddition with the *in situ* formed sulfur ylide.^438^

**Scheme 369:**
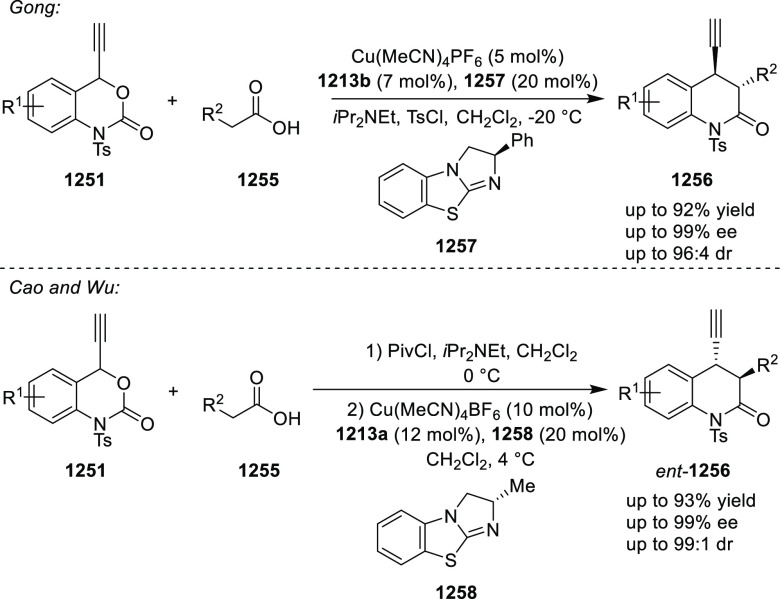
Asymmetric Cu-Catalyzed [4 + 2] Annulation of Ethynyl Benzoxazinanones with *In Situ* Generated Carboxylic Acid/Nucleophilic Lewis Base Derived Enolates

Gong^439^ and Cao and Wu^440^ independently reported the use of ethynyl benzoxazinanones **1251** in a similar Cu-catalyzed [4 + 2] annulation of the same copper allenylidene intermediate **1254** with *in situ* generated carboxylic acid/nucleophilic Lewis base derived enolates. Gong utilized *i*Pr-PyBOX ligand **1213b** to achieve high levels of stereoinduction of up to 99% *ee*, while Cao and Wu utilized Ph-PyBOX **1213a**, also achieving enantioselectivities of up to 99% *ee* (Scheme 369). Interestingly, Cao and Wu reported the opposite absolute stereochemistry of the quinolinone products **1256** to that reported by Gong (both reports determined the absolute stereochemistry by X-ray crystallographic analysis of the product). The (*R*,*R*)-PyBOX ligands (Ph **1213a** or *i*Pr **1213b**) were applied in both cases, while the nucleophilic Lewis base catalysts **1257** and **1258**, with opposite senses of stereochemistry were used. In both reports, a switch in the stereochemistry of the nucleophilic Lewis base catalyst led to the formation of the opposite enantiomer, so the nucleophilic Lewis base catalyst controls the absolute stereochemistry of the product of this cascade process. In fact, Gong performed the reaction in the presence of an achiral PyBOX ligand, isolating the product quinolinone in a slightly lower 92% *ee*, but with a much lower diastereoselectivity. Multiple Cu(PyBOX) catalytic systems have been shown to be proficient in the enantioselective [4 + 2] cycloaddition of Cu-allenylidenes (derived from ethynyl benzoxazinanones) with multiple synthetic partners.^441−443^

**Scheme 370:**
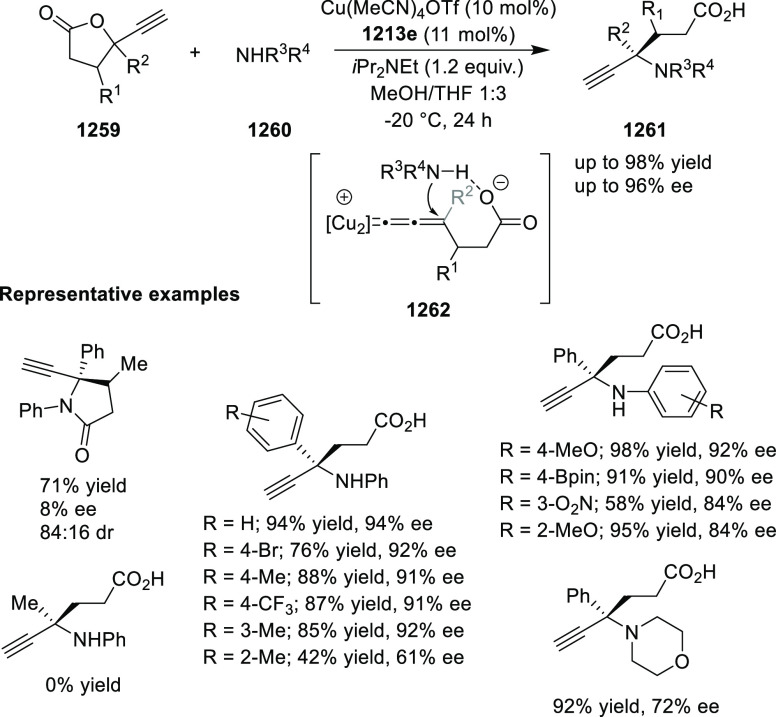
Cu-Catalyzed Asymmetric Synthesis of γ-Amino Acids

Kleij has developed a somewhat related Cu-catalyzed asymmetric synthesis of γ-amino acids bearing quaternary stereocenters. Utilizing chiral naphthyl-PyBOX ligand **1213e**, propargylic lactones **1259** undergo an enantioselective Cu-catalyzed amination with a variety of primary amines and one secondary amine **1260** to give the corresponding γ-amino acids **1261** in up to 98% yield and with up to 96% *ee* (Scheme 370). The proposed mechanism for this transformation involves a similar Cu-allenylidene intermediate **1262** which is nucleophilically attacked by the amine.^444^ Zhang has described a very similar Cu(**1213d**)-catalyzed enantioselective synthesis of β-amino alcohols, utilizing cyclic carbonates in place of lactones.^445^

**Scheme 371:**
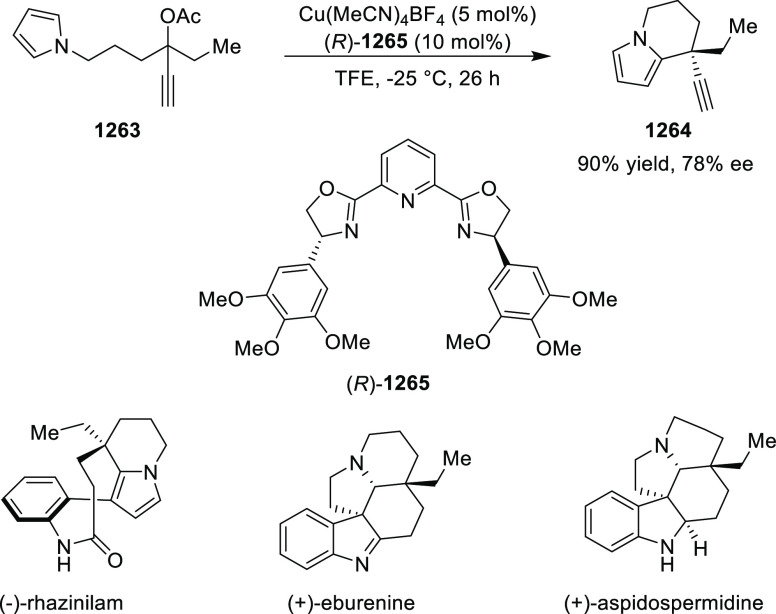
Asymmetric Cu-Catalyzed Propargylation for the Total Synthesis of (−)-Rhazinilam, Aspidospermidine, and (+)-Eburenine

Carreria has utilized the electron rich 3,4,5-trimethoxyphenyl PyBOX ligand (*R*,*R*)-**1265** in a Cu-catalyzed propargylation of an internal *C*-nucelophile as the key step in the enantioselective total synthesis of three natural products. Propargyl acetate **1263** was subjected to the reaction, which proceeds through a Cu-allenylidene intermediate, to give pyrrole **1264** containing the core stereogenic center of (−)-rhazinilam, in 90% yield and 78% *ee* (Scheme 371). (−)-Rhazinilam was synthesized in six additional steps. Pyrrole **1264** was also taken forward to synthesize (+)-eburenine in five additional steps. (+)-Aspidospermidine was synthesized in one step from (+)-eburenine.^446^

**Scheme 372:**
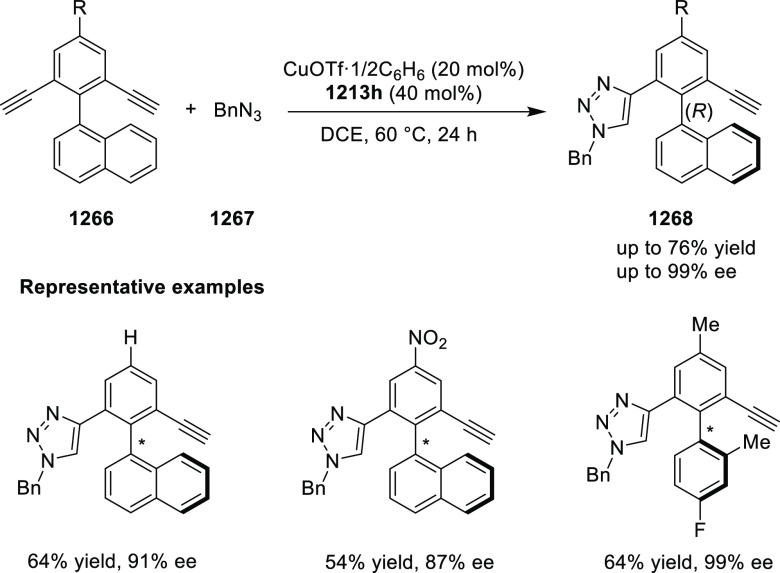
Enantioposition-Selective CuAAC to Construct Axially Chiral Biaryl Derivatives

Uozumi has developed an enantioposition-selective CuAAC to construct axially chiral biaryl derivatives by employing the l-serine-derived, di-OTBS protected PyBOX ligand **1213h**. The reaction of benzyl azide **1267** with prochiral biaryl dialkynes **1266** led to the formation of 1,2,3-triazoles **1268** bearing axially chiral biaryl groups in up to 76% yield and 99% *ee* (Scheme 372). The reaction was limited to the use of benzyl azide for good enantioposition-selectivity; however, changes to the top aryl ring were well tolerated, as was the use of both naphthalene and *ortho*-substituted benzene rings as the bottom aryl moiety.^447^

**Scheme 373:**
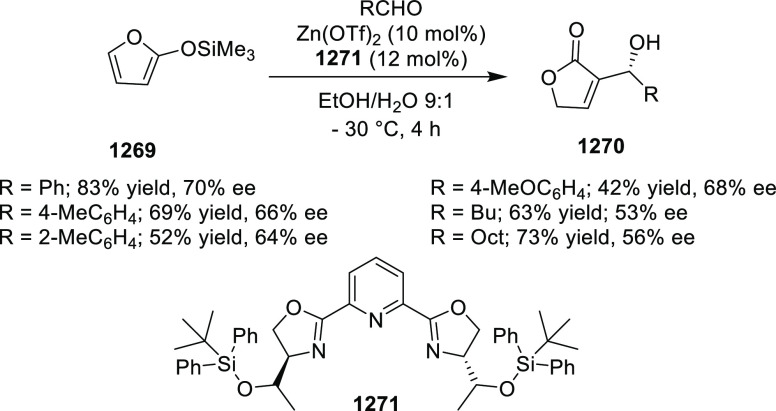
Asymmetric Zn-Catalyzed Mukaiyama Aldol Reaction of 2-(Trimethoxylsiloxy)-furans

Mlynarski applied the highly hindered TBDPS *O*-protected chiral PyBOX ligand **1271** in an unprecedented asymmetric Zn-catalyzed Mukaiyama aldol reaction of 2-(trimethoxylsiloxy)-furans **1269** with various aldehydes (Scheme 373). Some commercially available PyBOX ligands were tested in the reaction with little success, leading the authors to employ a more hindered PyBOX ligand **1271** which proved essential in obtaining decent levels of asymmetric induction. Several chiral α-butenolides **1270** were obtained in good yields of up to 82% and moderate to good enantioselectivies of up to 70% *ee*. A benzoic acid additive (10 mol %) was found to promote the reaction in the case of aryl aldehydes, but was not welcome in the reaction of aliphatic aldehydes.^448^

**Scheme 374:**
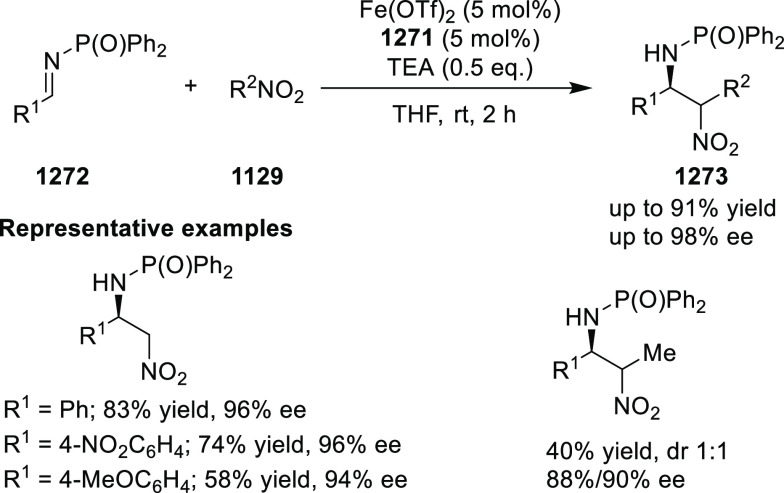
Fe-Catalyzed Nitro-Mannich Reaction for Enantioselective Synthesis of β-Nitroamines

Mlynarski has applied the same chiral PyBOX ligand **1271** in an Fe-catalyzed nitro-Mannich reaction for the enantioselective synthesis of β-nitroamines **1273** (Scheme 374). A wide range of chiral PyBOX ligands were found to induce good enantioselectivities with the very hindered PyBOX **1271** giving the best results. A range of β-nitroamines **1273** were prepared with good yields of up to 91% and consistently high enantioselectivies of up to 98% *ee*.^449^

**Scheme 375:**
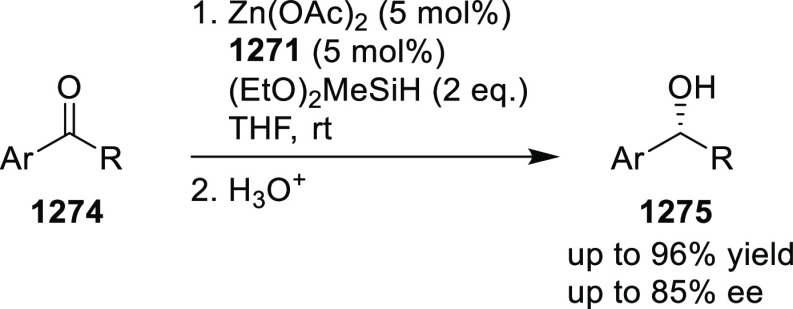
Enantioselective Zn-Catalyzed Hydrosilylation of Aromatic Ketones

Utilizing the same hindered PyBOX ligand **1271**, Mlynarski has developed an enantioselective Zn-catalyzed hydrosilylation of aromatic ketones **1274** with (EtO)_2_MeSiH (Scheme 375). A range of chiral secondary alcohols **1275** were obtained in good yields up to 96% (generally >99% conv.) and moderate to good enantiomeric excesses of up to 85% *ee*.^450^

**Scheme 376:**
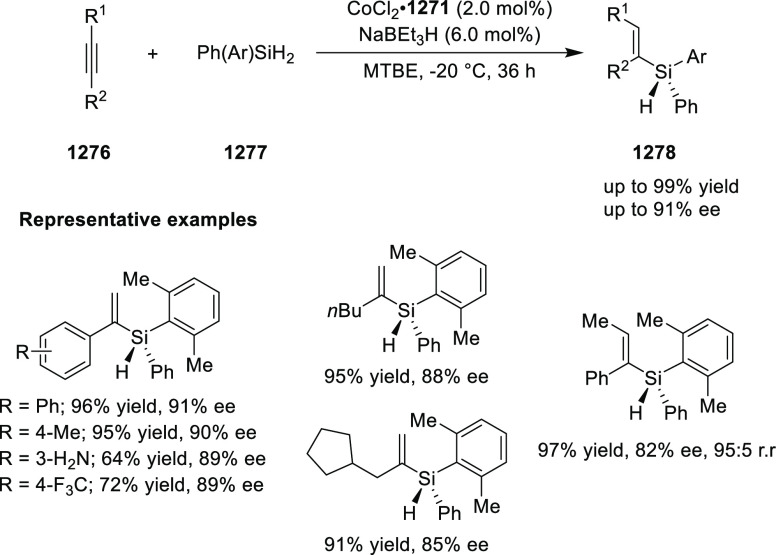
Asymmetric Co-Catalyzed Regioselective Alkyne Hydrosilylation

Huang utilized **1271** in an asymmetric Co-catalyzed regioselective alkyne hydrosilylation with dihydrosilane **1277**. A range of aryl, alkyl, internal and terminal alkynes **1276** were applied in the hydrosilylation to yield alkenes **1278** bearing silicon stereogenic centers in up to 99% yield and with up to 91% *ee* (Scheme 376). Reactions with terminal alkynes proceeded with high Markovnikov regioselectivity.^451^

**Scheme 377:**
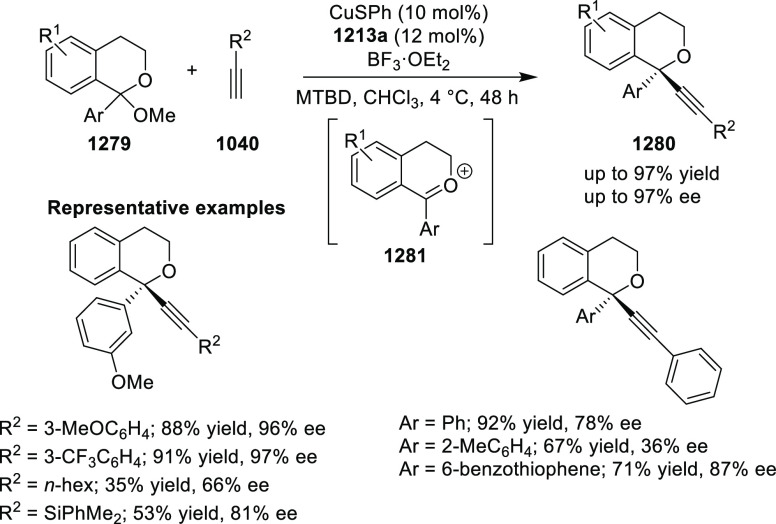
Asymmetric Cu-Catalyzed Alkynylation of Oxocarbenium Ions

Watson has reported a Cu-catalyzed alkynylation of oxocarbenium ions **1281** derived from isochroman acetals **1279**, utilizing chiral PyBOX ligand **1213a** to access tetrasubstituted biaryl stereocenters in high enantioselectivities. A range of alkynes **1040** can be added to isochroman oxocarbenium species **1281** bearing a variety of aryl groups to access isochroman ketals **1280** in high yields of up to 97% and enantioselectivities of up to 97% *ee* (Scheme 377). Changes to the substitution on the aryl group of the isochroman acetals **1279** affected the stereoselectivity of the reaction, for example when the 2-methyl substituted substrate was reacted with phenylacetylene, the product was isolated in 36% *ee*. Generally, aryl- and silane-based acetylenes performed well, with alkylacetylenes giving the product with lower enantioselectivities. For example, subjecting *n*-hexaneacetylene to the reaction with the 3-MeO-substituted isochroman acetal gave the product in only 66% *ee*, as compared to 81% *ee* for -SiPhMe_2_ acetylene.^452^

**Scheme 378:**
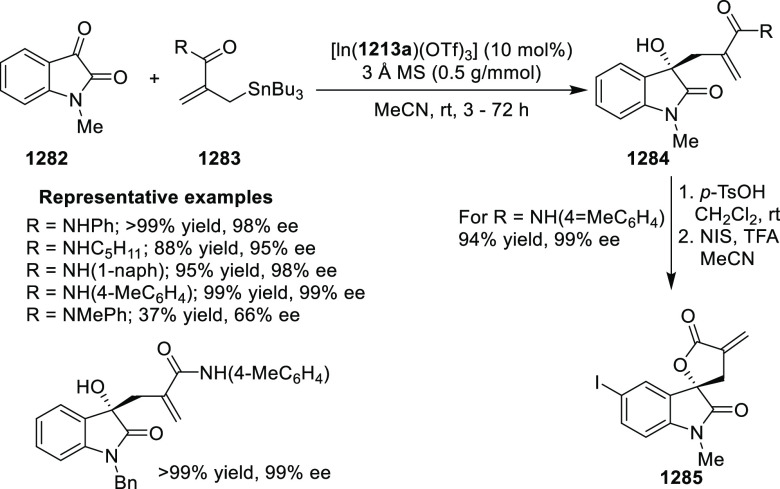
Asymmetric In-Catalyzed Amide Allylation of *N*-Methyl Isatin

Yoda has described an asymmetric In-catalyzed amide allylation of *N*-methyl isatin **1282** with *N*-substituted-β-amido allyltributylstannanes **1283** to synthesize the corresponding chiral allylated products **1284** in excellent yields of up to >99% and enantioselectivities of up to 99% *ee* (Scheme 378). Further derivatization of the R = NH(4-MeC_6_H_4_) product, by means of an acid promoted cyclization followed by *C*_5_-iodination, led to the formation of an antineoplastic spiro-fused 2-oxindole/*R*-methylene-γ-butyrolactone **1285**, retaining the high optical purity of 99% *ee*.^453^ A follow-up report by Yoda expanded the substrate scope to isatins bearing different *N*-protecting groups and *C*_5_-substitution, retaining the excellent yields and enantioselectivities from the previous report.^454^

**Scheme 379:**
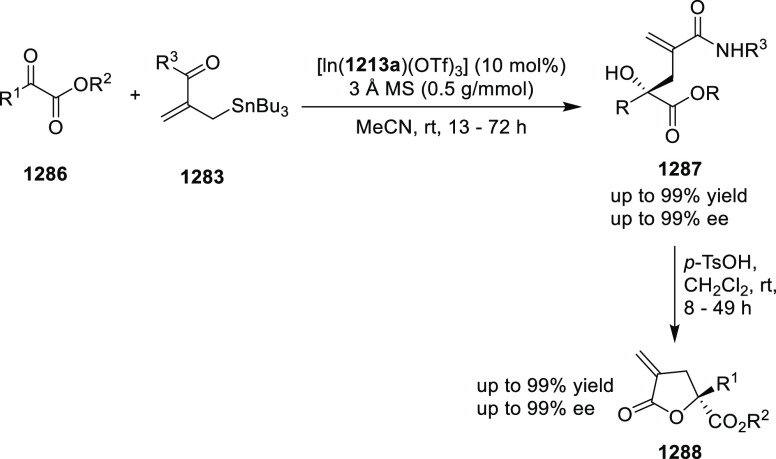
In-Catalyzed Amide Allylation with Allylation/Lactonization of α-Keto Esters

Yoda further explored the In-catalyzed amide allylation with the allylation/lactonization of α-keto esters **1286** for the enantioselective synthesis of ester-functionalized α-methylene-γ-butyrolactones **1288** in excellent yields of up to 99% and enantiomeric excesses of up to 99% (Scheme 379). Following the [In(**1213a**)(OTf)_3_]-catalyzed allylation of α-keto esters **1286** with allylstannanes **1283**, an acid promoted lactonization of the corresponding amides **1287** gives access to the α-methylene-γ-butyrolactones **1288**.^455^

**Scheme 380:**
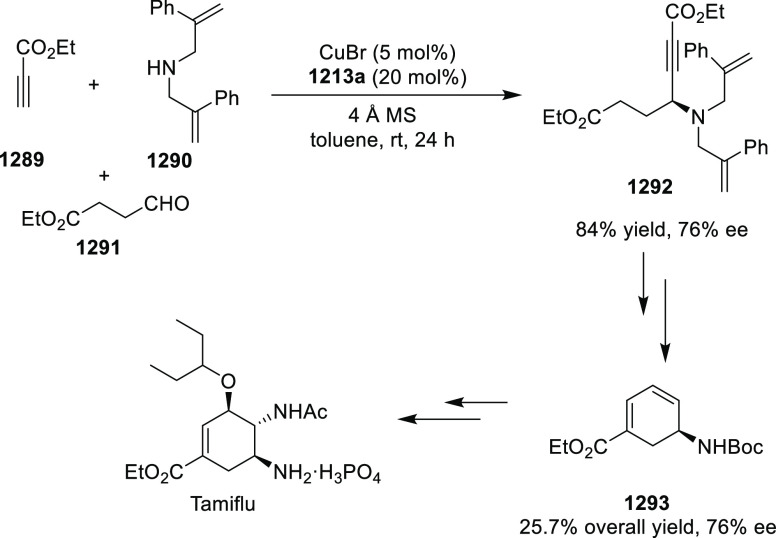
Asymmetric Synthesis of Tamiflu

Watanabe and Shibasaki reported the use of chiral PyBOX ligand **1213a** in an asymmetric Cu-catalyzed A^3^-coupling reaction for the synthesis of an oseltamivir phosphate precursor **1293** in 84% yield and 76% *ee* (Scheme 380). This propargylic amine **1292** was taken forward to synthesize a direct precursor of oseltamivir phosphate consisting of 5 purification steps, 25.7% overall yield and an optical purity of 76% *ee*. This sequence represents a formal total synthesis of the antiviral drug Tamiflu.^456^

**Scheme 381:**
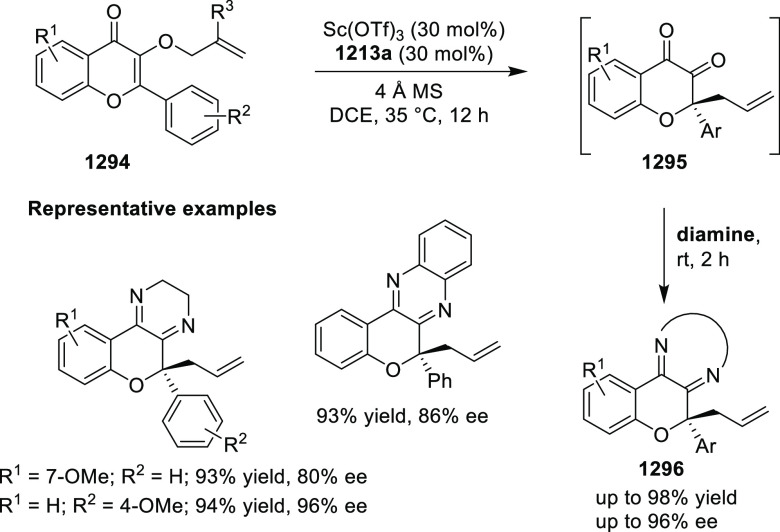
Asymmetric Sc-Catalyzed Rearrangement of 3-Allyloxyflavones for the Preparation of Chiral 3,4-Chromanediones

Porco and Schaus have reported the asymmetric Sc-catalyzed rearrangement of 3-allyloxyflavones **1294** for the preparation of chiral 3,4-chromanediones **1295**. For purification purposes, the authors condensed the dicarbonyl products with various diamines to yield the corresponding dihydopyrazines **1296**. Employing chiral PyBOX ligand **1213a**, the dihydropyrazines **1296** could be accessed in high yields of up to 98% and enantioselectivities of up to 96% *ee* (Scheme 381). Mechanistic studies support the intramolecular rearrangement pathway proceeding through a benzopyrylium intermediate.^457^

**Scheme 382:**
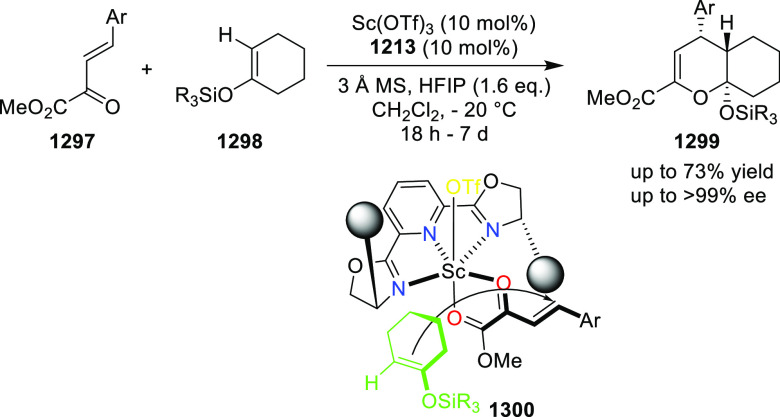
Enantioselective Formal Hetero-Diels–Alder Reaction of (*E*)-4-Aryl-2-oxo-3-butenoates

Desimoni studied the enantioselective formal hetero-Diels–Alder (HDA) reaction of (*E*)-4-aryl-2-oxo-3-butenoates **1297** utilizing **1213**-Sc(III) catalysts (Scheme 382). The authors found that these compounds, behaving as α-dicarbonyl derivatives, operate through a bidentate coordination to form rigid complexes that are characterized by a 5-membered structure. The reaction intermediate (as shown in **1300**) gives excellent facial discrimination, determined by the configuration of the PyBOX *C*_4_-substituent in a tandem Mukaiyama–Michael addition/intramolecular ring closure, which is a formal HDA reaction. This process yields the products **1299** with *trans*–*trans*-fused junctions in bicyclic six to six-membered ring systems in up to 73% yield and >99% *ee*.^458^

**Scheme 383:**
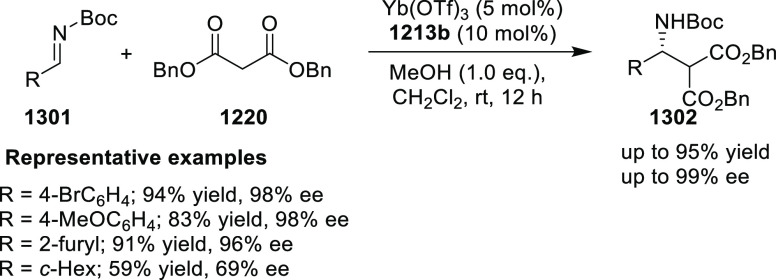
Asymmetric Yb-Catalyzed Mannich Reaction of Malonates with *N*-*tert*-Butoxycarbonyl (Boc) Imines

Karimi and Enders have described a highly efficient Yb(**1213b**)-catalyzed Mannich reaction of malonates with *N*-*tert*-butoxycarbonyl (Boc) imines **1301** to access the corresponding chiral amines **1302** in excellent yields and enantioselectivities (Scheme 383). Using MeOH as an additive, the authors could synthesize a range of chiral amines in up to 95% yield and 99% *ee*. They found the substituent on the *N*-atom of the imine played an important role in the formation of the product, with only **N*-*Boc imines allowing the reaction to proceed.^459^

**Scheme 384:**
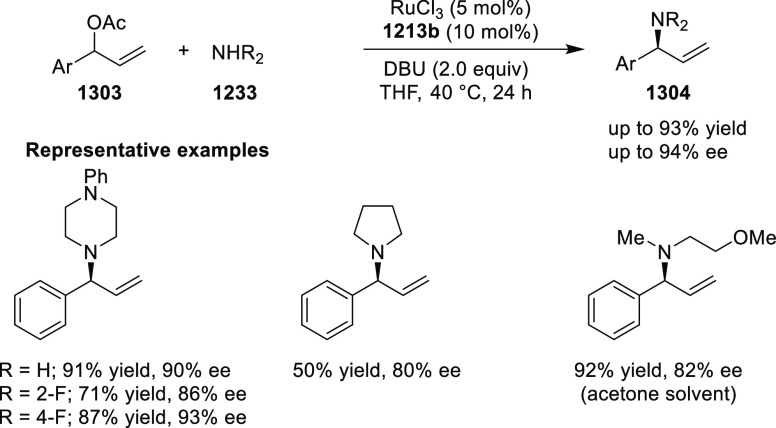
Ru-Catalyzed Asymmetric Allylic Amination of Racemic 1-Aryl Allyl Esters with Amines

Kawatsura and Itoh have demonstrated a RuCl_3_/**1213b**-catalyzed allylic amination of racemic 1-aryl allyl esters **1303** with amines **1233**. Using mainly cyclic secondary amines, they could access enantiomerically enriched allylic amine products **1304** in good yields up to 93% and high enantioselectivities of up to 94% *ee* (Scheme 384). The reaction was shown to proceed with perfect regioselectivity for several allyl esters.^460^

**Scheme 385:**
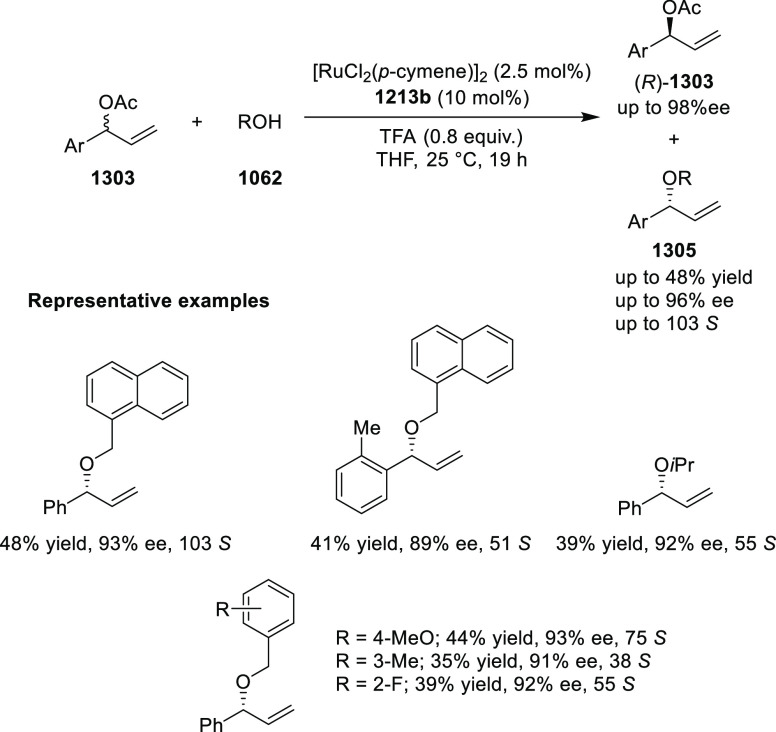
Kinetic Resolution of Allyl Acetates *via* a Ru-Catalyzed Asymmetric Etherification

Kawatsura has reported a related kinetic resolution of allyl acetates via a Ru-catalyzed asymmetric etherification. A variety of aryl allyl acetates **1303** were subjected to the reaction with alkyl and benzyl alcohols **1062** in the presence of [RuCl_2_(*p*-cymene)]_2_ and *i*Pr-PyBOX **1213b** to give the corresponding ethers **1305** in up to 48% yield, 96% *ee* and with selectivity factors (*S*) of up 103 (Scheme 385). The enantiomerically enriched allyl acetate (*R*)-**1303** could also be isolated in up to 98% *ee*. The reaction proceeded with perfect regioselectivity in all cases.^461^

**Scheme 386:**
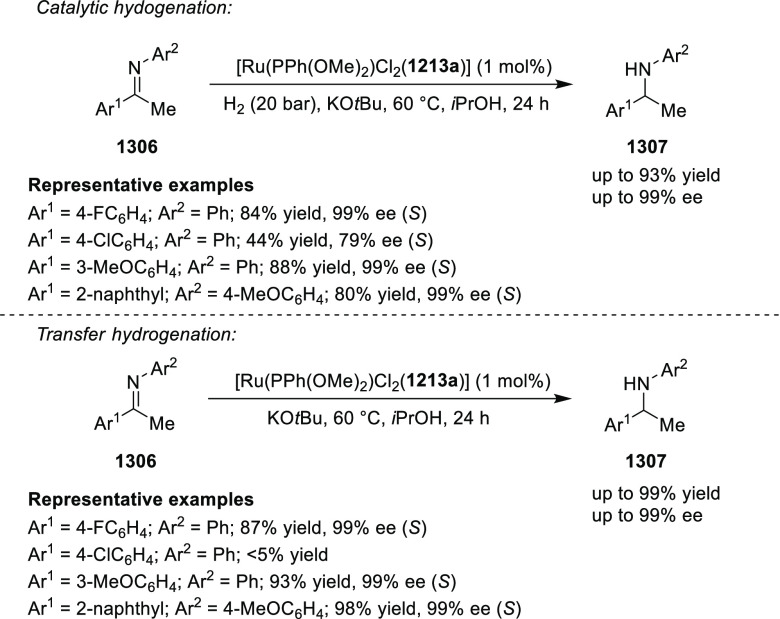
Ru-Catalyzed Asymmetric Hydrogenation and Asymmetric Transfer Hydrogenation of Imines

Gamasa and Pizzano have described a Ru-**1213a**-catalyzed hydrogenation and transfer hydrogenation of imines for the asymmetric synthesis of benzylamines. Subjecting diaryl imines **1306** to 1 mol% of the Ru catalyst in *i*PrOH at 60 °C under 20 bar H_2_ led to the isolation of enantioenriched benzyl amines **1307** in up to 93% yield and up to 99% *ee* (Scheme 386). Conducting the transfer hydrogenation under N_2_ in place of H_2_ led to the isolation of the enantioenriched benzyl amines **1307** in up to 99% yield and up to 99% *ee*. Overall, the two processes gave similar results. Substrates bearing Ar^1^ = 4-ClC_6_H_4_ or 4-BrC_6_H_4_ did not perform well under catalytic hydrogenation, while they were found to be completely incompatible with transfer hydrogenation.^462^

**Scheme 387:**
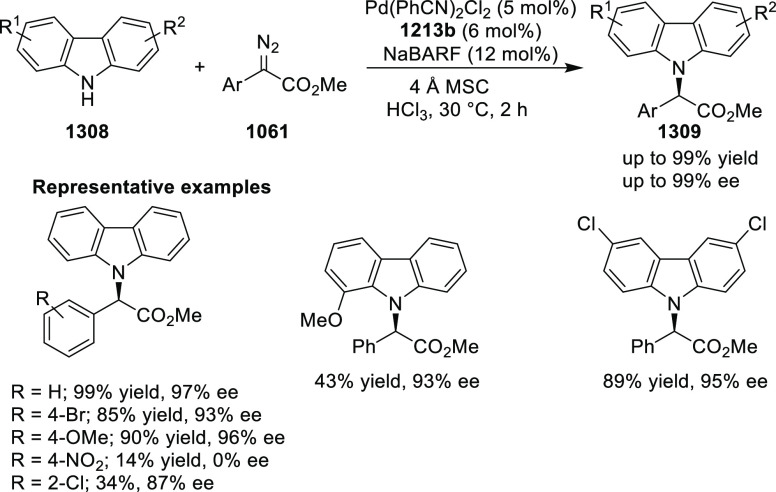
Enantioselective Pd-Catalyzed Carbene Insertion into Carbazole Derivatives

Van Vranken has reported an enantioselective Pd-catalyzed carbene insertion into carbazole derivatives **1308** (Scheme 387). While a range of oxazoline-containing chiral ligands were tested, the *i*Pr-PyBOX ligand **1213b** was found to induce the highest levels of enantioselectivity, giving the chiral amine products **1309** in up to 99% yield and 99% *ee*. Carbenes bearing a strongly electron-withdrawing group were found to perform poorly in the reaction. For example, the reaction of a 4-nitro-substituted carbene with carbazole led to the formation of the product as a racemate.^463^

**Scheme 388:**
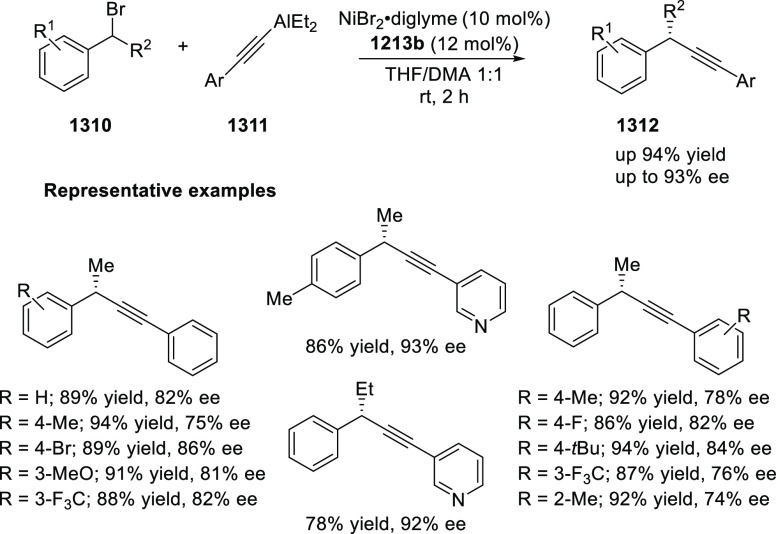
Ni-Catalyzed Asymmetric Cross-Coupling Reaction of Secondary Benzylic Bromides with Aryl Alkynyl Aluminum Reagents

Zhou has reported a Ni-catalyzed asymmetric cross-coupling reaction of secondary benzylic bromides **1310** with aryl alkynyl aluminum reagents **1311** (Scheme 388). *i*Pr-PyBOX ligand **1213b** was found to effectively induce good to high levels of enantioselectivity, leading to the isolation of a range of internal alkyne products **1312** in up to 94% yield and with up to 93% *ee*. Other PyBOX and BOX ligands were tested in the reaction, but these did not give comparable levels of stereoinduction. A range of aryl groups were well tolerated on the alkyl bromide coupling partner, while aryl and heteroaryl groups performed well on the alkynyl aluminum partner.^464^

**Scheme 389:**
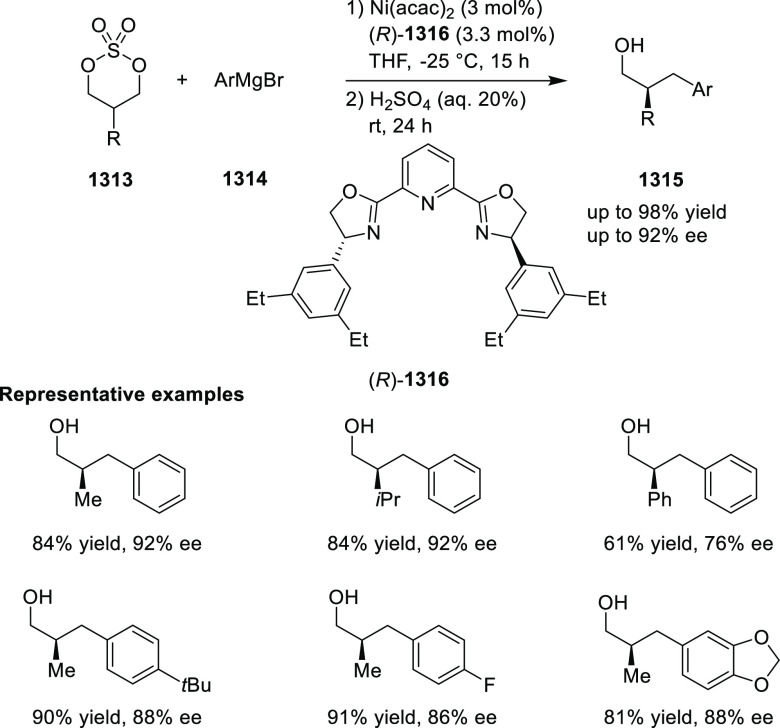
Asymmetric Ni-Catalyzed Kumada Cross-Coupling of Symmetric Cyclic Sulfates

Morken has applied 3,5-EtC_6_H_3_–PyBOX ligand **1316** in the development of an asymmetric Ni-catalyzed Kumada cross-coupling of symmetric cyclic sulfates **1313**. A range of aryl Grignard reagents **1314** were successfully coupled with the cyclic sulfates **1313** to give, following acid hydrolysis, chiral alcohols **1315** in up to 98% yield and with high enantioselectivities up to 92% *ee* (Scheme 389).^465^

**Scheme 390:**
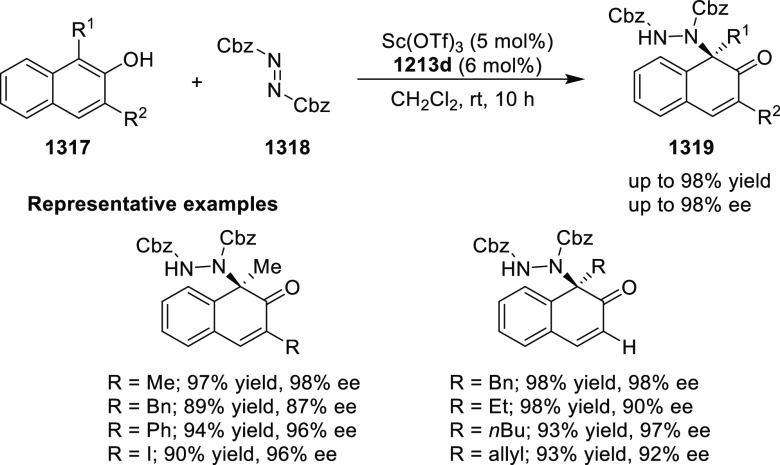
Asymmetric Dearomatization of Naphthols *via* a Sc-Catalyzed Electrophilic Amination Reaction with DEAD

Luan achieved an asymmetric dearomatization of naphthols **1317***via* a Sc-catalyzed electrophilic amination reaction with DEAD **1318**. A series of chiral PyBOX ligands were screened, but only ligand **1213d** gave high levels of asymmetric induction. Both monosubstituted 2-naphthols and disubstituted 1,3-naphthols performed well in the reaction, allowing access to the products **1319** in up to 98% yield and 98% *ee* with a wide substrate scope and at gram scale (Scheme 390).^466^

**Scheme 391:**
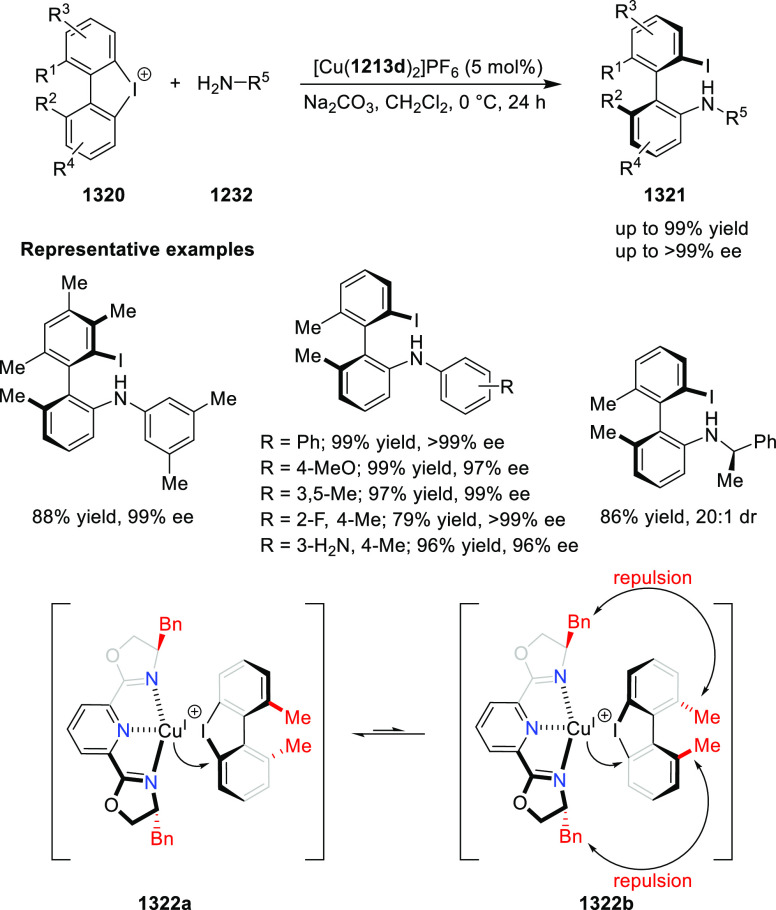
Cu-Catalyzed Asymmetric Ring-Opening Reaction of Diaryliodonium Salts

Fu and Gu have also utilized Bn-PyBOX ligand **1213d** in a Cu-catalyzed asymmetric ring-opening reaction of diaryliodonium salts **1320** with amines **1232** (Scheme 391). Diaryliodonium salts **1320** exist in two rapidly interconverting conformers. The interaction of the two conformers with the Cu(**1213d**) species leads to the formation of **1322a** and **1322b**. **1322b** is sterically disfavored due to the steric interaction between the Bn-group of the ligand and the methyl group of the diaryliodonium salt. Thus, **1322b** rapidly converts to **1322a** and undergoes the ring-opening reaction with the Cu(**1213d**) species, selectively establishing axial chirality. Base promoted coordination of the amine with the Cu-species, followed by reductive elimination leads to the formation of the products **1321**. A range of di-*o*-substituted diaryliodonium salts **1320** were successfully reacted with a range of aryl and benzylic amine **1232** giving the axially chiral products **1321** in up to 99% yield and >99% *ee*. It should be noted that the reaction works equally well when indanyl PyBOX ligand (*R*,*S*)-**1337** (below) is utilized.^467^ Further mechanistic insights, and an improved experimental procedure, in which the amine is added slowly via a syringe pump, leading to an expanded substrate scope (benzylic and aliphatic amines) were subsequently reported by Gu.^468^

**Scheme 392:**
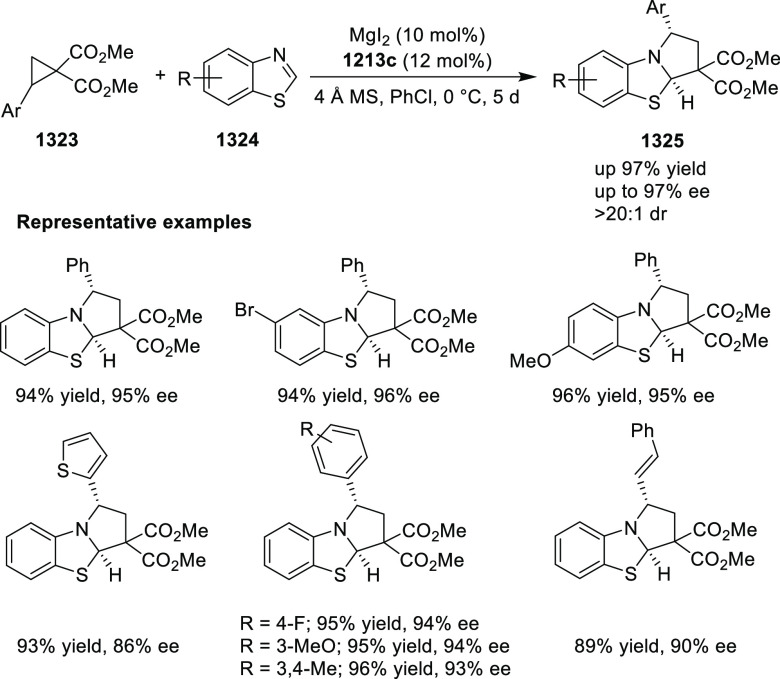
Mg-Catalyzed Asymmetric Dearomative [3 + 2] Cycloaddition of Benzotriazoles with Cyclopropane-1,1-dicarboxylates

You has described a MgI_2_/**1213c**-catalyzed asymmetric dearomative [3 + 2] cycloaddition of benzotriazoles **1324** with cyclopropane-1,1-dicarboxylates **1323** (Scheme 392). By subjecting a range of aryl- and alkene-substituted cyclopropanes **1323** to the reaction with a range of benzotriazoles **1324** in the presence of 4 Å MS at 0 °C for 5 days, the polycyclic products **1325** containing two stereocenters were isolated in excellent yields of up to 97%, with excellent enantioselectivities of up to 97% *ee* (only one example <90% *ee*) and all >20:1 dr. Conducting the reaction with both the enantiomerically pure (*S*)- and (*R*)-enantiomers of the cyclopropane **1323** showed that the catalyst preferentially reacts with the (*R*)-enantiomer. The results also suggested that this transformation is a simple kinetic resolution and not a dynamic kinetic resolution, with >2.0 equiv of the racemic cyclopropane required to achieve a high yield.^469^

**Scheme 393:**
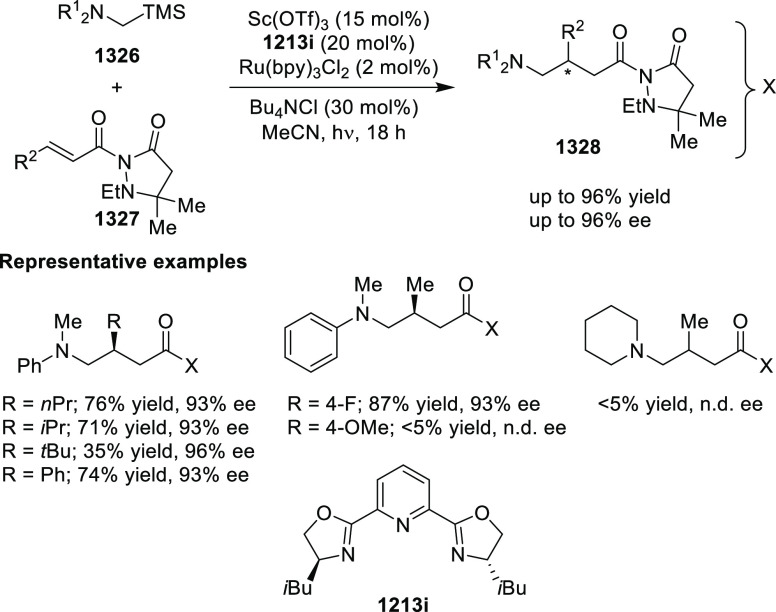
Asymmetric α-Amino Radical Conjugate Addition

Yoon has reported an asymmetric α-amino radical conjugate addition by the merging of photoredox catalysis and Lewis acid catalysis. Following a screen of isomers of chiral PyBOX ligand **1213c** they found that the *i*Bu-containing PyBOX ligand **1213i** in combination with Sc(OTf)_3_ and Ru(bpy)_3_Cl_2_, provided a system that was optimal for catalyzing the reaction and achieving high levels of stereoinduction. A range of chiral amines **1328** were synthesized in good yields of up to 96% and enantioselectivities of up to 96% *ee* (Scheme 393). Aryl amines appeared to perform best in the reaction, while the Michael acceptor bearing an auxiliary group X was required.^470^

**Scheme 394:**
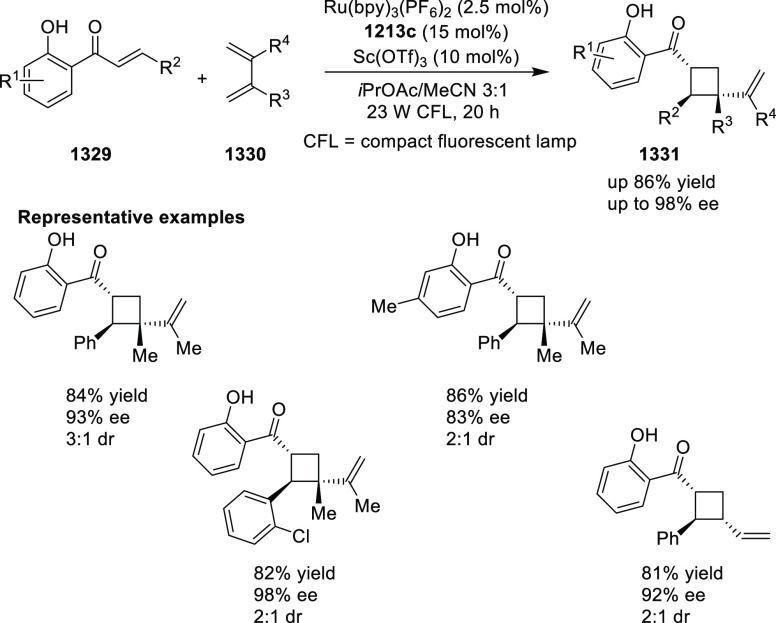
Chiral Lewis Acid-Catalyzed Triplet Energy Transfer Strategy for the Asymmetric [2 + 2] Cycloaddition of 2′-Hydroxychalchones with Dienes

Yoon subsequently described a chiral Lewis acid-catalyzed triplet energy transfer strategy for the asymmetric [2 + 2] cycloaddition of 2′-hydroxychalchones **1329** with dienes **1330**. They discovered that the coordination of a Sc(OTf)_3_-**1213c** complex to the 2′-hydroxychalchone substrates **1329** significantly decreased their triplet state energy, giving access to enantioselective reactions of the electronically excited states. A range of 2′-hydroxychalcones **1329** were subjected to the [2 + 2] cycloaddition with a small range of dienes **1330**, in the presence of Ru(bpy)(PF_6_) as the photosensitizer, to yield chiral cyclobutanes **1331** in high yields of up to 86%, excellent enantioselectivities of up to 98% *ee* and low diastereoselectivities of up to 4:1 dr (Scheme 394).^471^

**Scheme 395:**
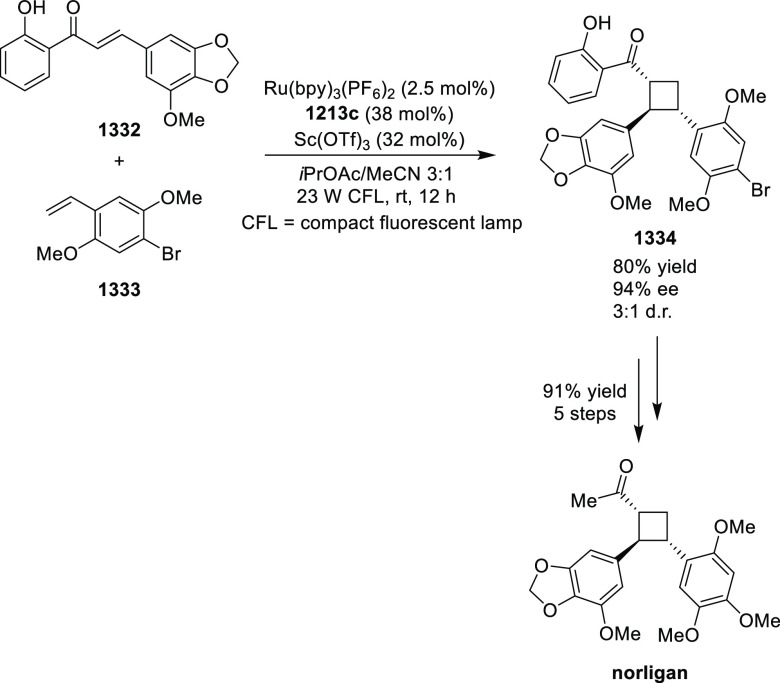
Asymmetric Total Synthesis of Norlignan

A later report by Yoon expanded the scope of the [2 + 2] cycloaddition to include styrene substrates in place of the dienes **1330**, maintaining the high levels of enantioselectivity. This strategy was applied in the total synthesis of norlignan, a diaryl cyclobutane natural product (Scheme 395).^472^

Overall, PyBOX ligands are some of the most used bis(oxazoline) ligands in asymmetric catalysis. They have been combined with a range of metal catalysts to induce excellent levels of stereoselectivity. The tridentate and planar PyBOX ligands appear to be particularly useful in Cu-catalyzed asymmetric transformations, such as progargylation and cycloaddition reactions. However, they have also been applied *inter alia* in the chemistries of Pd, Ni, Ru, Fe, Sc, and In. Notably, the application of these ligands in asymmetric photoredox catalysis is an important step forward. We predict there will be further developments in this area.

#### 3.2.13 Bis(oxazoline) Ligands with Pyridine Linkers and Disubstituted Oxazoline Rings

**Figure 66:**
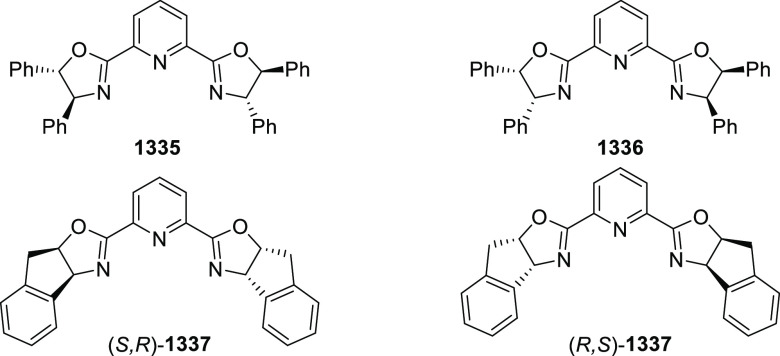
PyBOX ligands derived from disubstituted amino alcohols.

PyBOX ligands derived from disubstituted amino alcohols are commonly applied in a wide range of transformations in asymmetric catalysis (Figure 66).

**Scheme 396:**
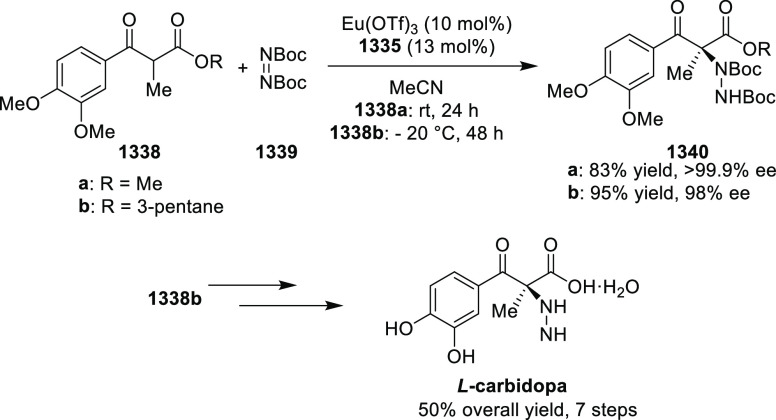
Asymmetric Synthesis of l-Carbidopa via an Enantioselective Eu-Catalyzed α-Amination of Acyclic β-Keto Esters

Vallribera has reported an enantioselective synthesis of l-carbidopa in 7 steps from β-keto ester **1338b**. The synthesis involves a key Eu-catalyzed α-amination of acyclic β-keto esters, utilizing diphenyl-PyBOX ligand (*R*,*R*)-**1335**. Monosubstituted PyBOX ligands **1213a**–**b** were found to give the product **1340a** with good enantioselectivities, although lower than (*R*,*R*)-**1335**. β-Ketoesters **1338a**–**b** were reacted with di-*tert*-butyl azodicarboxylate **1339** in the presence of Eu(OTf)_3_ and **1335** to yield the chiral amines **1340a** and **1340b** in 83% and 95% yields, respectively, and >99.9% and 98% *ee* (Scheme 396). Chiral amine **1340b** was brought forward to synthesize l-carbidopa in a 50% overall yield.^473^ Sodupe and Vallribera later applied this methodology to the asymmetric synthesis of fluorous l-carbidopa precursors with similarly high enantioselectivities of up to 99% *ee*.^474^

**Scheme 397:**
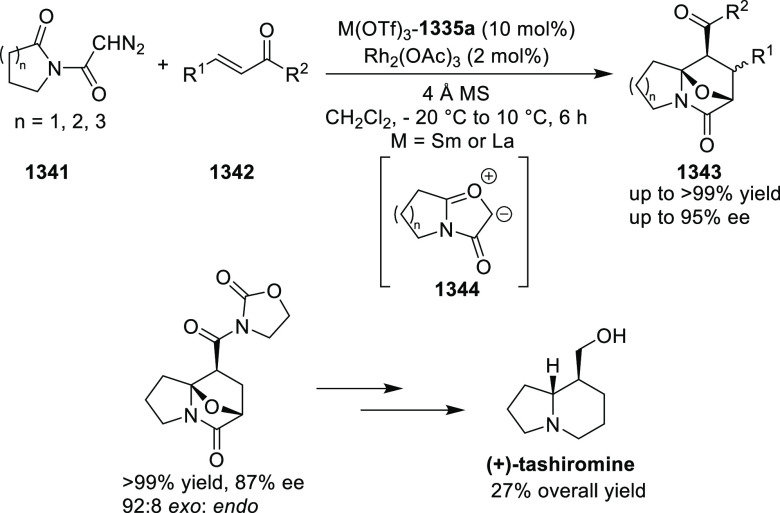
Chiral Lewis Acid-Catalyzed Asymmetric Cycloaddition of Carbonyl Ylides

Suga has described the chiral Lewis acid-catalyzed asymmetric cycloaddition of carbonyl ylides, employing chiral PyBOX ligand **1335**, for the synthesis of indolizidine alkaloids. The carbonyl ylides **1344** were first derived from various sized (*n* = 5, 6, 7) *N*-diazoacetyl lactams **1341** in the presence of Rh_2_(OAc)_4_, followed by the chiral Lewis acid-catalyzed cycloaddition with the appropriate dienophile **1342**, to yield the corresponding heterocycle **1343** (*n* = 5, 6, 7) in up to >99% yield and 95% *ee* (Scheme 397). This methodology was applied to the asymmetric total synthesis of the indolizidine alkaloid (+)-tashiromine in 27% overall yield.^475^

**Scheme 398:**
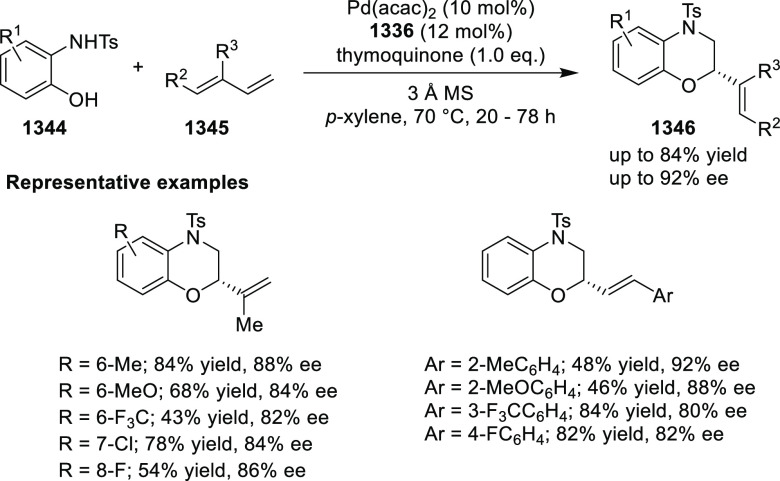
Pd-Catalyzed Regioselective Asymmetric Aminohydroxylation of 1,3-Dienes with *N*-Tosyl-2-aminophenols

Gong has reported a Pd-catalyzed regioselective asymmetric aminohydroxylation of 1,3-dienes **1345** with *N*-tosyl-2-aminophenols **1344**. Employing chiral PyBOX ligand **1336**, the corresponding 3,4-dihydro-2*H*-1,4-benzoxazine **1346** was formed with perfect regioselectivity for a range of substrates in up to 84% yield and 92% *ee* (Scheme 398). The reaction proceeds via a cascade aminopalladation (aza-Wacker)/asymmetric allylation sequence, with the ligand **1336** chelating in a bidentate-mode throughout.^476^

**Scheme 399:**
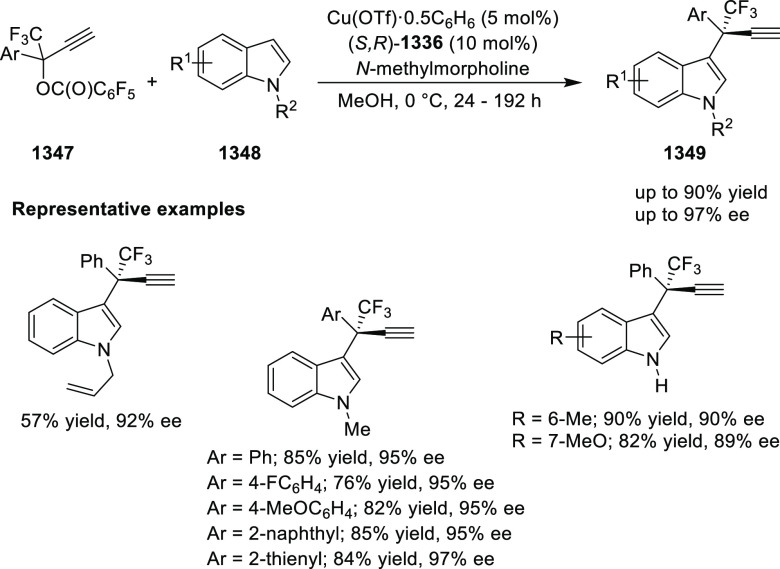
Enantioselective Cu-Catalyzed Propargylation of Indoles

Nishibayashi has described an enantioselective Cu-catalyzed propargylation of indoles **1348** with CF_3_-substituted propargylic esters **1347** for the construction of all-carbon quaternary stereocenters. Employing chiral PyBOX ligand (*S*,*R*)-**1336** in the Cu(OTf)·0.5C_6_H_6_-catalyzed process, a range of propargylic esters **1349** bearing different aryl groups were reacted with a range of indoles **1348** for the enantioselective synthesis of propargylic indoles **1349** in excellent yields of up to 90% yield and with up to 97% *ee* (Scheme 399).^477^

**Scheme 400:**
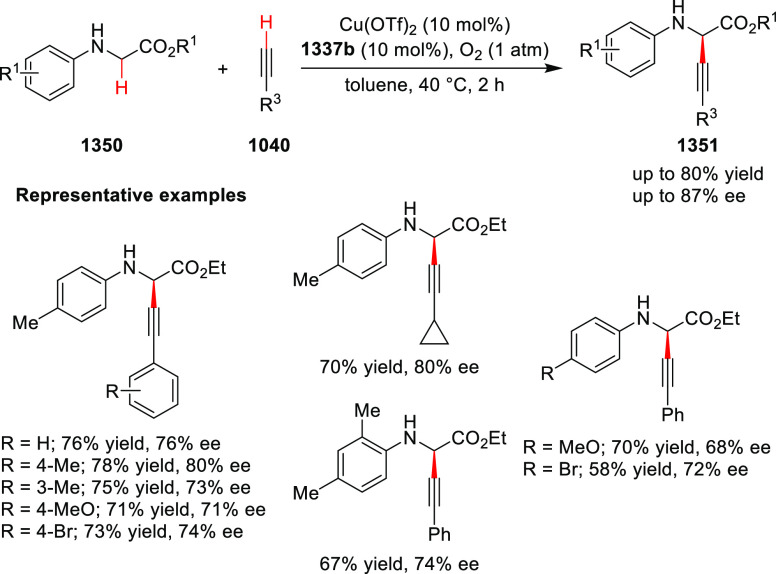
Cu-Catalyzed Asymmetric Dehydrogenative C_(sp3)_H–C_(sp)_H Cross-Coupling

Liu has described a Cu-catalyzed asymmetric dehydrogenative C_(sp3)_H–C_(sp)_H cross-coupling employing O_2_ as the terminal oxidant. Following a screen of multiple oxazoline-based chiral ligands, indanyl PyBOX ligand (*R*,*S*)-**1337** was found to induce moderate to high levels of enantioselectivity in the Cu-catalyzed cross-coupling of *N*-aryl glycine esters **1350** (C_(sp3)_H) with terminal alkynes **1040** (C_(sp)_H) to give the enantioenriched α-amino esters **1351** in up to 80% yield and with up to 87% *ee* (Scheme 400). A range of aryl and alkyl alkynes **1040** were well tolerated in the reaction giving access to enantioenriched non-natural α-amino acids.^478^

**Scheme 401:**
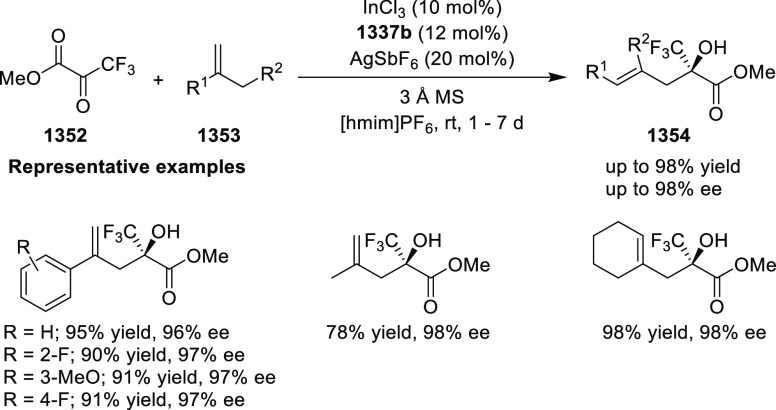
In-Catalyzed Asymmetric Ketone-ene Reaction of Trifluoropyruvate

Loh has reported a chiral In(II)-(*R*,*S*)-**1337** complex-catalyzed asymmetric ketone-ene reaction of trifluoropyruvate **1352** conducted in the ionic liquid [hmim]PF_6_. Subjecting a range of alkenes **1353** to the reaction led to the isolation of the corresponding tertiary allylic alcohols **1354** in high yields of up to 98% and excellent enantioselectivities of up to 98% *ee* (Scheme 401). The chiral In(II)-(*R*,*S*)-**1337** complex in the ionic liquid was recycled up to seven times with retention of high yields and enantioselectivities.^479^

**Scheme 402:**
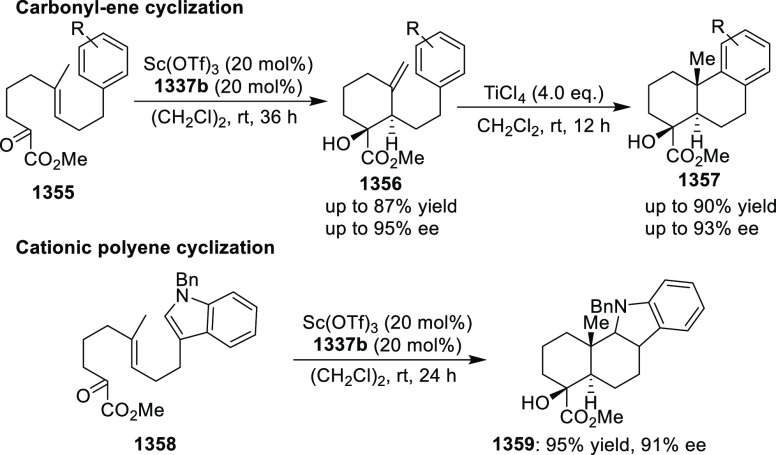
Asymmetric Carbonyl-ene and Cationic Polyene Cyclization Reactions

Loh has described a study on the asymmetric carbonyl-ene and cationic polyene cyclization reactions of 1,5-keto-olefins **1355** with Sc(III)-(*R*,*S*)-**1337** complexes. First, promoting the cyclization of 1,5-keto-olefins **1355** with a Sc(III)-(*R*,*S*)-**1337** complex led to the isolation of carbonyl-ene product **1356** in high yields up to 87% and enantioselectivities up to 95% *ee* (Scheme 402). Treatment of the ene-product **1356** with TiCl_4_ led to formation of the polyene-products **1357** without much loss in enantioselectivity. Subjecting substrates with terminator groups which are more nucleophilic, like furan, indole or tetrasubstituted alkenes, to the reaction conditions led to formation of the polyene-products **1359** in high yields and enantioselectivities without the need for TiCl_4_.^480^

**Scheme 403:**
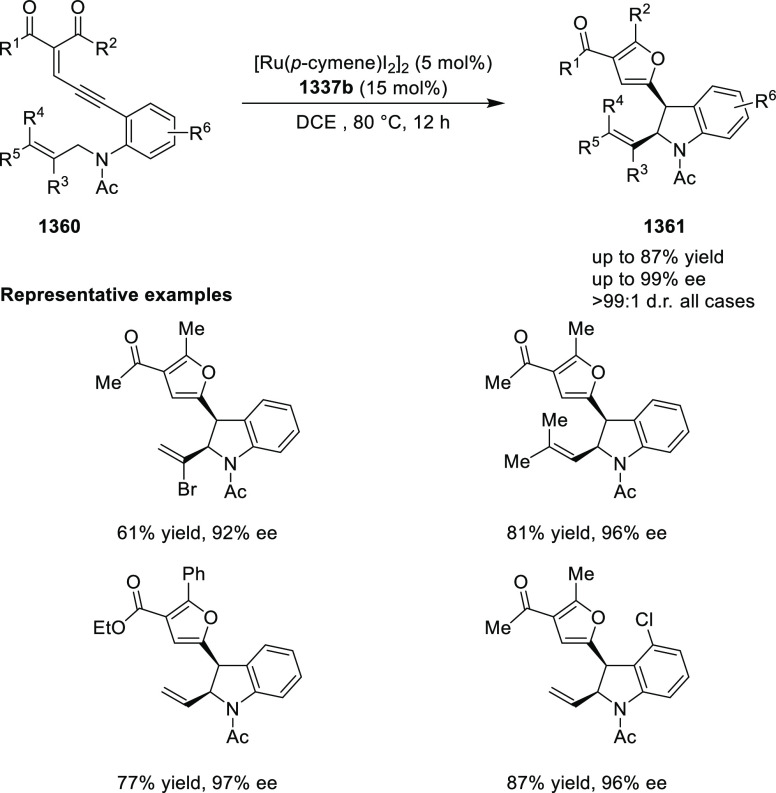
Enantioselective Intramolecular Allylic C–H Insertion Reaction

Zhu has reported a highly enantioselective intramolecular allylic C–H insertion reaction. Using indanyl PyBOX ligand (*R*,*S*)-**1337** in this Ru-catalyzed process, a variety of *N*-allylic enynones **1360** were transformed into di-*syn*-substituted indolines **1361** in just one step, giving the products in up to 87% yield, with excellent enantioselectivities up to >99% *ee*, and in all cases with diastereoselectivities of >99:1 dr (Scheme 403).^481^

**Scheme 404:**
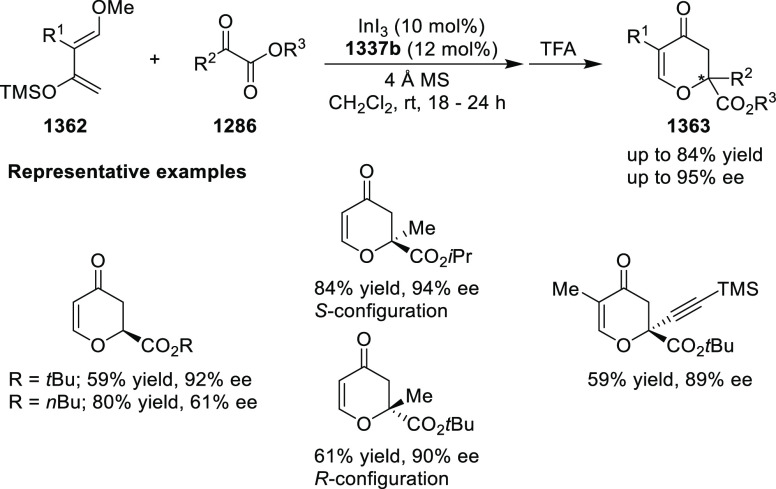
In-Catalyzed HDA Reaction of Danishefsky-type Dienes with α-Carbonyl Esters

Loh has applied (*R*,*S*)-**1337** in an In-catalyzed HDA reaction of Danishefsky-type dienes **1362** with α-carbonyl esters **1286**. A variety of substrates were successfully applied in the reaction to give the corresponding chiral 2,3-dihydro-4-pyranones **1363** in high yields up to 84% and enantioselectivities up to 95% *ee* (Scheme 404). Products containing both tertiary and quaternary stereocenters could be isolated with similarly good results.^482^

**Scheme 405:**
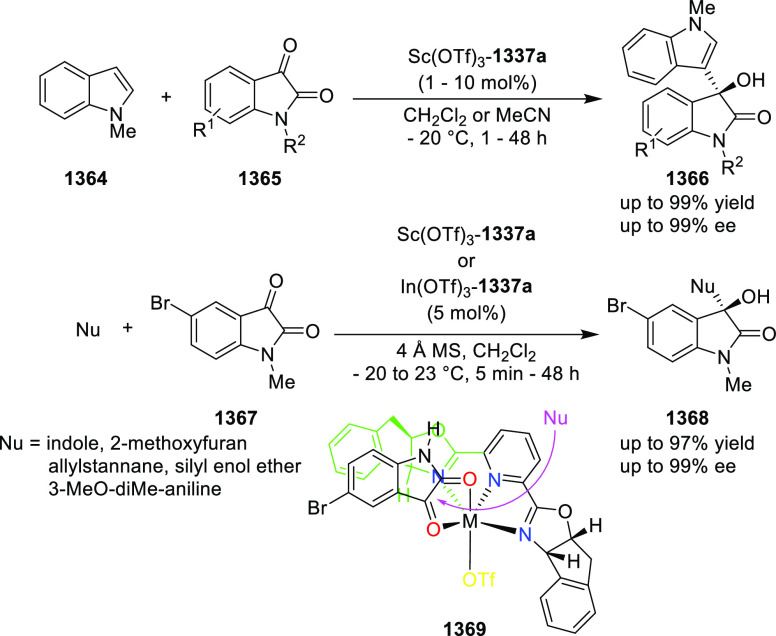
Asymmetric Synthesis of 3-Hydroxy-2-oxindoles

Franz has reported an asymmetric synthesis of 3-hydroxy-2-oxindoles **1366** via the Friedel–Crafts alkylation of indoles **1364** and electron-rich arenes with isatins catalyzed by Sc(III)- and In(III)-(*S,R*)-**1337** complexes. Other PyBOX ligands tested were found to induce only moderate to good enantioselectivities. The product oxindoles **1366** were isolated in up to 99% yield and 99% *ee* for a range of substrates (Scheme 405). The catalysts were also found to promote asymmetric allylation and aldol reactions of isatins in up to 97% yield and 99% *ee*. An octahedral model **1369** for this process is invoked to explain the sense of stereoinduction. If the isatin amide carbonyl group is bound to the apical position of the complex, the nucleophile is forced to attack the *Si*-face. This study into the activity of both Sc(III) and In(III) complexes provides a useful guide for further research in this area.^483^ Franz has extended this work to include *N*-methyl-pyrrole^484^ and allylsilanes^485^ as nucleophiles.

**Scheme 406:**
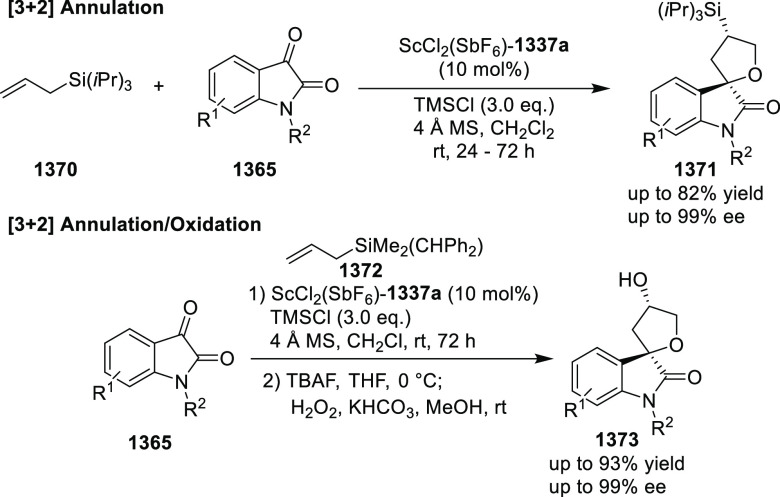
Asymmetric [3 + 2] Annulation of Isatins with Allyl Silanes

Franz has described another extension of this work wherein allyl silanes based on a bulky −Si(*i*Pr)_3_ moiety is used as nucleophiles in an asymmetric [3 + 2] annulation with isatins. Various isatin derivatives **1365** react with allylsilanes **1370**, which bear a bulky silyl-group, in an ScCl_2_(SbF_6_)-(*S,R*)-**1337**-catalyzed nucleophilic addition/1,2-silyl migration/annulation to give a range of spirooxindoles **1371** in up to 82% yield and 99% *ee* (Scheme 406). Subjecting allyl silane **1372**, bearing an −SiMe_2_(CHPh_2_) group, to the annulation reaction conditions followed by C–Si oxidation affords the corresponding secondary alcohols **1373** in excellent yields and enantioselectivities.^486^

**Scheme 407:**
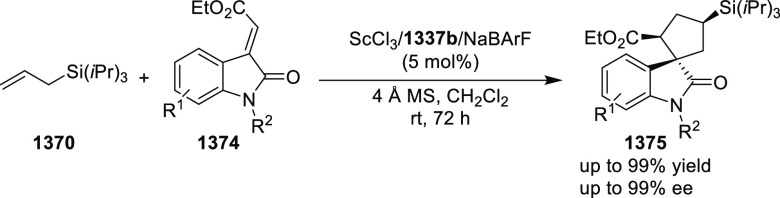
Asymmetric [3 + 2] Annulation of Alkylidene Oxindoles with Allyl Silanes

Franz has extended this carboannulation methodology to include alkylidene oxindoles **1374** as electrophiles, utilizing a ScCl_3_/(*R*,*S*)-**1337**/NaBARF catalytic system, to give the corresponding spirooxindoles **1375** in up to 99% yield and 99% *ee* (Scheme 407).^487^

**Scheme 408:**
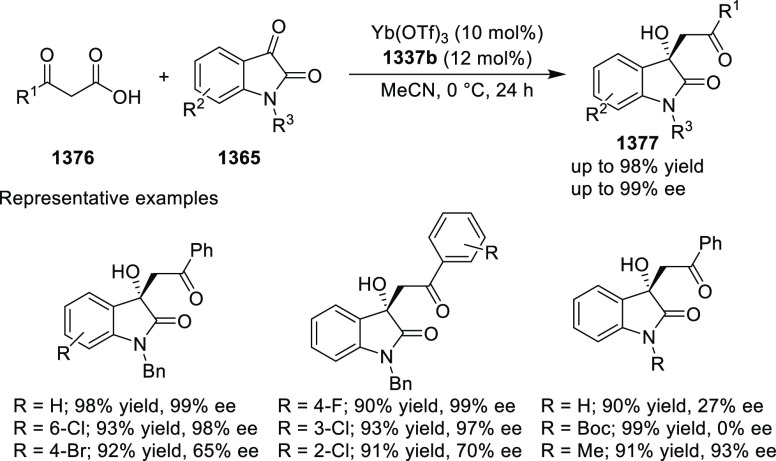
Yb-Catalyzed Decarboxylative Asymmetric Addition of β-Ketoacidsto Isatins

Wang has described a related Yb-catalyzed decarboxylative asymmetric addition of β-ketoacids **1376** to isatins **1365**, employing chiral PyBOX ligand (*R*,*S*)-**1337**. A range of 3-hydroxy-oxindoles **1377** were synthesized in up to 98% yield and 99% *ee* (Scheme 408). The same stereochemical model is used to explain the outcome of this reaction as in Franz’s previous example, with the opposite enantiomer of the ligand giving *Re*-face attack in this instance.^488^

**Scheme 409:**
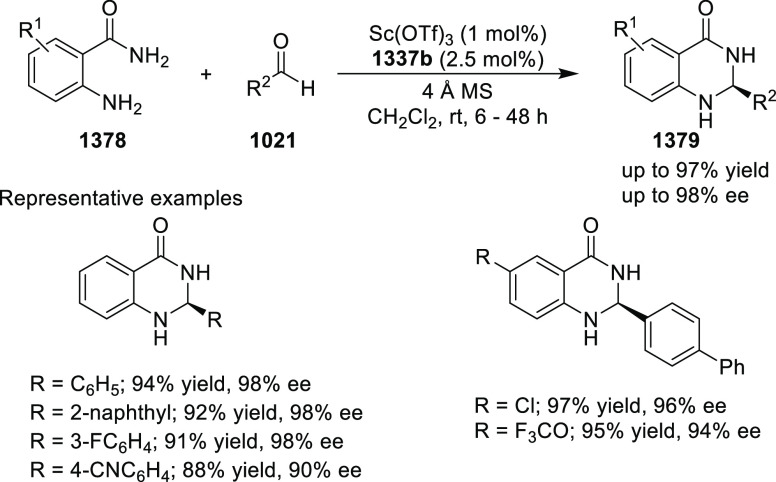
Enantioselective Sc-Catalyzed Intramolecular Amidation of Imines

Kesavan has reported a highly enantioselective Sc(OTf)_3_-(*R*,*S*)-**1337**-catalyzed intramolecular amidation of imines for the synthesis of a range of 2,3-dihydroquinazolinones **1379** in high yields of up to 97% and excellent enantiomeric excesses of up to 98% (Scheme 409). Aryl aldehydes **1021** were reacted with *ortho*-amide-substituted aniline derivatives **1378** with high to excellent enantioselectivities in all reported cases.^489^

**Scheme 410:**
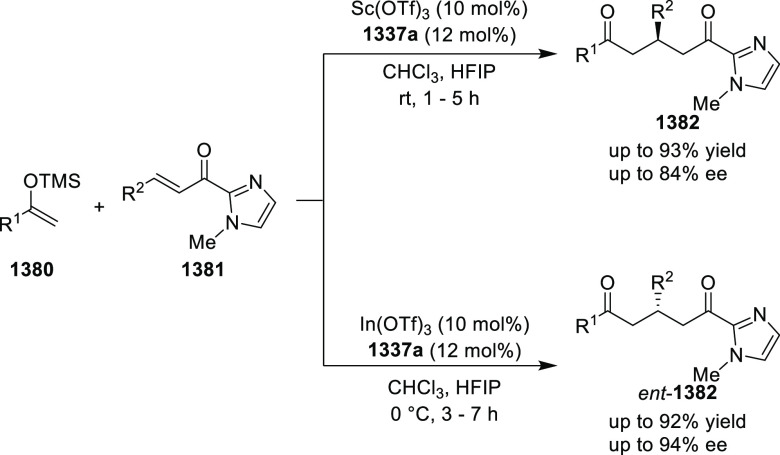
Enantiodivergent Sc(III)- and In(III)-Catalyzed Mukaiyama–Michael Addition of Silyl Enol Ethers

Singh has reported a Sc(III)- and In(III)-catalyzed Mukaiyama–Michael addition of silyl enol ethers **1380** to α,β-unsaturated 2-acyl imidazoles **1381**. Utilizing indanyl PyBOX ligand (*S,R*)-**1337**, the enantioselectivity of the process could be switched by using either Sc(OTf)_3_-(*S,R*)-**1337** or In(OTf)_3_-(*S,R*)-**1337** as the catalyst. In the reaction catalyzed by Sc(OTf)_3_-(*S,R*)-**1337**, a range of aryl-substituted silyl enol ethers **1380** were tolerated, as were a range of aryl groups on the Michael acceptors **1381**, to yield the enantioenriched 1,5-carbonyl products **1382** in up to 93% yield and with up to 84% *ee* (Scheme 410). The reaction catalyzed by In(OTf)_3_-(*S,R*)-**1337** gave similar, but opposite results, leading to the formation of the products *ent*-**1382** in up to 92% yield and with higher levels of enantioselectivity of up to 94% *ee*. The larger ionic radius of In(III), which leads to the formation of a bipyramidal trigonal complex in the key intermediate, as compared to Sc(III), which leads to the formation of an octahedral complex intermediate, was presented as an explanation for the observed switch in enantioselectivity.^490^ Singh has also employed (*S,R*)-**1337** in the Sc(III)- and Er(III)-catalyzed Mukaiyama–Michael addition of siloxyfurans to the same α,β-unsaturated 2-acyl imidazoles.^491^

**Scheme 411:**
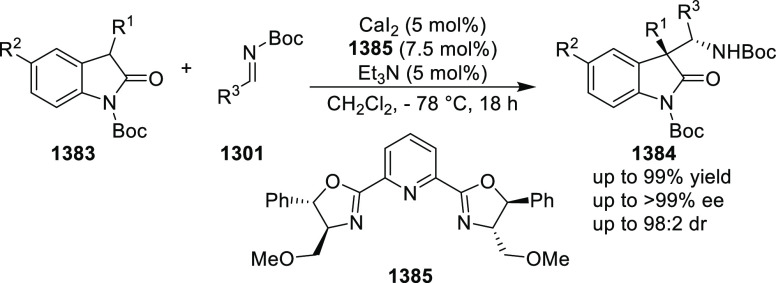
Asymmetric Ca-Catalyzed Reaction of Oxindoles with *N*-Boc Imines

**Figure 67:**
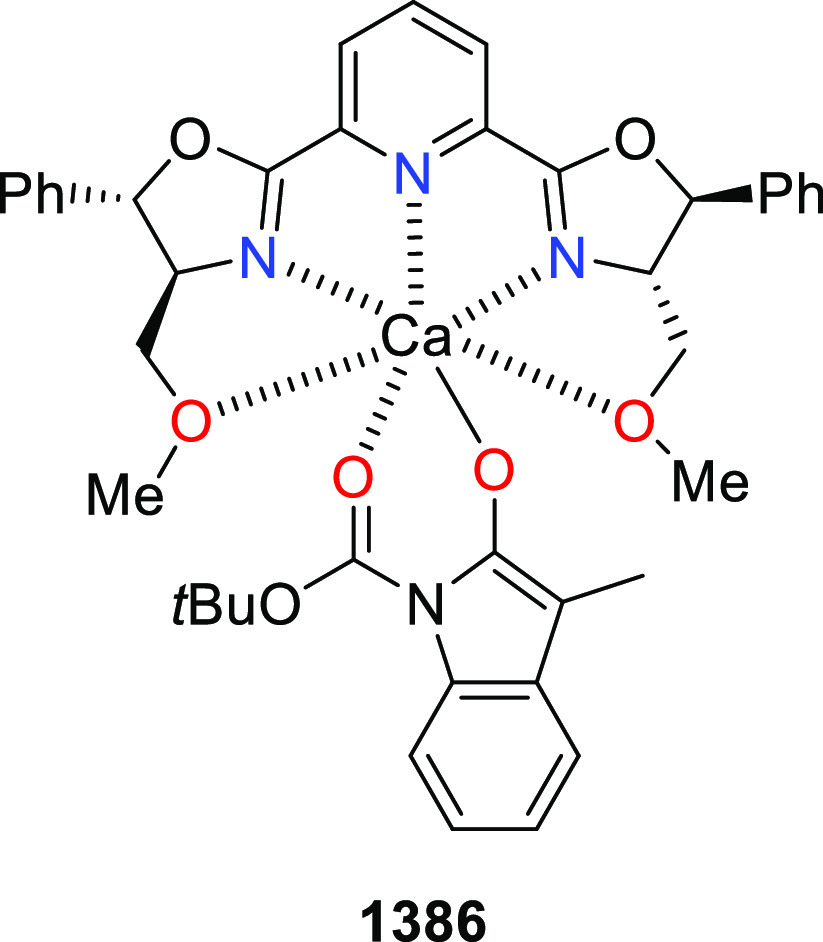
Stereochemical model for the asymmetric Ca-catalyzed reaction of oxindoles with *N*-Boc imines.

Kobayashi has described a Ca-catalyzed reaction of oxindoles **1383** with *N*-Boc imines **1301** for the asymmetric synthesis of 3-tetrasubstituted oxindoles **1384**. Utilizing chiral PyBOX ligand **1385**, a range of oxindoles **1383** and imines **1301** were successfully subjected to the reaction to give the corresponding chiral amine products **1384** in up to 99% yield, >99% *ee*, and 98:2 *anti*/*syn* dr (Scheme 411). A DFT study of the CaCl_2_-**1385** complex and the corresponding oxindole enolate complex suggested the formation of a *C*_2_-symmetric complex with all three nitrogen atoms coordinated to the metal center. The distance between the Ca and O (of the methoxy groups) suggested a weak coordination of O to Ca, and a stereochemical model **1386** in which the −CH_2_OMe moiety of the ligand shields the *Re*-face of the enolate was proposed (Figure 67).^492^

**Figure 68:**
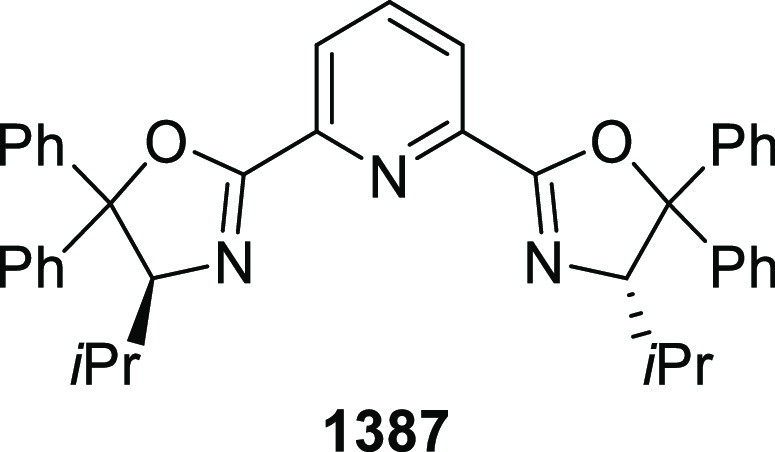
PyBOX ligand **1387**.

Singh has reported the use of *gem*-diPh-*i*Pr PyBOX ligand **1387** in several enantioselective catalytic transformations (Figure 68).

**Scheme 412:**
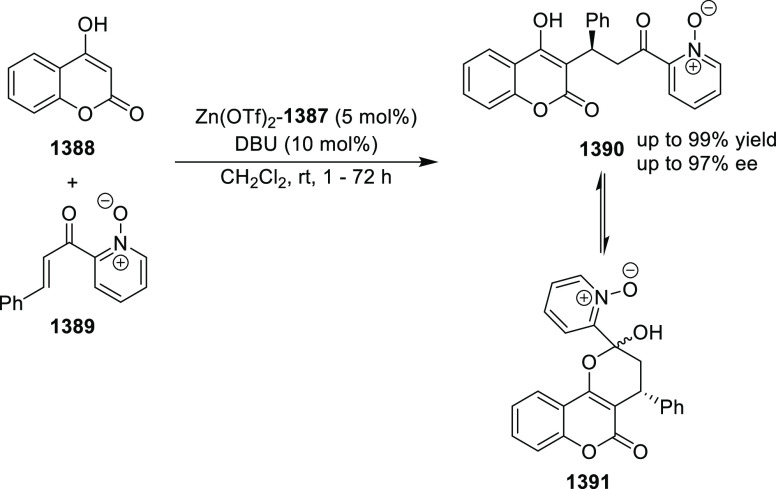
Asymmetric Synthesis of Coumarin Derivatives

They reported an asymmetric synthesis of coumarin derivatives **1390***via* a Zn(II)-**1387**-catalyzed Michael addition of 4-hydroxycoumarin **1388** with 2-enolpyridine *N*-oxides **1389** in up to 99% yield and 97% *ee* (Scheme 412). PyBOX ligands without *gem*-disubstitution did not perform well in the transformation. The coumarin products **1390** were found to be in equilibrium with both diastereomeric forms of cyclized **1391**. Washing the products of the reaction with ethyl acetate significantly increased their enantiomeric purity up to >99.9% *ee*.^493^ The scope of this reaction was further extended to include aliphatic cyclic 1,3-diketones^494^ and silyl enol ethers^495^ as the nucleophiles with similar results.

**Scheme 413:**
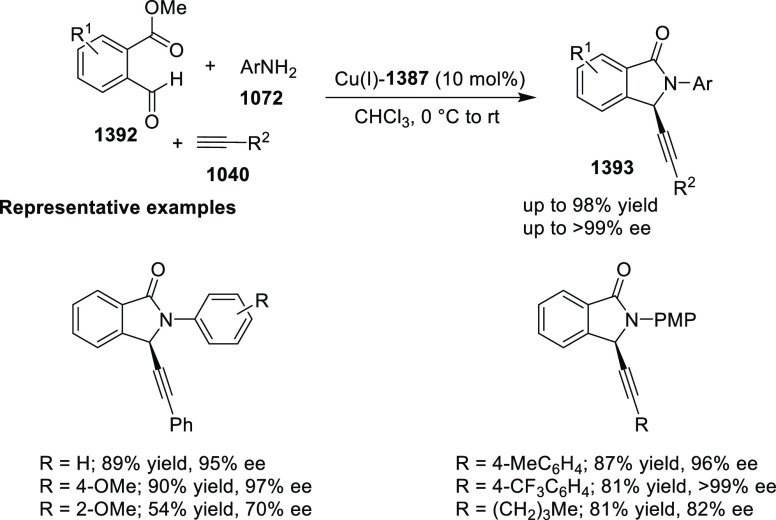
Cu(I)-Catalyzed Asymmetric Alkynylation/Lactamization

Singh also reported a Cu(I)-**1387**-catalyzed asymmetric alkynylation/lactamization cascade for the synthesis of chiral isoindolinones **1393**. The three component reaction of various methyl-2-formylbenzoate derivatives **1392** with aryl amines **1072** and alkynes **1040** proceeds through an A^3^-coupling to give, following intramolecular lactamization of the intermediate chiral amine, the corresponding isoindolinones **1393** in high yields of up to 98% and enantiomeric excesses of up to >99% (Scheme 413).^496^ Singh further employed this methodology in the asymmetric synthesis of medicinally relevant target compounds.^497^

**Scheme 414:**
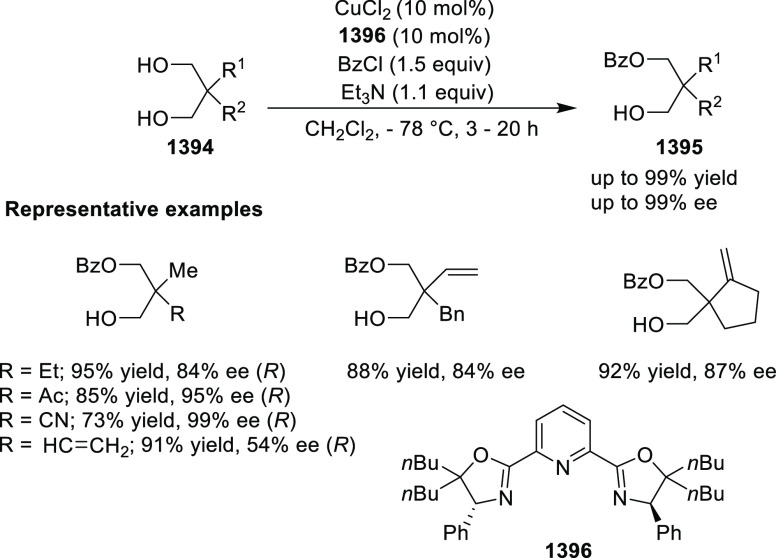
Desymmetrization of 2,2-Disubstituted 1,3-Propanediols

**Figure 69:**
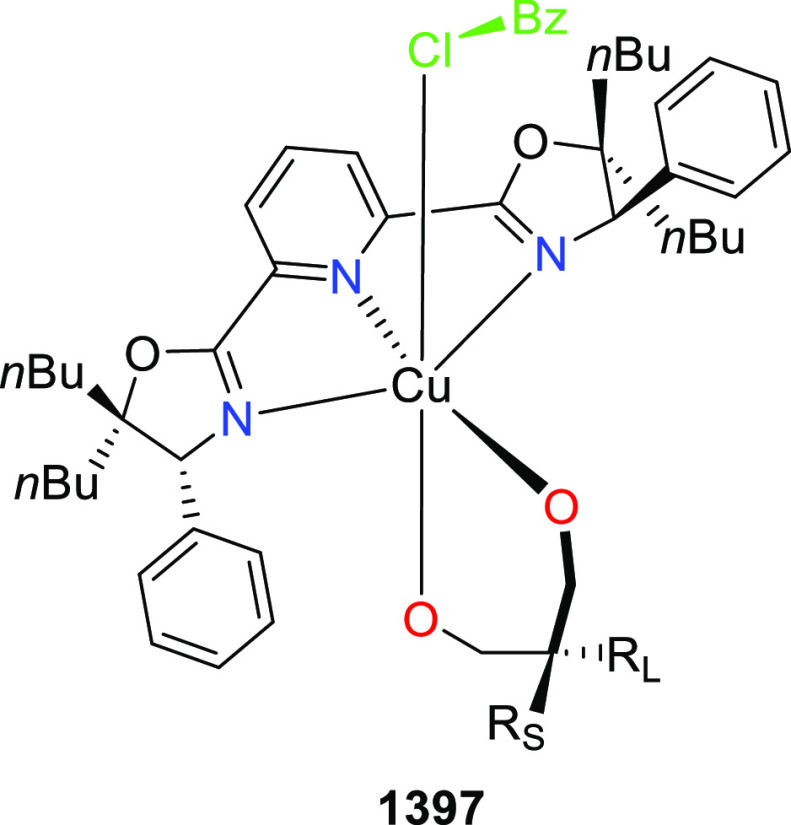
Stereochemical model for the desymmetrization of 2,2-disubstituted 1,3-propanediols.

Kang has reported a desymmetrization reaction of 2,2-disubstituted 1,3-propanediols for the synthesis of all-carbon quaternary stereocenters. Utilizing *gem*-dibutyl CuCl_2_-**1396**, an asymmetric benzoylation of one of the two alcohols was achieved by reacting various 1,3-propanediols **1394** with benzoyl chloride under basic conditions to give the desymmetrized products **1395** in up to 99% yield and 99% *ee* (Scheme 414). A stereochemical model **1397** was proposed to explain the asymmetric induction in the reaction (Figure 69). If the tridentate chiral PyBOX ligand sits equatorial in the octahedral complex, the benzoyl chloride axial and the diol axial and equatorial, the smaller R-group on the diol is most likely to occupy the space near the 4-Ph group of the oxazoline ring. The benzoyl cation is then attacked by the equatorial alcohol group to give the desymmetrized products. As a result, substrates with lower levels of steric differentiation between the two R-groups gave lower enantioselectivities, for example in the case of R^1^ = Me, R^2^ = −CH=CH_2_, the product was obtained in only 54% *ee*.^498^

Overall, PyBOX ligands with disubstituted oxazoline rings appear to be more useful in asymmetric lanthanide-metal-catalyzed processes than their monosubstituted counterparts. They have also been applied in a range of Sc- and In-catalyzed processes.

#### 3.2.14 Bis(oxazoline) Ligands with Modified Pyridine Linkers

**Figure 70:**
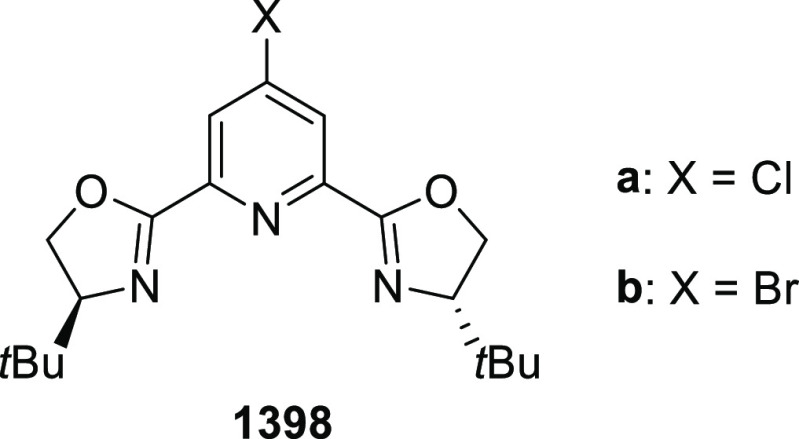
4-Halogenated PyBOX ligands.

Less common modifications to the chiral PyBOX ligand structure are pyridine ring-based modifications. Johnson has applied 4-halogenated chiral *t*Bu-PyBOX ligands **1398a**–**b** in dynamic kinetic asymmetric reactions of strained cyclopropane rings (Figure 70).

**Scheme 415:**
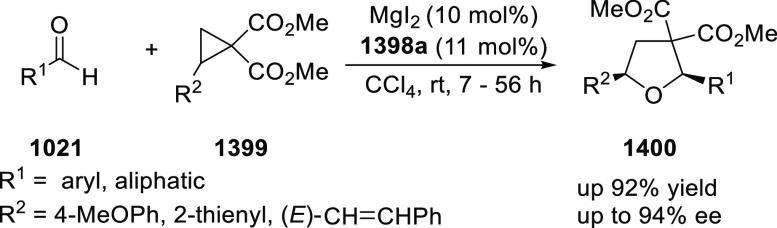
Dynamic Kinetic Asymmetric [3 + 2] Cycloaddition of Racemic Cyclopropanes

For example, they applied chlorinated chiral PyBOX ligand **1398a** in a dynamic kinetic asymmetric [3 + 2] cycloaddition of racemic cyclopropanes **1399** bearing electron-rich donor groups, such as *p*-methoxybenzene, and aldehydes **1021**. A range of chiral tetrahydrofurans **1400** were synthesized in the MgI_2_-catalyzed process in up to 92% yield and 94% *ee* (Scheme 415). A range of alkyl and aryl aldehydes **1021** performed well in the reaction, with the lowest enantiomeric excess obtained for the *iso*-propyl aldehyde of 82%. A range of ligands with pyridine ring substitution were tested, with the halogenated substrates giving the best results. For comparison, the halogenated *t*Bu-PyBOX ligand **1213c** gave the product in 62% yield and 91% *ee*, compared to 74% yield and 92% *ee* with ligand **1398a** for the same substrate.^499^

**Scheme 416:**
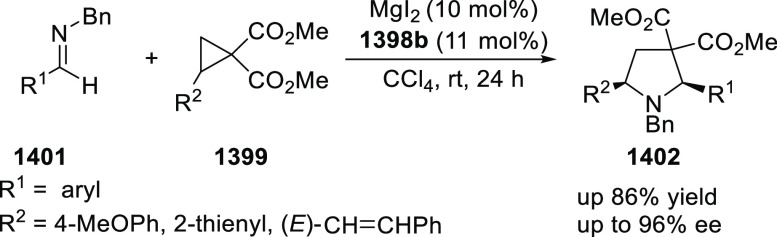
Dynamic Kinetic [3 + 2] Annulation to *N*-Benzyl-(*E*)-aldimine with Cyclopropanes

Johnson subsequently extended the dynamic kinetic [3 + 2] annulation to *N*-benzyl-(*E*)-aldimines **1401** utilizing brominated chiral PyBOX ligand **1398b** under similar conditions to the previous reaction. Chiral 2,5-*cis*-pyrrolidines **1402** were synthesized from cyclopropanes **1399** bearing electron-rich donor groups and aryl aldimines **1401** in up to 86% yield and 96% *ee* (Scheme 416). Mechanistic studies suggest that the aldimine reacts through the (*E*)-isomer, and that isomerization to the (*Z*)-isomer is not a pathway that furnishes the major 2,5-*cis*-disubstituted products.^500^

**Scheme 417:**
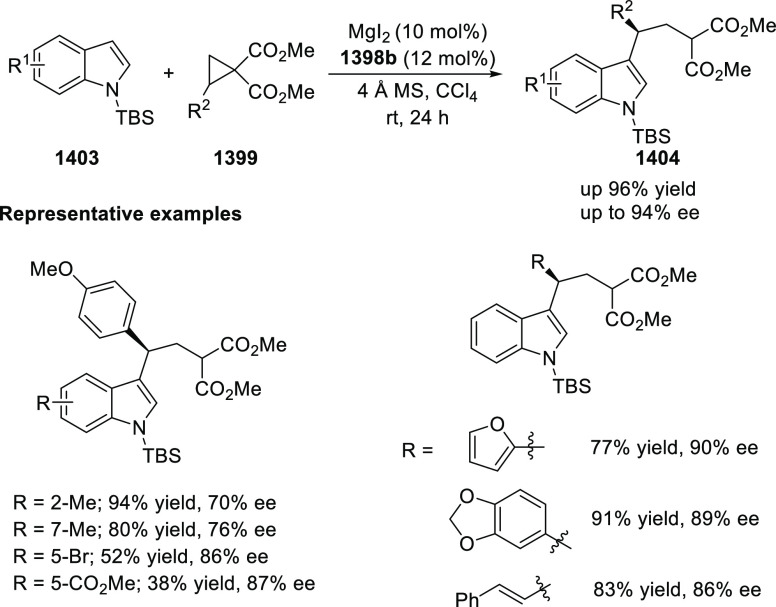
Mg-Catalyzed Dynamic Kinetic Friedel–Crafts Alkylation of Indoles with Cyclopropanes

Johnson has further described a MgI_2_-catalyzed dynamic kinetic Friedel–Crafts alkylation of indoles with cyclopropanes for the asymmetric synthesis of 3-substituted indoles. A range of *N*-TBS protected indoles **1403** were reacted with racemic substituted cyclopropanes **1399** in the presence of Br-modified chiral PyBOX ligand **1398b** to give the chiral 3-substituted indoles **1404** in up to 96% yield and 94% *ee* (Scheme 417).^501^

**Scheme 418:**
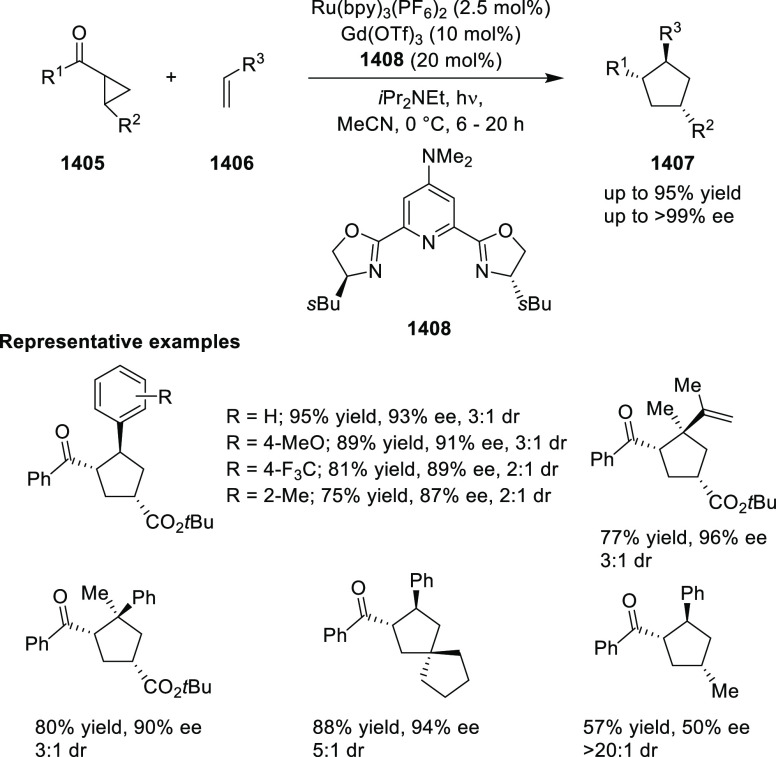
Asymmetric Photocatalytic [3 + 2] Cycloaddition of Aryl Cyclopropyl Ketones with Alkenes

Yoon has applied dimethylamino-substituted *s*-Bu-PyBOX ligand **1408** in the asymmetric photocatalytic [3 + 2] cycloaddition of aryl cyclopropyl ketones **1405** with alkenes **1406** for the synthesis of enantioenriched cylopentanes **1407**. Employing Gd(OTf)_3_ as the Lewis acid in this process, a range of substituted and spirocyclic cyclopentanes **1407** were accessed in up to 95% yield and with up to >99% *ee* (Scheme 418). Generally, the diastereoselectivity of the reaction was relatively low, giving the products in 2:1 to 5:1 dr, with two examples >20:1 dr^502^

**Scheme 419:**
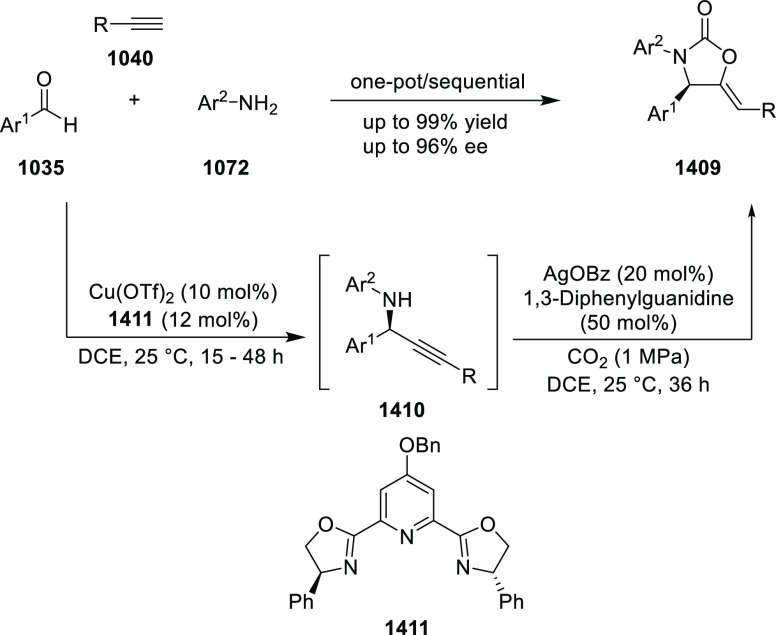
One-Pot/Sequential Tandem Asymmetric A^3^-Coupling/Carboxylation/Cyclization

Zhou has reported a one-pot/sequential tandem asymmetric A^3^-coupling/carboxylation/cyclization for the enantioselective synthesis of 2-oxazolidinones **1409**. Employing the novel −OBn substituted Ph-PyBOX **1411**, aryl aldehydes **1035**, mostly aryl alkynes **1040** and aniline derivatives **1072** were successfully reacted in the Cu-catalyzed A^3^-coupling/Ag-catalyzed carboxylation sequence to access a range of chiral 2-oxazolidinones **1409** in up to 99% yield and with excellent enantioselectivities up to 96% *ee* (all examples reported were >90% *ee*) (Scheme 419). An interesting ligand-accelerating effect was found for the cyclization step. The cyclization occurs without the presence of the ligand **1411** or Cu(OTf)_2_, but the rate of the reaction and subsequent yield of the isolated product was found to increase dramatically when the ligand was present, and more so when Cu(OTf)_2_ was also present. More interesting still, the presence of excess aniline was found to increase the rate of the cyclization step. Thus, the leftover reagents from the first step were found to promote the upstream cyclization.^503^

**Scheme 420:**
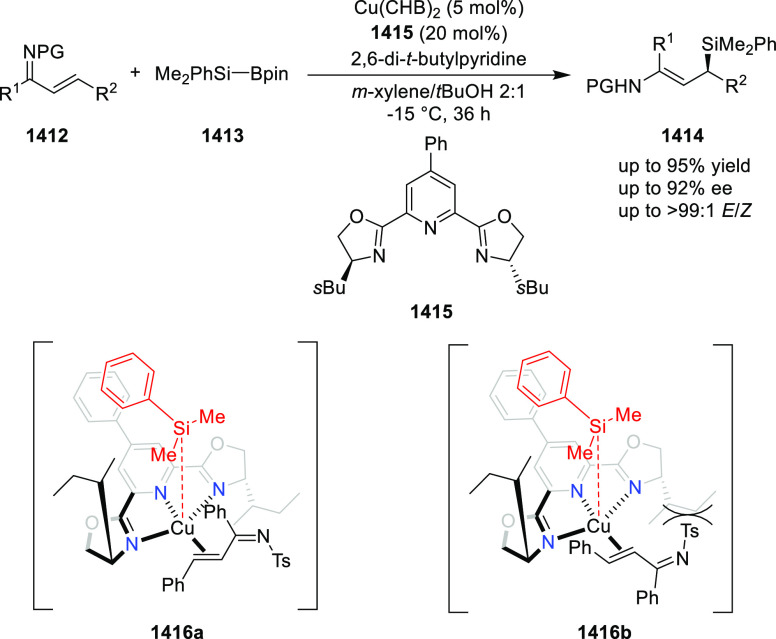
Cu-Catalyzed Enantioselective 1,4-Protosilylation of α,β-Unsaturated Ketimines

Loh and Xu have utilized the Ph-derivative of the *s*-Bu-PyBOX ligand **1415** in a Cu-catalyzed enantioselective 1,4-protosilylation of α,β-unsaturated ketimines **1412**. A range of aryl-substituted ketimines **1412**, bearing a variety of protecting groups on *N*, were successfully employed in the reaction for the synthesis of (*E*)-allyl silanes **1414** in up to 95% yield, with up to 92% *ee* and excellent *E*/*Z* selectivity of up to >99:1 (Scheme 420). The choice of chiral ligand, bearing Ph-substitution on the pyridine ring, was based on the formation of a favorable π—π interaction between the Si-Ph moiety and the pyridine-Ph moiety in the transition state. The enantioselectivity is rationalized by the disfavored steric interaction between the *N*-PG of the substrate with one of the *s*-Bu groups of the ligand in **1416b**, forcing the ketimine to approach as in **1416a** with the *N*-PG pointing away, for selective delivery of the silyl group.^504^

Jayashankaran has also employed the novel 4-substituted pyridine ring-modified chiral PyBOX ligands in asymmetric catalysis. A range of modified *i*Pr-PyBOX ligands of the type **1419** bearing a substituted aryl ring in the 4-position of the pyridine ring were screened in the Rh-catalyzed hydrosilylation of aromatic ketones with Ph_2_SiH. Acetophenone **1417** was chosen as a model substrate and subjected to the reaction with various preprepared Rh-catalysts [RhCl_3_(**1419a**–**f**)] (Table 13). While all the ligands induced good levels of enantiocontrol, ligand **1419d** bearing a 4-EtC_6_H_4_ group at the 4-position of the pyridine ring gave the best result, yielding the alcohol **1418** in 94% yield and 98% *ee* (entry 4). A variety of aromatic ketones were subjected to the hydrosilylation using [RhCl_3_(**1419d**)] as the catalyst to afford the corresponding alcohols in up to 94% yield and 99% *ee*.^505^

**Table 13:**
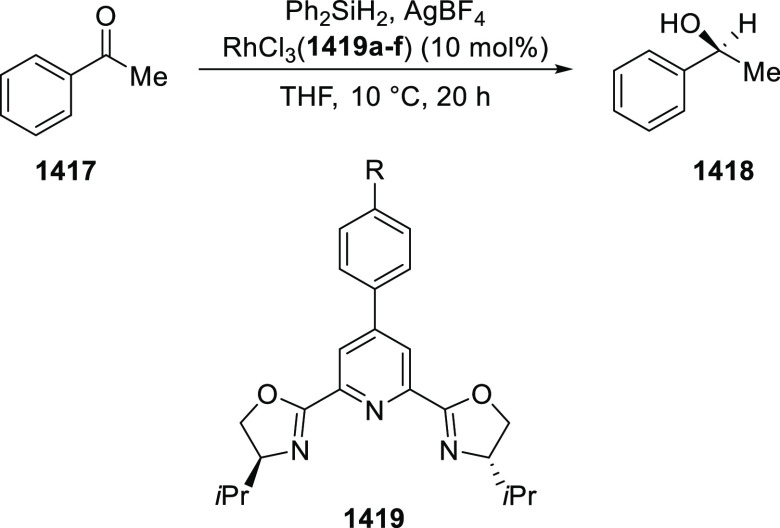
Rh-Catalyzed Hydrosilylation of Aromatic Ketones with Ph_2_SiH

entry	**1419**	R	yield (%)	% *ee*
1	a	H	88	90
2	b	OMe	90	78
3	c	CN	96	75
4	d	Et	94	98
5	e	Cl	89	84
6	f	CO_2_Et	78	73

The modification of the pyridine ring of the PyBOX scaffold offers an opportunity to alter the electronic properties of these ligands. It is clear from the reports described in this Review that this can be a successful strategy for increasing the levels of stereoinduction in a particular transformation, and this should be considered by researchers in the future.

#### 3.2.15 Bis(oxazoline) Ligands with Phenyl Linkers

**Figure 71:**
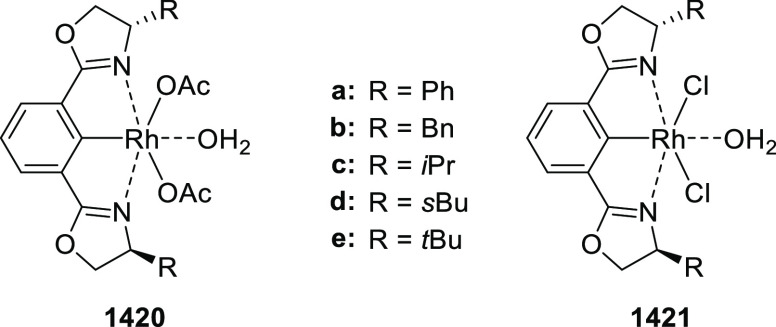
Rh(PheBOX) complexes.

Nishiyama has pioneered the development of *N*,*C*,*N*-tridentate bis(oxazoline) ligands linked by a phenyl-anion unit (PheBOX).^506^ These ligands form strong C–M (M = metal) covalent bonds that stabilize the resulting chiral ligand–metal complexes. The most widely applied PheBOX-complexes in asymmetric catalysis are the Rh-complexes like **1420** and **1421**, although other metals have also been used like Ru, Pt, Pd, and Ni (Figure 71).

**Scheme 421:**
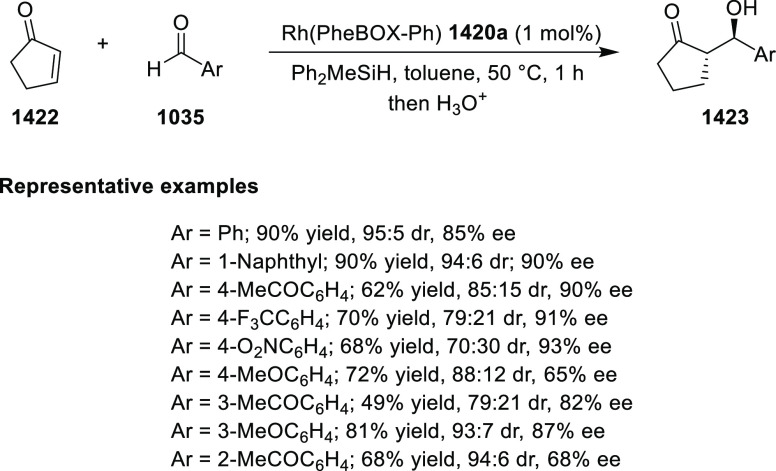
Diastereo- and Enantioselective Rh-Catalyzed Reductive Coupling of Cyclopentenone with Aromatic Aldehydes

While a range of PheBOX analogues with different phenyl- and oxazoline-ring substitution patterns have been developed, the standard PheBOX metal complexes are more abundantly reported. For example, Nishiyama has developed a diastereo- and enantioselective **1420a**-catalyzed reductive coupling of cyclopentenone **1422** with aromatic aldehydes **1035** to yield a range of *anti*-alcohols **1423** in up to 90% yield, with up to 95:5 dr and 93% *ee*. The outcome of the reaction was sensitive to substitution on the aryl ring of the aldehyde **1035**, for example, *p*-anisaldehyde gave the alcohol in only 65% *ee* (Scheme 421).^507^

**Figure 72:**
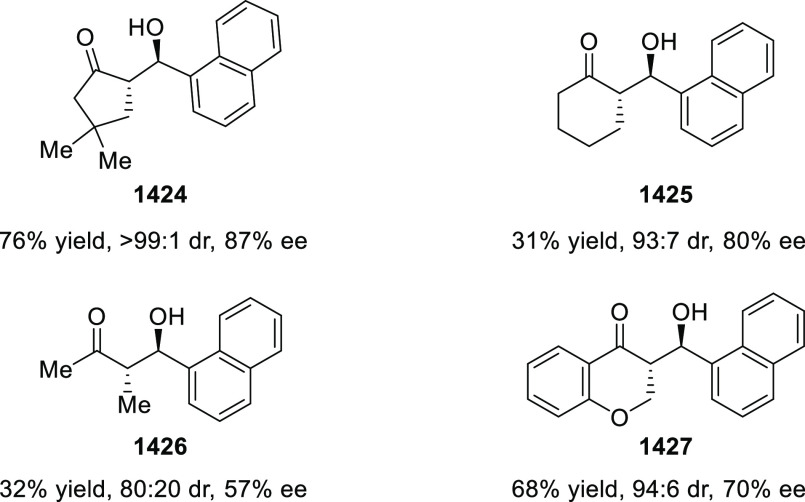
Products of the Rh-catalyzed reductive coupling of various enones with naphthaldehyde.

Some other enones were tested, with cyclic enones giving the corresponding alcohols **1424**–**1427** with better enantioselectivities than the only acyclic example, methylvinyl ketone, which gave the *anti*-alcohol **1426** with 80:20 dr and 57% *ee* (Figure 72).

**Figure 73:**
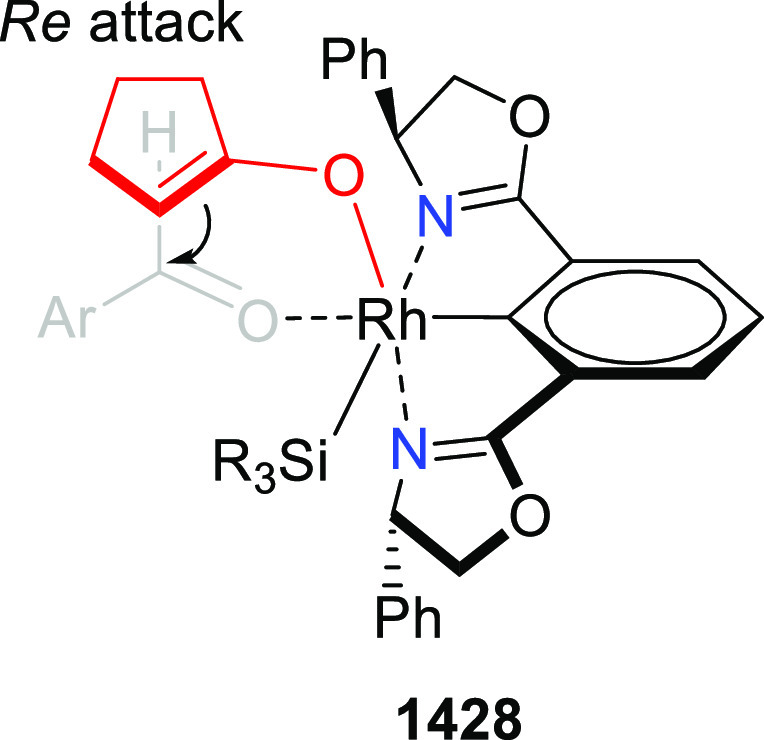
Stereochemical model for the Rh-catalyzed reductive coupling of cyclopentenone with aromatic aldehydes.

The possible transition state **1428** was proposed to explain the stereochemical outcome of the reaction, in which the *Re*-face of the enolate, which forms following hydride addition to the enone, attacks the *Re*-face of the aldehyde to avoid a steric interaction with one of the oxazoline-Ph groups (Figure 73).

**Scheme 422:**
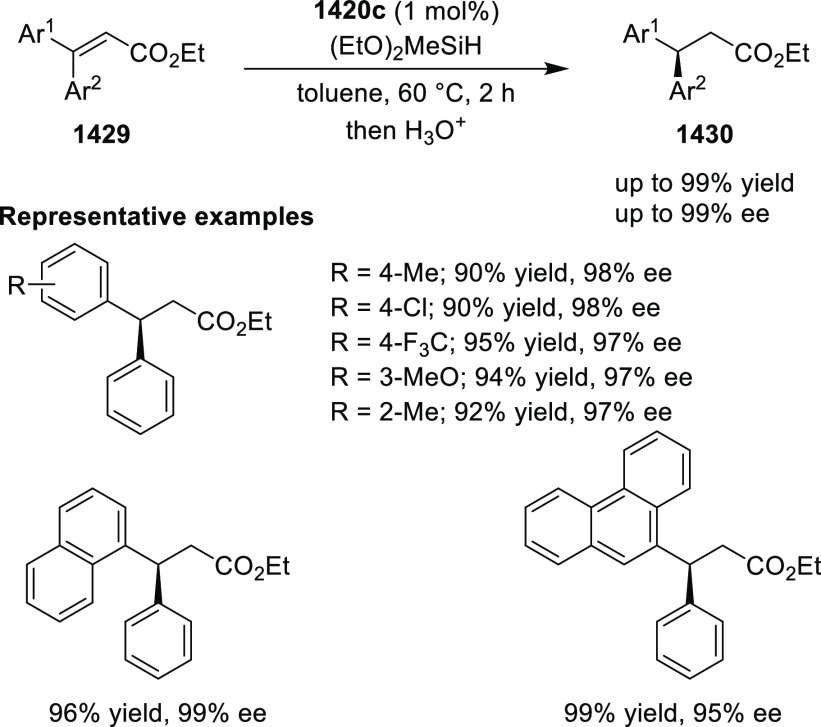
Rh-Catalyzed Asymmetric Hydrosilylation of α,β-Unsaturated Esters

Nishiyama also developed the asymmetric hydrosilylation of α,β-unsaturated esters **1429** catalyzed by *i*Pr-PheBOX-Rh **1420c**. Under optimized conditions, a range of enantioenriched, diaryl propanoates **1430** were accessed in up to 99% yield and with up to 99% *ee* with all reported examples isolated with >95% *ee* (Scheme 422). The reaction of heteroaromatic substrates did not proceed smoothly under the optimized conditions.^508^

**Scheme 423:**
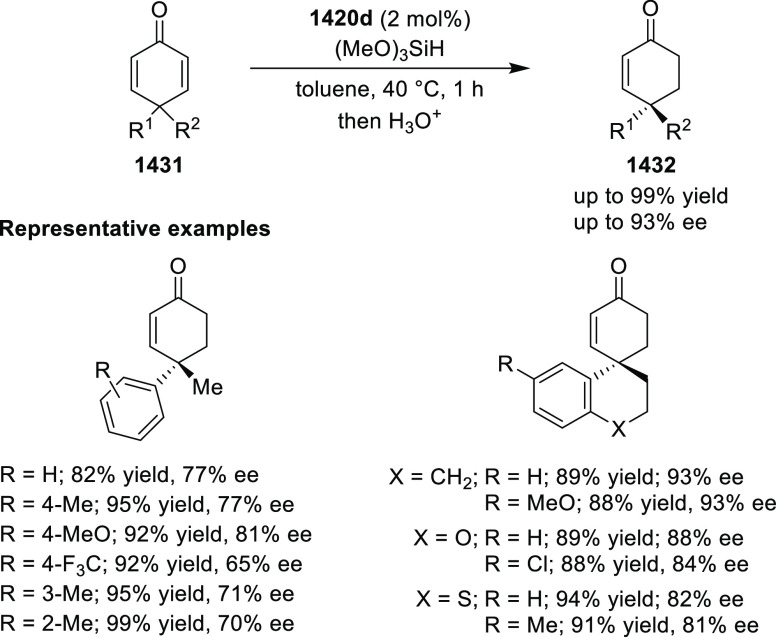
Rh-Catalyzed Asymmetric Hydrosilylation of Cyclohexadienones

Nishiyama has applied *s*Bu-PheBOX-Rh **1420d** in the asymmetric hydrosilylation of cyclohexadienones **1431** with asymmetric induction at remote quaternary centers. A small range of nonspirocyclic products **1432** were accessed in up to 99% yield and with up to 81% *ee*, generally only giving moderate levels of enantioselectivity (<80% *ee*) (Scheme 423). Spirocyclic cyclohexadienones were found to perform better in this process giving the enantioenriched products in up to 99% yield and with 93% *ee* with most examples giving high levels of enantioselectivity >80% *ee*.^509^

**Scheme 424:**
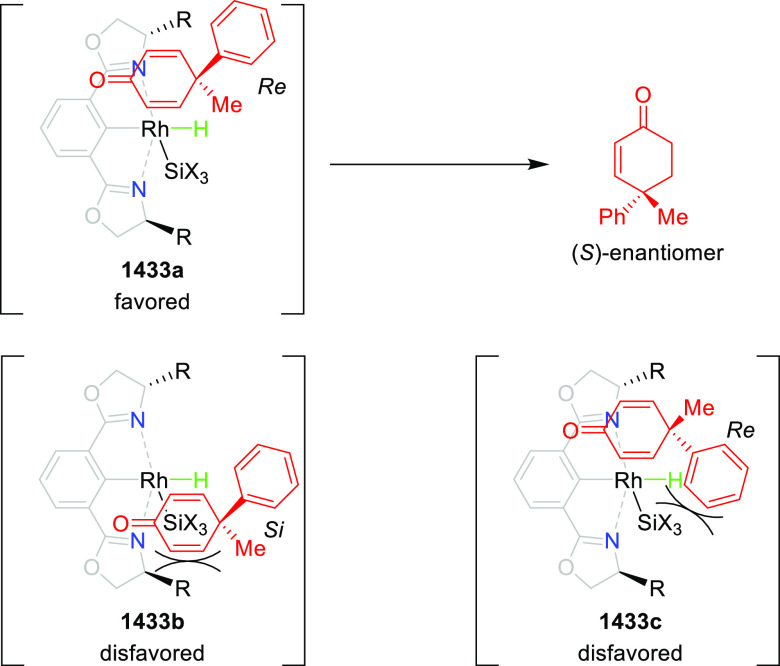
Stereochemical Model for Rh-Catalyzed Asymmetric Hydrosilylation of Cyclohexadienones

A possible transition state **1433a** was proposed to explain the stereochemical outcome of this hydrosilylation, with stereoinduction at a nonreacting quaternary center (Scheme 424). The Rh–H bond sits in the equatorial position with bond formation occurring on the *Re*-face of the enone, due to the steric repulsion of the oxazoline-*s*Bu-group and the cyclohexadienone, as shown in **1433b**. The steric environment of the γ-position (Ph and Me) is differentiated due to steric repulsion of the bulky trimethoxysilyl group with the Ph-group of the cyclohexadienone, as shown in **1433c**.

**Scheme 425:**
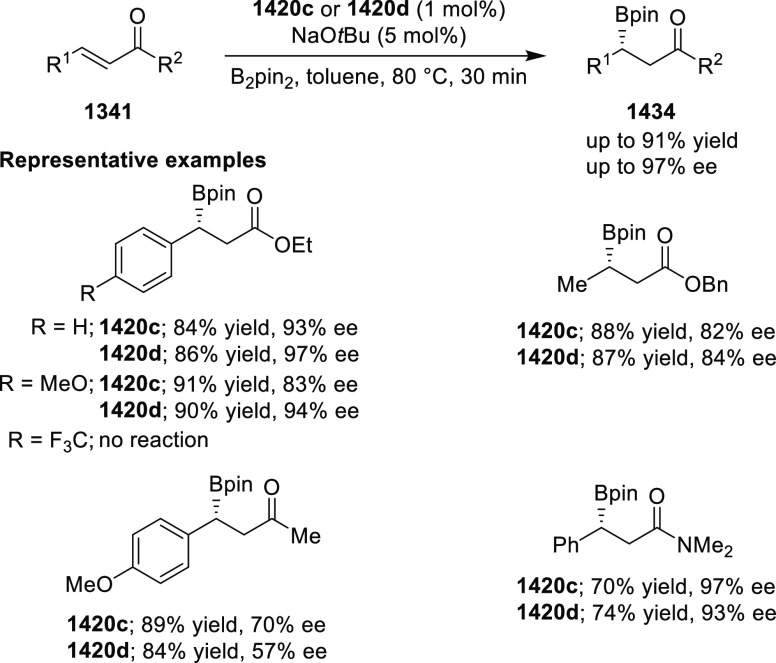
Asymmetric β-Borylation of α,β-Unsaturated Esters, Amides, and Ketones

The asymmetric β-borylation of α,β-unsaturated esters, amides and ketones **1341** with *i*Pr- and *s*Bu-PheBOX-Rh catalysts **1420c** and **1420d** was reported by Nishiyama. Enantioenriched esters **1434** were formed in this transformation with a number of examples isolated in up to 91% yield and with up to 97% *ee* (Scheme 425). Methyl ketones did not perform well, with two examples isolated in up to 89% yield and with up to 70% *ee*. Finally, a single dimethylamide example was isolated in 70% yield and with 97% *ee* (**1420c**) and 74% yield and with 93% *ee* (**1420d**). When the reaction was run with (*Z*)-ethyl cinnamate as the substrate and **1420c** as the catalyst, the borylated product was isolated as the same (*S*)-enantiomer, as in the reaction with (*E*)-ethyl cinnamate (84% yield, 95% *ee*), in 70% yield and with 93% *ee*. While a stereochemical model for this transformation was proposed, it did not take the outcome of the reaction with the (*Z*)-alkene into account.^510^

**Scheme 426:**
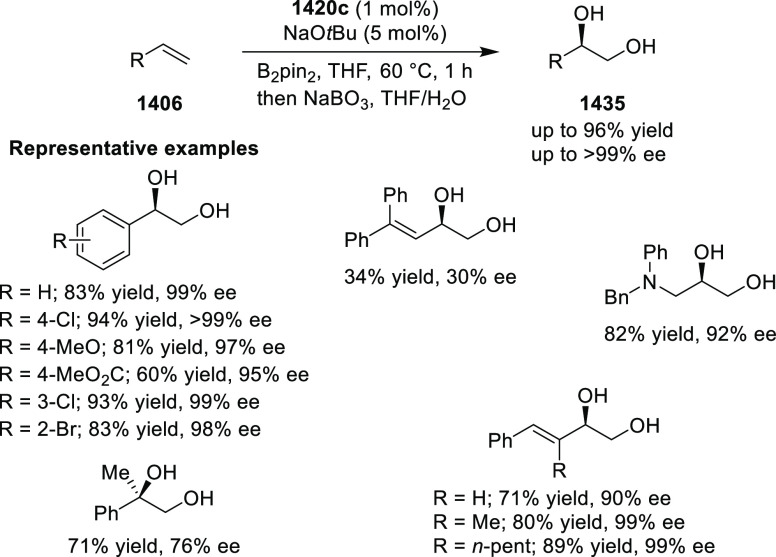
Asymmetric Synthesis of 1,2-Diols

Nishiyama continued to explore asymmetric borylation reactions of alkenes and reported the *i*Pr-PheBOX-Rh **1420c**-catalyzed diboration/oxidation of unactivated terminal alkenes **1406** for the asymmetric synthesis of 1,2-diols **1435**. A wide range of aryl, alkenyl and aliphatic substituted-alkenes **1406** were successfully transformed into enantioenriched 1,2-diols **1435** in up to 96% yield and with up to >99% *ee* (Scheme 426).^511^

**Figure 74:**
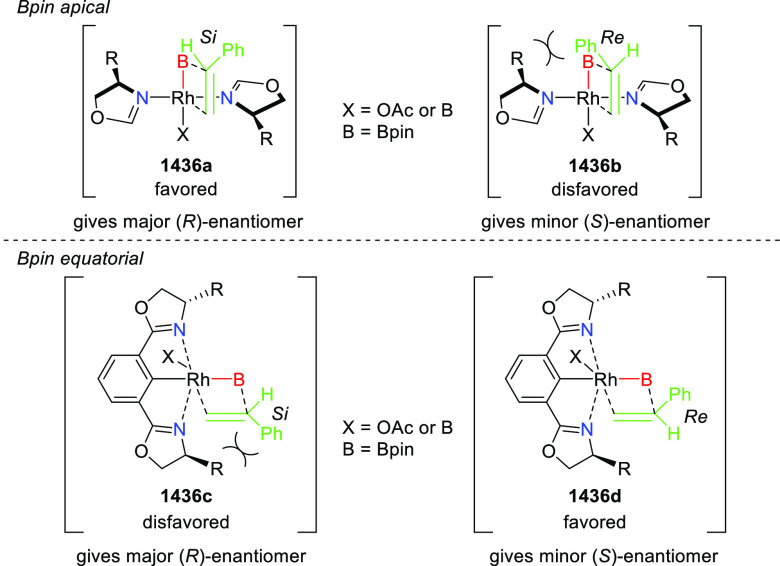
Stereochemical mode for the Rh-catalyzed borylation of alkenes.

A stereochemical model was proposed to explain the formation of the major (*R*)-enantiomer in this transformation. In transition states **1436a** and **1436b**, the Bpin (B) sits in one of the apical positions (Figure 74). In **1436a**, C–B bond formation on the *Si*-face of the alkene gives the major (*R*)-enantiomer of the product, while transition state **1436b**, with C–B bond formation on the *Re*-face of the alkene, is disfavored due to the steric interaction between the Ph-group of the alkene and one of the oxazoline R-groups. Transition states **1436c** and **1436d** show the Bpin in one of the equatorial positions. Transition state **1436c** shows a disfavored steric interaction as in **1436b**. **1436d** has no disfavored interactions, but C–B bond formation on the *Re*-face of the alkene gives rise to the minor (*S*)-enantiomer of product.

**Scheme 427:**
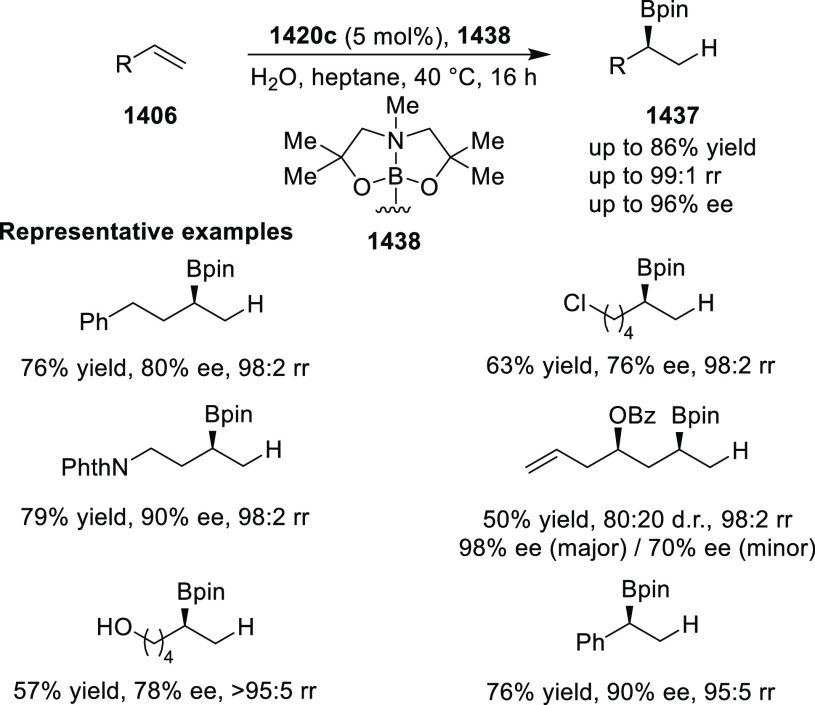
Rh-Catalyzed Markovnikov Hydroboration of Unactivated Terminal Alkenes

Aggarwal built on the work by Nishiyama in the diboration of terminal alkenes and has reported an *i*Pr-PheBOX-Rh **1420c**-catalyzed Markovnikov hydroboration of unactivated terminal alkenes **1406** with a novel borylating reagent **1438** and H_2_O as a proton source. Following optimized conditions, the enantioenriched products **1437** were isolated in moderate to good yields up to 86%, with regioselectivities up to 99:1 rr and enantioselectivities up to 96% *ee* (Scheme 427). Aliphatic and aryl substrates were reported with the process showing good functional group tolerance.^512^

**Figure 75:**
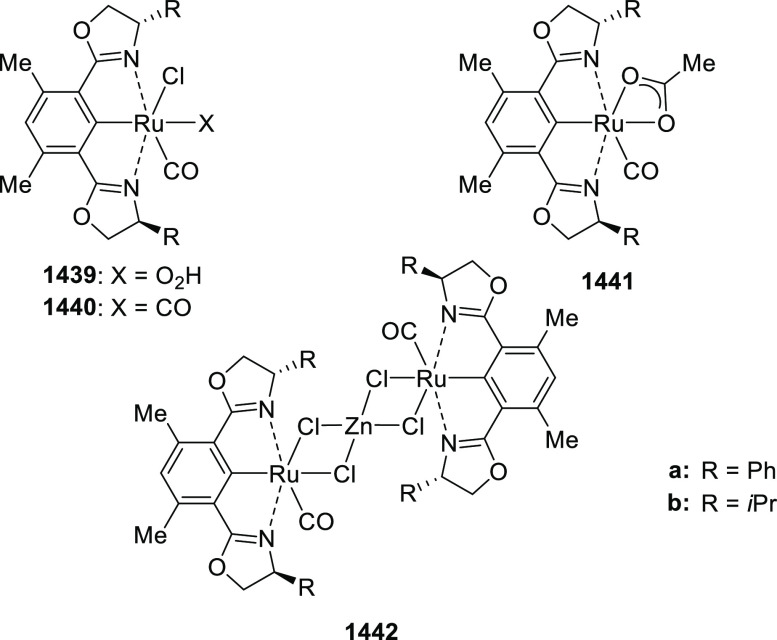
Ru(PheBOX) complexes.

Analogues of the PheBOX ligands have been developed for use in Ru-catalysis. For example, Nishiyama has developed complexes of the type **1439**–**1442**, bearing PheBOX ligands with dimethyl-substituted bridging-phenyl rings to prevent Ru *C*-H insertion at these positions (Figure 75).

**Scheme 428:**
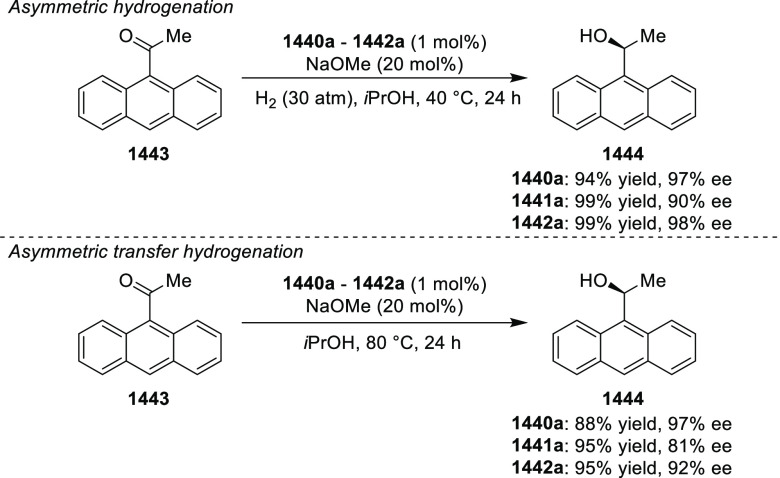
Ru-Catalyzed Asymmetric Hydrogenation and Asymmetric Transfer Hydrogenation of Ketone **1443**

Nishiyama first applied Ru-complexes **1440**–**1442** in the asymmetric hydrogenation of ketones. Reactions with Ru-complexes **1440a** and **1442a** were found to give access to enantioenriched alcohols in high yields up to 99% and with up to 98% *ee* (*S*). For example, ketone **1443** was subjected to the reaction with both complexes to give alcohol **1444** in 94% yield and with 97% *ee* (**1440a**) and in 99% yield and with 98% *ee* (**1442a**) (Scheme 428). These complexes were also applied in enantioselective transfer hydrogenation to give the alcohols with overall lower levels of enantioselectivity. For example, the same ketone **1443** was subjected to the transfer hydrogenation in the presence of both complexes to give the alcohol **1444** in 88% yield and with 97% *ee* (**1440a**) and in 95% yield and with 92% *ee* (**1442a**).^513^ In a report earlier that year, complex **1441a** was applied in the same reactions, giving overall lower levels of enantioselectivity to **1440a** and **1442a**.^514^

**Scheme 429:**
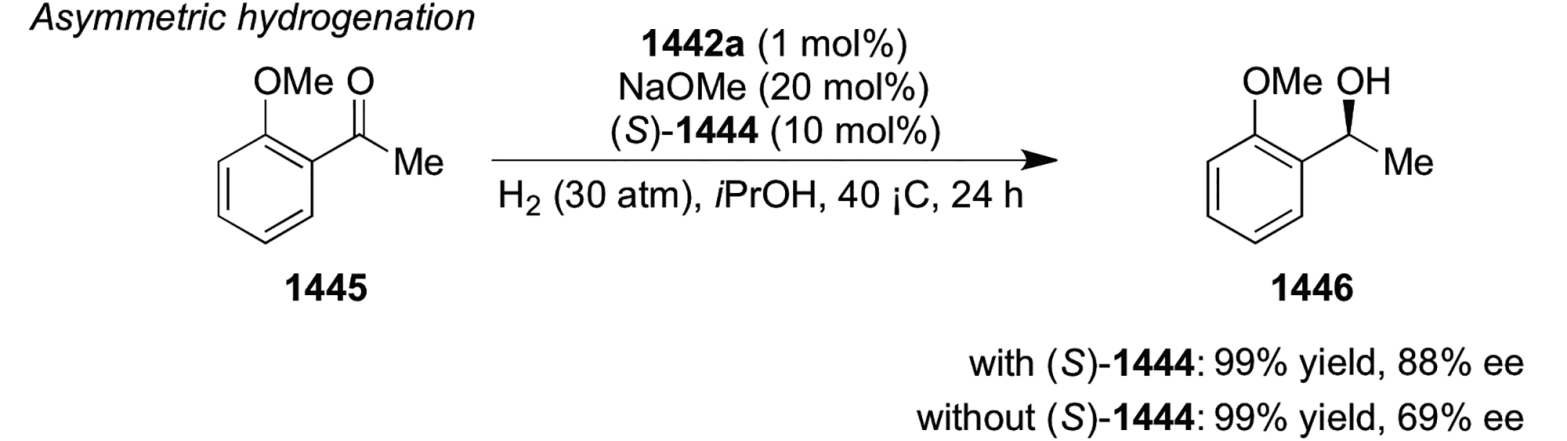
Ru-Catalyzed Asymmetric Hydrogenation of Ketone **1445**

Nishiyama later reported an enhancement of the enantioselectivity in the asymmetric hydrogenation of ketones by including alcohol (*S*)-**1444** as an additive. For example, for the asymmetric hydrogenation of ketone **1445**, catalyzed by complex **1442a**, the enantioselectivity increased from 69% *ee* without the additive to 88% *ee* with the additive. In all but one reported case, an increase in the enantioselectivity was observed (Scheme 429).^515^

**Scheme 430:**
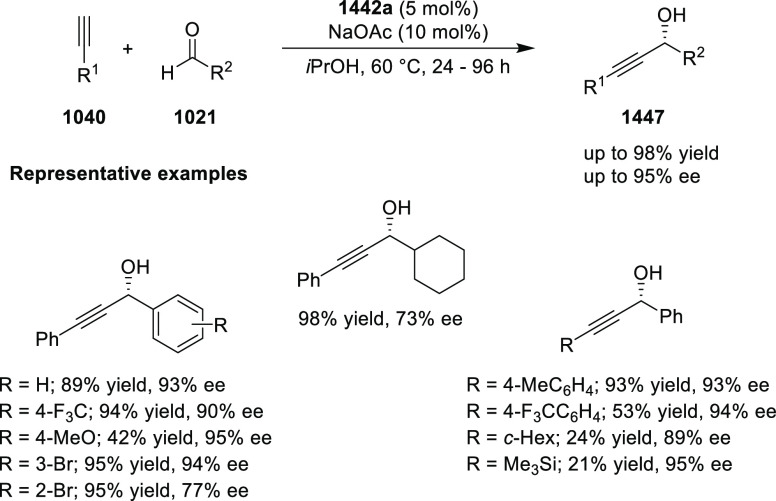
Asymmetric Ru-Catalyzed Alkynylation of Aldehydes

Nishiyama applied the Ph-PheBOX-Ru complex **1442a** in the asymmetric Ru-catalyzed alkynylation of aldehydes **1021**. A range of enantioenriched alcohols **1447** were isolated in up to 98% yield and with up to 95% *ee* (Scheme 430). Aryl alkynes were well tolerated in the reaction, while cyclohexyl and trimethylsilyl alkynes gave the corresponding alcohols in low yields but with high levels of enantioselectivity.^516^

**Scheme 431:**
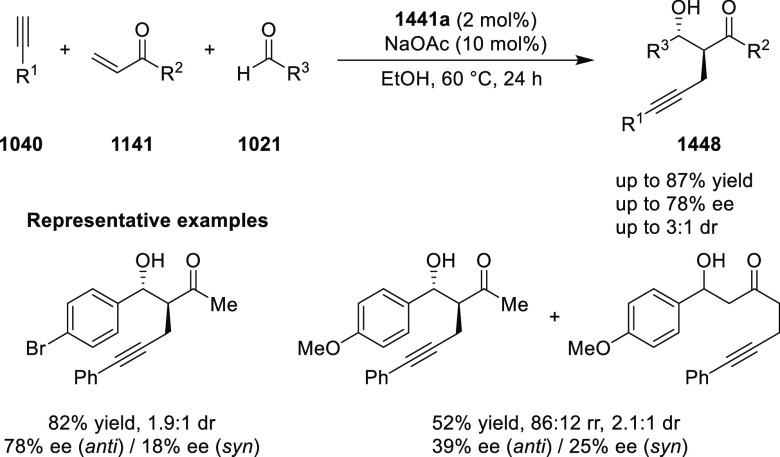
Ru-Catalyzed Enantioselective 3-Component Coupling Reaction of Alkynes, Enones, and Aldehydes

Nishiyama later applied Ph-PheBOX-Ru complex **1441a** in the racemic conjugate alkyne addition to α,β-unsaturated carbonyls. A single asymmetric example of an alkyne addition to a β-substituted enone was reported, giving the product in 82% *ee*.^517^ This led to the development of the **1441a**-catalyzed enantioselective 3-component coupling reaction of alkynes **1040**, enones **1141**, and aldehydes **1021**. The levels of enantioselectivity for the formation of the isolated products were mostly low to moderate, up to 78% *ee* (Scheme 431). The diastereoselectivity was also low, up to 3:1 dr, and in some cases there were regioselectivity issues.^518^

**Figure 76:**
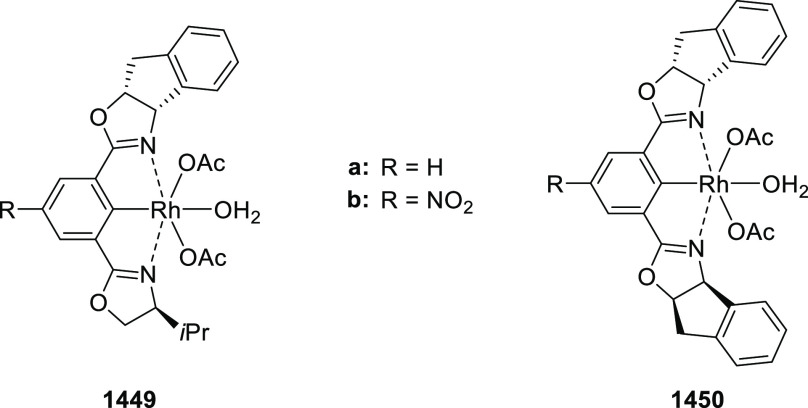
Rh(indanyl-PheBOX) complexes.

Ohshima has reported the asymmetric Rh-catalyzed alkynylation of α-keto esters^519^ and α-ketiminoesters^520^ with *C*_1_-symmetric indanyl-PheBOX-Rh complexes **1449a** and **1449b** and *C*_2_-symmetric indanyl-PheBOX-Rh complexes **1450a** and **1450b** (Figure 76).

**Scheme 432:**
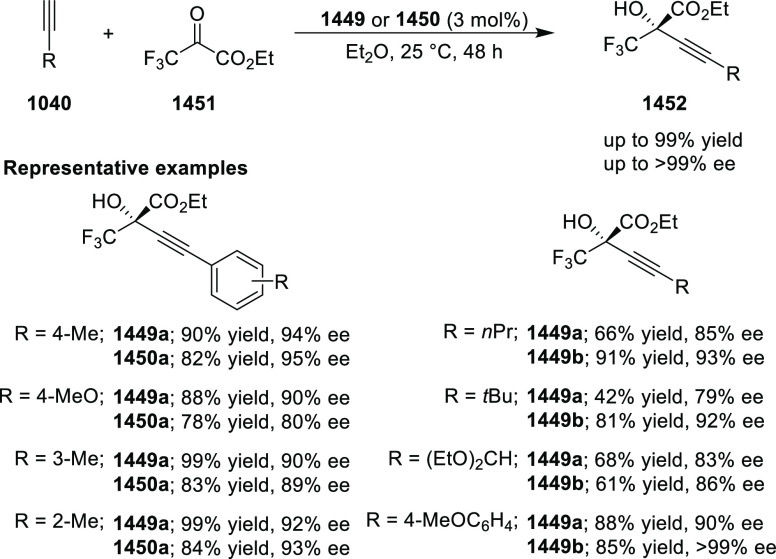
Asymmetric Rh-Catalyzed Alkynylation of α-Keto Esters

Under optimized conditions, enantioenriched tertiary alcohols **1452** could be accessed from α-keto ester **1451** in up to 99% yield and with up to >99% *ee* (Scheme 432). Initially, complexes **1449a** and **1450a** were applied in the reaction with aryl-substituted alkynes. *C*_1_-Symmetric **1449a** was found to outperform *C*_2_-symmetric **1450a** giving the products in generally higher yields and in most cases, slightly higher enantioselectivities. Nitro-substituted *C*_1_-symmetric ligand **1449b** was applied in the reactions of less reactive substrates and was found to drastically improve the isolated yields and enantiomeric excesses of the tertiary alcohol products **1452** when compared to **1449a**. Interestingly, in a competition experiment between benzaldehyde and α-keto ester **1451**, the reaction catalyzed by **1449a** gave no observable alkynylation of the aldehyde.

**Scheme 433:**
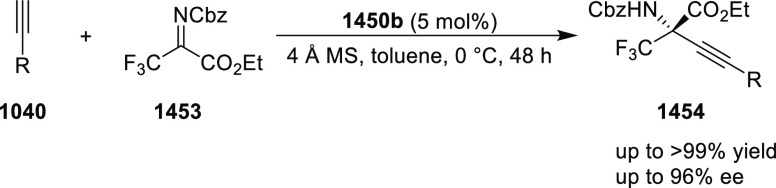
Asymmetric Rh-Catalyzed Alkynylation of α-Ketiminoesters

Later, Ohshima found that the *C*_2_-symmetric indanyl-PheBOX-Rh complexes **1450a** and **1450b** outperformed their *C*_1_-symmetric counterparts in the asymmetric alkynylation of α-ketiminoester **1453**. Under optimized conditions with nitro-substituted **1450b**, a range of aryl, alkenyl, alkyl and silyl-substituted alkynes **1040** were subjected to this reaction with α-ketiminoester **1453** to give a range of tertiary amines **1454** in up to >99% yield and with up to 96% *ee* (Scheme 433).

**Figure 77:**
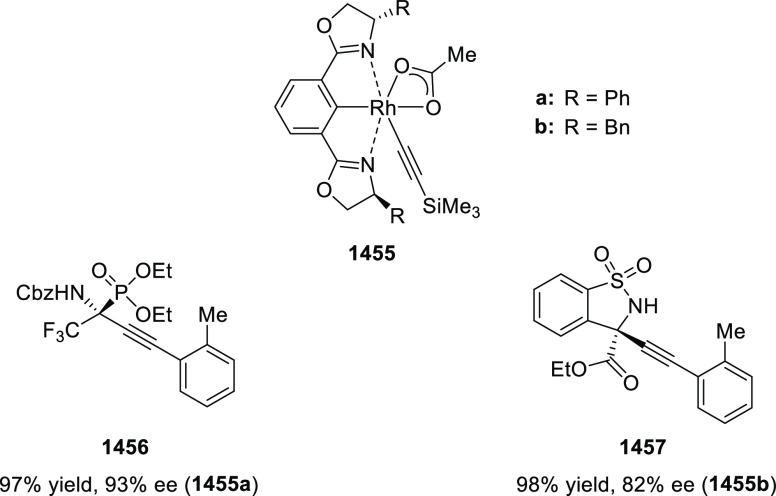
Rh(PheBOX) complex **1455** and cyclic *N*-sulfonyl ketiminoester- and α-ketiminophosphonate- derived amines **1456** and **1457**.

A follow-up mechanistic study found that the application of (trimethylsilylethynyl)(PheBOX)Rh complexes **1455a**–**b** in the alkynylation of α-ketoiminoesters allowed for the alkynylation of less reactive α-ketiminoesters, for example cyclic *N*-sulfonyl ketiminoester- and α-ketiminophosphonate- derived amines **1456** and **1457** were accessed in high yields of 97% and 98%, respectively, and enantioselectivities of 93% *ee* and 82% *ee*, respectively (Figure 77).^521^

**Scheme 434:**
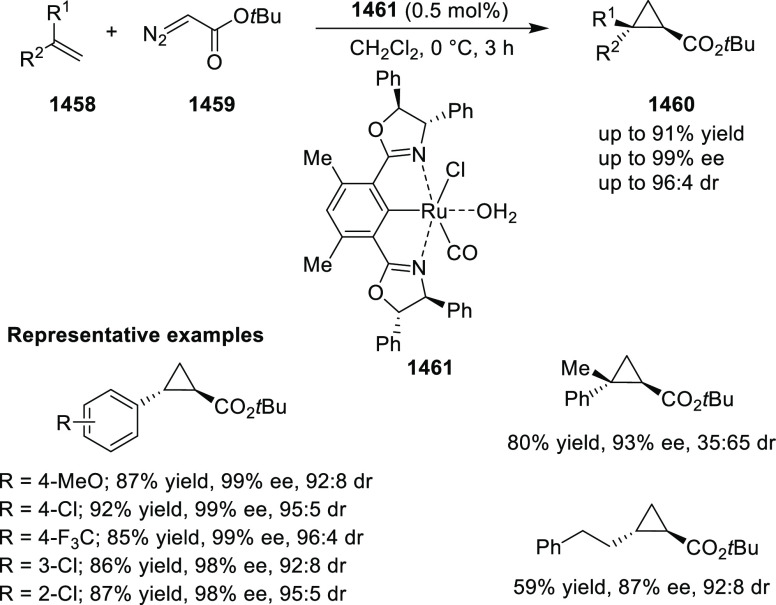
Asymmetric Ru-Catalyzed Cyclopropanation of Terminal Alkenes

Nishiyama has reported the asymmetric Ru-catalyzed cyclopropanation of terminal alkenes **1458** utilizing diPh-PheBOX-Ru **1461**. A range of *trans*-cyclopropanes **1460** were isolated from the reaction with *tert-*butyl-α-diazoacetate **1459** in up to 91% yield, with up to 96:4 dr and 99% *ee* (Scheme 434). Styrene derivatives performed well in the reaction, giving the *trans*-cyclopropanes with high dr, while the only reported disubstituted alkene example gave the opposite diastereoselectivity (35:65 *anti*/*syn*).^522^

**Scheme 435:**
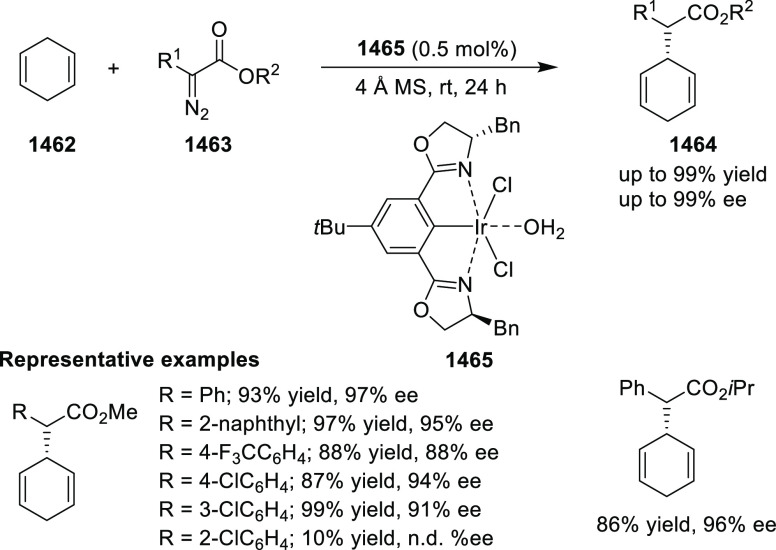
Ir-Catalyzed Asymmetric C–H Activation of 1,4-Cyclohexadiene

The vast majority of PheBOX applications over the past decade have been in Rh- and Ru-catalyzed transformations. However, Davies and Blakey have reported a PheBOX-Ir-catalyzed asymmetric C–H activation of 1,4-cyclohexadiene **1462** with carbenoids. A range of enantioenriched products were isolated from the reaction catalyzed by *t*Bu-substituted-Bn-PheBOX-Ir complex **1465** in up to 99% yield and with up to 99% *ee* (Scheme 435). The *t*Bu-group in the backbone of the ligand was found to increase the isolated yield of the product without affecting the high levels of enantioselectivity.^523^

**Scheme 436:**
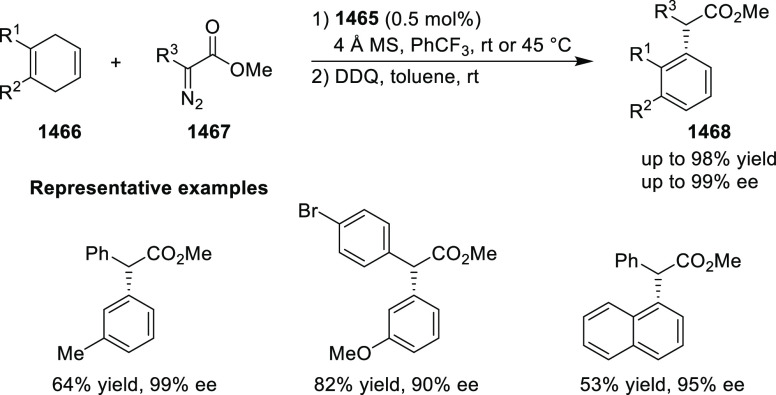
Ir-Catalyzed Asymmetric C–H Activation of Substituted 1,4-Cyclohexadienes

In the same report, a small range of substituted cyclohexadienes **1466** were also subjected to the site selective C–H activation with subsequent oxidation by DDQ to give a range of enantioenriched α-diarylesters **1468** in up to 98% yield and with up to 99% *ee* (Scheme 436).

PheBOX ligands have been successfully applied in a whole range of asymmetric transformations of Rh, Ru and Ir. The main disadvantage of these ligands is that the catalysts must be synthesized prior to use. While this will most likely prevent the widespread use of these ligands in asymmetric catalysis, we expect to see future developments, especially in asymmetric Ir-catalysis, which has not been extensively explored.

#### 3.2.16 Bis(oxazoline) Ligands with Dibenzofuran Linkers

**Figure 78:**
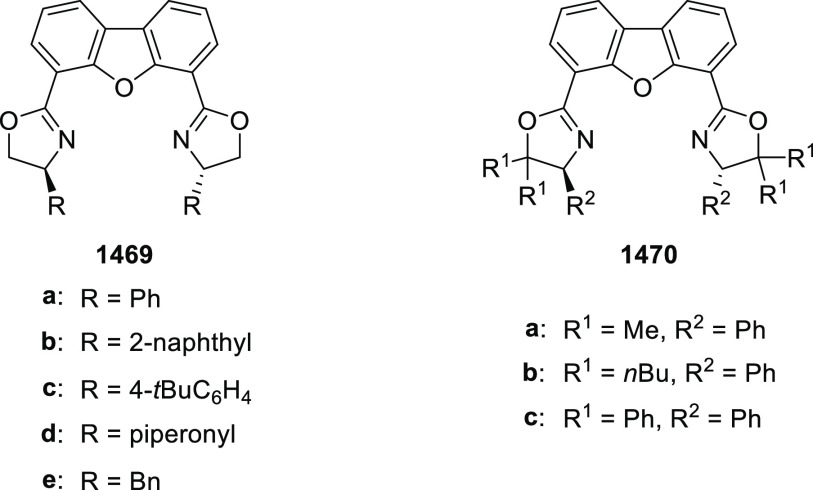
Dibenzofuran-4,6-bis(oxazoline) (DBFOX) ligands **1469**–**1470**.

Dibenzofuran-4,6-bis(oxazoline) (DBFOX) ligands of the type **1469** and **1470**, which bear a coordinating *O* atom in the bridging dibenzofuran moiety, have been developed for a range of applications in asymmetric metal catalysis (Figure 78). In fact, this area was comprehensively reviewed in 2018 by Itoh and Sibi.^524^ As a result, we will only discuss some recent examples in detail.

Since 2008, DBFOX ligands have been employed in, for example, enantioselective Friedel–Crafts of pyrroles,^525,526^ hydride shift/ring closure cascade,^527^ α-cyanation,^528^ α-fluorination,^529−531^ and α-hydroxylation of carbonyls,^532^ enolate protonation^533^ and radical conjugate addition^534^ chemistry, utilizing Zn-, Cu-, Ni- and Mg-catalysis.

**Scheme 437:**
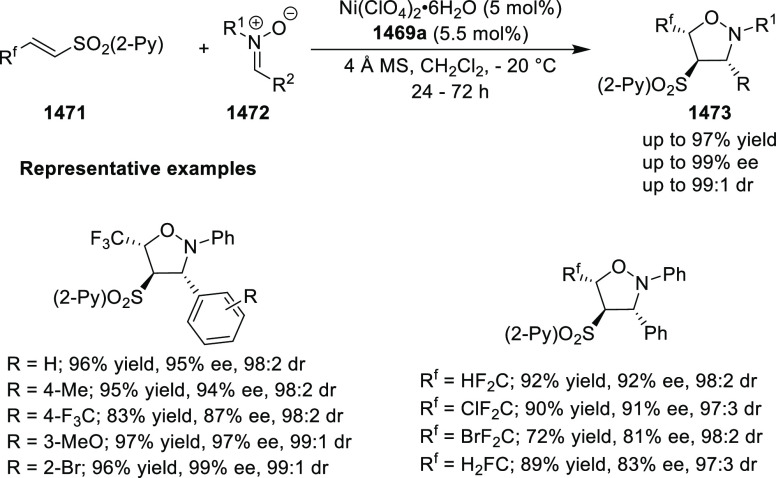
Asymmetric 1,3-Dipolar Cycloaddition of β-Fluoroalkylated α,β-Unsaturated 2-Pyridylsulfones with Nitrones

Recently, Huang and Shibata reported the catalytic asymmetric 1,3-dipolar cycloaddition of β-fluoroalkylated α,β-unsaturated 2-pyridylsulfones **1471** with nitrones **1472** for the enantioselective synthesis of chiral fluoroalkylated isoxalidinones **1473**, which can be converted into γ-amino alcohols. Under optimized conditions with Ni(ClO_4_)_2_·6H_2_O and Ph-DBFOX **1469a** as the chiral ligand, a range of isoxalidinones **1473** were accessed in up to 97% yield, with up to 99:1 dr and 99% *ee* (Scheme 437). A range of fluoroalkylated groups (R^f^) were tolerated in the reaction.^535^

**Scheme 438:**
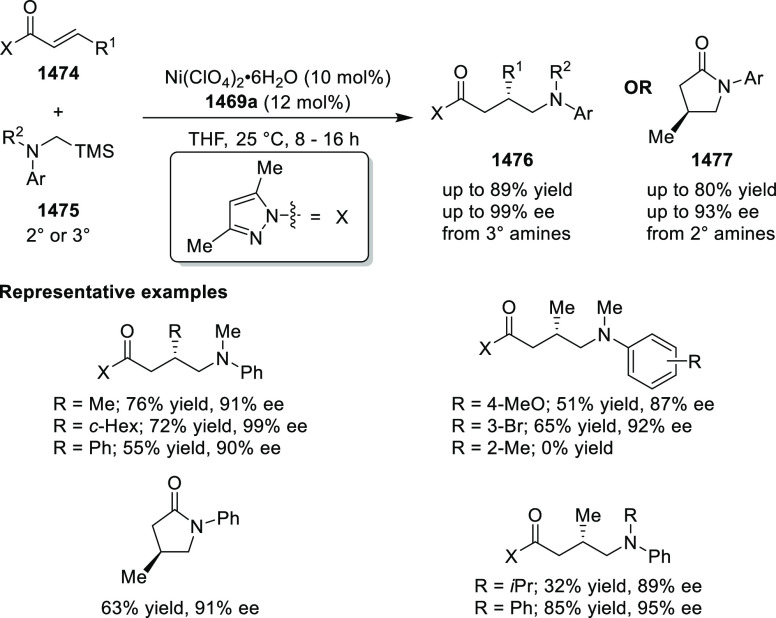
Visible-Light-Promoted Ni-Catalyzed Asymmetric Radical Conjugate Addition

Gong has developed a visible-light-promoted Ni-**1469a**-catalyzed asymmetric radical conjugate addition reaction. The bifunctional Ni-catalyst initiates single electron transfer and provides a chiral environment for effective asymmetric induction. A range of tertiary silylamines **1475** were reacted with α,β-unsaturated *N*-acyl pyrazoles **1474** to give γ-amino acids **1476** in up to 89% yield and with up to 99% *ee* (Scheme 438). When secondary amines were used in the reaction under the optimized conditions, subsequent lactamization occurred to give enantioenriched γ-lactams **1477** in up to 80% yield and with up to 93% *ee*.^536^

We chose to highlight the recent Ni-catalyzed transformations that the DBFOX ligands have been applied in because the development of asymmetric 3d-transition-metal-catalyzed reactions is an important area in modern organic chemistry. We hope that these ligands can be further applied in this area.

## 4 Tetradentate Bis(oxazoline) Ligands

**Figure 79:**
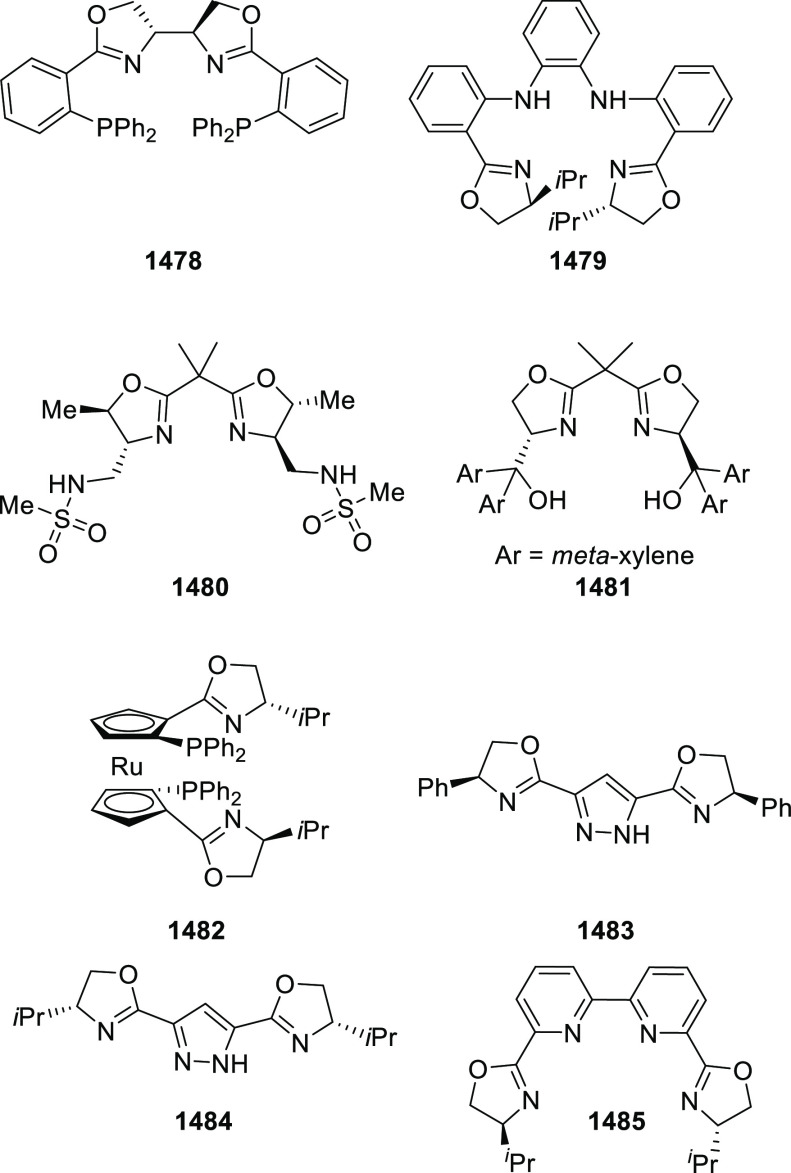
Summary of tetradentate bis(oxazolines) ligands.

**Scheme 439:**
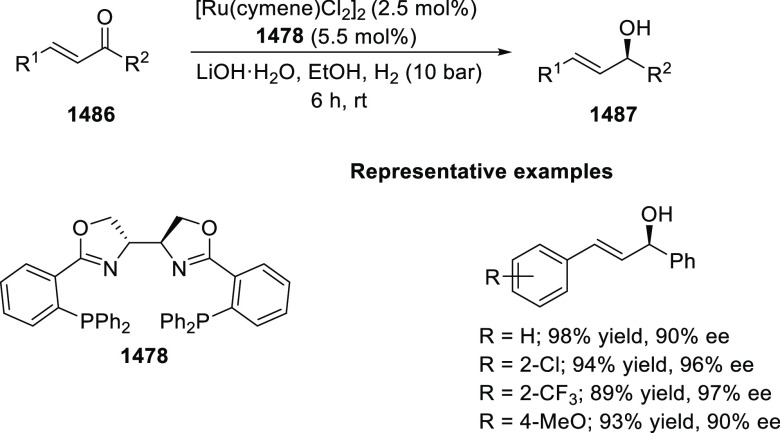
Ru-Catalyzed Asymmetric Reduction of Ketones

In 2013, Li reported the use of chiral biphosphinobioxazoline ligand **1478** in the asymmetric reduction of α,β-unsaturated ketones **1486** furnishing the product **1487** in up to 89% yield and 97% *ee* (Figure 79, Scheme 439). Interestingly when ^t^BuPHOX ligand was used instead in this catalytic transformation only <5% product **1489** was observed. This Ru-catalyst system was also noted to be tolerant of water and air, providing very mild conditions for access to enantioenriched allylic alcohols.^537^

**Scheme 440:**
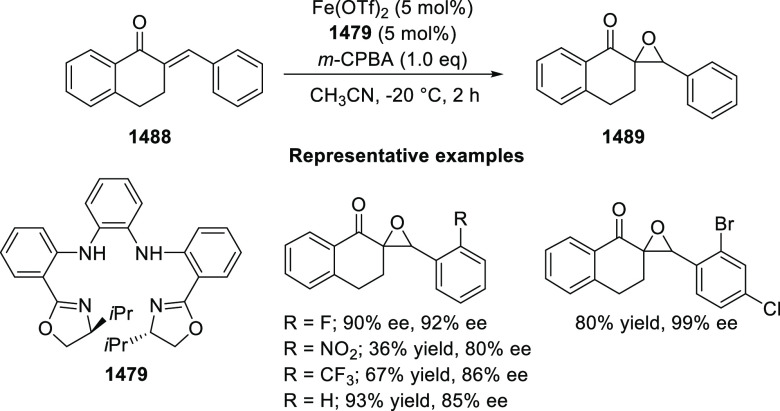
Fe-Catalyzed Asymmetric Epoxidation

Gao reported the use of the tetradentate porphyrin inspired ligand **1479** in the Fe-catalyzed asymmetric epoxidation of electron deficient alkenes **1488** (Scheme 440). A range of electronically varied alkenes **1488** were screened using the optimized reaction conditions furnishing the epoxide **1489** in up to 80% yield and 99% *ee*. The utility of this reaction was further tested by carrying out gram-scale epoxidations with no erosion of enantioselectivity observed.^538^

**Scheme 441:**
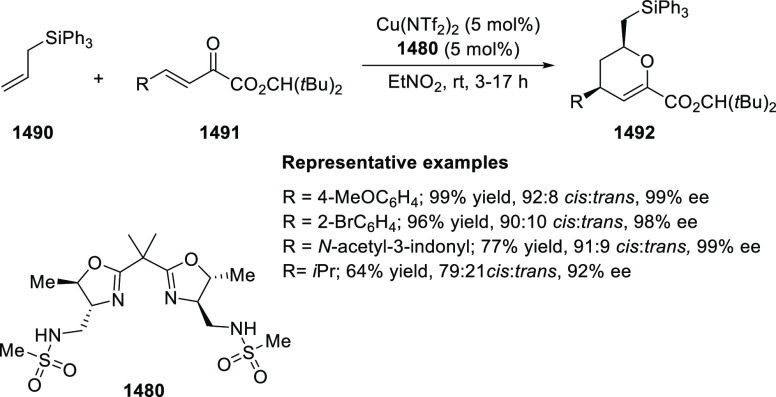
Cu-Catalyzed Asymmetric Synthesis of Oxanes

Ishihara developed a novel sulfonamide-based bis(oxazoline) ligand **1480** and applied it in the inverse-electron-demand hetero Diels–Alder reaction of β,γ-unsaturated α-keto esters **1491** with allyl silanes **1490** (Scheme 441). This methodology gives access to chiral oxanes **1492** rather than cyclic acetals which are the product of the conventional inverse-electron-demand hetero Diels–Alder reaction. A broad substrate scope was carried out furnishing the product **1492** in up to 99% yield and 99% *ee* in a 92:8 *cis*/*trans* ratio.^539^ It was also applied in the asymmetric Cu-catalyzed Diels–Alder reaction to great effect.^540^

**Scheme 442:**
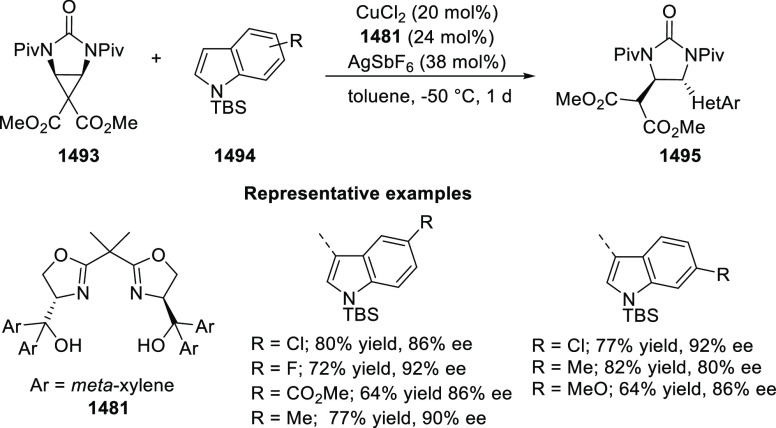
Cu-Catalyzed Desymmetrization of *meso*-Diaminocyclopropanes

Waser reported the Cu-catalyzed desymmetrization of *meso*-diaminocyclopropanes **1493** in 2018. This modified Friedel–Crafts reaction was originally attempted using BOX ligand **600c** which only furnished the product **1495** in 74% yield, 64% *ee*, and >20:1 dr. To increase the enantioselectivity the native BOX ligand **600c** was modified in the α-position to furnish a large range of ligands containing bulky alcohols. After screening the structure of the ligand further by fine-tuning the sterics, the Cu complex of ligand **1481** afforded the product **1495** in 80% yield, 86% *ee*, and >20:1 dr. With the optimized conditions in hand a range of electronically and sterically diverse *N*-TBS indoles **1494** were screened in this catalytic transformation, furnishing the product **1495** in up to 82% yield, 92% *ee*, and all in >20:1 dr (Scheme 442).^541^

**Scheme 443:**
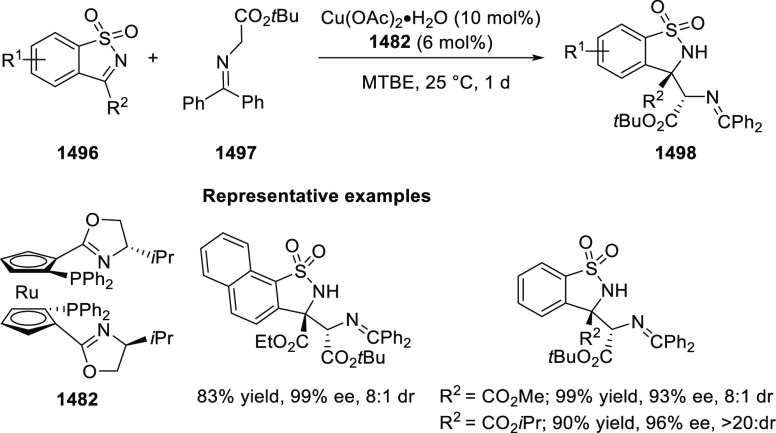
Cu-Catalyzed Enantioselective Mannich Reaction of Cyclic Ketimines

Zhang reported the Cu-catalyzed asymmetric Mannich reaction of cyclic ketimines **1496** and glycine Schiff bases **1497**. Planar chiral ferrocene ligands have been widely used in asymmetric catalysis in recent times, however in contrast, ruthenocene based ligands have not been as widely applied in asymmetric catalysis. In this reaction the tetradentate planar-chiral ruthenocene ligand **1482** showed excellent selectivity giving the product in up to 83% yield, 99% *ee*, and 7:1 dr. The substrate scope highlighted the trend that the ester functionality in the cyclic ketimine **1496** had a large effect on the enantioselectivity. Sterically bulky esters such as an isopropropyl ester led to an increase in enantioselectivity and diastereoselectivity (96% *ee*, >20:1 dr, and 90% yield) compared to methyl esters (99% yield, 93% *ee*, 8:1 dr). If the cyclic ketimine ester was replaced by a methyl group this caused a pronounced decrease in all selectivity to 26% yield, 8% *ee*, and 1:1 dr (Scheme 443).^542^ In other publications by Zhang’s group, this ligand was applied in the Pd-catalyzed allylation of amino acid derivatives, *N*-sulfonylimines and in the asymmetric synthesis of chromanols.^127,543−546^

**Scheme 444:**
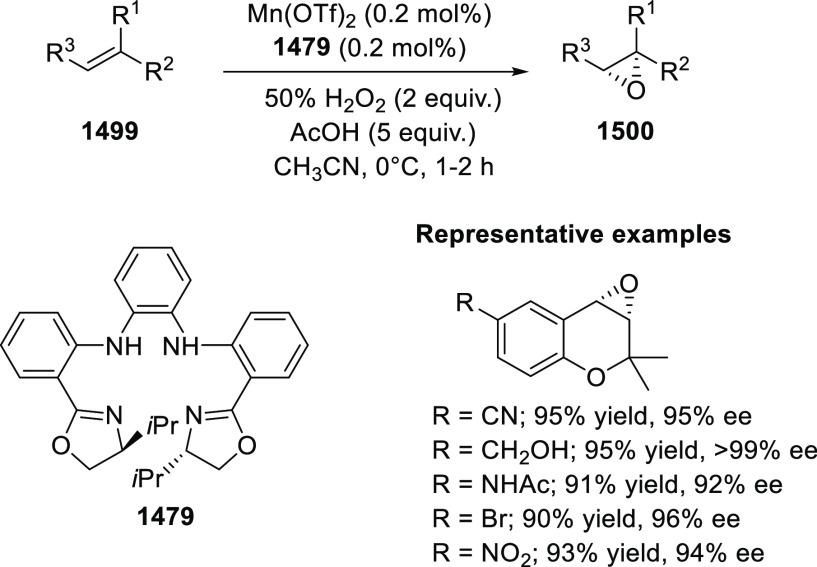
Mn-Catalyzed Enantioselective Epoxidation of Alkenes

In 2013 Gao reported the synthesis of ligand **1479** as a porphyrin mimic and subsequently utilized it in the Mn-catalyzed asymmetric epoxidation of alkenes **1499** (Scheme 444). This methodology was applied to a range of alkenes **1499** forming the product **1500** in up to 95% yield and >99% *ee*.^547^ In a follow up publication this methodology was further developed to include a wider substrate scope of cyclic and acyclic alkenes **1499**.^548^

**Scheme 445:**
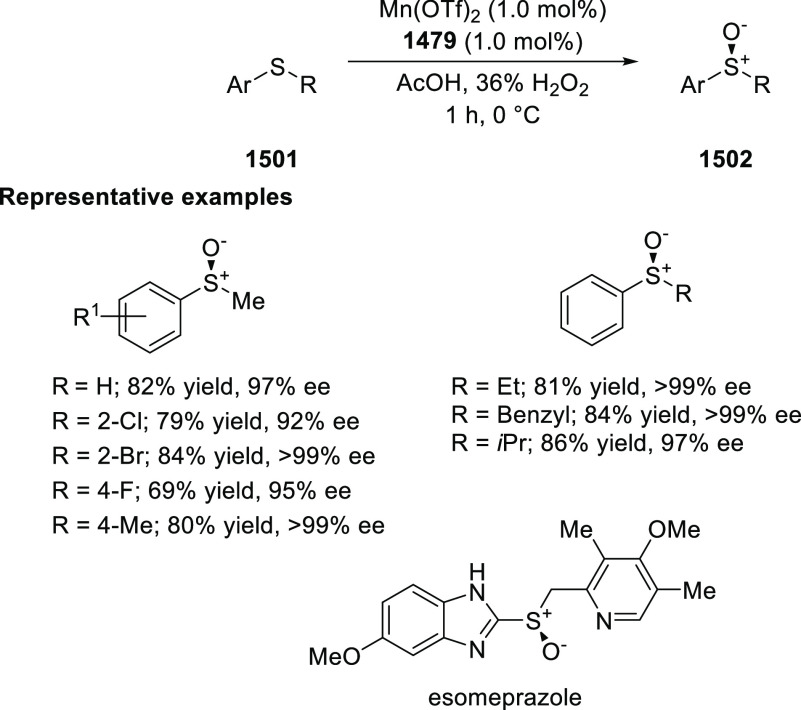
Mn-Catalyzed Enantioselective Epoxidation of Sulfides

Ligand **1479** was further used by Gao in the Mn-catalyzed asymmetric oxidation of aryl sulfides **1501**. This methodology encompassed a large substrate scope with all aryl methyl sulfides **1501** affording the product **1502** in up to 84% yield and >99% *ee* (Scheme 445). This methodology was expanded in a subsequent publication and transferred from a batch reaction to a flow set up. This allowed for quicker reactions, the lowering of catalyst loading from 1.0% to 0.5% and for a gram scale synthesis of chiral sulfoxides.^549,550^

**Scheme 446:**
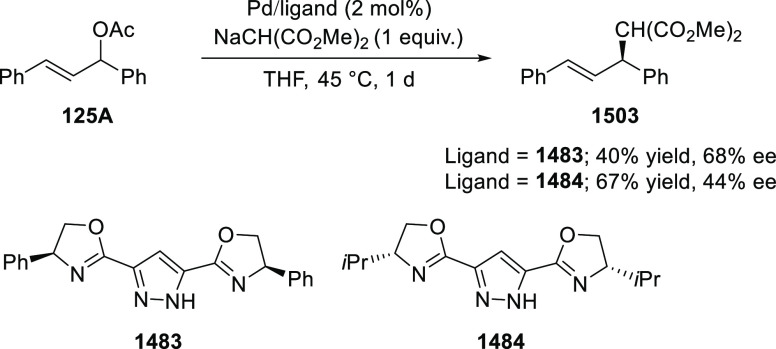
Pd-Catalyzed Enantioselective Allylic Alkylation

Meyer reported the synthesis of a pyrazole-bridged bis(oxazoline) ligands **1483**/**1484** and their application in the Pd-catalyzed allylic alkylation of 1,3-diphenylallyl acetate **125A** (Scheme 446). Interestingly, the active Pd-complex was shown to be a dinuclear palladium species with ligands **1483**/**1484**. While all Pd complexes of ligands **1483**/**1484** yielded the desired product **1503** there was a large variation in the levels of enantioselectivities observed, the phenyl-substituted ligand **1483** gave the product in 40% yield and 68% *ee* while **1484** did so in 67% yield and 44% *ee*.^551^

**Scheme 447:**
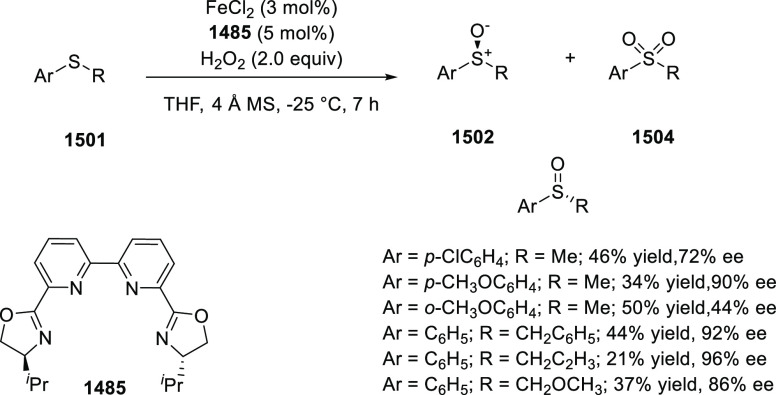
Fe-Catalyzed Asymmetric Oxidation of Aromatic Sulfides

In 2017 Ollevier reported the asymmetric oxidation and tandem kinetic resolution of aryl sulfides **1501** to sulfoxides **1502** (Scheme 447). Ligand **1485** was chosen as a porphyrin mimick creating a bioinspired nonhaem Fe^II^ catalyst with FeCl_2_. This system was employed and furnished the products in up to 96% *ee* and 21% yield. This catalysis was shown to be highly sensitive to the nature of the aryl substituents on the aryl sulfides. Placing a methyl group in the *ortho*-position drastically deminished the enantioselectivity to 44% *ee* and 50% yield.^552^

## 5 Tris(oxazoline) and Tetra(oxazoline) Ligands

### 5.1 Tris(oxazoline) Ligands

**Figure 80:**
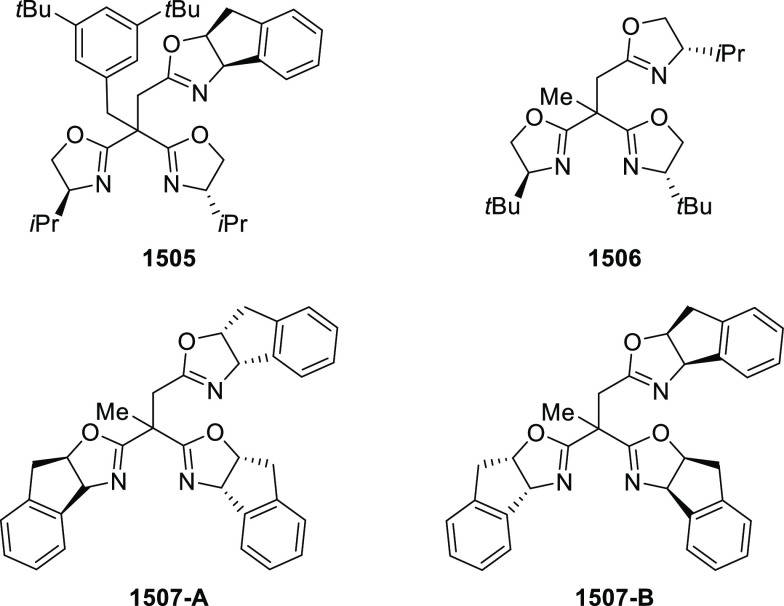
Trisoxazoline ligands (TOX).

Tang, one of the key pioneers in the design, synthesis and applications of metal complexes of trisoxazolines, developed a library of pseudo-C_3_-symmetric trisoxazolines **1505**–**1507** (TOX; Figure 80) by a “side arm” approach. The metal complexes of TOX ligands **1505**–**1507** were found to be efficient catalysts for the asymmetric Friedel–Crafts reaction of indole with alkylidene malonate, the asymmetric intramolecular Cannizzaro reaction of aryl and alkyl glyoxals, the asymmetric [3 + 3] cycloaddition of donor–acceptor (D-A)-substituted cyclopropane diesters with aromatic azomethine imines and the asymmetric oxa-[3 + 3]-annulation of oxygenated phenols and 3-aminophenols with β,γ-unsaturated α-keto esters. Compared with the corresponding bisoxazoline ligands, these metal-TOX complexes showed some promising properties, such as better enantioselectivity and stronger tolerance toward water and air.

**Scheme 448:**
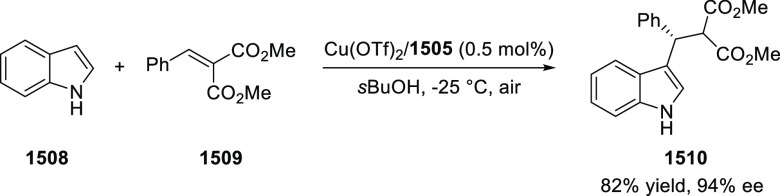
Asymmetric Friedel–Crafts Alkylation between Indoles and Pyrroles and Alkylidene Malonates

In 2011, Tang reported a new pseudo-C3-symmetric heterotrisoxazoline (**1505**), which in combination with Cu(OTf)_2_ displayed excellent enantioselectivity in the asymmetric Friedel–Crafts alkylation between indoles (**1508**) and pyrroles and alkylidene malonates (**1509**) (Scheme 448).^553^ Enantioselectivities of up to 94% *ee* were achieved with only 0.5 mol% of catalyst loading.

**Scheme 449:**
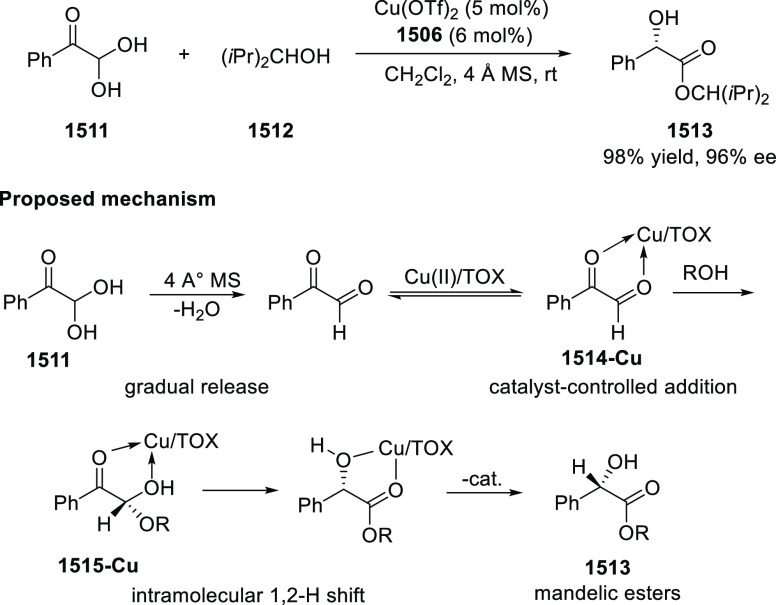
Asymmetric Intramolecular Cannizzaro Reaction of Aryl and Alkyl Glyoxals with Alcohols

Later in 2013, the first asymmetric intramolecular Cannizzaro reaction of aryl and alkyl glyoxals (**1511**) with alcohols (**1512**) was reported with excellent levels of enantioselectivity by using a newly developed congested TOX ligand (**1506**) and Cu(OTf)_2_ with a gradual liberation protocol of active glyoxals from glyoxal monohydrates (**1511**) (Scheme 449).^554^ This method allowed a facile entry to the preparation of a variety of α-hydroxy carboxylic acid derivatives (**1513**) with high optical purity (up to 96% *ee*). The proposed reaction pathway suggested that the chiral induction occurred at the catalyst-controlled face selective addition of alcohols to coordinated glyoxals. The improved stereocontrol with the TOX ligand **1506** over the addition of the alcohol to **1515-Cu** could be described in terms of a more congested environment created around the reactive site with the aid of the extra *t*-butyl oxazoline, while the more typical bisoxazoline (BOX) ligands lack such a side arm regulator.

**Scheme 450:**
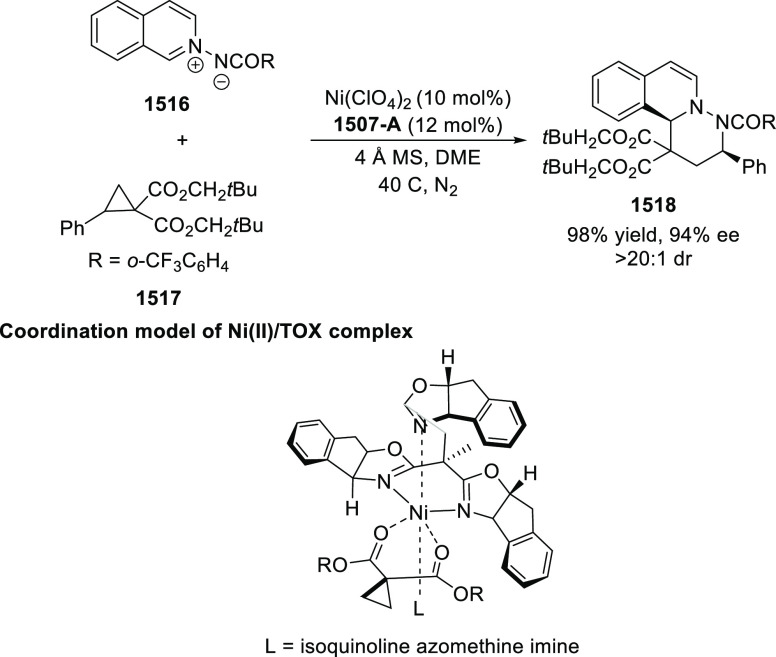
Ni/In-TOX-Catalyzed Highly Enantioselective Asymmetric [3 + 3] Cycloaddition

In 2013, Tang reported the Ni/In-TOX (**1507-A**)-catalyzed highly enantioselective asymmetric [3 + 3] cycloaddition of donor–acceptor (D–A)-substituted cyclopropane diesters (**1517**) with aromatic azomethine imines (**1516**) (Scheme 450).^555^ A variety of 6,6,6-tricyclic dihydroisoquinoline derivatives (**1518**) were synthesized in up to 98% yields with excellent diastereo- and enantioselectivities (>20:1 dr and up to 94% *ee*). Preliminary results and DFT studies suggested that the π–π interaction between the indane group of the ligated side arm and the phenyl group of the cyclopropane plays a vital role in the control of enantioselectivity. On the basis of computational studies, the optimized model of Ni(II)/TOX (**1507-A**) was proposed to be a six-coordinate Ni(II) complex with one molecule of isoquinoline azomethine imine coordinating to the Ni center.

**Scheme 451:**
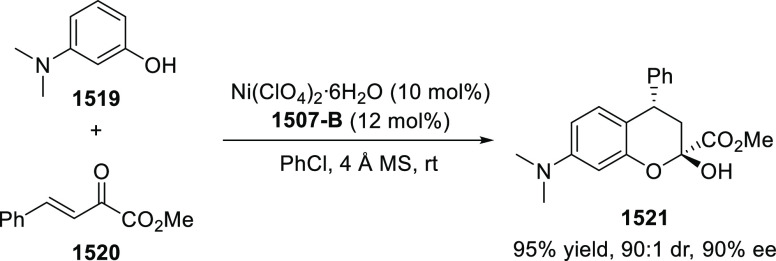
Ni(II)/TOX-Catalyzed Asymmetric Oxa-[3 + 3]-annulation

In 2018, the first Ni(II)/TOX-catalyzed asymmetric oxa-[3 + 3]-annulation of oxygenated phenols and 3-aminophenols (**1519**) with β,γ-unsaturated α-keto esters (**1520**) was disclosed (Scheme 451).^556^ This method allowed a rapid access to a variety of oxygenated and 7-aminated chromans (**1521**) in excellent yields (up to 95%) with excellent diastereoselectivities (90:1 dr) and enantioselectivities (up to 90% *ee*). The improved functionality tolerance was attributed to the probable interference of the oxazoline side arm with the coordination of the amino group to the catalyst. Also of note Tang applied the aforementioned TOX ligands in other chemistries such as the asymmetric Nazarov reaction, the enantioselective ring-opening of cyclopropanes and the asymmetric [4 + 3] annulation reaction.^557−560^ Greater detail of TOX ligands and side arm modification strategies for bis(oxazoline) ligand design has been discussed by Tang in the 2014 account.^561^

### 5.2 Tetra(oxazoline) Ligands

**Scheme 452:**
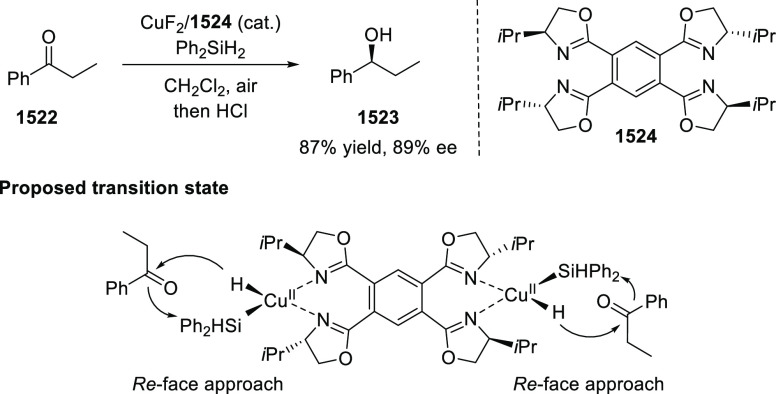
D_2_-Symmetrical Chiral Tetraoxazoline Ligand for Asymmetric Hydrosilylation

In 2010, Li reported a novel D_2_-symmetrical chiral tetraoxazoline ligand (**1524**) and applied it in a Cu-catalyzed asymmetric hydrosilylation of aromatic ketones (**1522**) with diphenylsilane to give optically active secondary alcohols (**1523**) (Scheme 452).^562^ The chiral catalyst showed excellent activities and enantioselectivities in the hydrosilylation of aryl ketones (**1522**) with up to 89% *ee*. A transition state model was proposed wherein diphenylsilane could only approach the *Re*-face of the aromatic ketones due to steric hindrance leading to the (*S*)-absolute configuration observed for all secondary alcohol products.

**Figure 81:**
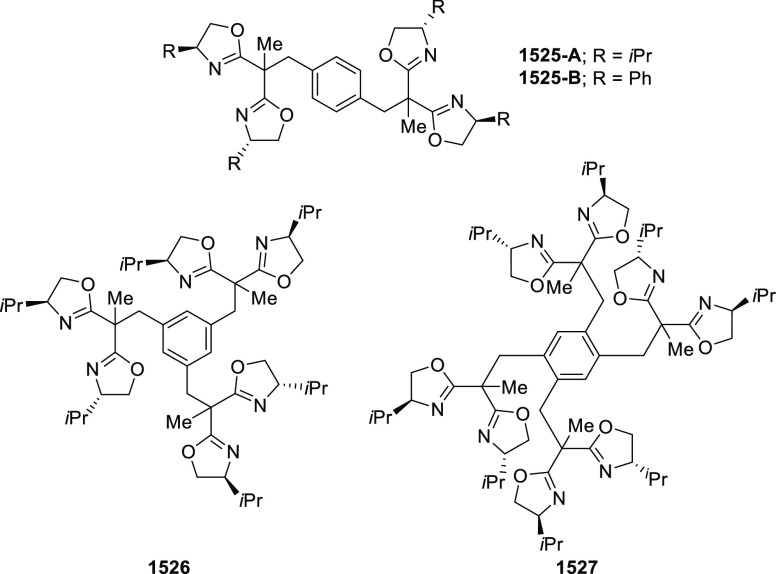
Recyclable oxazoline catalysts.

Bellemin-Laponnaz reported the synthesis and application of self-supported recyclable oxazoline catalysts **1525**–**1527** (Figure 81) in the α-hydrazination of β-keto esters **1535**.

**Scheme 453:**
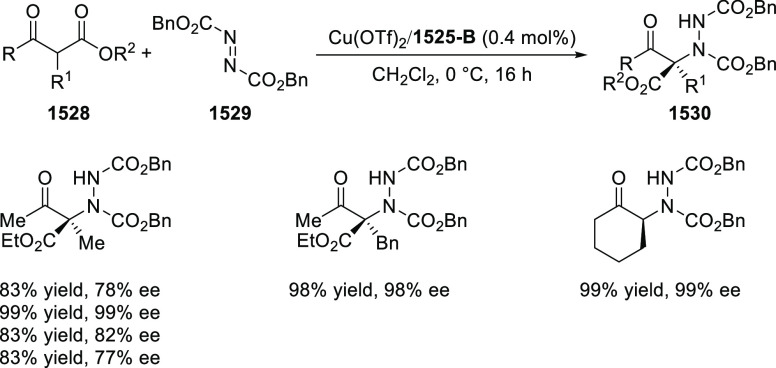
Asymmetric α-Hydrazination of β-Keto Esters

Di-, tri- and tetratopic ligands bearing isopropyl substituents **1525-A**, **1526**, and **1527** were synthesized and screened in the α-hydrazination of β-keto esters **1528**, while enantioselectivities were similar (78–82% *ee*), it was shown that the recyclability of the ligands varied greatly. After five catalytic runs ditopic tetraoxazoline ligand **1525-A** gave the product **1530** in 83% *ee* and 87% yield, tritopic hexaoxazoline ligand **1526** afforded the product in 38% *ee* and <5% yield and tetratopic octaoxazoline ligand **1527** did not give rise to the product **1530** at all. Catalysts were recycled by a solvent swap which allowed them to be decanted from the reaction mixture. It was thought that during the solvent swap Cu was leading to the reduced results for the tri- and tetratopic ligands. When a substrate scope was carried out with **1525-B** enantioselectivities of up to 99% *ee* and 99% yield were achieved with up to 10 catalytic runs possible with recycled catalysts (Scheme 453).^563^

## 6 Conclusion

This Review reports on further developments in the design and application of oxazoline-derived ligands in asymmetric catalysis over a 10-year period since 2009, when the area was previously reviewed by us.

The exceptionally wide utility of this ligand class in catalytic asymmetric synthesis has continued to be illustrated by the high levels of asymmetric induction in a variety of metal-catalyzed transformations in the review period. Many of the tried and trusted oxazoline-containing ligands like PHOX, BOX, and PyBOX continue to be applied with success to new transformations, and there are few examples where the levels of enantioselectivies obtained do not reach 99% *ee*. Variation in the ligand structures mainly relies upon small changes to well-established motifs with additional steric or electronic features being modified. Such structural changes to the parent ligands have been aided by mechanistic, spectroscopic, X-ray crystallographic, and computational studies. While there has been a considerable renaissance in metalla-photoredox and metalla-electrocatalysis in recent times, the combination of these methods with metal-catalyzed asymmetric synthesis has remained limited. As society moves toward increased sustainability, organic chemists must keep up, and there is considerable scope for adapting the privileged oxazoline motifs described in this Review to photoredox- and electrocatalytic methodologies. In a similar vein, we have seen a shift toward the development of catalysts based around the earth-abundant 3d transition metals such as cobalt and nickel. 3d Transition metals have been shown to have similar and, in some cases, superior reactivities compared to their more unsustainable, expensive and toxic late-transition-metal counterparts like palladium and rhodium. We hope to see considerable progress in 3d-transition-metal-catalyzed asymmetric synthesis in the coming years, and the use of oxazoline-based ligands in these transformations is a challenge that is just beginning to be addressed.

Although this Review highlights extensive research in the design, synthesis, and application of oxazoline-derived ligands, this topic is far from being exhausted and it is hoped that this Review will again stimulate both the development/design of new ligands and their applications in novel metal-catalyzed asymmetric transformations.

**Chart 1:**
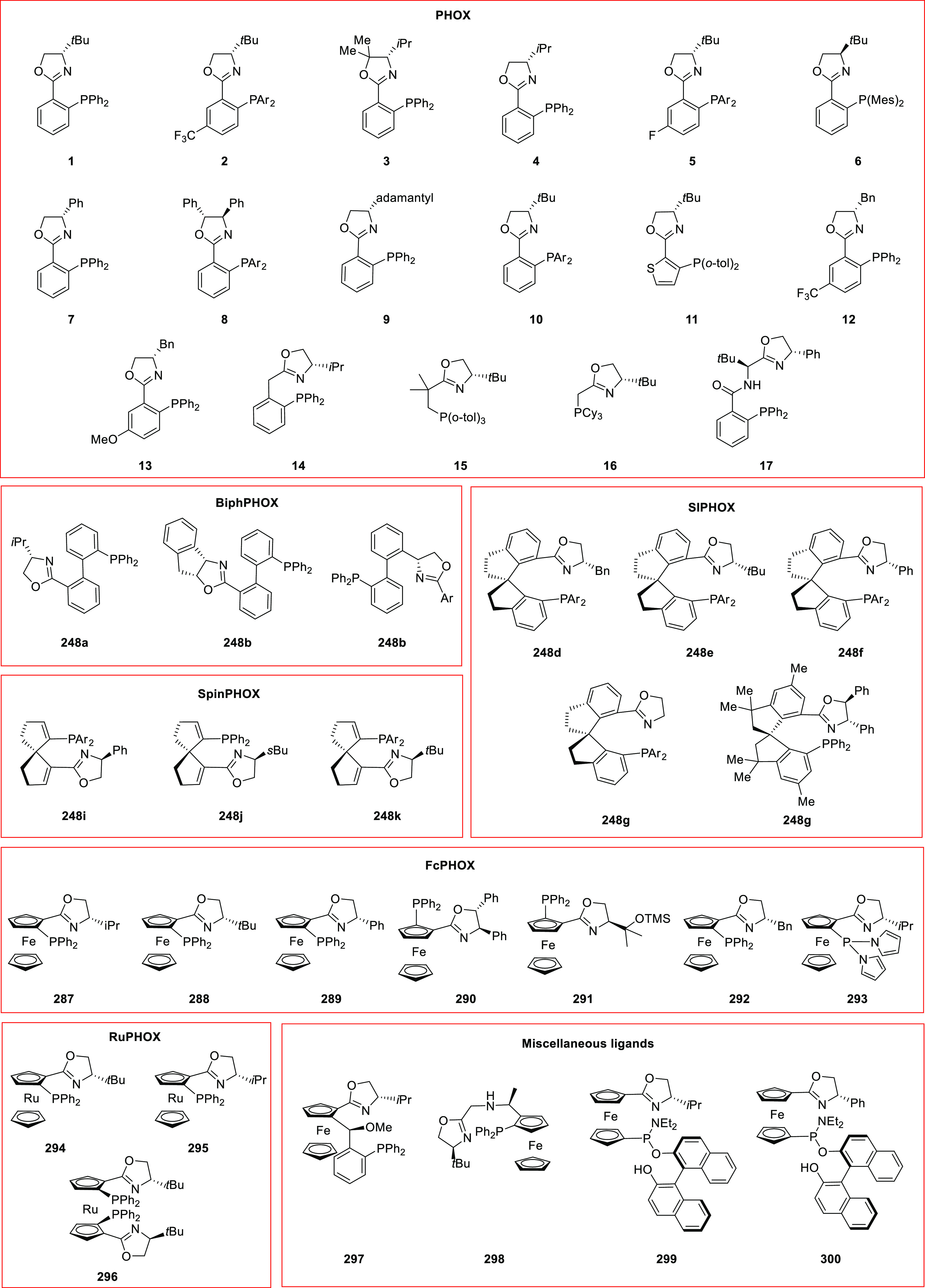

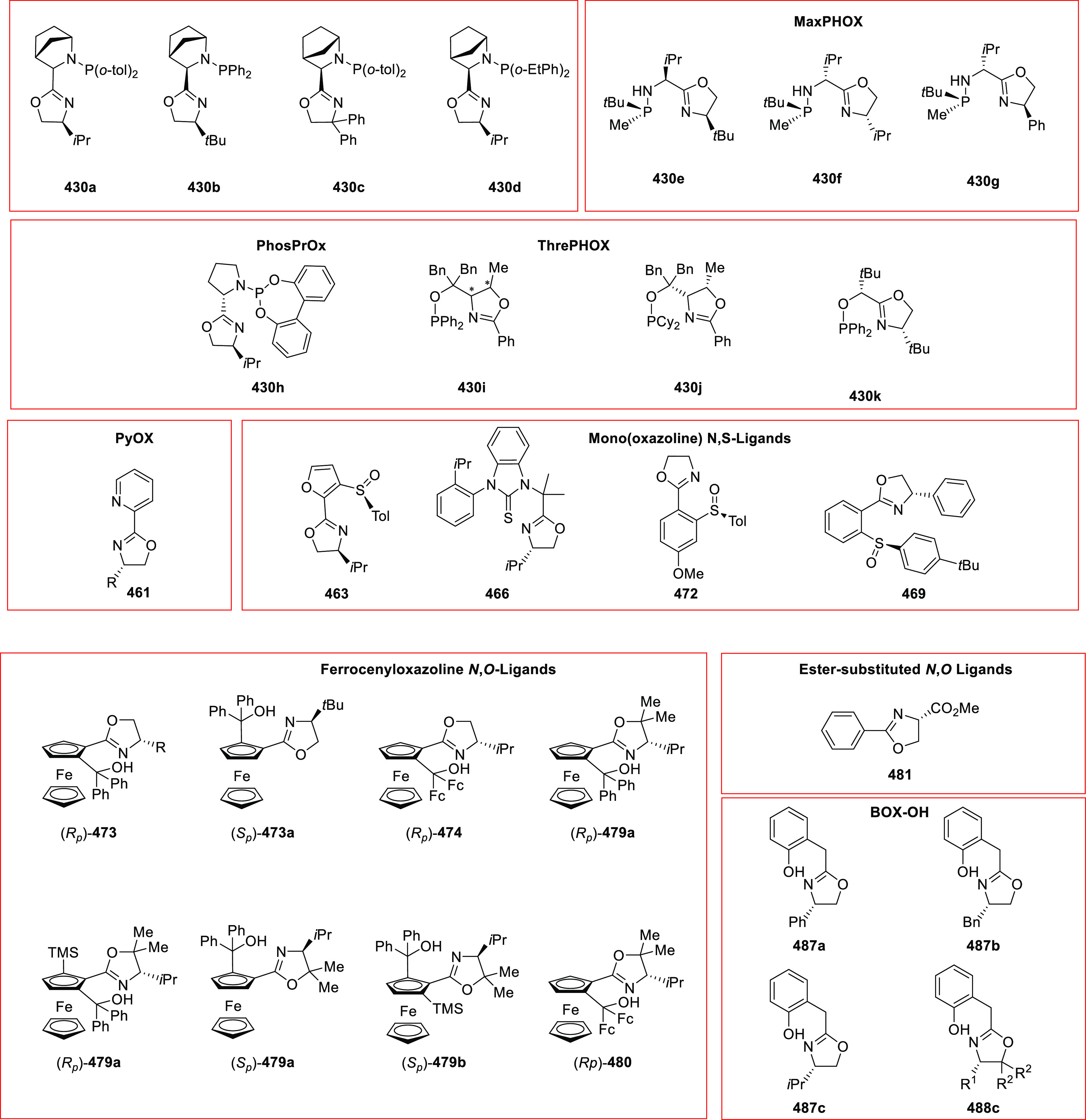

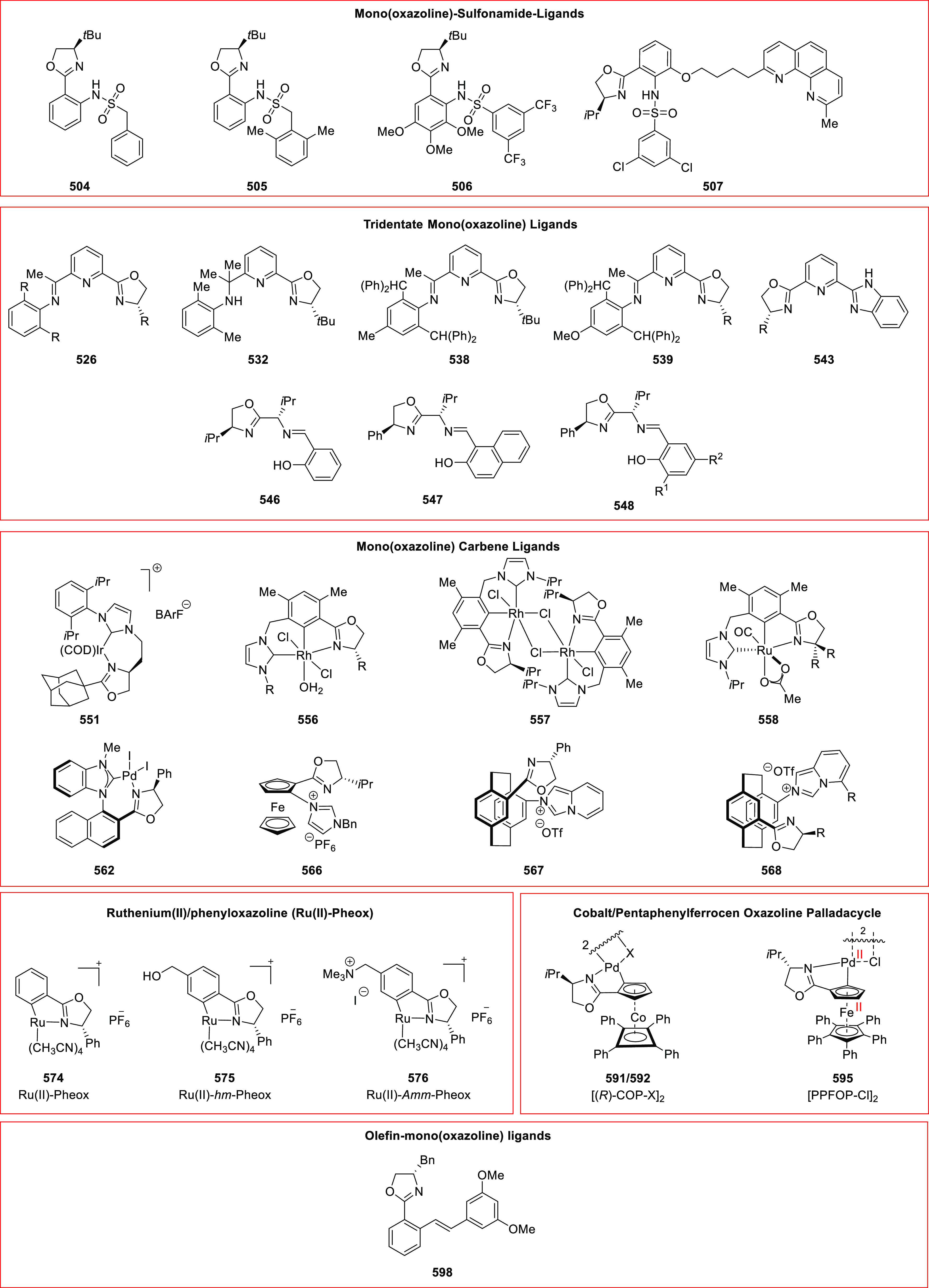

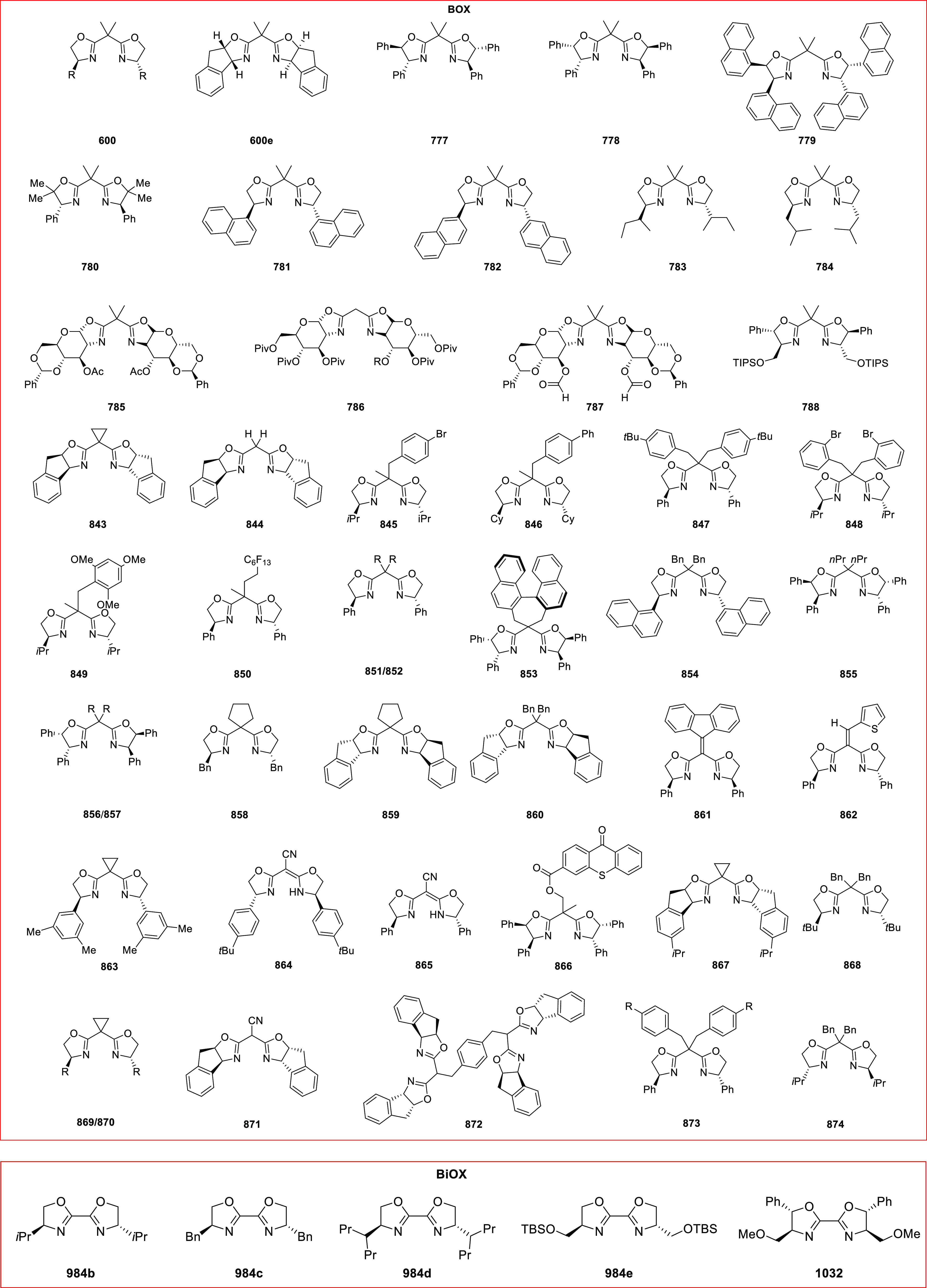

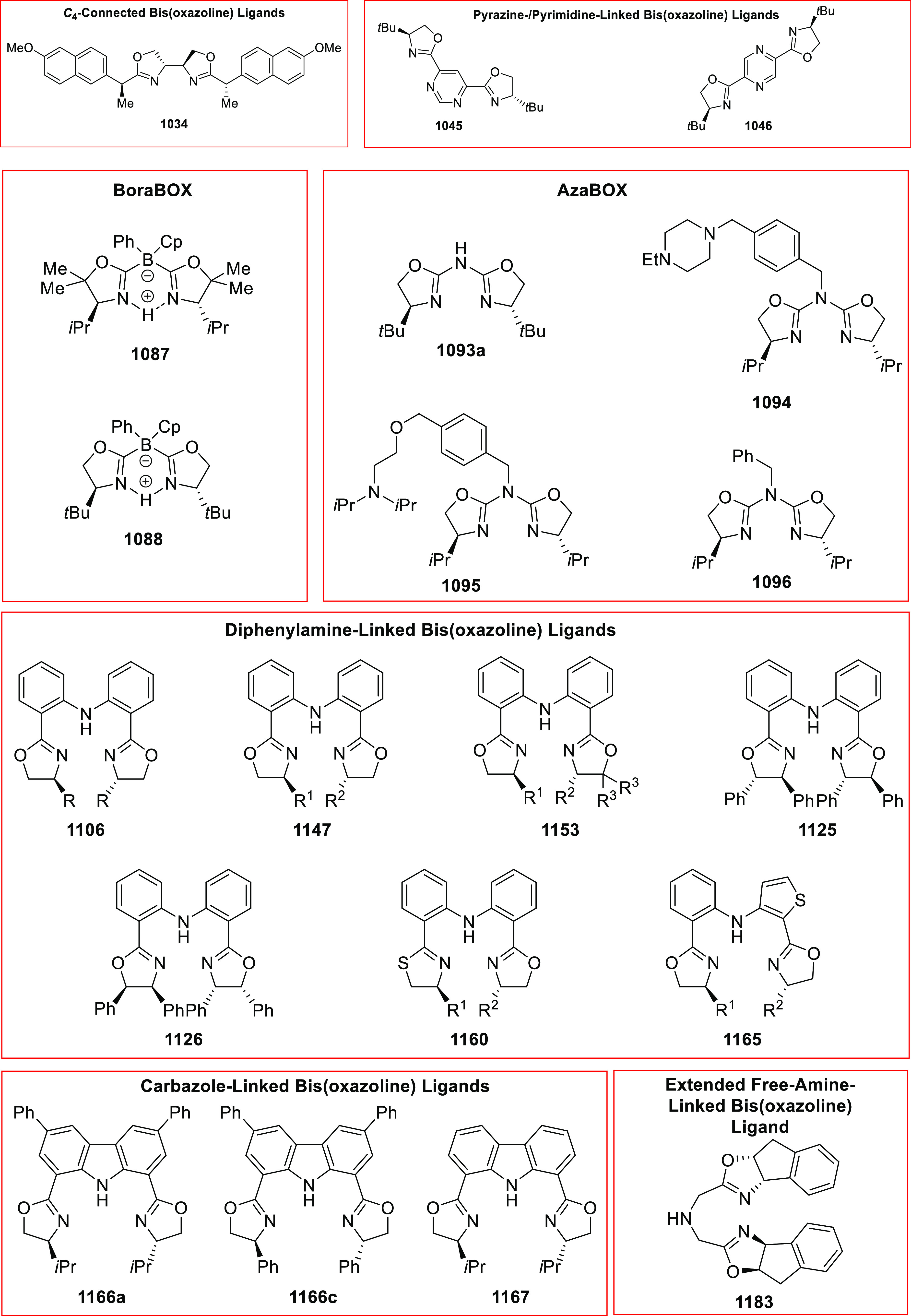

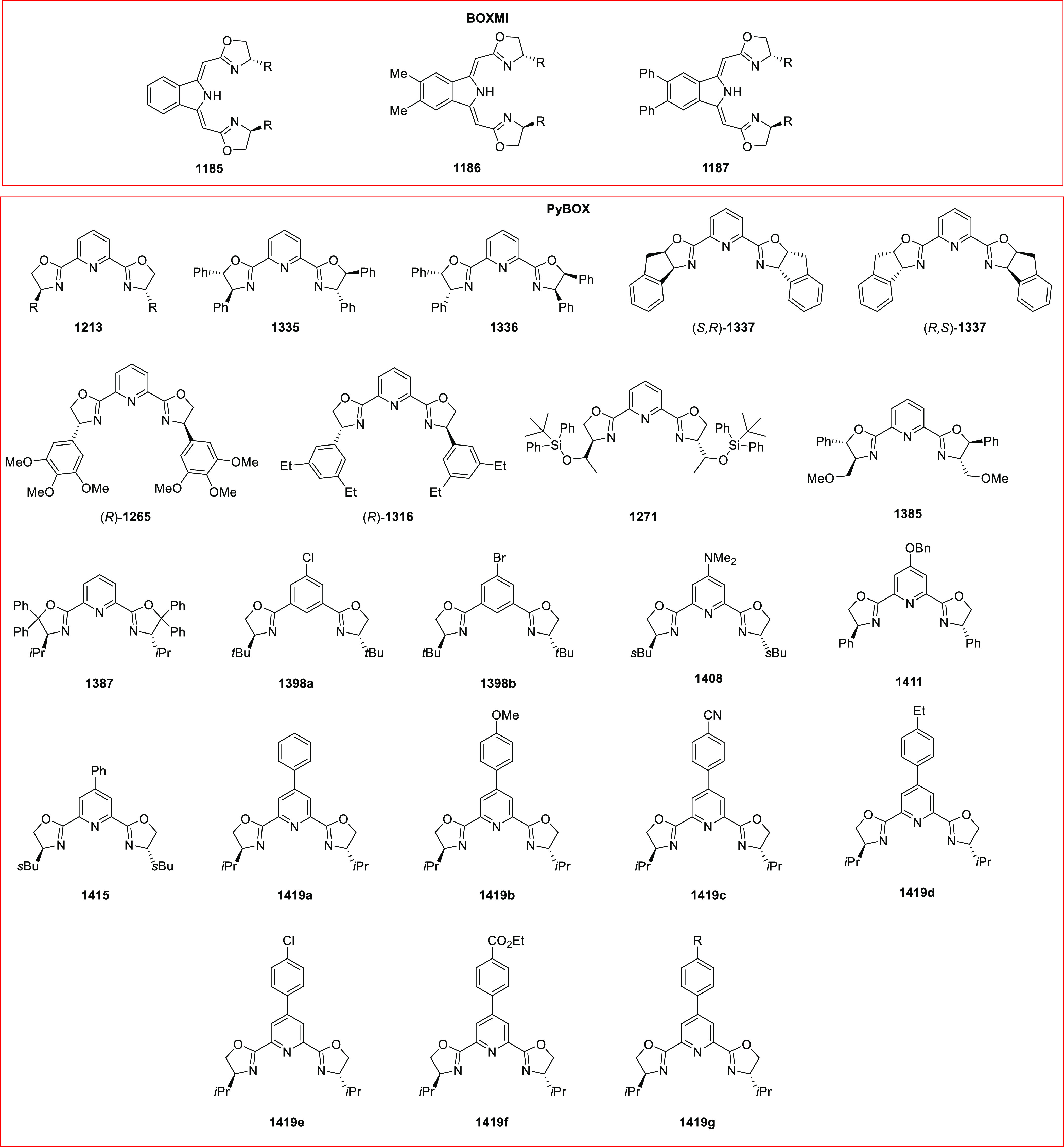

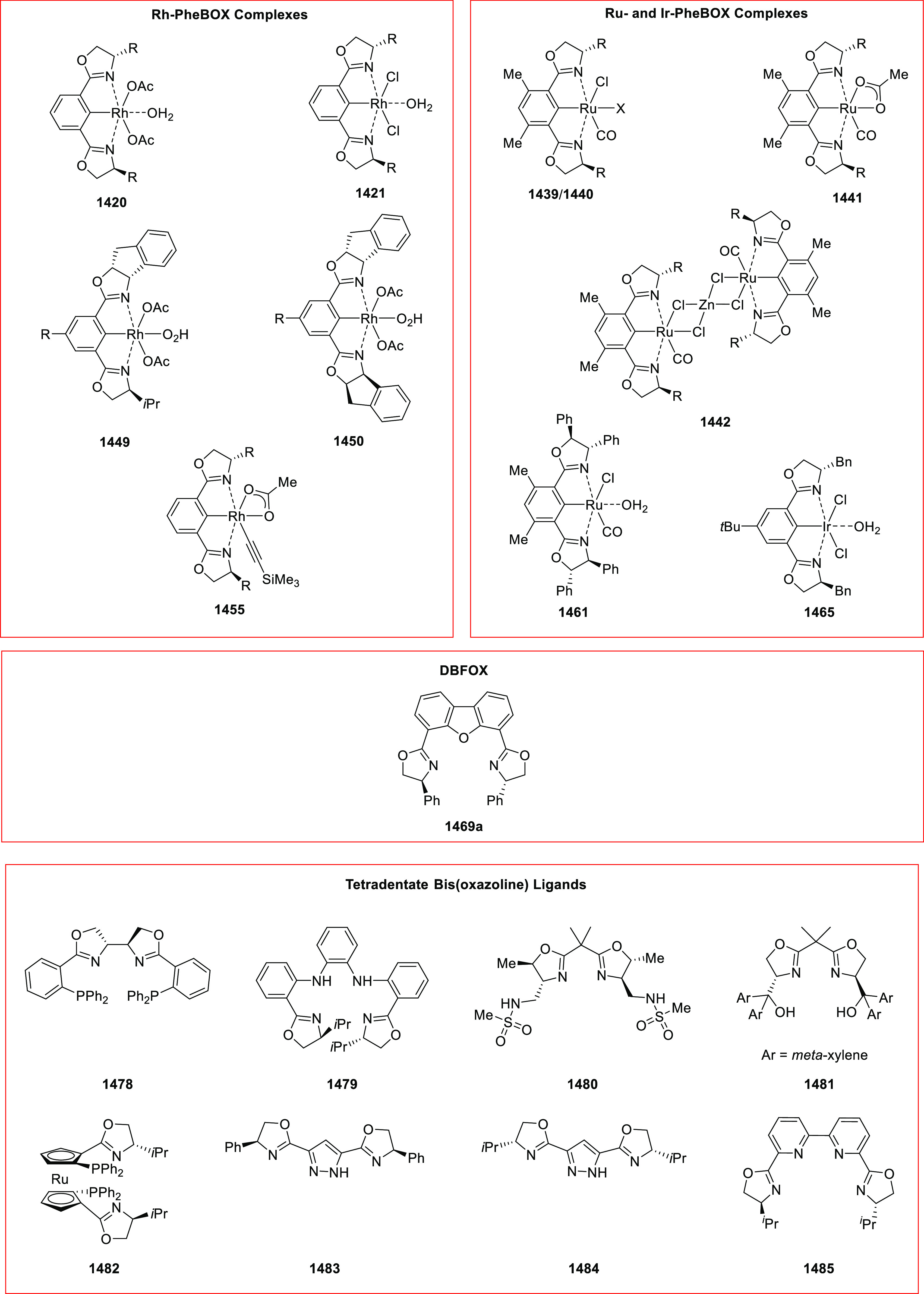

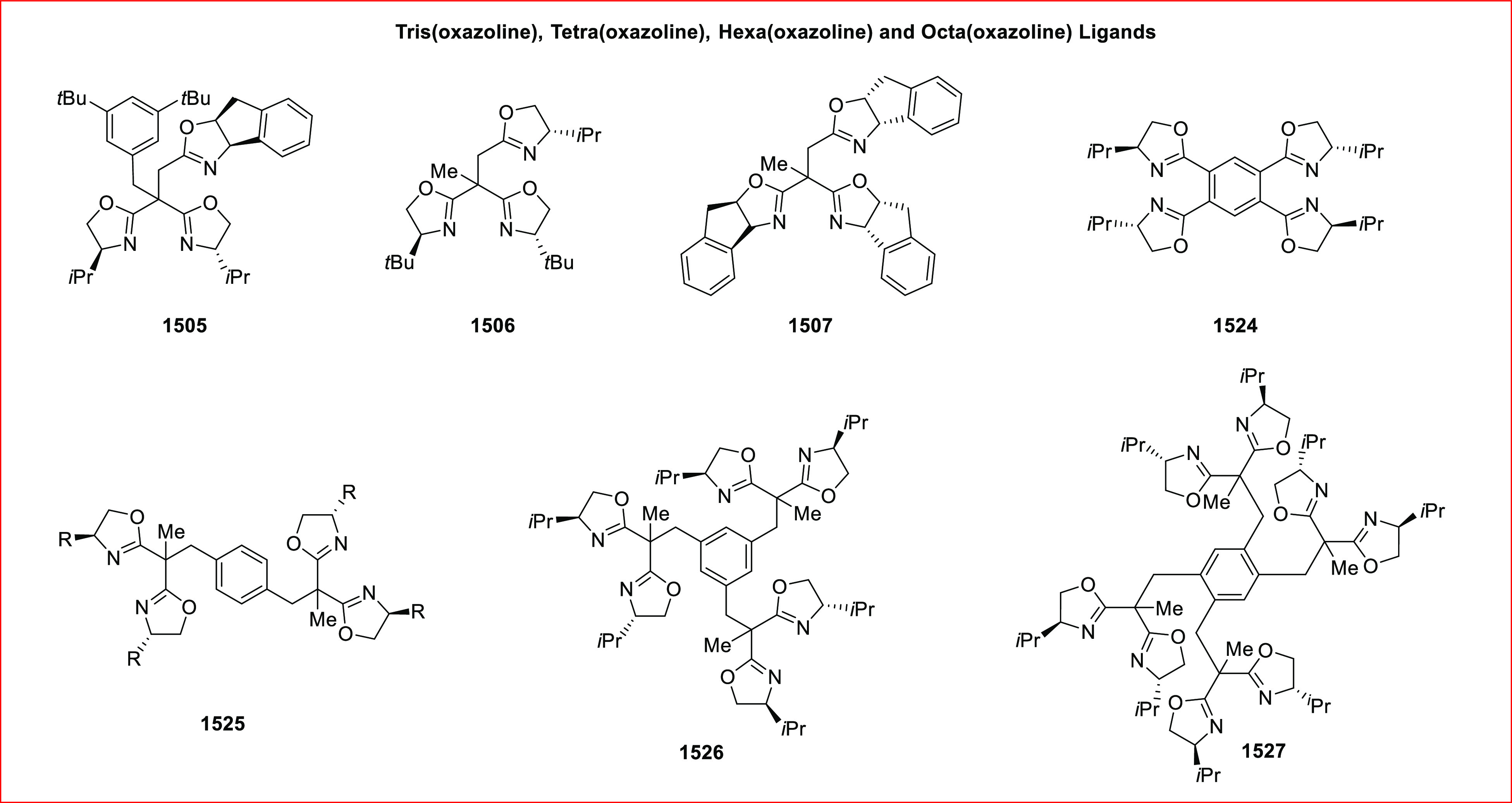
Master List of Ligands

## Dedication

Dedicated with respect and admiration to Dr. John Brown FRS, on the occasion of his 80th birthday, and with whom P.J.G. made his first bis(oxazoline) ligand in 1991.

## References

[ref1] Hargaden G. C.; Guiry P. J. Recent Applications of Oxazoline-Containing Ligands in Asymmetric Catalysis. Chem. Rev. 2009, 109, 2505–2550. 10.1021/cr800400z. 19378971

[ref2] McManus H. A.; Guiry P. J. Recent Developments in the Application of Oxazoline-Containing Ligands in Asymmetric Catalysis. Chem. Rev. 2004, 104, 4151–4202. 10.1021/cr040642v. 15352789

[ref3] Koch G.; Lloyd-Jones G. C.; Loiseleur O.; Pfaltz A.; Prétôt R.; Schaffner S.; Schnider P.; von Matt P. Synthesis of Chiral (Phosphinoaryl)Oxazolines, a Versatile Class of Ligands for Asymmetric Catalysis. Recl. des Trav. Chim. des Pays-Bas 1995, 114, 206–210. 10.1002/recl.19951140413.

[ref4] Helmchen G.; Pfaltz A. Phosphinooxazolines - A New Class of Versatile, Modular *P*,*N*-Ligands for Asymmetric Catalysis. Acc. Chem. Res. 2000, 33, 336–345. 10.1021/ar9900865. 10891051

[ref5] Tani K.; Behenna D. C.; McFadden R. M.; Stoltz B. M. A Facile and Modular Synthesis of Phosphinooxazoline Ligands. Org. Lett. 2007, 9, 2529–2531. 10.1021/ol070884s. 17536810

[ref6] Carroll M. P.; Guiry P. J. P,N Ligands in Asymmetric Catalysis. Chem. Soc. Rev. 2014, 43, 819–833. 10.1039/C3CS60302D. 24257099

[ref7] Behenna D. C.; Stoltz B. M. The Enantioselective Tsuji Allylation. J. Am. Chem. Soc. 2004, 126, 15044–15045. 10.1021/ja044812x. 15547998

[ref8] Trost B. M.; Xu J. Regio- and Enantioselective Pd-Catalyzed Allylic Alkylation of Ketones through Allyl Enol Carbonates. J. Am. Chem. Soc. 2005, 127, 2846–2847. 10.1021/ja043472c. 15740108

[ref9] James J.; Jackson M.; Guiry P. J. Palladium-Catalyzed Decarboxylative Asymmetric Allylic Alkylation: Development, Mechanistic Understanding and Recent Advances. Adv. Synth. Catal. 2019, 361, 3016–3049. 10.1002/adsc.201801575.

[ref10] Enquist J. A.; Stoltz B. M. The Total Synthesis of (−)-Cyanthiwigin F by Means of Double Catalytic Enantioselective Alkylation. Nature 2008, 453, 1228–1231. 10.1038/nature07046. 18580947 PMC2474750

[ref11] Enquist J. A.; Virgil S. C.; Stoltz B. M. Total Syntheses of Cyanthiwigins B, F, and G. Chem. - Eur. J. 2011, 17, 9957–9969. 10.1002/chem.201100425. 21769952 PMC3365662

[ref12] Kim K. E.; Stoltz B. M. A Second-Generation Synthesis of the Cyanthiwigin Natural Product Core. Org. Lett. 2016, 18, 5720–5723. 10.1021/acs.orglett.6b02962. 27762136

[ref13] Hong A. Y.; Krout M. R.; Jensen T.; Bennett N. B.; Harned A. M.; Stoltz B. M. Ring-Contraction Strategy for the Practical, Scalable, Catalytic Asymmetric Synthesis of Versatile γ-Quaternary Acylcyclopentenes. Angew. Chem., Int. Ed. 2011, 50, 2756–2760. 10.1002/anie.201007814. PMC3365661 21387482

[ref14] Reeves C. M.; Eidamshaus C.; Kim J.; Stoltz B. M. Enantioselective Construction of α-Quaternary Cyclobutanones by Catalytic Asymmetric Allylic Alkylation. Angew. Chem., Int. Ed. 2013, 52, 6718–6721. 10.1002/anie.201301815. PMC3817853 23686812

[ref15] Gartshore C. J.; Lupton D. W. Enantioselective Palladium-Catalyzed Decarboxylative Allylation of Carbazolones and Indolones: Formal Synthesis of (+)-KopsihainanineA. Angew. Chem., Int. Ed. 2013, 52, 4113–4116. 10.1002/anie.201209069. 23362227

[ref16] Li Z.; Zhang S.; Wu S.; Shen X.; Zou L.; Wang F.; Li X.; Peng F.; Zhang H.; Shao Z. Enantioselective Palladium-Catalyzed Decarboxylative Allylation of Carbazolones: Total Synthesis of (−)-Aspidospermidine and (+)-KopsihainanineA. Angew. Chem., Int. Ed. 2013, 52, 4117–4121. 10.1002/anie.201209878. 23483625

[ref17] Bennett N. B.; Duquette D. C.; Kim J.; Liu W. B.; Marziale A. N.; Behenna D. C.; Virgil S. C.; Stoltz B. M. Expanding Insight into Asymmetric Palladium-Catalyzed Allylic Alkylation of N-Heterocyclic Molecules and Cyclic Ketones. Chem. - Eur. J. 2013, 19, 4414–4418. 10.1002/chem.201300030. 23447555 PMC3815597

[ref18] Behenna D. C.; Liu Y.; Yurino T.; Kim J.; White D. E.; Virgil S. C.; Stoltz B. M. Enantioselective Construction of Quaternary N-Heterocycles by Palladium-Catalysed Decarboxylative Allylic Alkylation of Lactams. Nat. Chem. 2012, 4, 130–133. 10.1038/nchem.1222. PMC3266627 22270628

[ref19] Korch K. M.; Eidamshaus C.; Behenna D. C.; Nam S.; Horne D.; Stoltz B. M. Enantioselective Synthesis of α-Secondary and α-Tertiary Piperazin-2- Ones and Piperazines by Catalytic Asymmetric Allylic Alkylation. Angew. Chem., Int. Ed. 2015, 54, 179–183. 10.1002/anie.201408609. PMC4285707 25382664

[ref20] Numajiri Y.; Jiménez-Osés G.; Wang B.; Houk K. N.; Stoltz B. M. Enantioselective Synthesis of Dialkylated N-Heterocycles by Palladium-Catalyzed Allylic Alkylation. Org. Lett. 2015, 17, 1082–1085. 10.1021/ol503425t. 25714704 PMC6410707

[ref21] Rambla M.; Duroure L.; Chabaud L.; Guillou C. Enantioselective Synthesis of Spiroimines by Asymmetric Decarboxylative Alkylation/Isomerization/[3 + 2]-Cycloaddition Reaction of Azidoalkenes. Eur. J. Org. Chem. 2014, 2014, 7716–7720. 10.1002/ejoc.201403161.

[ref22] Pritchett B. P.; Kikuchi J.; Numajiri Y.; Stoltz B. M. Enantioselective Pd-Catalyzed Allylic Alkylation Reactions of Dihydropyrido[1,2-a]Indolone Substrates: Efficient Syntheses of (−)-Goniomitine, (+)-Aspidospermidine, and (−)-Quebrachamine. Angew. Chem., Int. Ed. 2016, 55, 13529–13532. 10.1002/anie.201608138. PMC5207349 27666731

[ref23] Lu Y.; Goldstein E. L.; Stoltz B. M. Palladium-Catalyzed Enantioselective Csp3-Csp3 Cross-Coupling for the Synthesis of (Poly)Fluorinated Chiral Building Blocks. Org. Lett. 2018, 20, 5657–5660. 10.1021/acs.orglett.8b02369. 30183315 PMC6192028

[ref24] Streuff J.; White D. E.; Virgil S. C.; Stoltz B. M. A Palladium-Catalysed Enolate Alkylation Cascade for the Formation of Adjacent Quaternary and Tertiary Stereocenters. Nat. Chem. 2010, 2, 192–196. 10.1038/nchem.518. 20697457 PMC2917108

[ref25] Bélanger É.; Houzé C.; Guimond N.; Cantin K.; Paquin J. F. Unexpected Effect of the Fluorine Atom on the Optimal Ligand-to-Palladium Ratio in the Enantioselective Pd-Catalyzed Allylation Reaction of Fluorinated Enol Carbonates. Chem. Commun. 2008, 1, 3251–3253. 10.1039/b803097a. 18622434

[ref26] White D. E.; Stewart I. C.; Grubbs R. H.; Stoltz B. M. The Catalytic Asymmetric Total Synthesis of Elatol. J. Am. Chem. Soc. 2008, 130, 810–811. 10.1021/ja710294k. 18163634 PMC2533138

[ref27] Day J. J.; McFadden R. M.; Virgil S. C.; Kolding H.; Alleva J. L.; Stoltz B. M. The Catalytic Enantioselective Total Synthesis of (+)-Liphagal. Angew. Chem., Int. Ed. 2011, 50, 6814–6818. 10.1002/anie.201101842. PMC3361906 21671325

[ref28] Mukherjee H.; McDougal N. T.; Virgil S. C.; Stoltz B. M. A Catalytic, Asymmetric Formal Synthesis of (+)-Hamigeran B. Org. Lett. 2011, 13, 825–827. 10.1021/ol102669z. 21271716 PMC3045637

[ref29] Zhang Z. W.; Wang C. C.; Xue H.; Dong Y.; Yang J. H.; Liu S.; Liu W. Q.; Li W. D. Z. Asymmetric Formal Synthesis of (−)-Cephalotaxine via Palladium-Catalyzed Enantioselective Tsuji Allylation. Org. Lett. 2018, 20, 1050–1053. 10.1021/acs.orglett.7b04008. 29400477

[ref30] Hanessian S.; Chénard E. A New Approach to the Synthesis of Peptidomimetic Renin Inhibitors: Palladium-Catalyzed Asymmetric Allylation of Acyclic Alkyl Aryl Ketones. Org. Lett. 2012, 14, 3222–3225. 10.1021/ol301332f. 22668074

[ref31] Alexy E. J.; Zhang H.; Stoltz B. M. Catalytic Enantioselective Synthesis of Acyclic Quaternary Centers: Palladium-Catalyzed Decarboxylative Allylic Alkylation of Fully Substituted Acyclic Enol Carbonates. J. Am. Chem. Soc. 2018, 140, 10109–10112. 10.1021/jacs.8b05560. 30049213 PMC6103296

[ref32] Seto M.; Roizen J. L.; Stoltz B. M. Catalytic Enantioselective Alkylation of Substituted Dioxanone Enol Ethers: Ready Access to C(α)-Tetrasubstituted Hydroxyketones, Acids, and Esters. Angew. Chem., Int. Ed. 2008, 47, 6873–6876. 10.1002/anie.200801424. PMC2762748 18651681

[ref33] Bélanger É.; Pouliot M. F.; Paquin J. F. Use of 5,5-(Dimethyl)-i-Pr-PHOX as a Practical Equivalent to t-Bu-PHOX in Asymmetric Catalysis. Org. Lett. 2009, 11, 2201–2204. 10.1021/ol9005618. 19388656

[ref34] Nahra F.; Macé Y.; Lambin D.; Riant O. Copper/Palladium-Catalyzed 1,4 Reduction and Asymmetric Allylic Alkylation of α,β-Unsaturated Ketones: Enantioselective Dual Catalysis. Angew. Chem., Int. Ed. 2013, 52, 3208–3212. 10.1002/anie.201208612. 23382027

[ref35] Grenning A. J.; Van Allen C. K.; Maji T.; Lang S. B.; Tunge J. A. Development of Asymmetric Deacylative Allylation. J. Org. Chem. 2013, 78, 7281–7287. 10.1021/jo400793a. 23734611 PMC3827786

[ref36] Keith J. A.; Behenna D. C.; Sherden N.; Mohr J. T.; Ma S.; Marinescu S. C.; Nielsen R. J.; Oxgaard J.; Stoltz B. M.; Goddard W. A. The Reaction Mechanism of the Enantioselective Tsuji Allylation: Inner-Sphere and Outer-Sphere Pathways, Internal Rearrangements, and Asymmetric C-C Bond Formation. J. Am. Chem. Soc. 2012, 134, 19050–19060. 10.1021/ja306860n. 23102088 PMC3537505

[ref37] Liu J.; Mishra S.; Aponick A. Enol Acetates: Versatile Substrates for the Enantioselective Intermolecular Tsuji Allylation. J. Am. Chem. Soc. 2018, 140, 16152–16158. 10.1021/jacs.8b08746. 30392366

[ref38] Craig R. A. II; Roizen J. L.; Smith R. C.; Jones A. C.; Virgil S. C.; Stoltz B. M. Enantioselective, Convergent Synthesis of the Ineleganolide Core by a Tandem Annulation Cascade. Chem. Sci. 2017, 8, 507–514. 10.1039/C6SC03347D. 28239443 PMC5321630

[ref39] Wang W.; Shen H.; Wan X. L.; Chen Q. Y.; Guo Y. Enantioselective Pd-Catalyzed Allylation of Acyclic α-Fluorinated Ketones. J. Org. Chem. 2014, 79, 6347–6353. 10.1021/jo500923u. 24915625

[ref40] Adamson N. J.; Hull E.; Malcolmson S. J. Enantioselective Intermolecular Addition of Aliphatic Amines to Acyclic Dienes with a Pd-PHOX Catalyst. J. Am. Chem. Soc. 2017, 139, 7180–7183. 10.1021/jacs.7b03480. 28453290 PMC5937019

[ref41] Park S.; Malcolmson S. J. Development and Mechanistic Investigations of Enantioselective Pd-Catalyzed Intermolecular Hydroaminations of Internal Dienes. ACS Catal. 2018, 8, 8468–8476. 10.1021/acscatal.8b01914.

[ref42] Adamson N. J.; Wilbur K. C. E.; Malcolmson S. J. Enantioselective Intermolecular Pd-Catalyzed Hydroalkylation of Acyclic 1,3-Dienes with Activated Pronucleophiles. J. Am. Chem. Soc. 2018, 140, 2761–2764. 10.1021/jacs.7b13300. 29446922 PMC5937024

[ref43] Balaraman K.; Wolf C. Catalytic Enantioselective and Diastereoselective Allylic Alkylation with Fluoroenolates: Efficient Access to C3-Fluorinated and All-Carbon Quaternary Oxindoles. Angew. Chem., Int. Ed. 2017, 56, 1390–1395. 10.1002/anie.201608752. PMC5289271 28026079

[ref44] Cheng Q.; Zhang H. J.; Yue W. J.; You S. L. Palladium-Catalyzed Highly Stereoselective Dearomative [3 + 2] Cycloaddition of Nitrobenzofurans. Chem. 2017, 3, 428–436. 10.1016/j.chempr.2017.06.015.

[ref45] Cheng Q.; Zhang F.; Cai Y.; Guo Y. L.; You S. L. Stereodivergent Synthesis of Tetrahydrofuroindoles through Pd-Catalyzed Asymmetric Dearomative Formal [3 + 2] Cycloaddition. Angew. Chem., Int. Ed. 2018, 57, 2134–2138. 10.1002/anie.201711873. 29330914

[ref46] Suetsugu S.; Nishiguchi H.; Tsukano C.; Takemoto Y. Asymmetric Synthesis of (−)-Aurantioclavine via Palladium-Catalyzed Intramolecular Allylic Amination. Org. Lett. 2014, 16, 996–999. 10.1021/ol4037314. 24460216

[ref47] Linton E. C.; Kozlowski M. C. Catalytic Enantioselective Meerwein-Eschenmoser Claisen Rearrangement: Asymmetric Synthesis of Allyl Oxindoles. J. Am. Chem. Soc. 2008, 130, 16162–16163. 10.1021/ja807026z. 18998679

[ref48] Cao T.; Deitch J.; Linton E. C.; Kozlowski M. C. Asymmetric Synthesis of Allenyl Oxindoles and Spirooxindoles by a Catalytic Enantioselective Saucy-Marbet Claisen Rearrangement. Angew. Chem., Int. Ed. 2012, 51, 2448–2451. 10.1002/anie.201107417. PMC4118689 22287059

[ref49] Kingston C.; James J.; Guiry P. J. Development of and Recent Advances in Pd-Catalyzed Decarboxylative Asymmetric Protonation. J. Org. Chem. 2019, 84, 473–485. 10.1021/acs.joc.8b02478. 30376624

[ref50] Marinescu S. C.; Nishimata T.; Mohr J. T.; Stoltz B. M. Homogeneous Pd-Catalyzed Enantioselective Decarboxylative Protonation. Org. Lett. 2008, 10, 1039–1042. 10.1021/ol702821j. 18303896 PMC2966305

[ref51] Zhao R.; Sun Z.; Mo M.; Peng F.; Shao Z. Catalytic Asymmetric Assembly of C3-Monosubstituted Chiral Carbazolones and Concise Formal Synthesis of (−)-Aspidofractinine: Application of Enantioselective Pd-Catalyzed Decarboxylative Protonation of Carbazolones. Org. Lett. 2014, 16, 4178–4181. 10.1021/ol501877x. 25060656

[ref52] Carroll M. P.; Müller-Bunz H.; Guiry P. J. Enantioselective Construction of Sterically Hindered Tertiary α-Aryl Ketones: A Catalytic Asymmetric Synthesis of Isoflavanones. Chem. Commun. 2012, 48, 11142–11144. 10.1039/c2cc36452b. 23042451

[ref53] Doran R.; Carroll M. P.; Akula R.; Hogan B. F.; Martins M.; Fanning S.; Guiry P. J. A Stereoselective Switch: Enantiodivergent Approach to the Synthesis of Isoflavanones. Chem. - Eur. J. 2014, 20, 15354–15359. 10.1002/chem.201405246. 25314579

[ref54] Doran R.; Guiry P. J. Catalytic Asymmetric Synthesis of Sterically Hindered Tertiary α-Aryl Ketones. J. Org. Chem. 2014, 79, 9112–9124. 10.1021/jo5014806. 25233274

[ref55] Kingston C.; Guiry P. J. Enantiodivergent Synthesis of Tertiary α-Aryl 1-Indanones: Evidence Toward Disparate Mechanisms in the Palladium-Catalyzed Decarboxylative Asymmetric Protonation. J. Org. Chem. 2017, 82, 3806–3819. 10.1021/acs.joc.7b00303. 28345917

[ref56] Lu S. M.; Bolm C. Highly Enantioselective Synthesis of Optically Active Ketones by Iridium-Catalyzed Asymmetric Hydrogenation. Angew. Chem., Int. Ed. 2008, 47, 8920–8923. 10.1002/anie.200803709. 18855959

[ref57] Lu W. J.; Chen Y. W.; Hou X. L. Iridium-Catalyzed Highly Enantioselective Hydrogenation of the C=C Bond of α, β-Unsaturated Ketones. Angew. Chem., Int. Ed. 2008, 47, 10133–10136. 10.1002/anie.200803872. 19021171

[ref58] Zheng Z.; Cao Y.; Chong Q.; Han Z.; Ding J.; Luo C.; Wang Z.; Zhu D.; Zhou Q. L.; Ding K. Chiral Cyclohexyl-Fused Spirobiindanes: Practical Synthesis, Ligand Development, and Asymmetric Catalysis. J. Am. Chem. Soc. 2018, 140, 10374–10381. 10.1021/jacs.8b07125. 30036064

[ref59] Baeza A.; Pfaltz A. Iridium-Catalyzed Asymmetric Hydrogenation of Unfunctionalized Enamines. Chem. - Eur. J. 2009, 15, 2266–2269. 10.1002/chem.200802576. 19177480

[ref60] Weise C. F.; Pischl M.; Pfaltz A.; Schneider C. A Non-Iterative, Flexible, and Highly Stereoselective Synthesis of Polydeoxypropionates—Synthesis of (+)-Vittatalactone. Chem. Commun. 2011, 47, 3248–3250. 10.1039/c0cc05215a. 21279191

[ref61] Pischl M. C.; Weise C. F.; Haseloff S.; Müller M. A.; Pfaltz A.; Schneider C. A Highly Stereoselective and Flexible Strategy for the Convergent Synthesis of Long-Chain Polydeoxypropionates: Application towards the Synthesis of the Glycolipid Membrane Components Hydroxyphthioceranic and Phthioceranic Acid. Chem. - Eur. J. 2014, 20, 17360–17374. 10.1002/chem.201404034. 25351748

[ref62] Lu W. J.; Chen Y. W.; Hou X. L. Highly Enantioselective Iridium-Catalyzed Hydrogenation of Trisubstituted Olefins, α,β-Unsaturated Ketones and Imines with Chiral Benzylic Substituted P,N Ligands. Adv. Synth. Catal. 2010, 352, 103–107. 10.1002/adsc.200900618.

[ref63] Schrems M. G.; Pfaltz A. NeoPHOX - An Easily Accessible *P*,*N*-Ligand for Iridium-Catalyzed Asymmetric Hydrogenation: Preparation, Scope and Application in the Synthesis of Demethyl Methoxycalamenene. Chem. Commun. 2009, (41), 6210–6212. 10.1039/b912680e. 19826671

[ref64] Baeza A.; Pfaltz A. Iridium-Catalyzed Asymmetric Hydrogenation of N-Protected Indoles. Chem. - Eur. J. 2010, 16, 2036–2039. 10.1002/chem.200903105. 20104554

[ref65] Ikeda R.; Kuwano R. Asymmetric Hydrogenation of Isoxazolium Triflates with a Chiral Iridium Catalyst. Chem. - Eur. J. 2016, 22, 8610–8618. 10.1002/chem.201600732. 27105605

[ref66] Clarke C.; Incerti-Pradillos C. A.; Lam H. W. Enantioselective Nickel-Catalyzed Anti-Carbometallative Cyclizations of Alkynyl Electrophiles Enabled by Reversible Alkenylnickel E/Z Isomerization. J. Am. Chem. Soc. 2016, 138, 8068–8071. 10.1021/jacs.6b04206. 27333360 PMC4957849

[ref67] Karad S. N.; Panchal H.; Clarke C.; Lewis W.; Lam H. W. Enantioselective Synthesis of Chiral Cyclopent-2-Enones by Nickel-Catalyzed Desymmetrization of Malonate Esters. Angew. Chem., Int. Ed. 2018, 57, 9122–9125. 10.1002/anie.201805578. PMC6485403 29768708

[ref68] Panchal H.; Clarke C.; Bell C.; Karad S. N.; Lewis W.; Lam H. W. Nickel-Catalyzed, Ligand-Free, Diastereoselective Synthesis of 3-Methyleneindan-1-Ols. Chem. Commun. 2018, 54, 12389–12392. 10.1039/C8CC06388E. 30328418

[ref69] Chen M. H.; Hsieh J. C.; Lee Y. H.; Cheng C. H. Controlled Synthesis of Enantioselective 1-Aminoindenes via Cobalt-Catalyzed [3 + 2] Annulation Reaction. ACS Catal. 2018, 8, 9364–9369. 10.1021/acscatal.8b02566.

[ref70] Nguyen T. L. N.; Incerti-Pradillos C. A.; Lewis W.; Lam H. W. Enantioselective Nickel-Catalyzed Arylative Intramolecular 1,4-Allylations. Chem. Commun. 2018, 54, 5622–5625. 10.1039/C8CC03204A. 29770399

[ref71] Zhou B.; Jiang C.; Gandi V. R.; Lu Y.; Hayashi T. Palladium-Catalyzed Asymmetric Arylation of Trifluoromethylated/Perfluoroalkylated 2-Quinazolinones with High Enantioselectivity. Chem. - Eur. J. 2016, 22, 13068–13071. 10.1002/chem.201603105. 27377667

[ref72] Yan Z.; Wu B.; Gao X.; Zhou Y. G. Enantioselective Synthesis of Quaternary α-Aminophosphonates by Pd-Catalyzed Arylation of Cyclic α-Ketiminophosphonates with Arylboronic Acids. Chem. Commun. 2016, 52, 10882–10885. 10.1039/C6CC04096A. 27530154

[ref73] Chen W.; Meng D.; N’Zemba B.; Morris W. J. Palladium-Catalyzed Enantioselective Synthesis of Cyclic Sulfamidates and Application to a Synthesis of Verubecestat. Org. Lett. 2018, 20, 1265–1268. 10.1021/acs.orglett.7b03639. 29461065

[ref74] Mazet C.; Gérard D. Highly Regio- and Enantioselective Catalytic Asymmetric Hydroboration of α-Substituted Styrenyl Derivatives. Chem. Commun. 2011, 47, 298–300. 10.1039/C0CC01547D. 20697639

[ref75] García-Fortanet J.; Buchwald S. L. Asymmetric Palladium-Catalyzed Intramolecular α-Arylation of Aldehydes. Angew. Chem., Int. Ed. 2008, 47, 8108–8111. 10.1002/anie.200803809. PMC2748771 18792905

[ref76] Pagar V. V.; RajanBabu T. V. Tandem Catalysis for Asymmetric Coupling of Ethylene and Enynes to Functionalized Cyclobutanes. Science 2018, 361, 68–72. 10.1126/science.aat6205. 29976822 PMC6055924

[ref77] Dounay A. B.; Humphreys P. G.; Overman L. E.; Wrobleski A. D. Total Synthesis of the Strychnos Alkaloid (+)-Minfiensine: Tandem Enantioselective Intramolecular Heck-Iminium Ion Cyclization. J. Am. Chem. Soc. 2008, 130, 5368–5377. 10.1021/ja800163v. 18303837 PMC2995331

[ref78] Fitzpatrick M. O.; Muller-Bunz H.; Guiry P. J. The Synthesis of New HetPHOX Ligands and Their Application to the Intermolecular Asymmetric Heck Reaction. Eur. J. Org. Chem. 2009, 2009, 1889–1895. 10.1002/ejoc.200800761.

[ref79] Kong W.; Wang Q.; Zhu J. Water as a Hydride Source in Palladium-Catalyzed Enantioselective Reductive Heck Reactions. Angew. Chem., Int. Ed. 2017, 56, 3987–3991. 10.1002/anie.201700195. 28272769

[ref80] Jiang Z.; Hou L.; Ni C.; Chen J.; Wang D.; Tong X. Enantioselective Construction of Quaternary Tetrahydropyridines by Palladium-Catalyzed Vinylborylation of Alkenes. Chem. Commun. 2017, 53, 4270–4273. 10.1039/C7CC01488K. 28361147

[ref81] Hou L.; Yuan Y.; Tong X. Pd(0)-Catalysed Asymmetric Reductive Heck-Type Cyclization of (: Z)-1-Iodo-1,6-Dienes and Enantioselective Synthesis of Quaternary Tetrahydropyridines. Org. Biomol. Chem. 2017, 15, 4803–4806. 10.1039/C7OB00762K. 28548157

[ref82] Babu K. N.; Kinthada L. K.; Pratim Das P.; Bisai A. Cu(II)-TBu-PHOX Catalyzed Enantioselective Malonate Addition onto 3-Hydroxy 2-Oxindoles: Application in the Synthesis of Dimeric Pyrroloindoline Alkaloids. Chem. Commun. 2018, 54, 7963–7966. 10.1039/C8CC04338H. 29956704

[ref83] Cook M. J.; Rovis T. Enantioselective Rhodium-Catalyzed Alkylative Desymmetrization of 3,5-Dimethylglutaric Anhydride. Synthesis 2009, 2009 (2), 335–338. 10.1055/s-0028-1083275.

[ref84] Shin M.; Gu M.; Lim S. S.; Kim M.-J.; Lee J.; Jin H.; Jang Y. H.; Jung B. Cu I -Catalysed Enantioselective Alkyl 1,4-Additions to (E)-Nitroalkenes and Cyclic Enones with Phosphino-Oxazoline Ligands. Eur. J. Org. Chem. 2018, 2018, 3122–3130. 10.1002/ejoc.201800476.

[ref85] Liu Y.; Zhang W. Iridium-Catalyzed Asymmetric Hydrogenation of α-Alkylidene Succinimides. Angew. Chem., Int. Ed. 2013, 52, 2203–2206. 10.1002/anie.201209126. 23335151

[ref86] Xia J.; Yang G.; Zhuge R.; Liu Y.; Zhang W. Iridium-Catalyzed Asymmetric Hydrogenation of Unfunctionalized Exocyclic C=C Bonds. Chem. - Eur. J. 2016, 22, 18354–18357. 10.1002/chem.201604298. 27770544

[ref87] Xia J.; Nie Y.; Yang G.; Liu Y.; Zhang W. Iridium-Catalyzed Asymmetric Hydrogenation of 2*H*-Chromenes: A Highly Enantioselective Approach to Isoflavan Derivatives. Org. Lett. 2017, 19, 4884–4887. 10.1021/acs.orglett.7b02341. 28857571

[ref88] Meng K.; Xia J.; Wang Y.; Zhang X.; Yang G.; Zhang W. Ir/BiphPHOX-Catalyzed Asymmetric Hydrogenation of 3-Substituted 2,5-Dihydropyrroles and 2,5-Dihydrothiophene 1,1-Dioxides. Org. Chem. Front. 2017, 4, 1601–1605. 10.1039/C7QO00248C.

[ref89] Quan M.; Tang L.; Shen J.; Yang G.; Zhang W. Ni(Ii)-Catalyzed Asymmetric Addition of Arylboronic Acids to Cyclic Imines. Chem. Commun. 2017, 53, 609–612. 10.1039/C6CC08759K. 27981326

[ref90] Wang Q.; Zhang Z.; Chen C.; Yang H.; Han Z.; Dong X. Q.; Zhang X. Iridium Catalysts with Modular Axial-Unfixed Biphenyl Phosphine-Oxazoline Ligands: Asymmetric Hydrogenation of α,β-Unsaturated Carboxylic Acids. Org. Chem. Front. 2017, 4, 627–630. 10.1039/C6QO00677A.

[ref91] Song S.; Zhu S. F.; Li Y.; Zhou Q. L. Iridium-Catalyzed Enantioselective Hydrogenation of α,β- Unsaturated Carboxylic Acids with Tetrasubstituted Olefins. Org. Lett. 2013, 15, 3722–3725. 10.1021/ol401593a. 23822146

[ref92] Song S.; Zhu S. F.; Yu Y. B.; Zhou Q. L. Carboxy-Directed Asymmetric Hydrogenation of 1,1-Diarylethenes and 1,1-Dialkylethenes. Angew. Chem., Int. Ed. 2013, 52, 1556–1559. 10.1002/anie.201208606. 23255224

[ref93] Race N. J.; Faulkner A.; Fumagalli G.; Yamauchi T.; Scott J. S.; Rydén-Landergren M.; Sparkes H. A.; Bower J. F. Enantioselective Narasaka-Heck Cyclizations: Synthesis of Tetrasubstituted Nitrogen-Bearing Stereocenters. Chem. Sci. 2017, 8, 1981–1985. 10.1039/C6SC04466B. 28451314 PMC5390761

[ref94] Wang Y. N.; Yang L. C.; Rong Z. Q.; Liu T. L.; Liu R.; Zhao Y. Pd-Catalyzed Enantioselective [6 + 4] Cycloaddition of Vinyl Oxetanes with Azadienes to Access Ten-Membered Heterocycles. Angew. Chem., Int. Ed. 2018, 57, 1596–1600. 10.1002/anie.201711648. 29265722

[ref95] Sun W.; Gu H.; Lin X. Synthesis and Application of Hexamethyl-1,1′-Spirobiindane-Based Phosphine-Oxazoline Ligands in Ni-Catalyzed Asymmetric Arylation of Cyclic Aldimines. J. Org. Chem. 2018, 83, 4034–4043. 10.1021/acs.joc.8b00422. 29554420

[ref96] Zhang Y.; Han Z.; Li F.; Ding K.; Zhang A. Highly Enantioselective Hydrogenation of α-Aryl-β-Substituted Acrylic Acids Catalyzed by Ir-SpinPHOX. Chem. Commun. 2010, 46, 156–158. 10.1039/B919902K. 20024325

[ref97] Liu X.; Han Z.; Wang Z.; Ding K. SpinPhox/Iridium(I)-Catalyzed Asymmetric Hydrogenation of Cyclic α-Alkylidene Carbonyl Compounds. Angew. Chem., Int. Ed. 2014, 53, 1978–1982. 10.1002/anie.201309521. 24446418

[ref98] Ge Y.; Han Z.; Wang Z.; Feng C. G.; Zhao Q.; Lin G. Q.; Ding K. Ir-SpinPHOX Catalyzed Enantioselective Hydrogenation of 3-Ylidenephthalides. Angew. Chem., Int. Ed. 2018, 57, 13140–13144. 10.1002/anie.201807639. 30129227

[ref99] Qiu Z.; Sun R.; Teng D. Synthesis of Highly Rigid Phosphine-Oxazoline Ligands for Palladium-Catalyzed Asymmetric Allylic Alkylation. Org. Biomol. Chem. 2018, 16, 7717–7724. 10.1039/C8OB02265H. 30289146

[ref100] Gao Y.; Qiu Z.; Sun R.; Gao N.; Cao G.; Teng D. New Spiro Phosphinooxazolines for Palladium-Catalyzed Asymmetric Allylic Amination. Tetrahedron Lett. 2018, 59, 3938–3941. 10.1016/j.tetlet.2018.09.044.

[ref101] Nishibayashi Y.; Uemura S. Asymmetric Synthesis and Highly Diastereoselective Ortho-Lithiation of Oxazolinylferrocenes. Synlett 1995, 1995, 79–81. 10.1055/s-1995-4881.

[ref102] Tong M. C.; Chen X.; Li J.; Huang R.; Tao H.; Wang C. J. Catalytic Asymmetric Synthesis of [2,3]-Fused Indoline Heterocycles through Inverse-Electron-Demand Aza-Diels-Alder Reaction of Indoles with Azoalkenes. Angew. Chem., Int. Ed. 2014, 53, 4680–4684. 10.1002/anie.201400109. 24668693

[ref103] Wei L.; Yao L.; Wang Z. F.; Li H.; Tao H. Y.; Wang C. J. Copper(I)-Catalyzed Asymmetric 1,3-Dipolar [3 + 4] Cycloaddition of Nitrones with Azoalkenes. Adv. Synth. Catal. 2016, 358, 3748–3752. 10.1002/adsc.201600457.

[ref104] Yang W. L.; Li C. Y.; Qin W. J.; Tang F. F.; Yu X.; Deng W. P. Cu(I)-Catalyzed Chemoselective and Stereoselective [3 + 3] Cycloaddition of Azomethine Ylides with 2-Indolylnitroethylenes: Facile Access to Highly Substituted Tetrahydro-γ-Carbolines. ACS Catal. 2016, 6, 5685–5690. 10.1021/acscatal.6b01596.

[ref105] Liu Y. Z.; Shang S. J.; Zhu J. Y.; Yang W. L.; Deng W. P. Regioselective and Stereoselective [3 + 3] Annulation of Ketones Derived Azomethine Ylides with 2-Indolylethylenes: Direct Access to Highly Substituted Tetrahydro-γ-Carbolines. Adv. Synth. Catal. 2018, 360, 2191–2203. 10.1002/adsc.201800274.

[ref106] Yang Q. L.; Xie M. S.; Xia C.; Sun H. L.; Zhang D. J.; Huang K. X.; Guo Z.; Qu G. R.; Guo H. M. A Rapid and Divergent Access to Chiral Azacyclic Nucleoside Analogues via Highly Enantioselective 1,3-Dipolar Cycloaddition of β-Nucleobase Substituted Acrylates. Chem. Commun. 2014, 50, 14809–14812. 10.1039/C4CC06632D. 25319349

[ref107] Tang L. W.; Zhao B. J.; Dai L.; Zhang M.; Zhou Z. M. Asymmetric Construction of Pyrrolidines Bearing a Trifluoromethylated Quaternary Stereogenic Center via CuI-Catalyzed 1,3-Dipolar Cycloaddition of Azomethine Ylides with β-CF3-β,β-Disubstituted Nitroalkenes. Chem. - Asian J. 2016, 11, 2470–2477. 10.1002/asia.201600941. 27459478

[ref108] Liu B.; Zhang Z. M.; Xu B.; Xu S.; Wu H. H.; Liu Y.; Zhang J. Cu(i)-Catalyzed Michael Addition of Ketiminoesters to β-Trifluoromethyl β,β-Disubstituted Enones: Rapid Access to 1-Pyrrolines Bearing a Quaternary All-Carbon Stereocenter. Org. Chem. Front. 2017, 4, 1772–1776. 10.1039/C7QO00291B.

[ref109] He F. S.; Li C. S.; Deng H.; Zheng X.; Yang Z. T.; Deng W. P. The Facile and Stereoselective Synthesis of Pyrrolidine β-Amino Acids: Via Copper(i)-Catalyzed Asymmetric 1,3-Dipolar Cycloaddition. Org. Chem. Front. 2017, 4, 52–56. 10.1039/C6QO00512H.

[ref110] Deng H.; Yang W. L.; Tian F.; Tang W.; Deng W. P. Asymmetric Construction of 3-Azabicyclo[3.1.0]Hexane Skeleton with Five Contiguous Stereogenic Centers by Cu-Catalyzed 1,3-Dipolar Cycloaddition of Trisubstituted Cyclopropenes. Org. Lett. 2018, 20, 4121–4125. 10.1021/acs.orglett.8b01686. 29943995

[ref111] Xu S.; Zhang Z. M.; Xu B.; Liu B.; Liu Y.; Zhang J. Enantioselective Regiodivergent Synthesis of Chiral Pyrrolidines with Two Quaternary Stereocenters via Ligand-Controlled Copper(I)-Catalyzed Asymmetric 1,3-Dipolar Cycloadditions. J. Am. Chem. Soc. 2018, 140, 2272–2283. 10.1021/jacs.7b12137. 29303569

[ref112] Ma C.; Huang Y.; Zhao Y. Stereoselective 1,6-Conjugate Addition/Annulation of Para-Quinone Methides with Vinyl Epoxides/Cyclopropanes. ACS Catal. 2016, 6, 6408–6412. 10.1021/acscatal.6b01845.

[ref113] Yamauchi M.; Morimoto M.; Miura T.; Murakami M. Enantioselective Synthesis of 3,4-Dihydroisoquinolin-1(2H)-Ones by Nickel-Catalyzed Denitrogenative Annulation of 1,2,3-Benzotriazin-4(3H)-Ones with Allenes. J. Am. Chem. Soc. 2010, 132, 54–55. 10.1021/ja909603j. 20000760

[ref114] Miura T.; Morimoto M.; Murakami M. Enantioselective [2 + 2 + 2] Cycloaddition Reaction of Isocyanates and Allenes Catalyzed by Nickel. J. Am. Chem. Soc. 2010, 132, 15836–15838. 10.1021/ja105541r. 20839803

[ref115] Ochi Y.; Kurahashi T.; Matsubara S. Decarbonylative Cycloaddition of Phthalic Anhydrides with Allenes. Org. Lett. 2011, 13, 1374–1377. 10.1021/ol200044y. 21332138

[ref116] Hernandez L. W.; Pospech J.; Klöckner U.; Bingham T. W.; Sarlah D. Synthesis of (+)-Pancratistatins via Catalytic Desymmetrization of Benzene. J. Am. Chem. Soc. 2017, 139, 15656–15659. 10.1021/jacs.7b10351. 29059521 PMC5960067

[ref117] Hernandez L. W.; Klöckner U.; Pospech J.; Hauss L.; Sarlah D. Nickel-Catalyzed Dearomative Trans −1,2-Carboamination. J. Am. Chem. Soc. 2018, 140, 4503–4507. 10.1021/jacs.8b01726. 29544244 PMC5971658

[ref118] Okumura M.; Shved A. S.; Sarlah D. Palladium-Catalyzed Dearomative Syn-1,4-Carboamination. J. Am. Chem. Soc. 2017, 139, 17787–17790. 10.1021/jacs.7b11663. 29183109 PMC5971112

[ref119] Strohmeier M.; Leach K.; Zajac M. A. Asymmetric Conjugate Addition of Glycine Derivatives under Copper Catalysis. Angew. Chem., Int. Ed. 2011, 50, 12335–12338. 10.1002/anie.201105258. 22031124

[ref120] Li Q.; Ding C. H.; Hou X. L.; Dai L. X. Diastereo- And Enantioselective Synthesis of α,γ-Diaminobutyric Acid Derivatives via Cu-Catalyzed Asymmetric Michael Reaction. Org. Lett. 2010, 12, 1080–1083. 10.1021/ol100060t. 20143857

[ref121] Chen C. G.; Hou X. L.; Pu L. Highly Enantioselective Cu-Catalyzed Conjugate Addition-Elimination of Activated Ally Lie Acetates with Glycine Derivatives. Org. Lett. 2009, 11, 2073–2075. 10.1021/ol900439h. 19358567

[ref122] He Z. T.; Zhao Y. S.; Tian P.; Wang C. C.; Dong H. Q.; Lin G. Q. Copper-Catalyzed Asymmetric Hydroboration of α-Dehydroamino Acid Derivatives: Facile Synthesis of Chiral β-Hydroxy-α-Amino Acids. Org. Lett. 2014, 16, 1426–1429. 10.1021/ol500219e. 24528372

[ref123] He F. S.; Jin J. H.; Yang Z. T.; Yu X.; Fossey J. S.; Deng W. P. Direct Asymmetric Synthesis of β-Bis-Aryl-α-Amino Acid Esters via Enantioselective Copper-Catalyzed Addition of p-Quinone Methides. ACS Catal. 2016, 6, 652–656. 10.1021/acscatal.5b02619.

[ref124] Ma Z.; Xie F.; Yu H.; Zhang Y.; Wu X.; Zhang W. Copper-Catalyzed Asymmetric 1,4-Conjugate Addition of Grignard Reagents to Linear α,β,γ,δ-Unsaturated Ketones. Chem. Commun. 2013, 49, 5292–5294. 10.1039/c3cc42088d. 23648997

[ref125] Zhu J. Y.; Yang W. L.; Liu Y. Z.; Shang S. J.; Deng W. P. A Copper(i)-Catalyzed Asymmetric Mannich Reaction of Glycine Schiff Bases with Isatin-Derived Ketimines: Enantioselective Synthesis of 3-Substituted 3-Aminooxindoles. Org. Chem. Front. 2018, 5, 70–74. 10.1039/C7QO00691H.

[ref126] Quan M.; Butt N.; Shen J.; Shen K.; Liu D.; Zhang W. The Synthesis of Chiral β-Aryl-α,β-Unsaturated Amino Alcohols via a Pd-Catalyzed Asymmetric Allylic Amination. Org. Biomol. Chem. 2013, 11, 7412–7419. 10.1039/c3ob41642a. 24077558

[ref127] Huo X.; He R.; Fu J.; Zhang J.; Yang G.; Zhang W. Stereoselective and Site-Specific Allylic Alkylation of Amino Acids and Small Peptides via a Pd/Cu Dual Catalysis. J. Am. Chem. Soc. 2017, 139, 9819–9822. 10.1021/jacs.7b05460. 28686426

[ref128] Huo X.; Fu J.; He X.; Chen J.; Xie F.; Zhang W. Pd/Cu Dual Catalysis: Highly Enantioselective Access to α-Substituted α-Amino Acids and α-Amino Amides. Chem. Commun. 2018, 54, 599–602. 10.1039/C7CC08732B. 29256570

[ref129] Liu P.; Huo X.; Li B.; He R.; Zhang J.; Wang T.; Xie F.; Zhang W. Stereoselective Allylic Alkylation of 1-Pyrroline-5-Carboxylic Esters via a Pd/Cu Dual Catalysis. Org. Lett. 2018, 20, 6564–6568. 10.1021/acs.orglett.8b02902. 30303386

[ref130] Huo X.; Zhang J.; Fu J.; He R.; Zhang W. Ir/Cu Dual Catalysis: Enantio- and Diastereodivergent Access to α,α-Disubstituted α-Amino Acids Bearing Vicinal Stereocenters. J. Am. Chem. Soc. 2018, 140, 2080–2084. 10.1021/jacs.8b00187. 29381351

[ref131] Wei L.; Xu S. M.; Zhu Q.; Che C.; Wang C. J. Synergistic Cu/Pd Catalysis for Enantioselective Allylic Alkylation of Aldimine Esters: Access to α,α-Disubstituted α-Amino Acids. Angew. Chem., Int. Ed. 2017, 56, 12312–12316. 10.1002/anie.201707019. 28741842

[ref132] Wei L.; Xiao L.; Wang C. J. Synergistic Cu/Pd Catalysis for Enantioselective Allylation of Ketimine Esters: The Direct Synthesis of α-Substituted α-Amino Acids and 2H-Pyrrols. Adv. Synth. Catal. 2018, 360, 4715–4719. 10.1002/adsc.201800986.

[ref133] Wei L.; Zhu Q.; Xu S. M.; Chang X.; Wang C. J. Stereodivergent Synthesis of α,α-Disubstituted α-Amino Acids via Synergistic Cu/Ir Catalysis. J. Am. Chem. Soc. 2018, 140, 1508–1513. 10.1021/jacs.7b12174. 29303578

[ref134] Nakao Y.; Ebata S.; Yada A.; Hiyama T.; Ikawa M.; Ogoshi S. Intramolecular Arylcyanation of Alkenes Catalyzed by Nickel/AlMe 2Cl. J. Am. Chem. Soc. 2008, 130, 12874–12875. 10.1021/ja805088r. 18778055

[ref135] Hsieh J. C.; Ebata S.; Nakao Y.; Hiyama T. Asymmetric Synthesis of Indolines Bearing a Benzylic Quaternary Stereocenter through Intramolecular Arylcyanation of Alkenes. Synlett 2010, 2010, 1709–1711. 10.1055/s-0029-1219964.

[ref136] Wang K.; Ding Z.; Zhou Z.; Kong W. Ni-Catalyzed Enantioselective Reductive Diarylation of Activated Alkenes by Domino Cyclization/Cross-Coupling. J. Am. Chem. Soc. 2018, 140, 12364–12368. 10.1021/jacs.8b08190. 30234979

[ref137] Lu W. J.; Hou X. L. Highly Enantioselective Construction of the α-Chiral Center of Amides via Iridium-Catalyzed Hydrogenation of α,β-Unsaturated Amides. Adv. Synth. Catal. 2009, 351, 1224–1228. 10.1002/adsc.200900080.

[ref138] Wang Y.; Liu Y.; Li K.; Yang G.; Zhang W. Iridium-Catalyzed Asymmetric Hydrogenation of Unsaturated Piperazin-2-Ones. Adv. Synth. Catal. 2017, 359, 1933–1941. 10.1002/adsc.201700175.

[ref139] Wang Y.; Yang G.; Xie F.; Zhang W. A Ferrocene-Based NH-Free Phosphine-Oxazoline Ligand for Iridium-Catalyzed Asymmetric Hydrogenation of Ketones. Org. Lett. 2018, 20, 6135–6139. 10.1021/acs.orglett.8b02591. 30226059

[ref140] Wu W.; Liu S.; Duan M.; Tan X.; Chen C.; Xie Y.; Lan Y.; Dong X. Q.; Zhang X. Iridium Catalysts with F-Amphox Ligands: Asymmetric Hydrogenation of Simple Ketones. Org. Lett. 2016, 18, 2938–2941. 10.1021/acs.orglett.6b01290. 27257935

[ref141] Wu W.; Xie Y.; Li P.; Li X.; Liu Y.; Dong X. Q.; Zhang X. Asymmetric Hydrogenation of α-Hydroxy Ketones with an Iridium/f-Amphox Catalyst: Efficient Access to Chiral 1,2-Diols. Org. Chem. Front. 2017, 4, 555–559. 10.1039/C6QO00810K.

[ref142] Hu Y.; Wu W.; Dong X. Q.; Zhang X. Efficient Access to Chiral 1,2-Amino Alcohols: Via Ir/f-Amphox-Catalyzed Asymmetric Hydrogenation of α-Amino Ketones. Org. Chem. Front. 2017, 4, 1499–1502. 10.1039/C7QO00237H.

[ref143] Wu W.; You C.; Yin C.; Liu Y.; Dong X. Q.; Zhang X. Enantioselective and Diastereoselective Construction of Chiral Amino Alcohols by Iridium-f-Amphox-Catalyzed Asymmetric Hydrogenation via Dynamic Kinetic Resolution. Org. Lett. 2017, 19, 2548–2551. 10.1021/acs.orglett.7b00844. 28448152

[ref144] Gu G.; Yang T.; Yu O.; Qian H.; Wang J.; Wen J.; Dang L.; Zhang X. Enantioselective Iridium-Catalyzed Hydrogenation of α-Keto Amides to α-Hydroxy Amides. Org. Lett. 2017, 19, 5920–5923. 10.1021/acs.orglett.7b02912. 29072464

[ref145] Hu Y.; Yin X.; Chen Z.; Dong X. Q.; Zhang X. Highly Enantioselective Ir/f-Amphox-Catalyzed Hydrogenation of Ketoamides: Efficient Access to Chiral Hydroxy Amides. Org. Chem. Front. 2018, 5, 2000–2003. 10.1039/C8QO00307F.

[ref146] Yin C.; Wu W.; Hu Y.; Tan X.; You C.; Liu Y.; Chen Z.; Dong X. Q.; Zhang X. Iridium-Catalyzed Asymmetric Hydrogenation of Halogenated Ketones for the Efficient Construction of Chiral Halohydrins. Adv. Synth. Catal. 2018, 360, 2119–2124. 10.1002/adsc.201800267.

[ref147] Zhang K.; Peng Q.; Hou X. L.; Wu Y. D. Highly Enantioselective Palladium-Catalyzed Alkylation of Acyclic Amides. Angew. Chem., Int. Ed. 2008, 47, 1741–1744. 10.1002/anie.200704629. 18214868

[ref148] Lei B. L.; Ding C. H.; Yang X. F.; Wan X. L.; Hou X. L. Kinetic Resolution of 2,3-Dihydro-2-Substituted 4-Quinolones by Palladium-Catalyzed Asymmetric Allylic Alkylation. J. Am. Chem. Soc. 2009, 131, 18250–18251. 10.1021/ja9082717. 19994886

[ref149] Yang X. F.; Ding C. H.; Li X. H.; Huang J. Q.; Hou X. L.; Dai L. X.; Wang P. J. Regio- and Enantioselective Palladium-Catalyzed Allylic Alkylation of Nitromethane with Monosubstituted Allyl Substrates: Synthesis of (R)-Rolipram and (R)-Baclofen. J. Org. Chem. 2012, 77, 8980–8985. 10.1021/jo301506p. 22992268

[ref150] Huang Y.; Zhang Q. S.; Fang P.; Chen T. G.; Zhu J.; Hou X. L. Pd-Catalyzed Highly Regio-, Diastereo-, and Enantioselective Allylic Alkylation of α-Fluorophosphonates. Chem. Commun. 2014, 50, 6751–6753. 10.1039/C4CC02158D. 24831029

[ref151] Cheruku P.; Gohil S.; Andersson P. G. Asymmetric Hydrogenation of Enol Phosphinates by Iridium Catalysts Having N,P Ligands. Org. Lett. 2007, 9, 1659–1661. 10.1021/ol070325l. 17388604

[ref152] Cheruku P.; Diesen J.; Andersson P. G. Asymmetric Hydrogenation of Di and Trisubstituted Enol Phosphinates with N,P-Ligated Iridium Complexes. J. Am. Chem. Soc. 2008, 130, 5595–5599. 10.1021/ja711372c. 18370383

[ref153] Paptchikhine A.; Cheruku P.; Engman M.; Andersson P. G. Iridium-Catalyzed Enantioselective Hydrogenation of Vinyl Boronates. Chem. Commun. 2009, (40), 5996–5998. 10.1039/b912590f. 19809622

[ref154] Verendel J. J.; Zhou T.; Li J. Q.; Paptchikhine A.; Lebedev O.; Andersson P. G. Highly Flexible Synthesis of Chiral Azacycles via Iridium-Catalyzed Hydrogenation. J. Am. Chem. Soc. 2010, 132, 8880–8881. 10.1021/ja103901e. 20557052

[ref155] Li J. Q.; Peters B.; Andersson P. G. Highly Enantioselective Asymmetric Isomerization of Primary Allylic Alcohols with an Iridium-N,P Complex. Chem. - Eur. J. 2011, 17, 11143–11145. 10.1002/chem.201101524. 21922551

[ref156] Li J. Q.; Quan X.; Andersson P. G. Highly Enantioselective Iridium-Catalyzed Hydrogenation of α,β-Unsaturated Esters. Chem. - Eur. J. 2012, 18, 10609–10616. 10.1002/chem.201200907. 22807347

[ref157] Ponra S.; Rabten W.; Yang J.; Wu H.; Kerdphon S.; Andersson P. G. Diastereo- and Enantioselective Synthesis of Fluorine Motifs with Two Contiguous Stereogenic Centers. J. Am. Chem. Soc. 2018, 140, 13878–13883. 10.1021/jacs.8b08778. 30265529

[ref158] Orgué S.; Flores-Gaspar A.; Biosca M.; Pàmies O.; Diéguez M.; Riera A.; Verdaguer X. Stereospecific SN2@P Reactions: Novel Access to Bulky P-Stereogenic Ligands. Chem. Commun. 2015, 51, 17548–17551. 10.1039/C5CC07504A. 26477668

[ref159] Salomó E.; Orgué S.; Riera A.; Verdaguer X. Highly Enantioselective Iridium-Catalyzed Hydrogenation of Cyclic Enamides. Angew. Chem., Int. Ed. 2016, 55, 7988–7992. 10.1002/anie.201602219. PMC5084810 27186653

[ref160] Salomó E.; Rojo P.; Hernández-Lladó P.; Riera A.; Verdaguer X. P-Stereogenic and Non-P-Stereogenic Ir-MaxPHOX in the Asymmetric Hydrogenation of N -Aryl Imines. Isolation and X-Ray Analysis of Imine Iridacycles. J. Org. Chem. 2018, 83, 4618–4627. 10.1021/acs.joc.8b00361. 29616810

[ref161] Cabré A.; Khaizourane H.; Garçon M.; Verdaguer X.; Riera A. Total Synthesis of (R)-Sarkomycin Methyl Ester via Regioselective Intermolecular Pauson-Khand Reaction and Iridium-Catalyzed Asymmetric Isomerization. Org. Lett. 2018, 20, 3953–3957. 10.1021/acs.orglett.8b01525. 29905075

[ref162] Bess E. N.; Sigman M. S. Distinctive Meta-Directing Group Effect for Iridium-Catalyzed 1,1-Diarylalkene Enantioselective Hydrogenation. Org. Lett. 2013, 15, 646–649. 10.1021/ol303465c. 23311924

[ref163] Müller M. A.; Pfaltz A. Asymmetric Hydrogenation of α,β-Unsaturated Nitriles with Base-Activated Iridium N,P Ligand Complexes. Angew. Chem., Int. Ed. 2014, 53, 8668–8671. 10.1002/anie.201402053. 24652627

[ref164] Lölsberg W.; Werle S.; Neudörfl J. M.; Schmalz H. G. An Enantioselective Total Synthesis of Helioporins C and E. Org. Lett. 2012, 14, 5996–5999. 10.1021/ol302898h. 23148527

[ref165] Maurer F.; Huch V.; Ullrich A.; Kazmaier U. Development of Catalysts for the Stereoselective Hydrogenation of α,β-Unsaturated Ketones. J. Org. Chem. 2012, 77, 5139–5143. 10.1021/jo300246c. 22571628

[ref166] Yang G.; Zhang W. Renaissance of Pyridine-Oxazolines as Chiral Ligands for Asymmetric Catalysis. Chem. Soc. Rev. 2018, 47, 1783–1810. 10.1039/C7CS00615B. 29469141

[ref167] Brunner H.; Obermann U.; Wimmer P. Asymmetrische Katalysen. XXXII. Enantioselektive Phenylierung von Cis-Cyclohexan-1,2-Diol Und Meso-Butan-2,3-Diol. J. Organomet. Chem. 1986, 316, 6–8.

[ref168] Arai Y.; Bunya Y.; Sengoku T.; Imamura Y. Synthesis of Chiral (Sulfinyl)Furyloxazoline Ligands and Its Application to Enantioselective Palladium-Catalyzed Allylic Alkylation. Heterocycles 2008, 76, 833–843. 10.3987/COM-08-S(N)18.

[ref169] Gao Y. X.; Chang L.; Shi H.; Liang B.; Wongkhan K.; Chaiyaveij D.; Batsanov A. S.; Marder T. B.; Li C. C.; Yang Z.; et al. A Thiourea-Oxazoline Library with Axial Chirality: Ligand Synthesis and Studies of the Palladium-Catalyzed Enantioselective Bis(Methoxycarbonylation) of Terminal Olefins. Adv. Synth. Catal. 2010, 352, 1955–1966. 10.1002/adsc.201000070.

[ref170] Ammann S. E.; Liu W.; White M. C. Enantioselective Allylic C-H Oxidation of Terminal Olefins to Isochromans by Palladium(II)/Chiral Sulfoxide Catalysis. Angew. Chem., Int. Ed. 2016, 55, 9571–9575. 10.1002/anie.201603576. PMC5729747 27376625

[ref171] Liu W.; Ali S. Z.; Ammann S. E.; White M. C. Asymmetric Allylic C-H Alkylation via Palladium(II)/ Cis-ArSOX Catalysis. J. Am. Chem. Soc. 2018, 140, 10658–10662. 10.1021/jacs.8b05668. 30091907 PMC6370307

[ref172] Chen S.-S.; Wu M.-S.; Han Z.-Y. Palladium-Catalyzed Cascade Sp2 C-H Functionalization/Intramolecular Asymmetric Allylation: From Aryl Ureas and 1,3-Dienes to Chiral Indolines. Angew. Chem., Int. Ed. 2017, 56, 6641–6645. 10.1002/anie.201702745. 28467624

[ref173] Bolm C.; Muñiz Fernández K.; Seger A.; Raabe G. A New Ferrocene in the Asymmetric Addition of Diethylzinc to Aldehydes. Synlett 1997, 1997, 1051–1052. 10.1055/s-1997-1528.

[ref174] Garabatos-Perera J. R.; Butenschön H. New Chiral Ferrocenyloxazolines: The First Planar Chiral Triferrocenylmethane Derivative and Its Use in Asymmetric Catalysis. J. Organomet. Chem. 2009, 694, 2047–2052. 10.1016/j.jorganchem.2009.01.057.

[ref175] Nottingham C.; Benson R.; Müller-Bunz H.; Guiry P. J. Synthesis of Ferrocene Oxazoline N,O Ligands and Their Application in Asymmetric Ethyl- and Phenylzinc Additions to Aldehydes. J. Org. Chem. 2015, 80, 10163–10176. 10.1021/acs.joc.5b01766. 26324068

[ref176] Zhou G.; Aboo A. H.; Robertson C. M.; Liu R.; Li Z.; Luzyanin K.; Berry N. G.; Chen W.; Xiao J. N,O- vs N,C-Chelation in Half-Sandwich Iridium Complexes: A Dramatic Effect on Enantioselectivity in Asymmetric Transfer Hydrogenation of Ketones. ACS Catal. 2018, 8, 8020–8026. 10.1021/acscatal.8b02068.

[ref177] Yang D.; Wang L.; Han F.; Li D.; Zhao D.; Wang R. Intermolecular Enantioselective Dearomatization Reaction of β-Naphthol Using Meso-Aziridine: A Bifunctional in Situ Generated Magnesium Catalyst. Angew. Chem., Int. Ed. 2015, 54, 2185–2189. 10.1002/anie.201410257. 25589219

[ref178] Wang L.; Yang D.; Li D.; Zhu H.; Wang P.; Liu X.; Bai L.; Wang R. Diversiform Reactivity of Naphthols in Asymmetric Dearomatization or O-Alkylation Reactions with Aziridines. Adv. Synth. Catal. 2018, 360, 4491–4496. 10.1002/adsc.201801041.

[ref179] Yang D.; Wang L.; Kai M.; Li D.; Yao X.; Wang R. Application of a C-C Bond-Forming Conjugate Addition Reaction in Asymmetric Dearomatization of β-Naphthols. Angew. Chem., Int. Ed. 2015, 54, 9523–9527. 10.1002/anie.201503056. 26173841

[ref180] Wang L.; Yang D.; Li D.; Wang P.; Wang K.; Wang J.; Jiang X.; Wang R. Mg(II)-Mediated Catalytic Asymmetric Dearomatization (CADA) Reaction of β-Naphthols with Dialkyl Acetylenedicarboxylates. Chem. - Eur. J. 2016, 22, 8483–8487. 10.1002/chem.201601399. 27139904

[ref181] Wang K.; Wang L.; Liu X.; Li D.; Zhu H.; Wang P.; Liu Y.; Yang D.; Wang R. Development of Biligands Magnesium Catalysis in Asymmetric Conjugate Reactions of C3-Pyrrolyl-Oxindoles. Org. Lett. 2017, 19, 4351–4354. 10.1021/acs.orglett.7b02044. 28753011

[ref182] Wang L.; Yang D.; Li D.; Liu X.; Zhao Q.; Zhu R.; Zhang B.; Wang R. Catalytic Asymmetric [3 + 2] Cyclization Reactions of 3-Isothiocyanato Oxindoles and Alkynyl Ketones Via an in Situ Generated Magnesium Catalyst. Org. Lett. 2015, 17, 4260–4263. 10.1021/acs.orglett.5b02052. 26291201

[ref183] Kurosu M.; Lin M. H.; Kishi Y. Fe/Cr- and Co/Cr-Mediated Catalytic Asymmetric 2-Haloallylations of Aldehydes. J. Am. Chem. Soc. 2004, 126, 12248–12249. 10.1021/ja045557j. 15453741

[ref184] Zhang Z.; Huang J.; Ma B.; Kishi Y. Further Improvement on Sulfonamide-Based Ligand for Catalytic Asymmetric 2-Haloallylation and Allylation. Org. Lett. 2008, 10, 3073–3076. 10.1021/ol801093p. 18549224

[ref185] Zhang Z.; Aubry S.; Kishi Y. Iterative Cr-Mediated Catalytic Asymmetric Allylation to Synthesize Syn/Syn- and Anti/Anti-1,3,5-Triols. Org. Lett. 2008, 10, 3077–3080. 10.1021/ol801094e. 18549227

[ref186] Liu S.; Kim J. T.; Dong C.-G.; Kishi Y. Catalytic Enantioselective Cr-Mediated Propargylation: Application to Halichondrin Synthesis. Org. Lett. 2009, 11, 4520–4523. 10.1021/ol9016595. 19810761 PMC2760007

[ref187] Guo H.; Dong C. G.; Kim D. S.; Urabe D.; Wang J.; Kim J. T.; Liu X.; Sasaki T.; Kishi Y. Toolbox Approach to the Search for Effective Ligands for Catalytic Asymmetric Cr-Mediated Coupling Reactions. J. Am. Chem. Soc. 2009, 131, 15387–15393. 10.1021/ja905843e. 19795862

[ref188] Kim D. S.; Dong C. G.; Kim J. T.; Guo H.; Huang J.; Tiseni P. S.; Kishi Y. New Syntheses of E7389 C14-C35 and Halichondrin C14-C38 Building Blocks: Double-Inversion Approach. J. Am. Chem. Soc. 2009, 131, 15636–15641. 10.1021/ja9058475. 19807076

[ref189] Dong C. G.; Henderson J. A.; Kaburagi Y.; Sasaki T.; Kim D. S.; Kim J. T.; Urabe D.; Guo H.; Kishi Y. New Syntheses of E7389 C14-C35 and Halichondrin C14-C38 Building Blocks: Reductive Cyclization and Oxy-Michael Cyclization Approaches. J. Am. Chem. Soc. 2009, 131, 15642–15646. 10.1021/ja9058487. 19807077

[ref190] Liu X.; Henderson J. A.; Sasaki T.; Kishi Y. Dramatic Improvement in Catalyst Loadings and Molar Ratios of Coupling Partners for Ni/Cr-Mediated Coupling Reactions: Heterobimetallic Catalysts. J. Am. Chem. Soc. 2009, 131, 16678–16680. 10.1021/ja9079308. 19874019 PMC2784119

[ref191] Zhang L.; Zuo Z.; Wan X.; Huang Z. Cobalt-Catalyzed Enantioselective Hydroboration of 1,1-Disubstituted Aryl Alkenes. J. Am. Chem. Soc. 2014, 136, 15501–15504. 10.1021/ja5093908. 25325782

[ref192] Chen J.; Xi T.; Ren X.; Cheng B.; Guo J.; Lu Z. Asymmetric Cobalt Catalysts for Hydroboration of 1,1-Disubstituted Alkenes. Org. Chem. Front. 2014, 1, 1306–1309. 10.1039/C4QO00295D.

[ref193] Chen J.; Xi T.; Lu Z. Iminopyridine Oxazoline Iron Catalyst for Asymmetric Hydroboration of 1,1-Disubstituted Aryl Alkenes. Org. Lett. 2014, 16, 6452–6455. 10.1021/ol503282r. 25438006

[ref194] Guo J.; Chen J.; Lu Z. Cobalt-Catalyzed Asymmetric Hydroboration of Aryl Ketones with Pinacolborane. Chem. Commun. 2015, 51, 5725–5727. 10.1039/C5CC01084E. 25719276

[ref195] Zhang H.; Lu Z. Dual-Stereocontrol Asymmetric Cobalt-Catalyzed Hydroboration of Sterically Hindered Styrenes. ACS Catal. 2016, 6, 6596–6600. 10.1021/acscatal.6b02278.

[ref196] Chen J.; Chen C.; Ji C.; Lu Z. Cobalt-Catalyzed Asymmetric Hydrogenation of 1,1-Diarylethenes. Org. Lett. 2016, 18, 1594–1597. 10.1021/acs.orglett.6b00453. 26974555

[ref197] Zhang H.; Lu Z. Nickel-Catalyzed Enantioselective Sequential Nazarov Cyclization/Decarboxylation. Org. Chem. Front. 2018, 5, 1763–1767. 10.1039/C8QO00279G.

[ref198] Zuo Z.; Zhang L.; Leng X.; Huang Z. Iron-Catalyzed Asymmetric Hydrosilylation of Ketones. Chem. Commun. 2015, 51, 5073–5076. 10.1039/C5CC00612K. 25712676

[ref199] Cheng B.; Lu P.; Zhang H.; Cheng X.; Lu Z. Highly Enantioselective Cobalt-Catalyzed Hydrosilylation of Alkenes. J. Am. Chem. Soc. 2017, 139, 9439–9442. 10.1021/jacs.7b04137. 28654260

[ref200] Cheng B.; Liu W.; Lu Z. Iron-Catalyzed Highly Enantioselective Hydrosilylation of Unactivated Terminal Alkenes. J. Am. Chem. Soc. 2018, 140, 5014–5017. 10.1021/jacs.8b01638. 29589919

[ref201] Ye W.; Zhao M.; Du W.; Jiang Q.; Wu K.; Wu P.; Yu Z. Highly Active Ruthenium(II) Complex Catalysts Bearing an Unsymmetrical NNN Ligand in the (Asymmetric) Transfer Hydrogenation of Ketones. Chem. - Eur. J. 2011, 17, 4737–4741. 10.1002/chem.201002039. 21404339

[ref202] Qi M. H.; Wang F. J.; Shi M. Synthesis of Novel Chiral Oxazoline-Schiff Base Ligands for the Catalytic Asymmetric Chlorination of β-Keto Esters. Tetrahedron: Asymmetry 2010, 21, 247–253. 10.1016/j.tetasy.2010.02.003.

[ref203] Zhu Y.; Burgess K. Iridium-Catalyzed Asymmetric Hydrogenation of Vinyl Ethers. Adv. Synth. Catal. 2008, 350, 979–983. 10.1002/adsc.200700546.

[ref204] Ito J. I.; Sugino K.; Matsushima S.; Sakaguchi H.; Iwata H.; Ishihara T.; Nishiyama H. Synthesis of NHC-Oxazoline Pincer Complexes of Rh and Ru and Their Catalytic Activity for Hydrogenation and Conjugate Reduction. Organometallics 2016, 35, 1885–1894. 10.1021/acs.organomet.6b00239.

[ref205] Wang F.; Li S.; Qu M.; Zhao M. X.; Liu L. J.; Shi M. A Highly Efficient Kinetic Resolution of Morita-Baylis-Hillman Adducts Achieved by N-Ar Axially Chiral Pd-Complexes Catalyzed Asymmetric Allylation. Chem. Commun. 2011, 47, 12813–12815. 10.1039/c1cc15543a. 22048620

[ref206] Kuang Y.; Sun X.; Chen H.; Liu P.; Jiang R. A Novel Planar Chiral N-Heterocyclic Carbene-Oxazoline Ligand for the Asymmetric Hydrosilylation of Ketones. Catal. Commun. 2009, 10, 1493–1496. 10.1016/j.catcom.2009.03.027.

[ref207] Hong B.; Ma Y.; Zhao L.; Duan W.; He F.; Song C. Synthesis of Planar Chiral Imidazo[1,5-a]Pyridinium Salts Based on [2.2]Paracyclophane for Asymmetric β-Borylation of Enones. Tetrahedron: Asymmetry 2011, 22, 1055–1062. 10.1016/j.tetasy.2011.06.023.

[ref208] Niu Z.; Chen J.; Chen Z.; Ma M.; Song C.; Ma Y. Application of Bidentate Oxazoline-Carbene Ligands with Planar and Central Chirality in Asymmetric β-Boration of α,β-Unsaturated Esters. J. Org. Chem. 2015, 80, 602–608. 10.1021/jo5021135. 25479457

[ref209] Wang X.; Chen Z.; Duan W.; Song C.; Ma Y. Synthesis of [2.2]Paracyclophane-Based Bidentate Oxazoline-Carbene Ligands for the Asymmetric 1,2-Silylation of N-Tosylaldimines. Tetrahedron: Asymmetry 2017, 28, 783–790. 10.1016/j.tetasy.2017.05.001.

[ref210] Abu-Elfotoh A. M.; Phomkeona K.; Shibatomi K.; Iwasa S. Asymmetric Inter- and Intramolecular Cyclopropanation Reactions Catalyzed by a Reusable Macroporous-Polymer-Supported Chiral Ruthenium(II)/Phenyloxazoline Complex. Angew. Chem., Int. Ed. 2010, 49, 8439–8443. 10.1002/anie.201002240. 20862663

[ref211] Chanthamath S.; Ozaki S.; Shibatomi K.; Iwasa S. Highly Stereoselective Synthesis of Cyclopropylphosphonates Catalyzed by Chiral Ru(II)-Pheox Complex. Org. Lett. 2014, 16, 3012–3015. 10.1021/ol501135p. 24856177

[ref212] Kotozaki M.; Chanthamath S.; Fujisawa I.; Shibatomi K.; Iwasa S. Highly Stereoselective Cyclopropanation of Various Olefins with Diazosulfones Catalyzed by Ru(II)-Pheox Complexes. Chem. Commun. 2017, 53, 12193–12196. 10.1039/C7CC05951E. 29072719

[ref213] Kotozaki M.; Chanthamath S.; Fujii T.; Shibatomi K.; Iwasa S. Highly Enantioselective Synthesis of Trifluoromethyl Cyclopropanes by Using Ru(II)-Pheox Catalysts. Chem. Commun. 2018, 54, 5110–5113. 10.1039/C8CC02286K. 29717307

[ref214] Abu-Elfotoh A. M.; Nguyen D. P. T.; Chanthamath S.; Phomkeona K.; Shibatomi K.; Iwasa S. Water-Soluble Chiral Ruthenium(II) Phenyloxazoline Complex: Reusable and Highly Enantioselective Catalyst for Intramolecular Cyclopropanation Reactions. Adv. Synth. Catal. 2012, 354, 3435–3439. 10.1002/adsc.201200508.

[ref215] Chanthamath S.; Mandour H. S. A.; Tong T. M. T.; Shibatomi K.; Iwasa S. Highly Stereoselective Cyclopropanation of Diazo Weinreb Amides Catalyzed by Chiral Ru(II)-: Amm -Pheox Complexes. Chem. Commun. 2016, 52, 7814–7817. 10.1039/C6CC02498J. 27240664

[ref216] Mandour H. S. A.; Chanthamath S.; Shibatomi K.; Iwasa S. Inter- and Intramolecular Cyclopropanations of Diazo Weinreb Amides Catalyzed by Ruthenium(II)-Amm-Pheox. Adv. Synth. Catal. 2017, 359, 1742–1746. 10.1002/adsc.201601345.

[ref217] Overman L. E.; Owen C. E.; Pavan M. M.; Richards C. J. Catalytic Asymmetric Rearrangement of Allylic N-Aryl Trifluoroacetimidates. A Useful Method for Transforming Prochiral Allylic Alcohols to Chiral Allylic Amines. Org. Lett. 2003, 5, 1809–1812. 10.1021/ol0271786. 12762658

[ref218] Cannon J. S.; Overman L. E. Palladium(II)-Catalyzed Enantioselective Reactions Using COP Catalysts. Acc. Chem. Res. 2016, 49, 2220–2231. 10.1021/acs.accounts.6b00398. 27689745

[ref219] Fischer D. F.; Barakat A.; Xin Z.; Weiss M. E.; Peters R. The Asymmetric Aza-Claisen Rearrangement: Development of Widely Applicable Pentaphenylferrocenyl Palladacycle Catalysts. Chem. - Eur. J. 2009, 15, 8722–8741. 10.1002/chem.200900712. 19691065

[ref220] Eitel S. H.; Bauer M.; Schweinfurth D.; Deibel N.; Sarkar B.; Kelm H.; Krüger H. J.; Frey W.; Peters R. Paramagnetic Palladacycles with Pd III Centers Are Highly Active Catalysts for Asymmetric Aza-Claisen Rearrangements. J. Am. Chem. Soc. 2012, 134, 4683–4693. 10.1021/ja2098222. 22320886

[ref221] Bauer J. M.; Frey W.; Peters R. Asymmetric Cascade Reaction to Allylic Sulfonamides from Allylic Alcohols by Palladium(II)/Base-Catalyzed Rearrangement of Allylic Carbamates. Angew. Chem., Int. Ed. 2014, 53, 7634–7638. 10.1002/anie.201403090. 24898109

[ref222] Bauer J. M.; Frey W.; Peters R. Dual Palladium(II)/Tertiary Amine Catalysis for Asymmetric Regioselective Rearrangements of Allylic Carbamates. Chem. - Eur. J. 2016, 22, 5767–5777. 10.1002/chem.201600138. 26990446

[ref223] Hahn B. T.; Tewes F.; Fröhlich R.; Glorius F. Olefin-Oxazolines (OlefOx): Highly Modular, Easily Tunable Ligands for Asymmetric Catalysis. Angew. Chem., Int. Ed. 2010, 49, 1143–1146. 10.1002/anie.200905712. 20039245

[ref224] Desimoni G.; Faita G.; Jørgensen K. A. Update 1 of: C 2 -Symmetric Chiral Bis(Oxazoline) Ligands in Asymmetric Catalysis. Chem. Rev. 2011, 111, 284–437. 10.1021/cr100339a. 22077602

[ref225] Srinivas H. D.; Maity P.; Yap G. P. A.; Watson M. P. Enantioselective Copper-Catalyzed Alkynylation of Benzopyranyl Oxocarbenium Ions. J. Org. Chem. 2015, 80, 4003–4016. 10.1021/acs.joc.5b00364. 25847687 PMC4822504

[ref226] Maity P.; Srinivas H. D.; Watson M. P. Copper-Catalyzed Enantioselective Additions to Oxocarbenium Ions: Alkynylation of Isochroman Acetals. J. Am. Chem. Soc. 2011, 133, 17142–17145. 10.1021/ja207585p. 21988178 PMC4203715

[ref227] Fu S.; Yang H.; Li G.; Deng Y.; Jiang H.; Zeng W. Copper(II)-Catalyzed Enantioselective Intramolecular Cyclization of N-Alkenylureas. Org. Lett. 2015, 17, 1018–1021. 10.1021/acs.orglett.5b00131. 25668749

[ref228] Hou M.; Deng R.; Gu Z. Cu-Catalyzed Enantioselective Atropisomer Synthesis via Thiolative Ring Opening of Five-Membered Cyclic Diaryliodoniums. Org. Lett. 2018, 20, 5779–5783. 10.1021/acs.orglett.8b02477. 30192153

[ref229] Truong P. M.; Zavalij P. Y.; Doyle M. P. Highly Enantioselective Carbonyl-Ene Reactions of 2,3-Diketoesters: Efficient and Atom-Economical Process to Functionalized Chiral α-Hydroxy-β-Ketoesters. Angew. Chem., Int. Ed. 2014, 53, 6468–6472. 10.1002/anie.201402233. 24846809

[ref230] Bovino M. T.; Chemler S. R. Catalytic Enantioselective Alkene Aminohalogenation/Cyclization Involving Atom Transfer. Angew. Chem., Int. Ed. 2012, 51, 3923–3927. 10.1002/anie.201109044. PMC3324620 22392873

[ref231] Harrar K.; Reiser O. Enantioselective Synthesis of (−)-Paeonilide. Chem. Commun. 2012, 48, 3457–3459. 10.1039/c2cc18172j. 22362262

[ref232] Chen S.; Rong C.; Feng P.; Li S.; Shi Y. Stereoselective Construction of the Tetracyclic Core of Cryptotrione. Org. Biomol. Chem. 2012, 10, 5518–5520. 10.1039/c2ob25923k. 22717843

[ref233] Buter J.; Moezelaar R.; Minnaard A. J. Enantioselective Palladium Catalyzed Conjugate Additions of Ortho-Substituted Arylboronic Acids to β,β-Disubstituted Cyclic Enones: Total Synthesis of Herbertenediol, Enokipodin A and Enokipodin B. Org. Biomol. Chem. 2014, 12, 5883–5890. 10.1039/C4OB01085J. 24984187

[ref234] Gottumukkala A. L.; Matcha K.; Lutz M.; De Vries J. G.; Minnaard A. J. Palladium-Catalyzed Asymmetric Quaternary Stereocenter Formation. Chem. - Eur. J. 2012, 18, 6907–6914. 10.1002/chem.201200694. 22532469

[ref235] Holmquist M.; Blay G.; Pedro J. R. Highly Enantioselective Aza-Henry Reaction with Isatin N-Boc Ketimines. Chem. Commun. 2014, 50, 9309–9312. 10.1039/C4CC04051A. 24999609

[ref236] Wu X.; Zhou W.; Wu H. H.; Zhang J. Enantioselective [3 + 2] Cycloaddition of Azomethine Ylides and Aldehydes: Via Ni/Bis(Oxazoline)-Catalyzed Ring Opening of N-Tosylaziridines through a Chirality Transfer Approach. Chem. Commun. 2017, 53, 5661–5664. 10.1039/C7CC02906C. 28485434

[ref237] Casavant B. J.; Hosseini A. S.; Chemler S. R. 6-Azabicyclo[3.2.1]Octanes Via Copper-Catalyzed Enantioselective Alkene Carboamination. Adv. Synth. Catal. 2014, 356, 2697–2702. 10.1002/adsc.201400317. 25484848 PMC4254729

[ref238] Fu J.; Shang H.; Wang Z.; Chang L.; Shao W.; Yang Z.; Tang Y. Gold-Catalyzed Rearrangement of Allylic Oxonium Ylides: Efficient Synthesis of Highly Functionalized Dihydrofuran-3-Ones. Angew. Chem., Int. Ed. 2013, 52, 4198–4202. 10.1002/anie.201208305. 23468251

[ref239] Gao S.; Chen J. R.; Hu X. Q.; Cheng H. G.; Lu L. Q.; Xiao W. J. Copper-Catalyzed Enantioselective Inverse Electron-Demand Hetero-Diels-Alder Reactions of Diazadienes with Enol Ethers: Efficient Synthesis of Chiral Pyridazines. Adv. Synth. Catal. 2013, 355, 3539–3544. 10.1002/adsc.201300723.

[ref240] Bigot A.; Williamson A. E.; Gaunt M. J. Enantioselective α-Arylation of N-Acyloxazolidinones with Copper(II)-Bisoxazoline Catalysts and Diaryliodonium Salts. J. Am. Chem. Soc. 2011, 133, 13778–13781. 10.1021/ja206047h. 21848264

[ref241] Kong K.; Moussa Z.; Lee C.; Romo D. Total Synthesis of the Spirocyclic Imine Marine Toxin (−)-Gymnodimine and an Unnatural C4-Epimer. J. Am. Chem. Soc. 2011, 133, 19844–19856. 10.1021/ja207385y. 22023219 PMC3256008

[ref242] Liwosz T. W.; Chemler S. R. Copper-Catalyzed Enantioselective Intramolecular Alkene Amination/Intermolecular Heck-Type Coupling Cascade. J. Am. Chem. Soc. 2012, 134, 2020–2023. 10.1021/ja211272v. 22257169 PMC3314298

[ref243] Cherney A. H.; Kadunce N. T.; Reisman S. E. Catalytic Asymmetric Reductive Acyl Cross-Coupling: Synthesis of Enantioenriched Acyclic α,α-Disubstituted Ketones. J. Am. Chem. Soc. 2013, 135, 7442–7445. 10.1021/ja402922w. 23634932

[ref244] Liang Y.; Fu G. C. Stereoconvergent Negishi Arylations of Racemic Secondary Alkyl Electrophiles: Differentiating between a CF3 and an Alkyl Group. J. Am. Chem. Soc. 2015, 137, 9523–9526. 10.1021/jacs.5b04725. 26203662 PMC4610818

[ref245] Bao H.; Tambar U. K. Catalytic Enantioselective Allylic Amination of Unactivated Terminal Olefins via an Ene Reaction/[2,3]-Rearrangement. J. Am. Chem. Soc. 2012, 134, 18495–18498. 10.1021/ja307851b. 23106555 PMC3511869

[ref246] Jaschinski T.; Hiersemann M. {1,6}-Transannular Catalytic Asymmetric Gosteli-Claisen Rearrangement. Org. Lett. 2012, 14, 4114–4117. 10.1021/ol3017676. 22860898

[ref247] Becker J.; Butt L.; Von Kiedrowski V.; Mischler E.; Quentin F.; Hiersemann M. Catalytic Asymmetric Claisen Rearrangement of Gosteli-Type Allyl Vinyl Ethers: Total Synthesis of (−)-9,10-Dihydroecklonialactone B. J. Org. Chem. 2014, 79, 3040–3051. 10.1021/jo5001466. 24621347

[ref248] Mao J.; Liu F.; Wang M.; Wu L.; Zheng B.; Liu S.; Zhong J.; Bian Q.; Walsh P. J. Cobalt-Bisoxazoline-Catalyzed Asymmetric Kumada Cross-Coupling of Racemic α-Bromo Esters with Aryl Grignard Reagents. J. Am. Chem. Soc. 2014, 136, 17662–17668. 10.1021/ja5109084. 25479180

[ref249] Flynn C. J.; Elcoate C. J.; Lawrence S. E.; Maguire A. R. Highly Enantioselective Intramolecular Copper Catalysed C-H Insertion Reactions of α -Diazosulfones Supporting Information Table of Contents:. J. Am. Chem. Soc. 2010, 132, 1184–1185. 10.1021/ja909713a. 20050655

[ref250] Slattery C. N.; Maguire A. R. Asymmetric Copper-Catalysed Intramolecular C-H Insertion Reactions of α-Diazo-β-Keto Sulfones. Org. Biomol. Chem. 2011, 9, 667–669. 10.1039/C0OB00914H. 21120229

[ref251] Shiely A. E.; Slattery C. N.; Ford A.; Eccles K. S.; Lawrence S. E.; Maguire A. R. Enantioselective Copper Catalysed Intramolecular C-H Insertion Reactions of α-Diazo-β-Keto Sulfones, α-Diazo-β-Keto Phosphine Oxides and 2-Diazo-1,3-Diketones; the Influence of the Carbene Substituent. Org. Biomol. Chem. 2017, 15, 2609–2628. 10.1039/C7OB00214A. 28267185

[ref252] Shiely A. E.; Clarke L.-A.; Flynn C. J.; Buckley A. M.; Ford A.; Lawrence S. E.; Maguire A. R. Substrate and Catalyst Effects in the Enantioselective Copper-Catalysed C-H Insertion Reactions of α-Diazo-β-Oxo Sulfones. Eur. J. Org. Chem. 2018, 2018, 2277–2289. 10.1002/ejoc.201800077.

[ref253] Hajra S.; Sinha D. Catalytic Enantioselective Aziridoarylation of Aryl Cinnamyl Ethers toward Synthesis of Trans-3-Amino-4-Arylchromans. J. Org. Chem. 2011, 76, 7334–7340. 10.1021/jo200711s. 21797274

[ref254] Choi J.; Martín-Gago P.; Fu G. C. Stereoconvergent Arylations and Alkenylations of Unactivated Alkyl Electrophiles: Catalytic Enantioselective Synthesis of Secondary Sulfonamides and Sulfones. J. Am. Chem. Soc. 2014, 136, 12161–12165. 10.1021/ja506885s. 25127186 PMC4151784

[ref255] Wu X. P.; Su Y.; Gu P. Catalytic Enantioselective Desymmetrization of 1,3-Diazido-2-Propanol via Intramolecular Interception of Alkyl Azides with Diazo(Aryl)Acetates. Org. Lett. 2014, 16, 5339–5341. 10.1021/ol502608d. 25290237

[ref256] Vita M. V.; Waser J. Azidation of β-Keto Esters and Silyl Enol Ethers with a Benziodoxole Reagent. Org. Lett. 2013, 15, 3246–3249. 10.1021/ol401229v. 23773166

[ref257] Trillo P.; Baeza A.; Nájera C. Copper-Catalyzed Asymmetric Alkylation of β-Keto Esters with Xanthydrols. Adv. Synth. Catal. 2013, 355, 2815–2821. 10.1002/adsc.201300627.

[ref258] Weng J. Q.; Fan R. J.; Deng Q. M.; Liu R. R.; Gao J. R.; Jia Y. X. Enantioselective Friedel-Crafts Alkylation Reactions of 3-Substituted Indoles with Electron-Deficient Alkenes. J. Org. Chem. 2016, 81, 3023–3030. 10.1021/acs.joc.6b00123. 26959867

[ref259] Sha W.; Zhu Y.; Mei H.; Han J.; Soloshonok V. A.; Pan Y. Catalytic Enantioselective Cyano-Trifluoromethylation of Styrenes. Chem. Sel. 2017, 2, 1129–1132. 10.1002/slct.201601893.

[ref260] Zhang H. X.; Nie J.; Cai H.; Ma J. A. Cyclic Aldimines as Superior Electrophiles for Cu-Catalyzed Decarboxylative Mannich Reaction of β-Ketoacids with a Broad Scope and High Enantioselectivity. Org. Lett. 2014, 16, 2542–2545. 10.1021/ol500929d. 24762142

[ref261] Barroso S.; Blay G.; Munoz M. C.; Pedro J. R. Highly Enantioselective Nitrone Cycloadditions with 2-Alkenoyl Pyridine N-Oxides Catalyzed by Cu(II)-BOX Complexes. Org. Lett. 2011, 13, 402 10.1021/ol102716e. 21175140

[ref262] Leighty M. W.; Shen B.; Johnston J. N. Enantioselective Synthesis of α-Oxy Amides via Umpolung Amide Synthesis. J. Am. Chem. Soc. 2012, 134, 15233–15236. 10.1021/ja306225u. 22967461 PMC3477818

[ref263] Shikora J. M.; Chemler S. R. Synthesis of Benzyl Amines via Copper-Catalyzed Enantioselective Aza-Friedel-Crafts Addition of Phenols to N -Sulfonyl Aldimines. Org. Lett. 2018, 20, 2133–2137. 10.1021/acs.orglett.8b00282. 29589946 PMC5937259

[ref264] Xie H.; Sammis G. M.; Flamme E. M.; Kraml C. M.; Sorensen E. J. The Catalytic Asymmetric Diels-Alder Reactions and Post-Cycloaddition Reductive Transpositions of 1-Hydrazinodienes. Chem. - Eur. J. 2011, 17, 11131–11134. 10.1002/chem.201102394. 21898626 PMC3222330

[ref265] Holmquist M.; Blay G.; Muñoz M. C.; Pedro J. R. Enantioselective Addition of Nitromethane to 2-Acylpyridine N -Oxides. Expanding the Generation of Quaternary Stereocenters with the Henry Reaction. Org. Lett. 2014, 16, 1204–1207. 10.1021/ol500082d. 24517643

[ref266] Zhu S.; MacMillan D. W. C. Enantioselective Copper-Catalyzed Construction of Aryl Pyrroloindolines via an Arylation-Cyclization Cascade. J. Am. Chem. Soc. 2012, 134, 10815–10818. 10.1021/ja305100g. 22716914 PMC3392034

[ref267] De Nanteuil F.; Serrano E.; Perrotta D.; Waser J. Dynamic Kinetic Asymmetric [3 + 2] Annulation Reactions of Aminocyclopropanes. J. Am. Chem. Soc. 2014, 136, 6239–6242. 10.1021/ja5024578. 24730733

[ref268] Harvey J. S.; Simonovich S. P.; Jamison C. R.; MacMillan D. W. C. Enantioselective α-Arylation of Carbonyls via Cu(I)-Bisoxazoline Catalysis. J. Am. Chem. Soc. 2011, 133, 13782–13785. 10.1021/ja206050b. 21848265 PMC3211083

[ref269] Wen L.; Shen Q.; Wan X.; Lu L. Enantioselective Friedel-Crafts Alkylation of Indoles with Trifluoroethylidene Malonates by Copper-Bis(Oxazoline) Complexes: Construction of Trifluoromethyl-Substituted Stereogenic Tertiary Carbon Center. J. Org. Chem. 2011, 76, 2282–2285. 10.1021/jo1024333. 21375277

[ref270] Sawa M.; Miyazaki S.; Yonesaki R.; Morimoto H.; Ohshima T. Catalytic Enantioselective Decarboxylative Mannich-Type Reaction of N-Unprotected Isatin-Derived Ketimines. Org. Lett. 2018, 20, 5393–5397. 10.1021/acs.orglett.8b02306. 30106593

[ref271] Huang R.; Chang X.; Li J.; Wang C. J. Cu(I)-Catalyzed Asymmetric Multicomponent Cascade Inverse Electron-Demand Aza-Diels-Alder/Nucleophilic Addition/Ring-Opening Reaction Involving 2-Methoxyfurans as Efficient Dienophiles. J. Am. Chem. Soc. 2016, 138, 3998–4001. 10.1021/jacs.6b01008. 26974596

[ref272] Suzuki S.; Tokunaga E.; Reddy D. S.; Matsumoto T.; Shiro M.; Shibata N. Enantioselective 5-Endo-Dig Carbocyclization of β-Ketoesters with Internal Alkynes Employing a Four-Component Catalyst System. Angew. Chem., Int. Ed. 2012, 51, 4131–4135. 10.1002/anie.201201060. 22422729

[ref273] Baidya M.; Griffin K. A.; Yamamoto H. Catalytic Enantioselective O -Nitrosocarbonyl Aldol Reaction of β-Dicarbonyl Compounds. J. Am. Chem. Soc. 2012, 134, 18566–18569. 10.1021/ja309311z. 23106266 PMC3515642

[ref274] Zhang Y. C.; Zhang B. W.; Geng R. L.; Song J. Enantioselective [3 + 2] Cycloaddition Reaction of Ethynylethylene Carbonates with Malononitrile Enabled by Organo/Metal Cooperative Catalysis. Org. Lett. 2018, 20, 7907–7911. 10.1021/acs.orglett.8b03454. 30540196

[ref275] Hajra S.; Bar S. Catalytic Enantioselective Synthesis of A-86929, a Dopamine D1 Agonist. Chem. Commun. 2011, 47, 3981–3982. 10.1039/c1cc10263j. 21321777

[ref276] Stache E. E.; Rovis T.; Doyle A. G. Dual Nickel- and Photoredox-Catalyzed Enantioselective Desymmetrization of Cyclic Meso-Anhydrides. Angew. Chem., Int. Ed. 2017, 56, 3679–3683. 10.1002/anie.201700097. PMC5510164 28230304

[ref277] Hajra S.; Akhtar S. M. S.; Aziz S. M. Catalytic Asymmetric Aminolactonization of 1,2-Disubstituted Alkenoic Acid Esters: Efficient Construction of Aminolactones with an All-Carbon Quaternary Stereo-Centre. Chem. Commun. 2014, 50, 6913–6916. 10.1039/C4CC01944J. 24840361

[ref278] Zhu R.; Buchwald S. L. Enantioselective Functionalization of Radical Intermediates in Redox Catalysis: Copper-Catalyzed Asymmetric Oxytrifluoromethylation of Alkenes. Angew. Chem., Int. Ed. 2013, 52, 12655–12658. 10.1002/anie.201307790. PMC3880552 24133010

[ref279] Miró J.; del Pozo C.; Toste F. D.; Fustero S. Enantioselective Palladium-Catalyzed Oxidative β,β-Fluoroarylation of α,β-Unsaturated Carbonyl Derivatives. Angew. Chem., Int. Ed. 2016, 55, 9045–9049. 10.1002/anie.201603046. PMC6482284 27272390

[ref280] Xie L.; Ma H.; Li J.; Yu Y.; Qin Z.; Fu B. Ni(II)-Catalyzed Enantioselective Mukaiyama-Mannich Reaction between Silyl Enol Ethers and Cyclic: N -Sulfonyl α-Ketiminoesters. Org. Chem. Front. 2017, 4, 1858–1862. 10.1039/C7QO00370F.

[ref281] Tsuda Y.; Kuriyama M.; Onomura O. Synthesis of Optically Active Oxazoline Derivatives via Catalytic Asymmetric Desymmetrization of 1,3-Diols. Chem. - Eur. J. 2012, 18, 2481–2483. 10.1002/chem.201103800. 22298351

[ref282] Xiong H. Y.; Yang Z. Y.; Chen Z.; Zeng J. L.; Nie J.; Ma J. A. Copper-Catalyzed One-Pot Denitrogenative-Dehydrogenative-Decarboxylative Coupling of β-Ketoacids with Trifluorodiazoethane: Facile Access to Trifluoromethylated Aldol Products. Chem. - Eur. J. 2014, 20, 8325–8329. 10.1002/chem.201403073. 24889186

[ref283] Wei L.; Wang Z. F.; Yao L.; Qiu G.; Tao H.; Li H.; Wang C. J. Copper(II)-Catalyzed Asymmetric 1,3-Dipolar [3 + 4] Cycloaddition and Kinetic Resolution of Azomethine Imines with Azoalkenes. Adv. Synth. Catal. 2016, 358, 3955–3959. 10.1002/adsc.201600961.

[ref284] Sen S.; Kamma S. R.; Gundla R.; Adepally U.; Kuncha S.; Thirnathi S.; Prasad U. V. A Reagent Based DOS Strategy via Evans Chiral Auxiliary: Highly Stereoselective Michael Reaction towards Optically Active Quinolizidinones, Piperidinones and Pyrrolidinones. RSC Adv. 2013, 3, 2404–2411. 10.1039/c2ra22115b.

[ref285] Kurono N.; Ohtsuga K.; Wakabayashi M.; Kondo T.; Ooka H.; Ohkuma T. Kinetic Resolution of Racemic α-Tert-Alkyl-α-Hydroxy Esters by Enantiomer-Selective Carbamoylation. J. Org. Chem. 2011, 76, 10312–10318. 10.1021/jo201940w. 22034833

[ref286] Desyatkin V. G.; Beletskaya I. P. Asymmetric Friedel-Crafts/Michael Reaction of Indoles and Pyrroles with Coumarin-3-Carbonylates. Synthesis 2017, 49, 4327–4334. 10.1055/s-0036-1589024.

[ref287] Mikami K.; Aikawa K.; Aida J. Fragment-Based Reaction Discovery of Non-Ene-Type Carbon-Carbon Bond-Forming Reactions: Catalytic Asymmetric Oxetane Synthesis by Screening Olefinic Reactants without Allylic Hydrogen. Synlett 2011, 18, 2719–2724. 10.1055/s-0031-1289540.

[ref288] Wu L.; Liu R. R.; Zhang G.; Wang D. J.; Wu H.; Gao J.; Jia Y. X. Enantioselective Construction of Cyclic Indolyl α-Amino Esters via a Friedel-Crafts Alkylation Reaction. Adv. Synth. Catal. 2015, 357, 709–713. 10.1002/adsc.201400987.

[ref289] Gao J.-R.; Wu H.; Xiang B.; Yu W.-B.; Han L.; Jia Y.-X. Highly Enantioselective Construction of Trifluoromethylated All-Carbon Quaternary Stereocenters via Nickel-Catalyzed Friedel-Crafts Alkylation Reaction. J. Am. Chem. Soc. 2013, 135, 2983–2986. 10.1021/ja400650m. 23409797

[ref290] Weng J. Q.; Deng Q. M.; Wu L.; Xu K.; Wu H.; Liu R. R.; Gao J. R.; Jia Y. X. Asymmetric Friedel-Crafts Alkylation of α-Substituted β-Nitroacrylates: Access to B2,2-Amino Acids Bearing Indolic All-Carbon Quaternary Stereocenters. Org. Lett. 2014, 16, 776–779. 10.1021/ol403480v. 24410114

[ref291] Best D.; Kujawa S.; Lam H. W. Diastereo- and Enantioselective Pd(II)-Catalyzed Additions of 2-Alkylazaarenes to N -Boc Imines and Nitroalkenes. J. Am. Chem. Soc. 2012, 134, 18193–18196. 10.1021/ja3083494. 23051617

[ref292] Simpson A. J.; Lam H. W. Enantioselective Nickel-Catalyzed Michael Additions of 2-Acetylazaarenes to Nitroalkenes. Org. Lett. 2013, 15, 2586–2589. 10.1021/ol400578c. 23659368

[ref293] Valli M.; Chiesa F.; Gandini A.; Porta A.; Vidari G.; Zanoni G. A Unified Stereodivergent Strategy for Prostaglandin and Isoprostanoid Synthesis. J. Org. Chem. 2014, 79, 2632–2639. 10.1021/jo500093k. 24552168

[ref294] Cheng Q. Q.; Yedoyan J.; Arman H.; Doyle M. P. Copper-Catalyzed Divergent Addition Reactions of Enoldiazoacetamides with Nitrones. J. Am. Chem. Soc. 2016, 138, 44–47. 10.1021/jacs.5b10860. 26699516

[ref295] Zhang Y. Q.; Yuan Y. A.; Liu G. S.; Xu H. Iron(II)-Catalyzed Asymmetric Intramolecular Aminohydroxylation of Indoles. Org. Lett. 2013, 15, 3910–3913. 10.1021/ol401666e. 23875702

[ref296] Liu C.; Yi J. C.; Liang X. W.; Xu R. Q.; Dai L. X.; You S. L. Copper(I)-Catalyzed Asymmetric Dearomatization of Indole Acetamides with 3-Indolylphenyliodonium Salts. Chem. - Eur. J. 2016, 22, 10813–10816. 10.1002/chem.201602229. 27171171

[ref297] Williamson K. S.; Yoon T. P. Iron Catalyzed Asymmetric Oxyamination of Olefins. J. Am. Chem. Soc. 2012, 134, 12370–12373. 10.1021/ja3046684. 22793789 PMC3422551

[ref298] You Y. S.; Kim T. W.; Kang S. H. Asymmetric Formation of Tert-Alkylamines from Serinols by a Dual Function Catalyst. Chem. Commun. 2013, 49, 9669–9671. 10.1039/c3cc45099f. 24022526

[ref299] Zhou Z.; Andrus M. B. Naphthyl-Substituted Bisoxazoline and Pyridylbisoxazoline-Copper(I) Catalysts for Asymmetric Allylic Oxidation. Tetrahedron Lett. 2012, 53, 4518–4521. 10.1016/j.tetlet.2012.06.010.

[ref300] Li M.; Hawkins A.; Barber D. M.; Bultinck P.; Herrebout W.; Dixon D. J. Enantio- and Diastereoselective Palladium Catalysed Arylative and Vinylative Allene Carbocyclisation Cascades. Chem. Commun. 2013, 49, 5265–5267. 10.1039/c3cc42079e. 23636233

[ref301] Hou G.; Yu J.; Yu C.; Wu G.; Miao Z. Enantio- and Diastereoselective Vinylogous Mukaiyama Aldol Reactions of α-Keto Phosphonates with 2-(Trimethylsilyloxy)-Furan Catalyzed by Bis(Oxazoline)-Copper Complexes. Adv. Synth. Catal. 2013, 355, 589–593. 10.1002/adsc.201200810.

[ref302] Minuth T.; Boysen M. M. K. Bis(Oxazolines) Based on Glycopyranosides - Steric, Configurational and Conformational Influences on Stereoselectivity. Beilstein J. Org. Chem. 2010, 6, 1–7. 10.3762/bjoc.6.23. 20502606 PMC2874406

[ref303] Özüduru G.; Schubach T.; Boysen M. M. K. Enantioselective Cyclopropanation of Indoles: Construction of All-Carbon Quaternary Stereocenters. Org. Lett. 2012, 14, 4990–4993. 10.1021/ol302388t. 23009104

[ref304] Minuth T.; Boysen M. M. K. Carbohydrate-Derived Bis(Oxazoline) Ligand in the Total Synthesis of Grenadamide. Synthesis 2010, 2010 (16), 2799–2803. 10.1055/s-0030-1258143.

[ref305] George J.; Reddy B. V. S. Enantioselective Friedel-Crafts Alkylation of Indoles with 2-Enoylpyridine-N-Oxides Catalyzed by GlucoBOX-Cu(II) Complex. Org. Biomol. Chem. 2012, 10, 4731–4738. 10.1039/c2ob25315a. 22585295

[ref306] Livieri A.; Boiocchi M.; Desimoni G.; Faita G. Enantioselective Cycloadditions of 2-Alkenoylpyridine-N-Oxides Catalysed by a Bis(Oxazoline)/Cu(II) Complex: Structure of the Reactive Intermediate. Chem. - Eur. J. 2011, 17, 516–520. 10.1002/chem.201002017. 21207568

[ref307] Livieri A.; Boiocchi M.; Desimoni G.; Faita G. Enantioselective Addition of Cyclic Enol Silyl Ethers to 2-Alkenoyl-Pyridine-N-Oxides Catalysed by Cu(II)-Bis(Oxazoline) Complexes. Chem. - Eur. J. 2012, 18, 11662–11668. 10.1002/chem.201201214. 22847937

[ref308] Zhang G.; Zhang Y.; Wang R. Catalytic Asymmetric Activation of a Csp3-H Bond Adjacent to a Nitrogen Atom: A Versatile Approach to Optically Active α-Alkyl α-Amino Acids and C1-Alkylated Tetrahydroisoquinoline Derivatives. Angew. Chem., Int. Ed. 2011, 50, 10429–10432. 10.1002/anie.201105123. 21915984

[ref309] Zhang H.; Kang H.; Hong L.; Dong W.; Li G.; Zheng X.; Wang R. Construction of the N1-C3 Linkage Stereogenic Centers by Catalytic Asymmetric Amination Reaction of 3-Bromooxindoles with Indolines. Org. Lett. 2014, 16, 2394–2397. 10.1021/ol5007423. 24725065

[ref310] Williamson K. S.; Sawicki J. W.; Yoon T. P. Iron-Catalyzed Kinetic Resolution of N-Sulfonyl Oxaziridines. Chem. Sci. 2014, 5, 3524–3527. 10.1039/C4SC01371A.

[ref311] Adachi S.; Takeda N.; Sibi M. P. Evaluation of Achiral Templates with Fluxional Brønsted Basic Substituents in Enantioselective Conjugate Additions. Org. Lett. 2014, 16, 6440–6443. 10.1021/ol503275w. 25490703

[ref312] Ray S. K.; Singh P. K.; Singh V. K. Enantioselective Michael Addition of Malonates to 2-Enoylpyridine N-Oxides Catalyzed by Chiral Bisoxazoline-Zn(II) Complex. Org. Lett. 2011, 13, 5812–5815. 10.1021/ol202405v. 21970689

[ref313] Cheng H. G.; Lu L. Q.; Wang T.; Yang Q. Q.; Liu X. P.; Li Y.; Deng Q. H.; Chen J. R.; Xiao W. J. Highly Enantioselective Friedel-Crafts Alkylation/N-Hemiacetalization Cascade Reaction with Indoles. Angew. Chem., Int. Ed. 2013, 52, 3250–3254. 10.1002/anie.201209998. 23401220

[ref314] Feng L. W.; Ren H.; Xiong H.; Wang P.; Wang L.; Tang Y. Reaction of Donor-Acceptor Cyclobutanes with Indoles: A General Protocol for the Formal Total Synthesis of (±)-Strychnine and the Total Synthesis of (±)-Akuammicine. Angew. Chem., Int. Ed. 2017, 56, 3055–3058. 10.1002/anie.201611734. 28170147

[ref315] Liu Q. J.; Wang L.; Kang Q. K.; Zhang X. P.; Tang Y. Cy-SaBOX/Copper(II)-Catalyzed Highly Diastereo- and Enantioselective Synthesis of Bicyclic N,O Acetals. Angew. Chem., Int. Ed. 2016, 55, 9220–9223. 10.1002/anie.201603911. 27351738

[ref316] Deng C.; Wang L. J.; Zhu J.; Tang Y. A Chiral Cagelike Copper(I) Catalyst for the Highly Enantioselective Synthesis of 1,1-Cyclopropane Diesters. Angew. Chem., Int. Ed. 2012, 51, 11620–11623. 10.1002/anie.201206376. 23065765

[ref317] Li J.; Zheng L.; Chen H.; Wang L.; Sun X. L.; Zhu J.; Tang Y. Highly Enantioselective Cyclopropanation of Trisubstituted Olefins. Sci. China: Chem. 2018, 61, 526–530. 10.1007/s11426-017-9200-9.

[ref318] Hu J. L.; Feng L. W.; Wang L.; Xie Z.; Tang Y.; Li X. Enantioselective Construction of Cyclobutanes: A New and Concise Approach to the Total Synthesis of (+)-Piperarborenine B. J. Am. Chem. Soc. 2016, 138, 13151–13154. 10.1021/jacs.6b08279. 27604907

[ref319] Xu H.; Qu J. P.; Liao S.; Xiong H.; Tang Y. Highly Enantioselective [3 + 2] Annulation of Cyclic Enol Silyl Ethers with Donor-Acceptor Cyclopropanes: Accessing 3a-Hydroxy [n.3.0]Carbobicycles. Angew. Chem., Int. Ed. 2013, 52, 4004–4007. 10.1002/anie.201300032. 23460304

[ref320] Deng T.; Cai C. Fluorous Chiral Bis(Oxazolines): Synthesis and Application in Asymmetric Henry Reaction. J. Fluorine Chem. 2013, 156, 183–186. 10.1016/j.jfluchem.2013.09.014.

[ref321] Deng T.; Wang H.; Cai C. Copper-Catalyzed Asymmetric Addition to Isatins to Give 3-Hydroxy-2-Oxindoles by C-H Activation. Eur. J. Org. Chem. 2014, 2014, 7259–7264. 10.1002/ejoc.201402852.

[ref322] Deng T.; Cai C. Bis(Oxazoline)-Copper Catalyzed Enantioselective Hydrophosphonylation of Aldehydes. RSC Adv. 2014, 4, 27853–27856. 10.1039/C4RA03269A.

[ref323] Li N. K.; Kong L. P.; Qi Z. H.; Yin S. J.; Zhang J. Q.; Wu B.; Wang X. W. Friedel-Crafts Reaction of Indoles with Isatin-Derived β,γ-Unsaturated α-Keto Esters Using a BINOL-Derived Bisoxazoline (BOX)/Copper(II) Complex as Catalyst. Adv. Synth. Catal. 2016, 358, 3100–3112. 10.1002/adsc.201600272.

[ref324] Chu W. D.; Zhang L.; Zhang Z.; Zhou Q.; Mo F.; Zhang Y.; Wang J. Enantioselective Synthesis of Trisubstituted Allenes via Cu(I)-Catalyzed Coupling of Diazoalkanes with Terminal Alkynes. J. Am. Chem. Soc. 2016, 138, 14558–14561. 10.1021/jacs.6b09674. 27788320

[ref325] Quan M.; Wang X.; Wu L.; Gridnev I. D.; Yang G.; Zhang W. Ni(II)-Catalyzed Asymmetric Alkenylations of Ketimines. Nat. Commun. 2018, 9, 1–11. 29884893 10.1038/s41467-018-04645-3 PMC5993804

[ref326] Wu L.; Shao Q.; Yang G.; Zhang W. Cobalt-Catalyzed Asymmetric Allylation of Cyclic Ketimines. Chem. - Eur. J. 2018, 24, 1241–1245. 10.1002/chem.201704760. 29120070

[ref327] Liang Y.; Fu G. C. Catalytic Asymmetric Synthesis of Tertiary Alkyl Fluorides: Negishi Cross-Couplings of Racemic α,α-Dihaloketones. J. Am. Chem. Soc. 2014, 136, 5520–5524. 10.1021/ja501815p. 24678878 PMC4004247

[ref328] Choi J.; Fu G. C. Catalytic Asymmetric Synthesis of Secondary Nitriles via Stereoconvergent Negishi Arylations and Alkenylations of Racemic α-Bromonitriles. J. Am. Chem. Soc. 2012, 134, 9102–9105. 10.1021/ja303442q. 22612264 PMC3415582

[ref329] Lou S.; Fu G. C. Nickel/Bis(Oxazoline)-Catalyzed Asymmetric Kumada Reactions of Alkyl Electrophiles: Cross-Couplings of Racemic α-Bromoketones. J. Am. Chem. Soc. 2010, 132, 1264–1266. 10.1021/ja909689t. 20050651 PMC2814537

[ref330] Qiao J.-B.; Zhao Y.-M.; Gu P. Asymmetric Intramolecular Desymmetrization of Meso -α,α ′-Diazido Alcohols with Aryldiazoacetates: Assembly of Chiral C_3_ Fragments with Three Continuous Stereocenters. Org. Lett. 2016, 18, 1984–1987. 10.1021/acs.orglett.6b00570. 27109428

[ref331] Zhang W.; Wang F.; McCann S. D.; Wang D.; Chen P.; Stahl S. S.; Liu G. Enantioselective Cyanation of Benzylic C-H Bonds via Copper-Catalyzed Radical Relay. Science 2016, 353, 1014–1018. 10.1126/science.aaf7783. 27701109 PMC5488708

[ref332] Shibata M.; Ikeda M.; Motoyama K.; Miyake Y.; Nishibayashi Y. Enantioselective Alkylation of β-Keto Phosphonates by Direct Use of Diaryl Methanols as Electrophiles. Chem. Commun. 2012, 48, 9528–9530. 10.1039/c2cc35262a. 22895465

[ref333] Gerten A. L.; Slade M. C.; Pugh K. M.; Stanley L. M. Catalytic, Enantioselective 1,3-Dipolar Cycloadditions of Nitrile Imines with Methyleneindolinones. Org. Biomol. Chem. 2013, 11, 7834–7837. 10.1039/c3ob41815d. 24132663 PMC3876797

[ref334] Guo C.; Song J.; Luo S. W.; Gong L. Z. Enantioselective Oxidative Cross-Coupling Reaction of 3indolylmethyl c-h Bonds with 1, 3-Dicarbonyls Using a Chiral Lewis Acid-Bonded Nucleophile to Control Stereochemistry. Angew. Chem., Int. Ed. 2010, 49, 5558–5562. 10.1002/anie.201002108. 20589821

[ref335] Fu L.; Zhou S.; Wan X.; Chen P.; Liu G. Enantioselective Trifluoromethylalkynylation of Alkenes via Copper-Catalyzed Radical Relay. J. Am. Chem. Soc. 2018, 140, 10965–10969. 10.1021/jacs.8b07436. 30133271

[ref336] Sha W.; Deng L.; Ni S.; Mei H.; Han J.; Pan Y. Merging Photoredox and Copper Catalysis: Enantioselective Radical Cyanoalkylation of Styrenes. ACS Catal. 2018, 8, 7489–7494. 10.1021/acscatal.8b01863.

[ref337] Cherney A. H.; Reisman S. E. Nickel-Catalyzed Asymmetric Reductive Cross-Coupling between Vinyl and Benzyl Electrophiles. J. Am. Chem. Soc. 2014, 136, 14365–14368. 10.1021/ja508067c. 25245492 PMC4210114

[ref338] Li J.; Chen H. L.; Liu L.; Fu B. Synthesis of New C2- Symmetric Fluoren-9-Ylidene Malonate Derived Bis(Oxazoline) Ligands and Their Application in Friedel-Crafts Reactions. Molecules 2010, 15, 8582–8592. 10.3390/molecules15128582. 21116227 PMC6259425

[ref339] Ma H. L.; Xie L.; Zhang Z. H.; Li J. Q.; Qin Z. H.; Fu B. Enantioselective Copper(II)-Catalyzed Conjugate Addition of Indoles to β-Substituted Unsaturated Acyl Phosphonates. Adv. Synth. Catal. 2016, 358, 1011–1016. 10.1002/adsc.201500923.

[ref340] Li B.; Chao Z.; Li C.; Gu Z. Cu-Catalyzed Enantioselective Ring Opening of Cyclic Diaryliodoniums toward the Synthesis of Chiral Diarylmethanes. J. Am. Chem. Soc. 2018, 140, 9400–9403. 10.1021/jacs.8b05743. 30015479

[ref341] aFritschi H.; Leutenegger U.; Siegmann K.; Pfaltz A.; Keller W.; Kratky C. Synthesis of Chiral Semicorrin Ligands and General Concept. Helv. Chim. Acta 1988, 71, 1541–1552. 10.1002/hlca.19880710620.

[ref342] Ding W.; Lu L. Q.; Zhou Q. Q.; Wei Y.; Chen J. R.; Xiao W. J. Bifunctional Photocatalysts for Enantioselective Aerobic Oxidation of β-Ketoesters. J. Am. Chem. Soc. 2017, 139, 63–66. 10.1021/jacs.6b11418. 28001382

[ref343] Liu C.; Yi J. C.; Zheng Z. B.; Tang Y.; Dai L. X.; You S. L. Enantioselective Synthesis of 3a-Amino-Pyrroloindolines by Copper-Catalyzed Direct Asymmetric Dearomative Amination of Tryptamines. Angew. Chem., Int. Ed. 2016, 55, 751–754. 10.1002/anie.201508570. 26603145

[ref344] Zhu M.; Wang D. C.; Xie M. S.; Qu G. R.; Guo H. M. Enantioselective Friedel-Crafts Alkylation Reactions of β-Naphthols with Donor-Acceptor Aminocyclopropanes. Chem. - Eur. J. 2018, 24, 15512–15516. 10.1002/chem.201804032. 30125402

[ref345] Fang Z. J.; Zheng S. C.; Guo Z.; Guo J. Y.; Tan B.; Liu X. Y. Asymmetric Synthesis of Axially Chiral Isoquinolones: Nickel-Catalyzed Denitrogenative Transannulation. Angew. Chem., Int. Ed. 2015, 54, 9528–9532. 10.1002/anie.201503207. 26119725

[ref346] Cichowicz N. R.; Kaplan W.; Khomutnyk Y.; Bhattarai B.; Sun Z.; Nagorny P. Concise Enantioselective Synthesis of Oxygenated Steroids via Sequential Copper(II)-Catalyzed Michael Addition/Intramolecular Aldol Cyclization Reactions. J. Am. Chem. Soc. 2015, 137, 14341–14348. 10.1021/jacs.5b08528. 26491886 PMC4651737

[ref347] Hut’Ka M.; Tsubogo T.; Kobayashi S. Synthesis of Glutamic Acid and Highly Functionalized Pyrrolidine Derivatives by Utilizing Tunable Calcium Catalysts for Chemoselective Asymmetric 1,4-Addition and [3 + 2] Cycloaddition Reactions. Adv. Synth. Catal. 2013, 355, 1561–1569. 10.1002/adsc.201300171.

[ref348] Angulo B.; García J. I.; Herrerías C. I.; Mayoral J. A.; Miñana A. C. Polytopic Bis(Oxazoline)-Based Ligands for Recoverable Catalytic Systems Applied to the Enantioselective Henry Reaction. Org. Biomol. Chem. 2015, 13, 9314–9322. 10.1039/C5OB01033K. 26240011

[ref349] Li Y.; Zhou K.; Wen Z.; Cao S.; Shen X.; Lei M.; Gong L. Copper(II)-Catalyzed Asymmetric Photoredox Reactions: Enantioselective Alkylation of Imines Driven by Visible Light. J. Am. Chem. Soc. 2018, 140, 15850–15858. 10.1021/jacs.8b09251. 30372057

[ref350] aUetake Y.; Uwamori M.; Nakada M. Enantioselective Approach to Polycyclic Polyprenylated Acylphloroglucinols via Catalytic Asymmetric Intramolecular Cyclopropanation. J. Org. Chem. 2015, 80, 1735–1745. 10.1021/jo5026699. 25581002

[ref351] Tellis J. C.; Primer D. N.; Molander G. A. Single-Electron Transmetalation in Organoboron Cross-Coupling by Photoredox/Nickel Dual Catalysis. Science 2014, 345, 433–436. 10.1126/science.1253647. 24903560 PMC4406487

[ref352] Woods B. P.; Orlandi M.; Huang C. Y.; Sigman M. S.; Doyle A. G. Nickel-Catalyzed Enantioselective Reductive Cross-Coupling of Styrenyl Aziridines. J. Am. Chem. Soc. 2017, 139, 5688–5691. 10.1021/jacs.7b03448. 28406622

[ref353] Poremba K. E.; Kadunce N. T.; Suzuki N.; Cherney A. H.; Reisman S. E. Nickel-Catalyzed Asymmetric Reductive Cross-Coupling To Access 1,1-Diarylalkanes. J. Am. Chem. Soc. 2017, 139, 5684–5687. 10.1021/jacs.7b01705. 28406620 PMC5851002

[ref354] Banerjee A.; Yamamoto H. Nickel Catalyzed Regio-, Diastereo-, and Enantioselective Cross-Coupling of 3,4-Epoxyalcohol with Aryl Iodides. Org. Lett. 2017, 19, 4363–4366. 10.1021/acs.orglett.7b02076. 28753019

[ref355] Liu J.; Ding W.; Zhou Q. Q.; Liu D.; Lu L. Q.; Xiao W. J. Enantioselective Di-/Perfluoroalkylation of β-Ketoesters Enabled by Cooperative Photoredox/Nickel Catalysis. Org. Lett. 2018, 20, 461–464. 10.1021/acs.orglett.7b03826. 29313355

[ref356] Chen J.; Lu X.; Lou W.; Ye Y.; Jiang H.; Zeng W. Palladium(II)-Catalyzed Enantioselective Arylation of α-Imino Esters. J. Org. Chem. 2012, 77, 8541–8548. 10.1021/jo301423e. 22989204

[ref357] Wei X. H.; Wang G. W.; Yang S. D. Enantioselective Synthesis of Arylglycine Derivatives by Direct C-H Oxidative Cross-Coupling. Chem. Commun. 2015, 51, 832–835. 10.1039/C4CC07361D. 25348347

[ref358] Beisel T.; Manolikakes G. Palladium-Catalyzed Enantioselective Three-Component Synthesis of α-Substituted Amines. Org. Lett. 2015, 17, 3162–3165. 10.1021/acs.orglett.5b01502. 26053313

[ref359] Beisel T.; Diehl A. M.; Manolikakes G. Palladium-Catalyzed Enantioselective Three-Component Synthesis of α-Arylglycines. Org. Lett. 2016, 18, 4116–4119. 10.1021/acs.orglett.6b02045. 27505131

[ref360] He Y.; Yang Z.; Thornbury R. T.; Toste F. D. Palladium-Catalyzed Enantioselective 1,1-Fluoroarylation of Aminoalkenes. J. Am. Chem. Soc. 2015, 137, 12207–12210. 10.1021/jacs.5b07795. 26378886 PMC4601482

[ref361] Oliveira C. C.; Angnes R. A.; Correia C. R. D. Intermolecular Enantioselective Heck-Matsuda Arylations of Acyclic Olefins: Application to the Synthesis of β-Aryl-γ-Lactones and β-Aryl Aldehydes. J. Org. Chem. 2013, 78, 4373–4385. 10.1021/jo400378g. 23570395

[ref362] Do H.-Q.; Chandrashekar E. R. R.; Fu G. C. Nickel/Bis(Oxazoline)-Catalyzed Asymmetric Negishi Arylations of Racemic Secondary Benzylic Electrophiles to Generate Enantioenriched 1,1-Diarylalkanes. J. Am. Chem. Soc. 2013, 135, 16288–16291. 10.1021/ja408561b. 24164502 PMC3869004

[ref363] Balaraman K.; Vasanthan R.; Kesavan V. Asymmetric Henry Reaction Catalyzed by Novel Chiral Bioxazolines from Tartaric Acid. Synthesis 2012, 44, 2455–2462. 10.1055/s-0031-1289811.

[ref364] Balaraman K.; Vasanthan R.; Kesavan V. Application of Tartarate Derived Bidentate Bioxazolines in Enantioselective Addition of Terminal Alkynes to Imines. Tetrahedron Lett. 2013, 54, 3613–3616. 10.1016/j.tetlet.2013.04.108.

[ref365] Jayakumar S.; Prakash M.; Balaraman K.; Kesavan V. Highly Enantioselective Alkylation of Allyl Acetates Using Tartrate-Derived Bioxazoline Ligands. Eur. J. Org. Chem. 2014, 2014, 606–615. 10.1002/ejoc.201301208.

[ref366] Jayakumar S.; Kumarswamyreddy N.; Prakash M.; Kesavan V. Palladium Catalyzed Asymmetric Allylation of 3-OBoc-Oxindoles: An Efficient Synthesis of 3-Allyl-3-Hydroxyoxindoles. Org. Lett. 2015, 17, 1066–1069. 10.1021/acs.orglett.5b00034. 25675215

[ref367] Oliveira C. C.; Pfaltz A.; Correia C. R. D. Quaternary Stereogenic Centers through Enantioselective Heck Arylation of Acyclic Olefins with Aryldiazonium Salts: Application in a Concise Synthesis of (*R*)-Verapamil. Angew. Chem., Int. Ed. 2015, 54, 14036–14039. 10.1002/anie.201507927. 26404102

[ref368] Khan I. U.; Kattela S.; Hassan A.; Correia C. R. D. Enantioselective Total Synthesis of the Highly Selective Sphingosine-1-Receptor VPC01091 by the Heck Desymmetrization of a Non-Activated Cyclopentene-Fused Spiro-Pyrrolidinone. Org. Biomol. Chem. 2016, 14, 9476–9480. 10.1039/C6OB01892K. 27722726

[ref369] de Azambuja F.; Carmona R. C.; Chorro T. H. D.; Heerdt G.; Correia C. R. D. Noncovalent Substrate-Directed Enantioselective Heck Reactions: Synthesis of S- and P-Stereogenic Heterocycles. Chem. - Eur. J. 2016, 22, 11205–11209. 10.1002/chem.201602572. 27273079

[ref370] Zhu S. F.; Cai Y.; Mao H. X.; Xie J. H.; Zhou Q. L. Enantioselective Iron-Catalysed O-H Bond Insertions. Nat. Chem. 2010, 2, 546–551. 10.1038/nchem.651. 20571572

[ref371] Zhu S. F.; Song X. G.; Li Y.; Cai Y.; Zhou Q. L. Enantioselective Copper-Catalyzed Intramolecular O-H Insertion: An Efficient Approach to Chiral 2-Carboxy Cyclic Ethers. J. Am. Chem. Soc. 2010, 132, 16374–16376. 10.1021/ja1078464. 21033664

[ref372] Zhu S. F.; Chen W. Q.; Zhang Q. Q.; Mao H. X.; Zhou Q. L. Enantioselective Copper-Catalyzed O-H Insertion of α-Diazo Phosphonates. Synlett 2011, 2011, 919–922. 10.1055/s-0030-1259710.

[ref373] Xie X. L.; Zhu S. F.; Guo J. X.; Cai Y.; Zhou Q. L. Enantioselective Palladium-Catalyzed Insertion of α-Aryl-α- Diazoacetates into the O-H Bonds of Phenols. Angew. Chem., Int. Ed. 2014, 53, 2978–2981. 10.1002/anie.201309820. 24500845

[ref374] Cheng Q.-Q.; Zhu S.-F.; Zhang Y.-Z.; Xie X.-L.; Zhou Q.-L. Copper-Catalyzed B-H Bond Insertion Reaction: A Highly Efficient and Enantioselective C-B Bond-Forming Reaction with Amine-Borane and Phosphine-Borane Adducts. J. Am. Chem. Soc. 2013, 135, 14094–14097. 10.1021/ja408306a. 24025045

[ref375] Zhu S. F.; Xu B.; Wang G. P.; Zhou Q. L. Well-Defined Binuclear Chiral Spiro Copper Catalysts for Enantioselective N-H Insertion. J. Am. Chem. Soc. 2012, 134, 436–442. 10.1021/ja2084493. 22066865

[ref376] Song X. G.; Ren Y. Y.; Zhu S. F.; Zhou Q. L. Enantioselective Copper-Catalyzed Intramolecular N-H Bond Insertion: Synthesis of Chiral 2-Carboxytetrahydroquinolines. Adv. Synth. Catal. 2016, 358, 2366–2370. 10.1002/adsc.201600390.

[ref377] Shen J. J.; Zhu S. F.; Cai Y.; Xu H.; Xie X. L.; Zhou Q. L. Enantioselective Iron-Catalyzed Intramolecular Cyclopropanation Reactions. Angew. Chem., Int. Ed. 2014, 53, 13188–13191. 10.1002/anie.201406853. 25283384

[ref378] Cai Y.; Zhu S. F.; Wang G. P.; Zhou Q. L. Iron-Catalyzed C-H Fuctionalization of Indoles. Adv. Synth. Catal. 2011, 353, 2939–2944. 10.1002/adsc.201100334.

[ref379] Gu H.; Han Z.; Xie H.; Lin X. Iron-Catalyzed Enantioselective Si-H Bond Insertions. Org. Lett. 2018, 20, 6544–6549. 10.1021/acs.orglett.8b02868. 30295494

[ref380] Li J.; Chen G.; Wang Z.; Zhang R.; Zhang X.; Ding K. Spiro-2,2′-Bichroman-Based Bisoxazoline (SPANbox) Ligands for ZnII Catalyzed Enantioselective Hydroxylation of β-Keto Esters and 1,3-Diester. Chem. Sci. 2011, 2, 1141–1144. 10.1039/c0sc00607f.

[ref381] Li J.; Pan W.; Wang Z.; Zhang X.; Ding K. Access to Both Enantiomers of α-Chloro-β-Keto Esters with a Single Chiral Ligand: Highly Efficient Enantioselective Chlorination of Cyclic β-Keto Esters Catalyzed by Chiral Copper(II) and Zinc(II) Complexes of a Spiro-2,2′-Bischroman-Based Bisoxazoline Li. Adv. Synth. Catal. 2012, 354, 1980–1986. 10.1002/adsc.201200088.

[ref382] Toussaint A.; Pfaltz A. Asymmetric Henry Reactions Catalyzed by Metal Complexes of Chiral Boron-Bridged Bisoxazoline (Borabox) Ligands. Eur. J. Org. Chem. 2008, 2008, 4591–4597. 10.1002/ejoc.200800570. 18688831

[ref383] Köhler V.; Mazet C.; Toussaint A.; Kulicke K.; Häussinger D.; Neuburger M.; Schaffner S.; Kaiser S.; Pfaltz A. Chiral Boron-Bridged Bisoxazoline (Borabox) Ligands: Structures and Reactivities of Pd and Cu Complexes. Chem. - Eur. J. 2008, 14, 8530–8539. 10.1002/chem.200800822. 18688831

[ref384] Manna K.; Xu S.; Sadow A. D. A Highly Enantioselective Zirconium Catalyst for Intramolecular Alkene Hydroamination: Significant Isotope Effects on Rate and Stereoselectivity. Angew. Chem., Int. Ed. 2011, 50, 1865–1868. 10.1002/anie.201006163. 21328658

[ref385] Manna K.; Everett W. C.; Schoendorff G.; Ellern A.; Windus T. L.; Sadow A. D. Highly Enantioselective Zirconium-Catalyzed Cyclization of Aminoalkenes. J. Am. Chem. Soc. 2013, 135, 7235–7250. 10.1021/ja4000189. 23631736

[ref386] Manna K.; Kruse M. L.; Sadow A. D. Concerted C-N/C-H Bond Formation in Highly Enantioselective Yttrium(III)-Catalyzed Hydroamination. ACS Catal. 2011, 1, 1637–1642. 10.1021/cs200511z.

[ref387] Glos M.; Reiser O. Aza-Bis(Oxazolines): New Chiral Ligands for Asymmetric Catalysis. Org. Lett. 2000, 2, 2045–2048. 10.1021/ol005947k. 10891226

[ref388] Lang K.; Park J.; Hong S. Development of Bifunctional Aza-Bis(Oxazoline) Copper Catalysts for Enantioselective Henry Reaction. J. Org. Chem. 2010, 75, 6424–6435. 10.1021/jo1009867. 20822170

[ref389] Pilsl L. K. A.; Ertl T.; Reiser O. Enantioselective Three-Step Synthesis of Homo-β-Proline: A Donor-Acceptor Cyclopropane as Key Intermediate. Org. Lett. 2017, 19, 2754–2757. 10.1021/acs.orglett.7b01111. 28485599

[ref390] McManus H. A.; Guiry P. J. Coupling of Bulky, Electron-Deficient Partners in Aryl Amination in the Preparation of Tridentate Bis(Oxazoline) Ligands for Asymmetric Catalysis. J. Org. Chem. 2002, 67, 8566–8573. 10.1021/jo0262558. 12444640

[ref391] Inagaki T.; Ito A.; Ito J. I.; Nishiyama H. Asymmetric Iron-Catalyzed Hydrosilane Reduction of Ketones: Effect of Zinc Metal upon the Absolute Configuration. Angew. Chem., Int. Ed. 2010, 49, 9384–9387. 10.1002/anie.201005363. 21053232

[ref392] Inagaki T.; Phong L. T.; Furuta A.; Ito J. I.; Nishiyama H. Iron- and Cobalt-Catalyzed Asymmetric Hydrosilylation of Ketones and Enones with Bis(Oxazolinylphenyl)Amine Ligands. Chem. - Eur. J. 2010, 16, 3090–3096. 10.1002/chem.200903118. 20119993

[ref393] Wang Y.; Wang H.; Jiang Y.; Zhang C.; Shao J.; Xu D. Fast, Solvent-Free and Highly Enantioselective Fluorination of β-Keto Esters Catalyzed by Chiral Copper Complexes in a Ball Mill. Green Chem. 2017, 19, 1674–1677. 10.1039/C6GC03306G.

[ref394] Jia Y.; Yang W.; Du D. M. Asymmetric Friedel-Crafts Alkylation of Indoles with 3-Nitro-2*H*-Chromenes Catalyzed by Diphenylamine-Linked Bis(Oxazoline) and Bis(Thiazoline) Zn(II) Complexes. Org. Biomol. Chem. 2012, 10, 4739–4746. 10.1039/c2ob25360g. 22588514

[ref395] Zhao J. Q.; Zhou X. J.; Zhou Y.; Xu X. Y.; Zhang X. M.; Yuan W. C. Diastereo- and Enantioselective Dearomative [3 + 2] Cycloaddition Reaction of 2-Nitrobenzofurans with 3-Isothiocyanato Oxindoles. Org. Lett. 2018, 20, 909–912. 10.1021/acs.orglett.7b03667. 29384383

[ref396] Zhao J. Q.; Zhou X. J.; Chen Y. Z.; Xu X. Y.; Zhang X. M.; Yuan W. C. Zn-Catalyzed Diastereo- and Enantioselective Dearomative [3 + 2] Cycloaddition Reaction of 2-Nitroindoles and 2-Nitrobenzothiophenes. Adv. Synth. Catal. 2018, 360, 2482–2487. 10.1002/adsc.201800266.

[ref397] Yue D.-F.; Zhao J.-Q.; Chen Y.-Z.; Zhang X.-M.; Xu X.-Y.; Yuan W.-C. Zinc-Catalyzed Enantioselective Dearomative [3 + 2] Cycloaddition Reaction of 3-Nitrobenzothiophenes and 3-Nitrothieno[2,3-*b*]yridine with 3-Isothiocyanato Oxindoles. Adv. Synth. Catal. 2018, 360, 1420–1425. 10.1002/adsc.201701557.

[ref398] Zhao J. Q.; Wu Z. J.; Zhou M. Q.; Xu X. Y.; Zhang X. M.; Yuan W. C. Zn-Catalyzed Diastereo- and Enantioselective Cascade Reaction of 3-Isothiocyanato Oxindoles and 3-Nitroindoles: Stereocontrolled Syntheses of Polycyclic Spirooxindoles. Org. Lett. 2015, 17, 5020–5023. 10.1021/acs.orglett.5b02489. 26412346

[ref399] Tan F.; Lu L. Q.; Yang Q. Q.; Guo W.; Bian Q.; Chen J. R.; Xiao W. J. Enantioselective Cascade Michael Addition/Cyclization Reactions of 3-Nitro-2*H*-Chromenes with 3-Isothiocyanato Oxindoles: Efficient Synthesis of Functionalized Polycyclic Spirooxindoles. Chem. - Eur. J. 2014, 20, 3415–3420. 10.1002/chem.201303583. 24677230

[ref400] Tan F.; Xiao C.; Cheng H.-G.; Wu W.; Ding K.-R.; Xiao W.-J. Enantioselective [4 + 2] Cycloadditions of 2-Vinyl-1 H-Indoles with 3-Nitro-2 *H*-Chromenes Catalyzed by a Zn(OTf)_2_/Bis(Oxazoline) Complex: An Efficient Approach to Fused Heterocycles with a Quaternary Stereocenter. Chem. - Asian J. 2012, 7, 493–497. 10.1002/asia.201100820. 22267282

[ref401] Liu H.; Lu S.; Xu J.; Du D. Asymmetric Friedel-Crafts Alkylation of Electron-Rich N-Heterocycles with Nitroalkenes Catalyzed by Diphenylamine-Tethered Bis(Oxazoline) and Bis(Thiazoline) Zn^II^ Complexes. Chem. - Asian J. 2008, 3, 1111–1121. 10.1002/asia.200800071. 18494013

[ref402] Peng J.; Du D.-M. Asymmetric Friedel-Crafts Alkylation of Indoles with Nitrodienes and 2-Propargyloxy-β-Nitrostyrenes Catalyzed by Diphenylamine-Linked Bis(Oxazoline)-Zn-(OTf)_2_ Complexes. Eur. J. Org. Chem. 2012, 2012, 4042–4051. 10.1002/ejoc.201200382.

[ref403] Despotopoulou C.; McKeon S. C.; Connon R.; Coeffard V.; Müller-Bunz H.; Guiry P. J. Application of a One-Pot Friedel-Crafts Alkylation/Michael Addition Methodology to the Asymmetric Synthesis of Ergoline Derivatives. Eur. J. Org. Chem. 2017, 2017 (45), 6734–6738. 10.1002/ejoc.201701480.

[ref404] Wang H.; Wang Y.; Zhang C.; Jiang Y.; Chu M.; Li Z.; Du X.; Xu D. Asymmetric Conjugate Additions of 2-Substituted Benzofuran-3(2*H*)-Ones to α,β-Unsaturated Ketones Catalyzed by Chiral Copper Complexes. Org. Biomol. Chem. 2017, 15, 4191–4198. 10.1039/C7OB00677B. 28443921

[ref405] O’Reilly S.; Guiry P. Recent Applications of C1-Symmetric Bis(Oxazoline)-Containing Ligands in Asymmetric Catalysis. Synthesis 2014, 46, 722–739. 10.1055/s-0033-1340829.

[ref406] Coeffard V.; Aylward M.; Guiry P. J. First Regio- and Enantioselective Chromium-Catalyzed Homoallenylation of Aldehydes. Angew. Chem., Int. Ed. 2009, 48, 9152–9155. 10.1002/anie.200903647. 19798709

[ref407] O’Reilly S.; Aylward M.; Keogh-Hansen C.; Fitzpatrick B.; McManus H. A.; Müller-Bunz H.; Guiry P. J. Synthesis of Bis(Oxazoline) Ligands Possessing C-5 Gem-Disubstitution and Their Application in Asymmetric Friedel-Crafts Alkylations. J. Org. Chem. 2015, 80, 10177–10186. 10.1021/acs.joc.5b01767. 26406290

[ref408] McKeon S. C.; Müller-Bunz H.; Guiry P. J. New Thiazoline-Oxazoline Ligands and Their Application in the Asymmetric Friedel-Crafts Reaction. Eur. J. Org. Chem. 2009, 2009, 4833–4841. 10.1002/ejoc.200900683.

[ref409] McKeon S. C.; Müller-Bunz H.; Guiry P. J. Synthesis of Thiazoline-Oxazoline Ligands and Their Application in Asymmetric Catalysis. Eur. J. Org. Chem. 2011, 2011, 7107–7115. 10.1002/ejoc.201101335.

[ref410] Hargaden G. C.; O’Sullivan T. P.; Guiry P. J. Synthesis of Non-Symmetric Bis(Oxazoline)-Containing Ligands and Their Application in the Catalytic Enantioselective Nozaki-Hiyama-Kishi Allylation of Benzaldehyde. Org. Biomol. Chem. 2008, 6, 562–566. 10.1039/B715834C. 18219428

[ref411] Suzuki T.; Kinoshita A.; Kawada H.; Nakada M. A New Asymmetric Tridentate Carbazole Ligand: Its Preparation and Application to Nozaki-Hiyama Allylation. Synlett 2003, 0570–0572. 10.1055/s-2003-37535.

[ref412] Durán-Galván M.; Connell B. T. Asymmetric Synthesis of (1,3-Butadien-2-Yl)Methanols from Aldehydes via [1-(Silylmethyl)Allenyl]Methanols. Eur. J. Org. Chem. 2010, 2010, 2445–2448. 10.1002/ejoc.201000199.

[ref413] Niwa T.; Nakada M. A Non-Heme Iron(III) Complex with Porphyrin-like Properties That Catalyzes Asymmetric Epoxidation. J. Am. Chem. Soc. 2012, 134, 13538–13541. 10.1021/ja304219s. 22861141

[ref414] Chen W.; Yang Q.; Zhou T.; Tian Q.; Zhang G. Enantioselective Synthesis of α-Exo-Methylene γ-Butyrolactones via Chromium Catalysis. Org. Lett. 2015, 17, 5236–5239. 10.1021/acs.orglett.5b02597. 26496023

[ref415] Tian Q.; Bai J.; Chen B.; Zhang G. Chromium-Catalyzed Asymmetric Dearomatization Addition Reactions of Halomethyl Heteroarenes. Org. Lett. 2016, 18, 1828–1831. 10.1021/acs.orglett.6b00559. 27043431

[ref416] Chen W.; Bai J.; Zhang G. Chromium-Catalysed Asymmetric Dearomatization Addition Reactions of Bromomethylnaphthalenes. Adv. Synth. Catal. 2017, 359, 1227–1231. 10.1002/adsc.201600962.

[ref417] Wang Z.; Ji H.; He W. M.; Xiong Y.; Zhang G. Chromium-Catalyzed Asymmetric Dearomatization-Addition Reactions of Halomethyloxazoles and Indoles. Synthesis 2018, 50, 4915–4921. 10.1055/s-0037-1609753.

[ref418] Xiong Y.; Zhang G. Enantioselective 1,2-Difunctionalization of 1,3-Butadiene by Sequential Alkylation and Carbonyl Allylation. J. Am. Chem. Soc. 2018, 140, 2735–2738. 10.1021/jacs.7b12760. 29421869

[ref419] Li W.; Hou G.; Wang C.; Jiang Y.; Zhang X. Asymmetric Hydrogenation of Ketones Catalyzed by a Ruthenium(II)-Indan-Ambox Complex. Chem. Commun. 2010, 46, 3979 10.1039/b927028k. 20407691

[ref420] Deng Q. H.; Wadepohl H.; Gade L. H. The Synthesis of a New Class of Chiral Pincer Ligands and Their Applications in Enantioselective Catalytic Fluorinations and the Nozaki-Hiyama-Kishi Reaction. Chem. - Eur. J. 2011, 17, 14922–14928. 10.1002/chem.201102375. 22052847

[ref421] Deng Q. H.; Wadepohl H.; Gade L. H. Highly Enantioselective Copper-Catalyzed Alkylation of β-Ketoesters and Subsequent Cyclization to Spirolactones/Bi-Spirolactones. J. Am. Chem. Soc. 2012, 134, 2946–2949. 10.1021/ja211859w. 22280225

[ref422] Deng Q.-H.; Wadepohl H.; Gade L. H. Highly Enantioselective Copper-Catalyzed Electrophilic Trifluoromethylation of β-Ketoesters. J. Am. Chem. Soc. 2012, 134, 10769–10772. 10.1021/ja3039773. 22693950

[ref423] Deng Q. H.; Rettenmeier C.; Wadepohl H.; Gade L. H. Copper-Boxmi Complexes as Highly Enantioselective Catalysts for Electrophilic Trifluoromethylthiolations. Chem. - Eur. J. 2014, 20, 93–97. 10.1002/chem.201303641. 24339164

[ref424] Deng Q. H.; Bleith T.; Wadepohl H.; Gade L. H. Enantioselective Iron-Catalyzed Azidation of β-Keto Esters and Oxindoles. J. Am. Chem. Soc. 2013, 135, 5356–5359. 10.1021/ja402082p. 23537339

[ref425] Bleith T.; Wadepohl H.; Gade L. H. Iron Achieves Noble Metal Reactivity and Selectivity: Highly Reactive and Enantioselective Iron Complexes as Catalysts in the Hydrosilylation of Ketones. J. Am. Chem. Soc. 2015, 137, 2456–2459. 10.1021/ja512986m. 25659289

[ref426] Wang J.; Frings M.; Bolm C. Enantioselective Nitrene Transfer to Sulfides Catalyzed by a Chiral Iron Complex. Angew. Chem., Int. Ed. 2013, 52, 8661–8665. 10.1002/anie.201304451. 23825040

[ref427] Wang J.; Frings M.; Bolm C. Iron-Catalyzed Imidative Kinetic Resolution of Racemic Sulfoxides. Chem. - Eur. J. 2014, 20, 966–969. 10.1002/chem.201303850. 24375662

[ref428] Poisson T.; Yamashita Y.; Kobayashi S. Catalytic Asymmetric Protonation of Chiral Calcium Enolates. J. Am. Chem. Soc. 2010, 2010, 7890–7892. 10.1021/ja102555a. 20481615

[ref429] Espinosa M.; Blay G.; Cardona L.; Pedro J. R. Asymmetric Conjugate Addition of Malonate Esters to α,β-Unsaturated N -Sulfonyl Imines: An Expeditious Route to Chiral δ-Aminoesters and Piperidones. Chem. - Eur. J. 2013, 19, 14861–14866. 10.1002/chem.201302687. 24105771

[ref430] Blay G.; Incerti C.; Muñoz M. C.; Pedro J. R. Enantioselective La^III^-PyBOX-Catalyzed Nitro-Michael Addition to (*E*)-2-Azachalcones. Eur. J. Org. Chem. 2013, 2013, 1696–1705. 10.1002/ejoc.201201579.

[ref431] Detz R. J.; Abiri Z.; Le Griel R.; Hiemstra H.; Van Maarseveen J. H. Enantioselective Copper-Catalysed Propargylic Substitution: Synthetic Scope Study and Application in Formal Total Syntheses of (+)-Anisomycin and (−)-Cytoxazone. Chem. - Eur. J. 2011, 17, 5921–5930. 10.1002/chem.201003727. 21500294

[ref432] Nakajima K.; Shibata M.; Nishibayashi Y. Copper-Catalyzed Enantioselective Propargylic Etherification of Propargylic Esters with Alcohols. J. Am. Chem. Soc. 2015, 137, 2472–2475. 10.1021/jacs.5b00004. 25658141

[ref433] Shibata M.; Nakajima K.; Nishibayashi Y. Enantioselective Intramolecular Propargylic Amination Using Chiral Copper-Pybox Complexes as Catalysts. Chem. Commun. 2014, 50, 7874–7877. 10.1039/C4CC01676A. 24911134

[ref434] Li R. Z.; Tang H.; Yang K. R.; Wan L. Q.; Zhang X.; Liu J.; Fu Z.; Niu D. Enantioselective Propargylation of Polyols and Desymmetrization of Meso 1,2-Diols by Copper/Borinic Acid Dual Catalysis. Angew. Chem., Int. Ed. 2017, 56, 7213–7217. 10.1002/anie.201703029. 28523904

[ref435] Hashimoto T.; Omote M.; Maruoka K. Catalytic Asymmetric Alkynylation of C1-Substituted C,N-Cyclic Azomethine Imines by CuI/Chiral Brønsted Acid Co-Catalyst. Angew. Chem., Int. Ed. 2011, 50, 8952–8955. 10.1002/anie.201104017. 21834106

[ref436] Dasgupta S.; Liu J.; Shoffler C. A.; Yap G. P. A.; Watson M. P. Enantioselective, Copper-Catalyzed Alkynylation of Ketimines to Deliver Isoquinolines with α-Diaryl Tetrasubstituted Stereocenters. Org. Lett. 2016, 18, 6006–6009. 10.1021/acs.orglett.6b02787. 27934382 PMC5161116

[ref437] Zhou F.; Tan C.; Tang J.; Zhang Y. Y.; Gao W. M.; Wu H. H.; Yu Y. H.; Zhou J. Asymmetric Copper(I)-Catalyzed Azide-Alkyne Cycloaddition to Quaternary Oxindoles. J. Am. Chem. Soc. 2013, 135, 10994–10997. 10.1021/ja4066656. 23855917

[ref438] Wang Q.; Li T.-R.; Lu L.-Q.; Li M.-M.; Zhang K.; Xiao W.-J. Catalytic Asymmetric [4 + 1] Annulation of Sulfur Ylides with Copper-Allenylidene Intermediates. J. Am. Chem. Soc. 2016, 138, 8360–8363. 10.1021/jacs.6b04414. 27355096

[ref439] Song J.; Zhang Z.-J.; Gong L.-Z. Asymmetric [4 + 2] Annulation of C1 Ammonium Enolates with Copper-Allenylidenes. Angew. Chem., Int. Ed. 2017, 56, 5212–5216. 10.1002/anie.201700105. 28370965

[ref440] Lu X.; Ge L.; Cheng C.; Chen J.; Cao W.; Wu X. Enantioselective Cascade Reaction for Synthesis of Quinolinones through Synergistic Catalysis Using Cu-Pybox and Chiral Benzotetramisole as Catalysts. Chem. - Eur. J. 2017, 23, 7689–7693. 10.1002/chem.201701741. 28425212

[ref441] Ji D.; Wang C.; Sun J. Asymmetric [4 + 2]-Cycloaddition of Copper-Allenylidenes with Hexahydro-1,3,5-Triazines: Access to Chiral Tetrahydroquinazolines. Org. Lett. 2018, 20, 3710–3713. 10.1021/acs.orglett.8b01584. 29877089

[ref442] Chen H.; Lu X.; Xia X.; Zhu Q.; Song Y.; Chen J.; Cao W.; Wu X. Asymmetric Catalytic [4 + 2] Cycloaddition via Cu-Allenylidene Intermediate: Stereoselective Synthesis of Tetrahydroquinolines Fused with a γ-Lactone Moiety. Org. Lett. 2018, 20, 1760–1763. 10.1021/acs.orglett.8b00253. 29537854

[ref443] Shao W.; You S. L. Highly Diastereo- and Enantioselective Synthesis of Tetrahydro-5*H*-Indolo[2,3-*b*]Quinolines through Copper-Catalyzed Propargylic Dearomatization of Indoles. Chem. - Eur. J. 2017, 23, 12489–12493. 10.1002/chem.201703443. 28748548

[ref444] Gómez J. E.; Guo W.; Gaspa S.; Kleij A. W. Copper-Catalyzed Synthesis of γ-Amino Acids Featuring Quaternary Stereocenters. Angew. Chem., Int. Ed. 2017, 56, 15035–15038. 10.1002/anie.201709511. 29024315

[ref445] Tian L.; Gong L.; Zhang X. Copper-Catalyzed Enantioselective Synthesis of β-Amino Alcohols Featuring Tetrasubstituted Tertiary Carbons. Adv. Synth. Catal. 2018, 360, 2055–2059. 10.1002/adsc.201701613.

[ref446] Shemet A.; Carreira E. M. Total Synthesis of (−)-Rhazinilam and Formal Synthesis of (+)-Eburenine and (+)-Aspidospermidine: Asymmetric Cu-Catalyzed Propargylic Substitution. Org. Lett. 2017, 19, 5529–5532. 10.1021/acs.orglett.7b02619. 28968107

[ref447] Osako T.; Uozumi Y. Enantioposition-Selective Copper-Catalyzed Azide-Alkyne Cycloaddition for Construction of Chiral Biaryl Derivatives. Org. Lett. 2014, 16, 5866–5869. 10.1021/ol502778j. 25360824

[ref448] Woyciechowska M.; Forcher G.; Buda S.; Mlynarski J. General Switch in Regioselectivity in the Mukaiyama Aldol Reaction of Silyloxyfuran with Aldehydes in Aqueous Solvents. Chem. Commun. 2012, 48, 11029–11031. 10.1039/c2cc36656h. 23037879

[ref449] Dudek A.; Mlynarski J. Iron-Catalyzed Asymmetric Nitro-Mannich Reaction. J. Org. Chem. 2017, 82, 11218–11224. 10.1021/acs.joc.7b01786. 28968086

[ref450] Lowicki D.; Bezlada A.; Mlynarski J. Asymmetric Hydrosilylation of Ketones Catalyzed by Zinc Acetate with Hindered Pybox Ligands. Adv. Synth. Catal. 2014, 356, 591–595. 10.1002/adsc.201300682.

[ref451] Wen H.; Wan X.; Huang Z. Asymmetric Synthesis of Silicon-Stereogenic Vinylhydrosilanes by Cobalt-Catalyzed Regio- and Enantioselective Alkyne Hydrosilylation with Dihydrosilanes. Angew. Chem., Int. Ed. 2018, 57, 6319–6323. 10.1002/anie.201802806. 29624830

[ref452] Dasgupta S.; Rivas T.; Watson M. P. Enantioselective Copper(I)-Catalyzed Alkynylation of Oxocarbenium Ions to Set Diaryl Tetrasubstituted Stereocenters. Angew. Chem., Int. Ed. 2015, 54, 14154–14158. 10.1002/anie.201507373. PMC4819992 26403641

[ref453] Murata Y.; Takahashi M.; Yagishita F.; Sakamoto M.; Sengoku T.; Yoda H. Construction of Spiro-Fused 2-Oxindole/α-Methylene- γ-Butyrolactone Systems with Extremely High Enantioselectivity via Indium-Catalyzed Amide Allylation of N-Methyl Isatin. Org. Lett. 2013, 15, 6182–6185. 10.1021/ol403014u. 24224753

[ref454] Takahashi M.; Murata Y.; Yagishita F.; Sakamoto M.; Sengoku T.; Yoda H. Catalytic Enantioselective Amide Allylation of Isatins and Its Application in the Synthesis of 2-Oxindole Derivatives Spiro-Fused to the α-Methylene-γ-Butyrolactone Functionality. Chem. - Eur. J. 2014, 20, 11091–11100. 10.1002/chem.201403357. 25049083

[ref455] Takahashi M.; Murata Y.; Ishida M.; Yagishita F.; Sakamoto M.; Sengoku T.; Yoda H. Catalytic Amide Allylation of α-Ketoesters: Extremely High Enantioselective Synthesis of Ester Functionalised α-Methylene-γ-Butyrolactones. Org. Biomol. Chem. 2014, 12, 7686–7689. 10.1039/C4OB01508H. 25167094

[ref456] Alagiri K.; Furutachi M.; Yamatsugu K.; Kumagai N.; Watanabe T.; Shibasaki M. Two Approaches toward the Formal Total Synthesis of Oseltamivir Phosphate (Tamiflu): Catalytic Enantioselective Three-Component Reaction Strategy and l-Glutamic Acid Strategy. J. Org. Chem. 2013, 78, 4019–4026. 10.1021/jo400360j. 23517385

[ref457] Marié J. C.; Xiong Y.; Min G. K.; Yeager A. R.; Taniguchi T.; Berova N.; Schaus S. E.; Porco J. A. Enantioselective Synthesis of 3,4-Chromanediones via Asymmetric Rearrangement of 3-Allyloxyflavones. J. Org. Chem. 2010, 75, 4584–4590. 10.1021/jo100889c. 20527786 PMC2896495

[ref458] Desimoni G.; Faita G.; Livieri A.; Mella M.; Ponta L.; Boiocchi M. The Asymmetric Formal Hetero-Diels-Alder Reaction of Methyl (*E*)-4-Aryl-2-Oxo-3-Butenoates Catalyzed by [Sc(OTf)_3_/Pybox] Complexes. Eur. J. Org. Chem. 2012, 2012, 2916–2928. 10.1002/ejoc.201200056.

[ref459] Karimi B.; Jafari E.; Enders D. Highly Efficient Catalytic Enantioselective Mannich Reaction of Malonates with N-Tert-Butoxycarbonyl Imines by Using Yb(OTf)_3_/Pybox Catalysts at Room Temperature. Chem. - Eur. J. 2013, 19, 10142–10145. 10.1002/chem.201300241. 23787963

[ref460] Kawatsura M.; Uchida K.; Terasaki S.; Tsuji H.; Minakawa M.; Itoh T. Ruthenium-Catalyzed Regio- and Enantioselective Allylic Amination of Racemic 1-Arylallyl Esters. Org. Lett. 2014, 16, 1470–1473. 10.1021/ol5002768. 24524275

[ref461] Shinozawa T.; Terasaki S.; Mizuno S.; Kawatsura M. Kinetic Resolution of Racemic and Branched Monosubstituted Allylic Acetates by a Ruthenium-Catalyzed Regioselective Allylic Etherification. J. Org. Chem. 2016, 81, 5766–5774. 10.1021/acs.joc.6b00939. 27276556

[ref462] de Julián E.; Menéndez-Pedregal E.; Claros M.; Vaquero M.; Díez J.; Lastra E.; Gamasa P.; Pizzano A. Practical Synthesis of Enantiopure Benzylamines by Catalytic Hydrogenation or Transfer Hydrogenation Reactions in Isopropanol Using a Ru-Pybox Catalyst. Org. Chem. Front. 2018, 5, 841–849. 10.1039/C7QO00908A.

[ref463] Arredondo V.; Hiew S. C.; Gutman E. S.; Premachandra I. D. U. A.; Van Vranken D. L. Enantioselective Palladium-Catalyzed Carbene Insertion into the N-H Bonds of Aromatic Heterocycles. Angew. Chem., Int. Ed. 2017, 56, 4156–4159. 10.1002/anie.201611845. 28295890

[ref464] Fang H.; Yang Z.; Zhang L.; Wang W.; Li Y.; Xu X.; Zhou S. Transmetal-Catalyzed Enantioselective Cross-Coupling Reaction of Racemic Secondary Benzylic Bromides with Organoaluminum Reagents. Org. Lett. 2016, 18, 6022–6025. 10.1021/acs.orglett.6b02933. 27934393

[ref465] Eno M. S.; Lu A.; Morken J. P. Nickel-Catalyzed Asymmetric Kumada Cross-Coupling of Symmetric Cyclic Sulfates. J. Am. Chem. Soc. 2016, 138, 7824–7827. 10.1021/jacs.6b03384. 27276235 PMC5539537

[ref466] Nan J.; Liu J.; Zheng H.; Zuo Z.; Hou L.; Hu H.; Wang Y.; Luan X. Direct Asymmetric Dearomatization of 2-Naphthols by Scandium-Catalyzed Electrophilic Amination. Angew. Chem., Int. Ed. 2015, 54, 2356–2360. 10.1002/anie.201409565. 25564754

[ref467] Zhao K.; Duan L.; Xu S.; Jiang J.; Fu Y.; Gu Z. Enhanced Reactivity by Torsional Strain of Cyclic Diaryliodonium in Cu-Catalyzed Enantioselective Ring-Opening Reaction. Chem. 2018, 4, 599–612. 10.1016/j.chempr.2018.01.017.

[ref468] Xu S.; Zhao K.; Gu Z. Copper-Catalyzed Asymmetric Ring-Opening of Cyclic Diaryliodonium with Benzylic and Aliphatic Amines. Adv. Synth. Catal. 2018, 360, 3877–3883. 10.1002/adsc.201800637.

[ref469] Wang D.-C.; Xie M.-S.; Guo H.-M.; Qu G.-R.; Zhang M.-C.; You S.-L. Enantioselective Dearomative [3 + 2] Cycloaddition Reactions of Benzothiazoles. Angew. Chem., Int. Ed. 2016, 55, 14111–14115. 10.1002/anie.201607852. 27723190

[ref470] Ruiz Espelt L.; McPherson I. S.; Wiensch E. M.; Yoon T. P. Enantioselective Conjugate Additions of α-Amino Radicals via Cooperative Photoredox and Lewis Acid Catalysis. J. Am. Chem. Soc. 2015, 137, 2452–2455. 10.1021/ja512746q. 25668687 PMC4547529

[ref471] Blum T. R.; Miller Z. D.; Bates D. M.; Guzei I. A.; Yoon T. P. Enantioselective Photochemistry through Lewis Acid-Catalyzed Triplet Energy Transfer. Science 2016, 354, 1391–1395. 10.1126/science.aai8228. 27980203 PMC5501084

[ref472] Miller Z. D.; Lee B. J.; Yoon T. P. Enantioselective Crossed Photocycloadditions of Styrenic Olefins by Lewis Acid Catalyzed Triplet Sensitization. Angew. Chem., Int. Ed. 2017, 56, 11891–11895. 10.1002/anie.201706975. PMC5661956 28776908

[ref473] Pericas L.; Shafir A.; Vallribera A. Asymmetric Synthesis of L-Carbidopa Based on a Highly Enantioselective α-Amination. Org. Lett. 2013, 15, 1448–1451. 10.1021/ol400136y. 23477289

[ref474] Granados A.; del Olmo A.; Peccati F.; Billard T.; Sodupe M.; Vallribera A. Fluorous L-Carbidopa Precursors: Highly Enantioselective Synthesis and Computational Prediction of Bioactivity. J. Org. Chem. 2018, 83, 303–313. 10.1021/acs.joc.7b02685. 29200295

[ref475] Suga H.; Hashimoto Y.; Yasumura S.; Takezawa R.; Itoh K.; Kakehi A. Chiral Lewis Acid Catalyzed Asymmetric Cycloadditions of Carbonyl Ylides Generated from Diazoimide Derivatives and Their Synthetic Applications to Indolizidine Alkaloids. J. Org. Chem. 2013, 78, 10840–10852. 10.1021/jo401837d. 24099422

[ref476] Shen H. C.; Wu Y. F.; Zhang Y.; Fan L. F.; Han Z. Y.; Gong L. Z. Palladium-Catalyzed Asymmetric Aminohydroxylation of 1,3-Dienes. Angew. Chem., Int. Ed. 2018, 57, 2372–2376. 10.1002/anie.201712350. 29336513

[ref477] Tsuchida K.; Senda Y.; Nakajima K.; Nishibayashi Y. Construction of Chiral Tri- and Tetra-Arylmethanes Bearing Quaternary Carbon Centers: Copper-Catalyzed Enantioselective Propargylation of Indoles with Propargylic Esters. Angew. Chem., Int. Ed. 2016, 55, 9728–9732. 10.1002/anie.201604182. 27346363

[ref478] Xie Z.; Liu X.; Liu L. Copper-Catalyzed Aerobic Enantioselective Cross-Dehydrogenative Coupling of N-Aryl Glycine Esters with Terminal Alkynes. Org. Lett. 2016, 18, 2982–2985. 10.1021/acs.orglett.6b01328. 27269737

[ref479] Zhao J. F.; Tan B. H.; Zhu M. K.; Tjan T. B. W.; Loh T. P. Enantioselective Carbonyl-Ene Reactions of Trifluoropyruvate in Ionic Liquid via a Recyclable Indium(III)-Pybox Complex. Adv. Synth. Catal. 2010, 352, 2085–2088. 10.1002/adsc.201000170.

[ref480] Zhao Y. J.; Li B.; Tan L. J. S.; Shen Z. L.; Loh T. P. Enantioselective Cationic Polyene Cyclization vs Enantioselective Intramolecular Carbonyl-Ene Reaction. J. Am. Chem. Soc. 2010, 132, 10242–10244. 10.1021/ja104119j. 20614887

[ref481] Zhu D.; Chen L.; Zhang H.; Ma Z.; Jiang H.; Zhu S. Highly Chemo- and Stereoselective Catalyst-Controlled Allylic C-H Insertion and Cyclopropanation Using Donor/Donor Carbenes. Angew. Chem., Int. Ed. 2018, 57, 12405–12409. 10.1002/anie.201805676. 30059187

[ref482] Zhao B.; Loh T. P. Asymmetric Hetero-Diels-Alder Reaction of Danishefsky’s Dienes with α-Carbonyl Esters Catalyzed by an Indium(III)-PyBox Complex. Org. Lett. 2013, 15, 2914–2917. 10.1021/ol400841s. 23738771

[ref483] Hanhan N. V.; Sahin A. H.; Chang T. W.; Fettinger J. C.; Franz A. K. Catalytic Asymmetric Synthesis of Substituted 3-Hydroxy-2oxindoles. Angew. Chem., Int. Ed. 2010, 49, 744–747. 10.1002/anie.200904393. 20029857

[ref484] Gutierrez E. G.; Wong C. J.; Sahin A. H.; Franz A. K. Enantioselective and Regioselective Indium(III)-Catalyzed Addition of Pyrroles to Isatins. Org. Lett. 2011, 13, 5754–5757. 10.1021/ol202329s. 21992567 PMC3235954

[ref485] Hanhan N. V.; Tang Y. C.; Tran N. T.; Franz A. K. Scandium(III)-Catalyzed Enantioselective Allylation of Isatins Using Allylsilanes. Org. Lett. 2012, 14, 2218–2221. 10.1021/ol300496v. 22506841

[ref486] Hanhan N. V.; Ball-Jones N. R.; Tran N. T.; Franz A. K. Catalytic Asymmetric [3 + 2] Annulation of Allylsilanes with Isatins: Synthesis of Spirooxindoles. Angew. Chem., Int. Ed. 2012, 51, 989–992. 10.1002/anie.201105739. 22162028

[ref487] Ball-Jones N. R.; Badillo J. J.; Tran N. T.; Franz A. K. Catalytic Enantioselective Carboannulation with Allylsilanes. Angew. Chem., Int. Ed. 2014, 53, 9462–9465. 10.1002/anie.201403607. PMC4795922 25045133

[ref488] Duan Z.; Han J.; Qian P.; Zhang Z.; Wang Y.; Pan Y. Enantioselective Synthesis of 3-Hydroxy Oxindoles by Ytterbium-Catalysed Decarboxylative Addition of β-Ketoacids to Isatins. Org. Biomol. Chem. 2013, 11, 6456–6459. 10.1039/c3ob41460d. 23979548

[ref489] Prakash M.; Kesavan V. Highly Enantioselective Synthesis of 2,3-Dihydroquinazolinones through Intramolecular Amidation of Imines. Org. Lett. 2012, 14, 1896–1899. 10.1021/ol300518m. 22458670

[ref490] Rout S.; Das A.; Singh V. K. Metal-Controlled Switching of Enantioselectivity in the Mukaiyama-Michael Reaction of α,β-Unsaturated 2-Acyl Imidazoles Catalyzed by Chiral Metal-Pybox Complexes. J. Org. Chem. 2018, 83, 5058–5071. 10.1021/acs.joc.8b00399. 29658718

[ref491] Rout S.; Das A.; Singh V. K. An Asymmetric Vinylogous Mukaiyama-Michael Reaction of α,β-Unsaturated 2-Acyl Imidazoles Catalyzed by Chiral Sc(III)- or Er(III)-Pybox Complexes. Chem. Commun. 2017, 53, 5143–5146. 10.1039/C7CC01763D. 28435957

[ref492] Shimizu S.; Tsubogo T.; Xu P.; Kobayashi S. Calcium-Catalyzed Asymmetric Synthesis of 3-Tetrasubstituted Oxindoles: Efficient Construction of Adjacent Quaternary and Tertiary Chiral Centers. Org. Lett. 2015, 17, 2006–2009. 10.1021/acs.orglett.5b00749. 25849712

[ref493] Ray S. K.; Singh P. K.; Molleti N.; Singh V. K. Enantioselective Synthesis of Coumarin Derivatives by PYBOX-DIPH-Zn(II) Complex Catalyzed Michael Reaction. J. Org. Chem. 2012, 77, 8802–8808. 10.1021/jo301513x. 22957472

[ref494] Ray S. K.; Rout S.; Singh V. K. Enantioselective Synthesis of 3,4-Dihydropyran Derivatives via a Michael Addition Reaction Catalysed by Chiral Pybox-Diph-Zn(II) Complex. Org. Biomol. Chem. 2013, 11, 2412 10.1039/c3ob40246k. 23471180

[ref495] Rout S.; Ray S. K.; Singh V. K. Enantioselective Mukaiyama-Michael with 2-Enoyl Pyridine N-Oxides Catalyzed by PYBOX-DIPH-Zn(II)-Complexes at Ambient Temperature. Org. Biomol. Chem. 2013, 11, 4537–4545. 10.1039/c3ob40445e. 23722314

[ref496] Bisai V.; Suneja A.; Singh V. K. Asymmetric Alkynylation/Lactamization Cascade: An Expeditious Entry to Enantiomerically Enriched Isoindolinones. Angew. Chem., Int. Ed. 2014, 53, 10737–10741. 10.1002/anie.201405074. 25146686

[ref497] Suneja A.; Bisai V.; Singh V. K. Asymmetric Syntheses of Medicinally Important Isoindolinones (*S*)-PD 172938, (*R*)-JM 1232, and Related Structures. J. Org. Chem. 2016, 81, 4779–4788. 10.1021/acs.joc.6b00770. 27148957

[ref498] Lee J. Y.; You Y. S.; Kang S. H. Asymmetric Synthesis of All-Carbon Quaternary Stereocenters via Desymmetrization of 2,2-Disubstituted 1,3-Propanediols. J. Am. Chem. Soc. 2011, 133, 1772–1774. 10.1021/ja1103102. 21265528

[ref499] Parsons A. T.; Johnson J. S. Catalytic Enantioselective Synthesis of Tetrahydrofurans: A Dynamic Kinetic Asymmetric [3 + 2] Cycloaddition of Racemic Cyclopropanes and Aldehydes. J. Am. Chem. Soc. 2009, 131, 3122–3123. 10.1021/ja809873u. 19256562

[ref500] Parsons A. T.; Smith A. G.; Neel A. J.; Johnson J. S. Dynamic Kinetic Asymmetric Synthesis of Substituted Pyrrolidines from Racemic Cyclopropanes and Aldimines: Reaction Development and Mechanistic Insights. J. Am. Chem. Soc. 2010, 132, 9688–9692. 10.1021/ja1032277. 20572661

[ref501] Wales S. M.; Walker M. M.; Johnson J. S. Asymmetric Synthesis of Indole Homo-Michael Adducts via Dynamic Kinetic Friedel-Crafts Alkylation with Cyclopropanes. Org. Lett. 2013, 15, 2558–2561. 10.1021/ol4010646. 23654283

[ref502] Amador A. G.; Sherbrook E. M.; Yoon T. P. Enantioselective Photocatalytic [3 + 2] Cycloadditions of Aryl Cyclopropyl Ketones. J. Am. Chem. Soc. 2016, 138, 4722–4725. 10.1021/jacs.6b01728. 27015009 PMC5070469

[ref503] Gao X. T.; Gan C. C.; Liu S. Y.; Zhou F.; Wu H. H.; Zhou J. Utilization of CO_2_ as a C_1_ Building Block in a Tandem Asymmetric A3 Coupling-Carboxylative Cyclization Sequence to 2-Oxazolidinones. ACS Catal. 2017, 7, 8588–8593. 10.1021/acscatal.7b03370.

[ref504] Da B. C.; Liang Q. J.; Luo Y. C.; Ahmad T.; Xu Y. H.; Loh T. P. Copper-Catalyzed Stereo- and Enantioselective 1,4-Protosilylation of α,β-Unsaturated Ketimines to Synthesize Functionalized Allylsilanes. ACS Catal. 2018, 8, 6239–6245. 10.1021/acscatal.8b01547.

[ref505] Ghoshal A.; Sarkar A. R.; Manickam G.; Kumaran R. S.; Jayashankaran J. Rhodium-Catalyzed Asymmetric Hydrosilylation of Ketones Employing a New Ligand Embodying the Bis(Oxazolinyl)Pyridine Moiety. Synlett 2010, 2010, 1459–1462. 10.1055/s-0029-1219949.

[ref506] Nishiyama H.; Ito J. Bis(Oxazolinyl)Phenyl Transition-Metal Complexes: Asymmetric Catalysis and Some Reactions of the Metals. Chem. Commun. 2010, 46, 203–212. 10.1039/B918923H. 20024328

[ref507] Shiomi T.; Adachi T.; Ito J. I.; Nishiyama H. Intermolecular Antiselective and Enantioselective Reductive Coupling of Enones and Aromatic Aldehydes with Chiral Rh(Phebox) Catalysts. Org. Lett. 2009, 11, 1011–1014. 10.1021/ol802939u. 19161317

[ref508] Itoh K.; Tsuruta A.; Ito J. I.; Yamamoto Y.; Nishiyama H. Enantioselective Synthesis of Optically Active 3,3-Diarylpropanoates by Conjugate Hydrosilylation with Chiral Rh-Bis(Oxazolinyl)Phenyl Catalysts. J. Org. Chem. 2012, 77, 10914–10919. 10.1021/jo302357b. 23140754

[ref509] Naganawa Y.; Kawagishi M.; Ito J. I.; Nishiyama H. Asymmetric Induction at Remote Quaternary Centers of Cyclohexadienones by Rhodium-Catalyzed Conjugate Hydrosilylation. Angew. Chem., Int. Ed. 2016, 55, 6873–6876. 10.1002/anie.201601636. 27100774

[ref510] Shiomi T.; Adachi T.; Toribatake K.; Zhou L.; Nishiyama H. Asymmetric β-Boration of α,β-Unsaturated Carbonyl Compounds Promoted by Chiral Rhodium-Bisoxazolinylphenyl Catalysts. Chem. Commun. 2009, (40), 5987–5989. 10.1039/b915759j. 19809619

[ref511] Toribatake K.; Nishiyama H. Asymmetric Diboration of Terminal Alkenes with a Rhodium Catalyst and Subsequent Oxidation: Enantioselective Synthesis of Optically Active 1,2-Diols. Angew. Chem., Int. Ed. 2013, 52, 11011–11015. 10.1002/anie.201305181. 24000239

[ref512] Smith J. R.; Collins B. S. L.; Hesse M. J.; Graham M. A.; Myers E. L.; Aggarwal V. K. Enantioselective Rhodium(III)-Catalyzed Markovnikov Hydroboration of Unactivated Terminal Alkenes. J. Am. Chem. Soc. 2017, 139, 9148–9151. 10.1021/jacs.7b05149. 28665124 PMC5515510

[ref513] Ito J. I.; Ujiie S.; Nishiyama H. New Bis (Oxazolinyl)Phenyl-Ruthenium (II) Complexes and Their Catalytic Activity for Enantioselective Hydrogenation and Transfer Hydrogenation of Ketones. Organometallics 2009, 28, 630–638. 10.1021/om800953f.

[ref514] Ito J. I.; Ujiie S.; Nishiyama H. A New NCN Pincer Ruthenium Complex and Its Catalytic Activity for Enantioselective Hydrogenation of Ketones. Chem. Commun. 2008, (16), 1923–1925. 10.1039/b800387d. 18401520

[ref515] Ito J. I.; Teshima T.; Nishiyama H. Enhancement of Enantioselectivity by Alcohol Additives in Asymmetric Hydrogenation with Bis(Oxazolinyl)Phenyl Ruthenium Catalysts. Chem. Commun. 2012, 48, 1105–1107. 10.1039/C1CC16057E. 22080393

[ref516] Ito J. I.; Asai R.; Nishiyama H. Asymmetric Direct Alkynylation Catalyzed by Chiral Ru-Bis(Oxazolinyl)Phenyl Complexes. Org. Lett. 2010, 12, 3860–3862. 10.1021/ol1015338. 20698490

[ref517] Ito J. I.; Fujii K.; Nishiyama H. Direct Conjugate Addition of Alkynes with α,β-Unsaturated Carbonyl Compounds Catalyzed by NCN-Pincer Ru Complexes. Chem. - Eur. J. 2013, 19, 601–605. 10.1002/chem.201203380. 23180508

[ref518] Ubukata S.; Ito J. I.; Oguri R.; Nishiyama H. Asymmetric Three-Component Coupling Reaction of Alkyne, Enone, and Aldehyde Catalyzed by Chiral Phebox Ruthenium Catalysts. J. Org. Chem. 2016, 81, 3347–3355. 10.1021/acs.joc.6b00374. 27008318

[ref519] Ohshima T.; Kawabata T.; Takeuchi Y.; Kakinuma T.; Iwasaki T.; Yonezawa T.; Murakami H.; Nishiyama H.; Mashima K. C1-Symmetric Rh/Phebox-Catalyzed Asymmetric Alkynylation of α-Ketoesters. Angew. Chem., Int. Ed. 2011, 50, 6296–6300. 10.1002/anie.201100252. 21626620

[ref520] Morisaki K.; Sawa M.; Nomaguchi J. Y.; Morimoto H.; Takeuchi Y.; Mashima K.; Ohshima T. Rh-Catalyzed Direct Enantioselective Alkynylation of α-Ketiminoesters. Chem. - Eur. J. 2013, 19, 8417–8420. 10.1002/chem.201301237. 23670946

[ref521] Morisaki K.; Sawa M.; Yonesaki R.; Morimoto H.; Mashima K.; Ohshima T. Mechanistic Studies and Expansion of the Substrate Scope of Direct Enantioselective Alkynylation of α-Ketiminoesters Catalyzed by Adaptable (Phebox)Rhodium(III) Complexes. J. Am. Chem. Soc. 2016, 138, 6194–6203. 10.1021/jacs.6b01590. 27092817

[ref522] Ito J. I.; Ujiie S.; Nishiyama H. Chiral Bis(Oxazolinyl)Phenyl RuII Catalysts for Highly Enantioselective Cyclopropanation. Chem. - Eur. J. 2010, 16, 4986–4990. 10.1002/chem.200903514. 20340118

[ref523] Owens C. P.; Varela-Álvarez A.; Boyarskikh V.; Musaev D. G.; Davies H. M. L.; Blakey S. B. Iridium(III)-Bis(Oxazolinyl)Phenyl Catalysts for Enantioselective C-H Functionalization. Chem. Sci. 2013, 4, 2590 10.1039/c3sc50886b. PMC6005272 29997805

[ref524] Itoh K.; Sibi M. P. Dibenzofuran-4,6-Bis(Oxazoline) (DBFOX). A Novel Trans -Chelating Bis(Oxazoline) Ligand for Asymmetric Reactions. Org. Biomol. Chem. 2018, 16, 5551–5565. 10.1039/C8OB01010B. 29947634

[ref525] Huang Y.; Tokunaga E.; Suzuki S.; Shiro M. Enantioselective Friedel-Crafts Reaction of -Trifluoromethylated Acrylates with Pyrroles and Its. Org. Lett. 2010, 86, 2008–2010. 10.1021/ol100171z 20143845

[ref526] Huang Y.; Suzuki S.; Liu G.; Tokunaga E.; Shiro M.; Shibata N. Asymmetric Synthesis of Chiral Trifluoromethylated Heliotridane via Highly Catalytic Asymmetric Friedel-Crafts Alkylation with β-Trifluoromethylated Acrylates and Pyrroles. New J. Chem. 2011, 35, 2614–2621. 10.1039/c1nj20550a.

[ref527] Murarka S.; Deb I.; Zhang C.; Seidel D. Catalytic Enantioselective Intramolecular Redox Reactions: Ring-Fused Tetrahydroquinolines. J. Am. Chem. Soc. 2009, 131, 13226–13227. 10.1021/ja905213f. 19711900

[ref528] Qiu J. S.; Wang Y. F.; Qi G. R.; Karmaker P. G.; Yin H. Q.; Chen F. X. Highly Enantioselective α-Cyanation with 4-Acetylphenyl Cyanate. Chem. - Eur. J. 2017, 23, 1775–1778. 10.1002/chem.201605610. 27917550

[ref529] Shibata N.; Ishimaru T.; Reddy D. S.; Horikawa T.; Nakamura S.; Toru T. DBFOX-Ph/Metal Complexes: Evaluation as Catalysts for Enantioselective Fluorination of 3-(2-Arylacetyl)-2-Thiazolidinones. Beilstein J. Org. Chem. 2008, 4, 55–57. 10.3762/bjoc.4.16. PMC2486487 18941488

[ref530] Reddy D. S.; Shibata N.; Horikawa T.; Suzuki S.; Nakamura S.; Toru T.; Shiro M. A DBFOX-Ph-Based Combinatorial Catalyst for Enantioselective Fluorination of Aryl Acetyl and 3-Butenoyl Thiazolidinones. Chem. - Asian J. 2009, 4, 1411–1415. 10.1002/asia.200900164. 19557785

[ref531] Reddy D. S.; Shibata N.; Nagai J.; Nakamura S.; Toru T.; Kanemasa S. Desymmetrization-like Catalytic Enantioselective Fluorination of Malonates and Its Application to Pharmaceutically Attractive Molecules. Angew. Chem., Int. Ed. 2008, 47, 164–168. 10.1002/anie.200704093. 17997510

[ref532] Reddy D. S.; Shibata N.; Nagai J.; Nakamura S.; Toru T. A Dynamic Kinetic Asymmetric Transformation in the α-Hydroxylation of Racemic Malonates and Its Application to Biologically Active Molecules. Angew. Chem., Int. Ed. 2009, 48, 803–806. 10.1002/anie.200804476. 19101968

[ref533] Sibi M. P.; Coulomb J.; Stanley L. M. Enantioselective Enolate Protonations: Friedel-Crafts Reactions with α-Substituted Acrylates. Angew. Chem., Int. Ed. 2008, 47, 9913–9915. 10.1002/anie.200804221. 19016291

[ref534] Banerjee B.; Capps S. G.; Kang J.; Robinson J. W.; Castle S. L. Second-Generation DBFOX Ligands for the Synthesis of β-Substituted α-Amino Acids via Enantioselective Radical Conjugate Additions. J. Org. Chem. 2008, 73, 8973–8978. 10.1021/jo801721z. 18947256 PMC2627584

[ref535] Yang X.; Cheng F.; Kou Da Y.; Pang S.; Shen Y. C.; Huang Y. Y.; Shibata N. Catalytic Asymmetric 1,3-Dipolar Cycloaddition of β-Fluoroalkylated α,β-Unsaturated 2-Pyridylsulfones with Nitrones for Chiral Fluoroalkylated Isoxazolidines and γ-Amino Alcohols. Angew. Chem., Int. Ed. 2017, 56, 1510–1514. 10.1002/anie.201610605. 28067017

[ref536] Shen X.; Li Y.; Wen Z.; Cao S.; Hou X.; Gong L. A Chiral Nickel DBFOX Complex as a Bifunctional Catalyst for Visible-Light-Promoted Asymmetric Photoredox Reactions. Chem. Sci. 2018, 9, 4562–4568. 10.1039/C8SC01219A. 29899949 PMC5969497

[ref537] Lu S. M.; Gao Q.; Li J.; Liu Y.; Li C. A Robust Ru-PNNP Catalyst System for the Asymmetric Hydrogenation of α,β-Unsaturated Ketones to Allylic Alcohol. Tetrahedron Lett. 2013, 54, 7013–7016. 10.1016/j.tetlet.2013.10.051.

[ref538] Dai W.; Li G.; Chen B.; Wang L.; Gao S. A Porphyrin-Inspired Iron Catalyst for Asymmetric Epoxidation of Electron-Deficient Olefins. Org. Lett. 2015, 17, 904–907. 10.1021/acs.orglett.5b00018. 25643130

[ref539] Matsumura Y.; Suzuki T.; Sakakura A.; Ishihara K. Catalytic Enantioselective Inverse Electron Demand Hetero-Diels-Alder Reaction with Allylsilanes. Angew. Chem., Int. Ed. 2014, 53, 6131–6134. 10.1002/anie.201402934. 24782343

[ref540] Sakakura A.; Kondo R.; Matsumura Y.; Akakura M.; Ishihara K. Rational Design of Highly Effective Asymmetric Diels-Alder Catalysts Bearing 4,4-Sulfonamidomethyl Groups. J. Am. Chem. Soc. 2009, 131, 17762–17764. 10.1021/ja906098b. 19924906

[ref541] Perrotta D.; Wang M. M.; Waser J. Lewis Acid Catalyzed Enantioselective Desymmetrization of Donor-Acceptor Meso-Diaminocyclopropanes. Angew. Chem., Int. Ed. 2018, 57, 5120–5123. 10.1002/anie.201800494. 29461662

[ref542] Shao Q.; Wu L.; Chen J.; Gridnev I. D.; Yang G.; Xie F.; Zhang W. Copper (II)/RuPHOX-Catalyzed Enantioselective Mannich-Type Reaction of Glycine Schiff Bases with Cyclic Ketimines. Adv. Synth. Catal. 2018, 360, 4625–4633. 10.1002/adsc.201800850.

[ref543] An Q.; Liu D.; Shen J.; Liu Y.; Zhang W. The Construction of Chiral Fused Azabicycles Using a Pd-Catalyzed Allylic Substitution Cascade and Asymmetric Desymmetrization Strategy. Org. Lett. 2017, 19, 238–241. 10.1021/acs.orglett.6b03529. 28009523

[ref544] Huo X.; Yang G.; Liu D.; Liu Y.; Gridnev I. D.; Zhang W. Palladium-Catalyzed Allylic Alkylation of Simple Ketones with Allylic Alcohols and Its Mechanistic Study. Angew. Chem., Int. Ed. 2014, 53, 6776–6780. 10.1002/anie.201403410. 24848670

[ref545] Huo X.; Quan M.; Yang G.; Zhao X.; Liu D.; Liu Y.; Zhang W. Hydrogen-Bond-Activated Palladium-Catalyzed Allylic Alkylation via Allylic Alkyl Ethers: Challenging Leaving Groups. Org. Lett. 2014, 16, 1570–1573. 10.1021/ol5000988. 24621181

[ref546] Ma Y.; Li J.; Ye J.; Liu D.; Zhang W. Synthesis of Chiral Chromanols: Via a RuPHOX-Ru Catalyzed Asymmetric Hydrogenation of Chromones. Chem. Commun. 2018, 54, 13571–13574. 10.1039/C8CC07787H. 30444507

[ref547] Dai W.; Li J.; Li G.; Yang H.; Wang L.; Gao S. Asymmetric Epoxidation of Alkenes Catalyzed by a Porphyrin-Inspired Manganese Complex. Org. Lett. 2013, 15, 4138–4141. 10.1021/ol401812h. 23947820

[ref548] Dai W.; Shang S.; Chen B.; Li G.; Wang L.; Ren L.; Gao S. Asymmetric Epoxidation of Olefins with Hydrogen Peroxide by an in Situ-Formed Manganese Complex. J. Org. Chem. 2014, 79, 6688–6694. 10.1021/jo501178k. 24969226

[ref549] Dai W.; Li J.; Chen B.; Li G.; Lv Y.; Wang L.; Gao S. Asymmetric Oxidation Catalysis by a Porphyrin-Inspired Manganese Complex: Highly Enantioselective Sulfoxidation with a Wide Substrate Scope. Org. Lett. 2013, 15, 5658–5661. 10.1021/ol402612x. 24156512

[ref550] Dai W.; Mi Y.; Lv Y.; Chen B.; Li G.; Chen G.; Gao S. Development of a Continuous-Flow Microreactor for Asymmetric Sulfoxidation Using a Biomimetic Manganese Catalyst. Adv. Synth. Catal. 2016, 358, 667–671. 10.1002/adsc.201501023.

[ref551] Ficks A.; Sibbald C.; John M.; Dechert S.; Meyer F. Dinuclear Allylpalladium Complexes of C2-Symmetric Pyrazolate-Bridged Bis(Oxazoline) Ligands (Pyrbox’s): Structures, Dynamic Behavior, and Application in Asymmetric Allylic Alkylation. Organometallics 2010, 29, 1117–1126. 10.1021/om900835f.

[ref552] Jalba A.; Régnier N.; Ollevier T. Enantioselective Aromatic Sulfide Oxidation and Tandem Kinetic Resolution Using Aqueous H 2 O 2 and Chiral Iron-Bis(Oxazolinyl)Bipyridine Catalysts. Eur. J. Org. Chem. 2017, 2017, 1628–1637. 10.1002/ejoc.201601597.

[ref553] Zhou Y. Y.; Sun X. L.; Zhu B. H.; Zheng J. C.; Zhou J. L.; Tang Y. Modification of Pseudo-C3-Symmetric Trisoxazoline and Its Application to the Friedel-Crafts Alkylation of Indoles and Pyrrole with Alkylidene Malonates. Synlett 2011, 2011, 935–938. 10.1055/s-0030-1259721.

[ref554] Wang P.; Tao W. J.; Sun X. L.; Liao S.; Tang Y. A Highly Efficient and Enantioselective Intramolecular Cannizzaro Reaction under TOX/Cu(II) Catalysis. J. Am. Chem. Soc. 2013, 135, 16849–16852. 10.1021/ja409859x. 24161001

[ref555] Zhou Y. Y.; Li J.; Ling L.; Liao S. H.; Sun X. L.; Li Y. X.; Wang L. J.; Tang Y. Highly Enantioselective [3 + 3] Cycloaddition of Aromatic Azomethine Imines with Cyclopropanes Directed by π-π Stacking Interactions. Angew. Chem., Int. Ed. 2013, 52, 1452–1456. 10.1002/anie.201207576. 23281073

[ref556] Ren H.; Song X. Y.; Wang S. R.; Wang L.; Tang Y. Highly Enantioselective Nickel-Catalyzed Oxa-[3 + 3]-Annulation of Phenols with Benzylidene Pyruvates for Chiral Chromans. Org. Lett. 2018, 20, 3858–3861. 10.1021/acs.orglett.8b01442. 29888607

[ref557] Cao P.; Deng C.; Zhou Y. Y.; Sun X. L.; Zheng J. C.; Xie Z.; Tang Y. Asymmetric Nazarov Reaction Catalyzed by Chiral Tris(Oxazoline)/ Copper(II). Angew. Chem., Int. Ed. 2010, 49, 4463–4466. 10.1002/anie.200907266. 20480470

[ref558] Zhou Y. Y.; Wang L. J.; Li J.; Sun X. L.; Tang Y. Side-Arm-Promoted Highly Enantioselective Ring-Opening Reactions and Kinetic Resolution of Donor-Acceptor Cyclopropanes with Amines. J. Am. Chem. Soc. 2012, 134, 9066–9069. 10.1021/ja302691r. 22578301

[ref559] Kang Q. K.; Wang L.; Liu Q. J.; Li J. F.; Tang Y. Asymmetric H2O-Nucleophilic Ring Opening of D-A Cyclopropanes: Catalyst Serves as a Source of Water. J. Am. Chem. Soc. 2015, 137, 14594–14597. 10.1021/jacs.5b10310. 26540202

[ref560] Xu H.; Hu J. L.; Wang L.; Liao S.; Tang Y. Asymmetric Annulation of Donor-Acceptor Cyclopropanes with Dienes. J. Am. Chem. Soc. 2015, 137, 8006–8009. 10.1021/jacs.5b04429. 26068395

[ref561] Liao S.; Sun X. L.; Tang Y. Side Arm Strategy for Catalyst Design: Modifying Bisoxazolines for Remote Control of Enantioselection and Related. Acc. Chem. Res. 2014, 47, 2260–2272. 10.1021/ar800104y. 24837859

[ref562] Li W. J.; Qiu S. X. A Novel D2-Symmetrical Chiral Tetraoxazoline Ligand for Highly Enantioselective Hydrosilylation of Aromatic Ketones. Adv. Synth. Catal. 2010, 352, 1119–1122. 10.1002/adsc.201000018.

[ref563] Torres M.; Maisse-François A.; Bellemin-Laponnaz S. Highly Recyclable Self-Supported Chiral Catalysts for the Enantioselective α-Hydrazination of β-Ketoesters. ChemCatChem 2013, 5, 3078–3085. 10.1002/cctc.201300395.

